# Chemodivergent aminocarbonylation enabled by oxygen vacancy–engineered Pd-doped In_2_O_3_ nanocatalysts

**DOI:** 10.1126/sciadv.aef9067

**Published:** 2026-06-26

**Authors:** Shujuan Liu, Teng Li, Kang Zhao, Chunhui Liu, Matthias Beller, Xinjiang Cui

**Affiliations:** ^1^ State Key Laboratory of Low Carbon Catalysis and Carbon Dioxide Utilization, Lanzhou Institute of Chemical Physics, Chinese Academy of Sciences, No. 18, Tianshui Middle Road, Lanzhou 730000, China.; ^2^ University of Chinese Academy of Sciences, No. 19A, Yuquan Road, Beijing 100049, China.; ^3^ Leibniz-Institut für Katalyse e.V. an der Universität Rostock, Rostock 18059, Germany.; ^4^ Joint Center of LICP-LIKAT for Synergies in Molecular- and Nano-Catalysis, No. 18, Tianshui Middle Road, Lanzhou 730000, China.

## Abstract

The amide bond is a crucial structural motif in numerous bioactive natural products and pharmaceutical compounds. Mono- (MC) and double-aminocarbonylation (DC) are key methodologies for synthesizing aryl amides, including versatile α-ketoamides. However, selectively controlling MC and DC of aryl halides has been a long-standing challenge. Here, we report a previously unknown strategy to fully invert the selectivity from MC to DC in palladium (Pd)–doped indium(III) oxide nanocatalysts by introducing oxygen vacancies (O_v_) to modulate the second-beyond coordination spheres (SBCSs) of Pd, and catalysts show excellent activity, selectivity, and reusability for synthesizing diverse amides and α-ketoamides (130 examples). SBCS modulation drives long-range electron transfer to fine-tune electron localization, elongating Pd-O bonds, and weakening their strength to promote the second carbon monoxide adsorption and insertion for the DC pathway.

## Teaser

Oxygen vacancies switch aminocarbonylation selectivity on Pd-doped In_2_O_3_ for efficient amide and α-ketoamide synthesis.

## INTRODUCTION

Aryl amides are crucial functional compounds found in a wide range of bioactive molecules, including peptides, natural proteins, DNA, RNA, and vitamins. In addition, they are integral to the pharmaceutical industry, being present in numerous active pharmaceutical ingredients (*1*–*4*). According to the Njarðarson Laboratory’s analysis, 64 of the top 200 global small-molecule drugs by retail sales in 2024 contain one or more aryl amides (*5*). Hence, the efficient synthesis of aryl amides has long been a key focus of synthetic chemistry. These compounds, including versatile α-ketoamides, are commonly synthesized via carbonylation reactions of aryl halides using carbon monoxide (CO) as a readily available C1 synthon, through mono-aminocarbonylation (MC) and double-aminocarbonylation (DC) pathways (*6*–*9*). However, controlling the selectivity between MC and DC reactions remains a substantial challenge, and general strategies to achieve this selectivity are still lacking.

**Fig. 1.**
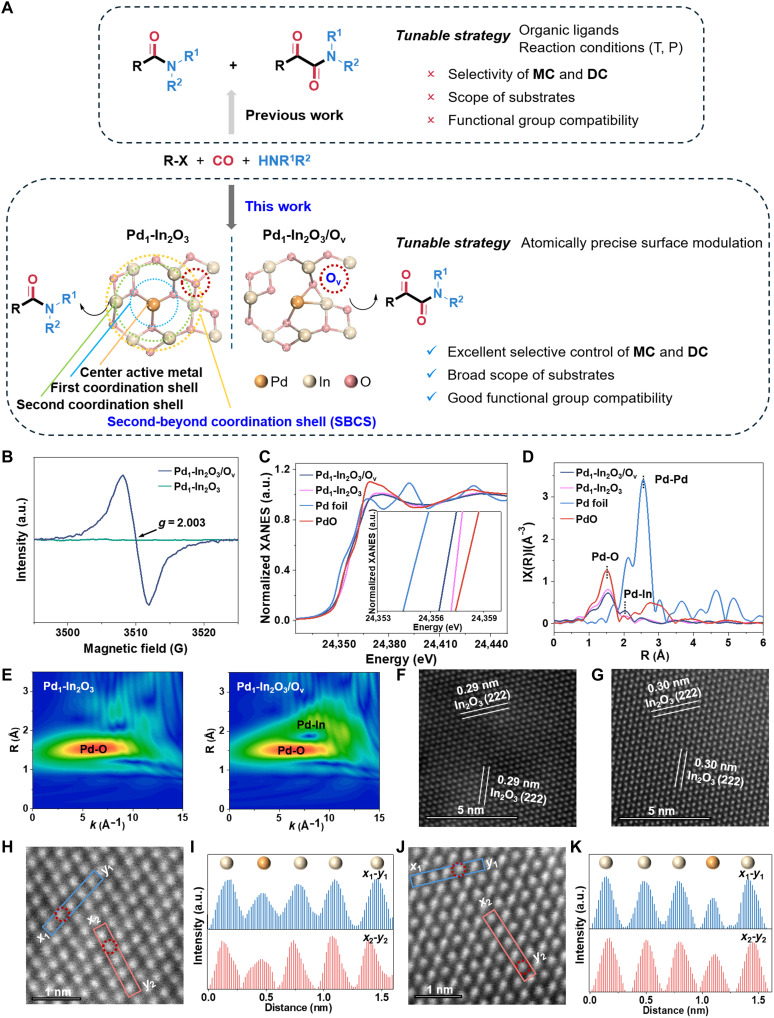
Characterization of Pd_1_-In_2_O_3_ and Pd_1_-In_2_O_3_/O_v_. (**A**) The tunable strategies of selectivity for aminocarbonylation of organic halides. (**B**) EPR spectra of Pd_1_-In_2_O_3_ and Pd_1_-In_2_O_3_/O_v_. Normalized XANES spectra (**C**), FT-EXAFS spectra (**D**), and wavelet transform EXAFS plots (**E**) at the Pd K-edges of Pd foil, PdO, Pd_1_-In_2_O_3_, and Pd_1_-In_2_O_3_/O_v_. (**F** and **G**) HAADF-STEM images of Pd_1_-In_2_O_3_ and Pd_1_-In_2_O_3_/O_v_. (**H**) AC-HAADF-STEM image of Pd_1_-In_2_O_3_, where the weakly intense Pd atoms are indicated by red circles. (**I**) The line scan measured along the *x*-*y* rectangle region marked in (H). (**J**) AC-HAADF-STEM image and of Pd_1_-In_2_O_3_/O_v_, where the weakly intense Pd atoms are indicated by red circles. (**K**) The line scan measured along the *x*-*y* rectangle region marked in (J). a.u., arbitrary units.

The selectivity of transition metal–catalyzed MC and DC depends on multiple factors (*10*). Recent studies have shown that controlling the oxidation state of catalysts, such as copper, through ligand choice or alkyl halide types can influence the secondary CO insertion (DC) or direct nucleophilic trapping (MC) of acyl radicals (*11*). In addition, adjusting the coordination strength between the solvent and the central atom has been found to influence CO insertion, enabling control over MC and DC reactions (*12*, *13*). Despite these advances, strategies to control MC and DC selectivity through simple variations in the active site structure of heterogeneous catalysts remain rare (Fig. 1A) (*14*–*18*). Notably, from a molecular point of view, selectivity in catalytic reactions can be achieved by strategically steering reactants to traverse the potential energy surface toward a specific geometry (*19*, *20*). Most of such efforts to date have focused on modulating the electronic and geometric properties of the first coordination sphere, with little attention given to the second-beyond coordination spheres (SBCSs) (*21*–*24*). However, the modification of binding pocket in the SBCS can finely tailor the steric and charge distribution at active sites, and it is highly desirable to explore the influence of active sites with well-defined and precisely regulated local coordination structure by SBCS on the selectivity. We hypothesized that modifying the SBCS could offer an avenue for controlling the selectivity of carbonylation catalysts, enabling the precise tuning of MC and DC pathways (*25*). To explore this concept, herein, we developed a previously unknown approach using isolated palladium (Pd) centers anchored onto In_2_O_3_ nanocrystals (Pd_1_-In_2_O_3_), which only modulated SBCS by oxygen vacancies (O_v_) to Pd_1_-In_2_O_3_ (denoted as Pd_1_-In_2_O_3_/O_v_), resulting in a dramatic switch of the reaction selectivity from MC to DC (Fig. 1A). Characterizations and density functional theory (DFT) calculations show that SBCS on the Pd_1_-In_2_O_3_/O_v_ surface tuned the coordination microenvironment of active sites, facilitating the adsorption and insertion into Pd–C bond of the second CO, and achieving a completely reverse selectivity. Overall, this study provides a viable strategy to control selectivity in catalytic reactions by the well-defined and tunable SBCS.

## RESULTS

### Synthesis and characterization of catalysts

The combination of annealing treatment atmosphere and organic solvent impregnation strategy was used to synthesize the pure and O_v_-rich materials, denoted Pd_1_-In_2_O_3_ and Pd_1_-In_2_O_3_/O_v_, respectively (fig. S1A), which was normally used to prepare O_v_ and single-metal atoms (*26*, *27*). In_2_O_3_ is a reducible metal oxide with a controllable O_v_ density, which can modulate active sites with different coordination numbers (CNs) and electronic structures (*28*). The x-ray diffraction (XRD) patterns showed cubic In_2_O_3_ (no. 06-0416) and no obvious Pd crystalline phase (*27*); the diffraction peaks of the Pd_1_-In_2_O_3_/O_v_ sample shifted slightly to lower angles (fig. S1B). Meanwhile, the stretching vibration of In-O-In at 361.1 cm^−1^ in Raman spectra was confirmed to reflect the degree of O_v_, and Pd_1_-In_2_O_3_/O_v_ sample showed red-shifted and broadened peaks, indicating the formation of O_v_ (fig. S1C) (*29*, *30*). In addition, the scattering feature of In-O vibration at 130.1 cm^−1^ was visibly shifted toward lower frequencies in Pd_1_-In_2_O_3_/O_v_ due to the addition of Pd (fig. S1, D and E) (*31*). Electron paramagnetic resonance (EPR) spectra demonstrated that weak signal was detected for the Pd_1_-In_2_O_3_, while an asymmetric signal of *g* = 2.003 was clearly observed for Pd_1_-In_2_O_3_/O_v_, indicating a much higher O_v_ concentration (Fig. 1B) (*32*). Compared with Pd 3d of x-ray photoelectron spectroscopy (XPS) in Pd_1_-In_2_O_3_, the positively charged Pd^δ+^ was notably shifted toward lower binding energy in Pd_1_-In_2_O_3_/O_v_ (336.3 versus 335.5) (*33*), which might be attributed to the creation of nonadjacent O_v_ that redistributes the long-range electrons to Pd sites (fig. S1F), which was consistent with DFT calculations, and an electron-adequate character of Bader charge compared with Pd_1_-In_2_O_3_ was presented (fig. S1G). A similar shift of energy positions was observed in their normalized Pd K-edge X-ray absorption near-edge structure (XANES) spectrum (Fig. 1C). The Pd atoms mainly existed as Pd^δ+^ species according to their binding energies of XPS spectra and their close Pd-K XANES absorption edge as the PdO reference (*31*). Furthermore, the Fourier-transformed extended x-ray absorption fine structure (EXAFS; Fig. 1D) spectrum showed a dominant feature centred at 1.5 Å, which was ascribed to the first shell of the Pd–O bond, and a weak peak at around 2.1 Å was speculated to be Pd–In bond formed in Pd_1_-In_2_O_3_/O_v_ (*34*). In addition, no signals corresponding to Pd-Pd coordination were detected, further substantiating the presence of Pd as atomically dispersed species in the catalysts (*35*). Moreover, EXAFS fitting analysis displayed that the CN of Pd–O in Pd_1_-In_2_O_3_ was 2.8, directly connected with three O atoms. Also, the CN of Pd–O in Pd_1_-In_2_O_3_/O_v_ was 2.4, and that of Pd-In was 0.8, indicating that connected with two O atoms and one In atom (fig. S1, H and I, and table S1), the total CN remained unchanged, indicating that the O_v_ was nonadjacent to Pd sites. Furthermore, wavelet transform EXAFS also exhibited a maximum at 1.5 Å, assigned to Pd-O coordination and the presence of Pd-In species for the Pd_1_-In_2_O_3_/O_v_ (Fig. 1E). Aberration-corrected high-angle annular dark-field scanning transmission electron microscopy (AC-HAADF-STEM) exhibited the increase of lattice parameters in Pd_1_-In_2_O_3_/O_v_ than Pd_1_-In_2_O_3_ (Fig. 1, F and G) and the substitution of Pd for In atoms on Pd_1_-In_2_O_3_ and Pd_1_-In_2_O_3_/O_v_ (Fig. 1, H to K). On the basis of characterizations, DFT calculations were conducted to afford a model structure, where Pd substitutes at In lattice sites on the In_2_O_3_(111) surface in both Pd_1_-In_2_O_3_ and Pd_1_-In_2_O_3_/O_v_. The Pd_1_-In_2_O_3_/O_v_ has configuration containing Pd center and O_v_ on SBCS, which is rarely reported as active site in heterogeneous catalysis.

### Model reaction, selectivity studies, and mechanistic investigations

**Fig. 2.**
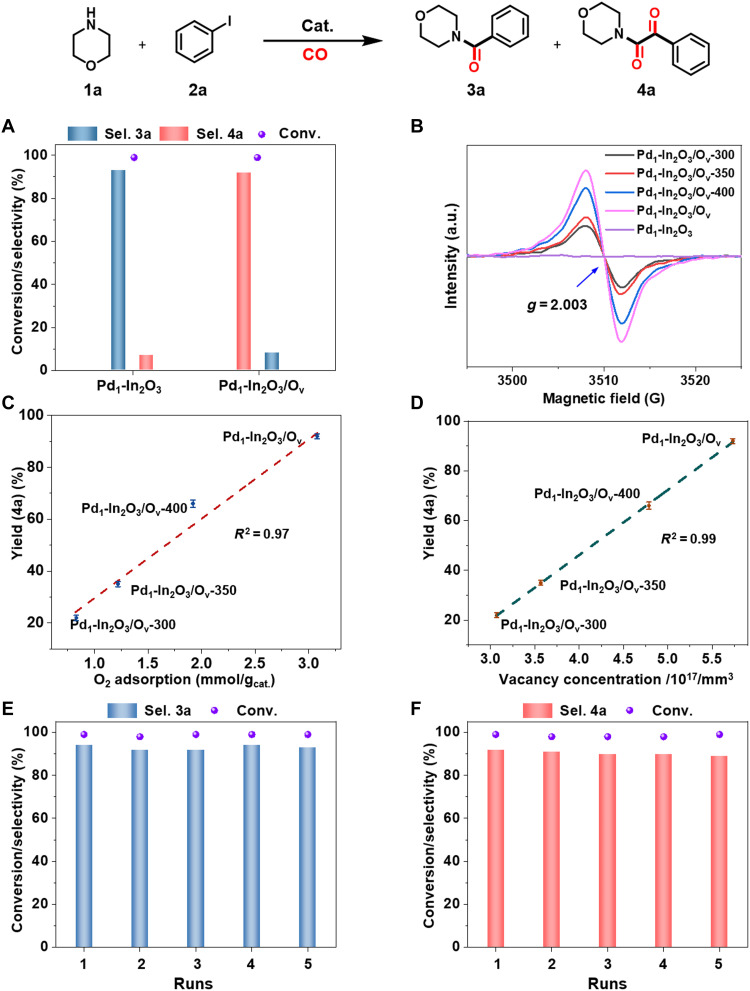
Catalytic performance assessment for aminocarbonylation of iodobenzene. (**A**) The catalytic performance of Pd_1_-In_2_O_3_ and Pd_1_-In_2_O_3_/O_v_. (**B**) EPR spectra of In_2_O_3_ support, which underwent pretreatment with H_2_ at temperatures of 300°, 350°, and 400°C. (**C**) The linear relationship between O_2_ pulses and yield of **4a**. (**D**) The linear relationship between the concentration of oxygen vacancies determined by EPR and yield of **4a**. (**E** and **F**) Stability and recycling tests using Pd_1_-In_2_O_3_ and Pd_1_-In_2_O_3_/O_v_. Reaction conditions: 1 mmol iodobenzene, 1.5 mmol morpholine, 20 mg of catalyst, 2.0 equiv. K_2_CO_3_, 10 bar CO, 3 ml of 1,4-dioxane, 125°C, 9 hours.

4-Benzoylmorpholine (3a) and 4-(phenylglyoxylyl)morpholine (4a) are important chemicals and widely applied in drugs or organic semiconductor modifications (*36*, *37*). In addition, with its moderate nucleophilicity and steric constraints, morpholine provides an optimal platform for assessing catalytic carbonylation activity and facilitating cross-amine comparisons. Conversely, α-ketoamide formation presents a more challenging test of chemoselectivity, offering a rigorous standard for catalyst evaluation. Therefore, the catalytic selectivity of Pd_1_-In_2_O_3_ and Pd_1_-In_2_O_3_/O_v_ catalysts was chosen using the carbonylation of iodobenzene and morpholine as the model reaction. The Pd content was 0.16 wt % for Pd_1_-In_2_O_3_ and 0.17 wt % for Pd_1_-In_2_O_3_/O_v_ samples (table S2, entries 1 and 2). A series of experiments for the aminocarbonylation by Pd_1_-In_2_O_3_ and Pd_1_-In_2_O_3_/O_v_ was conducted (table S3). Under the same reaction conditions, the selectivity of 3a over Pd_1_-In_2_O_3_ was determined to be 93%, while the selectivity of 4a was 92% by Pd_1_-In_2_O_3_/O_v_ (Fig. 2A), indicating the tunability of SBCS played an important role for 4a formation. Under identical conditions, commercial Pd/C exhibited mixture of products, and the selectivities of 70% for 3a and 30% for 4a (table S3, entry 13). Compared with previously reported results, this catalytic system demonstrated superior selectivity for both 3a and 4a (table S4).

To elucidate the SBCS-mediated change of selectivity for the aminocarbonylation, Pd_1_-In_2_O_3_/O_v_ was treated by O_2_ to annihilate O_v_, denoted Pd_1_-In_2_O_3_/O_v_-O_2_. The selectivity of 4a dropped dramatically from 92% in Pd_1_-In_2_O_3_/O_v_ to 11% in Pd_1_-In_2_O_3_/O_v_-O_2_ (table S3, entry 14), which was similar with the Pd_1_-In_2_O_3_ catalyst (7% selectivity for 4a). In addition, EPR spectra showed that weak signal of O_v_ was detected for the Pd_1_-In_2_O_3_/O_v_-O_2_ (fig. S2A), indicating that the decreased selectivity of 4a is attributed to the decline of O_v_ concentration. To further investigate the effects of the O_v_ concentration on the catalytic performance of DC, the In_2_O_3_ support underwent pretreatment with H_2_ at temperatures of 300°, 350°, 400°, and 500°C (the temperature used for target Pd_1_-In_2_O_3_/O_v_ catalyst) to modulate the O_v_ concentrations (Fig. 2B). Next, the quantitative relationship between the O_v_ concentration and catalytic performance was explored by O_2_ pulse and EPR measurements. As shown in Fig. 2 (C and D) and table S5, the yield of 4a proportionally increased with the O_v_ concentration, peaking at 92% for the Pd_1_-In_2_O_3_/O_v_ catalyst. This observation shows the importance of modulating SBCS on the catalytic selectivity of DC. To further explore the relationship between O_v_ concentration and intrinsic catalytic performance (conversion lower than 20%), a linear correlation was observed between O_v_ concentration and the yield of 4a, intrinsic catalytic performance (conversion of 2a) showed no substantial variation (Fig. S3), demonstrating that the electronic modulation derived from SBCS primarily tuned the reaction selectivity rather than governing the intrinsic reactivity. Further, the In_2_O_3_ support was reduced at 600°C to get greater O_v_ concentrations and higher DC yields but found that it completely converted to metallic indium, which fundamentally altered the nature of the support. Therefore, the reduction temperature for In_2_O_3_ was limited to 500°C.

To gain insight into the origin of activity for the aminocarbonylation reaction, activity tests were conducted over Pd_1_-In_2_O_3_ and Pd_1_-In_2_O_3_/O_v_ (fig. S4). The results showed that all reactants exhibited zero-order kinetics during the initial reaction stage at conversions below 25%, indicating that the catalyst surface was saturated with reactants and that the reaction was controlled by the surface reaction step, which was a typical and reasonable kinetic behavior in heterogeneous catalysis (fig. S4, A to C and E to G). The activation energies were obtained from the slopes of Arrhenius plots by linearly fitting ln(*k*) (where *k* is the rate constant) against 1/T × 1000. On the basis of these plots, the experimentally estimated apparent activation energies were 91.23 and 93.99 kJ/mol for Pd_1_-In_2_O_3_ and Pd_1_-In_2_O_3_/O_v_, respectively (fig. S4, D and H). Isotope-labeling experiments with ^13^CO were conducted to trace the origin of the carbonyl groups in mono- and double-carbonylation products and to verify the sequential CO insertion pathway (fig. S5). For the mono-carbonylation product, High-resolution mass spectrometry (HRMS) analysis confirmed the incorporation of a single ^13^C-labeled carbonyl group, consistent with one round of CO insertion (fig. S5A). For the double-carbonylation product, two ^13^C-labeled carbonyl groups were unambiguously detected, confirming the occurrence of a second CO insertion leading to the double-carbonylation product (fig. S5B).

Besides, excellent catalytic stabilities of Pd_1_-In_2_O_3_ and Pd_1_-In_2_O_3_/O_v_ were also witnessed in the recycling and hot filtration experiments (Fig. 2, E and F, and tables S6 and S7). Next, we performed the characterization of the spent Pd_1_-In_2_O_3_ and Pd_1_-In_2_O_3_/O_v_ catalysts (fig. S6 and table S2, entries 3 and 4). In agreement with the observed stability of the prepared catalyst materials, the structures of the spent and fresh catalysts exhibited no notable changes. The reaction solution after recycling and hot filtration tests using inductively coupled plasma optical emission spectrometry (ICP-OES) were also conducted, and the results showed that the Pd contents were below the detection limit of the instrument, indicating that no leaching of Pd occurred during the reaction, confirming the stability of catalysts. In addition, air sensitivity for Pd_1_-In_2_O_3_/O_v_ was also examined, and the catalyst retained activity and selectivity after being stored under ambient air for 7 days (0.2%Pd_1_-In_2_O_3_/O_v_, 7 days) (table S3, entry 15) and exhibited only a 1% decrease in O_v_ concentration (as determined by XPS, fig. S2B), indicating that the catalyst of Pd_1_-In_2_O_3_/O_v_ was air stable. To assess the scalability of catalysts, the gram-synthesized catalysts were tested under the same conditions and exhibited identical performance and selectivity compared to the small-scale synthesis (table S3, entries 16 and 17), which confirmed the great potential of catalysts for industrial applications.

The SBCS impact on the aminocarbonylation mechanism was explored using in situ diffuse reflectance infrared Fourier transform spectroscopy (DRIFTS, fig. S7, A and B). A key intermediate of PhCO* (1572 cm^−1^) (*38*), which was formed during the CO insertion elementary reaction, appeared on both catalysts of Pd_1_-In_2_O_3_ and Pd_1_-In_2_O_3_/O_v_ after exposure with CO at 70°C. Meanwhile, the formation of another important intermediate PhCOCO* (1646 cm^−1^) for 4a was observed exclusively through the DC process. As the reaction proceeded, the signal intensity of three bands at 1628, 1675, and 1747 cm^−1^ increased. These bands were ascribed to the stretching vibration of -C=O- of the target products 3a and 4a, respectively. For CO adsorbed on Pd_1_-In_2_O_3_, the spectra exhibited a single peak, centered at 2014 cm^−1^, which is identical to the linear CO adsorption. In contrast, the Pd_1_-In_2_O_3_/O_v_ sample exhibited two additional peaks located at 1867 and 1940 cm^−1^. These latter peaks can be assigned to the bridge-bonded CO adsorption on Pd and In sites, suggesting that In atoms are directly involved in the reaction.

**Fig. 3.**
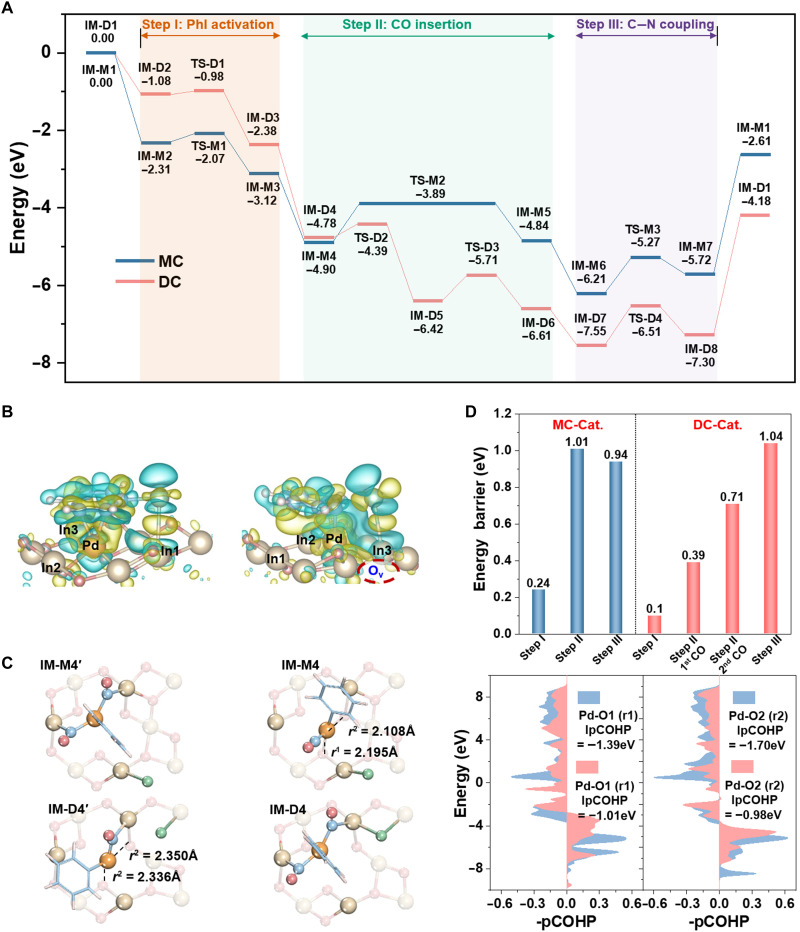
DFT calculations of the model reaction. (**A**) Energy profiles of PhI aminocarbonylation over Pd_1_-In_2_O_3_ (MC) and Pd_1_-In_2_O_3_/O_v_ (DC) surface. (**B**) Differential charge density plots (isosurface value of 0.001 eV/Å^3^; cyan, charge depletion; yellow, charge accumulation) of PhI on Pd_1_-In_2_O_3_ (left) and Pd_1_-In_2_O_3_/O_v_ (right) (side view). (**C**) Structural and bonding (projected crystal orbital Hamilton populations) analysis between the Pd and subsurface oxygen atoms for IM-4 and IM-4′. (**D**) Energy barriers comparison for the aminocarbonylation of iodobenzene by Pd_1_-In_2_O_3_ and Pd_1_-In_2_O_3_/O_v_.

To further shed light on the mechanisms of selectivity inversion from MC to DC, DFT calculations were performed on Pd-doped In_2_O_3_(111) without O_v_ (Pd_1_-In_2_O_3_, MC-Cat) and with O_v_ (Pd_1_-In_2_O_3_/O_v_, DC-Cat). Aminocarbonylation of PhI and HNR^1^R^2^ [morpholine, HN(CH_2_CH_2_)_2_O] was chosen as the model reaction, which was divided into three elementary reaction steps (*38*, *39*) (fig. S8, A and B) on both MC-Cat and DC-Cat: PhI activation (step I), CO insertion (step II), and C–N coupling (step III). In addition, the related potential energy surfaces and structures were enclosed in Fig. 3A and fig. S8 (C and D).

In step I (PhI activation), PhI preferred to adsorb at the Pd-In1 site (IM-M1 → IM-M2) and subsequently broke its C–I bond with an energy barrier of 0.24 eV (IM-M2 → TS-M1 → IM-M3) on MC-Cat. The energy barrier was reduced to 0.10 eV (IM-D2 → TS-D1 → IM-D3) when PhI dissociation occurred at the Pd-In3 site (IM-D1 → IM-D2) with the assistance of O_v_ on DC-Cat, which resulted in the elongation of C–I bond from 2.123 Å in IM-M2 to 2.224 Å in IM-D2 and greater electron depletion of C–I bond according to the charge density difference analysis (Fig. 3B).

With Ph and I group on surface, only one CO molecule could adhere to Pd atom (IM-M3 → IM-M4) on MC-Cat, but two CO molecules were adsorbed at the Pd-In3 and Pd-In2 sites gradually (IM-D3 → IM-D4) on DC-Cat. Such difference was caused by local structural deformation and Pd–O bond breaking between Pd and subsurface oxygen (Pd–O1 and Pd–O2) aroused by the second CO adsorption. According to Fig. 3C, with one CO on surface, Pd–O1 and Pd–O2 bonds in IM-M4′ showed shorter bond lengths and stronger bond strengths [lower values of IpCOHP (Integrated projected Crytsal Oribital Hamilton Population)] than those in IM-D4′. These two factors (local structural deformation and Pd-O bond breaking) enhanced the difficulty and positive adsorption energy for the second CO adsorption on MC-Cat. Therefore, CO insertion (step II) on MC-Cat only contained one elementary reaction (IM-M4 → TS-M2 → IM-M5), and the energy barrier was 1.01 on Pd-In2 site, while two CO on DC-Cat were inserted into Pd–C bond stepwise to form PhCO (IM-D4 → TS-D2 → IM-D5) on Pd-In3 site and PhCOCO (IM-D5 → TS-D3 → IM-D6) on Pd-In2 site; the energy barriers were 0.39 and 0.71 eV, lower than that (1.01 eV) for step II on MC-Cat, respectively. After the exchange between surface I atom (from PhI dissociation) and NR^1^R^2^ (IM-M5 → IM-M6 on MC-Cat, IM-D6 → IM-D7 on DC-Cat), C–N coupling (step III) occurred at Pd-In3 site on MC-Ca and Pd-In1 site on DC-Cat. The accompanied energy barriers were 0.94 and 1.04 eV. With the desorption of PhCONR^1^R^2^ and PhCOCONR^1^R^2^, the surfaces were recovered to original structures of IM-M1 and IM-D1.

According to Fig. 3A, which included energy variations of optimal paths for PhCONR_1_R_2_ and PhCOCONR_1_R_2_ formation, CO insertion (step II) exhibited higher energy barrier than PhI activation and C–N coupling on MC-Cat (1.01 versus 0.24, 0.94 eV), which was the rate-determining step (RDS), while C–N coupling (step III) was the RDS when compared with other three reactions (1.04 versus 0.10, 0.39, 0.71 eV) on DC-Cat (Fig. 3D). In addition, CO insertion occupying higher energy barrier than PhI activation on both MC-Cat and DC-Cat was in agreement with that on homogeneous Pd(PPh_3_)_2_ (*40*) and heterogeneous Fe_2_O_3_-O_vac_ (*38*). Furthermore, the calculated activation energies were 97.43 and 100.34 KJ/mol on MC-Cat and DC-Cat, respectively, which are almost identical to the experimental estimated apparent activation energies (91.23 and 93.99 kJ/mol), demonstrating the excellent agreement between theory and experiment and supporting the DFT computed reaction mechanism. On the basis of DFT results, the regulation of SBCS was beneficial for PhI activation, second CO adsorption, RDS shift, and finally to achieve the selectivity control of MC and DC.

### Exploration of synthetic scope and generality

**Fig. 4.**
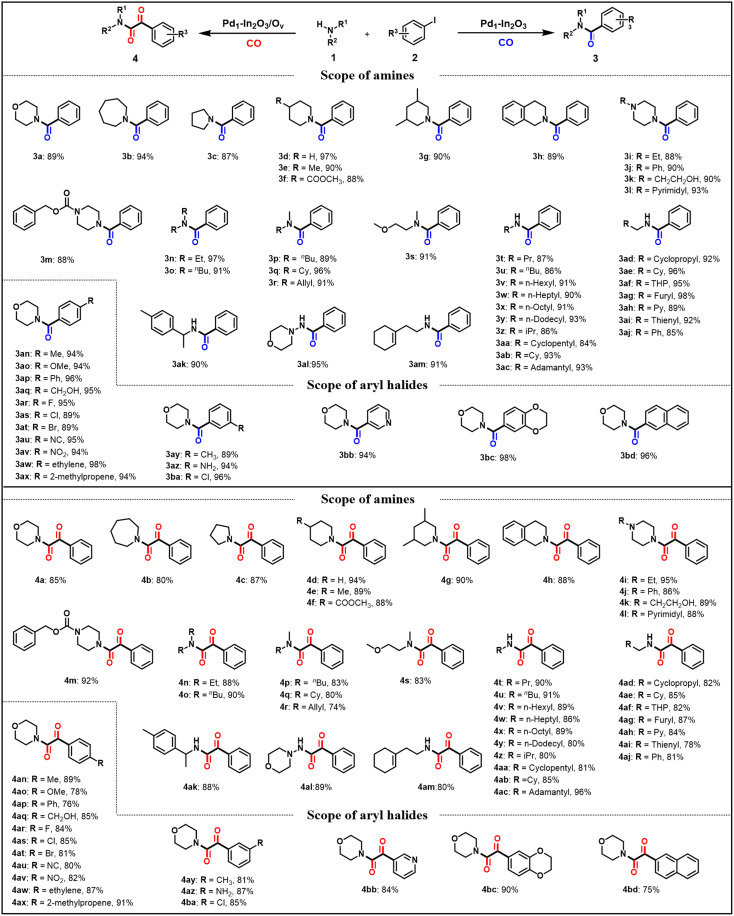
Substrate scope of aminocarbonylation. Reaction conditions: aryl halide (1.0 mmol), amines (1.5 mmol), Cat (20 mg), CO (1 MPa), K_2_CO_3_ (2.0 equiv.), 1,4-dioxane (2.0 mL), 24 hours, isolated yields.

With the optimized reaction conditions established, we explored the scope and functional group compatibility of the aminocarbonylation of various aryl halides to yield the corresponding amides and α-ketoamides using Pd_1_-In_2_O_3_ and Pd_1_-In_2_O_3_/O_v_, respectively. As shown in Fig. 4, the reaction was compatible with a variety of cyclic secondary amines, including morpholine, azepane, pyrrolidine, piperidine, and functionalized piperazines, producing the desired mono- (MC) and double- (DC) carbonylation products with yields ranging from 88 to 97% and 80 to 95%, respectively (3a-3m and 4a-4m). Secondary amines bearing alkyl, vinyl, and ether functional groups were also efficiently converted into the corresponding products, with yields of up to 97% for MC and 90% for DC (3n-3s and 4n-4s). Aliphatic primary amines and cyclic methylamines underwent smooth transformation to the corresponding amides (86 to 96% yields; 3t-3af) and α-ketoamides (80 to 96% yields; 4t-4af).

Furthermore, (hetero)aromatic methylamines and functionalized primary amines provided excellent to very good yields (85 to 98%) of the MC products (3ag-3am) and 80 to 89% yields of the DC products (4ag-4am). We further attempted to access α-ketoamides from aromatic amines; however, only mono-carbonylation products were observed (tables S8 and S9, entries 1 to 3), which can be rationalized by its notably lower nucleophilicity relative to aliphatic and cyclic amines, hindering the subsequent nucleophilic attack. We also examined a range of (hetero)aryl halides with various electronic and steric properties. These widely available coupling partners afforded a broad spectrum of valuable products in good to excellent yields (3an-3bd and 4an-4bd). Both electron-donating and electron-withdrawing substituents, such as halides, ethers, nitriles, nitro groups, amino groups, and heterocycles, were well tolerated, highlighting the flexibility of the catalytic system for further synthetic manipulations. In addition, alkenyl iodides reacted smoothly to give the desired products in good to excellent yields (3aw-3ax and 4aw-4ax). Unfortunately, ortho-substituted aryl iodides demonstrated poor selectivity for the synthesis of α-ketoamides due to steric hindrance (tables S8 and S9, entries 6 to 10).

**Fig. 5.**
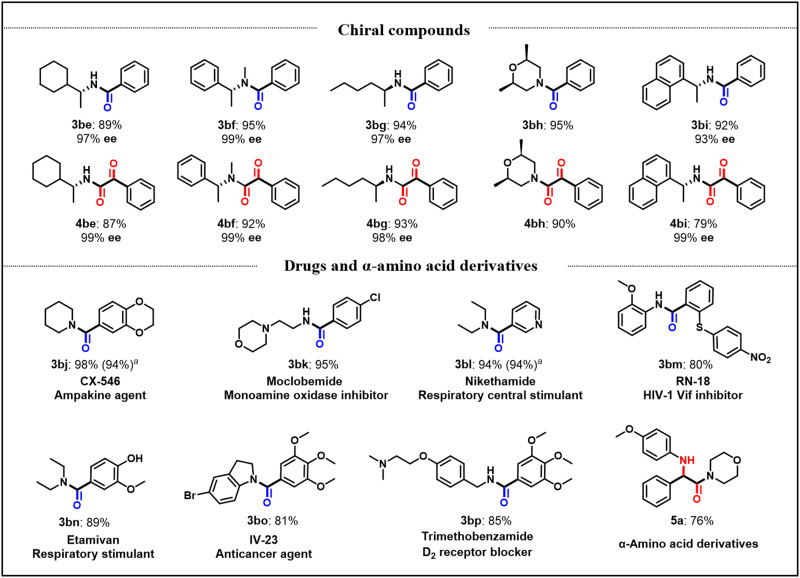
Synthesis of chiral compounds, drugs, and α-amino acid derivatives. Reaction conditions: aryl halide (1.0 mmol), amines (1.5 mmol), Cat (20 mg), CO (1 MPa), K_2_CO_3_ (2.0 equiv.), 1,4-dioxane (2.0 ml), 24 hours, isolated yields; *^a^*aryl halide (10.0 mmol), amines (15.0 mmol), Cat (100 mg), CO (1 MPa), K_2_CO_3_ (2.0 equiv.), 1,4-dioxane (5.0 ml), 48 hours, isolated yields.

In comparison to conventional homogeneous and heterogeneous catalytic systems (*12*, *17*), our Pd_1_-In_2_O_3_ and Pd_1_-In_2_O_3_/O_v_ catalysts exhibit exceptional selectivity for DC reactions, particularly with aliphatic primary amines, cyclic methylamines, and electron-withdrawing aryl halides. The broader substrate applicability and robust functional group tolerance of these catalysts further enhance their utility. Notably, six new α-ketoamide compounds (4k, 4s, 4w, 4al, 4aq, and 4az) were synthesized, none of which have been previously reported in the literature. The Pd_1_-In_2_O_3_ and Pd_1_-In_2_O_3_/O_v_ catalyst systems were also tested with chiral amines. As shown in Fig. 5, both primary and secondary chiral amines underwent smooth aminocarbonylation to deliver the corresponding products with yields ranging from 89 to 95% for MC products (3be-3bi) and 79 to 93% for DC products (4be-4bi). While the chiral center was not directly involved in the synthesis of chemical bond, the goal of this work was to determine whether the aminocarbonylation reaction could proceed without inducing racemization at the stereocenter. The complete preservation of enantiomeric excess (ee) throughout the transformation confirmed that our method operated under mild, racemization-free conditions, which is crucial for applications in complex molecule synthesis. To demonstrate the potential of this protocol for applications in life sciences, we explored the synthesis of several pharmaceuticals via aminocarbonylation. Notably, drugs such as CX-546, moclobemide, nikethamide, RN-18, etamivan, IV-23, and trimethobenzamide were synthesized in a single step with excellent yields (3bj-3bp). Further, we successfully scaled up the reaction to gram level for both CX-546 and nikethamide, which proceeded smoothly to afford the desired products in 94% yields, highlighting the practical applicability of this method in pharmaceutical synthesis. Last, from a synthetic point of view, it is worth mentioning that obtained α-ketoamides can be further converted in highly valuable α-amino carbonyl derivatives (*41*, *42*). As an example, 5a was prepared in 76% yield.

## DISCUSSION

In summary, we have developed Pd-doped indium oxide catalysts with precisely modulated coordination spheres. By tailoring the microenvironment around the atomically dispersed Pd centers, we can modulate the coordination sphere of the active sites, which in turn controls the selectivity of aminocarbonylation reactions. This approach enables a dynamic shift between MC and DC of (hetero)aryl halides. The well-defined nature of these active sites provides critical insights into the underlying catalytic mechanisms. Specifically, tuning SBCS enhances iodobenzene activation and stabilizes the adsorption of a second CO molecule, facilitating the DC pathway. The generality and versatility of the catalyst systems are underscored by their ability to synthesize a wide range of aryl amides and ketoamides, including biorelevant pharmaceuticals. This study introduces an underexplored strategy for selectively regulating (carbonylation) reactions, offering avenues for catalyst design and reaction control.

## MATERIALS AND METHODS

### General considerations

All solvents and chemicals, unless otherwise noted, were obtained commercially and were used as received without further purification. All glassware was dried before using. Analytical thin-layer chromatography was performed using precoated Jiangyou silica gel HSGF254 (0.2 ± 0.03 mm). Flash chromatography was performed using silica gel 60, 0.063 to 0.2 mm, 200 to 300 mesh (Jiangyou, Yantai) with the indicated solvent system.

### Characterizations

Gas chromatography–mass spectrometry (GC-MS) analysis was in general recorded on an Agilent 5977A MSD GC-MS. High pressure liquid chromatography (HPLC) analysis was performed on a Waters-Breeze instrument (2487 Dual λ Absorbance Detector and 1525 Binary HPLC Pump) or a SHMADZU system (SHMADZU LC-20AT pump, SHIMADZU LC-20A Absorbance Detector). Chiralpak OD-H, AD-H columns were purchased from Daicel Chemical Industries LTD. The in situ DRIFTS of samples were analyzed by a Bruker VERTEX 70 Fourier transform infrared spectrometer and used for the identification of infrared (IR) absorbance in the mid-IR region (400 to 4000 cm^−1^). It was equipped with liquid nitrogen–cooled Mercury Cadmium Telluride (MCT) detector and low-volume gas cell (8.7 ml) with a 123-mm path length and KBr windows. The sample was pretreated at 200°C for 60 min under Ar (20 ml/min) and cooled to 30°C. After adding iodobenzene, morpholine, and K_2_CO_3_, the system was pressurized with CO to 1 MPa and heated from 30° to 130°C. Raman spectroscopy (Thermo Fisher Scientific, dxr3) was performed by using a 532-nm laser beam. The liquid nuclear magnetic resonance (NMR) spectra were recorded on a Bruker AvanceTM III 400 MHz in deuterated chloroform unless otherwise noted. Data are reported in parts per million as follows: chemical shift, multiplicity (s, singlet; d, doublet; t, triplet; q, quartet; quint, quintet; m, multiplet; dd, doublet of doublet; and br, broad signal), coupling constant in hertz, and integration. XRD measurements were conducted by a STADIP automated transmission diffractometer (STOE) equipped with an incident beam curved germanium monochromator selecting CuKα1 radiation and a 6° position-sensitive detector (step size: 0.014°, step time: 25.05 s). The XRD patterns were scanned in the 2θ range of 10° to 90°. Pd contents were determined by ICP-OES (Agilent, 725-ES). Preparation of test sample: Twenty milligrams of the sample was dissolved with a mixture of concentrated nitric acid and hydrochloric acid, heated until the sample was completely dissolved, and then the clarified transparent solution was quantitatively transferred to a volumetric flask. XPS measurements were carried out by a VG ESCALAB 210 instrument equipped with a dual Mg/Al anode x-Ray source, a hemispherical capacitor analyzer, and a 5-keV Ar^+^ ion gun. All spectra were recorded by using AlKa (1361 eV) radiation. The electron binding energy was referenced to the C1s peak at 284.8 eV. AC-STEM images were captured on the Thermo Fisher Scientific Spectra 300 instrument. The catalyst samples after pretreatment were dispersed in ethanol, and the solution was mixed ultrasonically at room temperature. A part of solution was dropped on the grid for the measurement of AC-STEM images. XANES and EXAFS measurements were conducted at the Pd K edge using beamline BL11B at Shanghai Synchrotron Radiation Facility (SSRF). The samples were uniformly coated onto adhesive tape and analyzed under ambient conditions. The acquired data were processed using Athena and Artemis software for reduction and analysis. EPR spectra were recorded at room temperature on a Bruker cw spectrometer EMX-PLUS (X-band, ν ≈ 9.8 GHz) with a microwave power of 20 mW, a modulation frequency of 100 kHz, and modulation amplitude of up to 1 G; the usage of sample was 10 mg. O_2_ pulse experiments were performed on a chemisorption analyzer equipped with a thermal conductivity detector. The chemisorption analyzer was TP-5080D. A 50-mg sample was initially purged with He at a flow rate of 30 ml/min and a temperature of 200°C for 1 hour to eliminate water and then cooled to room temperature. Subsequently, a continuous pulsing of 5% O_2_/He was introduced to the catalyst surface until saturation was achieved.

### Synthesis of In_2_O_3_ and In_2_O_3_/O_v_

Typically, 1.2 g of urea and 0.44 g of InCl_3_ were dissolved in 60 ml of H_2_O, followed by stirring for 0.5 hours at room temperature. The above mixture was transferred into a 100-ml Teflon-lined stainless steel autoclave and heated to 140°C for 12 hours. After naturally cooling down, the reactant was centrifuged and washed with H_2_O and absolute ethanol for several times. The white powder was then dried at 60°C overnight, which is denoted as In(OH)_3_. Last, In(OH)_3_ was thermal treated in air at 500°C for 1 hour at a ramping rate of 5°C/min; yellow powder was obtained and denoted as In_2_O_3_, while In(OH)_3_ was thermal treated in H_2_ at the same conditions; gray powder was obtained and denoted as In_2_O_3_/O_v_.

### Synthesis of 0.2%Pd_1_-In_2_O_3_ and 0.2%Pd_1_-In_2_O_3_/O_v_

Pd_1_-In_2_O_3_ and Pd_1_-In_2_O_3_/O_v_ were prepared through the impregnation with Na_2_PdCl_4_ as the precursors. Na_2_PdCl_4_ solution (2.0 ml, 0.1 mg/ml) was added to 2.5 ml of acetone and agitated at room temperature until complete dissolution. Then, 100 mg of In_2_O_3_ or In_2_O_3_/O_v_ powder was added, the mixture was stirred at room temperature overnight, and the obtained power was thermal treated in H_2_ at 200°C for 2 hours at a ramping rate of 5°C/min. The catalysts were denoted as 0.2%Pd_1_-In_2_O_3_ and 0.2%Pd_1_-In_2_O_3_/O_v_. All other catalysts such as 0.1%Pd_1_-In_2_O_3_, 0.1%Pd_1_-In_2_O_3_/O_v_, 0.4%Pd_1_-In_2_O_3_, 0.4%Pd_1_-In_2_O_3_/O_v_, 0.2%Pd_1_-In_2_O_3_/O_v_-300, 0.2%Pd_1_-In_2_O_3_/O_v_-350, and 0.2%Pd_1_-In_2_O_3_/O_v_-400 were prepared in the same way.

### Synthesis of gram-scale 0.2%Pd_1_-In_2_O_3_ and gram-scale 0.2%Pd_1_-In_2_O_3_/O_v_

Na_2_PdCl_4_ solution (3.0 ml, 1.0 mg/ml), was added to 5 ml of acetone and agitated at room temperature until complete dissolution. Then, 1500 mg of In_2_O_3_ or In_2_O_3_/O_v_ powder was added, the mixture was stirred at room temperature overnight, and the obtained power was thermal treated in H_2_ at 200°C for 2 hours at a ramping rate of 5°C/min. The catalysts were denoted as gram-scale 0.2%Pd_1_-In_2_O_3_ and gram-scale 0.2%Pd_1_-In_2_O_3_/O_v_.

### General procedure for the aminocarbonylation of aryl halides

Typical procedure for carbonylation of aryl iodides with amines and CO. A mixture of aryl iodides (1.0 mmol), amines (1.5 mmol), catalysts (20 mg), K_2_CO_3_ (2.0 mmol), and dioxane (2 ml) was added into a glass tube, which was placed in an 80-ml autoclave. Then, the autoclave was purged and charged with CO (1.0 MPa). The reaction mixture was stirred at 125°C for 24 hours. After the reaction finished, the autoclave was cooled to room temperature, and the pressure was carefully released. Subsequently, the reaction mixture was diluted with 5 ml of methanol for analysis by GC-MS. The crude reaction mixture was concentrated by rot-vap and purified by column chromatography on a silica gel column to give the desired products.

### Scalability experiments for drug molecules

A mixture of aryl iodides (10.0 mmol), amines (15.0 mmol), 0.2% Pd_1_-In_2_O_3_ (100 mg), K_2_CO_3_ (2.0 mmol), and dioxane (5 ml) were added into a glass tube, which was placed in an 80-ml autoclave. Then, the autoclave was purged and charged with CO (1.0 MPa). The reaction mixture was stirred at 125°C for 48 hours. After the reaction finished, the autoclave was cooled to room temperature, and the pressure was carefully released. Subsequently, the reaction mixture was diluted with 8 ml of methanol for analysis by GC-MS. The crude reaction mixture was concentrated by rot-vap and purified by column chromatography on a silica gel column to give the desired products.

### General procedure for the α-amino acid derivatives (5a)

**Fig. 6.**
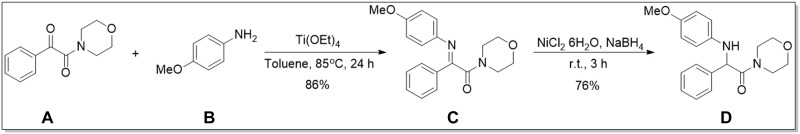
Synthesis of the α-amino acid derivatives (5a). (**A**) α-Ketoamide 4a, 4-(phenylglyoxylyl)morpholine. (**B**) 4-Methoxyaniline. (**C**) α-imino amide intermediate. (**D**) α-amino carbonyl derivative 5a. Et, ethyl; h, hours. Me, methyl; r.t., room temperature.

Compound A (5 mmol, 1.1 g) and compound B (5 mmol, 615 mg) were added into a 100-ml pressure tube, followed by the addition of 5 ml of toluene to dissolve them completely. Subsequently, 1 ml of titanium(IV) ethoxide was added slowly. The reaction system was maintained at 85°C for 24 hours under constant temperature. After the reaction was completed, the reaction was quenched by adding aqueous sodium hydroxide solution, and the resulting mixture was filtered by suction and concentrated by rotary evaporation to remove the solvent. The residue was purified by recrystallization using dichloromethane-petroleum ether (volume ratio 1:10), and, finally, red solid product C was obtained with a mass of 1.39 g and a yield of 86% (Fig. 6).

Compound C (2 mmol, 648 mg) was added into a 50-ml round-bottom flask, and 10 ml of methanol was added to dissolve it. Then, under stirring conditions, sodium borohydride (304 mg, 8 mmol) and nickel(II) chloride hexahydrate (119 mg, 0.5 mmol) were added to the system in batches, and the feeding process lasted for 5 min. After the feeding was completed, the reaction system was stirred at room temperature for 3 hours. After the reaction was finished, the reaction solution was concentrated by rotary evaporation, and the residue was purified by silica gel column chromatography. The eluent was subjected to gradient elution with petroleum ether-ethyl acetate (volume ratios sequentially 5:1 → 3.3:1 → 2:1), and, finally, a yellow oily product was obtained with a mass of 494 mg and a yield of 76%.

### DFT calculations

Spin-polarized DFT methods were performed in this work as imvia the Vienna ab initio simulation package (*43*). The projected augmented wave method was used to describe the interaction of electron and ion (*44*). The electron exchange and correlation energies were calculated within the generalized gradient approximation method using the Perdew-Burke-Ernzerhof functional (*45*, *46*). To make sure that the energy difference is less than 10^−4^ eV and the force per atom is less than 0.03 eV/Å, Gaussian smearing (0.02 eV) was used; Gamma method was adopted for sampling at Brillouin zone; and the k point was set as 2 by 2 by 1. The kinetic energy cutoff was set up to 450 eV, and dispersion correction was considered by using DFT-D3 method with Becke-Jonson damping (*47*). The adsorption energy (*E*_ads_) of adsorbate (X) is obtained from the equation *E*_ads_ = *E*_X/slab_ − *E*_slab_ – *E*_X_, where *E*_X/slab_ is the total energy after adsorption, *E*_slab_ is the total energy of the clean surface, and *E*_X_ is the total energy of the free adsorbate (X) in a 20 by 15 by 15 cubic box. Therefore, the more negative *E*_ads_, the stronger the interactions between the adsorbates and surface, and the opposite number of *E*_ads_ is regarded as the desorption energy *E*_des_. For reactions, the climbing-image nudged elastic band (*48*) method was adopted to search the transition states (TS), and the vibrational frequency analysis was also processed to verify the authentic TS with only one imaginary frequency. The reaction barrier (*E*_a_) is defined as *E*_a_ = *E*_TS_ − *E*_IS_, and the reaction energy (*E*_r_) is defined as *E*_r_ = *E*_FS_ − *E*_IS_, where *E*_IS_, *E*_FS_, and *E*_TS_ are the total energies of the initial, final, and transition states, respectively. Zero point energy correction was included in all energies. To study the aminocarbonylation reaction on the surface of Pd_1_-In_2_O_3_ and Pd_1_-In_2_O_3_/O_v_, we chose the (111) surface of In_2_O_3_ based on the XRD pattern and AC-HAADF-STEM images, in which one O atoms on the surface were removed to form oxygen O_v_. For Pd-doped In_2_O_3_ or In_2_O_3_/O_v_, one In atom was replaced by one Pd atom on the surface, and then we optimized the surface structure with the bottom two layers of atoms fixed, and the thickness of the vacuum layer was 20 Å.

## Supplementary Materials

### This PDF file includes:

Supplementary Text Figs. S1 to S8 Tables S1 to S9 Characterization data for products HPLC Spectra of Chiral compounds NMR spectra of products References

## References

[1] S. Agudo-Álvarez, S. S. Díaz-Mínguez, R. Benito-Arenas, The amide group and its preparation methods by acid-amine coupling reactions: An overview. Pure Appl. Chem. 96, 691–707 (2024).

[2] V. R. Pattabiraman, J. W. Bode, Rethinking amide bond synthesis. Nature 480, 471–479 (2011). 22193101 10.1038/nature10702

[3] B. C. Haas, A. E. Goetz, A. Bahamonde, J. C. McWilliams, M. S. Sigman, Predicting relative efficiency of amide bond formation using multivariate linear regression. Proc. Natl. Acad. Sci. U.S.A. 119, e2118451119 (2022). 35412905 10.1073/pnas.2118451119 PMC9169781

[4] H. Liu, G. Laurenczy, N. Yan, P. J. Dyson, Amide bond formation via C(sp^3^)-H bond functionalization and CO insertion. Chem. Commun. 50, 341–343 (2014). 10.1039/c3cc47015f 24244940

[5] N. E. Leadbeater, M. Marco, Preparation of polymer-supported ligands and metal complexes for use in catalysis. Chem. Rev. 102, 3217–3274 (2002). 12371884 10.1021/cr010361c

[6] L. Zeng, H. Li, J. Hu, D. Zhang, J. Hu, P. Peng, S. Wang, R. Shi, J. Peng, C.-W. Pao, J.-L. Chen, J.-F. Lee, H. Zhang, Y.-H. Chen, A. Lei, Electrochemical oxidative aminocarbonylation of terminal alkynes. Nat. Catal. 3, 438–445 (2020).

[7] L. J. Lu, X. L. Pei, Y. Mei, Y. Deng, H. Zhang, L. N. Zhang, A. W. Lei, Carbon nanofibrous microspheres promote the oxidative double carbonylation of alkanes with CO. Chem 4, 2861–2871 (2018).

[8] Y. W. Cao, Y. Peng, D. Y. Cheng, L. Chen, M. L. Wang, C. Shang, L. R. Zheng, D. Ma, Z. P. Liu, L. He, Room-temperature co oxidative coupling for oxamide production over interfacial Au/ZnO catalysts. ACS Catal. 13, 735–743 (2023).

[9] Y. Zhang, H. Q. Geng, X. F. Wu, Palladium-catalyzed perfluoroalkylative carbonylation of unactivated alkenes: Access to beta-perfluoroalkyl esters. Angew. Chem. Int. Ed. Engl. 60, 24292–24298 (2021). 34506080 10.1002/anie.202111206

[10] C. De Risi, G. P. Pollini, V. Zanirato, Recent developments in general methodologies for the synthesis of α-ketoamides. Chem. Rev. 116, 3241–3305 (2016). 26881454 10.1021/acs.chemrev.5b00443

[11] F. Zhao, H.-J. Ai, X.-F. Wu, Copper-catalyzed substrate-controlled carbonylative synthesis of α-Keto amides and amides from alkyl halides. Angew. Chem. Int. Ed. Engl. 61, e202200062 (2022). 35175679 10.1002/anie.202200062

[12] S.-Q. Yang, Y.-Q. Yao, X.-C. Chen, Y. Lu, X.-L. Zhao, Y. Liu, Pd-catalyst containing a hemilabile P,C-hybrid ligand in amino dicarbonylation of aryl halides for synthesis of α-ketoamides. Organometallics 40, 1032–1041 (2021).

[13] N. Uzunlu, P. Pongracz, L. Kollar, A. Takacs, Alkyl levulinates and 2-methyltetrahydrofuran: Possible biomass-based solvents in palladium-catalyzed aminocarbonylation. Molecules 28, 1–15 (2023). 10.3390/molecules28010442 PMC9823927 36615634

[14] B. Aranda, S. A. Moya, A. Vega, G. Valdebenito, S. Ramirez-Lopez, P. Aguirre, New palladium (II) complexes containing phosphine-nitrogen ligands and their use as catalysts in aminocarbonylation reaction. Appl. Organomet. Chem. 33, e4709 (2019).

[15] M. Genelot, N. Villandier, A. Bendjeriou, P. Jaithong, L. Djakovitch, V. Dufaud, Palladium complexes grafted onto mesoporous silica catalysed the double carbonylation of aryl iodides with amines to give α-ketoamides. Cat. Sci. Technol. 2, 1886 (2012).

[16] M. Papp, R. Skoda-Földes, Phosphine-free double carbonylation of iodobenzene in the presence of reusable supported palladium catalysts. J. Mol. Catal. A Chem. 378, 193–199 (2013).

[17] B. Urbán, E. Nagy, P. Nagy, M. Papp, R. Skoda-Földes, Double carbonylation of iodoarenes in the presence of a pyridinium SILP-Pd catalyst. J. Organomet. Chem. 918, 121287 (2020).

[18] M. Papp, P. Szabó, D. Srankó, G. Sáfrán, L. Kollár, R. Skoda-Földes, Mono- and double carbonylation of aryl iodides with amine nucleophiles in the presence of recyclable palladium catalysts immobilised on a supported dicationic ionic liquid phase. RSC Adv. 7, 44587–44597 (2017).

[19] A. W. Heard, J. M. Suárez, S. M. Goldup, Controlling catalyst activity, chemoselectivity and stereoselectivity with the mechanical bond. Nat. Rev. Chem. 6, 182–196 (2022). 37117433 10.1038/s41570-021-00348-4

[20] A. Milo, E. N. Bess, M. S. Sigman, Interrogating selectivity in catalysis using molecular vibrations. Nature 507, 210–214 (2014). 24622199 10.1038/nature13019

[21] B. Zhang, J. Wang, G. Liu, C. M. Weiss, D. Liu, Y. Chen, L. Xia, P. Zhou, M. Gao, Y. Liu, J. Chen, Y. Yan, M. Shao, H. Pan, W. Sun, A strongly coupled Ru-CrO_x_ cluster-cluster heterostructure for efficient alkaline hydrogen electrocatalysis. Nat. Catal. 7, 441–451 (2024).

[22] A. Parastaev, V. Muravev, E. H. Osta, T. F. Kimpel, J. F. M. Simons, A. J. F. van Hoof, E. Uslamin, L. Zhang, J. J. C. Struijs, D. B. Burueva, E. V. Pokochueva, K. V. Kovtunov, I. V. Koptyug, I. J. Villar-Garcia, C. Escudero, T. Altantzis, P. Liu, A. Béché, S. Bals, N. Kosinov, E. J. M. Hensen, Breaking structure sensitivity in CO_2_ hydrogenation by tuning metal-oxide interfaces in supported cobalt nanoparticles. Nat. Catal. 5, 1051–1060 (2022).

[23] S. Li, Y. Xu, Y. Chen, W. Li, L. Lin, M. Li, Y. Deng, X. Wang, B. Ge, C. Yang, S. Yao, J. Xie, Y. Li, X. Liu, D. Ma, Tuning the selectivity of catalytic carbon dioxide hydrogenation over iridium/cerium oxide catalysts with a strong metal-support interaction. Angew. Chem. Int. Ed. Engl. 56, 10761–10765 (2017). 28691396 10.1002/anie.201705002

[24] R. Gao, J. Xu, J. Wang, J. Lim, C. Peng, L. Pan, X. Zhang, H. Yang, J. J. Zou, Pd/Fe_2_O_3_ with electronic coupling single-site Pd-Fe pair sites for low-temperature semihydrogenation of alkynes. J. Am. Chem. Soc. 144, 573–581 (2022). 34955021 10.1021/jacs.1c11740

[25] Z. S. Zhu, S. Zhong, C. Cheng, H. Zhou, H. Sun, X. Duan, S. Wang, Microenvironment engineering of heterogeneous catalysts for liquid-phase environmental catalysis. Chem. Rev. 124, 11348–11434 (2024). 39383063 10.1021/acs.chemrev.4c00276

[26] X. Dai, S. Adomeit, J. Rabeah, C. Kreyenschulte, A. Bruckner, H. Wang, F. Shi, Sustainable co-synthesis of glycolic acid, formamides and formates from 1,3-dihydroxyacetone by a Cu/Al_2_O_3_ catalyst with a single active sites. Angew. Chem. Int. Ed. Engl. 58, 5251–5255 (2019). 30715789 10.1002/anie.201814050

[27] Z. Zhao, P. Wang, C. Song, T. Zhang, S. Zhan, Y. Li, Enhanced interfacial electron transfer by asymmetric Cu-O_v_-In sites on In_2_O_3_ for efficient peroxymonosulfate activation. Angew. Chem. Int. Ed. Engl. 62, e202216403 (2023). 36646650 10.1002/anie.202216403

[28] X. Zhang, F. Kraushofer, Q. Yuan, Y.-X. Wang, M. Krinninger, Z. Su, H. Gai, K. Goodman, X. Zhang, Y. Wang, X. Tong, T. Cheng, J.-F. Wu, B. A. J. Lechner, M. Blum, Pd-promoted reduction and restructuring of an In2O3-based catalyst for CO2 hydrogenation at room temperature. J. Catal. 454, 116618 (2026).

[29] Q. Jiang, X. Li, Y. Hao, J. Zuo, R. Duan, J. Li, G. Cao, J. Wang, J. Wang, M. Li, X. Yang, M. Li, W. Li, Y. Xi, J. Zhang, W. Xiao, Oxygen-vacancy-assisted dual functional surface coatings suppressing irreversible phase transition of Li-rich layered oxide cathodes. Adv. Funct. Mater. 35, 2400670 (2024).

[30] C. Huang, Z. Jiang, F. Liu, W. Li, Q. Liang, Z. Zhao, X. Ge, K. Song, L. Zheng, X. Zhou, S. Qiao, W. Zhang, W. Zheng, Oxygen vacancies boosted hydronium intercalation: A paradigm shift in aluminum-based batteries. Angew. Chem. Int. Ed. Engl. 63, e202405592 (2024). 38647330 10.1002/anie.202405592

[31] L. Luo, L. Fu, H. Liu, Y. Xu, J. Xing, C.-R. Chang, D.-Y. Yang, J. Tang, Synergy of Pd atoms and oxygen vacancies on In_2_O_3_ for methane conversion under visible light. Nat. Commun. 13, 2930 (2022). 35614052 10.1038/s41467-022-30434-0 PMC9132922

[32] S. Chen, H. Wang, Z. Kang, S. Jin, X. Zhang, X. Zheng, Z. Qi, J. Zhu, B. Pan, Y. Xie, Oxygen vacancy associated single-electron transfer for photofixation of CO_2_ to long-chain chemicals. Nat. Commun. 10, 788 (2019). 30770824 10.1038/s41467-019-08697-x PMC6377667

[33] X. Zhu, Q. Guo, Y. Sun, S. Chen, J.-Q. Wang, M. Wu, W. Fu, Y. Tang, X. Duan, D. Chen, Y. Wan, Optimising surface d charge of AuPd nanoalloy catalysts for enhanced catalytic activity. Nat. Commun. 10, 1428 (2019). 30926804 10.1038/s41467-019-09421-5 PMC6441046

[34] J. Chen, D. Zhang, B. Liu, K. Zheng, Y. Li, Y. Xu, Z. Li, X. Liu, Photoinduced precise synthesis of diatomic Ir_1_Pd_1_-In_2_O_3_ for CO_2_ hydrogenation to methanol via angstrom-scale-distance dependent synergistic catalysis. Angew. Chem. Int. Ed. Engl. 63, e202401168 (2024). 38336924 10.1002/anie.202401168

[35] L. Chen, X. Guan, X. Wu, H. Asakura, D. G. Hopkinson, C. Allen, J. Callison, P. J. Dyson, F. R. Wang, Thermally stable high-loading single Cu sites on ZSM-5 for selective catalytic oxidation of NH_3_. Proc. Natl. Acad. Sci. U.S.A. 121, e2404830121 (2024). 39042689 10.1073/pnas.2404830121 PMC11295017

[36] A. P. Kourounakis, D. Xanthopoulos, A. Tzara, Morpholine as a privileged structure: A review on the medicinal chemistry and pharmacological activity of morpholine containing bioactive molecules. Med. Res. Rev. 40, 709–752 (2020). 31512284 10.1002/med.21634

[37] H. Iino, J.-i. Hanna, Liquid crystalline organic semiconductors for organic transistor applications. Polym. J. 49, 23–30 (2017).

[38] S. Liu, T. Li, F. Shi, H. Ma, B. Wang, X. Dai, X. Cui, Constructing multiple active sites in iron oxide catalysts for improving carbonylation reactions. Nat. Commun. 14, 4973 (2023). 37591841 10.1038/s41467-023-40640-z PMC10435489

[39] A. G. Sergeev, A. Spannenberg, M. Beller, Palladium-catalyzed formylation of aryl bromides: Elucidation of the catalytic cycle of an industrially applied coupling reaction. J. Am. Chem. Soc. 130, 15549–15563 (2008). 18956867 10.1021/ja804997z

[40] J. Gu, F. Zhao, K. N. Houk, Q. Lu, F. Liu, Computational determination of the mechanism of the Pd-catalyzed formation of isatoic anhydrides from o-haloanilines, CO, and CO_2_. Dalton Trans. 50, 14453–14461 (2021). 34571528 10.1039/d1dt02551a

[41] A. Mukherjee, S. Mahato, D. S. Kopchuk, S. Santra, G. V. Zyryanov, A. Majee, O. N. Chupakhin, Synthesis of α-amino carbonyl compounds: A brief review. Russ. Chem. Rev. 92, RCR5046 (2023).

[42] Y. Zhang, J. Vanderghinste, J. Wang, S. Das, Challenges and recent advancements in the synthesis of α,α-disubstituted α-amino acids. Nat. Commun. 15, 1474 (2024). 38368416 10.1038/s41467-024-45790-2 PMC10874380

[43] G. Kresse, J. Furthmuller, Efficiency of ab-initio total energy calculations for metals and semiconductors using a plane-wave basis set. Comp. Mater. Sci. 6, 15–50 (1996). 10.1103/physrevb.54.11169 9984901

[44] P. E. Blöchl, Projector augmented-wave method. Phys. Rev. B 50, 17953–17979 (1994). 10.1103/physrevb.50.17953 9976227

[45] J. P. Perdew, K. Burke, M. Ernzerhof, Generalized gradient approximation made simple. Phys. Rev. Lett. 77, 3865–3868 (1996). 10062328 10.1103/PhysRevLett.77.3865

[46] J. P. Perdew, K. Burke, M. Ernzerhof, Generalized gradient approximation made simple. Phys. Rev. Lett. 78, 1396–1396 (1997). 10.1103/PhysRevLett.77.3865 10062328

[47] T. Risthaus, S. Grimme, Benchmarking of london dispersion-accounting density functional theory methods on very large molecular complexes. J. Chem. Theory. Comput. 9, 1580–1591 (2013). 26587619 10.1021/ct301081n

[48] H. Jónsson, G. Mills, K. W. Jacobsen, in *Classical and Quantum Dynamics in Condensed Phase Simulations*, B. J. Berne, G. Ciccotti, D. F. Coker, Eds. (World Scientific, 1998), pp. 385-404.

[49] S. Zheng, Y. Wang, C. Zhang, J. Liu, C. Xia, NHC-Pd complex-catalyzed double carbonylation of aryl iodides with secondary amines to α-keto amides. Appl. Organomet. Chem. 28, 48–53 (2013).

[50] M. Vico Solano, G. González Miera, V. Pascanu, A. K. Inge, B. Martín-Matute, Versatile heterogeneous palladium catalysts for diverse carbonylation reactions under atmospheric carbon monoxide pressure. ChemCatChem 10, 1089–1095 (2018).

[51] H. Du, Q. Ruan, M. Qi, W. Han, Ligand-free Pd-catalyzed double carbonylation of aryl iodides with amines to α-Ketoamides under atmospheric pressure of carbon monoxide and at room temperature. J. Org. Chem. 80, 7816–7823 (2015). 26140509 10.1021/acs.joc.5b01249

[52] B. Chen, F. Li, Z. Huang, T. Lu, G. Yuan, Stability or flexibility: Metal nanoparticles supported over cross-linked functional polymers as catalytic active sites for hydrogenation and carbonylation. Appl. Catal. A Gen. 481, 54–63 (2014).

[53] S. Maji, M. Roy, K. Shaikh, D. Adhikari, Organophotocatalytic dehydrogenative preparation of amides directly from alcohols. Green Chem. 25, 8019–8025 (2023).

[54] Y. Zheng, Y. Zhao, S. Tao, X. Li, X. Cheng, G. Jiang, X. Wan, Green esterification of carboxylic acids promoted by tert-butyl nitrite. Eur. J. Org. Chem. 2021, 2713–2718 (2021).

[55] J. Su, J.-N. Mo, X. Chen, A. Umanzor, Z. Zhang, K. N. Houk, J. Zhao, Generation of oxyphosphonium ions by photoredox/cobaloxime catalysis for scalable amide and peptide synthesis in batch and continuous-flow. Angew. Chem. Int. Ed. Engl. 61, e202112668 (2022). 34783121 10.1002/anie.202112668

[56] D. I. Tzaras, M. Gorai, T. Jacquemin, T. Arndt, B. M. Zimmermann, M. Breugst, J. F. Teichert, Site-selective copper(I)-catalyzed hydrogenation of amides. J. Am. Chem. Soc. 147, 1867–1874 (2025). 39752259 10.1021/jacs.4c14174 PMC11744755

[57] K. P. Patel, E. M. Gayakwad, G. S. Shankarling, Graphene oxide: A convenient metal-free carbocatalyst for facilitating amidation of esters with amines. New J. Chem. 44, 2661–2668 (2020).

[58] A. Sarswat, R. Kumar, L. Kumar, N. Lal, S. Sharma, Y. S. Prabhakar, S. K. Pandey, J. Lal, V. Verma, A. Jain, J. P. Maikhuri, D. Dalela, Kirti, G. Gupta, V. L. Sharma, Arylpiperazines for management of benign prostatic hyperplasia: Design, synthesis, quantitative structure-activity relationships, and pharmacokinetic studies. J. Med. Chem. 54, 302–311 (2011). 21128595 10.1021/jm101163m

[59] V. Vinayagam, S. K. Sadhukhan, D. V. Botla, R. R. Chittem, S. R. Kasu, T. V. Hajay Kumar, Mild method for deprotection of the *N*-benzyloxycarbonyl (N-Cbz) group by the combination of AlCl_3_ and HFIP. J. Org. Chem. 89, 5665–5674 (2024). 38574289 10.1021/acs.joc.4c00177

[60] X. Chen, T. Chen, Q. Li, Y. Zhou, L. B. Han, S. F. Yin, Copper-catalyzed aerobic oxidative inert C-C and C-N bond cleavage: A new strategy for the synthesis of tertiary amides. Chemistry 20, 12234–12238 (2014). 25099559 10.1002/chem.201403144

[61] S. Ghinato, D. Territo, A. Maranzana, V. Capriati, M. Blangetti, C. Prandi, A fast and general route to ketones from amides and organolithium compounds under aerobic conditions: Synthetic and mechanistic aspects. Chemistry 27, 2868–2874 (2021). 33150980 10.1002/chem.202004840

[62] J. Zhao, J. Shi, Y. Li, Benzyne-mediated esterification reaction. Org. Lett. 23, 7274–7278 (2021). 34498880 10.1021/acs.orglett.1c02702

[63] D. Xu, L. Shi, D. Ge, X. Cao, H. Gu, Platinum nanowires catalyzed direct amidation with aldehydes and amines. Sci. China Chem. 59, 478–481 (2016).

[64] A. R. Bayguzina, A. R. Lutfullina, R. I. Khusnutdinov, Synthesis of N-(adamantan-1-yl)carbamides by ritter reaction from adamantan-1-ol and nitriles in the presence of Cu-catalysts. Russ. J. Org. Chem. 54, 1127–1133 (2018).

[65] G. W. Wang, N. G. McCreanor, M. H. Shaw, W. G. Whittingham, J. F. Bower, New initiation modes for directed carbonylative C-C bond activation: Rhodium-catalyzed (3 + 1 + 2) cycloadditions of aminomethylcyclopropanes. J. Am. Chem. Soc. 138, 13501–13504 (2016). 27709913 10.1021/jacs.6b08608 PMC5073370

[66] N. Wang, X. Zou, J. Ma, F. Li, The direct synthesis of N-alkylated amides via a tandem hydration/N-alkylation reaction from nitriles, aldoximes and alcohols. Chem. Commun. 50, 8303–8305 (2014). 10.1039/c4cc02742f 24935726

[67] K. S. Sharma, N. Thadem, G. Pandey, Visible-light-induced secondary benzylic C(sp^3^)-H functionalization for nucleophilic substitution: An intermolecular C-X (C-N, C-C, and C-Br) bond forming reaction. J. Org. Chem. 90, 3384–3390 (2025). 39999344 10.1021/acs.joc.4c03052

[68] M. Ayub Ali, S. M. A. Hakim Siddiki, K. Kon, K.-i. Shimizu, Fe^3+^-exchanged clay catalyzed transamidation of amides with amines under solvent-free condition. Tetrahedron Lett. 55, 1316–1319 (2014).

[69] Y. He, C. Du, J. Han, J. Han, C. Zhu, J. Xie, Manganese-catalyzed anti-markovnikov hydroarylation of enamides: Modular synthesis of arylethylamines. Chin. J. Chem. 40, 1546–1552 (2022).

[70] Q. Li, D. D. Ma, J. Zhao, W. Wei, S. G. Han, X. T. Wu, R. Zou, Q. Xu, Q. L. Zhu, Modular synchronous synthesis of amides and α-Ketoamides realized by matching electrolysis-paired tandems. Angew. Chem. Int. Ed. Engl. 64, e202503440 (2025). 40519173 10.1002/anie.202503440

[71] Y. Chen, S. Gu, Y. Zhao, Y. You, F. Ma, F. Zhu, S. Zhu, L.-G. Xie, Degradation of sulfur hexafluoride and its application in the synthesis of esters and amides. Green Chem. 27, 2921–2930 (2025).

[72] C. Y. Huang, A. G. Doyle, Electron-deficient olefin ligands enable generation of quaternary carbons by Ni-catalyzed cross-coupling. J. Am. Chem. Soc. 137, 5638–5641 (2015). 25879801 10.1021/jacs.5b02503

[73] K. Zhu, M. P. Shaver, S. P. Thomas, Chemoselective nitro reduction and hydroamination using a single iron catalyst. Chem. Sci. 7, 3031–3035 (2016). 29997793 10.1039/c5sc04471e PMC6005157

[74] T. Truong, G. H. Dang, N. V. Tran, N. T. Truong, D. T. Le, N. T. S. Phan, Oxidative cross-dehydrogenative coupling of amines and α-carbonyl aldehydes over heterogeneous Cu-MOF-74 catalyst: A ligand- and base-free approach. J. Mol. Catal. A Chem. 409, 110–116 (2015).

[75] W. Wei, Y. Shao, H. Hu, F. Zhang, C. Zhang, Y. Xu, X. Wan, Coupling of methyl ketones and primary or secondary amines leading to α-ketoamides. J. Org. Chem. 77, 7157–7165 (2012). 22873786 10.1021/jo301117b

[76] J. Y. Chen, H. Y. Wu, Q. W. Gui, X. R. Han, Y. Wu, K. Du, Z. Cao, Y. W. Lin, W. M. He, Electrochemical synthesis of α-Ketoamides under catalyst-, oxidant-, and electrolyte-free conditions. Org. Lett. 22, 2206–2209 (2020). 32124611 10.1021/acs.orglett.0c00387

[77] X. Wang, Y. Li, N. Zhao, H. Liu, Y. Zhou, Copper-catalyzed synthesis of α-keto amides from sulfoxonium ylides. J. Org. Chem. 88, 8268–8278 (2023). 37222407 10.1021/acs.joc.3c00281

[78] F. Heaney, J. Fenlon, P. McArdleb, D. Cunninghamb, α-Keto amides as precursors to heterocycles-generation and cycloaddition reactions of piperazin-5-one nitrones. Org. Biomol. Chem. 1, 1122–1132 (2003). 12926386 10.1039/b210943n

[79] S. Singh, S. Popuri, Q. M. Junaid, S. Sabiah, J. Kandasamy, Diversification of α-ketoamides via transamidation reactions with alkyl and benzyl amines at room temperature. Org. Biomol. Chem. 19, 7134–7140 (2021). 34355726 10.1039/d1ob01021b

[80] S. Das, S. Mondal, S. P. Midya, S. Mondal, E. Ghosh, P. Ghosh, Base-promoted tandem pathway for keto-amides: Visible light-mediated room-temperature amidation using molecular oxygen as an oxidant. J. Org. Chem. 88, 14847–14859 (2023). 37867455 10.1021/acs.joc.3c00686

[81] Z. Li, Y. Zhang, K. Li, Z. Zhou, Z. Zha, Z. Wang, Selective electrochemical oxidation of aromatic hydrocarbons and preparation of mono/multi-carbonyl compounds. Sci. China Chem. 64, 2134–2141 (2021).

[82] Q. W. Tan, P. Chovatia, M. C. Willis, Copper-catalysed synthesis of alkylidene 2-pyrrolinone derivatives from the combination of α-keto amides and alkynes. Org. Biomol. Chem. 16, 7797–7800 (2018). 30327813 10.1039/c8ob02205d

[83] J. Ma, X. Cui, J. Xu, Y. Tan, Y. Wang, X. Wang, Y. Li, One-pot synthesis of α-ketoamides from α-keto acids and amines using ynamides as coupling reagents. J. Org. Chem. 87, 3661–3667 (2022). 35029390 10.1021/acs.joc.1c02453

[84] D. Chen, C. Cheng, S. Zeng, Y. Luo, J. Zhang, W. Deng, Z. Zeng, R. Wang, J. Xiang, Cu(OAc)_2_ and acids promoted the oxidative cleavage of α-aminocarbonyl compounds with amines: Efficient and selective synthesis of 2-t-amino-2-imino-carbonyl and 2-amino-2-oxocarbonyl. Tetrahedron Lett. 61, 151913 (2020).

[85] T. S. Brunner, P. W. Roesky, Enantiopure amidinate complexes of lutetium. J. Organomet. Chem. 849-850, 150–156 (2017).

[86] J. Iley, R. Tolando, L. Constantino, Chemical and microsomal oxidation of tertiary amides: Regio- and stereoselective aspects. J. Chem. Soc. Perkin Trans. *2*, 1299–1305 (2001).

[87] J. Li, B. Cheng, X. Shu, Z. Xu, C. Li, H. Huo, Enantioselective alkylation of α-amino C(sp^3^)−H bonds via photoredox and nickel catalysis. Nat. Catal. 7, 889–899 (2024).

[88] A. Welker, C. Kersten, C. Muller, R. Madhugiri, C. Zimmer, P. Muller, R. Zimmermann, S. Hammerschmidt, H. Maus, J. Ziebuhr, C. Sotriffer, T. Schirmeister, Structure-activity relationships of benzamides and isoindolines designed as SARS-CoV protease inhibitors effective against SARS-CoV-2. ChemMedChem 16, 340–354 (2021). 32930481 10.1002/cmdc.202000548 PMC7894572

[89] F. Jin, G. Xu, F. Li, W. Cao, B. Liu, Y. Xu, Y. Chu, W. Song, P. Peng, K. Feng, Copper-mediated oxidative coupling of difluoromethyl bromides/chlorides with primary amines: Direct synthesis of α-ketoamides. J. Org. Chem. 90, 11811–11817 (2025). 40801255 10.1021/acs.joc.5c00958

[90] A. Ali, J. Wang, R. S. Nathans, H. Cao, N. Sharova, M. Stevenson, T. M. Rana, Synthesis and structure-activity relationship studies of HIV-1 virion infectivity factor (Vif) inhibitors that block viral replication. ChemMedChem 7, 1217–1229 (2012). 22555953 10.1002/cmdc.201200079 PMC3517065

[91] R. J. Huang, T. G. Ong, R. J. Chein, Total synthesis of cassane-type diterpenoid pikrosalvin. J. Chin. Chem. Soc. 70, 2127–2135 (2023).

[92] S. Y. Wang, X. Liu, L. W. Meng, M. M. Li, Y. R. Li, G. X. Yu, J. Song, H. Y. Zhang, P. Chen, S. Y. Zhang, T. Hu, Discovery of indoline derivatives as anticancer agents via inhibition of tubulin polymerization. Bioorg. Med. Chem. Lett. 43, 128095 (2021). 33965530 10.1016/j.bmcl.2021.128095

[93] S. Kar, Y. Xie, Q. Q. Zhou, Y. Diskin-Posner, Y. Ben-David, D. Milstein, Near-ambient-temperature dehydrogenative synthesis of the amide bond: Mechanistic insight and applications. ACS Catal. 11, 7383–7393 (2021). 34168903 10.1021/acscatal.1c00728 PMC8218306

