# Supplementary material for: Chemodivergent aminocarbonylation enabled by oxygen vacancy–engineered Pd-doped In2O3 nanocatalysts
Source: Sci Adv. 2026 Jun 26;12(26):eaef9067. doi: 10.1126/sciadv.aef9067 (PMC13308618; doi:10.1126/sciadv.aef9067)
Supplement: Supplementary file 1 — Supplementary Text Figs. S1 to S8 Tables S1 to S9 Characterization data for products HPLC Spectra of Chiral compounds NMR spectra of products References [file sciadv.aef9067_sm.pdf]

Science Advances
AAAS

Supplementary Materials for

Chemodivergent aminocarbonylation enabled by oxygen vacancy–engineered Pd-doped In_2O_3 nanocatalysts

Shujuan Liu *et al.*

Corresponding author: Matthias Beller, Matthias.Beller@catalysis.de; Xinjiang Cui, xinjiangcui@licp.cas.cn

Sci. Adv. **12**, eaef9067 (2026)
DOI: 10.1126/sciadv.aef9067

This PDF file includes:

Supplementary Text
Figs. S1 to S8
Tables S1 to S9
Characterization data for products
HPLC Spectra of Chiral compounds
NMR spectra of products
References

Supplementary Text

The XRD revealed that the crystallization and composition of Pd_1-In_2O_3 and Pd_1-In_2O_3/O_v were well preserved (**Fig. S3a**). The O_v concentration in the spent Pd_1-In_2O_3 and Pd_1-In_2O_3/O_v by EPR also showed no difference as compared with that of the fresh sample (**Fig. S3b**). The XANES spectra and EXAFS oscillation of the spent Pd_1-In_2O_3 and Pd_1-In_2O_3/O_v remained unchanged (**Fig. S3c-3f**). No Pd-Pd bond can be detected in the spent Pd_1-In_2O_3 and Pd_1-In_2O_3/O_v, demonstrating that the atomically dispersed Pd species were reserved. In addition, the FT-EXAFS fitting analysis further confirmed that the coordination motifs remained after spent catalysts (**Table S1**). Besides, there were no clusters or particles caused by Pd aggregation on the surface of the samples after multiple uses based on the AC-HAADF-STEM measurements (**Fig. S3j-3l**). The recycling experiments and the characterizations of the spent Pd_1-In_2O_3 and Pd_1-In_2O_3/O_v catalysts confirmed that the catalysts were highly stable without the change of catalyst structure.

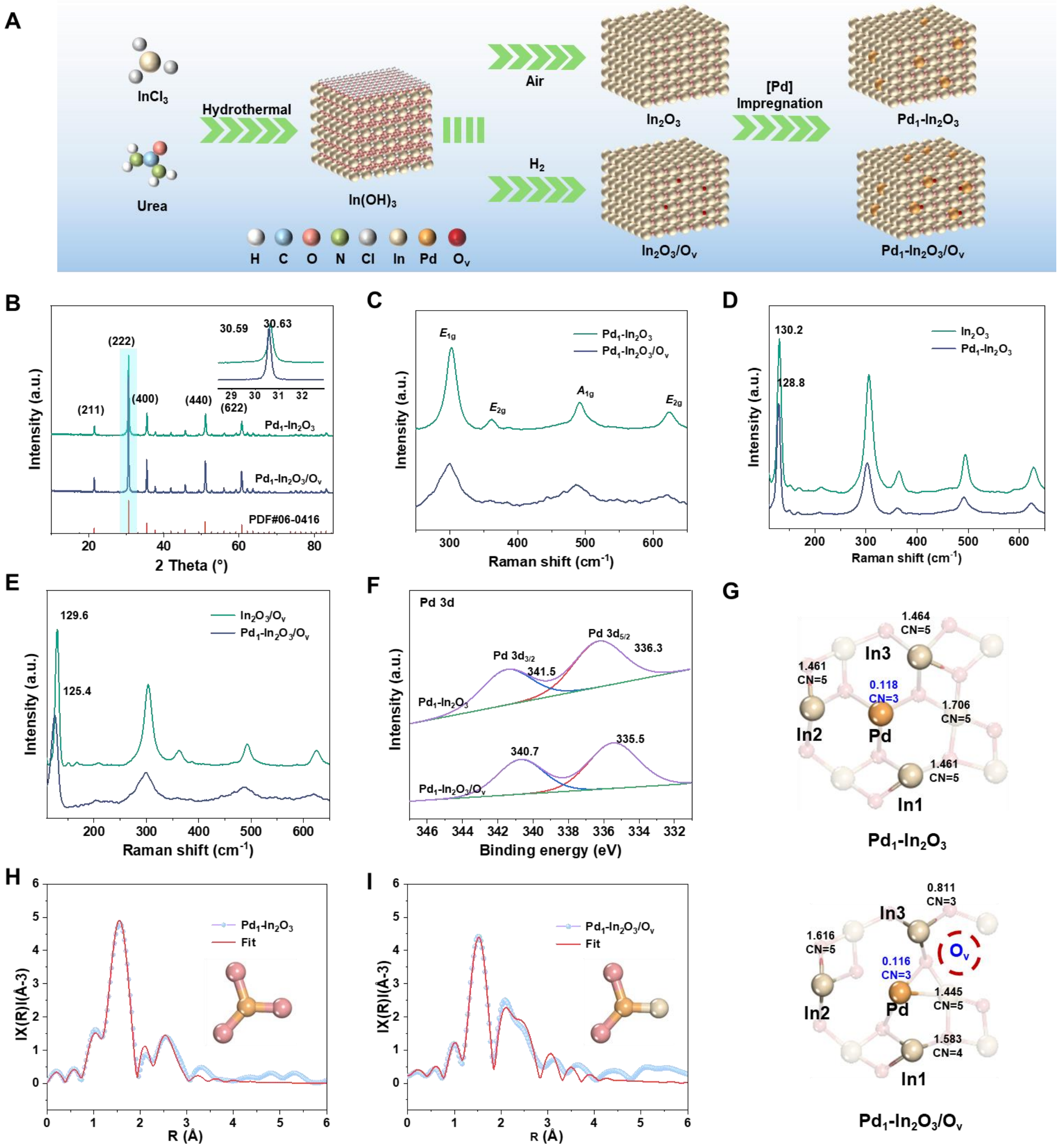

Fig. S1.

Catalysts synthesis and characterization. A, Strategy for the preparation of Pd_1-In_2O_3 and Pd_1-In_2O_3/O_v. **B**, XRD patterns. **C**, **D**, **E**, Raman spectra. **F**, The Pd 3d regions. **G**, Bader charge and coordination number (CN) of Pd and In atoms in Pd_1-In_2O_3 **(up)** and Pd_1-In_2O_3/O_v **(down)** surfaces. **H** and **I**, Experimental and best-fitted EXAFS spectra in R space for Pd_1-In_2O_3 and Pd_1-In_2O_3/O_v.

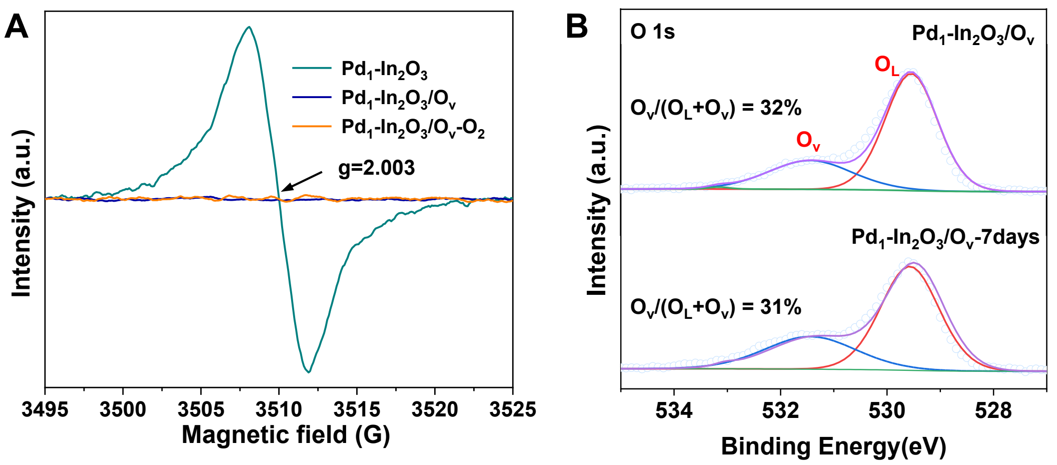

Fig. S2.

ERP and O 1s XPS spectra. A, The surface O_v of Pd_1-In_2O_3/O_v was annihilated by O_2 (donated as Pd_1-In_2O_3/O_v-O_2). **B**, The Ov concentration of Pd_1-In_2O_3/O_v and Pd_1-In_2O_3/O_v-7days.

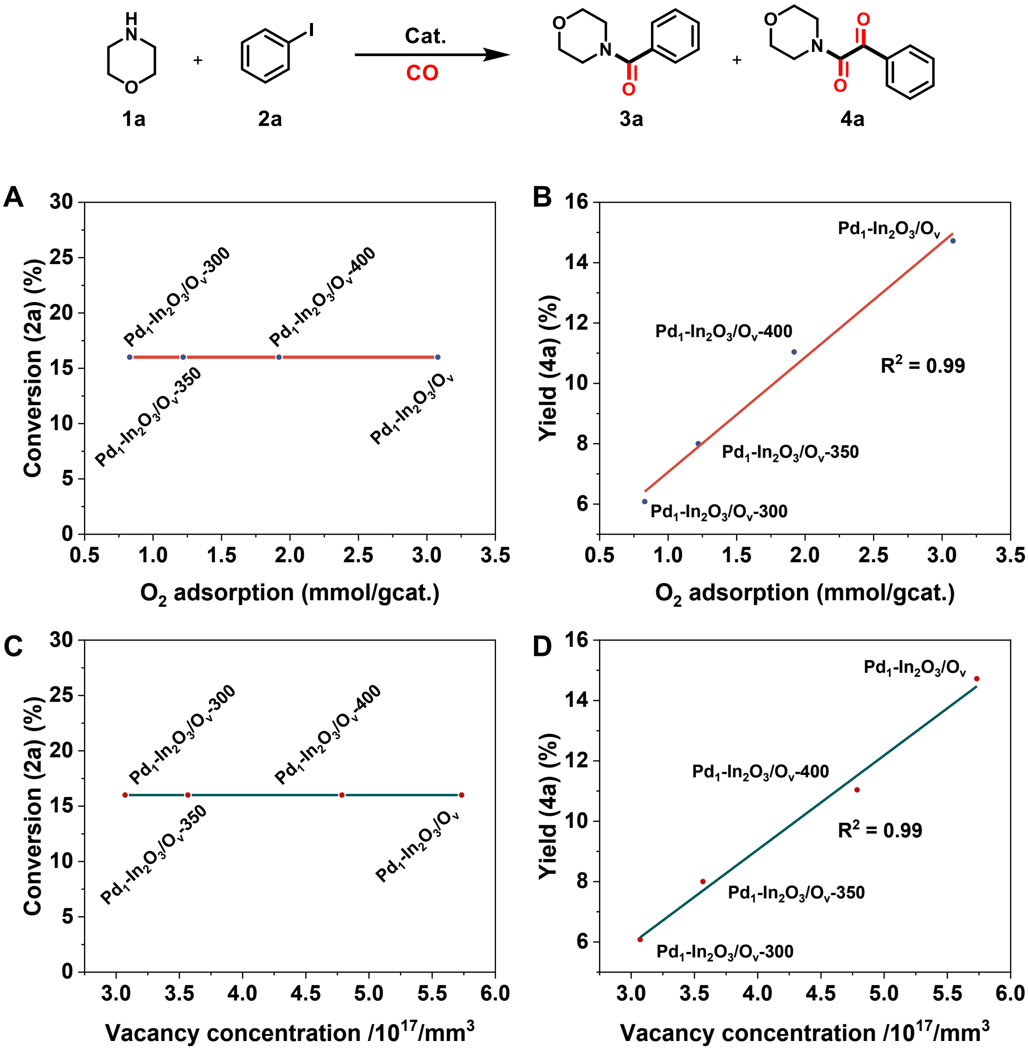

Fig. S3.

The quantitative correlation between O_v concentration and intrinsic catalytic activity. A, The relationship between O_2 pulses and intrinsic catalytic activity (Conversion of **2a**). **B**, The relationship between the concentration of oxygen vacancies determined by EPR and intrinsic catalytic activity (Conversion of **2a**). (**C**), The relationship between O_2 pulses and intrinsic yield of **4a**. **D**, The linear relationship between the concentration of oxygen vacancies determined by EPR and intrinsic yield of **4a**.

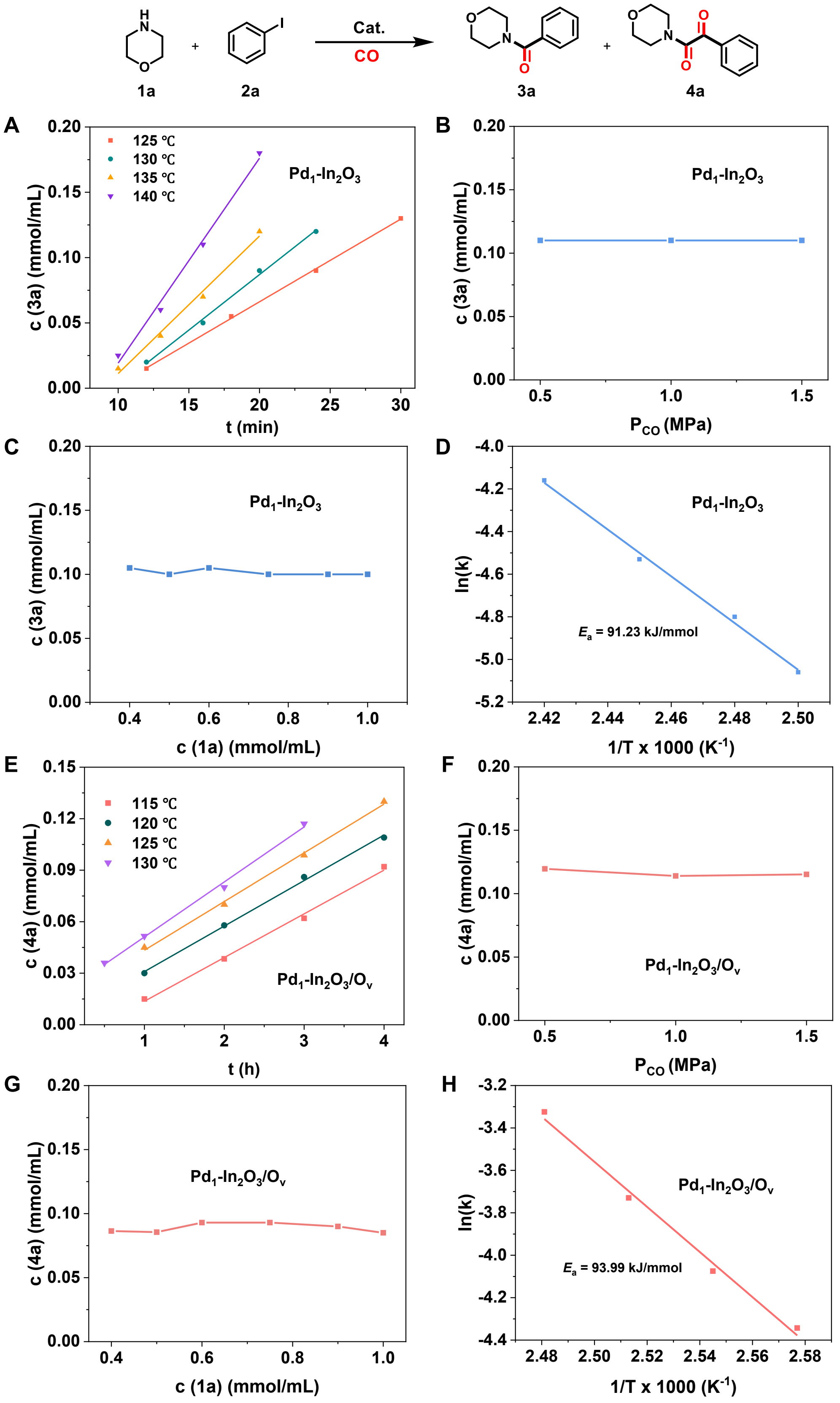

1a
2a
Cat.
CO
3a
4a
A
c (3a) (mmol/mL)
t (min)
125 ℃
130 ℃
135 ℃
140 ℃
Pd1-In2O3
B
c (3a) (mmol/mL)
PCO (MPa)
Pd1-In2O3
C
c (3a) (mmol/mL)
c (1a) (mmol/mL)
Pd1-In2O3
D
ln(k)
1/T x 1000 (K-1)
Ea = 91.23 kJ/mmol
Pd1-In2O3
E
c (4a) (mmol/mL)
t (h)
115 ℃
120 ℃
125 ℃
130 ℃
Pd1-In2O3/Ov
F
c (4a) (mmol/mL)
PCO (MPa)
Pd1-In2O3/Ov
G
c (4a) (mmol/mL)
c (1a) (mmol/mL)
Pd1-In2O3/Ov
H
ln(k)
1/T x 1000 (K-1)
Ea = 93.99 kJ/mmol
Pd1-In2O3/Ov

Fig. S4.

Reaction kinetic studies for the mono- and double-aminocarbonylation. Effect of temperature on rate of mono- and double-aminocarbonylation over Pd_1-In_2O_3 (**A**) and Pd_1-In_2O_3/O_v (**E**). Effect of the pressure of CO on rate of mono- and double-aminocarbonylation over Pd_1-In_2O_3 (**B**) and Pd_1-In_2O_3/O_v (**F**). Effect of the concentration of **1a** on rate of mono- and double-aminocarbonylation over Pd_1-In_2O_3 (**C**) and Pd_1-In_2O_3/O_v (**G**). Arrhenius plot for apparent activation energies of Pd_1-In_2O_3 (**D**) and Pd_1-In_2O_3/O_v (**H**).

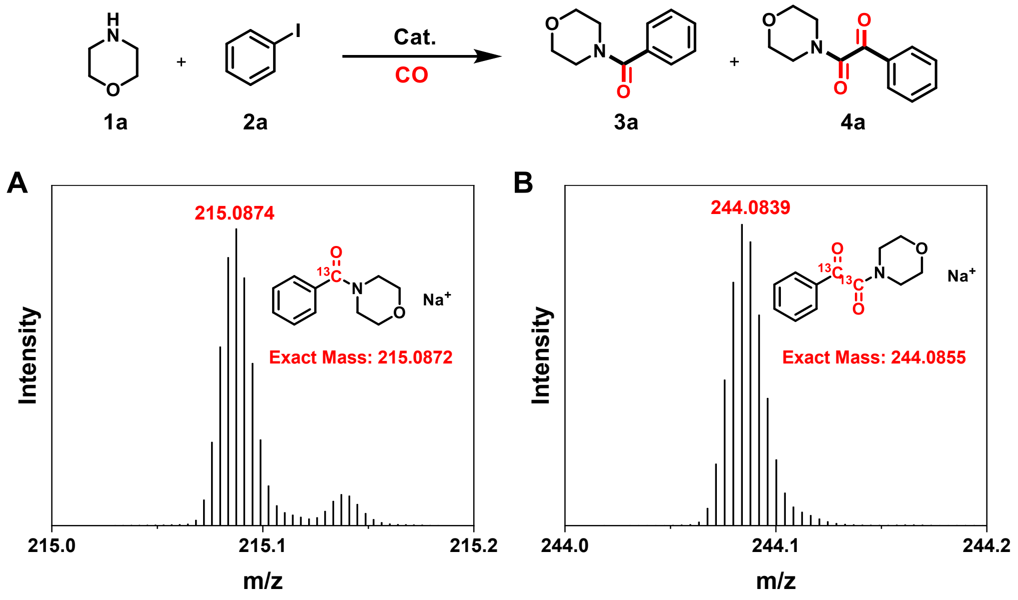

Fig. S5.

Isotope-labeling experiments of ^{13}CO. A, The mono-carbonylation product. **B**, The double-carbonylation product. Reaction conditions: **1a** (1.5 mmol), **2a** (1.0 mmol), K_2CO_3 (2.0 mmol), ^{13}CO (1.0 MPa), 1,4-dioxane (2 mL), 125 °C, 12 h.

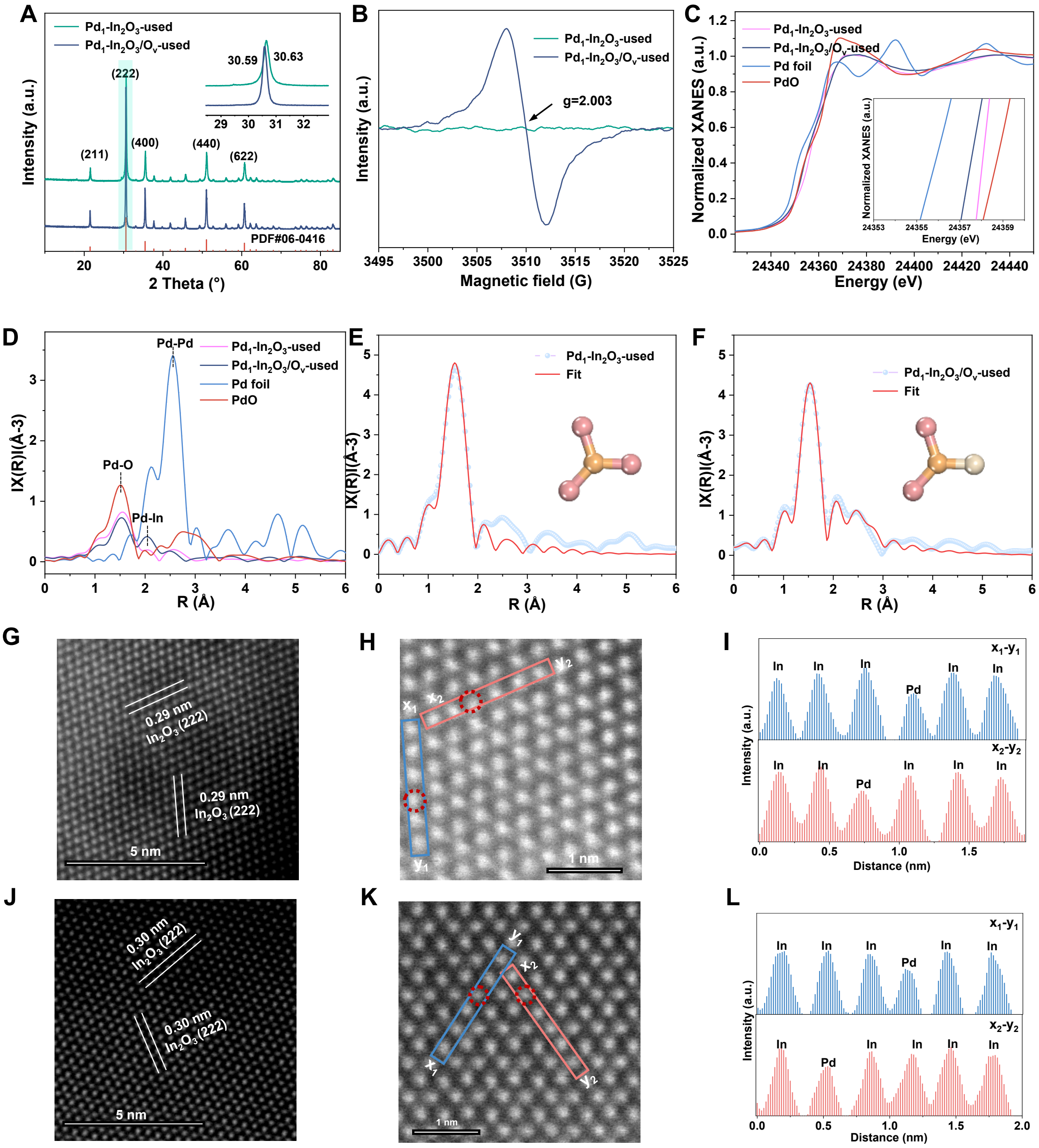

Fig. S6.

The used catalysts characterization of Pd_1-In_2O_3-used and Pd_1-In_2O_3/O_v-used. A, XRD patterns. **B**, EPR spectra. Normalized XANES spectra (**C**), FT-EXAFS spectra (**D**). **E** and **F**, Experimental and best-fitted EXAFS spectra in R space for Pd_1-In_2O_3-used and Pd_1-In_2O_3/O_v-used. **G** and **J**, HAADF-STEM images of Pd_1-In_2O_3 and Pd_1-In_2O_3/O_v. **H** and **K**, AC-HAADF-STEM image of Pd_1-In_2O_3-used and Pd_1-In_2O_3/O_v-used, where the weakly intense Pd atoms are indicated by red circles. **I** and **L**, the line scan measured along the x-y rectangle region marked in (**H**) and (**K**).

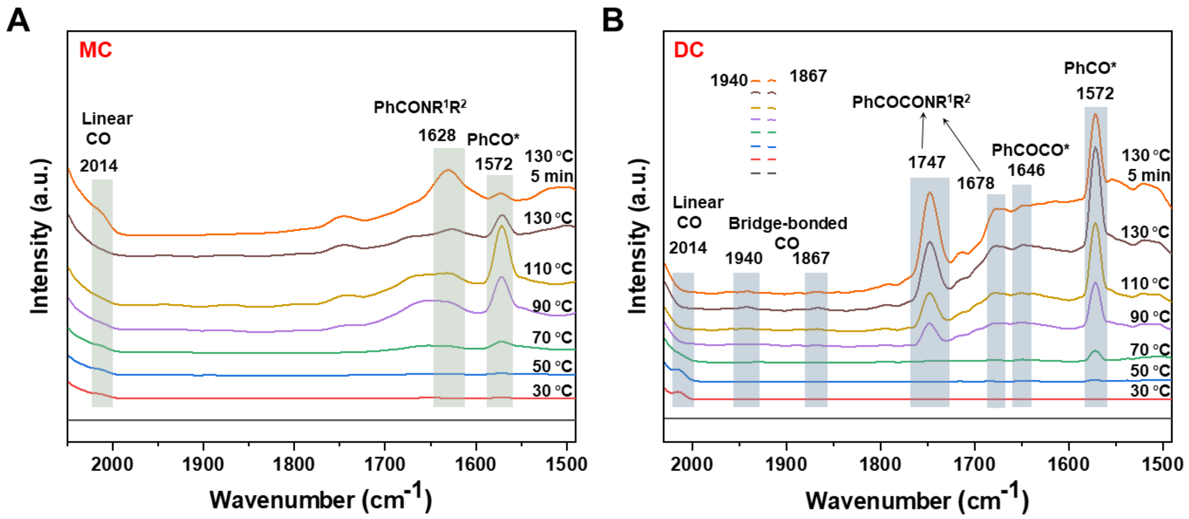

Fig. S7.

Mechanistic studies by DRIFTS. In situ DRIFT spectra for the aminocarbonylation of iodobenzene over Pd_1-In_2O_3 (**A**) and Pd1-In_2O_3/O_v (**B**).

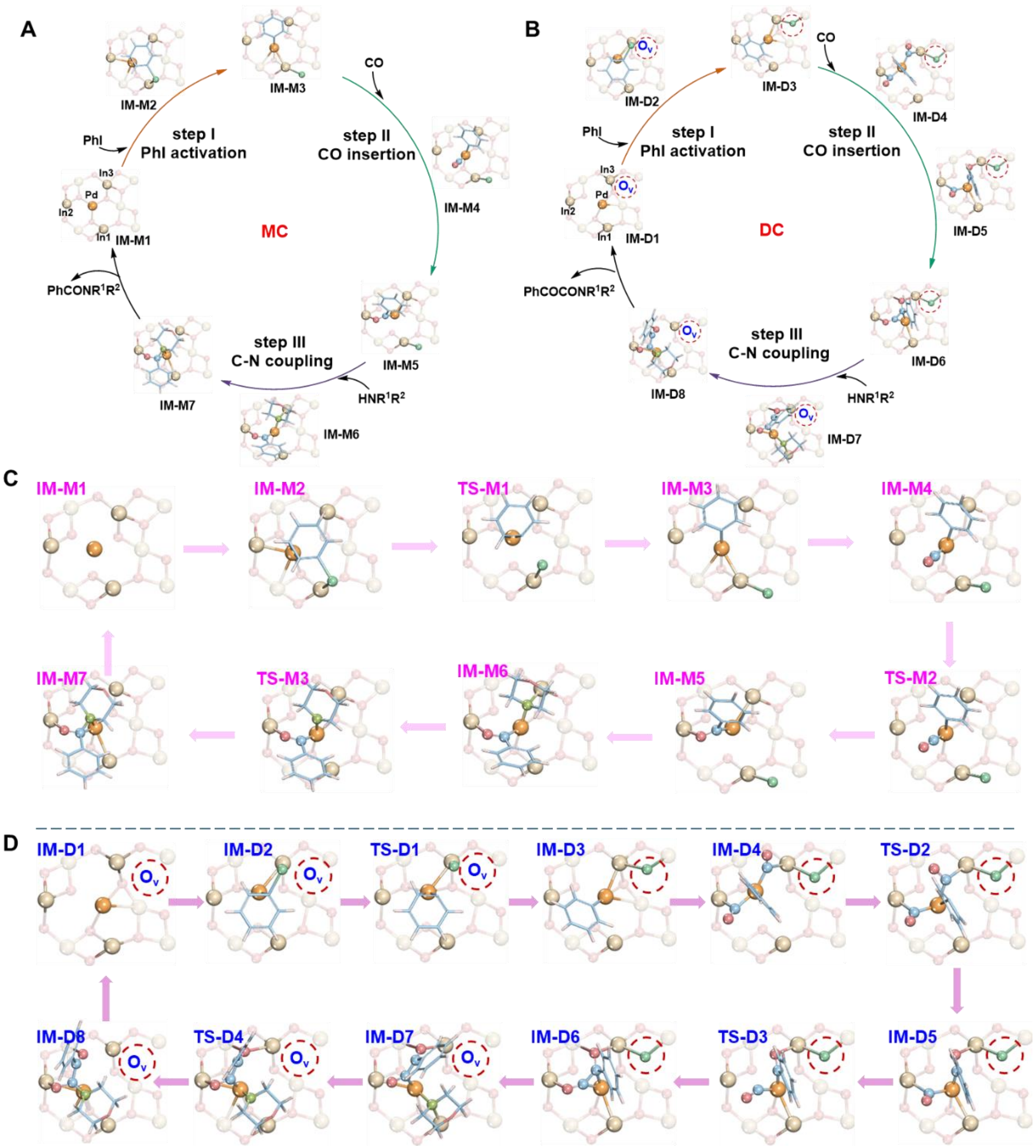

Fig. S8.

Mechanistic studies by DFT. The proposed reaction mechanisms for the aminocarbonylation of iodobenzene by Pd_1-In_2O_3 (left, **A**) and Pd_1-In_2O_3/O_v (right, **B**). The structures of the related potential energy surfaces (PESs). **C** and **D**, PhI aminocarbonylation over Pd_1-In_2O_3 (**MC**) and Pd_1-In_2O_3/O_v (**DC**) surface, respectively.

Table S1.

EXAFS fitting parameters at the Pd K-edge for various samples (S_0^2=0.80).

Sample	Shell	CN[a]	R(Å)[b]	σ^2(Å^2)[c]	ΔE_0(eV)[d]	R factor
Pd foil	Pd-Pd	12	2.74	0.0050	3.956	0.00695
Pd_1-In_2O_3	Pd-O	2.8	2.02	0.0038	4.879	0.00912
Pd_1-In_2O_3/O_v	Pd-O	2.4	2.02	0.0041	7.084	0.01125
	Pd-In	0.8	2.41	0.0041		
Pd_1-In_2O_3-used	Pd-O	2.9	2.02	0.0039	3.620	0.01919
Pd_1-In_2O_3/O_v-used	Pd-O	2.4	2.01	0.0043	7.757	0.02045
	Pd-In	0.8	2.45	0.0121		

[a] CN, coordination number, [b]R, distance between absorber and backscatter atoms, [c]σ^2, Debye-Waller factor to account for both thermal and structural disorders, [d]ΔE_0, inner potential correction, R factor indicates the goodness of the fit. S_0^2 was fixed to 0.80, according to the experimental EXAFS fit of Pd foil by fixing CN as the known crystallographic value.

Table S2.

The Pd content of various samples by ICP-OES.

Sample	Pd content/wt%	Pd leaching/ppm
Pd_1-In_2O_3	0.16	
Pd_1-In_2O_3/O_v	0.17	
Pd_1-In_2O_3-used	0.16	0.00
Pd_1-In_2O_3/O_v-used	0.17	0.00

Table S3.

Catalyst support screening and reaction conditions optimization for carbonylation of iodobenzene and morpholine [a].

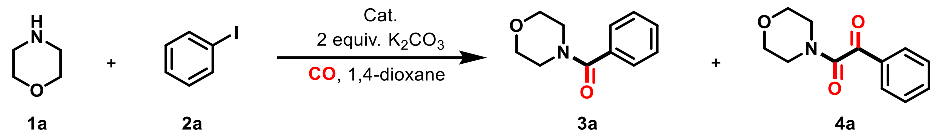

Entry	Cat.	T (ºC)	t (h)	Conv./%	Sel.(**3a**/**4a**)/%
1	In_2O_3/20 mg	135	6	--	--
2	In_2O_3/O_v/20 mg	135	6	--	--
3	0.2%Pd_1-In_2O_3/20 mg	135	6	99	72/28
4	0.2%Pd_1-In_2O_3/O_v/20 mg	135	6	90	24/76
5	0.1%Pd_1-In_2O_3/20 mg	135	6	99	63/37
6	0.1%Pd_1-In_2O_3/O_v/20 mg	135	6	87	29/71
7	0.4%Pd_1-In_2O_3/20 mg	135	6	98	50/50
8	0.4%Pd_1-In_2O_3/O_v/20 mg	135	6	77	31/69
9	0.2%Pd_1-In_2O_3/30 mg	135	6	99	74/26
10	0.2%Pd_1-In_2O_3/O_v/30 mg	135	6	90	27/73
11	0.2%Pd_1-In_2O_3/20 mg	125	9	99	93/7
12	0.2%Pd_1-In_2O_3/O_v/20 mg	125	9	99	8/92
13	10%Pd/C	125	9	99	70/30
14	0.2%Pd_1-In_2O_3/O_v-O_2/20 mg	125	9	99	89/11
15	0.2%Pd_1-In_2O_3/O_v-7days	125	9	99	9/91
16	gram-scale 0.2%Pd_1-In_2O_3/20 mg	125	9	99	93/7
17	gram-scale 0.2%Pd_1-In_2O_3/O_v/20 mg	125	9	99	8/92

[a]Reaction conditions: **1a** (1.5 mmol), **2a** (1.0 mmol), K_2CO_3 (2.0 mmol), CO (1.0 MPa), 1,4-dioxane (2 mL), conversion and selectivity were determined by GC-MS.

Table S4.

Homogeneous and heterogeneous noble metal palladium catalyzed carbonylation of aryl halides using amines with CO.

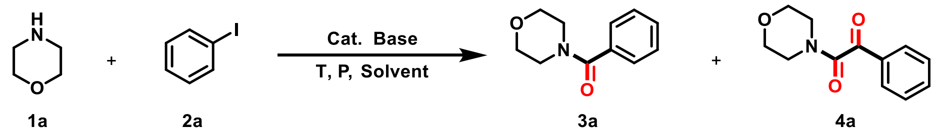

Entry	Cat.	Base	CO/MPa	Conv./%	**3a/4a(%)**	Ref.
1	0.25 mol%Pd-NHC complex	K_2CO_3	2	41	12/88	(*49*)
2	1 mol% $PdCl_2(PPh_2)_2$@SBA-15	K_2CO_3	4	99	13/69	(*15*)
3	0.49 mol% $Pd_2(dba)_3$-SILP	Et_3N	1	96	14/86	(*17*)
4	2 mol% Pd@MOF	Cs_2CO_3	0.1	99	72/28	(*50*)
5	2 mol% $Pd(OAc)_2$	Na_2CO_3	0.1	99	6/94	(*51*)
6	0.78 mol% Pd/CFP	Cs_2CO_3	4	99	49/51	(*52*)

Note: The comparison was intended to illustrate the performance and selectivity under the same model reaction and active metal for both mono- and double carbonylation.

Table S5.

Quantification of oxygen vacancies in Pd_1-In_2O_3/O_v by O_2 titration and EPR[a].

Entry	Cat.	Yield(**4a**)/%	O_2 adsorption/mmol/g	EPR/ Vacancy concentration /10^{17}/mm^3
1	0.2%Pd_1-In_2O_3/O_v-300	22	0.83	3.071
2	0.2%Pd_1-In_2O_3/O_v-350	35	1.22	3.567
3	0.2%Pd_1-In_2O_3/O_v-400	66	1.81	4.786
4	0.2%Pd_1-In_2O_3/O_v	92	3.08	5.733

[a]Reaction conditions: **1a** (1.5 mmol), **2a** (1.0 mmol), K_2CO_3 (2.0 mmol), CO (1.0 MPa), 1,4-dioxane (2 mL), yields were determined by GC-MS.

Table S6.

Time curves and hot filtration for mono-aminocarbonylation of iodobenzene and morpholine[a].

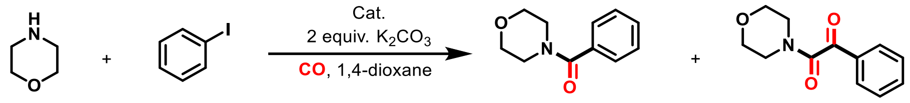

Entry	Cat.	t (h)	Conv./%	Sel.(3a/4a)/%
1	0.2%Pd_1-In_2O_3	0.5	27	94/6
2	0.2%Pd_1-In_2O_3	1	44	95/5
3	0.2%Pd_1-In_2O_3	3	60	93/7
4	0.2%Pd_1-In_2O_3	5	97	91/9
5	0.2%Pd_1-In_2O_3	6	99	94/6
6	0.2%Pd_1-In_2O_3	0.5	27	94/6
7	0.2%Pd_1-In_2O_3	1	44	95/5
8	After filtration	3	44	95/5
9		5	44	95/5
10		6	44	94/6

[a]Reaction conditions: **1a** (1.5 mmol), **2a** (1.0 mmol), K_2CO_3 (2.0 mmol), CO (1.0 MPa), 1,4-dioxane (2 mL), conversion and selectivity were determined by GC-MS.

Table S7.

Time curves and hot filtration for double-aminocarbonylation of iodobenzene and morpholine[a].

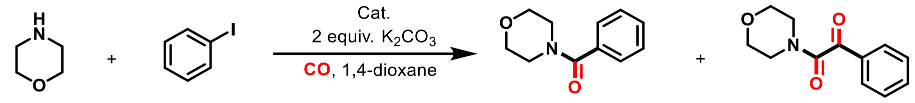

1a 2a 3a 4a

Entry	Cat.	t (h)	Conv./%	Sel.(3a/4a)/%
1	0.2%Pd_1-In_2O_3/O_v	2	24	9/91
2	0.2%Pd_1-In_2O_3/O_v	4	47	9/91
3	0.2%Pd_1-In_2O_3/O_v	6	70	8/92
4	0.2%Pd_1-In_2O_3/O_v	8	92	9/91
5	0.2%Pd_1-In_2O_3/O_v	9	99	8/92
6	0.2%Pd_1-In_2O_3/O_v	2	24	9/91
7	0.2%Pd_1-In_2O_3/O_v	4	48	9/91
8	After filtration	6	48	8/92
9		8	48	8/92
10		9	48	8/92

[a]Reaction conditions: **1a** (1.5 mmol), **2a** (1.0 mmol), K_2CO_3 (2.0 mmol), CO (1.0 MPa), 1,4-dioxane (2 mL), conversion and selectivity were determined by GC-MS.

Table S8.

Substrate scope of mono-aminocarbonylation for alkenyl and ortho-substituted aryl iodides[a].

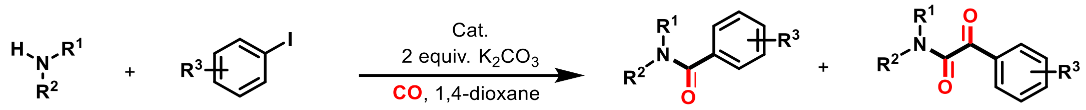

1 2 3 4

Entry	Substrate (1)	Substrate (2)	Conv./%	Sel.(3/4)/%	Yield(3)/%
1	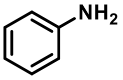	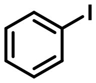	99	100/0	91
2	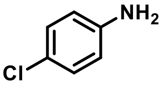	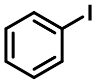	99	100/0	92
3	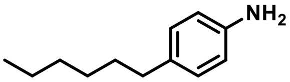	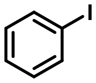	99	100/0	94
4	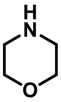	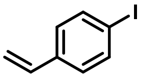	99	98/2	98
5	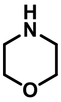	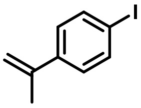	99	94/6	94
6	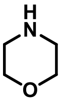	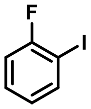	99	100/0	98
7	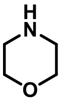	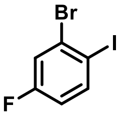	99	100/0	97
8	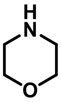	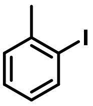	95	100/0	90
9	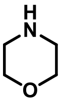	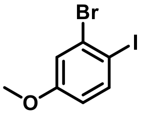	99	100/0	95
10	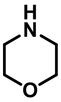	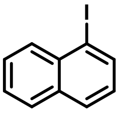	99	100/0	96

[a]Reaction conditions: **1a** (1.5 mmol), **2** (1.0 mmol), 20 mg 0.2%Pd_1-In_2O_3, K_2CO_3 (2.0 mmol), CO (1.0 MPa), 1,4-dioxane (2 mL), conversion and selectivity were determined by GC-MS, isolated yields.

Table S9.

Substrate scope of double-aminocarbonylation for alkenyl and ortho-substituted aryl iodides[a].

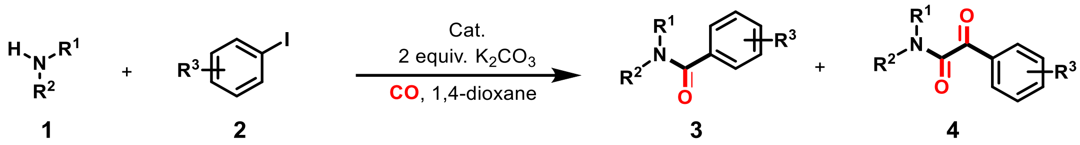

Entry	Substrate (1)	Substrate (2)	Conv./%	Sel.(3/4)/%	Yield (4)/%
1	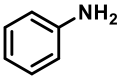	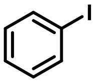	99	100/0	
2	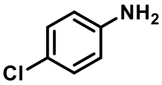	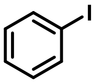	99	100/0	
3	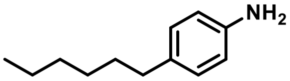	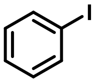	99	100/0	
4	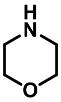	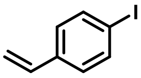	99	13/87	87
5	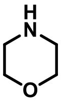	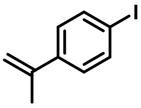	99	9/91	91
6	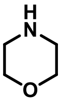	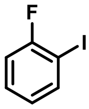	37	100/0	
7	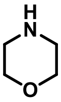	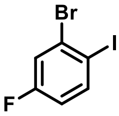	85	100/0	
8	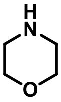	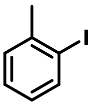	trace	100/0	
9	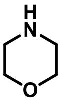	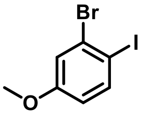	trace	100/0	
10	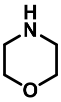	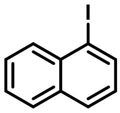	99	72/28	

[a]Reaction conditions: **1a** (1.5 mmol), **2** (1.0 mmol), 20 mg 0.2%Pd_1-In_2O_3/O_v, K_2CO_3 (2.0 mmol), CO (1.0 MPa), 1,4-dioxane (2 mL), conversion and selectivity were determined by GC-MS, isolated yields.

Characterization data for products

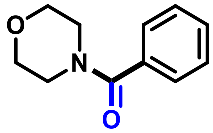

3a morpholino(phenyl)methanone (*38*): The title compound was prepared from iodobenzene (204 mg, 1.0 mmol) and morpholine (130.5 mg, 1.5 mmol) following the general procedure and purified by column chromatography using petroleum ether/diethyl ether (5:1) to afford the product as a white solid (170.0 mg, 89% yield); **^{1}H NMR** (400 MHz, $CDCl_3$) δ 7.43-7.28 (m, 5H), 3.73-3.46 (m, 8H); **^{13}C NMR** (100 MHz, $CDCl_3$) δ 170.3, 135.3, 129.8, 128.5, 127.0, 66.8, 48.1, 42.5.

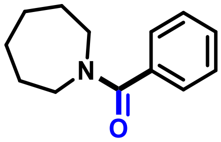

3b azepan-1-yl(phenyl)methanone (*52*): The title compound was prepared from iodobenzene (204 mg, 1.0 mmol) and azepane (148.5 mg, 1.5 mmol) following the general procedure and purified by column chromatography using petroleum ether/diethyl ether (2:1) to afford the product as a yellow oil (190.8 mg, 94% yield); **^{1}H NMR** (400 MHz, $CDCl_3$) δ 7.44 – 7.32 (m, 5H), 3.73 – 3.59 (m, 2H), 3.35 (t, J = 5.6 Hz, 2H), 1.89 – 1.78 (m, 2H), 1.70 – 1.49 (m, 6H); **^{13}C NMR** (100 MHz, $CDCl_3$) δ 171.5, 137.3, 129.0, 128.3, 126.4, 49.7, 46.3, 29.5, 27.8, 27.2, 26.4.

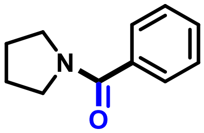

3c phenyl(pyrrolidin-1-yl)methanone (*38*): The title compound was prepared from iodobenzene (204 mg, 1.0 mmol) and pyrrolidine (106.5 mg, 1.5 mmol) following the general procedure and purified by column chromatography using petroleum ether/diethyl ether (5:1) to afford the product as a white solid (152.3 mg, 87% yield); **^{1}H NMR** (400 MHz, $CDCl_3$) δ 7.50 – 7.41 (m, 2H), 7.33 (dd, J = 5.2, 2.0 Hz, 3H), 3.58 (t, J = 7.0 Hz, 2H), 3.36 (t, J = 6.6 Hz, 2H), 1.89 (q, J = 6.3 Hz, 2H), 1.81 (q, J = 6.6 Hz, 2H); **^{13}C NMR** (100 MHz, $CDCl_3$) δ 169.5, 137.0, 129.5, 128.0, 126.8, 49.4, 45.9, 26.2, 24.2.

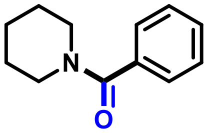

3d phenyl(piperidin-1-yl)methanone (*38*): The title compound was prepared from iodobenzene (204 mg, 1.0 mmol) and piperidine (126.0 mg, 1.5 mmol) following the general procedure and purified by column chromatography using petroleum ether/diethyl ether (5:1) to afford the product as a white solid (182.4 mg, 97% yield); **^{1}H NMR** (400 MHz, $CDCl_3$) δ 7.41-7.35 (m, 5H), 3.70 (s, 2H), 3.32 (s, 2H), 1.66-1.64 (m, 4H), 1.50-1.48 (m, 2H); **^{13}C NMR** (100 MHz, $CDCl_3$) δ 170.0, 136.1, 128.9, 128.0, 126.3, 48.3, 42.7, 26.1, 25.2, 24.1.

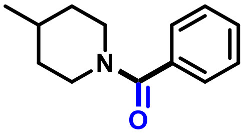

3e (4-methylpiperidin-1-yl)(phenyl)methanone (*53*): The title compound was prepared from iodobenzene (204 mg, 1.0 mmol) and 4-methylpiperidine (148.5 mg, 1.5 mmol) following the general procedure and purified by column chromatography using petroleum ether/diethyl ether (2:1) to afford the product as a white solid (182.7 mg, 90% yield); **^{1}H NMR** (400 MHz, $CDCl_3$) δ 7.38-7.35 (m, 5H), 4.66 (d, J = 13.1 Hz, 1H), 3.68 (d, J = 13.2 Hz, 1H), 3.08 – 2.60 (m, 2H), 1.84 – 1.49 (m, 3H), 1.34 – 1.01 (m, 2H), 0.95 (d, J = 6.4 Hz, 3H); **^{13}C NMR** (100 MHz, $CDCl_3$) δ 170.2, 136.4, 129.2, 128.3, 126.7, 48.0, 42.4, 34.6, 33.8, 31.1, 21.7.

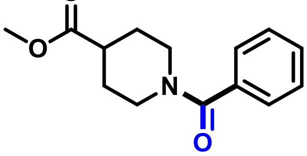

3f methyl 1-benzoylpiperidine-4-carboxylate (*54*): The title compound was prepared from iodobenzene (204 mg, 1.0 mmol) and methyl piperidine-4-carboxylate (214.5 mg, 1.5 mmol) following the

general procedure and purified by column chromatography using petroleum ether/diethyl ether (2:1) to afford the product as a white solid (217.4 mg, 88% yield); ^{1}**H NMR** (400 MHz, $CDCl_3$) δ ^{1}H NMR (400 MHz, Chloroform-*d*) δ 7.38 – 7.29 (m, 5H), 4.48-4.32 (m, 1H), 3.64 (s, 3H), 3.00 (d, *J* = 15.6 Hz, 2H), 2.54 (tt, *J* = 10.8, 4.0 Hz, 1H), 2.11 – 1.50 (m, 4H); ^{13}**C NMR** (100 MHz, $CDCl_3$) δ 174.4, 170.2, 135.8, 129.4, 128.3, 126.6, 51.7, 46.7, 41.3, 40.7, 28.3, 27.8.

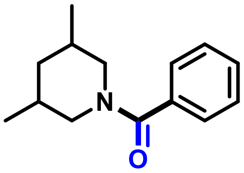

3g (3,5-dimethylpiperidin-1-yl)(phenyl)methanone (*55*): The title compound was prepared from iodobenzene (204 mg, 1.0 mmol) and 3,5-dimethylpiperidine (169.5 mg, 1.5 mmol) following the general procedure and purified by column chromatography using petroleum ether/diethyl ether (2:1) to afford the product as a white solid (195.3 mg, 90% yield); ^{1}**H NMR** (400 MHz, $CDCl_3$) δ 7.38 – 7.31 (m, 5H), 3.74 (s, 2H), 3.58 (t, *J* = 5.4 Hz, 2H), 3.39 (s, 2H), 2.89 (s, 1H), 2.52 (q, *J* = 5.4, 4.6 Hz, 4H), 2.40 (s, 2H); ^{13}**C NMR** (100 MHz, $CDCl_3$) δ 170.1, 135.4, 129.6, 128.3, 126.8, 59.4, 57.8, 52.5, 47.5, 41.9.

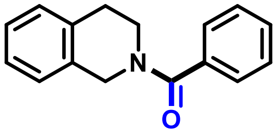

3h (3,4-dihydroisoquinolin-2(1*H*)-yl)(phenyl)methanone (*55*): The title compound was prepared from iodobenzene (204 mg, 1.0 mmol) and 1,2,3,4-tetrahydroisoquinoline (199.5 mg, 1.5 mmol) following the general procedure and purified by column chromatography using petroleum ether/diethyl ether (2:1) to afford the product as a white solid (210.9 mg, 89% yield); ^{1}**H NMR** (400 MHz, $CDCl_3$) δ 7.89 – 7.73 (m, 5H), 7.54 (dt, *J* = 14.6, 7.7 Hz, 4H), 5.27 (s, 1H), 4.95 (s, 1H), 4.36 (s, 1H), 3.99 (t, *J* = 6.1 Hz, 1H), 3.42 – 3.11 (m, 2H); ^{13}**C NMR** (100 MHz, $CDCl_3$) δ 170.7, 135.8, 132.7, 129.5, 128.4, 128.3, 126.9, 126.6, 126.3, 126.3, 49.5, 45.0, 44.6, 40.3, 29.3, 28.0.

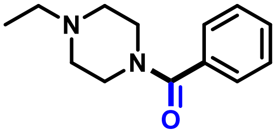

3i (4-ethylpiperazin-1-yl)(phenyl)methanone (*56*): The title compound was prepared from iodobenzene (204 mg, 1.0 mmol) and 1-ethylpiperazine (171.0 mg, 1.5 mmol) following the general procedure and purified by column chromatography using petroleum ether/diethyl ether (2:1) to afford the product as a white solid (191.8 mg, 88% yield); ^{1}**H NMR** (400 MHz, $CDCl_3$) δ 7.42-7.35 (m, 5H), 3.80 (s, 2H), 3.43 (s, 2H), 2.51 – 2.37 (m, 6H), 1.08 (t, *J* = 7.2 Hz, 3H); ^{13}**C NMR** (100 MHz, $CDCl_3$) δ 170.2, 135.8, 129.6, 128.4, 127.0, 53.1, 52.4, 52.2, 47.6, 42.0, 29.6, 11.8.

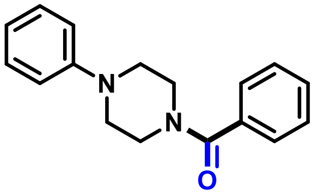

3j phenyl(4-phenylpiperazin-1-yl)methanone (*56*): The title compound was prepared from iodobenzene (204 mg, 1.0 mmol) and 1-phenylpiperazine (243.0 mg, 1.5 mmol) following the general procedure and purified by column chromatography using petroleum ether/diethyl ether (2:1) to afford the product as a white solid (239.4 mg, 90% yield); ^{1}**H NMR** (400 MHz, $CDCl_3$) δ 7.44 – 7.32 (m, 5H), 3.73 – 3.59 (m, 2H), 3.35 (t, *J* = 5.6 Hz, 2H), 1.89 – 1.78 (m, 2H), 1.70 – 1.49 (m, 6H); ^{13}**C NMR** (100 MHz, $CDCl_3$) δ 170.3, 150.8, 135.5, 129.7, 129.1, 128.4, 127.0, 120.5, 116.6, 49.6, 47.5, 42.0, 29.7.

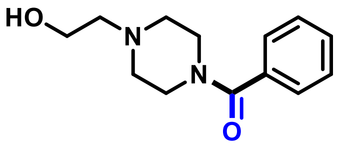

3k (4-(2-hydroxyethyl)piperazin-1-yl)(phenyl)methanone (*57*) :The title compound was prepared from iodobenzene (204 mg, 1.0 mmol) and 2-(piperazin-1-yl)ethan-1-ol (195.0 mg, 1.5 mmol) following the general procedure and purified by column chromatography using petroleum ether/diethyl ether (2:1) to afford the product as a white solid (210.6 mg, 90% yield); ^{1}**H NMR** (400 MHz, $CDCl_3$) δ 7.44 – 7.32 (m, 5H),

3.73 – 3.59 (m, 2H), 3.35 (t, J = 5.6 Hz, 2H), 1.89 – 1.78 (m, 2H), 1.70 – 1.49 (m, 6H); **^{13}C NMR** (100 MHz, $CDCl_3$) δ 170.3, 150.8, 135.5, 129.7, 129.1, 128.4, 127.0, 120.5, 116.6, 49.6, 47.5, 42.0, 29.7.

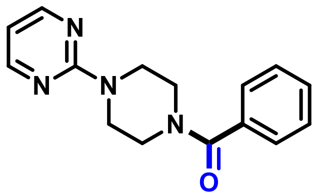

3l phenyl(4-(pyrimidin-2-yl)piperazin-1-yl)methanone (*58*) :The title compound was prepared from iodobenzene (204 mg, 1.0 mmol) and 2-(piperazin-1-yl)pyrimidine (246.0 mg, 1.5 mmol) following the general procedure and purified by column chromatography using petroleum ether/diethyl ether (2:1) to afford the product as a white solid (249.3 mg, 93% yield); **^{1}H NMR** (400 MHz, $CDCl_3$) δ 8.32 – 8.17 (m, 2H), 7.44 – 7.30 (m, 5H), 6.47 (td, J = 4.7, 4.1, 1.7 Hz, 1H), 3.80 (t, J = 26.7 Hz, 6H), 3.45 (s, 2H); **^{13}C NMR** (100 MHz, $CDCl_3$) δ 170.4, 161.3, 157.6, 135.4, 129.7, 128.4, 126.9, 110.3, 47.3, 43.7, 41.9, 29.5.

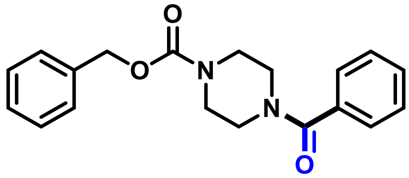

3m benzyl 4-benzoylpiperazine-1-carboxylate (*59*): The title compound was prepared from iodobenzene (204 mg, 1.0 mmol) and benzyl piperazine-1-carboxylate (328.5 mg, 1.5 mmol) following the general procedure and purified by column chromatography using petroleum ether/diethyl ether (2:1) to afford the product as a white solid (285.4 mg, 88% yield); **^{1}H NMR** (400 MHz, $CDCl_3$) δ 7.43 – 7.26 (m, 10H), 5.13 (d, J = 1.8 Hz, 2H), 3.62 (dd, J = 93.6, 31.1 Hz, 8H); **^{13}C NMR** (100 MHz, $CDCl_3$) δ 170.3, 154.8, 136.1, 135.1, 129.7, 128.4, 128.3, 127.9, 127.7, 126.8, 67.2, 47.2, 43.6, 41.8.

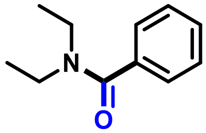

3n *N,N*-diethylbenzamide (*38*): The title compound was prepared from iodobenzene (204 mg, 1.0 mmol) and dimethylamine (109.5 mg, 1.5 mmol) following the general procedure and purified by column chromatography using petroleum ether/diethyl ether (2:1) to afford the product as a yellow oil (171.7 mg, 97% yield); **^{1}H NMR** (400 MHz, $CDCl_3$) δ 7.39-7.36 (m, 5H), 3.55 (q, J = 7.4 Hz, 2H), 3.25 (q, J = 7.4 Hz, 2H), 1.25 (d, J = 7.4 Hz, 3H), 1.11 (d, J = 7.4 Hz, 3H); **^{13}C NMR** (100 MHz, $CDCl_3$) δ 171.1, 137.2, 128.9, 128.2, 126.1, 43.1, 39.1, 14.1, 12.8.

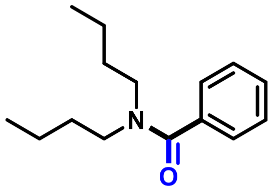

3o *N,N*-dibutylbenzamide (*16*): The title compound was prepared from iodobenzene (204 mg, 1.0 mmol) and dibutylamine (109.5 mg, 1.5 mmol) following the general procedure and purified by column chromatography using petroleum ether/diethyl ether (2:1) to afford the product as a white solid (212.1 mg, 91% yield); **^{1}H NMR** (400 MHz, $CDCl_3$) δ 7.37-7.31 (m, 5H), 3.49-3.45 (m, 2H), 3.18-3.14 (m, 2H), 1.65-1.60 (m, 2H), 1.47-1.37 (m, 4H), 1.12-1.10 (m, 2H), 0.95-0.93 (m, 4H), 0.77-0.74 (m, 3H); **^{13}C NMR** (100 MHz, $CDCl_3$) δ 171.5, 137.3, 128.9, 128.2, 126.3, 48.7, 44.3, 30.7, 29.6, 20.2, 19.6, 13.8, 13.5.

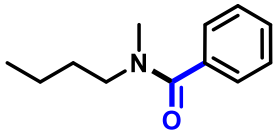

3p *N*-butyl-*N*-methylbenzamide (*55*): The title compound was prepared from iodobenzene (204 mg, 1.0 mmol) and *N*-methylbutan-1-amine (130.5 mg, 1.5 mmol) following the general procedure and purified by column chromatography using petroleum ether/diethyl ether (6:1) to afford the product as a white solid (170.0 mg, 89% yield); **^{1}H NMR** (400 MHz, $CDCl_3$) δ 7.27 (s, 5H), 3.43 (t, J = 7.7 Hz, 1H), 3.12 (t, J = 7.5 Hz, 1H), 2.88 (d, J = 59.8 Hz, 3H), 1.62 – 1.22 (m, 3H), 1.14 – 0.60 (m, 4H); **^{13}C NMR** (100 MHz, $CDCl_3$) δ 171.5, 136.5, 128.8, 127.9, 126.4, 126.2, 50.6, 37.0, 29.9, 19.1, 13.2.

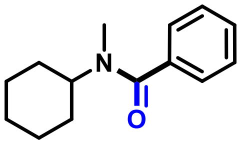

3q *N*-cyclohexyl-*N*-methylbenzamide (*60*): The title compound was prepared from iodobenzene (204 mg, 1.0 mmol) and *N*-methylcyclohexanamine (169.5 mg, 1.5 mmol) following the general procedure and purified by column chromatography using petroleum ether/diethyl ether (6:1) to afford the product as a white solid (208.3 mg, 96% yield); **^{1}H NMR** (400 MHz, $CDCl_3$) δ 7.30-7.24 (m, 5H), 4.46-4.33 (m, 1H), 2.78 (d, *J* = 76.1 Hz, 3H), 1.81 – 1.24 (m, 8H), 0.97-0.92 (m, 2H); **^{13}C NMR** (100 MHz, $CDCl_3$) δ 171.3, 136.9, 128.8, 128.0, 126.4, 125.7, 57.9, 52.4, 30.4, 29.3, 27.1, 25.2, 24.8.

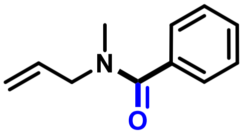

3r *N*-allyl-*N*-methylbenzamide (*61*): The title compound was prepared from iodobenzene (204 mg, 1.0 mmol) and *N*-methylprop-2-en-1-amine (106.5 mg, 1.5 mmol) following the general procedure and purified by column chromatography using petroleum ether/diethyl ether (6:1) to afford the product as a white solid (159.3 mg, 91% yield); **^{1}H NMR** (400 MHz, $CDCl_3$) δ 7.35 (dtd, *J* = 8.4, 6.5, 5.9, 3.2 Hz, 5H), 5.91 – 5.60 (m, 1H), 5.24 – 5.11 (m, 2H), 4.10 (d, *J* = 6.0 Hz, 1H), 3.79 (d, *J* = 5.1 Hz, 1H), 3.07 – 2.77 (m, 3H); **^{13}C NMR** (100 MHz, $CDCl_3$) δ 171.9, 136.0, 132.8, 129.4, 128.1, 126.4, 117.2, 53.7, 36.7.

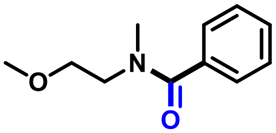

3s *N*-(2-methoxyethyl)-*N*-methylbenzamide (*56*): The title compound was prepared from iodobenzene (204 mg, 1.0 mmol) and 2-methoxy-*N*-methylethan-1-amine (133.5 mg, 1.5 mmol) following the general procedure and purified by column chromatography using petroleum ether/diethyl ether (2:1) to afford the product as a white solid (175.7 mg, 91% yield); **^{1}H NMR** (400 MHz, $CDCl_3$) δ 7.39 – 7.30 (m, 5H), 3.73 – 3.57 (m, 2H), 3.48 – 3.14 (m, 5H), 3.02 (d, *J* = 35.8 Hz, 3H); **^{13}C NMR** (100 MHz, $CDCl_3$) δ 171.3, 136.3, 129.2, 128.1, 126.7, 70.6, 58.7, 47.3, 38.9.

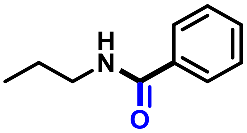

3t *N*-propylbenzamide (*62*): The title compound was prepared from iodobenzene (204 mg, 1.0 mmol) and propan-1-amine (88.5 mg, 1.5 mmol) following the general procedure and purified by column chromatography using petroleum ether/diethyl ether (5:1) to afford the product as a white solid (141.8 mg, 87% yield); **^{1}H NMR** (400 MHz, $CDCl_3$) δ 7.77 (d, *J* = 7.0 Hz, 2H), 7.52 – 7.35 (m, 2H), 6.40 (brs, 1H), 3.41 (q, *J* = 6.7 Hz, 3H), 1.63 (q, *J* = 7.3 Hz, 2H), 0.97 (t, *J* = 7.4 Hz, 3H); **^{13}C NMR** (100 MHz, $CDCl_3$) δ 167.5, 134.8, 131.2, 128.4, 126.8, 41.7, 22.9, 11.4.

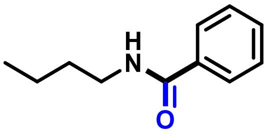

3u *N*-butylbenzamide (*38*): The title compound was prepared from iodobenzene (204 mg, 1.0 mmol) and butan-1-amine (109.5 mg, 1.5 mmol) following the general procedure and purified by column chromatography using petroleum ether/diethyl ether (5:1) to afford the product as a white solid (152.2 mg, 86% yield); **^{1}H NMR** (400 MHz, $CDCl_3$) δ 7.78 (d, *J* = 7.6 Hz, 2H), 7.49 – 7.42 (m, 1H), 7.38 (t, *J* = 7.5 Hz, 2H), 6.67 (brs, 1H), 3.44 – 3.35 (m, 2H), 1.57 (p, *J* = 7.3 Hz, 2H), 1.37 (h, *J* = 7.3 Hz, 2H), 0.92 (t, *J* = 7.3 Hz, 3H); **^{13}C NMR** (100 MHz, $CDCl_3$) δ 167.5, 134.7, 131.2, 128.3, 126.8, 39.7, 31.6, 20.0, 13.7.

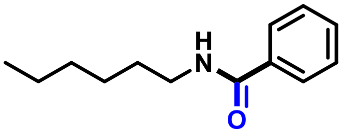

3v *N*-hexylbenzamide (*38*): The title compound was prepared from iodobenzene (204 mg, 1.0 mmol) and hexan-1-amine (151.5 mg, 1.5 mmol) following the general procedure and purified by column chromatography using petroleum ether/diethyl ether (10:1) to afford the product as a white solid (186.6 mg, 91% yield); **^{1}H NMR** (400 MHz, $CDCl_3$) δ 7.79-7.77 (m, 2H), 7.48-7.44 (m, 1H), 7.40-7.36 (m, 2H), 6.65 (brs, 1H), 3.43-

3.38 (m, 2H), 1.62-1.55 (m, 2H), 1.36-1.26 (m, 6H), 0.89 (t, *J* = 7.3 Hz, 3H); ^{13}C **NMR** (100 MHz, $CDCl_3$) δ 167.5, 134.8, 131.0, 128.4, 126.8, 40.1, 31.4, 29.6, 26.6, 22.5, 13.9.

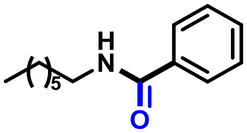

3w *N*-heptylbenzamide (*38*): The title compound was prepared from iodobenzene (204 mg, 1.0 mmol) and heptanamine (172.5 mg, 1.5 mmol) following the general procedure and purified by column chromatography using petroleum ether/diethyl ether (10:1) to afford the product as a white solid (197.1 mg, 90% yield); ^{1}H **NMR** (400 MHz, $CDCl_3$) δ 7.75 (dd, *J* = 7.1, 1.8 Hz, 2H), 7.51 – 7.44 (m, 1H), 7.41 (dd, *J* = 8.2, 6.6 Hz, 2H), 6.28 (brs, 1H), 3.52 – 3.37 (m, 2H), 1.60 (p, *J* = 7.2 Hz, 2H), 1.38 – 1.22 (m, 8H), 0.87 (t, *J* = 6.6 Hz, 3H); ^{13}C **NMR** (100 MHz, $CDCl_3$) δ 167.5, 134.8, 131.2, 128.5, 126.8, 40.1, 31.7, 29.7, 29.0, 26.9, 22.6, 14.0.

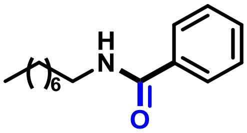

3x *N*-octylbenzamide (*38*): The title compound was prepared from iodobenzene (204 mg, 1.0 mmol) and octylamine (193.5 mg, 1.5 mmol) following the general procedure and purified by column chromatography using petroleum ether/diethyl ether (10:1) to afford the product as a white solid (212.0 mg, 91% yield); ^{1}H **NMR** (400 MHz, $CDCl_3$) δ 7.81 – 7.73 (m, 2H), 7.53 – 7.45 (m, 1H), 7.41 (dd, *J* = 8.3, 6.6 Hz, 2H), 6.29 (brs, 1H), 3.44 (q, *J* = 6.9 Hz, 2H), 1.66 – 1.55 (m, 2H), 1.36 – 1.24 (m, 10H), 0.92 – 0.83 (m, 3H); ^{13}C **NMR** (100 MHz, $CDCl_3$) δ 167.5, 134.9, 131.2, 128.5, 40.1, 31.8, 29.6, 29.3, 29.2, 27.0, 22.6, 14.1.

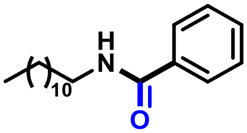

3y *N*-dodecylbenzamide (*63*): The title compound was prepared from iodobenzene (204 mg, 1.0 mmol) and dodecylamine (277.5 mg, 1.5 mmol) following the general procedure and purified by column chromatography using petroleum ether/diethyl ether (10:1) to afford the product as a white solid (268.8 mg, 93% yield); ^{1}H **NMR** (400 MHz, $CDCl_3$) δ 7.77 (d, *J* = 7.1 Hz, 2H), 7.47 (dd, *J* = 8.5, 6.1 Hz, 1H), 7.39 (dd, *J* = 8.3, 6.7 Hz, 2H), 6.47 (brs, 1H), 3.42 (q, *J* = 6.8 Hz, 2H), 1.59 (p, *J* = 7.2 Hz, 2H), 1.37-1.26 (m, 18H), 0.88 (t, *J* = 6.7 Hz, 3H); ^{13}C **NMR** (100 MHz, $CDCl_3$) δ 167.5, 134.8, 131.1, 128.4, 126.8, 40.1, 31.9, 29.6, 29.6, 29.6, 29.5, 29.3, 27.0, 22.6, 14.1.

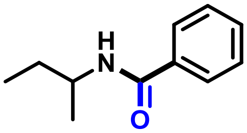

3z *N*-(sec-butyl)benzamide (*38*): The title compound was prepared from iodobenzene (204 mg, 1.0 mmol) and butan-2-amine (109.5 mg, 1.5 mmol) following the general procedure and purified by column chromatography using petroleum ether/diethyl ether (10:1) to afford the product as a white solid (152.2 mg, 86% yield); ^{1}H **NMR** (400 MHz, $CDCl_3$) δ 7.81 – 7.79 (m, 2H), 7.43 (t, *J* = 7.7 Hz, 1H), 7.38 – 7.31 (m, 2H), 7.06 (brs, 1H), 3.26 – 3.15 (m, 2H), 1.88 (dp, *J* = 13.5, 6.8 Hz, 1H), 0.92 (dd, *J* = 6.8, 1.3 Hz, 6H); ^{13}C **NMR** (100 MHz, $CDCl_3$) δ 167.6, 134.7, 130.9, 128.2, 126.8, 47.2, 28.4, 20.0.

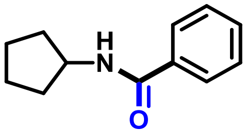

3aa *N*-cyclopentylbenzamide (*38*): The title compound was prepared from iodobenzene (204 mg, 1.0 mmol) and cyclopentanamine (127.5 mg, 1.5 mmol) following the general procedure and purified by column chromatography using petroleum ether/diethyl ether (10:1) to afford the product as a white solid (158.8 mg, 84% yield); ^{1}H **NMR** (400 MHz, $CDCl_3$) δ 7.76 – 7.74 (m, 2H), 7.50 – 7.46 (m, 1H), 7.43 – 7.39 (m, 2H), 6.15 (brs, 1H), 4.40 (h, *J* = 7.0 Hz, 1H), 2.13 – 2.04 (m, 2H), 1.79 – 1.59 (m, 4H), 1.49 (dddt, *J* = 17.2, 8.5, 4.0, 1.7 Hz, 2H); ^{13}C **NMR** (100 MHz, $CDCl_3$) δ 167.1, 134.9, 131.2, 128.4, 126.8, 51.6, 33.2, 23.8.

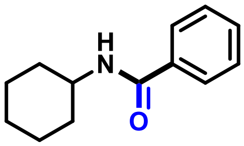

3ab ***N*-cyclohexylbenzamide (*38*):** The title compound was prepared from iodobenzene (204 mg, 1.0 mmol) and cyclohexanamine (148.5 mg, 1.5 mmol) following the general procedure and purified by column chromatography using petroleum ether/diethyl ether (2:1) to afford the product as a white solid (188.8 mg, 93% yield); ^{1}**H NMR** (400 MHz, $CDCl_3$) δ 7.78 – 7.71 (m, 2H), 7.49 – 7.40 (m, 1H), 7.42 – 7.33 (m, 2H), 6.23 (brs, 1H), 3.94 (dddd, *J* = 14.7, 10.7, 8.0, 3.9 Hz, 1H), 1.98 (dt, *J* = 12.2, 3.9 Hz, 2H), 1.79 – 1.67 (m, 2H), 1.67 – 1.56 (m, 1H), 1.46 – 1.30 (m, 2H), 1.29 – 1.09 (m, 3H); ^{13}**C NMR** (100 MHz, $CDCl_3$) δ 166.6, 135.0, 131.1, 128.3, 126.8, 48.6, 33.1, 25.5, 24.9.

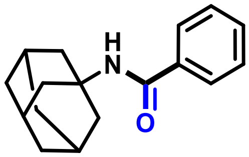

3ac ***N*-(adamantan-1-yl)benzamide (*64*):** The title compound was prepared from iodobenzene (204 mg, 1.0 mmol) and adamantan-1-amine (226.5 mg, 1.5 mmol) following the general procedure and purified by column chromatography using petroleum ether/diethyl ether (6:1) to afford the product as a white solid (237.2 mg, 93% yield); ^{1}**H NMR** (400 MHz, $CDCl_3$) δ ^{1}H NMR (400 MHz, Chloroform-*d*) δ 7.73 – 7.65 (m, 2H), 7.43 – 7.37 (m, 1H), 7.36 – 7.29 (m, 2H), 5.94 (brs, 1H), 2.13 – 2.02 (m, 9H), 1.67 (t, *J* = 2.5 Hz, 6H); ^{13}**C NMR** (100 MHz, $CDCl_3$) δ 166.4, 135.8, 130.7, 128.1, 126.5, 52.0, 41.4, 36.2, 29.3.

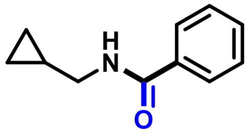

3ad ***N*-(cyclopropylmethyl)benzamide (*65*):** The title compound was prepared from iodobenzene (204 mg, 1.0 mmol) and cyclopropylmethanamine (106.5 mg, 1.5 mmol) following the general procedure and purified by column chromatography using petroleum ether/diethyl ether (6:1) to afford the product as a white solid (161.0 mg, 92% yield); ^{1}**H NMR** (400 MHz, $CDCl_3$) δ 7.81 – 7.74 (m, 2H), 7.44 – 7.38 (m, 1H), 7.36 – 7.30 (m, 2H), 6.92 (brs, 1H), 3.23 (dd, *J* = 7.1, 5.5 Hz, 2H), 1.01 (ddt, *J* = 12.4, 7.6, 4.6 Hz, 1H), 0.49 – 0.41 (m, 2H), 0.19 (dt, *J* = 6.2, 4.6 Hz, 2H); ^{13}**C NMR** (100 MHz, $CDCl_3$) δ 167.4, 134.5, 131.0, 128.2, 126.8, 44.6, 10.6, 3.3.

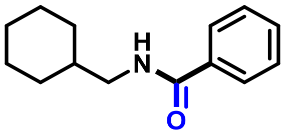

3ae ***N*-(cyclohexylmethyl)benzamide (*66*):** The title compound was prepared from iodobenzene (204 mg, 1.0 mmol) and cyclohexylmethanamine (169.5 mg, 1.5 mmol) following the general procedure and purified by column chromatography using petroleum ether/diethyl ether (4:1) to afford the product as a white solid (208.3 mg, 96% yield); ^{1}**H NMR** (400 MHz, $CDCl_3$) δ 7.80 – 7.75 (m, 2H), 7.38 (t, *J* = 7.4 Hz, 1H), 7.30 (t, *J* = 7.6 Hz, 2H), 7.17 (brs, 1H), 3.18 (t, *J* = 6.5 Hz, 2H), 1.74 – 1.45 (m, 6H), 1.20 – 1.03 (m, 3H), 0.92-0.82 (m, 2H); ^{13}**C NMR** (100 MHz, $CDCl_3$) δ 167.6, 134.6, 130.8, 128.1, 126.8, 46.0, 37.7, 30.7, 26.2, 25.6.

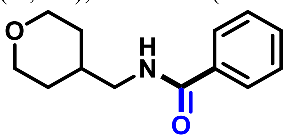

3af ***N*-((tetrahydro-2*H*-pyran-4-yl)methyl)benzamide (*66*):** The title compound was prepared from iodobenzene (204 mg, 1.0 mmol) and (tetrahydro-2H-pyran-4-yl)methanamine (172.5 mg, 1.5 mmol) following the general procedure and purified by column chromatography using petroleum ether/diethyl ether (3:1) to afford the product as a white solid (208.2 mg, 95% yield); ^{1}**H NMR** (400 MHz, $CDCl_3$) δ 7.78 – 7.73 (m, 2H), 7.52 – 7.46 (m, 1H), 7.45 – 7.38 (m, 2H), 6.43 (brs, 1H), 4.04 – 3.87 (m, 2H), 3.43 – 3.30 (m, 4H), 1.87 (tdq, *J* = 10.8, 7.0, 3.5 Hz, 1H), 1.65 (ddq, *J* = 13.0, 4.0, 2.0 Hz, 2H), 1.36 (dtd, *J* = 13.3, 11.8, 4.5 Hz, 2H); ^{13}**C NMR** (100 MHz, $CDCl_3$) δ 167.0, 134.5, 131.4, 128.5, 126.8, 67.6, 45.6, 35.3, 30.6.

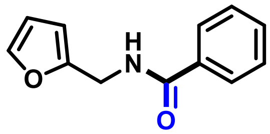

3ag ***N*-(furan-2-ylmethyl)benzamide (*66*):** The title compound was prepared from iodobenzene (204 mg, 1.0 mmol) and furan-2-ylmethanamine (145.5 mg, 1.5 mmol) following the general procedure and purified by column chromatography using petroleum ether/diethyl ether (3:1) to afford the product as a white solid (197.0 mg, 98% yield); **^{1}H NMR** (400 MHz, $CDCl_3$) δ 7.82 – 7.75 (m, 2H), 7.53 – 7.46 (m, 1H), 7.45 – 7.36 (m, 3H), 6.56 (brs, 1H), 6.33 (dd, *J* = 3.2, 1.9 Hz, 1H), 6.29 (dd, *J* = 3.2, 0.9 Hz, 1H), 4.63 (d, *J* = 5.4 Hz, 2H); **^{13}C NMR** (100 MHz, $CDCl_3$) δ 167.2, 151.2, 142.3, 134.1, 131.6, 128.5, 127.0, 37.0.

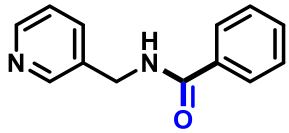

3ah ***N*-(pyridin-3-ylmethyl)benzamide (*66*):** The title compound was prepared from iodobenzene (204 mg, 1.0 mmol) and pyridin-3-ylmethanamine (162.0 mg, 1.5 mmol) following the general procedure and purified by column chromatography using petroleum ether/diethyl ether (5:1) to afford the product as a white solid (188.7 mg, 89% yield); **^{1}H NMR** (400 MHz, $CDCl_3$) δ 8.44 – 8.31 (m, 2H), 8.23 – 8.14 (m, 1H), 7.81 (d, *J* = 7.7 Hz, 2H), 7.60-7.57 (m, 1H), 7.48 – 7.38 (m, 1H), 7.34 – 7.29 (m, 2H), 7.16 – 7.13 (m, 1H), 4.51 (dt, *J* = 5.5, 2.5 Hz, 2H); **^{13}C NMR** (100 MHz, $CDCl_3$) δ 167.8, 148.7, 148.2, 135.4, 134.2, 133.8, 131.4, 128.2, 127.0, 123.4, 41.0.

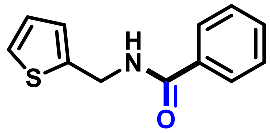

3ai ***N*-(thiophen-2-ylmethyl)benzamide (*66*):** The title compound was prepared from iodobenzene (204 mg, 1.0 mmol) and thiophen-2-ylmethanamine (169.5 mg, 1.5 mmol) following the general procedure and purified by column chromatography using petroleum ether/diethyl ether (5:1) to afford the product as a white solid (199.7 mg, 92% yield); **^{1}H NMR** (400 MHz, $CDCl_3$) δ 7.81 – 7.76 (m, 2H), 7.51 – 7.45 (m, 1H), 7.41 – 7.37 (m, 2H), 7.22 (dd, *J* = 5.1, 1.3 Hz, 1H), 7.00 (dt, *J* = 3.3, 1.0 Hz, 1H), 6.94 (dd, *J* = 5.1, 3.5 Hz, 1H), 6.87 (brs, 1H), 4.77 (dd, *J* = 5.6, 0.9 Hz, 2H); **^{13}C NMR** (100 MHz, $CDCl_3$) δ 167.2, 140.8, 134.0, 131.5, 128.4, 127.0, 126.8, 125.2, 38.7.

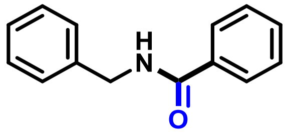

3aj ***N*-benzylbenzamide (*38*):** The title compound was prepared from iodobenzene (204 mg, 1.0 mmol) and phenylmethanamine (160.5 mg, 1.5 mmol) following the general procedure and purified by column chromatography using petroleum ether/diethyl ether (5:1) to afford the product as a white solid (179.4 mg, 95% yield); **^{1}H NMR** (400 MHz, $CDCl_3$) δ 7.84-7.74 (m, 2H), 7.53-7.45 (m, 1H), 7.42 (m, 2H), 7.36-7.29 (m, 5H), 6.49 (brs, 1H), 4.64 (d, *J* = 5.7 Hz, 2H); **^{13}C NMR** (100 MHz, $CDCl_3$) δ 167.3, 138.2, 134.3, 131.5, 128.7, 128.5, 127.8, 127.5, 126.9, 44.1.

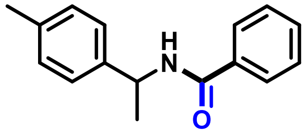

3ak ***N*-(1-(p-tolyl)ethyl)benzamide (*67*):** The title compound was prepared from iodobenzene (204 mg, 1.0 mmol) and 1-(p-tolyl)ethan-1-amine (202.5 mg, 1.5 mmol) following the general procedure and purified by column chromatography using petroleum ether/diethyl ether (10:1) to afford the product as a white solid (215.2 mg, 90% yield); **^{1}H NMR** (400 MHz, $CDCl_3$) δ 7.84 – 7.76 (m, 2H), 7.49 – 7.43 (m, 1H), 7.40 – 7.34 (m, 2H), 7.32 – 7.27 (m, 2H), 7.16 (d, *J* = 7.9 Hz, 2H), 6.86 (s, 1H), 5.30 (p, *J* = 7.1 Hz, 1H), 2.35 (s, 3H), 1.58 (d, *J* = 6.9 Hz, 3H); **^{13}C NMR** (100 MHz, $CDCl_3$) δ 166.5, 140.2, 136.7, 134.5, 131.1, 129.1, 128.2, 126.9, 126.0, 48.8, 21.6, 20.9.

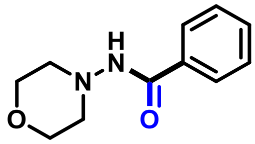

3al *N*-morpholinobenzamide (*68*): The title compound was prepared from iodobenzene (204 mg, 1.0 mmol) and morpholin-4-amine (153.0 mg, 1.5 mmol) following the general procedure and purified by column chromatography using petroleum ether/diethyl ether (2:1) to afford the product as a white solid (195.7 mg, 95% yield); **^{1}H NMR** (400 MHz, $CDCl_3$) δ 7.41 - 7.26 (m, 5H), 3.82 - 3.42 (m, 8H); **^{13}C NMR** (100 MHz, $CDCl_3$) δ 170.4, 135.2, 129.8, 128.5, 127.0, 66.8, 48.1, 42.5.

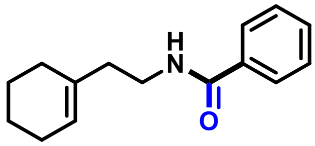

3am *N*-(2-(cyclohex-1-en-1-yl)ethyl)benzamide (*69*): The title compound was prepared from iodobenzene (204 mg, 1.0 mmol) and 2-(cyclohex-1-en-1-yl)ethan-1-amine (187.5 mg, 1.5 mmol) following the general procedure and purified by column chromatography using petroleum ether/diethyl ether (2:1) to afford the product as a white solid (208.4 mg, 91% yield); **^{1}H NMR** (400 MHz, $CDCl_3$) δ 7.76 – 7.69 (m, 2H), 7.48 – 7.41 (m, 1H), 7.39 – 7.34 (m, 2H), 6.46 (s, 1H), 5.49 (tt, *J* = 3.8, 1.6 Hz, 1H), 3.48 (td, *J* = 6.9, 5.4 Hz, 2H), 2.26 – 2.16 (m, 2H), 1.96 (dddd, *J* = 21.3, 10.7, 5.7, 3.0 Hz, 4H), 1.66 – 1.47 (m, 4H); **^{13}C NMR** (100 MHz, $CDCl_3$) δ 167.3, 134.8, 134.7, 131.1, 128.4, 126.7, 123.5, 37.6, 37.5, 27.8, 25.1, 22.7, 22.2.

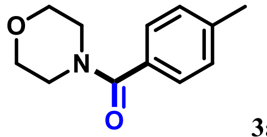

3an morpholino(*p*-tolyl)methanone (*38*): The title compound was prepared from 1-iodo-4-methylbenzene (218 mg, 1.0 mmol) and morpholine (130.5 mg, 1.5 mmol) following the general procedure and purified by column chromatography using petroleum ether/diethyl ether (2:1) to afford the product as a white solid (192.7 mg, 94% yield); **^{1}H NMR** (400 MHz, $CDCl_3$) δ 7.32 – 7.29 (m, 2H), 7.23 – 7.17 (m, 2H), 3.70 – 3.48 (m, 8H), 2.37 (s, 3H); **^{13}C NMR** (100 MHz, $CDCl_3$) δ 170.3, 139.7, 132.1, 128.8, 126.9, 66.6, 48.0, 42.4, 21.1.

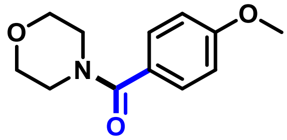

3ao (4-methoxyphenyl)(morpholino)methanone (*38*): The title compound was prepared from 1-iodo-4-methoxybenzene (234 mg, 1.0 mmol) and morpholine (130.5 mg, 1.5 mmol) following the general procedure and purified by column chromatography using petroleum ether/diethyl ether (2:1) to afford the product as a white solid (207.8 mg, 94% yield); **^{1}H NMR** (400 MHz, $CDCl_3$) δ 7.30 – 7.26 (m, 2H), 6.83 – 6.81 (m, 2H), 3.72 (s, 3H), 3.67 – 3.53 (m, 8H); **^{13}C NMR** (100 MHz, $CDCl_3$) δ 170.1, 160.6, 128.9, 126.9, 113.4, 66.5, 55.1.

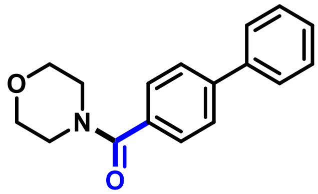

3ap [1,1'-biphenyl]-4-yl(morpholino)methanone (*55*): The title compound was prepared from 4-iodo-1,1'-biphenyl (281 mg, 1.0 mmol) and morpholine (130.5 mg, 1.5 mmol) following the general procedure and purified by column chromatography using petroleum ether/diethyl ether (2:1) to afford the product as a white solid (256.3 mg, 96% yield); **^{1}H NMR** (400 MHz, $CDCl_3$) δ 7.64 - 7.54 (m, 4H), 7.50 - 7.40 (m, 4H), 7.38 - 7.33 (m, 1H), 3.95 - 3.37 (m, 8H); **^{13}C NMR** (100 MHz, $CDCl_3$) δ 170.0, 142.6, 139.9, 133.9, 128.7, 127.6, 127.5, 127.0, 126.9, 66.7, 48.1, 42.4.

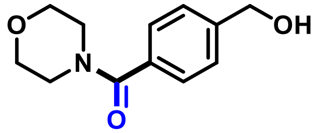

3aq (4-(hydroxymethyl)phenyl)(morpholino)methanone (*56*): The title compound was prepared from (4-iodophenyl)methanol (234 mg, 1.0 mmol) and morpholine (130.5 mg, 1.5 mmol) following the general procedure and purified by column chromatography using petroleum ether/diethyl ether (2:1) to afford the

product as a white solid (210.0 mg, 95% yield); **^{1}H NMR** (400 MHz, $CDCl_3$) δ 7.41 - 7.29 (m, 5H), 7.45 (s, 2H), 3.73 - 3.41 (m, 8H); **^{13}C NMR** (100 MHz, $CDCl_3$) δ 170.3, 143.5, 133.3, 126.8, 126.3, 66.5, 63.6, 63.6, 48.0, 42.3.

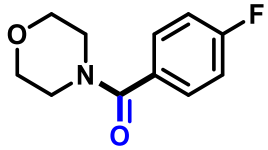

3ar (4-fluorophenyl)(morpholino)methanone (*38*): The title compound was prepared from 1-fluoro-4-iodobenzene (222 mg, 1.0 mmol) and morpholine (130.5 mg, 1.5 mmol) following the general procedure and purified by column chromatography using petroleum ether/diethyl ether (2:1) to afford the product as a white solid (198.6 mg, 95% yield); **^{1}H NMR** (400 MHz, $CDCl_3$) δ 7.44-7.41 (m, 2H), 7.13-7.08 (m, 2H), 3.70-3.47 (m, 8H); **^{13}C NMR** (100 MHz, $CDCl_3$) δ 169.4, 163.4 (d, J = 250.1 Hz), 131.22 (d, J = 3.5 Hz), 129.4 (d, J = 8.5 Hz), 115.6 (d, J = 21.9 Hz), 48.2, 42.7. **^{19}F NMR**(400 MHz, $CDCl_3$) δ -110.00.

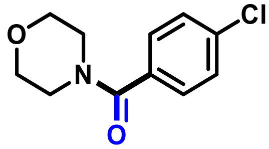

3as (4-chlorophenyl)(morpholino)methanone (*38*): The title compound was prepared from 1-chloro-4-iodobenzene (238 mg, 1.0 mmol) and morpholine (130.5 mg, 1.5 mmol) following the general procedure and purified by column chromatography using petroleum ether/diethyl ether (2:1) to afford the product as a white solid (200.3 mg, 89% yield); **^{1}H NMR** (400 MHz, $CDCl_3$) δ 7.33 - 7.30 (m, 2H), 7.29 – 7.26 (m, 2H), 3.67-3.36 (m, 8H); **^{13}C NMR** (100 MHz, $CDCl_3$) δ 169.0, 135.6, 133.4, 128.6, 128.4, 66.5, 47.9, 42.4.

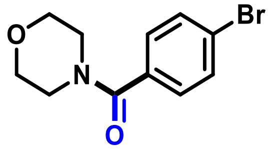

3at (4-bromophenyl)(morpholino)methanone (*70*): The title compound was prepared from 1-bromo-4-iodobenzene (282 mg, 1.0 mmol) and morpholine (130.5 mg, 1.5 mmol) following the general procedure and purified by column chromatography using petroleum ether/diethyl ether (2:1) to afford the product as a white solid (240.3 mg, 89% yield); **^{1}H NMR** (400 MHz, $CDCl_3$) δ 7.50-7.48 (m, 2H), 7.24-7.22 (m, 2H), 3.68-3.37 (m, 8H); **^{13}C NMR** (100 MHz, $CDCl_3$) δ 169.1, 133.9, 131.6, 128.7, 124.0, 66.6, 48.0, 42.4.

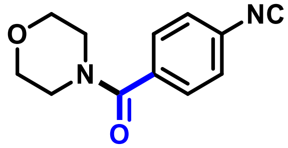

3au 4-(morpholine-4-carbonyl)benzonitrile (*38*): The title compound was prepared from 4-iodobenzonitrile (229 mg, 1.0 mmol) and morpholine (130.5 mg, 1.5 mmol) following the general procedure and purified by column chromatography using petroleum ether/diethyl ether (1:1) to afford the product as a white solid (205.3 mg, 95% yield); **^{1}H NMR** (400 MHz, $CDCl_3$) δ 7.72-7.70 (m, 2H), 7.50-7.48 (m, 2H), 3.76-3.36 (m, 8H); **^{13}C NMR** (100 MHz, $CDCl_3$) δ 168.2, 139.5, 127.7, 117.9, 113.6, 66.6, 47.9, 42.5.

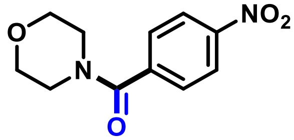

3av morpholino(4-nitrophenyl)methanone (*70*): The title compound was prepared from 1-iodo-4-nitrobenzene (249 mg, 1.0 mmol) and morpholine (130.5 mg, 1.5 mmol) following the general procedure and purified by column chromatography using petroleum ether/diethyl ether (2:1) to afford the product as a white solid (221.8 mg, 94% yield); **^{1}H NMR** (400 MHz, $CDCl_3$) δ 8.19 – 8.11 (m, 2H), 7.53 – 7.45 (m, 2H), 3.74 – 3.25 (m, 8H); **^{13}C NMR** (100 MHz, $CDCl_3$) δ 170.0, 142.6, 139.9, 133.9, 128.7, 127.6, 127.5, 127.0, 126.9, 66.7, 48.1, 42.4.

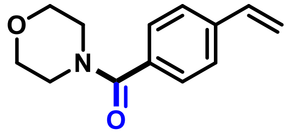

3aw morpholino(4-vinylphenyl)methanone (*71*): The title compound was prepared from 1-iodo-4-vinylbenzene (230 mg, 1.0 mmol) and morpholine (130.5 mg, 1.5 mmol) following the general

procedure and purified by column chromatography using petroleum ether/diethyl ether (2:1) to afford the product as a white solid (212.7 mg, 98% yield); ^{1}H NMR (400 MHz, $CDCl_3$) δ 7.44 – 7.37 (m, 2H), 7.37 – 7.31 (m, 2H), 6.68 (ddd, *J* = 18.1, 10.8, 2.9 Hz, 1H), 5.76 (dd, *J* = 17.6, 2.6 Hz, 1H), 5.29 (dd, *J* = 10.8, 2.9 Hz, 1H), 3.56 (d, *J* = 91.2 Hz, 8H). **^{13}C NMR** (100 MHz, $CDCl_3$) δ 170.01, 138.98, 135.84, 135.78, 134.26, 127.42, 127.33, 126.12, 115.31, 48.07, 42.49. HRMS (ESI) $[M+Na]^+$: calculated for $[C_{13}H_{15}NNaO_2]^+$: 217.1103, found: 240.0995.

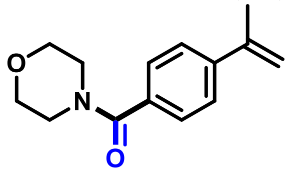

3ax morpholino(4-(prop-1-en-2-yl)phenyl)methanone (*72*): The title compound was prepared from 1-iodo-4-(prop-1-en-2-yl)benzene (244 mg, 1.0 mmol) and morpholine (130.5 mg, 1.5 mmol) following the general procedure and purified by column chromatography using petroleum ether/diethyl ether (2:1) to afford the product as a white solid (217.2 mg, 94% yield); **^{1}H NMR** (400 MHz, $CDCl_3$) δ 7.53 – 7.45 (m, 2H), 7.37 (dd, *J* = 9.5, 3.1 Hz, 2H), 5.40 (s, 1H), 5.13 (q, *J* = 1.5 Hz, 1H), 3.57 (d, *J* = 84.7 Hz, 8H), 2.23 – 2.07 (m, 3H). δ 8.19 – 8.11 (m, 2H), 7.53 – 7.45 (m, 2H), 3.74 – 3.25 (m, 8H); **^{13}C NMR** (100 MHz, $CDCl_3$) δ 169.77, 142.33, 141.98, 133.71, 126.94, 126.89, 126.85, 126.78, 125.21, 125.15, 125.11, 125.07, 113.29, 66.52, 66.43, 47.83, 42.23, 21.30, 21.22. HRMS (ESI) $[M+Na]^+$: calculated for $[C_{14}H_{17}NNaO_2]^+$: 231.2950, found: 254.1151.

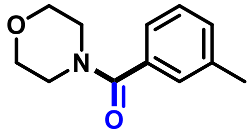

3ay morpholino(m-tolyl)methanone (*55*): The title compound was prepared from 1-iodo-3-methylbenzene (218 mg, 1.0 mmol) and morpholine (130.5 mg, 1.5 mmol) following the general procedure and purified by column chromatography using petroleum ether/diethyl ether (1:1) to afford the product as a white solid (182.5 mg, 89% yield); **^{1}H NMR** (400 MHz, $CDCl_3$) δ 7.32 – 7.26 (m, 1H), 7.24 – 7.20 (m, 2H), 7.17 (d, *J* = 7.5 Hz, 1H), 3.93 – 3.36 (m, 8H), 2.37 (s, 3H); **^{13}C NMR** (100 MHz, $CDCl_3$) δ 170.4, 138.3, 135.1, 130.4, 128.2, 127.5, 123.8, 66.7, 48.0, 42.3, 21.2.

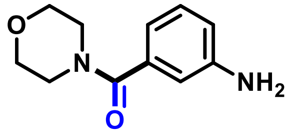

3az (3-aminophenyl)(morpholino)methanone (*73*): The title compound was prepared from 3-iodoaniline (219 mg, 1.0 mmol) and morpholine (130.5 mg, 1.5 mmol) following the general procedure and purified by column chromatography using petroleum ether/diethyl ether (0.5:1) to afford the product as a white solid (193.7 mg, 94% yield); **^{1}H NMR** (400 MHz, $CDCl_3$) δ 7.26 – 6.97 (m, 1H), 6.56 – 6.52 (m, 3H), 3.93 (brs, 2H), 3.93 – 3.30 (m, 8H); **^{13}C NMR** (100 MHz, $CDCl_3$) δ 170.3, 146.8, 135.8, 128.9, 115.7, 115.7, 112.8, 66.4, 47.7, 42.0.

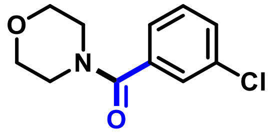

3ba (3-chlorophenyl)(morpholino)methanone (*55*): The title compound was prepared from 1-chloro-3-iodobenzene (238 mg, 1.0 mmol) and morpholine (130.5 mg, 1.5 mmol) following the general procedure and purified by column chromatography using petroleum ether/diethyl ether (2:1) to afford the product as a white solid (216.1 mg, 96% yield); **^{1}H NMR** (400 MHz, $CDCl_3$) δ 7.34 - 7.31 (m, 2H), 7.30 – 7.26 (m, 1H), 7.22 – 7.19 (m, 1H), 3.68-3.35 (m, 8H); **^{13}C NMR** (100 MHz, $CDCl_3$) δ 168.4, 136.7, 134.3, 129.7, 127.0, 124.9, 66.44, 47.8, 42.3.

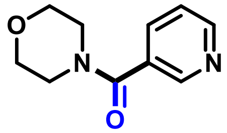

3bb morpholino(pyridin-3-yl)methanone (*55*): The title compound was prepared from 3-iodopyridine (205 mg, 1.0 mmol) and morpholine (130.5 mg, 1.5 mmol) following the general procedure and purified by column chromatography using petroleum ether/diethyl ether (1:1) to afford the product as a white solid (180.5 mg, 94% yield); **^{1}H NMR** (400 MHz, $CDCl_3$) δ 8.61 - 8.59 (m, 2H), 7.72 – 7.68 (m, 1H), 7.33 – 7.26 (m, 1H), 3.74 – 3.36 (m, 8H); **^{13}C NMR** (100 MHz, $CDCl_3$) δ 167.6, 150.8, 147.8, 134.9, 130.1, 123.3, 66.6, 48.1, 42.5.

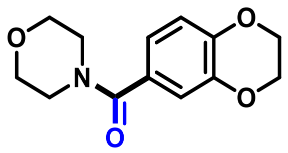

3bc (2,3-dihydrobenzo[*b*][1,4]dioxin-6-yl)(morpholino)methanone (*70*): The title compound was prepared from 6-iodo-2,3-dihydrobenzo[*b*][1,4]dioxine (262 mg, 1.0 mmol) and morpholine (130.5 mg, 1.5 mmol) following the general procedure and purified by column chromatography using petroleum ether/diethyl ether (3:1) to afford the product as a white solid (244.0 mg, 98% yield); **^{1}H NMR** (400 MHz, $CDCl_3$) δ 6.84 (d, *J* = 1.9 Hz, 1H), 6.79 – 6.74 (m, 2H), 4.16-4.11 (m, 4H), 3.56-3.48 (m, 8H); **^{13}C NMR** (100 MHz, $CDCl_3$) δ 169.4, 144.7, 143.0, 127.9, 120.3, 116.9, 116.4, 64.4, 64.0, 63.9, 47.8, 43.6.

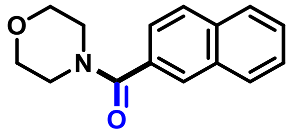

3bd morpholino(naphthalen-1-yl)methanone (*55*): The title compound was prepared from 2-iodonaphthalene (254 mg, 1.0 mmol) and morpholine (130.5 mg, 1.5 mmol) following the general procedure and purified by column chromatography using petroleum ether/diethyl ether (1:1) to afford the product as a white solid (231.4 mg, 96% yield); **^{1}H NMR** (400 MHz, $CDCl_3$) δ 7.82-7.78 (m, 3H), 7.49-7.34 (m, 4H), 3.96-3.75 (m, 4H), 3.43-3.36 (m, 2H), 3.12-3.08 (m, 2H); **^{13}C NMR** (100 MHz, $CDCl_3$) δ 169.0, 133.3, 133.1, 129.2, 128.9, 128.1, 126.7, 126.1, 124.8, 124.2, 123.5, 66.6, 66.5, 47.2, 41.8.

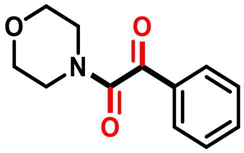

4a 1-morpholino-2-phenylethane-1,2-dione (*70*): The title compound was prepared from iodobenzene (204 mg, 1.0 mmol) and morpholine (130.5 mg, 1.5 mmol) following the general procedure and purified by column chromatography using petroleum ether/diethyl ether (5:1) to afford the product as a white solid (186.2 mg, 85% yield); **^{1}H NMR** (400 MHz, $CDCl_3$) δ 7.95 (d, J = 7.3 Hz, 2H), 7.65 (t, J = 7.4 Hz, 1H), 7.51 (t, J = 7.6 Hz, 2H), 3.78 (s, 4H), 3.64 (t, J = 4.8 Hz, 2H), 3.37 (t, J = 4.7 Hz, 2H); **^{13}C NMR** (100 MHz, $CDCl_3$) δ 191.1, 165.5, 134.9, 133.0, 129.6, 129.1, 66.7, 66.6, 46.2, 41.6.

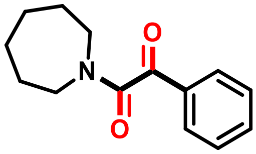

4b 1-(azepan-1-yl)-2-phenylethane-1,2-dione (*52*): The title compound was prepared from iodobenzene (204 mg, 1.0 mmol) and azepane (148.5 mg, 1.5 mmol) following the general procedure and purified by column chromatography using petroleum ether/diethyl ether (2:1) to afford the product as a yellow oil (184.8 mg, 80% yield); **^{1}H NMR** (400 MHz, $CDCl_3$) δ 7.88 (dd, J = 7.1, 2.0 Hz, 2H), 7.57 (td, J = 7.2, 1.7 Hz, 1H), 7.44 (td, J = 7.8, 2.0 Hz, 2H), 3.61 (td, J = 6.0, 2.2 Hz, 2H), 3.27 (td, J = 6.0, 2.2 Hz, 2H), 1.86 – 1.71 (m, 2H), 1.68 – 1.41 (m, 6H); **^{13}C NMR** (100 MHz, $CDCl_3$) δ 191.7, 167.0, 134.4, 133.0, 129.4, 128.8, 47.7, 44.9, 28.8, 27.4, 27.0, 26.3.

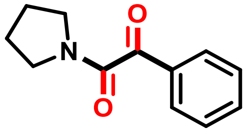

4c 1-phenyl-2-(pyrrolidin-1-yl)ethane-1,2-dione (*52*): The title compound was prepared from iodobenzene (204 mg, 1.0 mmol) and pyrrolidine (106.5 mg, 1.5 mmol) following the general procedure and purified by column chromatography using petroleum ether/diethyl ether (5:1) to afford the product as a white solid (176.6 mg, 87% yield); **^{1}H NMR** (400 MHz, $CDCl_3$) δ 7.92 – 7.89 (m, 2H), 7.57 – 7.53 (m, 1H), 7.42 (t, J = 7.7 Hz, 2H), 3.56 (t, J = 6.6 Hz, 2H), 3.33 (t, J = 6.5 Hz, 2H), 1.89 – 1.83 (m, 4H); **^{13}C NMR** (100 MHz, $CDCl_3$) δ 191.4, 164.5, 134.4, 132.6, 129.6, 128.7, 46.4, 45.0, 25.6, 23.8.

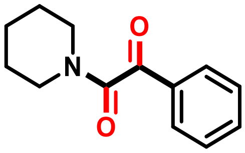

4d 1-phenyl-2-(piperidin-1-yl)ethane-1,2-dione (*17*): The title compound was prepared from iodobenzene (204 mg, 1.0 mmol) and piperidine (126.0 mg, 1.5 mmol) following the general procedure and purified by column chromatography using petroleum ether/diethyl ether (5:1) to afford the product as a white solid (204.1 mg, 94% yield); **^{1}H NMR** (400 MHz, $CDCl_3$) δ 7.88 – 7.86 (m, 2H), 7.56 (t, J = 7.4 Hz, 1H), 7.43 (t, J = 7.7 Hz, 2H), 3.62 (q, J = 3.8, 2.3 Hz, 2H), 3.22 – 3.19 (m, 2H), 1.64-1.57 (m, 4H), 1.47-1.44 (m, 2H); **^{13}C NMR** (100 MHz, $CDCl_3$) δ 191.7, 165.2, 134.4, 133.0, 129.2, 128.8, 46.7, 41.8, 25.9, 25.2, 24.1.

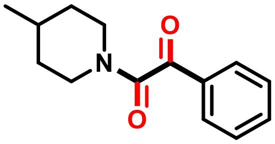

4e 1-(4-methylpiperidin-1-yl)-2-phenylethane-1,2-dione (*74*): The title compound was prepared from iodobenzene (204 mg, 1.0 mmol) and 4-methylpiperidine (148.5 mg, 1.5 mmol) following the general procedure and purified by column chromatography using petroleum ether/diethyl ether (2:1) to afford the product as a white solid (205.6 mg, 89% yield); **^{1}H NMR** (400 MHz, $CDCl_3$) δ 7.93 – 7.86 (m, 2H), 7.59 (td, J = 7.3, 1.3 Hz, 1H), 7.46 (t, J = 7.8 Hz, 2H), 4.57 (ddt, J = 13.3, 4.6, 2.3 Hz, 1H), 3.47 (ddt, J = 13.6, 4.4, 2.2 Hz, 1H), 3.08 – 2.95 (m, 1H), 2.75 (td, J = 12.8, 3.1 Hz, 1H), 1.74 (dt, J = 13.6, 2.8 Hz, 1H), 1.59 (ddt, J = 23.5, 14.5, 3.0 Hz, 2H), 1.29 – 0.97 (m, 2H); **^{13}C NMR** (100 MHz, $CDCl_3$) δ 191.8, 165.2, 134.5, 133.0, 129.4, 128.8, 46.1, 41.3, 34.1, 33.4, 30.1, 21.5.

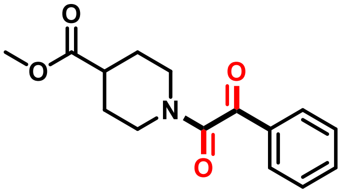

4f methyl 1-(2-oxo-2-phenylacetyl)piperidine-4-carboxylate (*75*): The title compound was prepared from iodobenzene (204 mg, 1.0 mmol) and methyl piperidine-4-carboxylate (214.5 mg, 1.5 mmol) following the general procedure and purified by column chromatography using petroleum ether/diethyl ether (2:1) to afford the product as a white solid (242.1 mg, 88% yield); **^{1}H NMR** (400 MHz, $CDCl_3$) δ 7.91 – 7.81 (m, 2H), 7.62 – 7.54 (m, 1H), 7.49 – 7.42 (m, 2H), 4.37 (dtd, *J* = 13.4, 4.3, 1.4 Hz, 1H), 3.62 (s, 3H), 3.49 (dtd, *J* = 13.7, 4.2, 1.4 Hz, 1H), 3.18 – 2.94 (m, 2H), 2.56 (tt, *J* = 10.5, 4.0 Hz, 1H), 2.00 (ddt, *J* = 11.8, 5.5, 4.1 Hz, 1H), 1.87 – 1.78 (m, 1H), 1.67 (ddtd, *J* = 44.2, 13.5, 10.8, 4.2 Hz, 2H); **^{13}C NMR** (100 MHz, $CDCl_3$) δ 191.4, 173.9, 165.2, 134.6, 132.8, 129.3, 128.9, 51.7, 45.0, 40.3, 40.2, 28.0, 27.4.

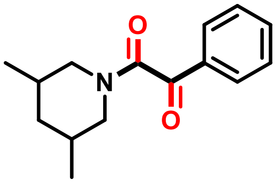

4g 1-(3,5-dimethylpiperidin-1-yl)-2-phenylethane-1,2-dione (*76*): The title compound was prepared from iodobenzene (204 mg, 1.0 mmol) and 3,5-dimethylpiperidine (169.5 mg, 1.5 mmol) following the general procedure and purified by column chromatography using petroleum ether/diethyl ether (2:1) to afford the product as a white solid (220.5 mg, 90% yield); **^{1}H NMR** (400 MHz, $CDCl_3$) δ 7.97 – 7.89 (m, 2H), 7.68 – 7.58 (m, 1H), 7.55 – 7.45 (m, 2H), 4.62 (ddt, *J* = 12.9, 4.1, 1.9 Hz, 1H), 3.41 (ddt, *J* = 13.3, 3.9, 1.8 Hz, 1H), 2.56 (dd, *J* = 13.3, 11.6 Hz, 1H), 2.23 (dd, *J* = 12.9, 11.7 Hz, 1H), 1.86 (dtt, *J* = 13.0, 3.5, 1.7 Hz, 1H), 1.79 – 1.53 (m, 2H), 0.96 (d, *J* = 6.6 Hz, 3H), 0.88 – 0.80 (m, 1H), 0.78 (d, *J* = 6.6 Hz, 3H) ; **^{13}C NMR** (100 MHz, $CDCl_3$) δ 191.8, 165.2, 134.6, 133.2, 129.5, 128.9, 52.9, 48.0, 42.2, 31.9, 31.1, 19.0, 18.7.

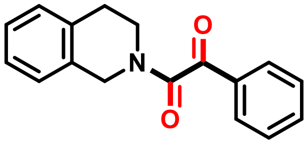

4h 1-(3,4-dihydroisoquinolin-2(1*H*)-yl)-2-phenylethane-1,2-dione (*75*): The title compound was prepared from iodobenzene (204 mg, 1.0 mmol) and 1,2,3,4-tetrahydroisoquinoline (199.5 mg, 1.5 mmol) following the general procedure and purified by column chromatography using petroleum ether/diethyl ether (2:1) to afford the product as a white solid (233.2 mg, 88% yield); **^{1}H NMR** (400 MHz, $CDCl_3$) δ 7.96 (ddt, *J* = 11.3, 6.9, 1.4 Hz, 2H), 7.67 – 7.55 (m, 1H), 7.47 (dt, *J* = 13.0, 7.8 Hz, 2H), 7.26 – 7.03 (m, 4H), 4.88 (s, 1H), 4.52 (s, 1H), 3.96 (t, *J* = 6.1 Hz, 1H), 3.58 (t, *J* = 5.9 Hz, 1H), 2.97 (t, *J* = 6.1 Hz, 1H), 2.82 (t, *J* = 5.9 Hz, 1H); **^{13}C NMR** (100 MHz, $CDCl_3$) δ 191.3, 165.8, 134.6, 133.9, 133.1, 132.8, 131.5, 129.4, 128.8, 126.9, 126.6, 126.3, 125.8, 47.0, 43.2, 43.1, 39.1, 28.9, 27.9.

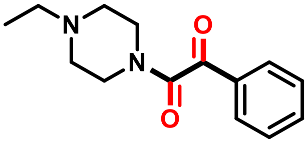

4i 1-(4-ethylpiperazin-1-yl)-2-phenylethane-1,2-dione (*76*): The title compound was prepared from iodobenzene (204 mg, 1.0 mmol) and 1-ethylpiperazine (171.0 mg, 1.5 mmol) following the general procedure and purified by column chromatography using petroleum ether/diethyl ether (2:1) to afford the product as a white solid (233.7 mg, 95% yield); **^{1}H NMR** (400 MHz, $CDCl_3$) δ 7.84 – 7.78 (m, 2H), 7.56 – 7.45 (m, 1H), 7.38 (dd, *J* = 8.4, 7.1 Hz, 2H), 3.75 – 3.58 (m, 2H), 3.24 (dd, *J* = 5.8, 4.3 Hz, 2H), 2.41 (t, *J* = 5.2 Hz, 2H), 2.34 – 2.19 (m, 4H), 0.94 (t, *J* = 7.2 Hz, 3H); **^{13}C NMR** (100 MHz, $CDCl_3$) δ 191.3, 165.1, 134.6, 132.9, 129.4, 128.9, 52.6, 52.0, 51.9, 45.7, 41.1, 11.7.

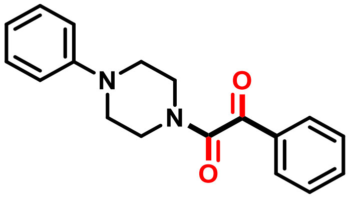

4j 1-phenyl-2-(4-phenylpiperazin-1-yl)ethane-1,2-dione (*75*): The title compound was prepared from iodobenzene (204 mg, 1.0 mmol) and 1-phenylpiperazine (243.0 mg, 1.5 mmol) following the general procedure and purified by column chromatography using petroleum ether/diethyl ether (2:1) to afford the product as a white solid (252.8 mg, 86% yield); ^{1}**H NMR** (400 MHz, $CDCl_3$) δ 8.05 – 7.97 (m, 2H), 7.66 (td, *J* = 7.3, 1.8 Hz, 1H), 7.53 (td, *J* = 7.8, 1.9 Hz, 2H), 7.29 (td, *J* = 7.7, 7.0, 1.9 Hz, 2H), 6.93 (dd, *J* = 8.6, 3.2 Hz, 3H), 3.93 (dt, *J* = 6.5, 3.3 Hz, 2H), 3.51 (dd, *J* = 6.6, 3.6 Hz, 2H), 3.28 (dd, *J* = 6.6, 3.7 Hz, 2H), 3.13 (q, *J* = 4.5 Hz, 2H); ^{13}**C NMR** (100 MHz, $CDCl_3$) δ 191.1, 165.1, 150.4, 134.7, 132.8, 129.4, 129.0, 128.9, 120.6, 116.6, 49.5, 49.2, 49.2, 45.5, 40.9.

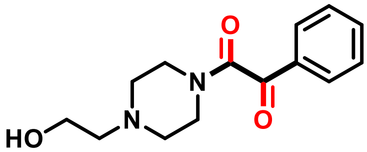

4k 1-(4-(2-hydroxyethyl)piperazin-1-yl)-2-phenylethane-1,2-dione: The title compound was prepared from iodobenzene (204 mg, 1.0 mmol) and 2-(piperazin-1-yl)ethan-1-ol (195.0 mg, 1.5 mmol) following the general procedure and purified by column chromatography using petroleum ether/diethyl ether (2:1) to afford the product as a white solid (233.2 mg, 89% yield); ^{1}**H NMR** (400 MHz, $CDCl_3$) δ 7.91 - 7.84 (m, 2H), 7.64 - 7.55 (m, 1H), 7.46 (t, *J* = 7.7 Hz, 2H), 3.72 (dd, *J* = 6.2, 4.1 Hz, 2H), 3.57 (t, *J* = 5.4 Hz, 2H), 3.31 (dd, *J* = 5.8, 4.3 Hz, 2H), 2.53 (dt, *J* = 18.6, 5.3 Hz, 4H), 2.42 (t, *J* = 7.7 Hz,, 2H); ^{13}**C NMR** (100 MHz, $CDCl_3$) δ 191.3, 165.2, 134.8, 132.8, 129.4, 128.9, 59.3, 57.8, 52.8, 52.2, 45.6, 41.0. HRMS (ESI) $[M+H]^+$: calculated for $[C_{14}H_{19}N_2O_3]^+$: 263.1396, found: 263.1395.

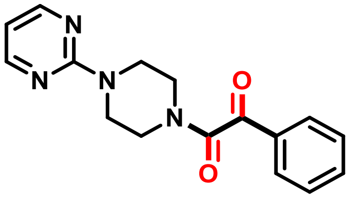

4l 1-phenyl-2-(4-(pyrimidin-2-yl)piperazin-1-yl)ethane-1,2-dione (*75*): The title compound was prepared from iodobenzene (204 mg, 1.0 mmol) and 2-(piperazin-1-yl)pyrimidine (246.0 mg, 1.5 mmol) following the general procedure and purified by column chromatography using petroleum ether/diethyl ether (2:1) to afford the product as a white solid (260.5 mg, 88% yield); ^{1}**H NMR** (400 MHz, $CDCl_3$) δ 8.23 (d, *J* = 4.8 Hz, 2H), 7.95 – 7.85 (m, 2H), 7.63 – 7.53 (m, 1H), 7.43 (t, *J* = 7.7 Hz, 2H), 6.46 (t, *J* = 4.8 Hz, 1H), 3.89 (dd, *J* = 6.5, 4.0 Hz, 2H), 3.75 (dd, *J* = 6.3, 3.9 Hz, 4H), 3.40 – 3.29 (m, 2H); ^{13}**C NMR** (100 MHz, $CDCl_3$) δ 191.0, 165.4, 161.1, 157.5, 134.7, 132.8, 129.4, 128.8, 77.3, 77.0, 76.7, 45.4, 43.6, 43.1, 40.9.

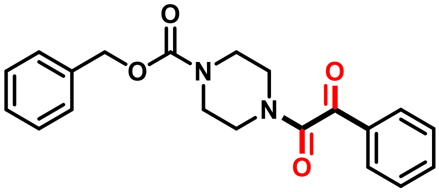

4m benzyl 4-(2-oxo-2-phenylacetyl)piperazine-1-carboxylate (*77*): The title compound was prepared from iodobenzene (204 mg, 1.0 mmol) and benzyl piperazine-1-carboxylate (328.5 mg, 1.5 mmol) following the general procedure and purified by column chromatography using petroleum ether/diethyl ether (2:1) to afford the product as a white solid (323.8 mg, 92% yield); ^{1}**H NMR** (400 MHz, $CDCl_3$) δ 7.97 – 7.87 (m, 2H), 7.67 – 7.55 (m, 1H), 7.47 (t, *J* = 7.8 Hz, 2H), 7.59 – 7.45 (m, 5H), 5.12 (s, 2H), 3.72 (t, *J* = 5.0 Hz, 2H), 3.64 – 3.54 (m, 2H), 3.51 – 3.43 (m, 2H), 3.30 (t, *J* = 5.2 Hz, 2H); ^{13}**C NMR** (100 MHz, $CDCl_3$) δ 190.8, 165.3, 154.7, 135.9, 134.7, 132.7, 129.4, 128.8, 128.3, 128.0, 127.7, 127.7, 67.3, 45.4, 43.7, 43.2, 40.8.

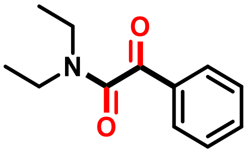

4n ***N,N*-diethyl-2-oxo-2-phenylacetamide (*17*):** The title compound was prepared from iodobenzene (204 mg, 1.0 mmol) and dimethylamine (109.5 mg, 1.5 mmol) following the general procedure and purified by column chromatography using petroleum ether/diethyl ether (6:1) to afford the product as a yellow oil (180.5 mg, 88% yield); **^{1}H NMR** (400 MHz, $CDCl_3$) δ 7.92 – 7.89 (m, 2H), 7.62 – 7.58 (m, 1H), 7.50 – 7.45 (m, 2H), 3.53 (q, *J* = 7.2 Hz, 2H), 3.21 (q, *J* = 7.1 Hz, 2H), 1.25 (t, *J* = 7.2 Hz, 3H), 1.12 (t, *J* = 7.1 Hz, 3H); **^{13}C NMR** (100 MHz, $CDCl_3$) δ 191.5, 166.6, 134.5, 133.1, 129.5, 128.8, 42.0, 38.7, 14.0, 12.7.

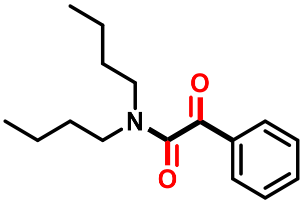

4o ***N,N*-dibutyl-2-oxo-2-phenylacetamide (*74*):** The title compound was prepared from iodobenzene (204 mg, 1.0 mmol) and dibutylamine (109.5 mg, 1.5 mmol) following the general procedure and purified by column chromatography using petroleum ether/diethyl ether (8:1) to afford the product as a white solid (234.9 mg, 90% yield); **^{1}H NMR** (400 MHz, $CDCl_3$) δ 7.89 – 7.87 (m, 2H), 7.60 – 7.55 (m, 1H), 7.47 – 7.42 (m, 2H), 3.47 – 3.43 (m, 2H), 3.12 – 3.08 (m, 2H), 1.68 – 1.57 (m, 2H), 1.48 (tt, *J* = 7.8, 6.6 Hz, 2H), 1.36 (dq, *J* = 14.8, 7.4 Hz, 2H), 1.13 (h, *J* = 7.4 Hz, 2H), 0.94 (t, *J* = 7.4 Hz, 3H), 0.75 (t, *J* = 7.4 Hz, 3H); **^{13}C NMR** (100 MHz, $CDCl_3$) δ 191.4, 166.9, 134.3, 133.2, 129.4, 128.8, 47.2, 43.8, 30.4, 29.3, 20.1, 19.6, 13.7, 13.4.

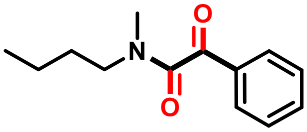

4p ***N*-butyl-*N*-methyl-2-oxo-2-phenylacetamide (*74*):** The title compound was prepared from iodobenzene (204 mg, 1.0 mmol) and *N*-methylbutan-1-amine (130.5 mg, 1.5 mmol) following the general procedure and purified by column chromatography using petroleum ether/diethyl ether (6:1) to afford the product as a white solid (181.8 mg, 83% yield); **^{1}H NMR** (400 MHz, $CDCl_3$) δ 7.97 – 7.90 (m, 2H), 7.63 (tdd, *J* = 6.9, 2.9, 1.4 Hz, 1H), 7.50 (tdd, *J* = 8.0, 2.7, 1.1 Hz, 2H), 3.57 – 3.49 (m, 1H), 3.21 – 3.12 (m, 1H), 3.00 (d, *J* = 67.5 Hz, 3H), 1.70 – 1.49 (m, 2H), 1.45 – 1.36 (m, 1H), 1.27 – 1.14 (m, 1H), 0.90 (dt, *J* = 69.0, 7.4 Hz, 3H); **^{13}C NMR** (100 MHz, $CDCl_3$) δ 191.6, 167.1, 134.6, 133.2, 129.6, 138.9, 49.7, 31.6, 30.1, 19.6, 13.5.

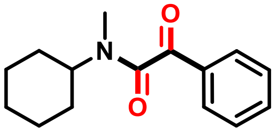

4q ***N*-cyclohexyl-*N*-methyl-2-oxo-2-phenylacetamide (*74*):** The title compound was prepared from iodobenzene (204 mg, 1.0 mmol) and *N*-methylcyclohexanamine (169.5 mg, 1.5 mmol) following the general procedure and purified by column chromatography using petroleum ether/diethyl ether (6:1) to afford the product as a white solid (196.0 mg, 80% yield); **^{1}H NMR** (400 MHz, $CDCl_3$) δ 7.81 (ddt, *J* = 9.2, 3.4, 1.5 Hz, 2H), 7.56 – 7.46 (m, 1H), 7.39 (dt, *J* = 7.9, 3.8 Hz, 2H), 4.47 – 3.06 (m, 1H), 2.95 – 2.58 (m, 3H), 1.90 – 1.55 (m, 4H), 1.50 – 1.32 (m, 4H), 0.97 (tdd, *J* = 22.4, 12.7, 9.6 Hz, 2H); **^{13}C NMR** (100 MHz, $CDCl_3$) δ 191.6, 166.9, 134.4, 133.1, 129.3, 128.8, 128.8, 57.4, 52.0, 30.2, 29.6, 29.2, 26.0, 25.3, 25.1, 24.7.

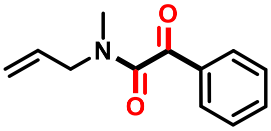

4r ***N*-allyl-*N*-methyl-2-oxo-2-phenylacetamide (*78*):** The title compound was prepared from iodobenzene (204 mg, 1.0 mmol) and *N*-methylprop-2-en-1-amine (106.5 mg, 1.5 mmol) following the general procedure and purified by column chromatography using petroleum ether/diethyl ether (6:1) to afford the product as a white solid (150.2 mg, 74% yield); **^{1}H NMR** (400 MHz, $CDCl_3$) δ 7.93 (dq, *J* = 8.4, 1.7 Hz, 2H), 7.66 – 7.57 (m, 1H), 7.49 (ddd, *J* = 8.8, 7.2, 5.2 Hz, 2H), 5.91 – 5.62 (m, 1H), 5.34 – 5.13 (m, 2H), 4.14 (dt, *J* = 6.0, 1.5 Hz, 1H), 3.79 (dt, *J* = 6.0, 1.4 Hz, 1H), 2.96 (d, *J* = 66.0 Hz, 3H); **^{13}C NMR** (100 MHz, $CDCl_3$) δ 191.5, 167.1, 134.7, 133.0, 131.8, 131.4, 129.6, 128.9, 119.2, 52.3, 34.4.

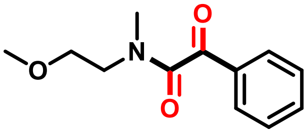

4s *N*-(2-methoxyethyl)-*N*-methyl-2-oxo-2-phenylacetamide: The title compound was prepared from iodobenzene (204 mg, 1.0 mmol) and 2-methoxy-*N*-methylethan-1-amine (133.5 mg, 1.5 mmol) following the general procedure and purified by column chromatography using petroleum ether/diethyl ether (2:1) to afford the product as a white solid (183.4 mg, 83% yield); **^{1}H NMR** (400 MHz, $CDCl_3$) δ 7.91 (dq, *J* = 8.5, 1.5 Hz, 2H), 7.59 (dtd, *J* = 11.5, 7.4, 1.5 Hz, 1H), 7.46 (dtd, *J* = 11.3, 7.8, 1.5 Hz, 2H), 3.72 – 3.60 (m, 2H), 3.39 (dd, *J* = 18.0, 1.5 Hz, 3H), 3.13 – 3.08 (m, 3H), 2.96 (d, *J* = 1.6 Hz, 2H); **^{13}C NMR** (100 MHz, $CDCl_3$) δ 191.6, 167.4, 134.6, 133.2, 129.8, 128.9, 70.0, 58.7, 49.2, 46.2, 36.2, 32.8. HRMS (ESI) $[M+Na]^+$: calculated for $[C_{12}H_{15}NNaO_3]^+$: 244.0950, found: 244.0939.

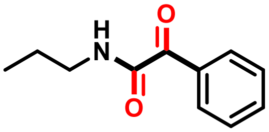

4t 2-oxo-2-phenyl-*N*-propylacetamide (*15*): The title compound was prepared from iodobenzene (204 mg, 1.0 mmol) and propan-1-amine (88.5 mg, 1.5 mmol) following the general procedure and purified by column chromatography using petroleum ether/diethyl ether (15:1) to afford the product as a white solid (171.9 mg, 90% yield); **^{1}H NMR** (400 MHz, $CDCl_3$) δ 8.33 – 8.31 (m, 2H), 7.63 – 7.59 (m, 1H), 7.46 (t, *J* = 7.6 Hz, 2H), 7.14 (brs, 1H), 3.35 (q, *J* = 6.8 Hz, 2H), 1.63 (h, *J* = 7.3 Hz, 2H), 0.97 (t, *J* = 7.4 Hz, 3H); **^{13}C NMR** (100 MHz, $CDCl_3$) δ 187.9, 161.8, 134.3, 133.3, 131.1, 128.4, 41.1, 22.5, 11.3.

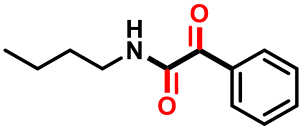

4u *N*-butyl-2-oxo-2-phenylacetamide (*52*): The title compound was prepared from iodobenzene (204 mg, 1.0 mmol) and butan-1-amine (109.5 mg, 1.5 mmol) following the general procedure and purified by column chromatography using petroleum ether/diethyl ether (15:1) to afford the product as a white solid (186.6 mg, 91% yield); **^{1}H NMR** (400 MHz, $CDCl_3$) δ 8.30 – 8.28 (m, 2H), 7.59 – 7.55 (m, 1H), 7.43 (t, *J* = 7.8 Hz, 2H), 7.20 (brs, 1H), 3.36 (q, *J* = 7.0 Hz, 2H), 1.55 (p, *J* = 7.2 Hz, 2H), 1.36 (h, *J* = 7.3 Hz, 2H), 0.92 (t, *J* = 7.3 Hz, 3H); **^{13}C NMR** (100 MHz, $CDCl_3$) δ 187.9, 161.8, 134.2, 133.3, 131.0, 128.3, 39.0, 31.2, 19.9, 13.6.

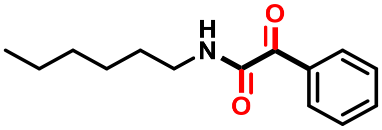

4v *N*-hexyl-2-oxo-2-phenylacetamide (*79*): The title compound was prepared from iodobenzene (204 mg, 1.0 mmol) and hexan-1-amine (151.5 mg, 1.5 mmol) following the general procedure and purified by column chromatography using petroleum ether/diethyl ether (15:1) to afford the product as a white solid (207.4 mg, 89% yield); **^{1}H NMR** (400 MHz, $CDCl_3$) δ 8.31 (dd, *J* = 8.2, 1.4 Hz, 2H), 7.60 (t, *J* = 7.4 Hz, 1H), 7.45 (t, *J* = 7.8 Hz, 2H), 7.26 (brs, 1H), 3.37 (q, *J* = 7.0 Hz, 2H), 1.58 (h, *J* = 6.8 Hz, 2H), 1.42 – 1.23 (m, 6H), 0.89 (t, *J* = 7.3 Hz, 3H); **^{13}C NMR** (100 MHz, $CDCl_3$) δ 187.9, 161.8, 134.1, 133.3, 131.0, 128.3, 39.3, 31.3, 29.1, 26.4, 22.4, 13.6.

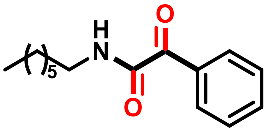

4w *N*-heptyl-2-oxo-2-phenylacetamide: The title compound was prepared from iodobenzene (204 mg, 1.0 mmol) and heptanamine (172.5 mg, 1.5 mmol) following the general procedure and purified by column chromatography using petroleum ether/diethyl ether (15:1) to afford the product as a white solid (212.4 mg, 86% yield); **^{1}H NMR** (400 MHz, $CDCl_3$) δ 8.34 – 8.32 (m, 2H), 7.63 – 7.59 (m, 1H), 7.47 (t, *J* = 7.8 Hz, 2H), 7.12 (brs, 1H), 3.38 (td, *J* = 7.3, 6.2 Hz, 2H), 1.66 – 1.56 (m, 2H), 1.35 – 1.27 (m, 8H), 0.87 (t, *J* = 6.8 Hz, 3H); **^{13}C NMR** (100 MHz, $CDCl_3$) δ 187.9, 161.7, 134.3, 133.4, 131.2, 128.4, 39.4, 31.7, 29.3, 28.7, 26.8, 22.5, 14.0. HRMS (ESI) $[M+Na]^+$: calculated for $[C_{15}H_{21}NNaO_2]^+$: 270.1470, found: 270.1463.

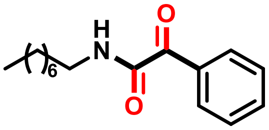

4x ***N*-octyl-2-oxo-2-phenylacetamide (*80*):** The title compound was prepared from iodobenzene (204 mg, 1.0 mmol) and octylamine (193.5 mg, 1.5 mmol) following the general procedure and purified by column chromatography using petroleum ether/diethyl ether (15:1) to afford the product as a white solid (232.3 mg, 89% yield); **^{1}H NMR** (400 MHz, $CDCl_3$) δ 8.31 – 8.29 (m, 2H), 7.58 (t, *J* = 7.4 Hz, 1H), 7.44 (t, *J* = 7.8 Hz, 2H), 7.19 (brs, 1H), 3.35 (q, *J* = 6.8 Hz, 2H), 1.57 (p, *J* = 7.5 Hz, 2H), 1.36 – 1.24 (m, 10H), 0.86 (t, *J* = 6.8 Hz, 3H); **^{13}C NMR** (100 MHz, $CDCl_3$) δ 187.9, 161.7, 134.2, 133.3, 131.1, 128.3, 39.4, 31.7, 29.2, 29.1, 29.1, 26.8, 22.5, 14.0.

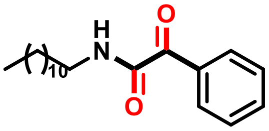

4y ***N*-dodecyl-2-oxo-2-phenylacetamide (*81*):** The title compound was prepared from iodobenzene (204 mg, 1.0 mmol) and dodecylamine (277.5 mg, 1.5 mmol) following the general procedure and purified by column chromatography using petroleum ether/diethyl ether (15:1) to afford the product as a white solid (317.6 mg, 80% yield); **^{1}H NMR** (400 MHz, $CDCl_3$) δ 8.34 – 8.32 (m, 2H), 7.61 (t, *J* = 7.4 Hz, 1H), 7.47 (t, *J* = 7.7 Hz, 2H), 7.12 (brs, 1H), 3.38 (q, *J* = 6.9 Hz, 2H), 1.60 (p, *J* = 7.2 Hz, 2H), 1.38 – 1.25 (m, 18H), 0.87 (t, *J* = 6.7 Hz, 3H); **^{13}C NMR** (100 MHz, $CDCl_3$) δ δ 187.9, 161.7, 134.3, 133.4, 131.2, 128.4, 39.4, 31.9, 29.6, 29.5, 29.5, 29.3, 29.3, 29.2, 26.8, 22.7, 14.1.

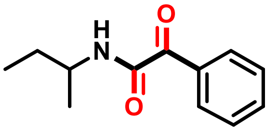

4z ***N*-(sec-butyl)-2-oxo-2-phenylacetamide (*79*):** The title compound was prepared from iodobenzene (204 mg, 1.0 mmol) and butan-2-amine (109.5 mg, 1.5 mmol) following the general procedure and purified by column chromatography using petroleum ether/diethyl ether (15:1) to afford the product as a white solid (164.0 mg, 80% yield); **^{1}H NMR** (400 MHz, $CDCl_3$) δ 8.33 – 8.31 (m, 2H), 7.61 (t, *J* = 7.4 Hz, 1H), 7.46 (t, *J* = 7.8 Hz, 2H), 7.18 (brs, 1H), 3.22 (t, *J* = 6.6 Hz, 2H), 1.88 (dp, *J* = 13.5, 6.7 Hz, 1H), 0.96 (d, *J* = 6.7 Hz, 6H); **^{13}C NMR** (100 MHz, $CDCl_3$) δ 187.9, 161.8, 134.3, 133.3, 131.1, 128.4, 46.6, 28.5, 20.0.

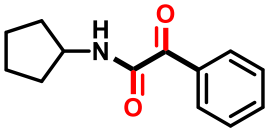

4aa ***N*-cyclopentyl-2-oxo-2-phenylacetamide (*80*):** The title compound was prepared from iodobenzene (204 mg, 1.0 mmol) and cyclopentanamine (127.5 mg, 1.5 mmol) following the general procedure and purified by column chromatography using petroleum ether/diethyl ether (20:1) to afford the product as a white solid (175.8 mg, 81% yield); **^{1}H NMR** (400 MHz, $CDCl_3$) δ 8.29 – 8.27 (m, 2H), 7.57 (t, *J* = 7.4 Hz, 1H), 7.42 (t, *J* = 7.7 Hz, 2H), 7.14 (brs, 1H), 4.26 (h, *J* = 7.0 Hz, 1H), 2.02 (dq, *J* = 12.4, 6.5 Hz, 2H), 1.71 – 1.59 (m, 4H), 1.52 – 1.46 (m, 2H); **^{13}C NMR** (100 MHz, $CDCl_3$) δ 187.9, 161.3, 134.1, 133.3, 131.0, 128.3, 51.0, 32.7, 23.7.

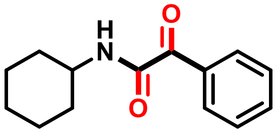

4ab ***N*-cyclohexyl-2-oxo-2-phenylacetamide (*75*):** The title compound was prepared from iodobenzene (204 mg, 1.0 mmol) and cyclohexanamine (148.5 mg, 1.5 mmol) following the general procedure and purified by column chromatography using petroleum ether/diethyl ether (2:1) to afford the product as a white solid (196.4 mg, 85% yield); **^{1}H NMR** (400 MHz, $CDCl_3$) δ 8.27 (dt, *J* = 8.5, 1.6 Hz, 2H), 7.56 (td, *J* = 7.3, 1.7 Hz, 1H), 7.42 (td, *J* = 7.9, 1.9 Hz, 2H), 7.06 (brs, 1H), 3.88 – 3.76 (m, 1H), 1.94 (dd, *J* = 12.8, 4.1 Hz, 2H), 1.72 (dt, *J* = 12.9, 4.1 Hz, 2H), 1.65 – 1.54 (m, 1H), 1.45 – 1.29 (m, 2H), 1.30 – 1.11 (m, 3H); **^{13}C NMR** (100 MHz, $CDCl_3$) δ 188.1, 160.9, 160.8, 134.1, 133.3, 131.0, 128.3, 48.3, 32.5, 25.3, 24.6.

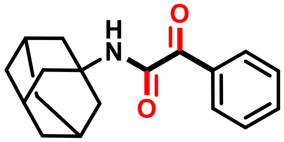

4ac N-(adamantan-1-yl)-2-oxo-2-phenylacetamide (*80*): The title compound was prepared from iodobenzene (204 mg, 1.0 mmol) and adamantan-1-amine (226.5 mg, 1.5 mmol) following the general procedure and purified by column chromatography using petroleum ether/diethyl ether (6:1) to afford the product as a white solid (271.7 mg, 96% yield); **^{1}H NMR** (400 MHz, $CDCl_3$) δ 8.33 – 8.22 (m, 2H), 7.63 – 7.54 (m, 1H), 7.45 (t, *J* = 7.8 Hz, 2H), 6.81 (brs, 1H), 2.20 – 1.99 (m, 9H), 1.70 (d, *J* = 2.9 Hz, 6H); **^{13}C NMR** (100 MHz, $CDCl_3$) δ 188.5, 160.8, 134.0, 133.4, 131.1, 128.3, 52.3, 41.0, 36.2, 29.3.

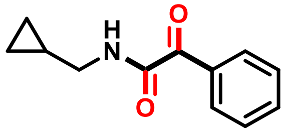

4ad *N*-(cyclopropylmethyl)-2-oxo-2-phenylacetamide (*81*): The title compound was prepared from iodobenzene (204 mg, 1.0 mmol) and cyclopropylmethanamine (106.5 mg, 1.5 mmol) following the general procedure and purified by column chromatography using petroleum ether/diethyl ether (25:1) to afford the product as a white solid (166.5 mg, 82% yield); **^{1}H NMR** (400 MHz, $CDCl_3$) δ 8.34 – 8.32 (m, 2H), 7.62 (t, *J* = 7.4 Hz, 1H), 7.48 (t, *J* = 7.8 Hz, 2H), 7.24 (brs, 1H), 3.27 – 3.24 (m, 2H), 1.09-0.99 (m, 1H), 0.58 – 0.56 (m, 2H), 0.30 – 0.28 (m, 2H); **^{13}C NMR** (100 MHz, $CDCl_3$) δ 187.9, 161.6, 134.3, 133.3, 131.1, 128.4, 44.2, 10.4, 3.5.

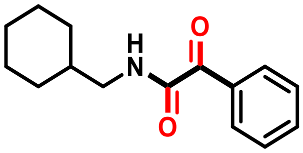

4ae *N*-(cyclohexylmethyl)-2-oxo-2-phenylacetamide (*82*): The title compound was prepared from iodobenzene (204 mg, 1.0 mmol) and cyclohexylmethanamine (169.5 mg, 1.5 mmol) following the general procedure and purified by column chromatography using petroleum ether/diethyl ether (20:1) to afford the product as a white solid (208.3 mg, 85% yield);**^{1}H NMR** (400 MHz, $CDCl_3$) δ 8.33 (dd, *J* = 8.2, 1.4 Hz, 2H), 7.61 (t, *J* = 7.4 Hz, 1H), 7.46 (t, *J* = 7.8 Hz, 2H), 7.23 (brs, 1H), 3.23 (t, *J* = 6.6 Hz, 2H), 1.78 – 1.65 (m, 5H), 1.57 (th, *J* = 10.8, 3.3 Hz, 1H), 1.29 – 1.13 (m, 3H), 0.98 (qd, *J* = 13.1, 12.4, 3.8 Hz, 2H); **^{13}C NMR** (100 MHz, $CDCl_3$) δ 187.9, 161.8, 134.2, 133.3, 131.1, 128.3, 45.5, 37.8, 30.7, 26.2, 25.7.

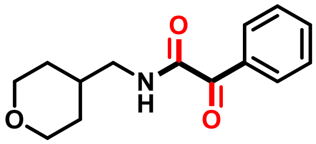

4af 2-oxo-2-phenyl-*N*-((tetrahydro-2*H*-pyran-4-yl)methyl)acetamide (*83*): The title compound was prepared from iodobenzene (204 mg, 1.0 mmol) and (tetrahydro-2H-pyran-4-yl)methanamine (172.5 mg, 1.5 mmol) following the general procedure and purified by column chromatography using petroleum ether/diethyl ether (2:1) to afford the product as a white solid (202.5 mg, 82% yield); **^{1}H NMR** (400 MHz, $CDCl_3$) δ 8.27 (dt, *J* = 8.6, 1.6 Hz, 2H), 7.59 (td, *J* = 7.4, 1.4 Hz, 1H), 7.44 (td, *J* = 7.7, 1.3 Hz, 2H), 7.33 (brs, 1H), 3.99 – 3.90 (m, 2H), 3.33 (tt, *J* = 11.8, 1.8 Hz, 2H), 3.25 (td, *J* = 6.7, 1.4 Hz, 2H), 1.81 (ddq, *J* = 14.8, 7.8, 4.0 Hz, 1H), 1.61 (ddt, *J* = 13.0, 3.8, 1.8 Hz, 2H), 1.40 – 1.26 (m, 2H); **^{13}C NMR** (100 MHz, $CDCl_3$) δ 187.7, 162.0, 162.0, 134.3, 133.1, 131.0, 128.4, 67.4, 44.9, 35.1, 30.4.

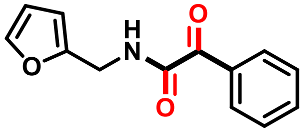

4ag *N*-(furan-2-ylmethyl)-2-oxo-2-phenylacetamide (*81*): The title compound was prepared from iodobenzene (204 mg, 1.0 mmol) and furan-2-ylmethanamine (145.5 mg, 1.5 mmol) following the general procedure and purified by column chromatography using petroleum ether/diethyl ether (20:1) to afford the product as a white solid (199.3 mg, 87% yield); **^{1}H NMR** (400 MHz, $CDCl_3$) δ 8.33 (dt, *J* = 8.5, 1.2 Hz, 2H), 7.61 (t, *J* = 7.4 Hz, 1H), 7.46 (t, *J* = 7.9 Hz, 2H), 7.36 (brs, 1H), 6.31 (dd, *J* = 13.1, 2.6 Hz, 2H), 4.55 (d, *J* = 5.9 Hz, 2H); **^{13}C NMR** (100 MHz, $CDCl_3$) δ 187.2, 161.4, 150.1, 142.5, 134.4, 133.2, 131.1, 128.4, 110.4, 108.0, 36.3.

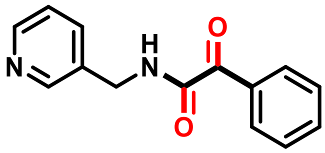

4ah 2-oxo-2-phenyl-*N*-(pyridin-3-ylmethyl)acetamide (*83*): The title compound was prepared from iodobenzene (204 mg, 1.0 mmol) and pyridin-3-ylmethanamine (162.0 mg, 1.5 mmol) following the general procedure and purified by column chromatography using petroleum ether/diethyl ether (20:1) to afford the product as a white solid (201.6 mg, 84% yield); ^{1}**H NMR** (400 MHz, $CDCl_3$) δ 8.58 (s, 1H), 8.53 (dd, J = 3.6 Hz, 1H), 8.33 – 8.31 (m, 2H), 7.88 (brs, 1H), 7.70 – 7.61 (m, 2H), 7.48 (t, J = 7.8 Hz, 2H), 7.29 – 7.26 (m, 1H), 4.59 (d, J = 6.2 Hz, 2H); ^{13}**C NMR** (100 MHz, $CDCl_3$) δ 187.3, 161.9, 149.2, 149.0, 135.6, 134.5, 133.1, 133.0, 131.1, 128.5, 123.6, 40.8.

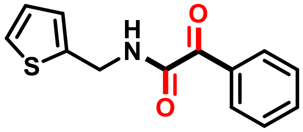

4ai 2-oxo-2-phenyl-*N*-(thiophen-2-ylmethyl)acetamide (*81*): The title compound was prepared from iodobenzene (204 mg, 1.0 mmol) and thiophen-2-ylmethanamine (169.5 mg, 1.5 mmol) following the general procedure and purified by column chromatography using petroleum ether/diethyl ether (20:1) to afford the product as a white solid (191.1 mg, 78% yield); ^{1}**H NMR** (400 MHz, $CDCl_3$) δ 8.32 – 8.30 (m, 2H), 7.65 (brs, 1H), 7.62 – 7.58 (m, 1H), 7.45 (dd, J = 8.4, 7.3 Hz, 2H), 7.22 (dd, J = 5.1, 1.3 Hz, 1H), 7.02 – 7.01 (m, 1H), 6.94 (dd, J = 5.1, 3.5 Hz, 2H), 4.71 (d, J = 6.0 Hz, 2H); ^{13}**C NMR** (100 MHz, $CDCl_3$) δ 187.3, 161.4, 139.4, 134.3, 133.0, 131.0, 128.3, 126.8, 126.4, 125.4, 37.9.

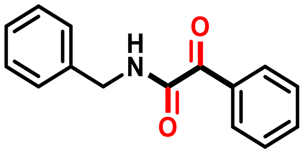

4aj *N*-benzyl-2-oxo-2-phenylacetamide (*15*): The title compound was prepared from iodobenzene (204 mg, 1.0 mmol) and phenylmethanamine (160.5 mg, 1.5 mmol) following the general procedure and purified by column chromatography using petroleum ether/diethyl ether (20:1) to afford the product as a white solid (193.6 mg, 81% yield); ^{1}**H NMR** (400 MHz, $CDCl_3$) δ 8.36-8.33 (m, 2H), 7.62 (t, J = 7.4 Hz, 1H), 7.47 (t, J = 7.8 Hz, 3H), 7.36 – 7.30 (m, 5H), 4.56 (d, J = 6.0 Hz, 2H); ^{13}**C NMR** (100 MHz, $CDCl_3$) δ 187.5, 161.5, 137.1, 134.4, 133.3, 131.2, 128.8, 128.5, 128.5, 128.2, 127.9, 127.8, 127.8, 43.4.

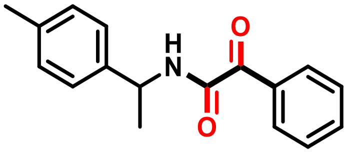

4ak 2-oxo-2-phenyl-*N*-(1-(p-tolyl)ethyl)acetamide (*83*): The title compound was prepared from iodobenzene (204 mg, 1.0 mmol) and 1-(p-tolyl)ethan-1-amine (202.5 mg, 1.5 mmol) following the general procedure and purified by column chromatography using petroleum ether/diethyl ether (20:1) to afford the product as a white solid (235.1 mg, 88% yield); ^{1}**H NMR** (400 MHz, $CDCl_3$) δ 8.32-8.30 (m, 2H), 7.58 (t, J = 7.4 Hz, 1H), 7.43 (t, J = 7.8 Hz, 3H), 7.25 (d, J = 8.2 Hz, 1H), 7.14 (d, J = 7.8 Hz, 2H), 5.15 (p, J = 7.1 Hz, 1H), 2.32 (s, 3H), 1.56 (d, J = 6.9 Hz, 3H); ^{13}**C NMR** (100 MHz, $CDCl_3$) δ 187.7, 160.7, 139.2, 137.2, 134.2, 133.3, 131.1, 129.3, 128.3, 126.0, 48.7, 21.6, 21.0.

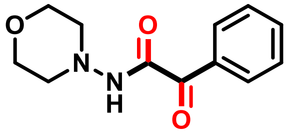

4al *N*-morpholino-2-oxo-2-phenylacetamide: The title compound was prepared from iodobenzene (204 mg, 1.0 mmol) and morpholin-4-amine (153.0 mg, 1.5 mmol) following the general procedure and purified by column chromatography using petroleum ether/diethyl ether (2:1) to afford the product as a white solid (208.3 mg, 89% yield); ^{1}**H NMR** (400 MHz, $CDCl_3$) δ7.91 – 7.87 (m, 2H), 7.61 – 7.57 (m, 1H), 7.47 – 7.43 (m, 2H), 3.74 – 3.70 (m, 4H), 3.58 – 3.56 (m, 2H), 3.32 – 3.29 (m, 2H); ^{13}**C NMR** (100 MHz, $CDCl_3$) δ 191.0, 165.2, 134.7, 132.7, 129.4, 128.9, 66.4, 66.4, 46.0, 41.3. HRMS (ESI) $[M+Na]^+$: calculated for $[C_{12}H_{14}N_2NaO_3]^+$: 257.0902, found: 257.0551.

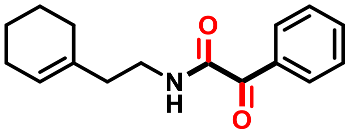

4am ***N*-(2-(cyclohex-1-en-1-yl)ethyl)-2-oxo-2-phenylacetamide (*75*):** The title compound was prepared from iodobenzene (204 mg, 1.0 mmol) and 2-(cyclohex-1-en-1-yl)ethan-1-amine (187.5 mg, 1.5 mmol) following the general procedure and purified by column chromatography using petroleum ether/diethyl ether (2:1) to afford the product as a white solid (205.6 mg, 80% yield); **^{1}H NMR** (400 MHz, $CDCl_3$) δ 8.36 - 8.25 (m, 2H), 7.65 - 7.55 (m, 1H), 7.46 (dd, *J* = 8.4, 7.2 Hz, 2H), 7.08 (s, 1H), 5.50 (tt, *J* = 3.6, 1.6 Hz, 1H), 3.45 (td, *J* = 6.8, 5.7 Hz, 2H), 2.26 - 2.16 (m, 2H), 2.04 - 1.90 (m, 4H), 1.68 - 1.49 (m, 4H); **^{13}C NMR** (100 MHz, $CDCl_3$) δ 187.9, 161.7, 134.2, 134.0, 133.3, 131.1, 128.4, 124.0, 37.3, 37.1, 27.8, 25.2, 22.7, 22.2.

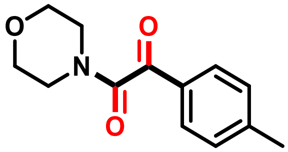

4an 1-morpholino-2-(p-tolyl)ethane-1,2-dione (*70*): The title compound was prepared from 1-iodo-4-methylbenzene (218 mg, 1.0 mmol) and morpholine (130.5 mg, 1.5 mmol) following the general procedure and purified by column chromatography using petroleum ether/diethyl ether (1:1) to afford the product as a white solid (207.4 mg, 89% yield); **^{1}H NMR** (400 MHz, $CDCl_3$) δ 7.85 (d, *J* = 8.2 Hz, 2H), 7.32 (d, *J* = 8.0 Hz, 2H), 3.78 (d, *J* = 1.9 Hz, 4H), 3.63 (dd, *J* = 5.5, 4.1 Hz, 2H), 3.36 (dd, *J* = 5.6, 4.1 Hz, 2H), 2.43 (s, 3H); **^{13}C NMR** (100 MHz, $CDCl_3$) δ 190.7, 165.4, 146.0, 130.4, 129.6, 129.5, 66.5, 66.4, 46.0, 41.3, 21.7.

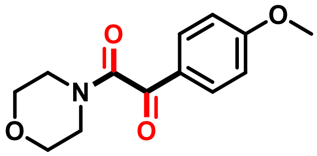

4ao 1-(4-methoxyphenyl)-2-morpholinoethane-1,2-dione (*70*): The title compound was prepared from 1-iodo-4-methoxybenzene (234 mg, 1.0 mmol) and morpholine (130.5 mg, 1.5 mmol) following the general procedure and purified by column chromatography using petroleum ether/diethyl ether (2:1) to afford the product as a white solid (194.2 mg, 78% yield); **^{1}H NMR** (400 MHz, $CDCl_3$) δ 7.87 (d, *J* = 8.4 Hz, 2H), 6.93 (d, *J* = 8.5 Hz, 2H), 3.83 (s, 3H), 3.72 (s, 2H), 3.59 (t, *J* = 4.7 Hz, 2H), 3.32 (t, *J* = 4.7 Hz, 2H); **^{13}C NMR** (100 MHz, $CDCl_3$) δ 189.7, 165.6, 164.8, 131.9, 125.9, 114.2, 66.6, 66.5, 55.5, 46.1, 41.4.

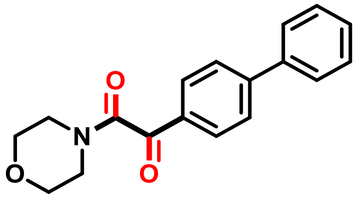

4ap 1-([1,1'-biphenyl]-4-yl)-2-morpholinoethane-1,2-dione (*70*): The title compound was prepared from 4-iodo-1,1'-biphenyl (281 mg, 1.0 mmol) and morpholine (130.5 mg, 1.5 mmol) following the general procedure and purified by column chromatography using petroleum ether/diethyl ether (2:1) to afford the product as a white solid (224.2 mg, 76% yield); **^{1}H NMR** (400 MHz, $CDCl_3$) δ 8.04 – 8.02 (m, 2H), 7.75 – 7.73 (m, 2H), 7.64 – 7.61 (m, 2H), 7.51 – 7.40 (m, 3H), 3.81 (s, 4H), 3.69 – 3.66 (m, 2H), 3.43 – 3.40 (m, 2H); **^{13}C NMR** (100 MHz, $CDCl_3$) δ 190.7, 165.4, 147.6, 139.4, 131.7, 130.2, 129.0, 128.6, 127.7, 127.3, 66.7, 66.6, 46.3, 41.6.

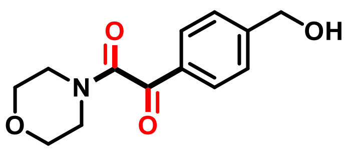

4aq 1-(4-(hydroxymethyl)phenyl)-2-morpholinoethane-1,2-dione: The title compound was prepared from (4-iodophenyl)methanol (234 mg, 1.0 mmol) and morpholine (130.5 mg, 1.5 mmol) following the general procedure and purified by column chromatography using petroleum ether/diethyl ether (2:1) to afford the product as a white solid (211.7 mg, 85% yield); **^{1}H NMR** (400 MHz, $CDCl_3$) δ 7.79 – 7.77 (m, 2H), 7.39 – 7.37 (m, 2H), 4.66 (s, 2H), 3.75 – 3.70 (m, 4H), 3.60 – 3.58 (m, 2H), 3.31 – 3.29 (m, 2H); **^{13}C NMR** (100 MHz, $CDCl_3$) δ 190.7, 165.5, 149.1, 131.6, 129.6, 126.7, 66.5, 66.5, 63.4, 46.1, 41.5. HRMS (ESI) $[M+Na]^+$: calculated for $[C_{13}H_{15}NNaO_4]^+$: 272.0899, found: 272.0933.

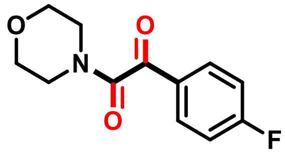
4ar 1-(4-fluorophenyl)-2-morpholinoethane-1,2-dione (*70*): The title compound was prepared from 1-fluoro-4-iodobenzene (222 mg, 1.0 mmol) and morpholine (130.5 mg, 1.5 mmol) following the general procedure and purified by column chromatography using petroleum ether/diethyl ether (3:1) to afford the product as a white solid (199.1 mg, 84% yield); **^{1}H NMR** (400 MHz, $CDCl_3$) δ 7.99 – 7.96 (m, 2H), 7.17 (t, J = 8.4 Hz, 2H), 3.76 (d, J = 1.7 Hz, 4H), 3.63 (t, J = 4.9 Hz, 2H), 3.64 – 3.35 (m, 2H); **^{13}C NMR** (100 MHz, $CDCl_3$) δ 189.3, 168.0, 165.2 (d, J = 42.3 Hz), 132.4 (d, J = 9.8 Hz), 129.5 (d, J = 2.9 Hz), 116.4 (d, J = 22.2 Hz), 66.6, 66.5, 46.2, 41.6; **^{19}F NMR**(400 MHz, $CDCl_3$) δ -101.19.

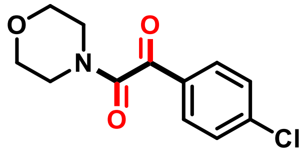
4as 1-(4-chlorophenyl)-2-morpholinoethane-1,2-dione (*17*): The title compound was prepared from 1-chloro-4-iodobenzene (238 mg, 1.0 mmol) and morpholine (130.5 mg, 1.5 mmol) following the general procedure and purified by column chromatography using petroleum ether/diethyl ether (2:1) to afford the product as a white solid (215.1 mg, 85% yield); **^{1}H NMR** (400 MHz, $CDCl_3$) δ 7.88-7.85 (m, 2H), 7.47 – 7.44 (m, 2H), 3.76-3.71 (m, 4H), 3.63 – 3.60 (m, 2H), 3.35 – 3.32 (m, 2H); **^{13}C NMR** (100 MHz, $CDCl_3$) δ 189.6, 164.7, 141.4, 131.3, 130.9, 129.3, 66.6, 66.5, 46.1, 41.5.

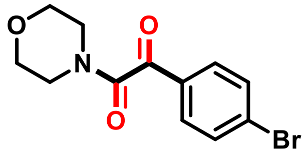
4at 1-(4-bromophenyl)-2-morpholinoethane-1,2-dione (*70*): The title compound was prepared from 1-bromo-4-iodobenzene (282 mg, 1.0 mmol) and morpholine (130.5 mg, 1.5 mmol) following the general procedure and purified by column chromatography using petroleum ether/diethyl ether (4:1) to afford the product as a white solid (241.4 mg, 81% yield); **^{1}H NMR** (400 MHz, $CDCl_3$) δ 7.77 – 7.74 (m, 2H), 7.61 – 7.58 (m, 2H), 3.73-3.68 (m, 4H), 3.59 – 3.56 (m, 2H), 3.31 – 3.29 (m, 2H); **^{13}C NMR** (100 MHz, $CDCl_3$) δ 189.7, 164.6, 132.2, 131.6, 130.8, 130.2, 66.5, 66.4, 46.0, 41.4.

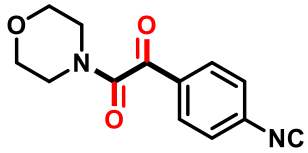
4au 1-(4-isocyanophenyl)-2-morpholinoethane-1,2-dione (*70*): The title compound was prepared from 4-iodobenzonitrile (229 mg, 1.0 mmol) and morpholine (130.5 mg, 1.5 mmol) following the general procedure and purified by column chromatography using petroleum ether/diethyl ether (2:1) to afford the product as a white solid (195.2 mg, 80% yield); **^{1}H NMR** (400 MHz, $CDCl_3$) δ 8.06 – 8.04 (m, 2H), 7.81 – 7.79 (m, 2H), 3.80-3.74 (m, 4H), 3.66 – 3.64 (m, 2H), 3.39 – 3.36 (m, 2H); **^{13}C NMR** (100 MHz, $CDCl_3$) δ 188.9, 164.0, 135.9, 132.7, 130.0, 117.8, 117.5, 66.6, 66.5, 46.2, 41.8.

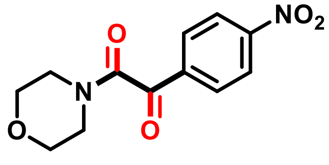
4av 1-morpholino-2-(4-nitrophenyl)ethane-1,2-dione (*70*): The title compound was prepared from 1-iodo-4-nitrobenzene (249 mg, 1.0 mmol) and morpholine (130.5 mg, 1.5 mmol) following the general procedure and purified by column chromatography using petroleum ether/diethyl ether (2:1) to afford the product as a white solid (216.5 mg, 82% yield); **^{1}H NMR** (400 MHz, $CDCl_3$) δ 8.36 -8.33 (m, 2H), 8.16 -8.14 (m, 2H), 3.80 (s, 4H), 3.70 – 3.37 (m, 2H), 3.46 - 3.38 (m, 2H); **^{13}C NMR** (100 MHz, $CDCl_3$) δ 188.6, 164.0, 151.1, 137.4, 130.8, 124.1, 66.7, 66.6, 46.3, 41.9.

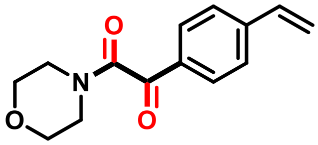

4aw 1-morpholino-2-(4-vinylphenyl)ethane-1,2-dione: The title compound was prepared from 1-iodo-4-vinylbenzene (230 mg, 1.0 mmol) and morpholine (130.5 mg, 1.5 mmol) following the general procedure and purified by column chromatography using petroleum ether/diethyl ether (2:1) to afford the product as a white solid (213.2 mg, 87% yield); **^{1}H NMR** (400 MHz, $CDCl_3$) δ 7.89 (d, *J* = 8.6 Hz, 2H), 7.56 (d, *J* = 8.5 Hz, 2H), 5.72 (t, *J* = 1.0 Hz, 1H), 5.49 (t, *J* = 1.0 Hz, 1H), 5.23 (t, *J* = 1.5 Hz, 1H), 3.76 (s, 4H), 3.62 (dd, *J* = 5.6, 4.1 Hz, 2H), 3.35 (dd, *J* = 5.5, 4.1 Hz, 2H), 2.14 (t, *J* = 1.1 Hz, 3H); **^{13}C NMR** (100 MHz, $CDCl_3$) δ ^{13}C NMR (100 MHz, $CDCl_3$) δ 190.56, 165.37, 147.57, 142.00, 131.71, 129.65, 129.63, 126.03, 125.96, 115.74, 66.62, 66.53, 46.13, 41.46. HRMS (ESI) $[M+H]^+$: calculated for $[C_{14}H_{15}NNaO_3]^+$: 268.0950, found: 268.0947.

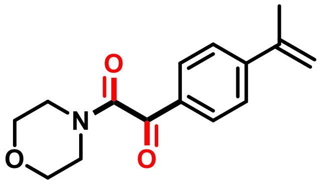

4ax 1-morpholino-2-(4-(prop-1-en-2-yl)phenyl)ethane-1,2-dione: The title compound was prepared from 1-iodo-4-(prop-1-en-2-yl)benzene (244 mg, 1.0 mmol) and morpholine (130.5 mg, 1.5 mmol) following the general procedure and purified by column chromatography using petroleum ether/diethyl ether (2:1) to afford the product as a white solid (235.8 mg, 91% yield); **^{1}H NMR** (400 MHz, $CDCl_3$) δ 7.89 (d, *J* = 8.6 Hz, 2H), 7.56 (d, *J* = 8.5 Hz, 2H), 5.49 (t, *J* = 1.0 Hz, 1H), 5.23 (t, *J* = 1.5 Hz, 1H), 3.76 (s, 4H), 3.62 (dd, *J* = 5.6, 4.1 Hz, 2H), 3.35 (dd, *J* = 5.5, 4.1 Hz, 2H), 2.14 (t, *J* = 1.1 Hz, 3H); **^{13}C NMR** (100 MHz, $CDCl_3$) δ ^{13}C NMR (100 MHz, $CDCl_3$) δ 190.56 , 165.37 , 147.57 , 142.00 , 131.71 , 129.65 , 129.63 , 126.03 , 125.96 , 115.74 , 66.62 , 66.53 , 46.13 , 41.46 , 21.44 , 21.34. HRMS (ESI) $[M+H]^+$: calculated for $[C_{15}H_{17}NNaO_3]^+$: 282.1106, found: 282.1114.

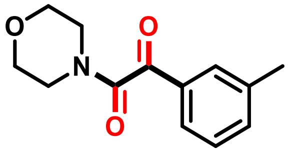

4ay 1-morpholino-2-(m-tolyl)ethane-1,2-dione (*84*): The title compound was prepared from 1-iodo-3-methylbenzene (218 mg, 1.0 mmol) and morpholine (130.5 mg, 1.5 mmol) following the general procedure and purified by column chromatography using petroleum ether/diethyl ether (1:1) to afford the product as a white solid (188.8 mg, 81% yield); **^{1}H NMR** (400 MHz, $CDCl_3$) δ 7.70 – 7.68 (m, 2H), 7.41 (dd, *J* = 7.6, 1.8 Hz, 1H), 7.35 (t, *J* = 7.3 Hz, 1H), 3.75-3.71 (m, 4H), 3.60 – 3.57 (m, 2H), 3.31 (dd, *J* = 5.5, 4.1 Hz, 2H), 2.36 (s, 3H); **^{13}C NMR** (100 MHz, $CDCl_3$) δ 191.2, 165.4, 138.9, 135.6, 132.8, 129.7, 128.8, 126.7, 66.5, 66.4, 46.0, 41.4, 21.1.

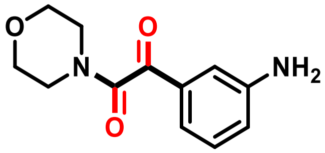

4az 1-(3-aminophenyl)-2-morpholinoethane-1,2-dione: The title compound was prepared from 3-iodoaniline (219 mg, 1.0 mmol) and morpholine (130.5 mg, 1.5 mmol) following the general procedure and purified by column chromatography using petroleum ether/diethyl ether (0.5:1) to afford the product as a white solid (203.6 mg, 87% yield); **^{1}H NMR** (400 MHz, $CDCl_3$) δ 7.19 – 7.16 (m, 3H), 6.85 (pd, *J* = 3.9, 2.4 Hz, 1H), 4.03 (s, 2H), 3.70 (p, *J* = 2.0 Hz, 4H), 3.57 (dd, *J* = 5.6, 4.1 Hz, 2H), 3.28 (dd, *J* = 5.5, 4.0 Hz, 2H); **^{13}C NMR** (100 MHz, $CDCl_3$) δ 191.6, 165.5, 147.4, 133.5, 129.7, 121.3, 119.5, 114.1, 66.4, 66.3, 46.0, 41.3. HRMS (ESI) $[M+Na]^+$: calculated for $[C_{12}H_{14}N_2NaO_3]^+$: 257.0902, found: 257.0906.

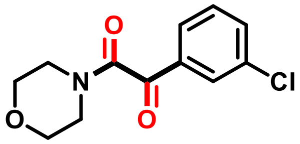

4ba 1-(3-chlorophenyl)-2-morpholinoethane-1,2-dione (*84*): The title compound was prepared from 1-chloro-3-iodobenzene (238 mg, 1.0 mmol) and morpholine (130.5 mg, 1.5 mmol) following the

general procedure and purified by column chromatography using petroleum ether/diethyl ether (2:1) to afford the product as a white solid (215.1 mg, 85% yield); **^{1}H NMR** (400 MHz, $CDCl_3$) δ 7.89 (t, *J* = 1.9 Hz, 1H), 7.79 (dt, *J* = 7.7, 1.4 Hz, 1H), 7.57 (ddd, *J* = 8.0, 2.2, 1.1 Hz, 1H), 7.42 (t, *J* = 7.9 Hz, 1H), 3.74 (q, *J* = 2.4 Hz, 4H), 3.63 – 3.60 (m, 2H), 3.35 - 3.32 (m, 2H); **^{13}C NMR** (100 MHz, $CDCl_3$) δ 189.4, 164.5, 135.2, 134.7, 134.4, 130.3, 129.2, 127.7, 66.5, 66.4, 46.1, 41.6.

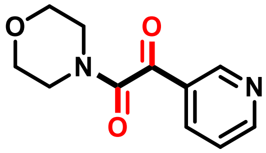

4bb 1-morpholino-2-(pyridin-3-yl)ethane-1,2-dione (*75*): The title compound was prepared from 3-iodopyridine (205 mg, 1.0 mmol) and morpholine (130.5 mg, 1.5 mmol) following the general procedure and purified by column chromatography using petroleum ether/diethyl ether (4:1) to afford the product as a white solid (184.8 mg, 84% yield); **^{1}H NMR** (400 MHz, $CDCl_3$) δ 9.09 (d, *J* = 2.2 Hz, 1H), 8.80 (dd, *J* = 4.9, 1.7 Hz, 1H), 8.21 (dt, *J* = 8.0, 2.0 Hz, 1H), 7.43 (dd, *J* = 8.0, 4.8 Hz, 1H), 3.74 (q, *J* = 2.1 Hz, 4H), 3.64 – 3.61 (m, 2H), 3.39 – 3.36 (m, 2H); **^{13}C NMR** (100 MHz, $CDCl_3$) δ 189.3, 164.0, 147.4, 133.5, 129.7, 121.3, 119.5, 114.1, 66.4, 66.3, 46.0, 41.3.

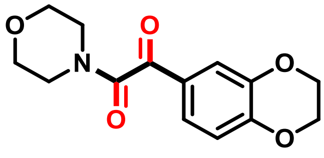

4bc 1-(2,3-dihydrobenzo[*b*][1,4]dioxin-6-yl)-2-morpholinoethane-1,2-dione (*70*): The title compound was prepared from 6-iodo-2,3-dihydrobenzo[*b*][1,4]dioxine (262 mg, 1.0 mmol) and morpholine (130.5 mg, 1.5 mmol) following the general procedure and purified by column chromatography using petroleum ether/diethyl ether (2:1) to afford the product as a white solid (249.3 mg, 90% yield); **^{1}H NMR** (400 MHz, $CDCl_3$) δ 7.37 (d, *J* = 7.9 Hz, 2H), 6.86 (d, *J* = 8.1 Hz, 1H), 4.30 – 4.16 (m, 4H), 3.68 (tt, *J* = 4.5, 2.6 Hz, 4H), 3.59 – 3.52 (m, 2H), 3.30 – 3.23 (m, 2H); **^{13}C NMR** (100 MHz, $CDCl_3$) δ 189.6, 166.4, 149.5, 143.6, 126.4, 123.8, 118.4, 117.6, 66.4, 66.3, 64.6, 63.8, 46.0, 41.2.

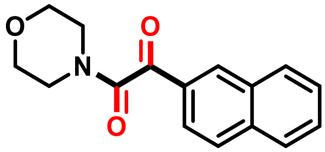

4bd 1-morpholino-2-(naphthalen-2-yl)ethane-1,2-dione (*77*): The title compound was prepared from 2-iodonaphthalene (254 mg, 1.0 mmol) and morpholine (130.5 mg, 1.5 mmol) following the general procedure and purified by column chromatography using petroleum ether/diethyl ether (4:1) to afford the product as a white solid (201.9 mg, 75% yield); **^{1}H NMR** (400 MHz, $CDCl_3$) δ 9.22 (d, *J* = 8.6 Hz, 1H), 8.03 (dd, *J* = 25.2, 7.7 Hz, 2H), 7.86 (d, *J* = 8.1 Hz, 1H), 7.65 (ddd, *J* = 8.5, 6.8, 1.5 Hz, 1H), 7.59 – 7.47 (m, 2H), 3.77 (s, 4H), 3.64 – 3.57 (m, 2H), 3.42 – 3.34 (m, 2H); **^{13}C NMR** (100 MHz, $CDCl_3$) δ 193.4, 165.8, 136.0, 134.3, 133.8, 130.6, 129.2, 128.6, 128.1, 126.9, 125.5, 124.3, 66.4, 66.4, 46.1, 41.5.

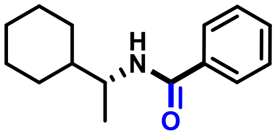

3be *(R)-N*-(1-cyclohexylethyl)benzamide (*85*): The title compound was prepared from iodobenzene (204 mg, 1.0 mmol) and (*R*)-1-cyclohexylethan-1-amine (190.5 mg, 1.5 mmol) following the general procedure and purified by column chromatography using petroleum ether/diethyl ether (6:1) to afford the product as a white solid (205.6 mg, 89% yield); $[a]_D^{20}$= -16.35 (c 0.422 in $CHCl_3$), **^{1}H NMR** (400 MHz, $CDCl_3$) δ 7.80 – 7.70 (m, 2H), 7.51 – 7.44 (m, 1H), 7.40 (dd, *J* = 8.2, 6.5 Hz, 2H), 6.08 (s, 1H), 4.06 (dp, *J* = 9.1, 6.7 Hz, 1H), 1.86 – 1.60 (m, 6H), 1.42 (dddt, *J* = 11.9, 9.3, 6.2, 3.1 Hz, 1H), 1.23 – 1.14 (m, 5H), 1.09 – 0.99 (m, 2H); **^{13}C NMR** (100 MHz, $CDCl_3$) δ 166.7, 134.9, 131.1, 128.3, 126.8, 50.1, 20.3, 17.3, 3.2, 3.1. HPLC-separation conditions: Chiralcel AD-H column, 30 ºC, 254 nm, hexane/i-PrOH = 90/10; flow rate 1.0 mL/min; t_1 = 6.414 min, t_2 = 6.891 min, t_R = 6.507 min (minor), t_R = 6.841 min (major); 97% ee.

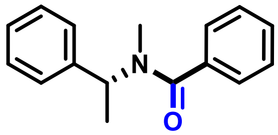

3bf *(R)*-*N*-methyl-*N*-(1-phenylethyl)benzamide (*86*): The title compound was prepared from iodobenzene (204 mg, 1.0 mmol) and (*R*)-*N*-methyl-1-phenylethan-1-amine (202.5 mg, 1.5 mmol) following the general procedure and purified by column chromatography using petroleum ether/diethyl ether (6:1) to afford the product as a white solid (227.1 mg, 95% yield); $[a]_D^{20}$= -12.76 (c 0.392 in $CHCl_3$), **^{1}H NMR** (400 MHz, $CDCl_3$) δ 7.30-7.24 (m, 5H), 4.46-4.33 (m, 1H), 2.78 (d, *J* = 76.1 Hz, 3H), 1.81 – 1.24 (m, 8H), 0.97-0.92 (m, 2H); **^{13}C NMR** (100 MHz, $CDCl_3$) δ 171.3, 136.9, 128.8, 128.0, 126.4, 125.7, 57.9, 52.4, 30.4, 29.3, 27.1, 25.2, 24.8. HPLC-separation conditions: Chiralcel AD-H column, 30 °C, 254 nm, hexane/i-PrOH = 90/10; flow rate 1.0 mL/min; t_1 = 6.505 min, t_2 = 6.982 min, t_R = 6.134 min (minor), t_R =6.727 min (major); 99% ee.

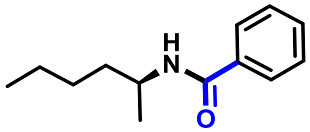

3bg *(R)*-*N*-(hexan-2-yl)benzamide (*87*): The title compound was prepared from iodobenzene (204 mg, 1.0 mmol) and (*S*)-hexan-2-amine (151.5 mg, 1.5 mmol) following the general procedure and purified by column chromatography using petroleum ether/diethyl ether (6:1) to afford the product as a white solid (192.7 mg, 94% yield); $[a]_D^{20}$= -17.59 (c 0.290 in $CHCl_3$), **^{1}H NMR** (400 MHz, $CDCl_3$) δ 7.85 – 7.75 (m, 2H), 7.46 – 7.39 (m, 1H), 7.34 (dd, *J* = 8.3, 6.8 Hz, 2H), 6.66 (d, *J* = 8.4 Hz, 1H), 4.15 (dq, *J* = 8.1, 6.4 Hz, 1H), 1.51 (dddd, *J* = 22.8, 14.1, 6.8, 4.2 Hz, 2H), 1.31 (hd, *J* = 7.2, 2.4 Hz, 4H), 1.19 (d, *J* = 6.6 Hz, 3H), 0.87 (td, *J* = 6.1, 5.2, 2.8 Hz, 3H); **^{13}C NMR** (100 MHz, $CDCl_3$) δ 166.7, 134.8, 130.8, 128.0, 126.8, 45.5, 36.4, 28.2, 22.4, 20.7, 13.8. HPLC-separation conditions: Chiralcel OD-H column, 40 °C, 254 nm, hexane/i-PrOH = 85/15; flow rate 1.0 mL/min; t_1 = 3.935 min, t_2 = 4.353 min, t_R = 4.045 min (major), t_R = 4.430 min (minor); 97% ee.

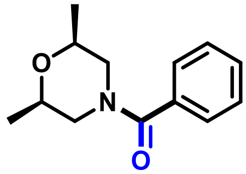

3bh *((2S,6R)*-2,6-dimethylmorpholino)(phenyl)methanone (*38*): The title compound was prepared from iodobenzene (204 mg, 1.0 mmol) and (2*R*,6*S*)-2,6-dimethylmorpholine (172.5 mg, 1.5 mmol) following the general procedure and purified by column chromatography using petroleum ether/diethyl ether (1:1) to afford the product as a white solid (208.1 mg, 95% yield); $[a]_D^{20}$= 1.45 (c 0.690 in $CHCl_3$), **^{1}H NMR** (400 MHz, $CDCl_3$) δ 7.40-7.37 (m, 5H), 4.57 - 4.54 (m, 1H), 3.65-3.50 (m, 3H), 2.81-2.49 (m, 2H), 1.22 (s, 3H), 1.07 (s, 3H); **^{13}C NMR** (100 MHz, $CDCl_3$) δ 170.0, 135.5, 129.7, 128.5, 127.0, 71.9, 53.2, 47.4, 18.6.

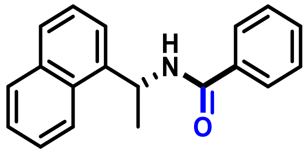

3bi *(R)*-*N*-(1-(naphthalen-1-yl)ethyl)benzamide (*88*): The title compound was prepared from iodobenzene (204 mg, 1.0 mmol) and (*R*)-1-(naphthalen-1-yl)ethan-1-amine (256.5 mg, 1.5 mmol) following the general procedure and purified by column chromatography using petroleum ether/diethyl ether (1:1) to afford the product as a white solid (253.1 mg, 92% yield); $[a]_D^{20}$= -18.15 (c 0.248 in $CHCl_3$), **^{1}H NMR** (400 MHz, $CDCl_3$) δ 8.18 – 8.16 (m, 1H), 7.87 (dd, *J* = 8.1, 1.5 Hz, 1H), 7.82 (dd, *J* = 8.3, 1.1 Hz, 1H), 7.75 – 7.70 (m, 2H), 7.59 (dt, *J* = 7.2, 1.0 Hz, 1H), 7.56 – 7.42 (m, 4H), 7.40 – 7.34 (m, 2H), 6.38 (d, *J* = 8.1 Hz, 1H), 6.12 (p, *J* = 7.0 Hz, 1H), 1.78 (d, *J* = 6.7 Hz, 3H); **^{13}C NMR** (100 MHz, $CDCl_3$) δ 166.4, 138.1, 134.4, 131.4, 131.2, 128.8, 128.5, 126.9, 126.7, 125.9, 125.2, 123.4, 122.7, 45.2, 20.6. HPLC-separation conditions: Chiralcel AD-H column, 30 °C, 254 nm, hexane/i-PrOH = 90/10; flow rate 1.0 mL/min; t_1 = 10.193 min, t_2 = 15.356 min, t_R = 10.197 min (major), t_R = 15.400 min (minor); 93% ee.

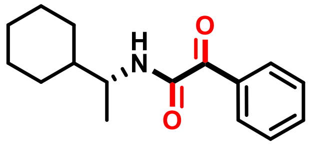

4be *(R)*-*N*-(1-cyclohexylethyl)-2-oxo-2-phenylacetamide: The title compound was prepared from iodobenzene (204 mg, 1.0 mmol) and (*R*)-1-cyclohexylethan-1-amine (190.5 mg, 1.5 mmol) following

the general procedure and purified by column chromatography using petroleum ether/diethyl ether (6:1) to afford the product as a white solid (225.3 mg, 87% yield); $[a]^{20}_{D}$=-17.31 (c 0.468 in $CHCl_3$), **^{1}H NMR** (400 MHz, $CDCl_3$) δ 8.37 – 8.27 (m, 2H), 7.62 – 7.54 (m, 1H), 7.45 (dd, *J* = 8.4, 7.2 Hz, 2H), 7.00 (s, 1H), 3.91 (dp, *J* = 9.4, 6.7 Hz, 1H), 1.82 – 1.69 (m, 4H), 1.68 – 1.59 (m, 1H), 1.47 – 1.31 (m, 1H), 1.27 – 0.97 (m, 8H); **^{13}C NMR** (100 MHz, $CDCl_3$) δ 188.0, 161.1, 134.2, 133.4, 131.1, 128.3, 49.7, 42.9, 29.1, 28.9, 26.2, 26.0, 17.7. HPLC-separation conditions: Chiralcel AD-H column, 30 ºC, 254 nm, hexane/i-PrOH = 90/10; flow rate 1.0 mL/min; t_1 = 5.957 min, t_2 = 7.105 min, t_R = 5.951 min (major), t_R = 7.101 min (minor); 99% ee. HRMS (ESI) $[M+Na]^+$: calculated for $[C_{16}H_{21}NNaO_2]^+$: 282.1470, found: 282.1474.

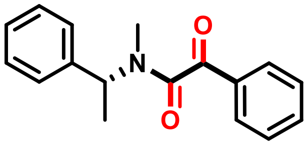

4bf *(R)*-*N*-methyl-2-oxo-2-phenyl-*N*-(1-phenylethyl)acetamide: The title compound was prepared from iodobenzene (204 mg, 1.0 mmol) and (*R*)-*N*-methyl-1-phenylethan-1-amine (202.5 mg, 1.5 mmol) following the general procedure and purified by column chromatography using petroleum ether/diethyl ether (6:1) to afford the product as a white solid (245.7 mg, 92% yield); $[a]^{20}_{D}$= 12.99 (c 0.462 in $CHCl_3$), **^{1}H NMR** (400 MHz, $CDCl_3$) δ 8.04 – 7.94 (m, 2H),, 7.65 (dt, *J* = 9.5, 7.5 Hz, 1H), 7.53 (dt, *J* = 11.5, 7.7 Hz, 2H), 7.42 – 7.38 (m, 2H), 7.36 – 7.26 (m, 3H), 4.89 (q, *J* = 7.1 Hz, 1H), 2.79 (s, 3H), 1.64 (d, *J* = 7.1 Hz, 3H); **^{13}C NMR** (100 MHz, $CDCl_3$) δ 191.7, 167.1, 139.0, 134.7, 133.2, 129.7, 129.0, 128.7, 128.0, 127.3, 127.1. HPLC-separation conditions: Chiralcel OD-H column, 40 ºC, 254 nm, hexane/i-PrOH = 90/10; flow rate 1.0 mL/min; t_1 = 7.164 min, t_2 = 7.774 min, t_R = 7.386 min (major), t_R = 8.096 min (minor); 99% ee. HRMS (ESI) $[M+Na]^+$: calculated for $[C_{17}H_{17}NNaO_2]^+$: 290.1157, found: 290.1143.

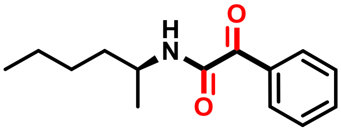

4bg *(S)*-*N*-(hexan-2-yl)-2-oxo-2-phenylacetamide: The title compound was prepared from iodobenzene (204 mg, 1.0 mmol) and (*S*)-hexan-2-amine (151.5 mg, 1.5 mmol) following the general procedure and purified by column chromatography using petroleum ether/diethyl ether (6:1) to afford the product as a white solid (216.7 mg, 93% yield); $[a]^{20}_{D}$=-13.05 (c 0.466 in $CHCl_3$), **^{1}H NMR** (400 MHz, $CDCl_3$) δ 8.39 – 8.24 (m, 2H), 7.63 – 7.52 (m, 1H), 7.43 (dd, *J* = 8.4, 7.2 Hz, 2H), 6.97 (s, 1H), 4.03 (dq, *J* = 8.9, 6.7 Hz, 1H), 1.50 (tdd, *J* = 8.6, 3.9, 2.4 Hz, 2H), 1.37 – 1.26 (m, 4H), 1.20 (d, *J* = 6.6 Hz, 3H), 0.91 – 0.83 (m, 3H); **^{13}C NMR** (100 MHz, $CDCl_3$) δ 188.0, 161.1, 134.1, 133.3, 131.0, 128.3, 45.5, 36.2, 28.1, 22.4, 20.5, 13.9. HPLC-separation conditions: Chiralcel AD-H column, 30 ºC, 254 nm, hexane/i-PrOH = 90/10; flow rate 1.0 mL/min; t_1 = 5.224 min, t_2 = 5.608 min, t_R = 5.211 min (major), t_R = 5.597 min (minor); 98% ee. HRMS (ESI) $[M+Na]^+$: calculated for $[C_{14}H_{19}NNaO_2]^+$: 256.1313, found: 256.1308.

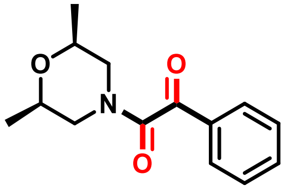

4bh 1-*((2R,6S)*-2,6-dimethylmorpholino)-2-phenylethane-1,2-dione: The title compound was prepared from iodobenzene (204 mg, 1.0 mmol) and (2*R*,6*S*)-2,6-dimethylmorpholine (172.5 mg, 1.5 mmol) following the general procedure and purified by column chromatography using petroleum ether/diethyl ether (4:1) to afford the product as a white solid (222.3 mg, 90% yield); $[a]^{20}_{D}$=0.71 (c 0.566 in $CHCl_3$), **^{1}H NMR** (400 MHz, $CDCl_3$) δ 7.88 – 7.85 (m, 2H), 7.56 (t, *J* = 7.5 Hz, 1H), 7.43 (t, *J* = 7.8 Hz, 2H), 4.42 (ddd, *J* = 13.2, 2.6, 1.6 Hz, 1H), 3.63 – 3.46 (m, 2H), 3.27 (dt, *J* = 13.2, 2.1 Hz, 1H), 2.78 (dd, *J* = 13.2, 10.6 Hz, 1H), 2.49 (dd, *J* = 13.2, 10.7 Hz, 1H), 1.17 (d, *J* = 6.3 Hz, 3H), 1.00 (d, *J* = 6.3 Hz, 3H); **^{13}C NMR** (100 MHz, $CDCl_3$) δ 190.9, 164.9, 134.6, 132.8, 129.4, 128.8, 71.6, 71.5, 50.9, 46.2, 18.5, 18.2. HRMS (ESI) $[M+Na]^+$: calculated for $[C_{14}H_{17}NNaO_3]^+$: 270.1106, found: 270.1101.

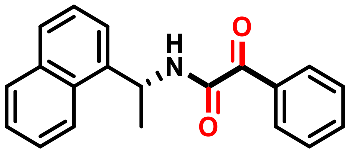
4bi *(R)-N*-(1-(naphthalen-1-yl)ethyl)-2-oxo-2-phenylacetamide (*89*): The title compound was prepared from iodobenzene (204 mg, 1.0 mmol) and (*R*)-1-(naphthalen-1-yl)ethan-1-amine (256.5 mg, 1.5 mmol) following the general procedure and purified by column chromatography using petroleum ether/diethyl ether (1:1) to afford the product as a white solid (239.5 mg, 79% yield); $[a]_D^{20}$=3.73 (c 0.402 in $CHCl_3$), **^{1}H NMR** (400 MHz, $CDCl_3$) δ 8.36 (dd, *J* = 8.4, 1.4 Hz, 2H), 8.17 (d, *J* = 9.0 Hz, 1H), 7.90 (d, *J* = 8.0 Hz, 1H), 7.83 (d, *J* = 8.2 Hz, 1H), 7.64 – 7.56 (m, 3H), 7.54 – 7.45 (m, 5H), 6.07 – 6.00 (m, 1H), 1.77 (d, *J* = 6.8 Hz, 3H); **^{13}C NMR** (100 MHz, $CDCl_3$) δ 187.6, 160.6, 137.6, 134.3, 133.9, 133.3, 131.2, 130.9, 128.9, 128.5, 128.4, 126.6, 125.9, 125.2, 123.0, 122.6, 44.9, 20.8. HPLC-separation conditions: Chiralcel OD-H column, 40 ºC, 254 nm, hexane/i-PrOH = 95/5; flow rate 1.0 mL/min; t_1 = 9.397 min, t_2 = 10.289 min, t_R = 9.654 min (minor), t_R = 10.468 min (major); 99% ee.

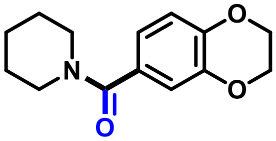
3bj CX-546 (*38*): The title compound was prepared from 6-iodo-2,3-dihydrobenzo[*b*][1,4]dioxine (262.0 mg, 1.0 mmol) and piperidine (127.5 mg, 1.5 mmol) following the general procedure and purified by column chromatography using petroleum ether/diethyl ether (3:1) to afford the product as a white solid (242.1 mg, 98% yield); **^{1}H NMR** (400 MHz, $CDCl_3$) δ 6.88-6.79 (m, 3H), 4.33 (s, 4H), 3.63-3.36 (m, 4H), 1.63-1.47 (m, 6H); **^{13}C NMR** (100 MHz, $CDCl_3$) δ 169.7, 144.6, 143.2, 129.6, 120.4, 117.1, 115.4, 64.4, 64.3, 24.6.

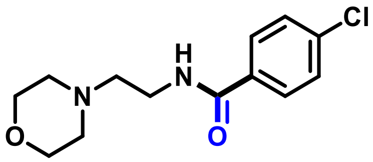
3bk 4-chloro-*N*-(2-morpholinoethyl)benzamide (Moclobemide) (*38*): The title compound was prepared from 1-chloro-4-iodobenzene 1-chloro-4-iodobenzene (238.0 mg, 1.0 mmol) and 2-morpholinoethan-1-amine (195.0 mg, 1.5 mmol) following the general procedure and purified by column chromatography using petroleum ether/diethyl ether (0.5:1) to afford the product as a white solid (254.6 mg, 95% yield); **^{1}H NMR** (400 MHz, $CDCl_3$) δ7.74 – 7.72 (m, 2H), 7.36 (t, *J* = 7.5 Hz, 2H), 6.97 (brs, 1H), 3.74 – 3.72 (m, 4H), 3.48 (q, *J* = 6.1 Hz, 2H), 2.52 (t, *J* = 6.2 Hz, 2H), 2.44 – 2.41 (m, 4H); **^{13}C NMR** (100 MHz, $CDCl_3$) δ 166.2, 137.4, 132.8, 128.6, 128.2, 66.8, 56.7, 53.2, 36.0.

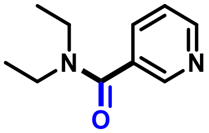
3bl *N,N*-diethylnicotinamide (Nikethamide) (*38*): The title compound was prepared from 3-iodopyridine (205.0 mg, 1.0 mmol) and diethylamine (109.5 mg, 1.5 mmol) following the general procedure and purified by column chromatography using petroleum ether/diethyl ether (0.5:1) to afford the product as a white solid (167.3 mg, 94% yield); **^{1}H NMR** (400 MHz, $CDCl_3$) δ 8.65 (dd, *J* = 4.9, 1.8 Hz, 2H), 7.73 (dt, *J* = 7.8, 2.0 Hz, 1H), 7.40 – 7.34 (m, 1H), 3.57 (q, *J* = 7.2 Hz, 2H), 3.28 (q, *J* = 7.2 Hz, 2H), 1.26 (t, *J* = 7.2 Hz, 3H), 1.15 (t, *J* = 7.2 Hz, 3H); **^{13}C NMR** (100 MHz, $CDCl_3$) δ 168.3, 150.0, 133.9, 132.7, 123.1, 43.2, 39.3, 14.0, 12.6.

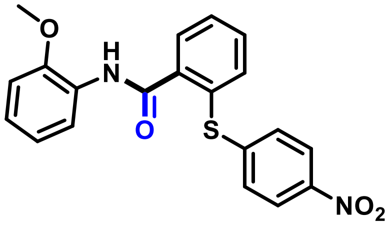
3bm *N*-(2-methoxyphenyl)-2-((4-nitrophenyl)thio)benzamide (RN-18) (*90*): The title compound was prepared from (2-iodophenyl)(4-nitrophenyl)sulfane (357.0 mg, 1.0 mmol) and 2-methoxyaniline (184.5 mg, 1.5 mmol) following the general procedure and purified by column chromatography using petroleum ether/diethyl ether (5:1) to afford the product as a white solid (304.0 mg, 80% yield); **^{1}H NMR** (400 MHz, $CDCl_3$) δ 8.45 – 8.36 (m, 2H), 8.09 – 8.01 (m, 2H), 7.82 – 7.75 (m, 1H), 7.59 – 7.47 (m, 3H), 7.31 – 7.24 (m, 2H),

7.06 (td, *J* = 7.8, 1.7 Hz, 1H), 6.96 (td, *J* = 7.7, 1.4 Hz, 1H), 6.86 (dd, *J* = 8.1, 1.4 Hz, 1H), 3.78 (s, 3H); **^{13}C NMR** (100 MHz, $CDCl_3$) δ 165.1, 147.9, 146.6, 145.8, 140.3, 135.4, 131.4, 129.8, 129.6, 129.2, 128.5, 127.2, 124.2, 124.0, 121.0, 119.7, 109.9, 55.8.

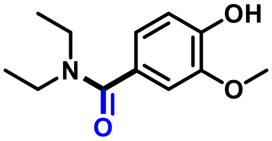

3bn *N,N*-diethyl-4-hydroxy-3-methoxybenzamide (Etamivan) (*91*): The title compound was prepared from 4-iodo-2-methoxyphenol (250.0 mg, 1.0 mmol) and diethylamine (109.5 mg, 1.5 mmol) following the general procedure and purified by column chromatography using petroleum ether/diethyl ether (1:1) to afford the product as a white solid (198.5 mg, 89% yield); **^{1}H NMR** (400 MHz, $CDCl_3$) δ 6.81-6.88 (m, 1H), 6.81 (d, J = 1.0 Hz, 2H), 3.78 (s, 3H), 3.41-3.25 (m, 4H), 1.13 (t, J = 7.2 Hz, 6H); **^{13}C NMR** (100 MHz, $CDCl_3$) δ 171.3, 146.9, 146.7, 128.3, 119.5, 114.1, 109.9, 55.7, 43.3, 39.5, 13.5.

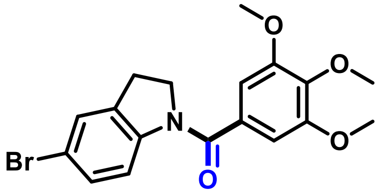

3bo (5-bromoindolin-1-yl)(3,4,5-trimethoxyphenyl)methanone (IV-23) (*92*): The title compound was prepared from 5-iodo-1,2,3-trimethoxybenzene (294 mg, 1.0 mmol) and 5-bromoindoline (297.0 mg, 1.5 mmol) following the general procedure and purified by column chromatography using petroleum ether/diethyl ether (1:1) to afford the product as a white solid (317.6 mg, 81% yield); **^{1}H NMR** (400 MHz, $CDCl_3$) δ 7.24 – 7.08 (m, 3H), 6.71 (s, 2H), 4.03 (t, *J* = 8.3 Hz, 2H), 3.80 (d, *J* = 11.9 Hz, 9H), 3.02 (t, *J* = 8.3 Hz, 2H); **^{13}C NMR** (100 MHz, $CDCl_3$) δ 168.2, 153.0, 141.5, 139.5, 134.5, 131.5, 129.7, 127.6, 116.0, 104.2, 60.6, 56.0, 50.5, 27.6.

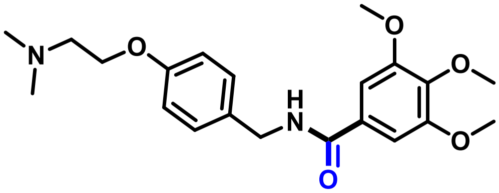

3bp *N*-(4-(2-(dimethylamino)ethoxy)benzyl)-3,4,5-trimethoxybenzamide (*93*): The title compound was prepared from 5-iodo-1,2,3-trimethoxybenzene (294 mg, 1.0 mmol) and 2-(4-(aminomethyl)phenoxy)-N,N-dimethylethan-1-amine (291.0 mg, 1.5 mmol) following the general procedure and purified by column chromatography using petroleum ether/diethyl ether (0.5:1) to afford the product as a white solid (392.8 mg, 85% yield); **^{1}H NMR** (400 MHz, $CDCl_3$) δ 7.30 – 7.23 (m, 2H), 7.04 (s, 2H), 6.92 – 6.85 (m, 2H), 5.30 (brs, 1H), 4.54 (d, J = 5.6 Hz, 2H), 4.05 (t, J = 5.6 Hz, 2H), 3.86 (s, 9H), 2.73 (t, J = 5.7 Hz, 2H), 2.34 (s, 6H); **^{13}C NMR** (100 MHz, $CDCl_3$) δ 166.9, 158.2, 153.0, 140.7, 131.9, 130.4, 129.8, 129.2, 114.7, 104.3, 67.0, 65.9, 60.8, 58.2, 56.2, 53.4, 45.8, 43.6.

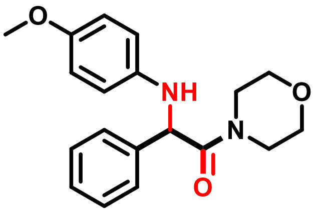

5a 2-((4-methoxyphenyl)amino)-1-morpholino-2-phenylethan-1-one: The title compound was prepared following the general procedure and purified by column chromatography using petroleum ether/diethyl ether (2:1) to afford the product as a white solid (494.0 mg, 76% yield); **^{1}H NMR** (400 MHz, $CDCl_3$) δ 7.35 (m, 5H), 7.19 (m, 2H), 6.84 (m, 2H), 5.77 (s, 1H), 4.84 (s, 1H), 3.67 (s, 3H), 3.58 – 3.32 (m, 6H), 3.10 – 2.86 (m, 2H); **^{13}C NMR** (100 MHz, $CDCl_3$) δ 169.7, 152.5, 140.6, 138.3, 129.0, 128.1, 127.6, 115.4, 114.8, 59.4, 55.7, 45.9, 42.8.

HPLC Spectra of Chiral compounds

(R)-N-(1-cyclohexylethyl)benzamide (**3be**)

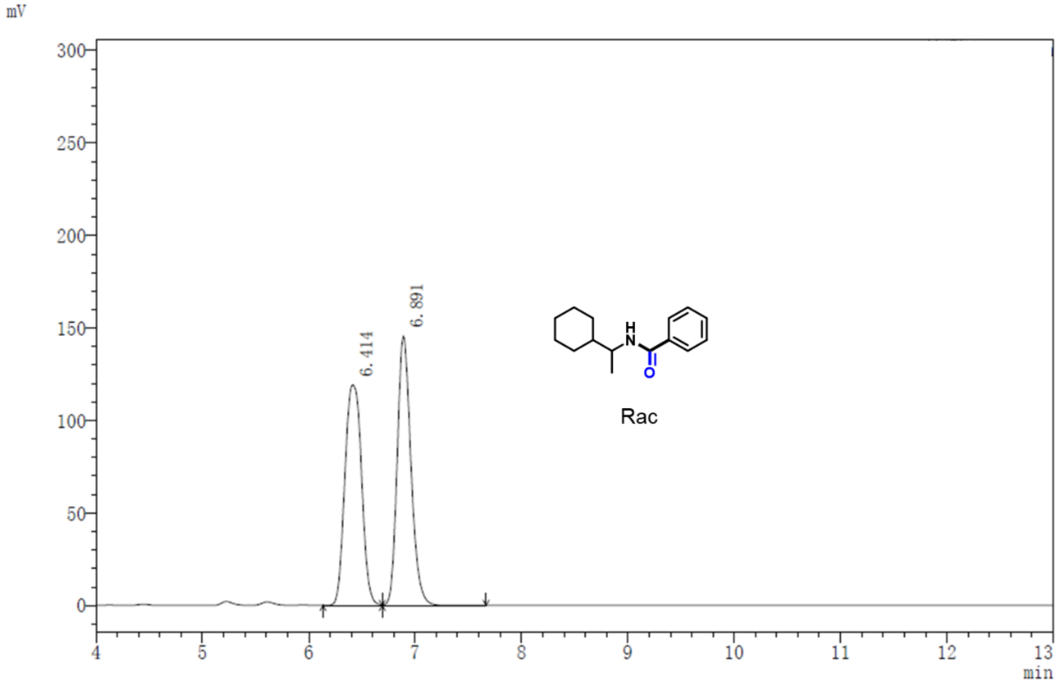

	Time	Area	Height	Area %
1	6.414	1302054	119232	49.97
2	6.891	1303492	145514	50.03

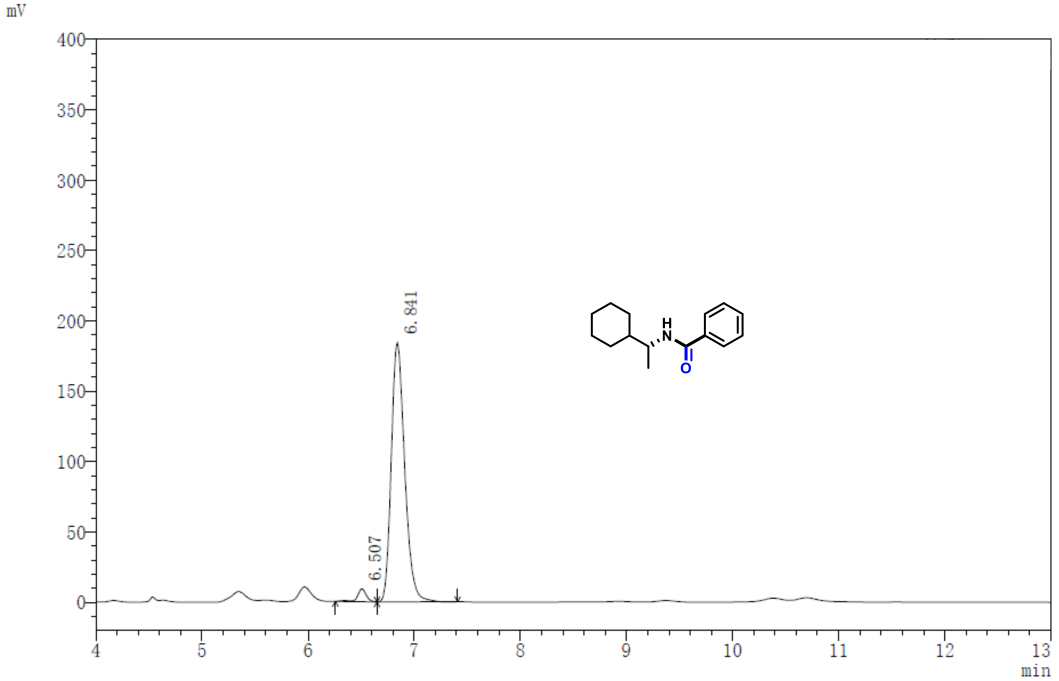

	Time	Area	Height	Area %
1	6.507	52339	9071	3.13
2	6.841	1618948	184051	96.87

***(R)*-N-methyl-*N*-(1-phenylethyl)benzamide (3bf)**

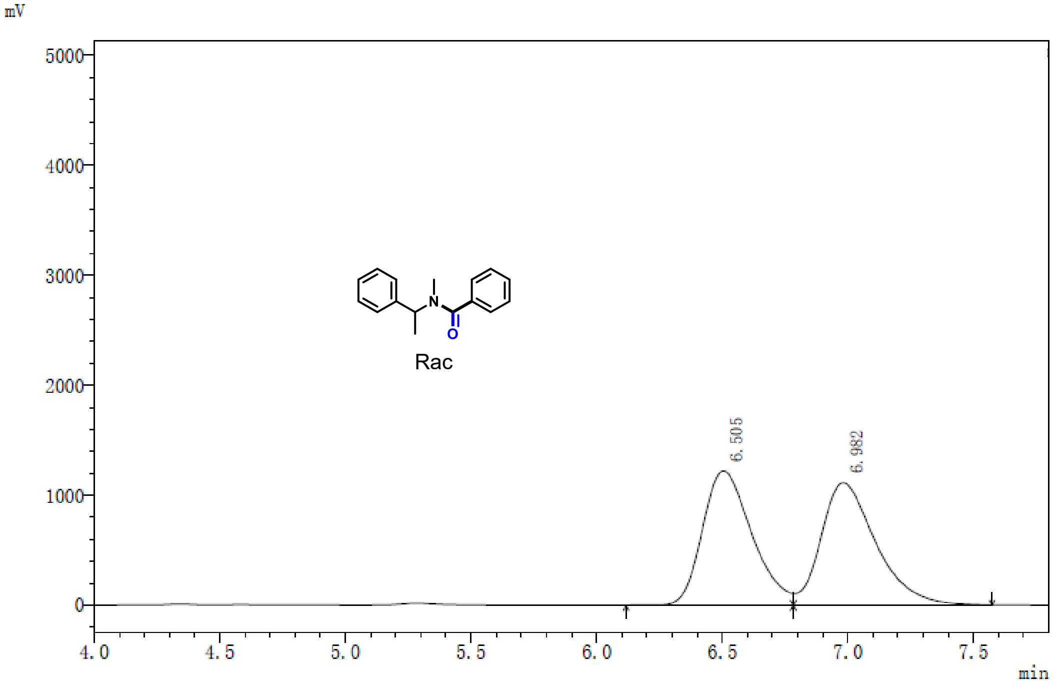

	Time	Area	Height	Area %
1	6.505	16729143	1223081	49.65
2	6.982	16961537	1114637	50.35

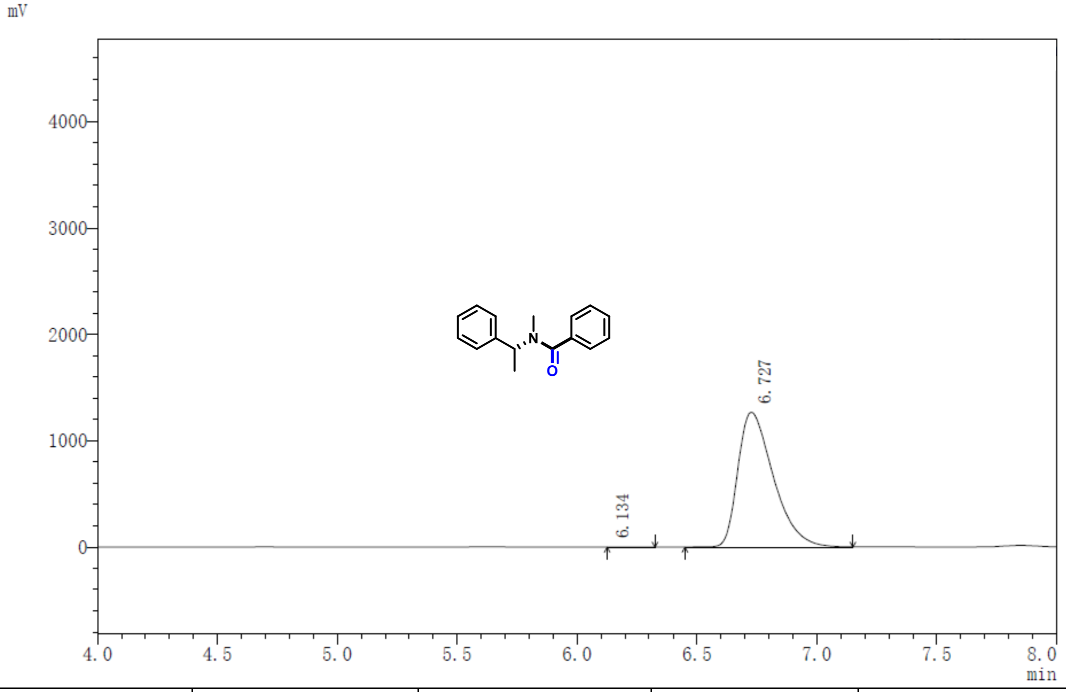

	Time	Area	Height	Area %
1	6.134	318	56	0.01
2	6.727	13086513	1268712	99.99

(R)-N-(hexan-2-yl)benzamide (3bg)

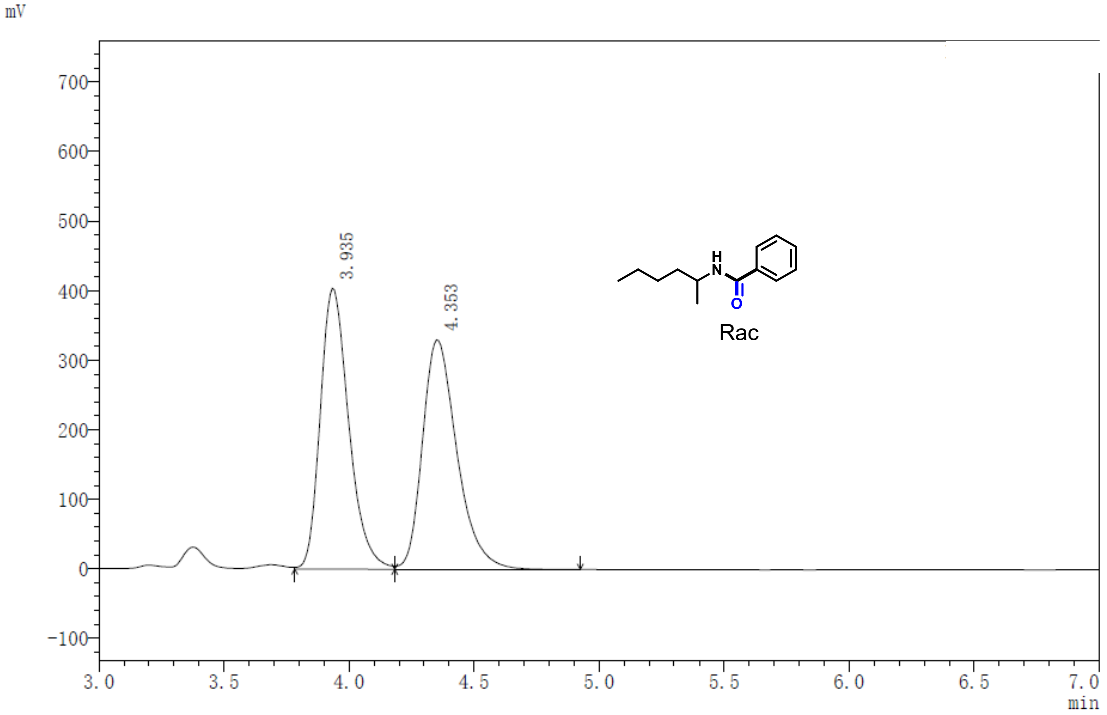

	Time	Area	Height	Area %
1	3.935	3204994	404050	50.33
2	4.353	3162533	329979	49.67

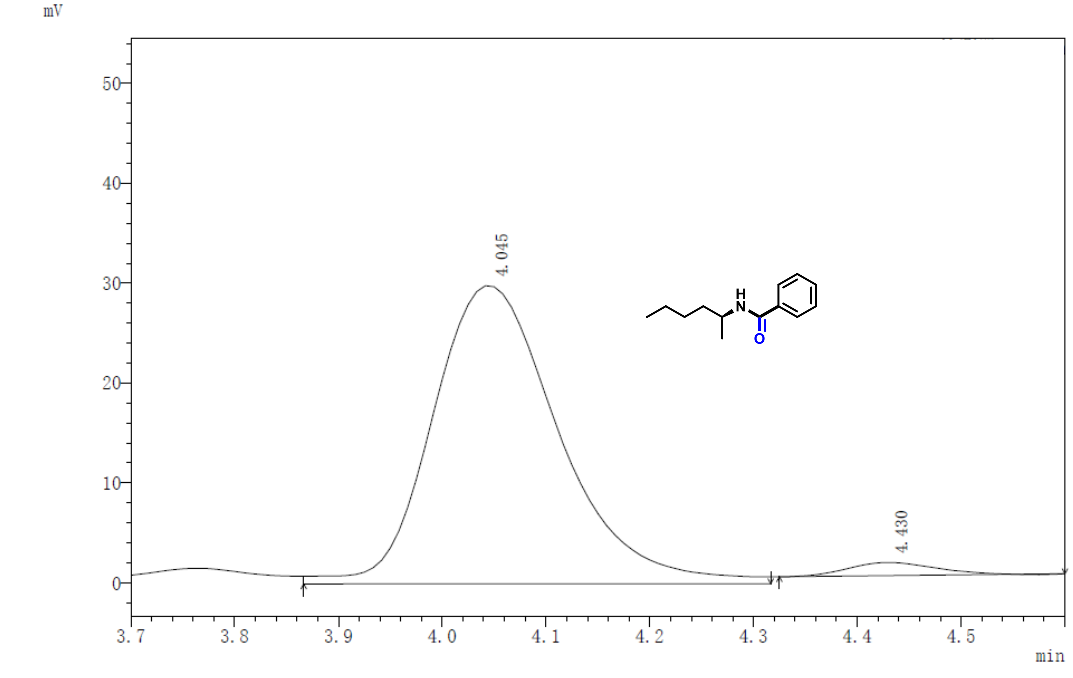

	Time	Area	Height	Area %
1	4.045	253560	29847	97.07
2	4.430	7651	1316	2.93

***(R)*-*N*-(1-(naphthalen-1-yl)ethyl)benzamide (3bi)**

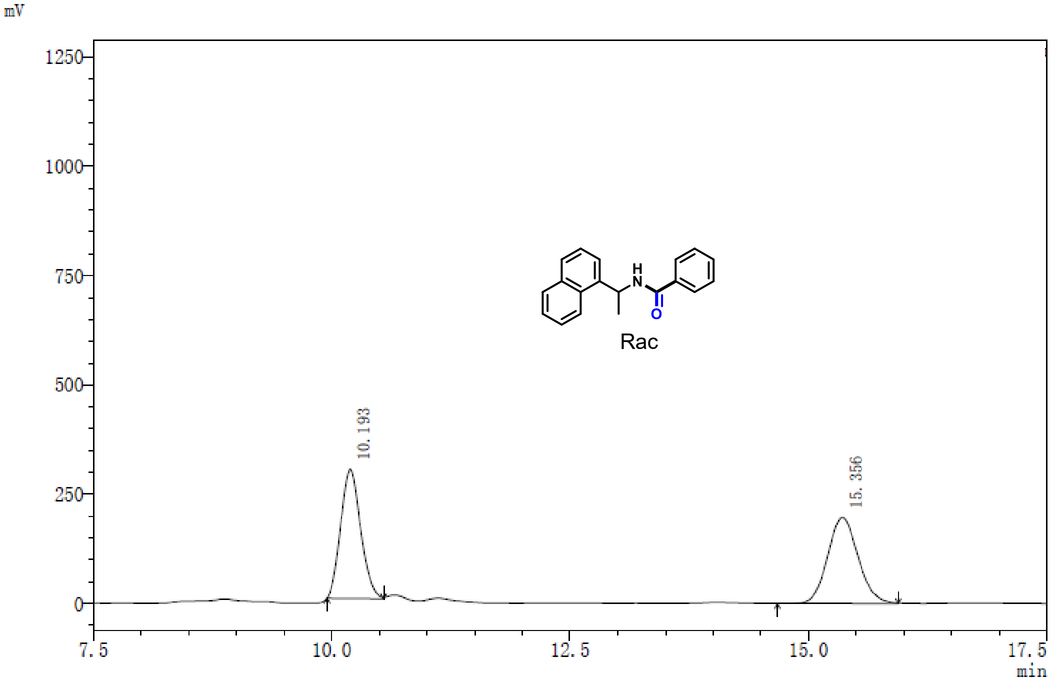

	Time	Area	Height	Area %
1	10.193	4321052	295049	49.59
2	15.356	4393269	197015	50.41

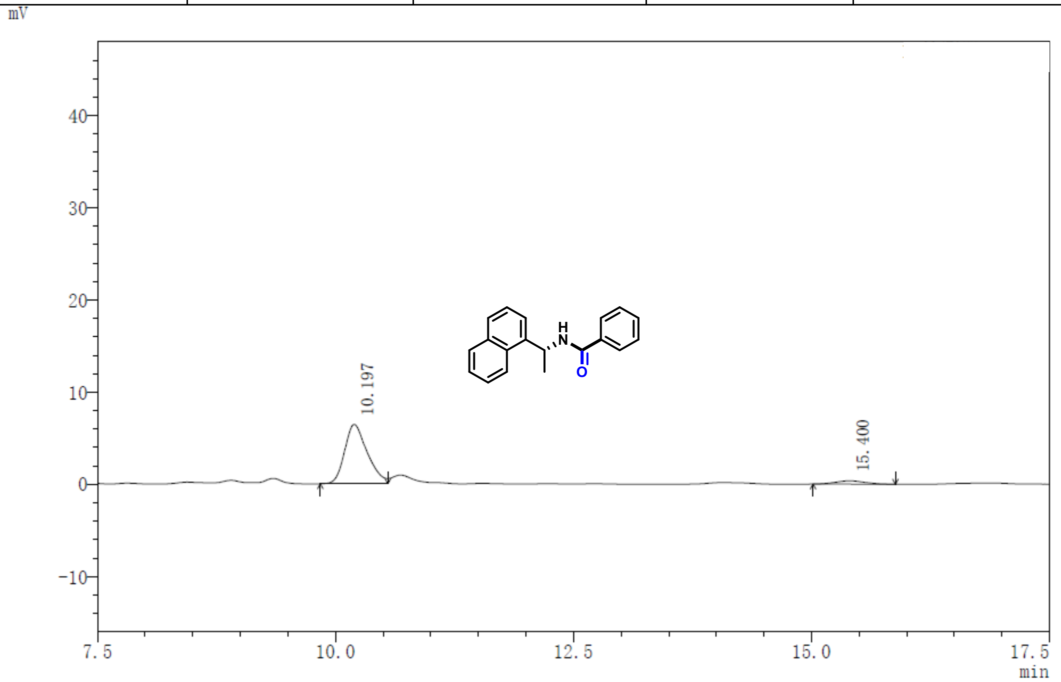

	Time	Area	Height	Area %
1	10.197	107008	6447	93.24
2	15.400	7761	363	6.76

(R)-N-(1-cyclohexylethyl)-2-oxo-2-phenylacetamide (4be)

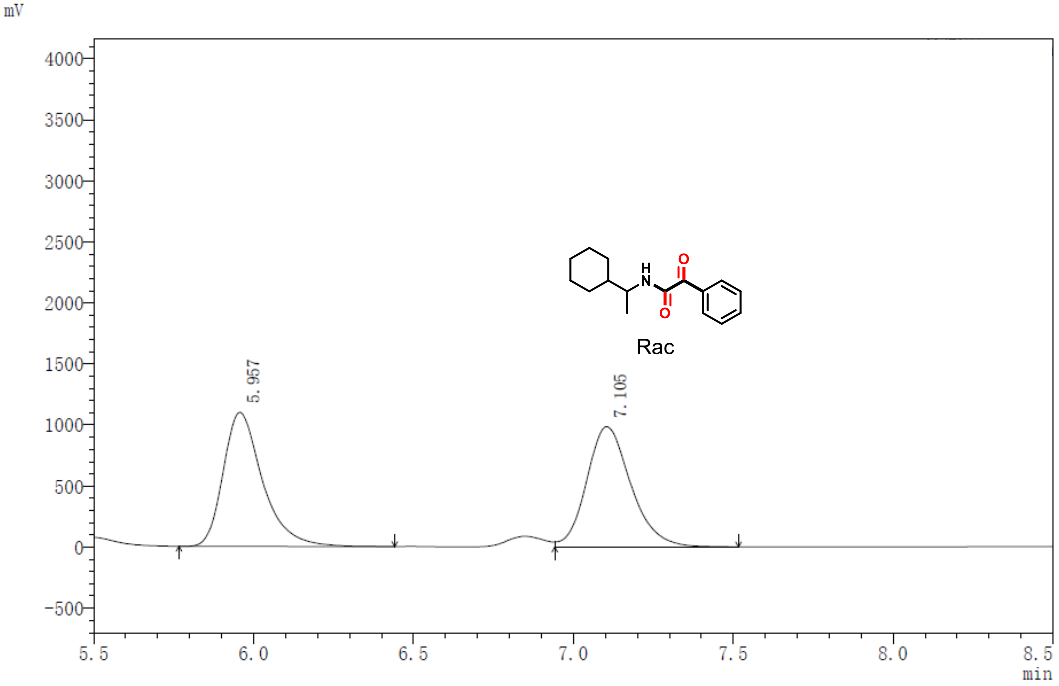

	Time	Area	Height	Area %
1	5.957	9422736	1100196	49.81
2	7.105	9494210	985185	50.19

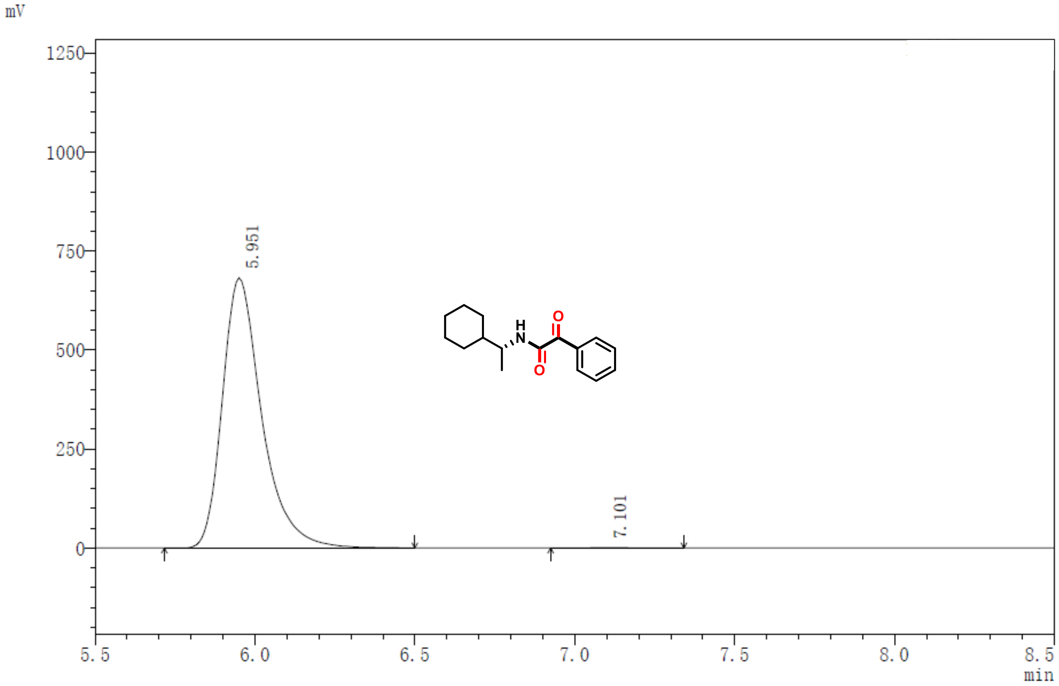

	Time	Area	Height	Area %
1	5.951	5848831	682052	99.84
2	7.101	9274	920	0.16

***(R)*-*N*-methyl-2-oxo-2-phenyl-*N*-(1-phenylethyl)acetamide (4bf)**

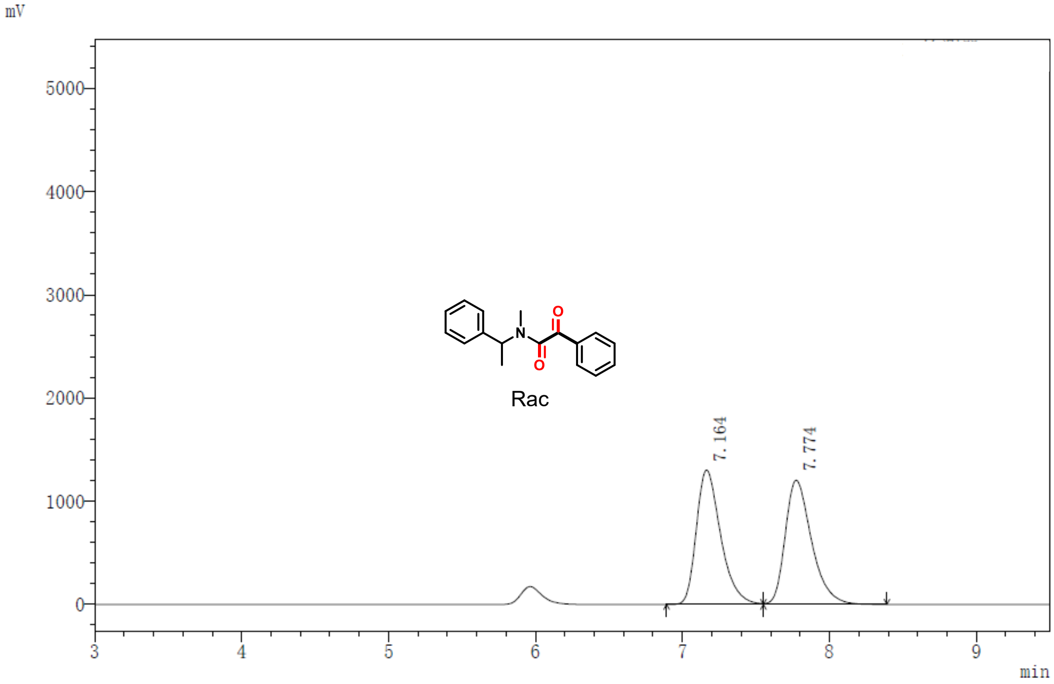

	Time	Area	Height	Area %
1	7.164	15021087	1302903	49.92
2	7.774	15072104	1204032	50.08

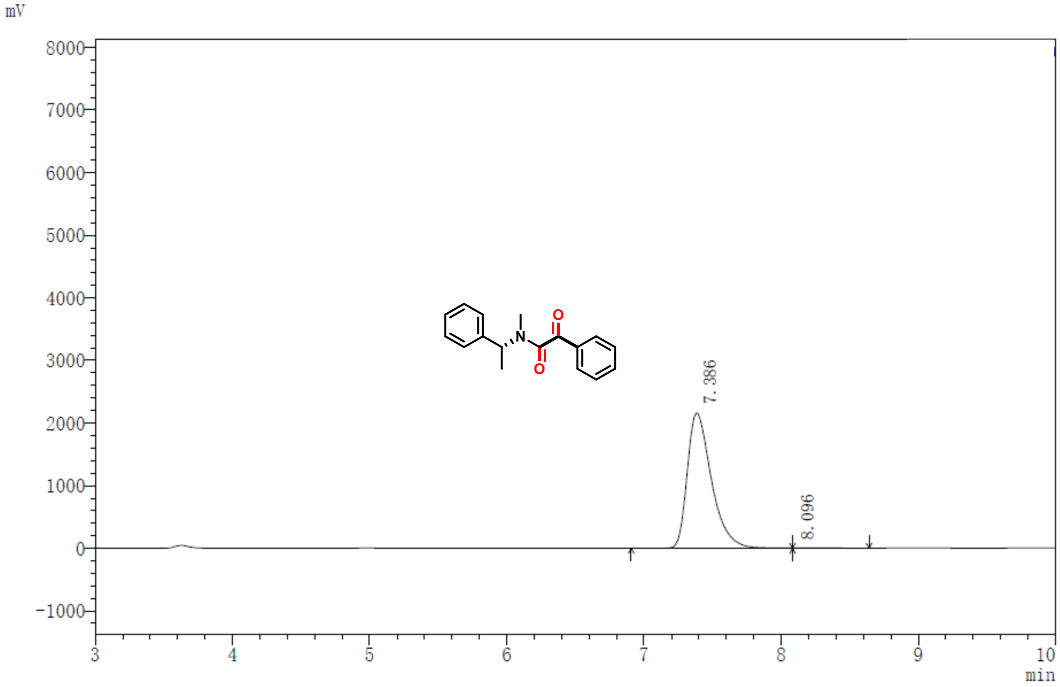

	Time	Area	Height	Area %
1	7.386	26390713	2160073	99.87
2	8.096	34147	1525	0.13

***(S)-N*-(hexan-2-yl)-2-oxo-2-phenylacetamide (4bg)**

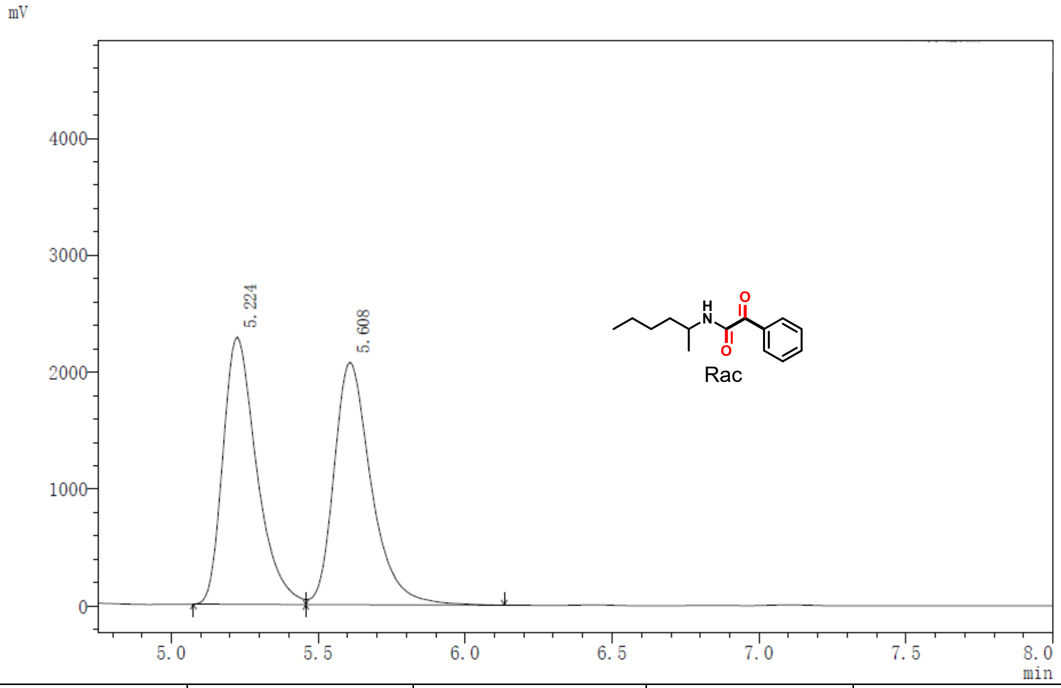

	Time	Area	Height	Area %
1	5.224	18241403	2285642	49.94
2	5.608	18285769	2075158	50.06

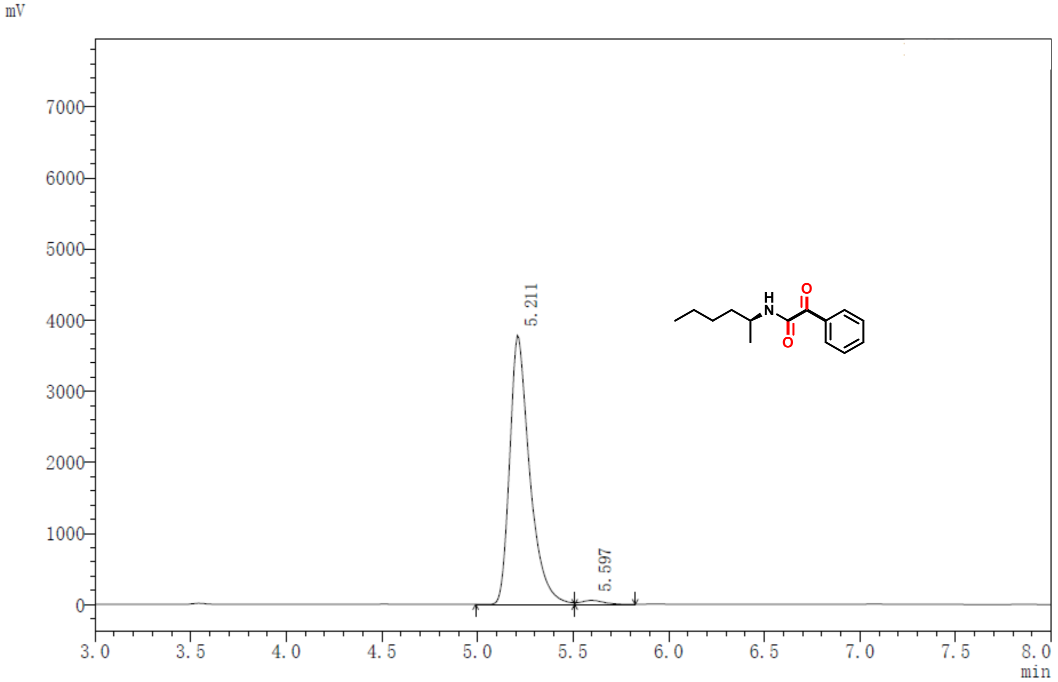

	Time	Area	Height	Area %
1	5.211	27531258	3781170	98.33
2	5.597	468380	57338	1.67

***(R)-N*-(1-(naphthalen-1-yl)ethyl)-2-oxo-2-phenylacetamide (4bi)**

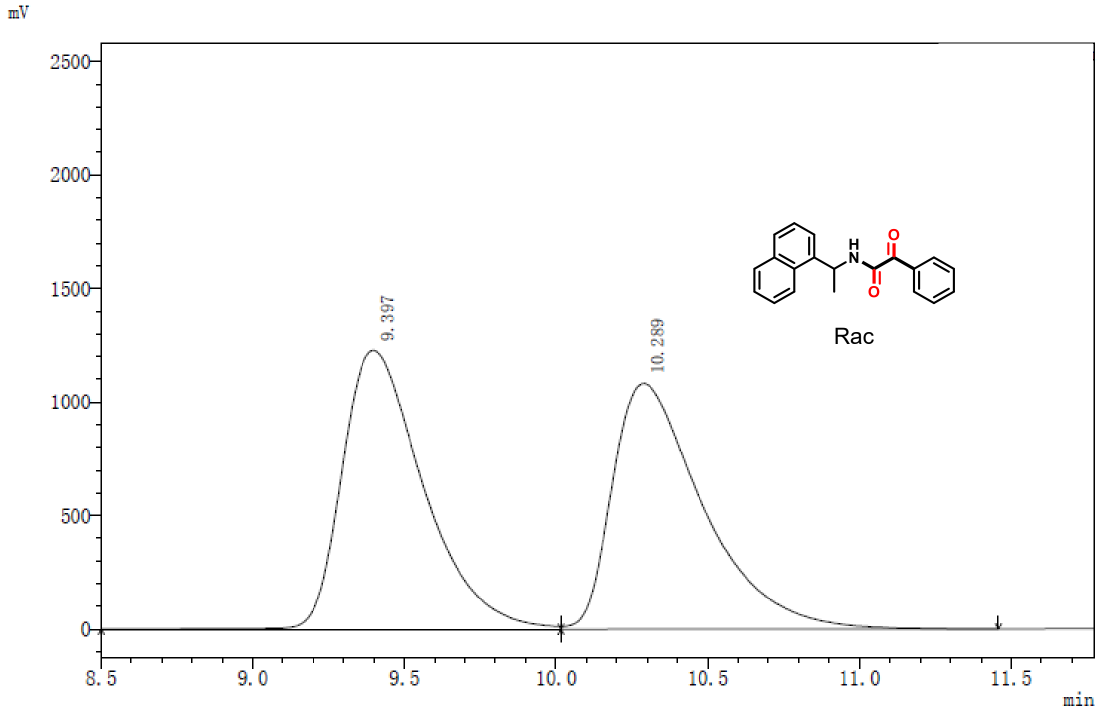

	Time	Area	Height	Area %
1	9.397	22859291	1228504	50.35
2	10.289	22538619	1081351	49.65

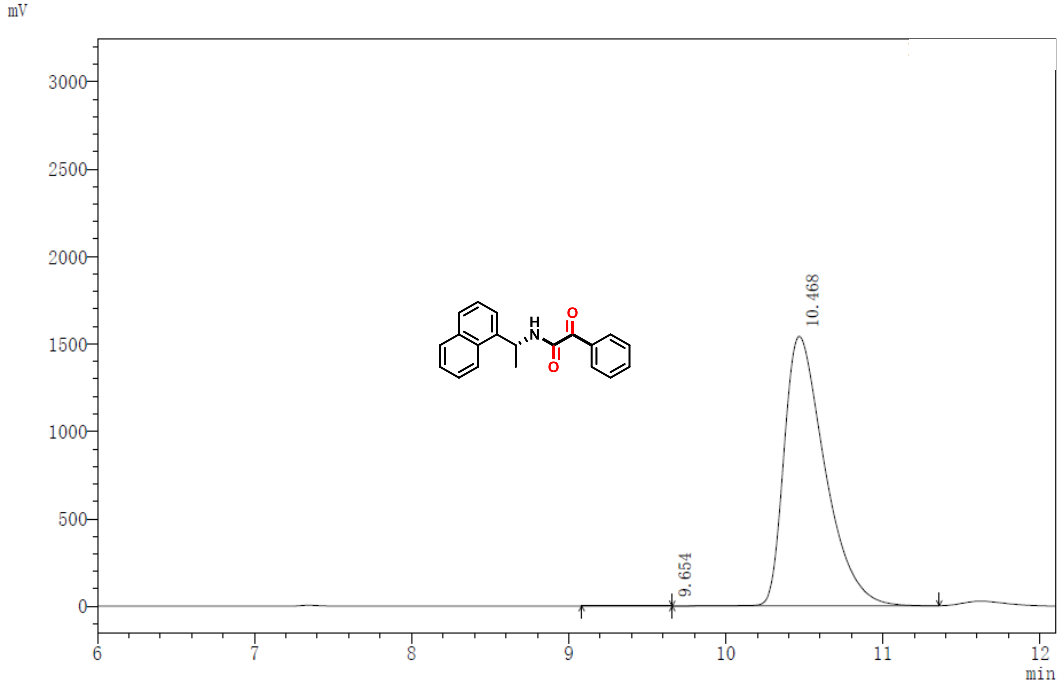

	Time	Area	Height	Area %
1	9.654	2367	286	0.01
2	10.468	27697820	1541013	99.99

NMR spectra

3a Morpholino(phenyl)methanone

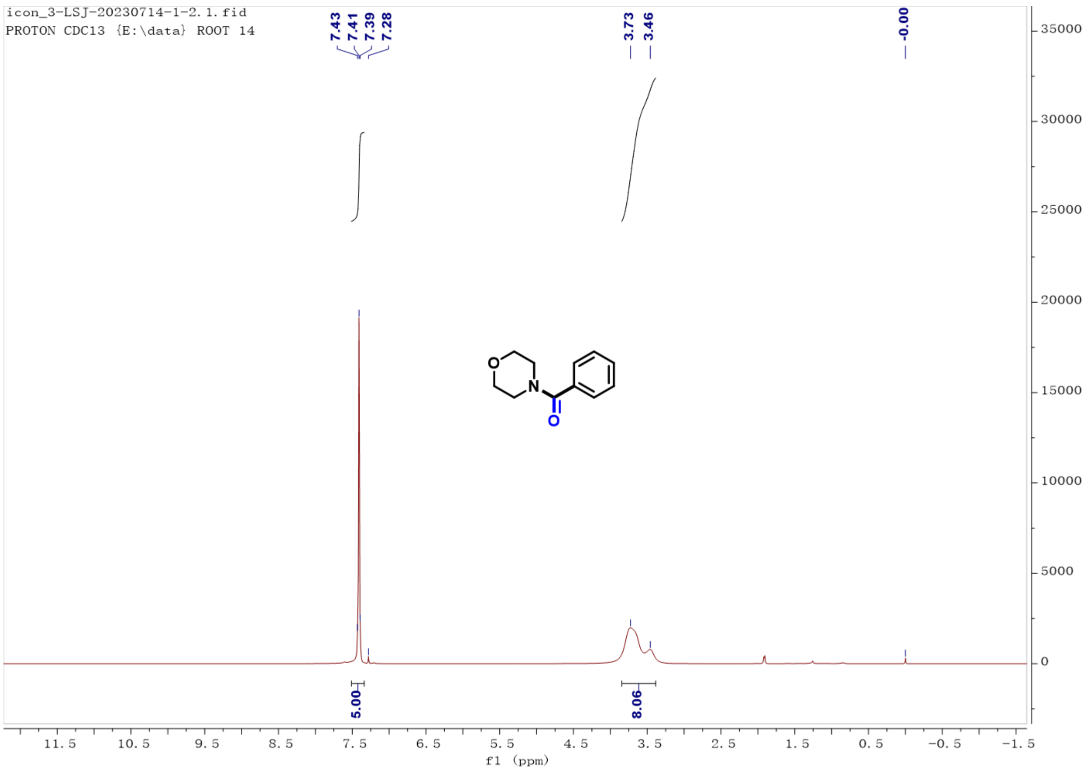

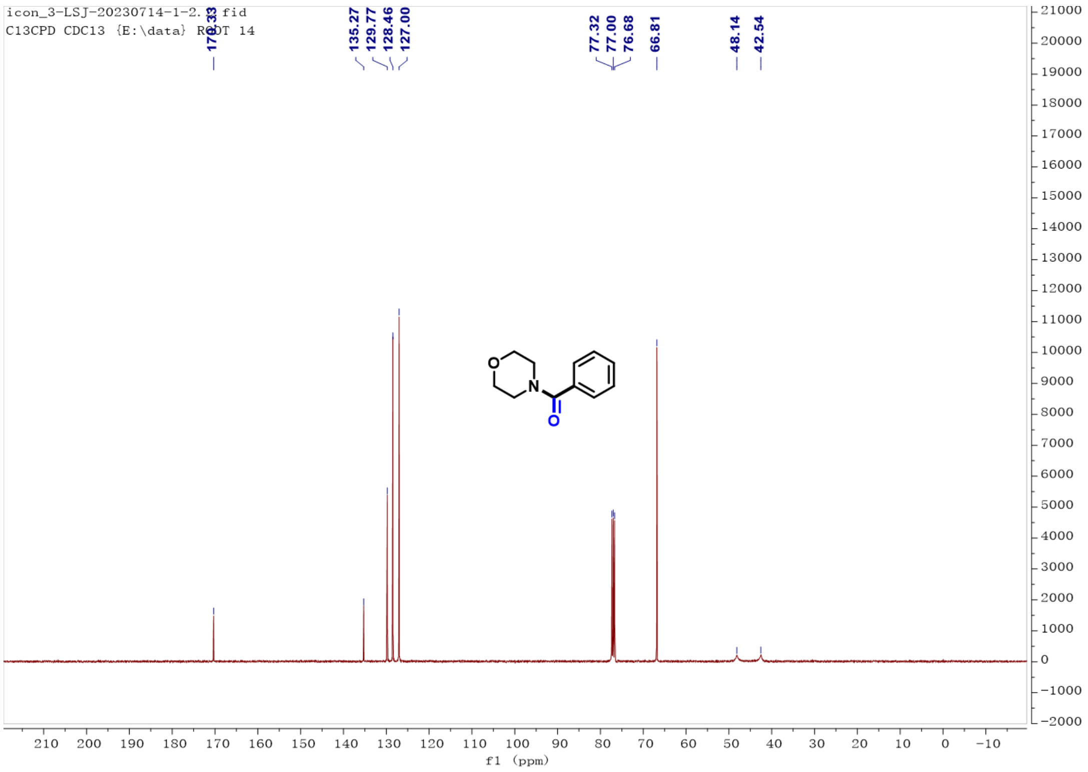

3b (4-methylpiperidin-1-yl)(phenyl)methanone

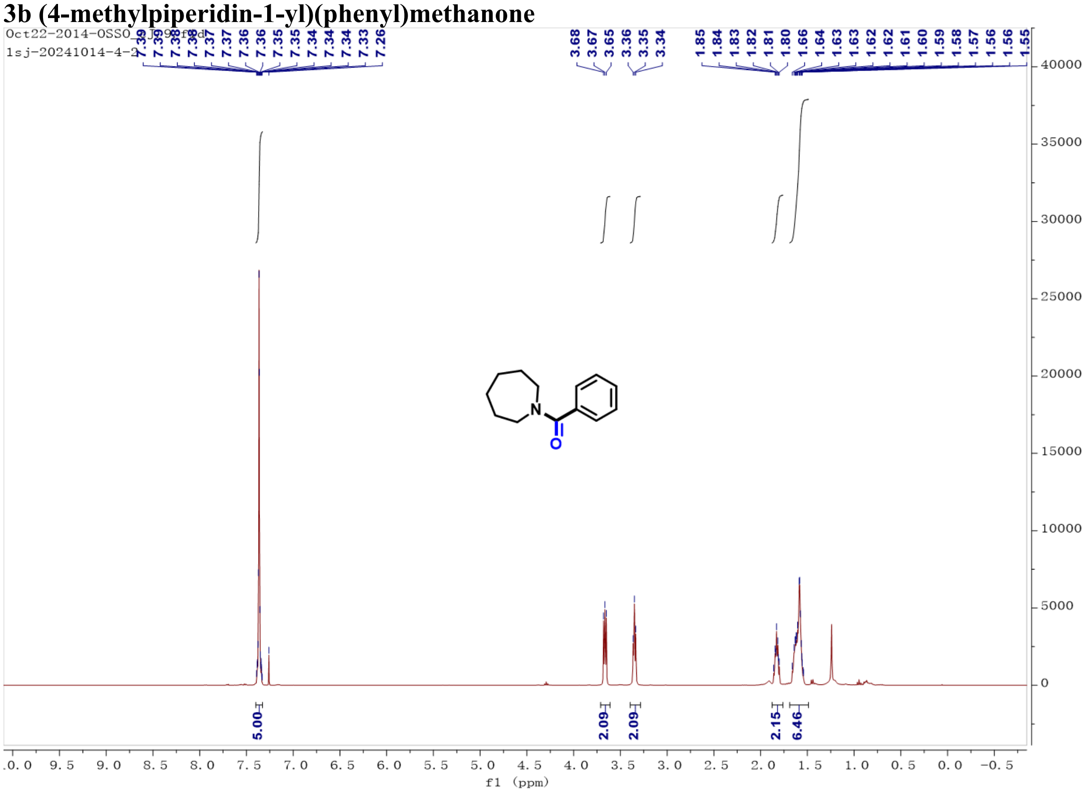

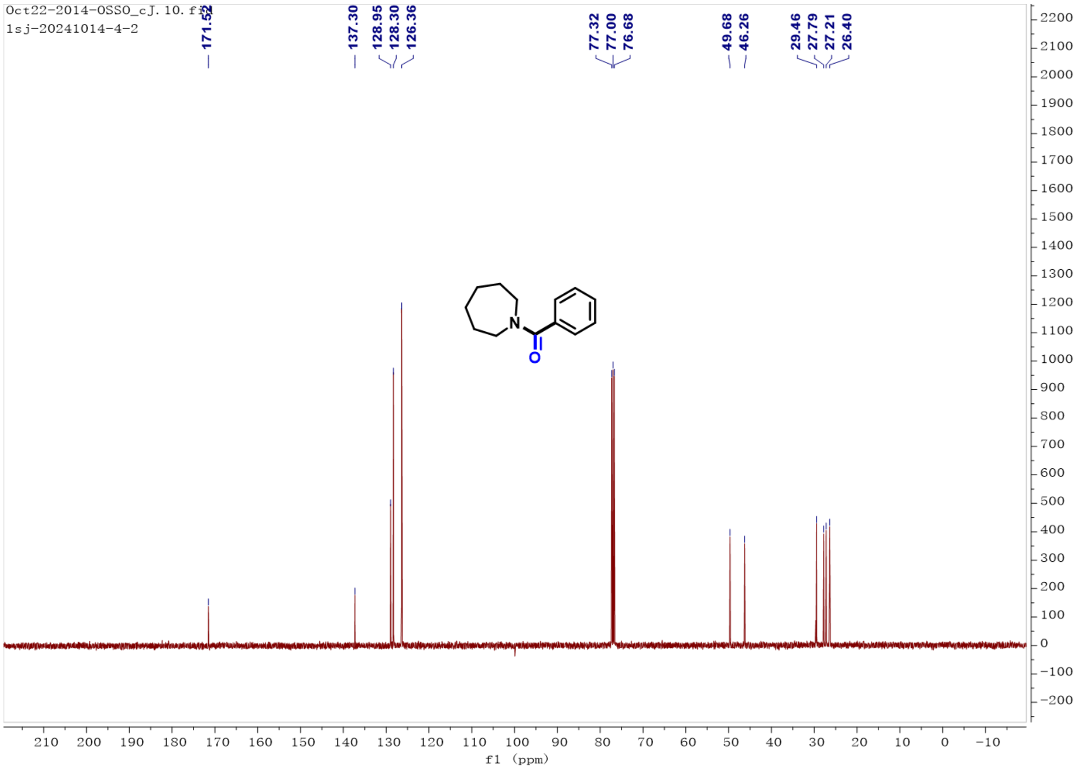

3c phenyl(pyrrolidin-1-yl)methanone

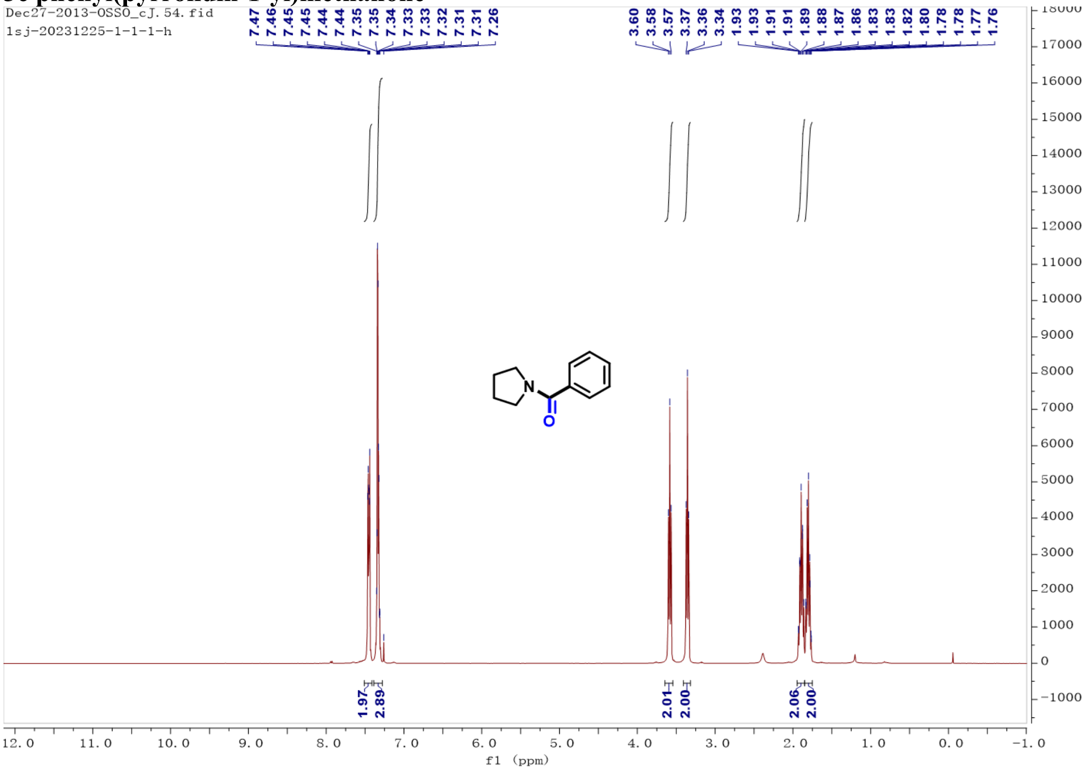

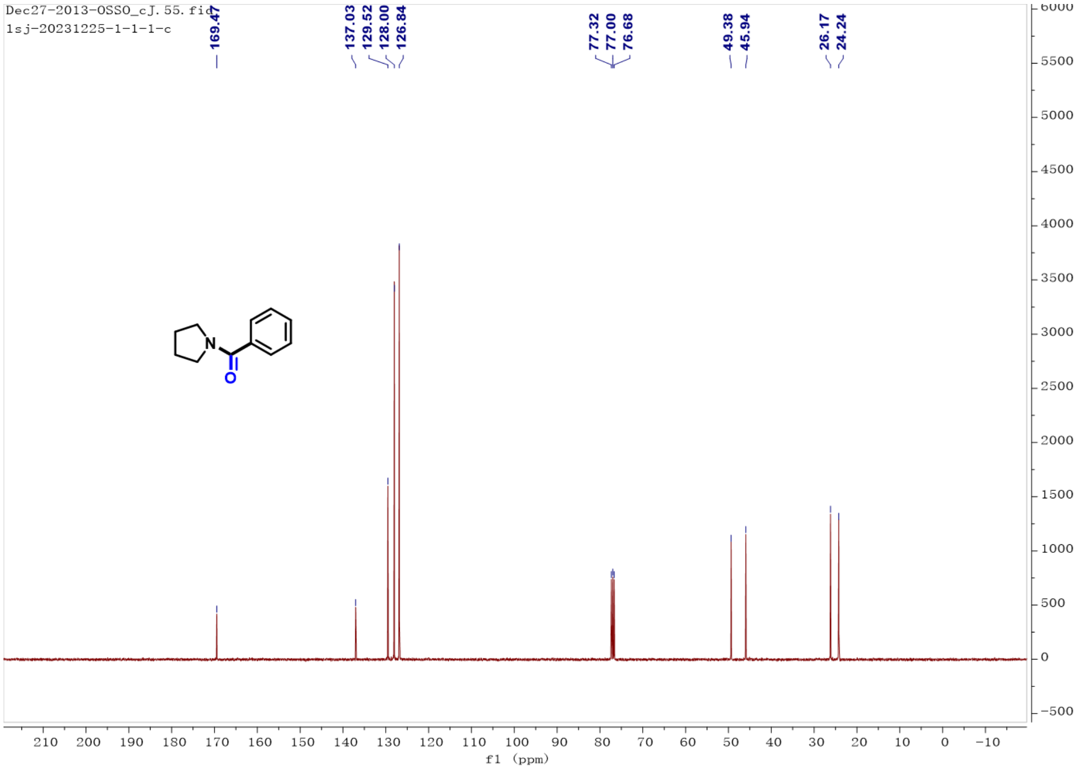

3d phenyl(piperidin-1-yl)methanone

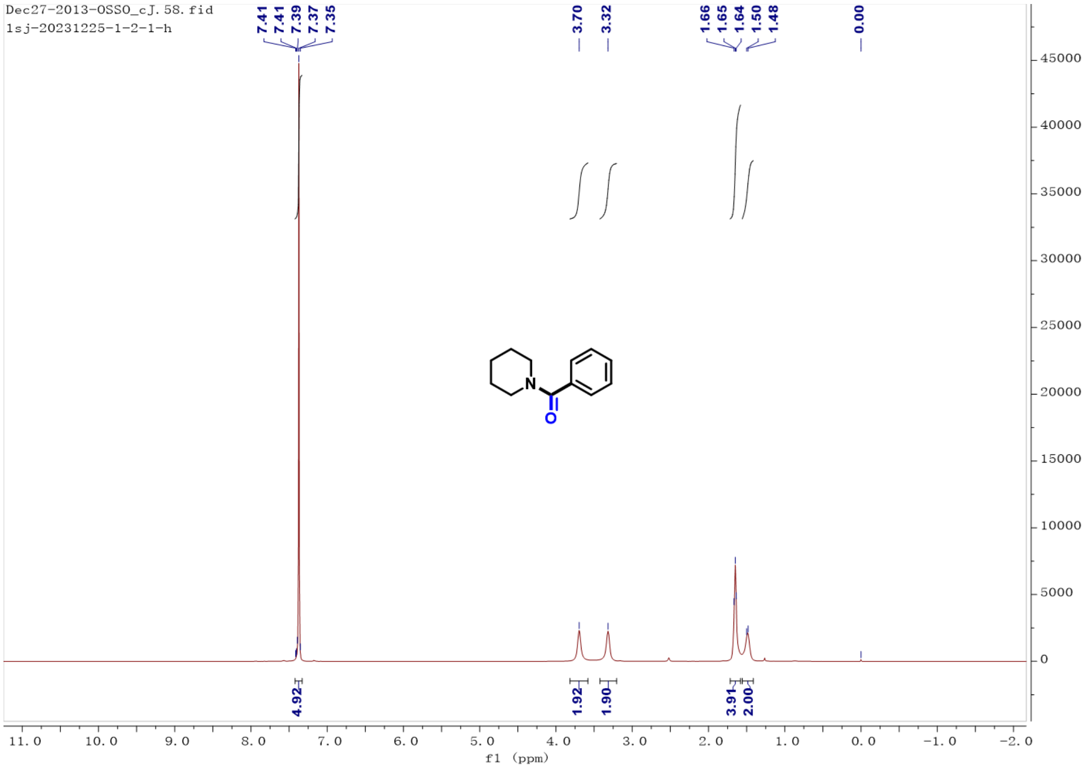

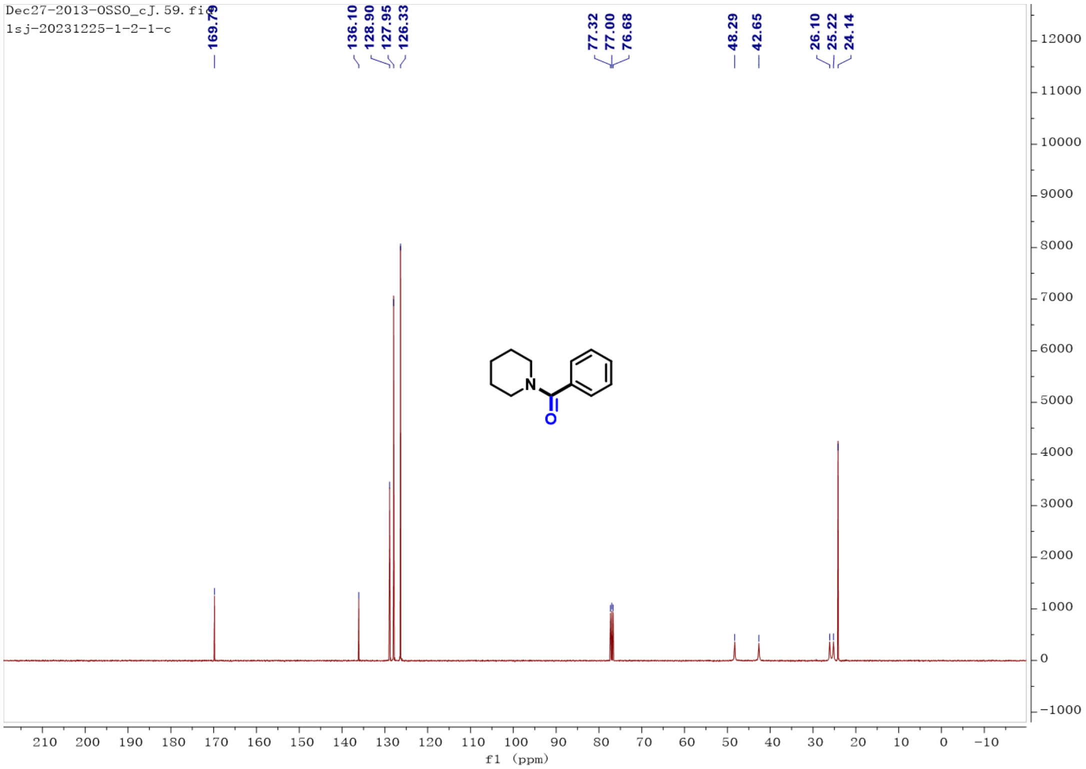

3e (4-methylpiperidin-1-yl)(phenyl)methanone

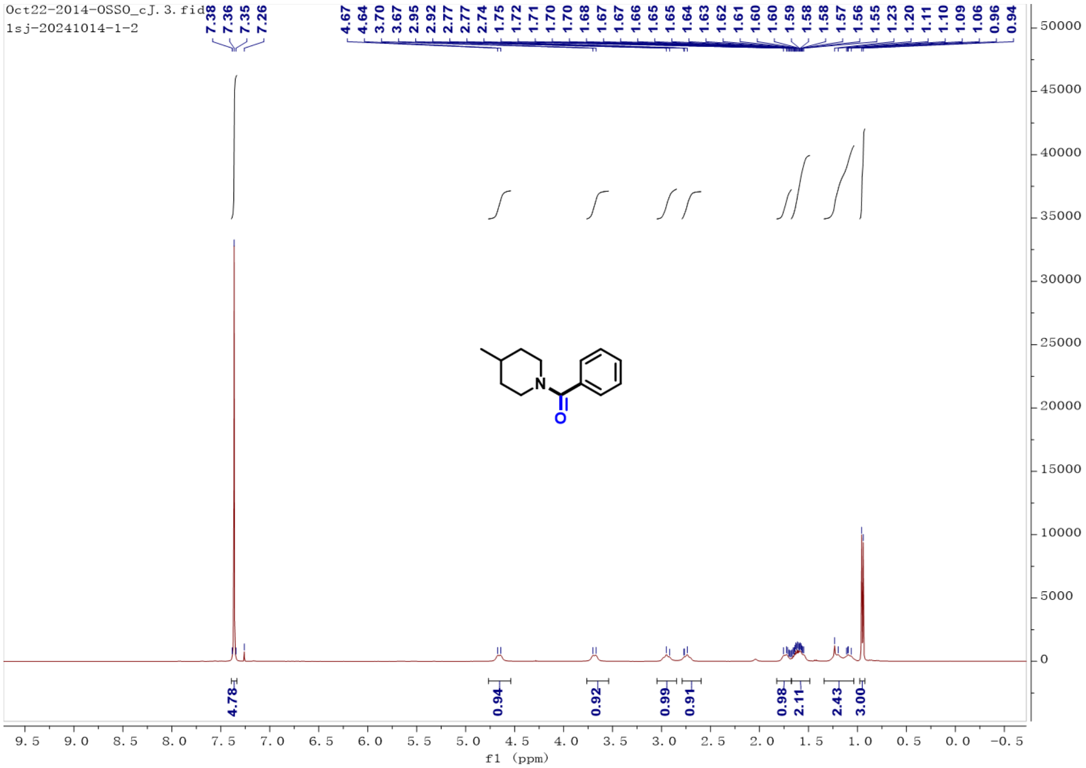

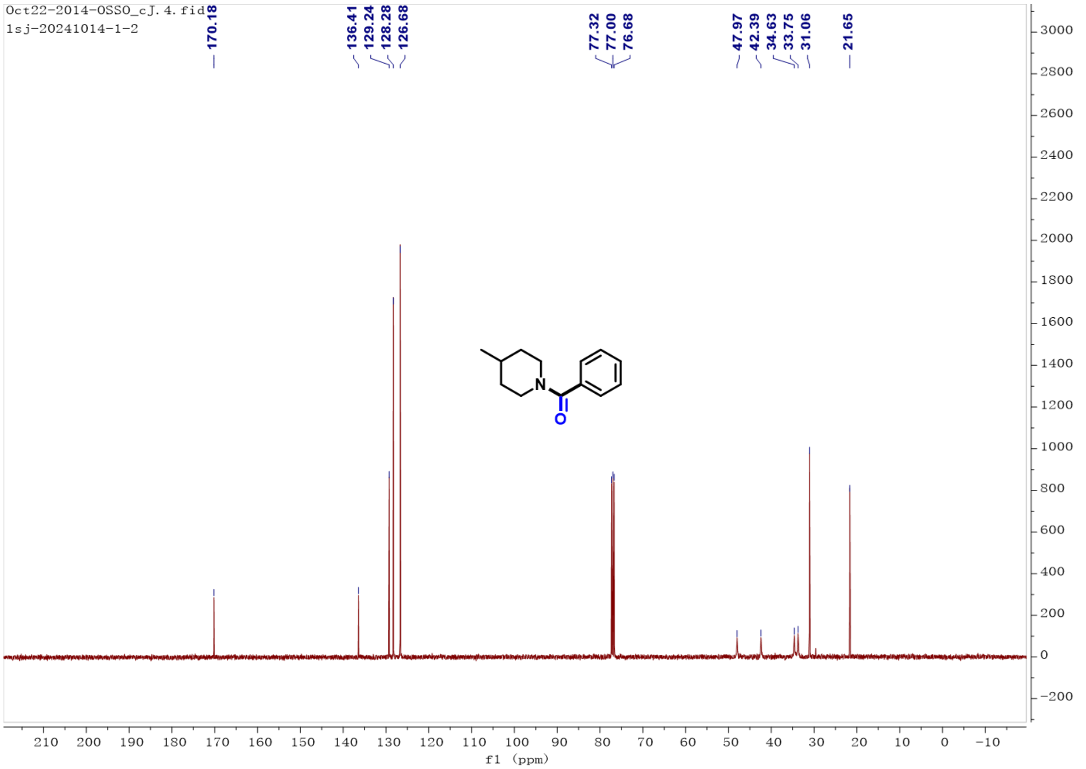

3f methyl 1-benzoylpiperidine-4-carboxylate

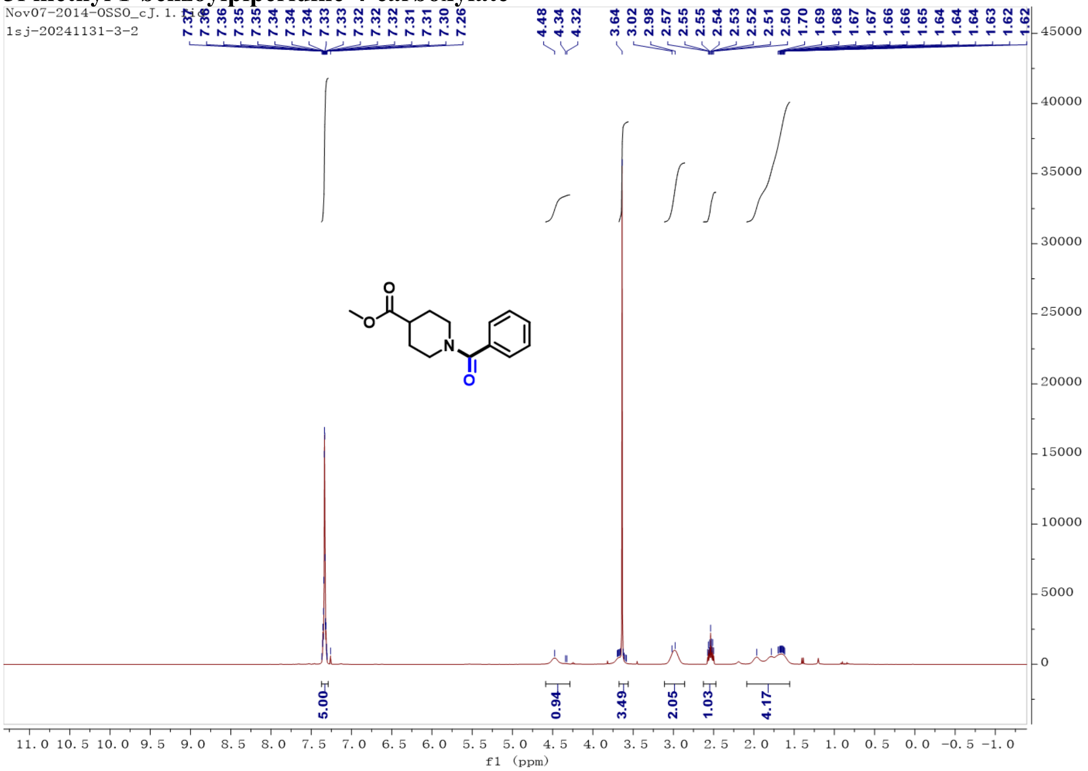

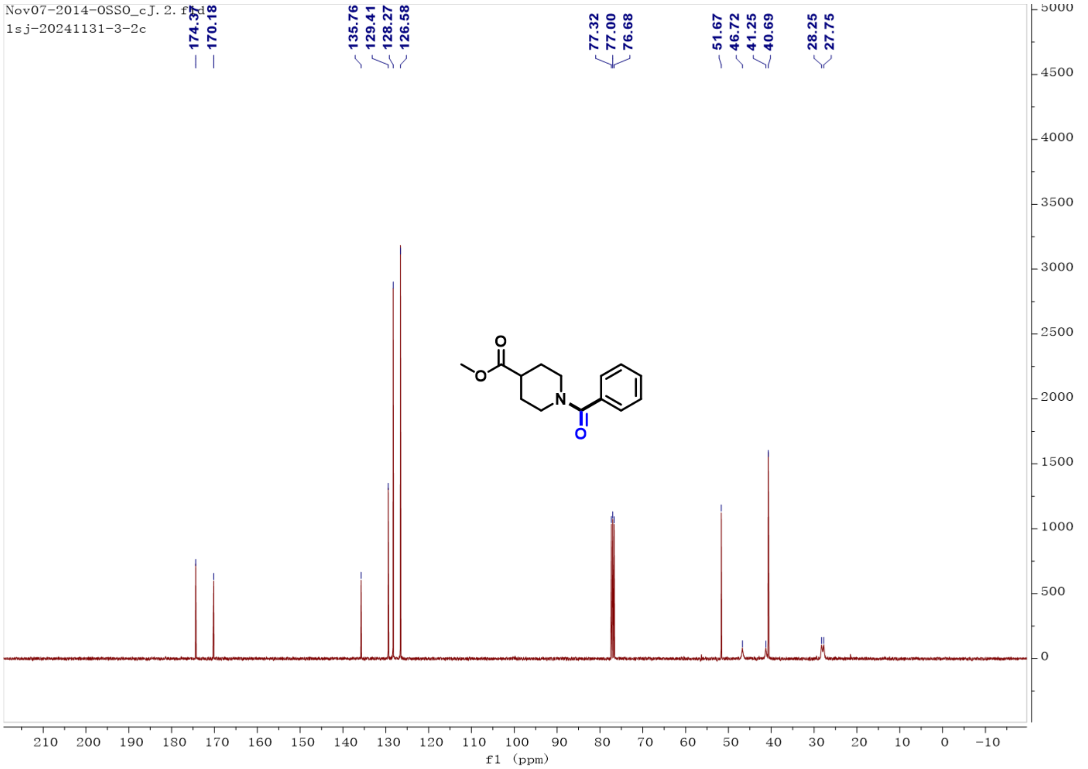

3g (3,5-dimethylpiperidin-1-yl)(phenyl)methanone

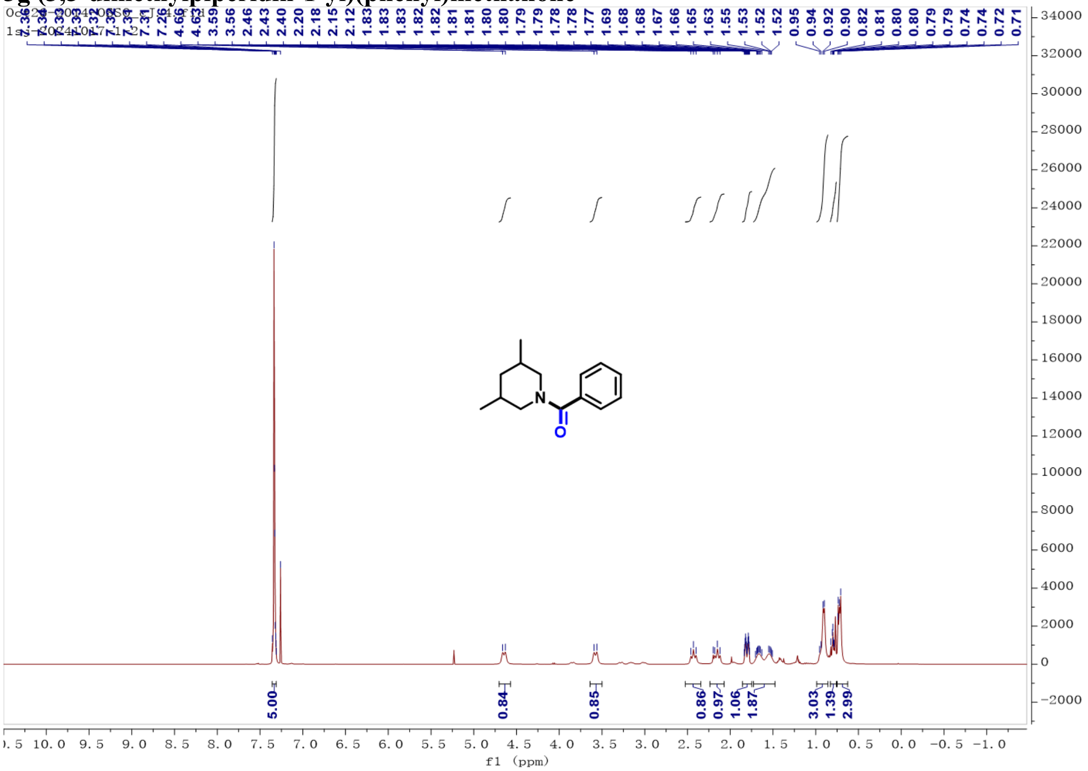

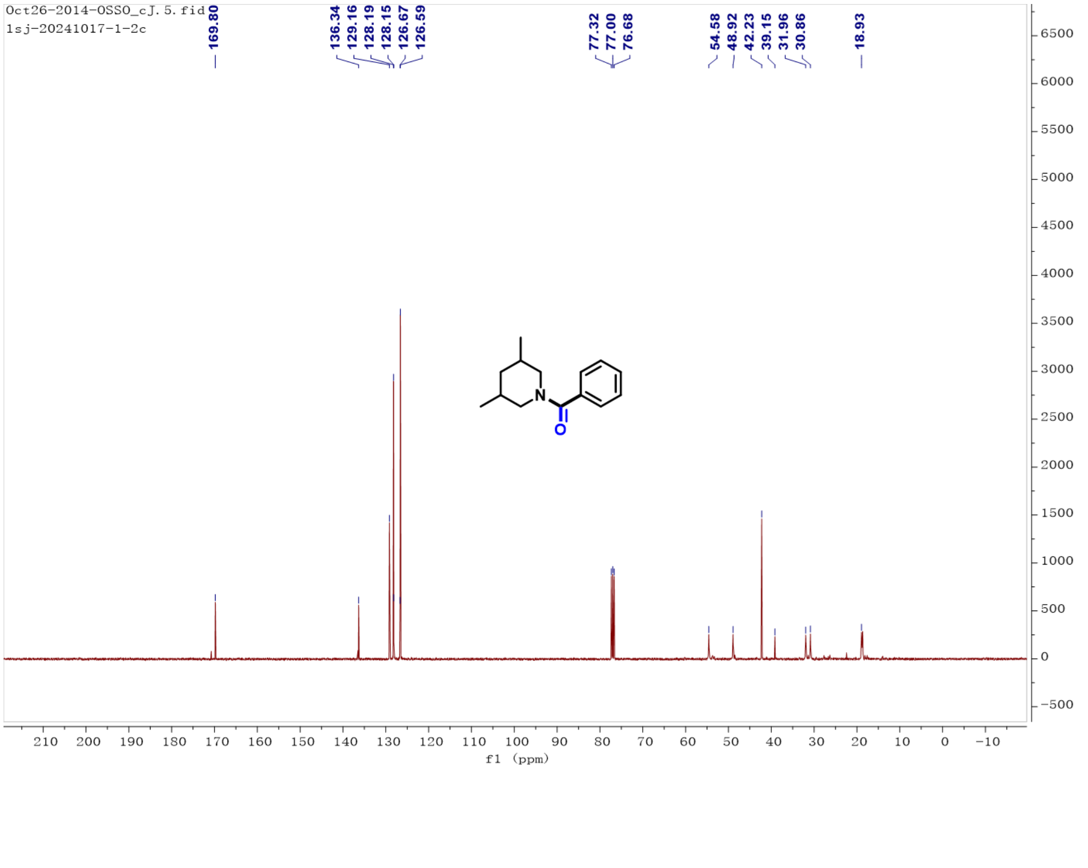

3h (3,4-dihydroisoquinolin-2(1*H*)-yl)(phenyl)methanone

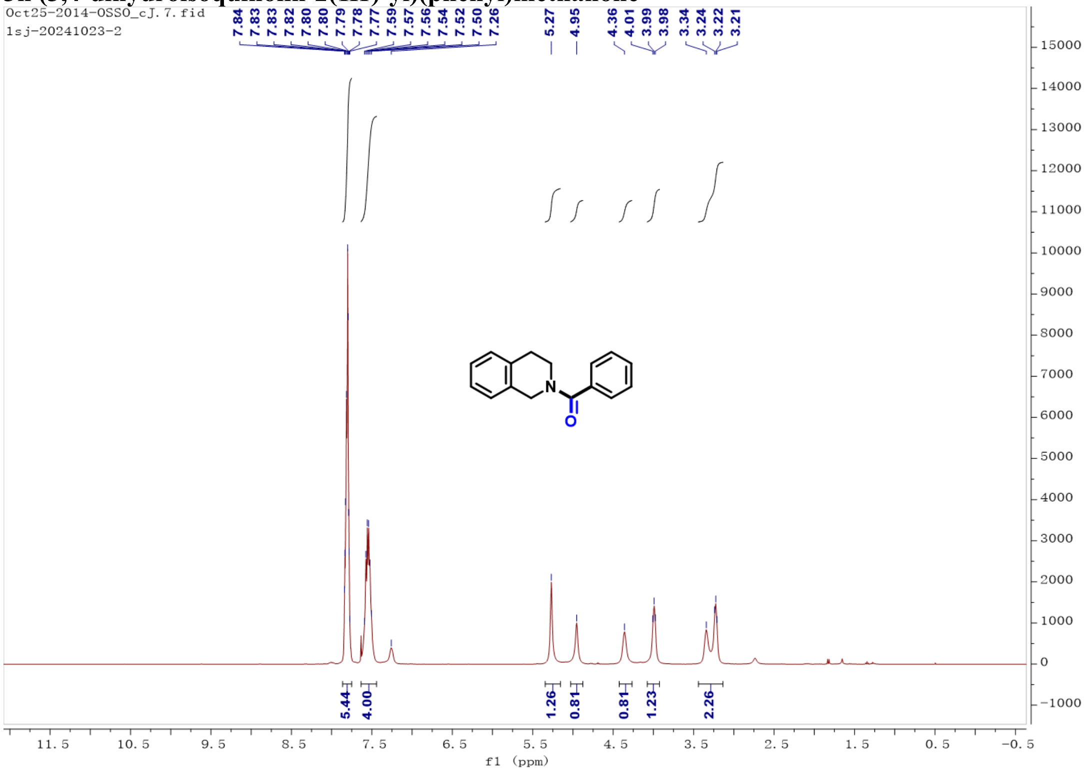

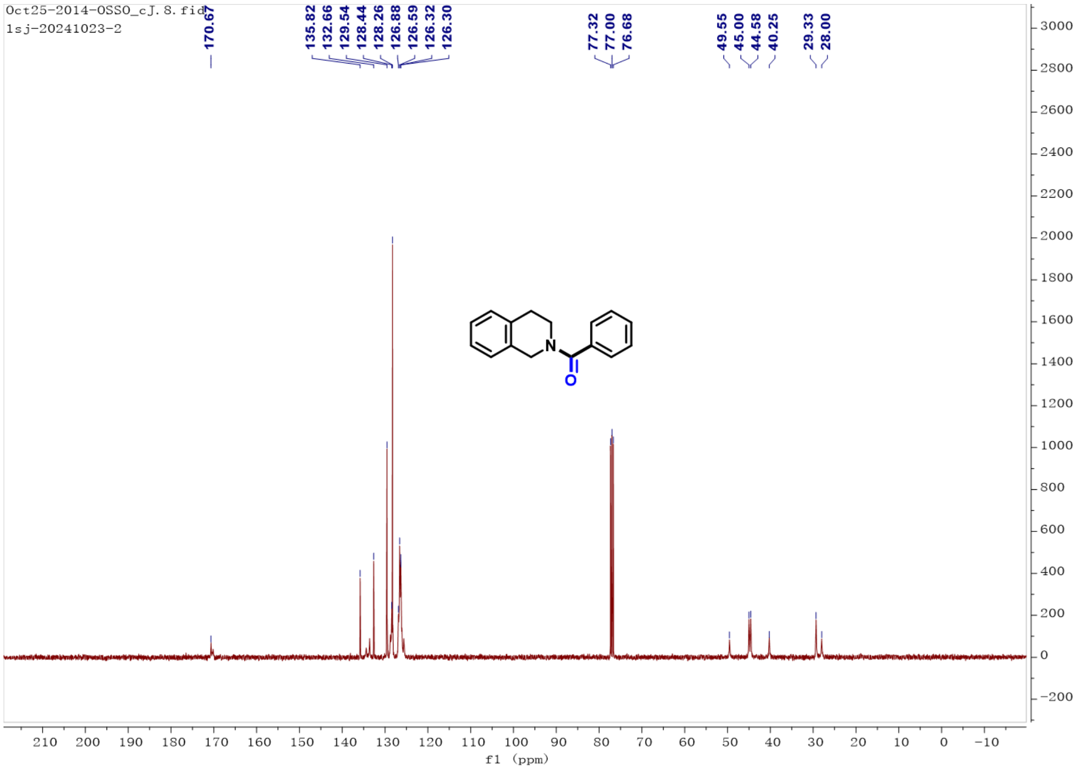

3i (4-ethylpiperazin-1-yl)(phenyl)methanone

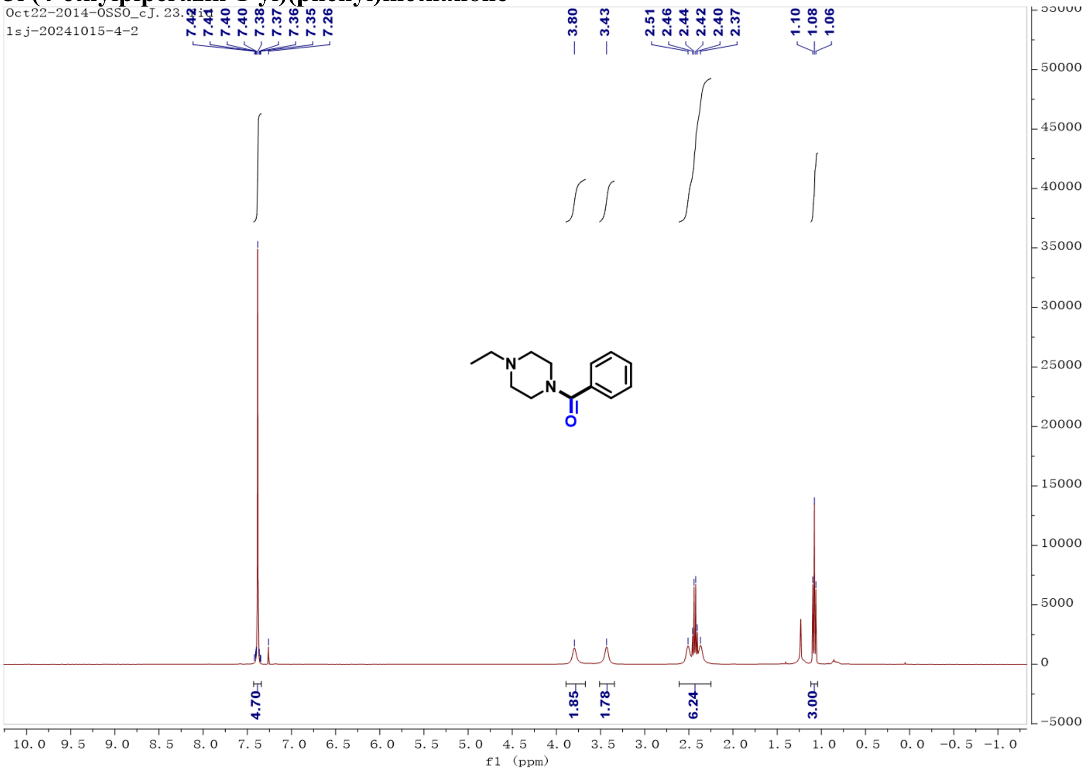

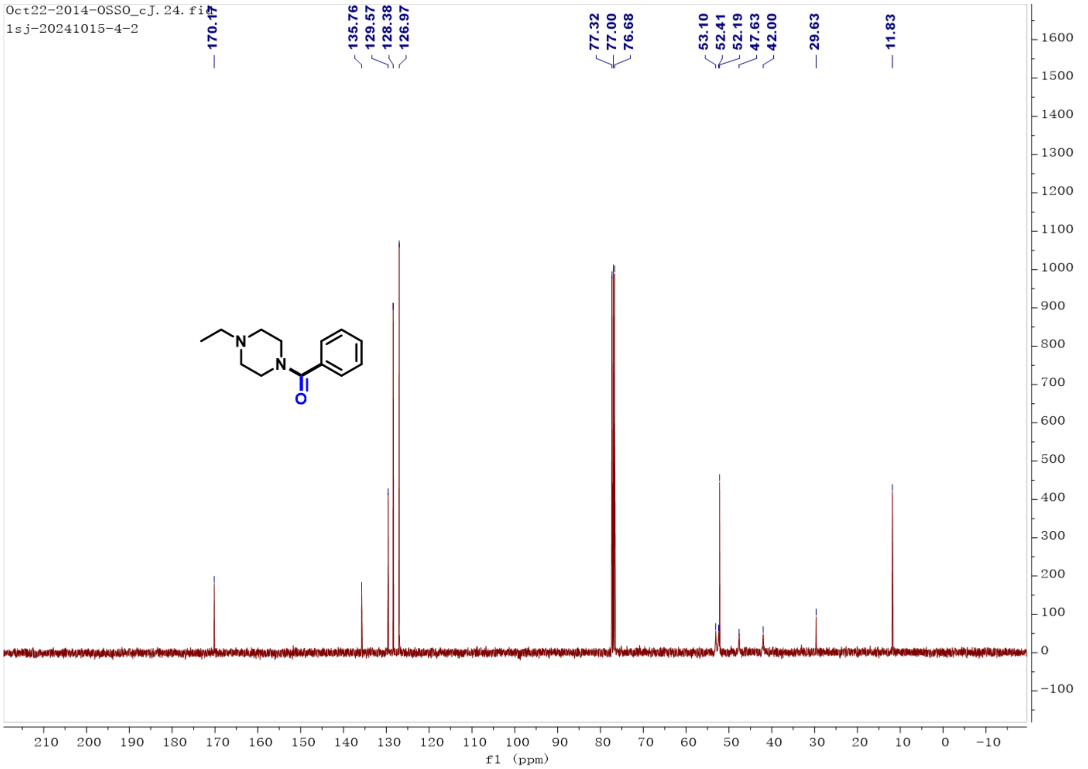

3j phenyl(4-phenylpiperazin-1-yl)methanone

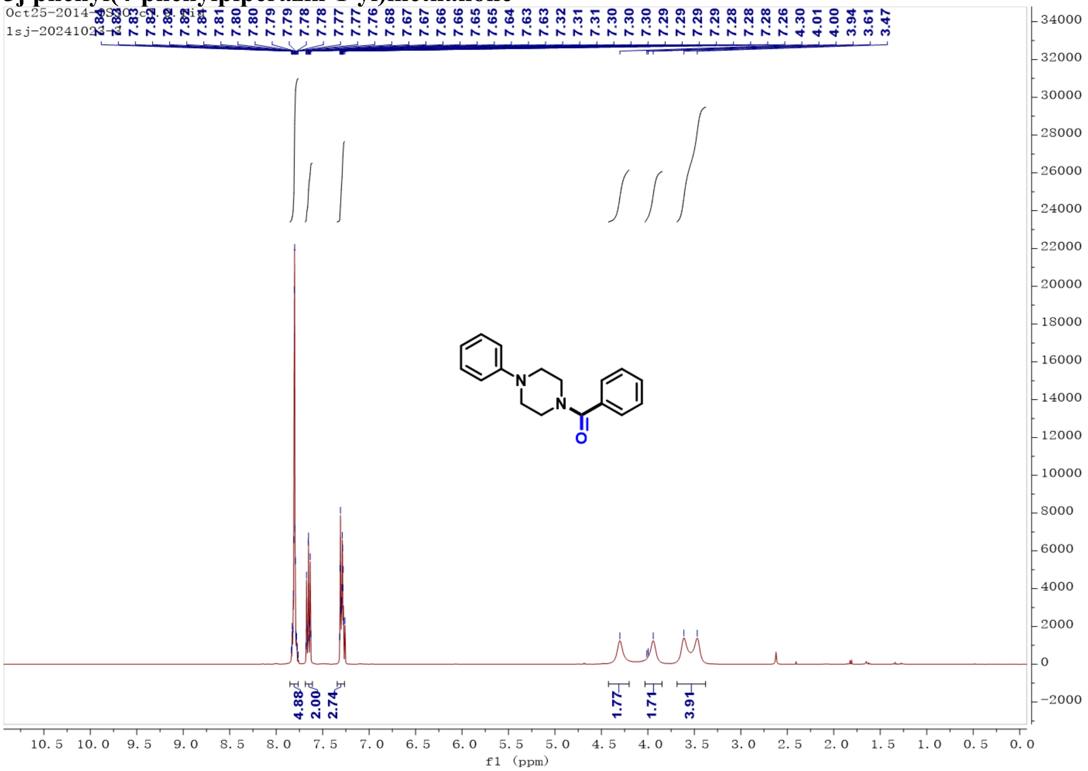

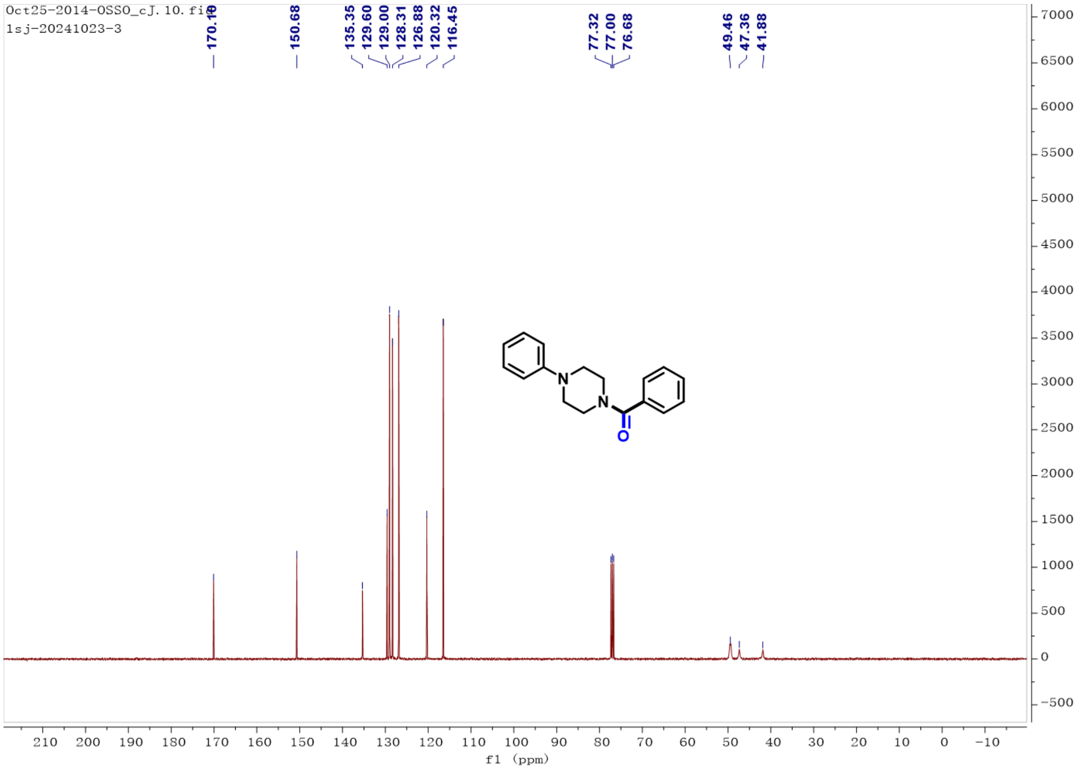

3k(4-(2-hydroxyethyl)piperazin-1-yl)(phenyl)methanone

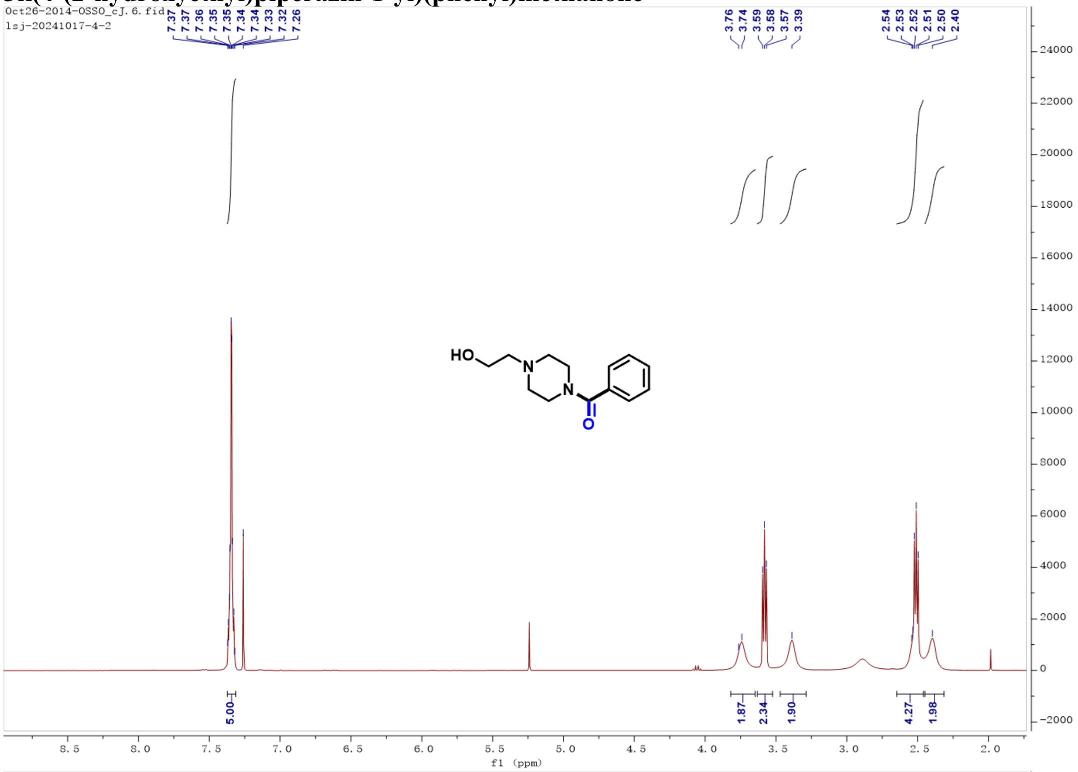

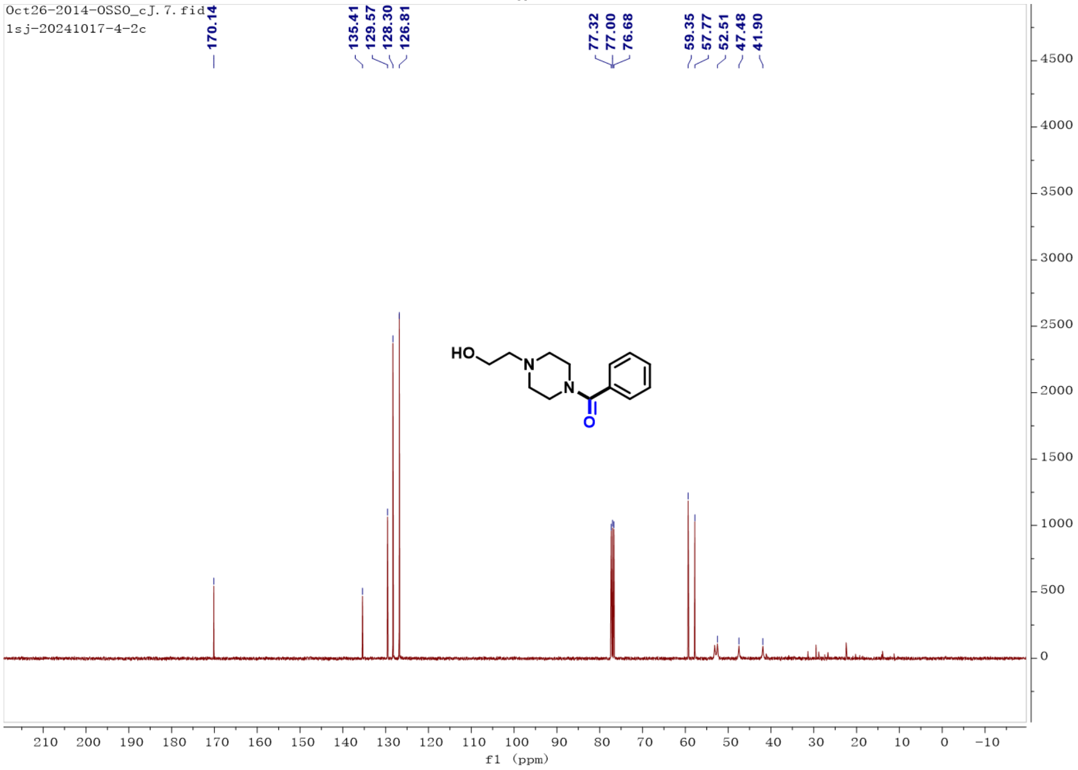

3l phenyl(4-(pyrimidin-2-yl)piperazin-1-yl)methanone

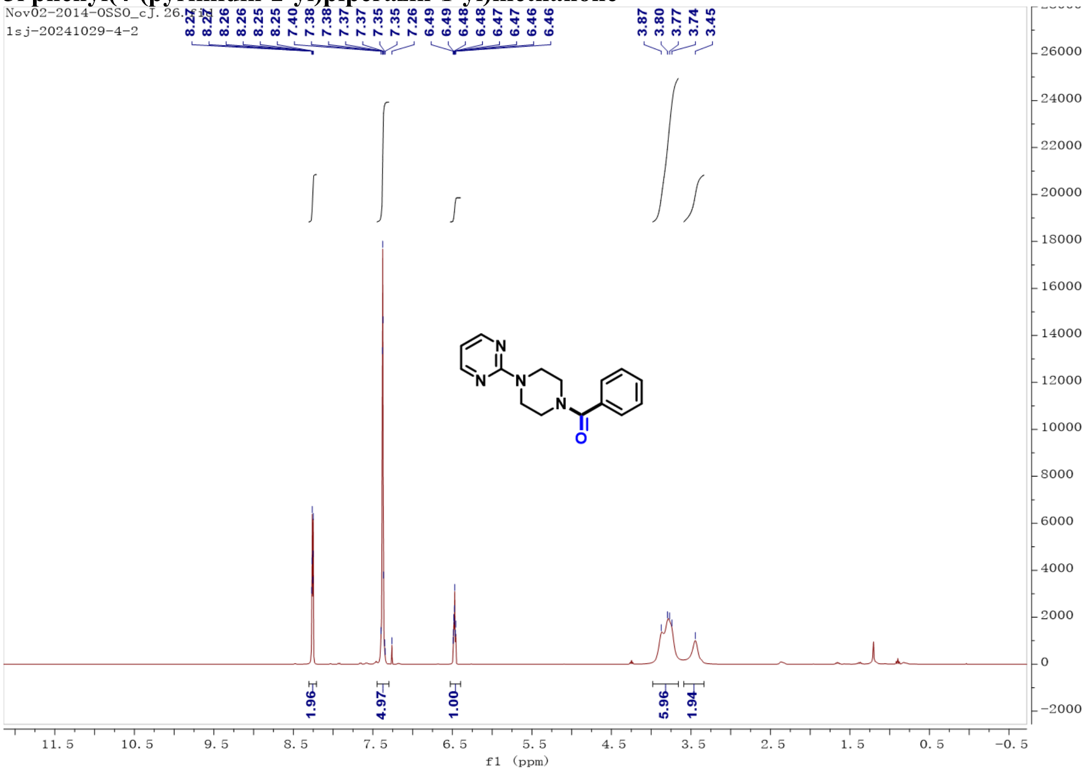

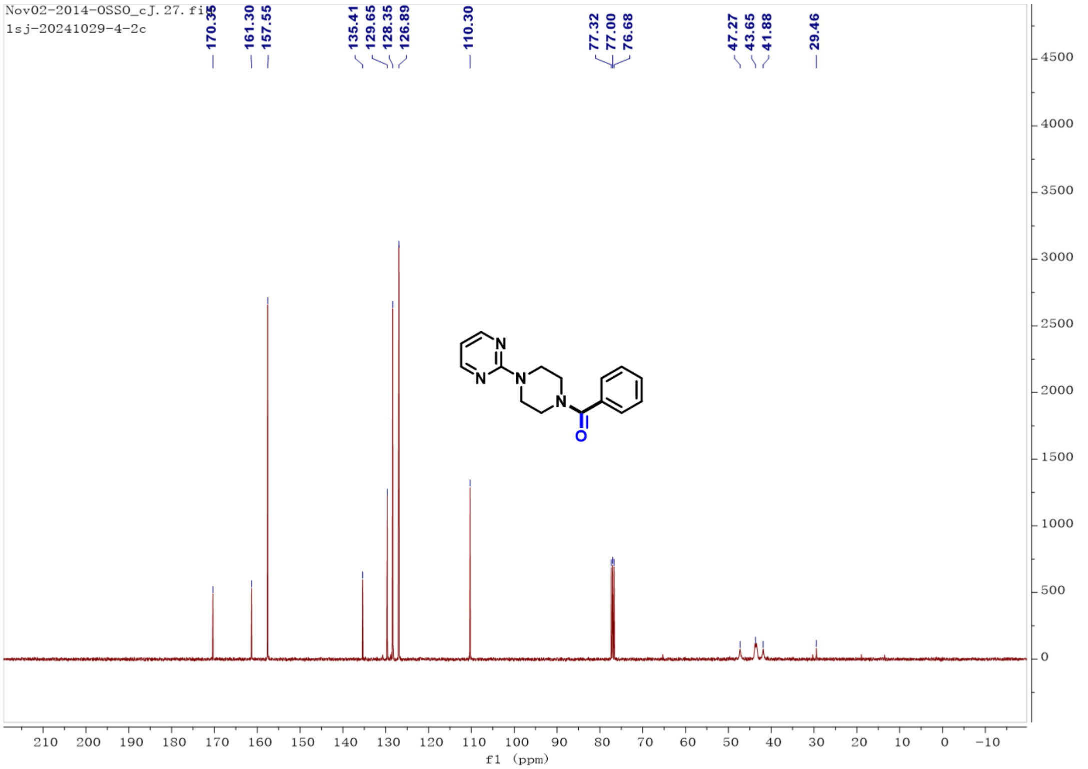

3m benzyl 4-benzoylpiperazine-1-carboxylate

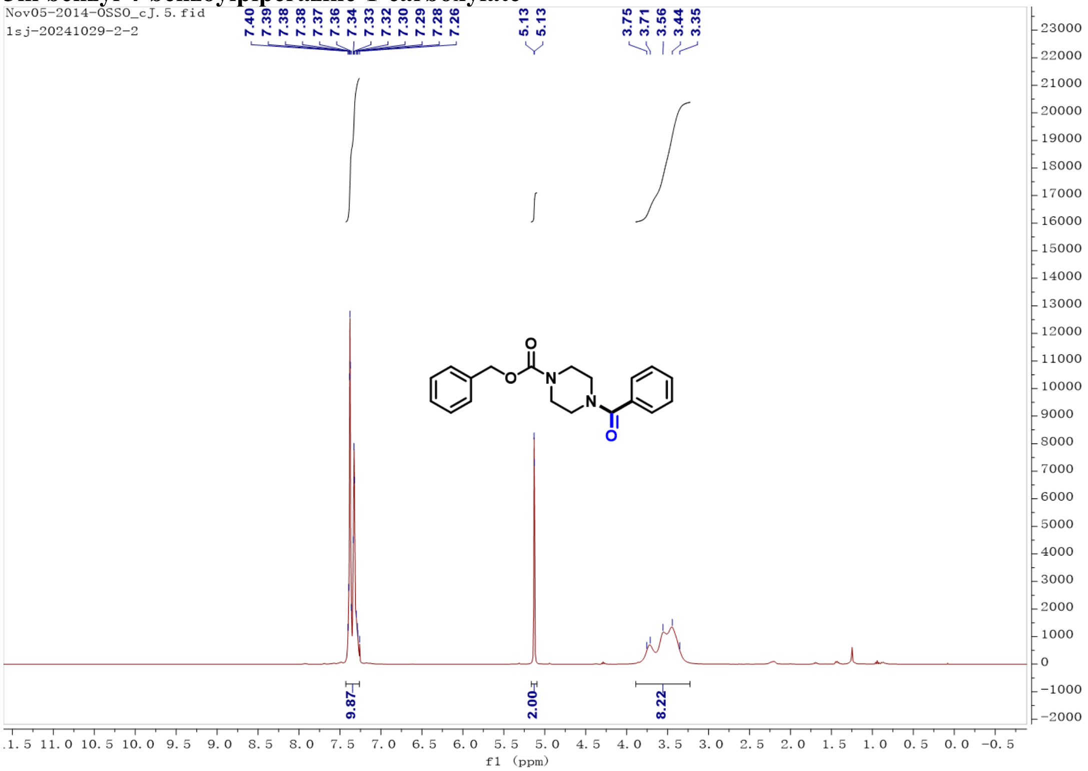

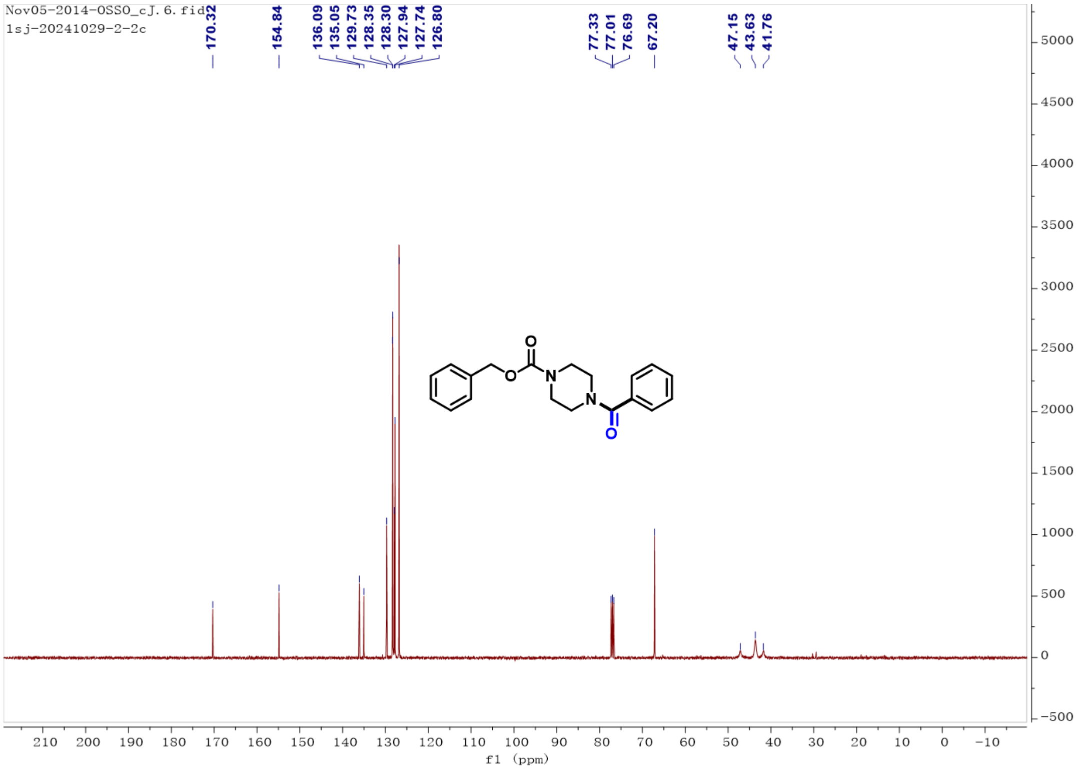

3n *N,N*-diethylbenzamide

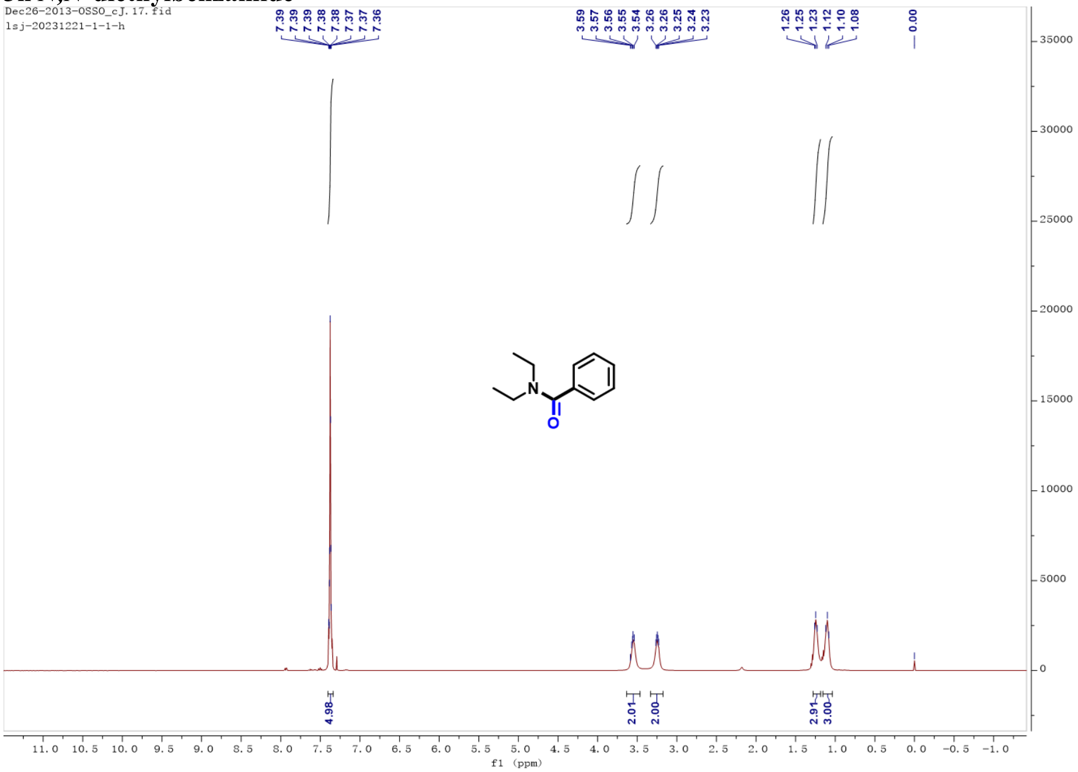

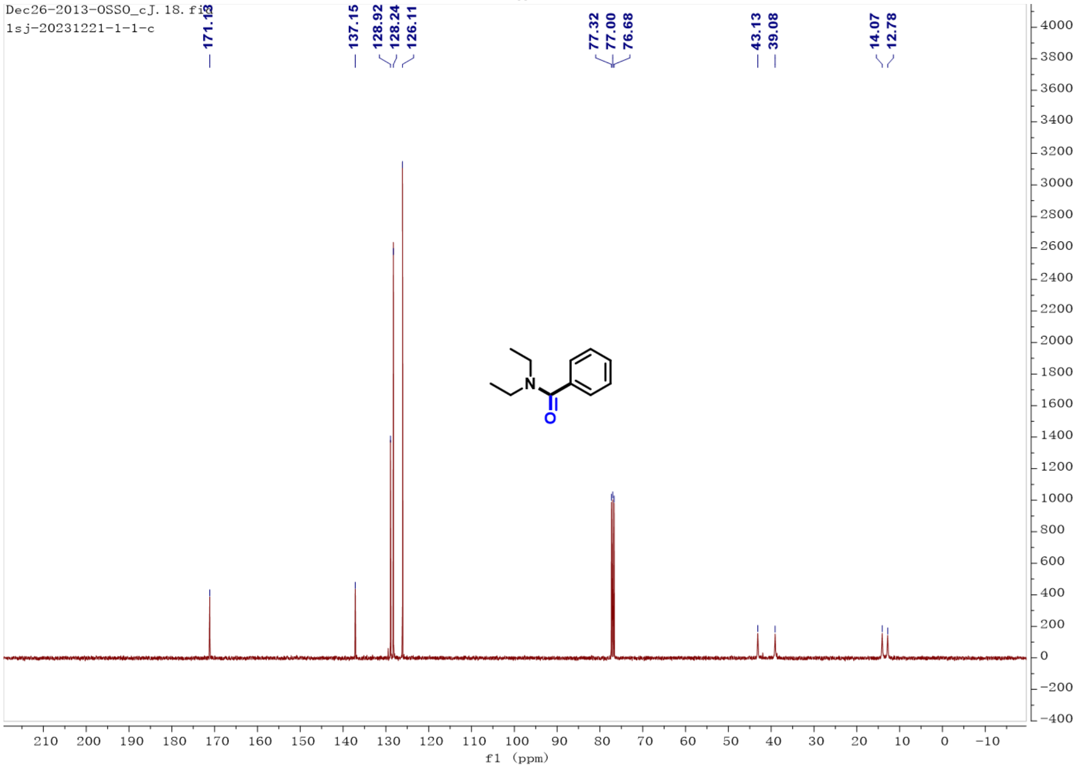

3o *N,N*-dibutylbenzamide

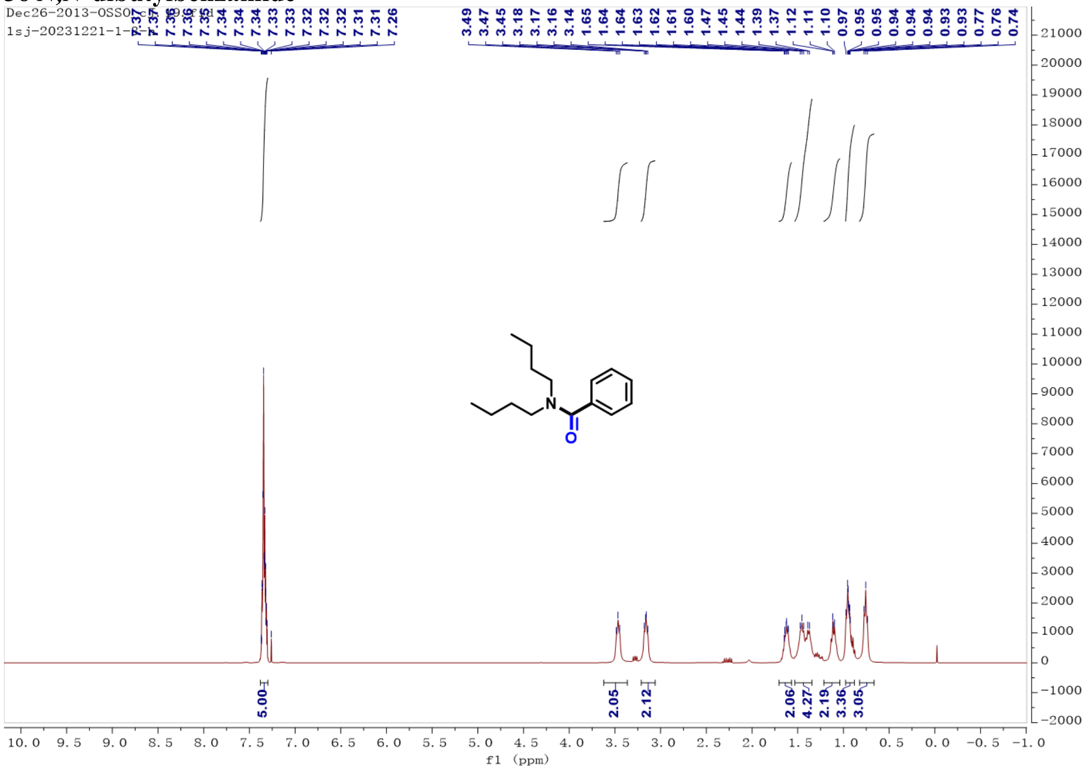

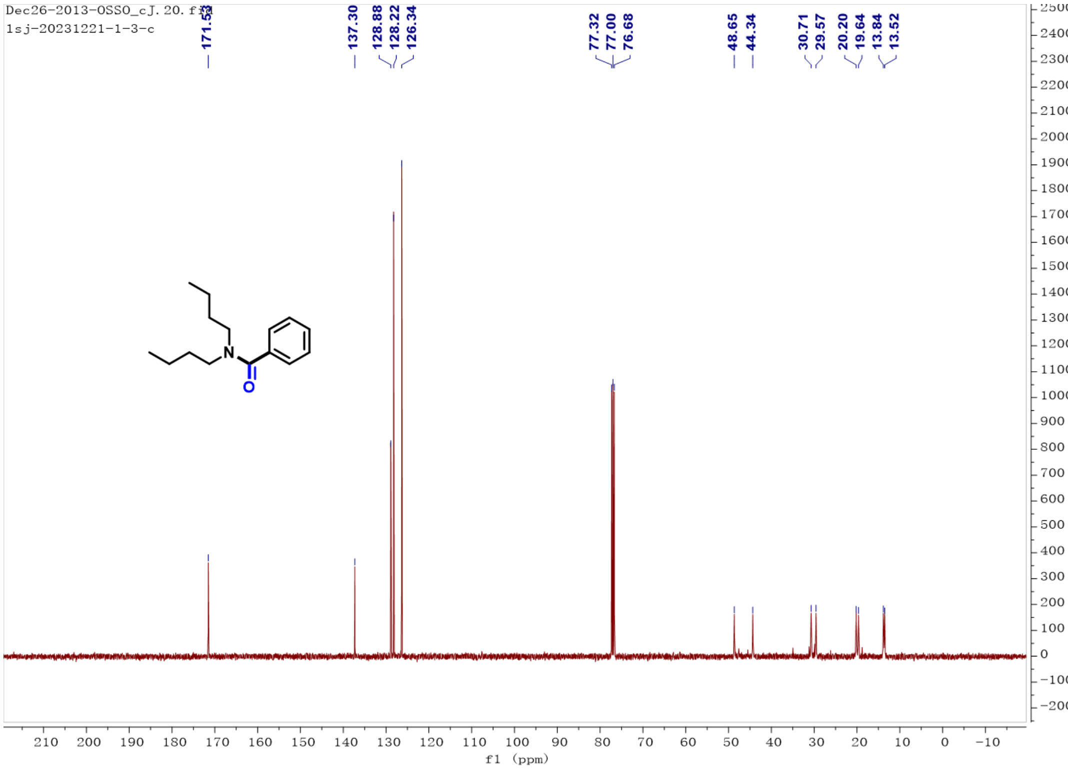

3p *N*-butyl-*N*-methylbenzamide

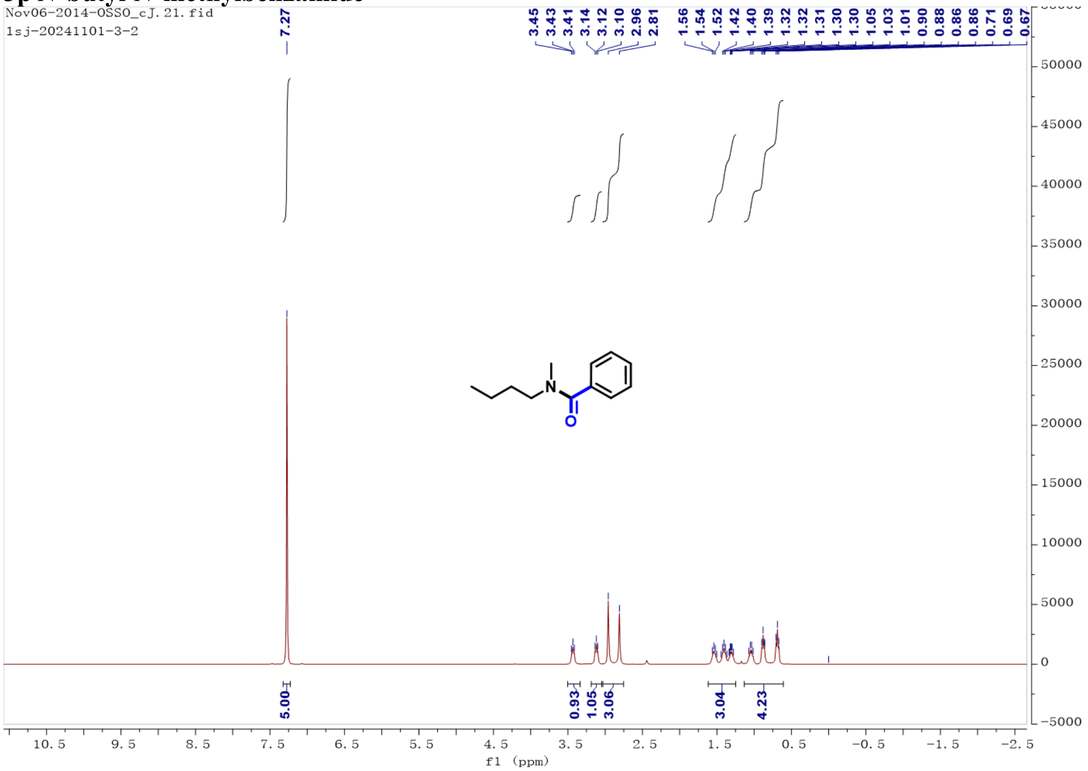

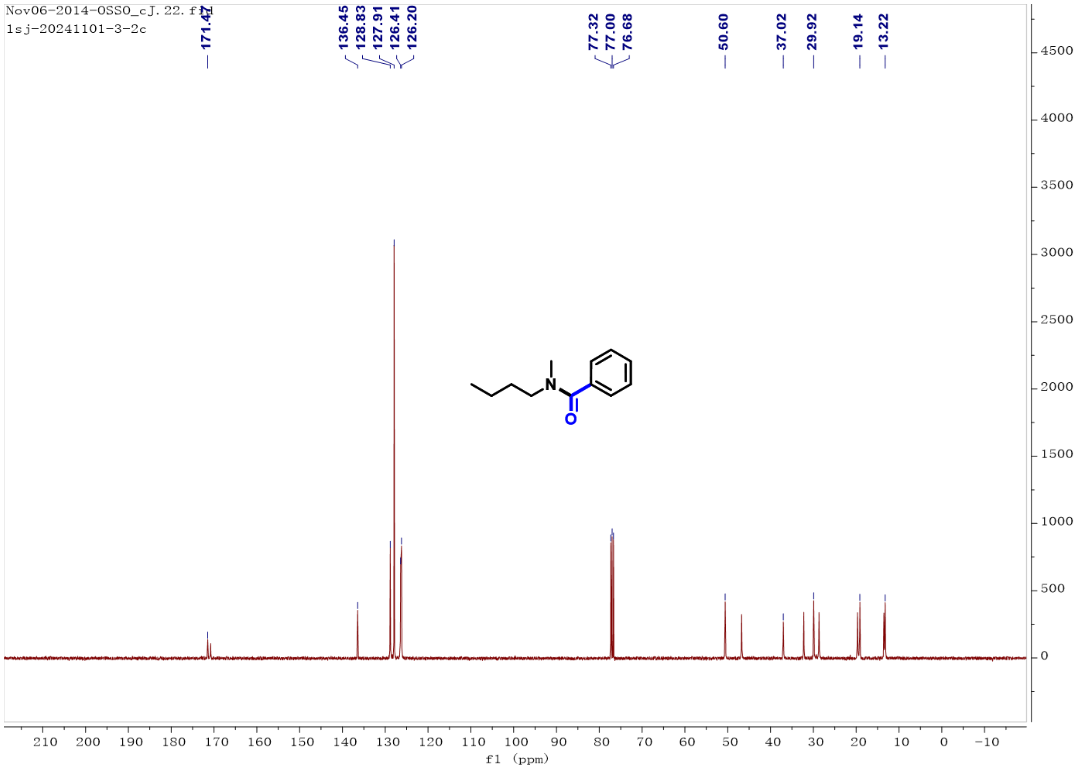

3q *N*-cyclohexyl-*N*-methylbenzamide

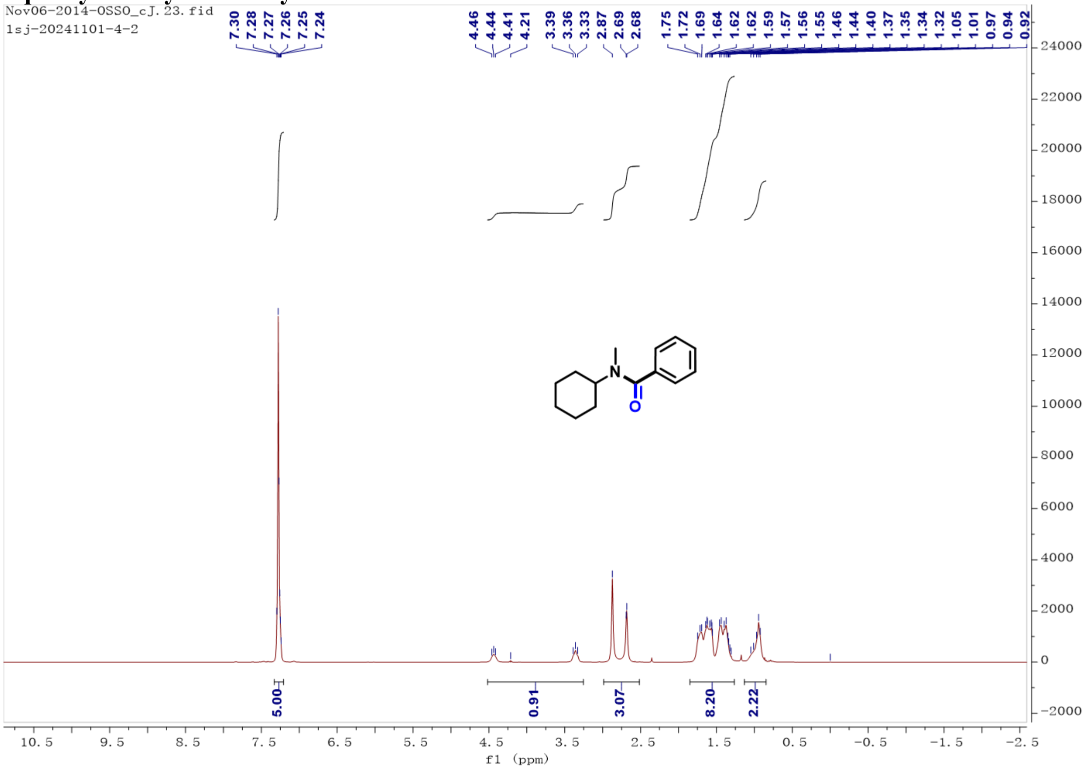

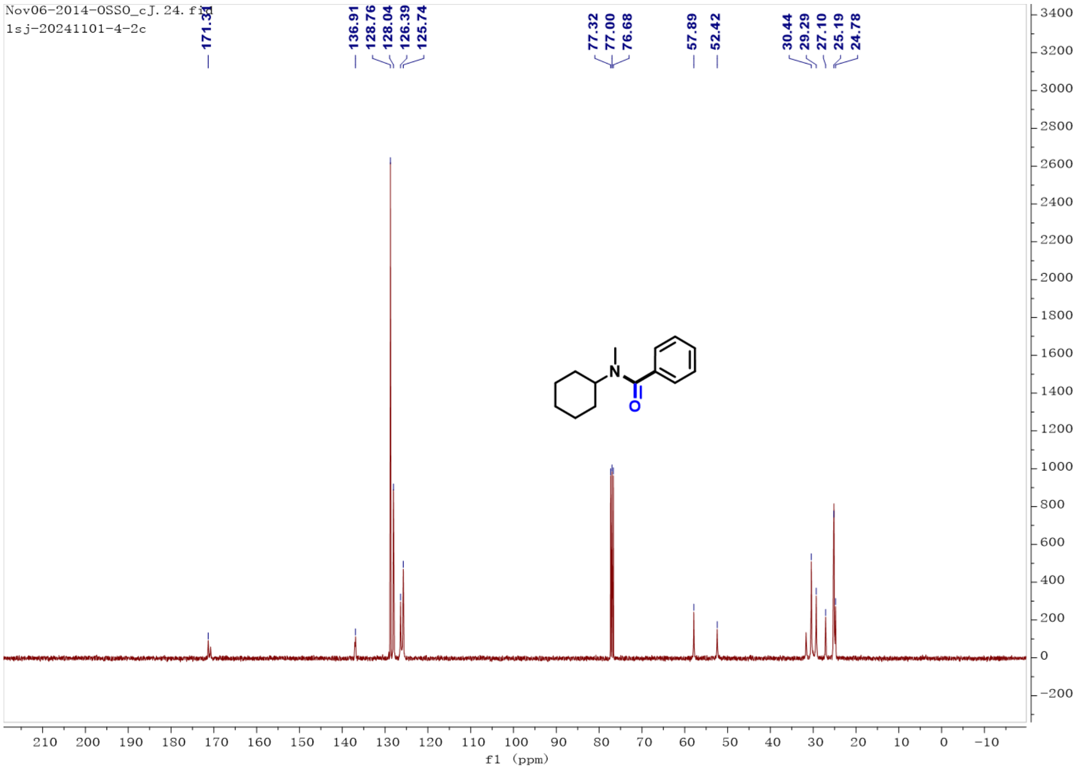

3r *N*-allyl-*N*-methylbenzamide

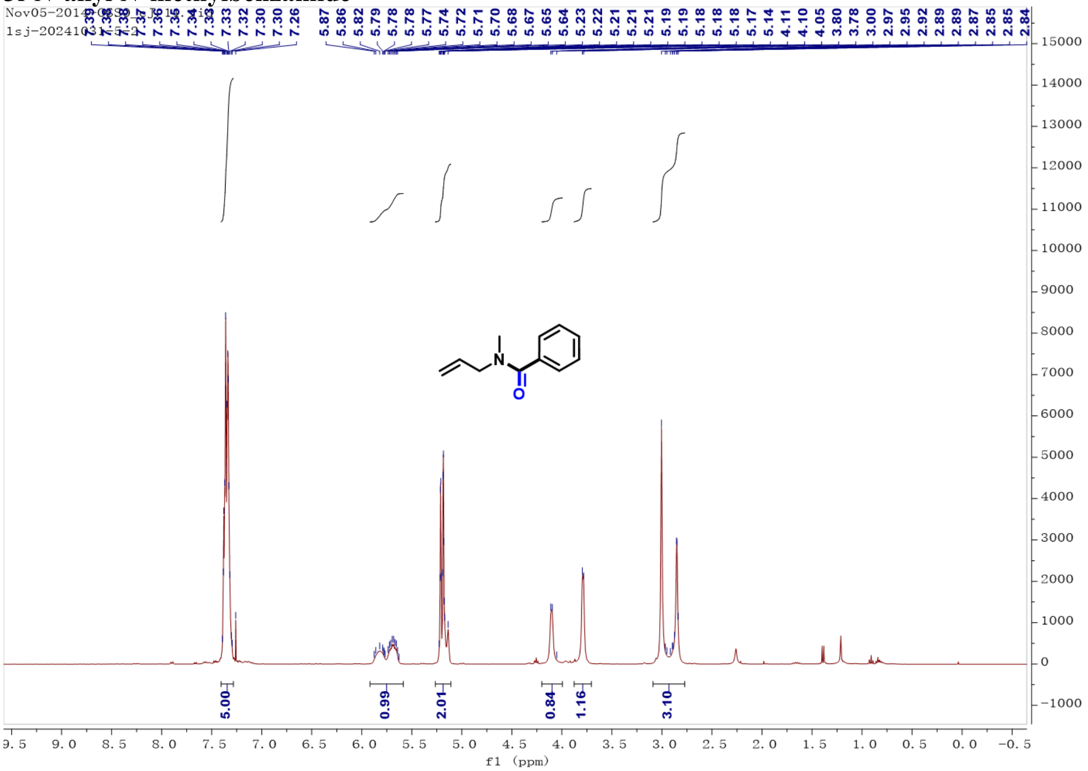

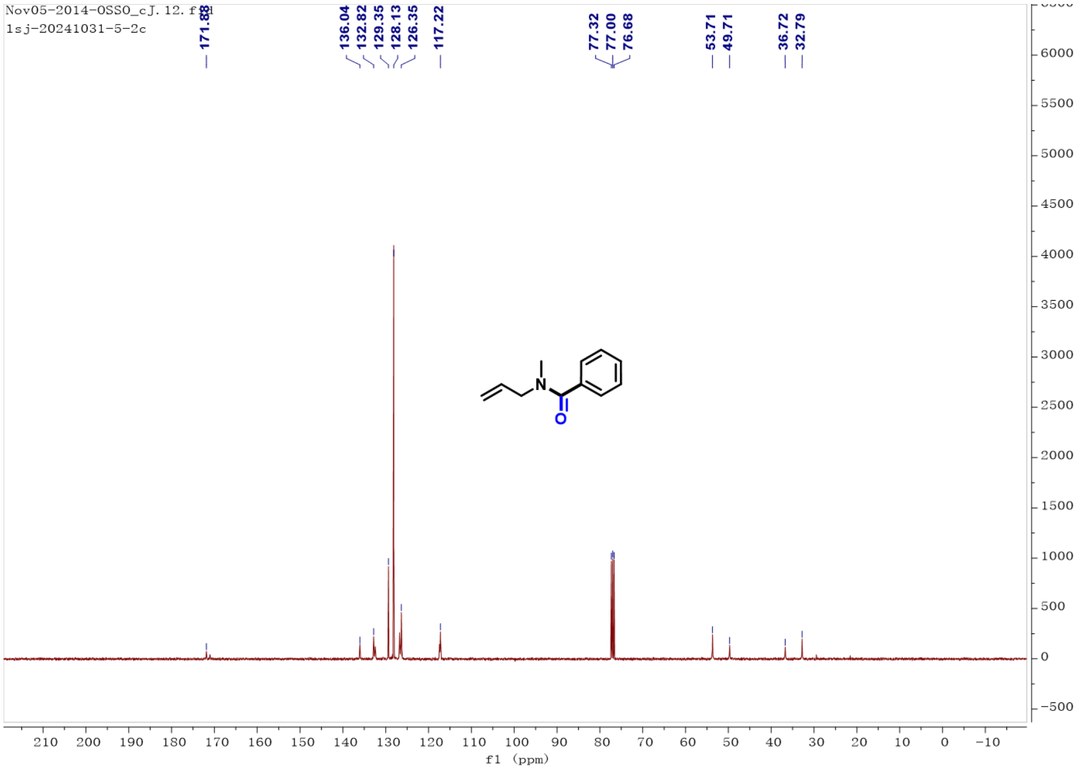

3s *N*-(2-methoxyethyl)-*N*-methylbenzamide

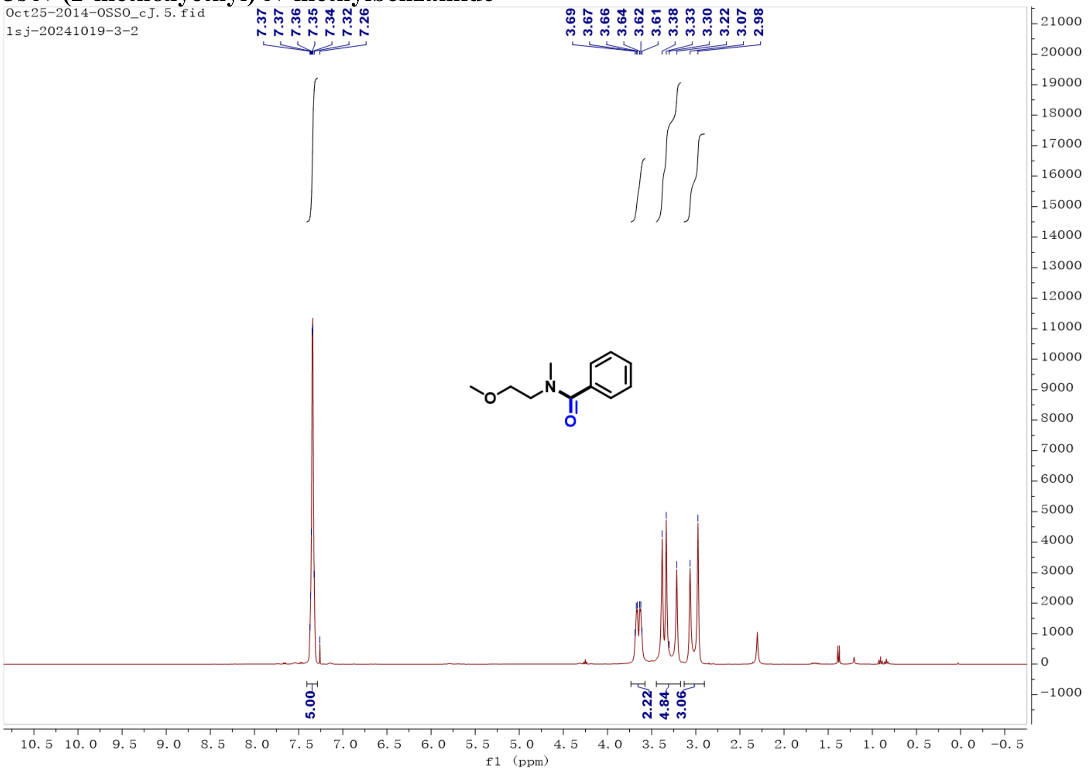

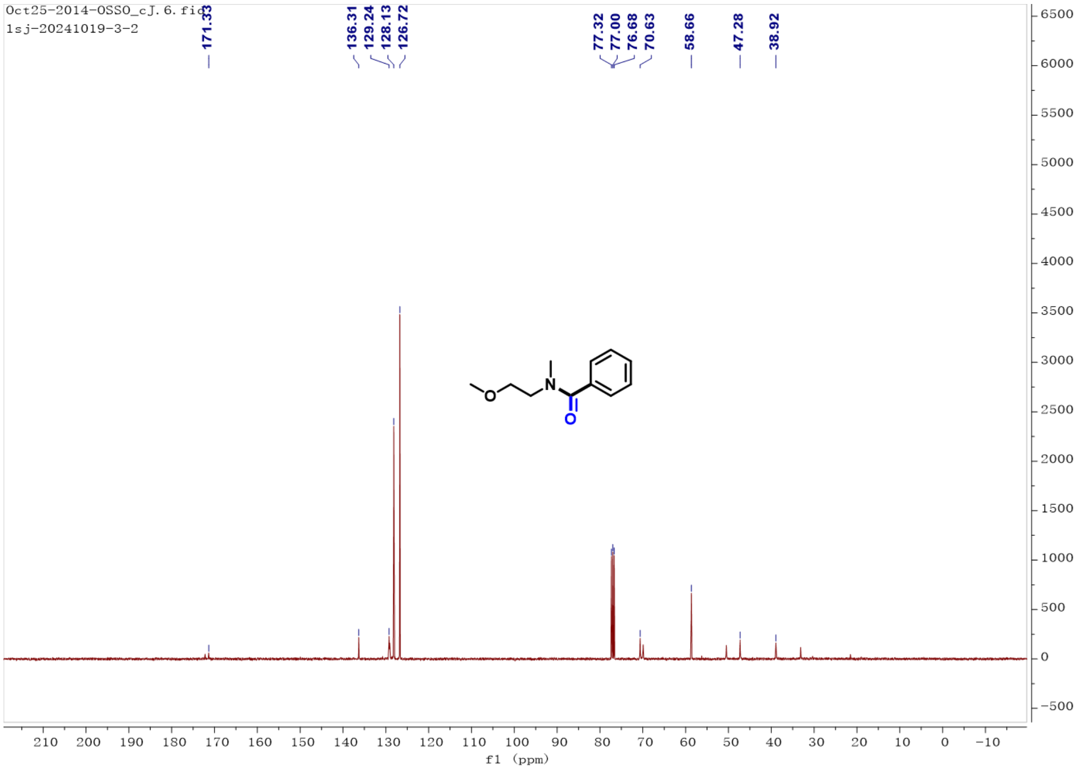

3t *N*-propylbenzamide

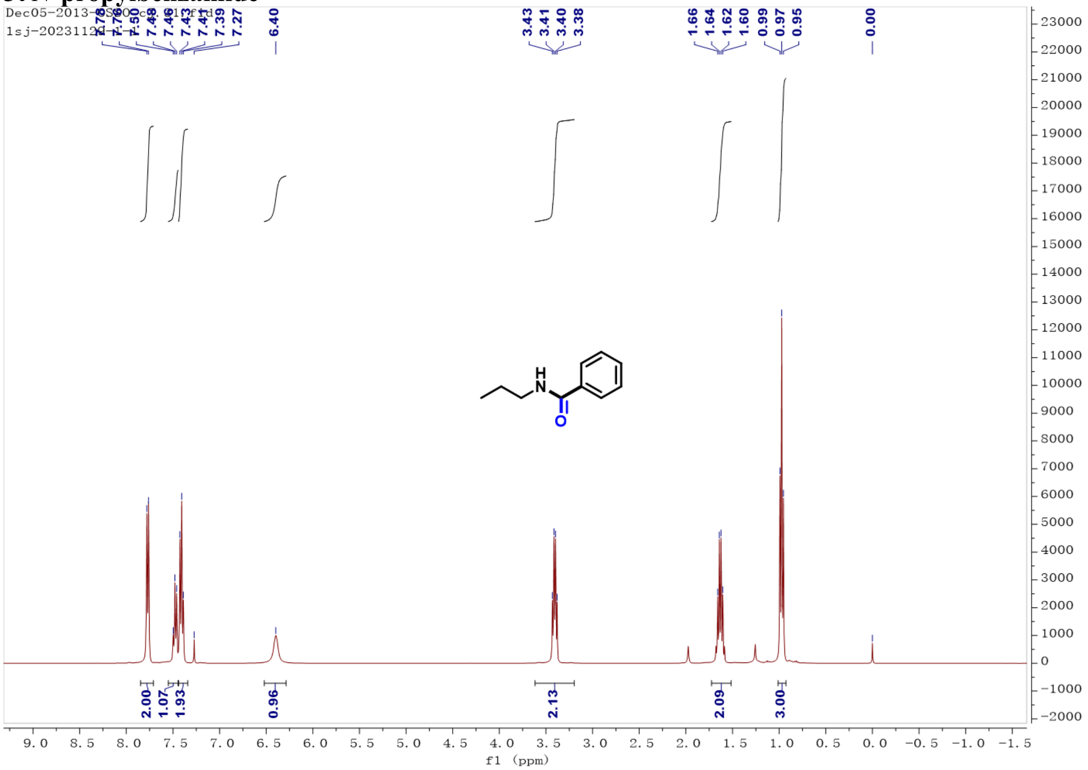

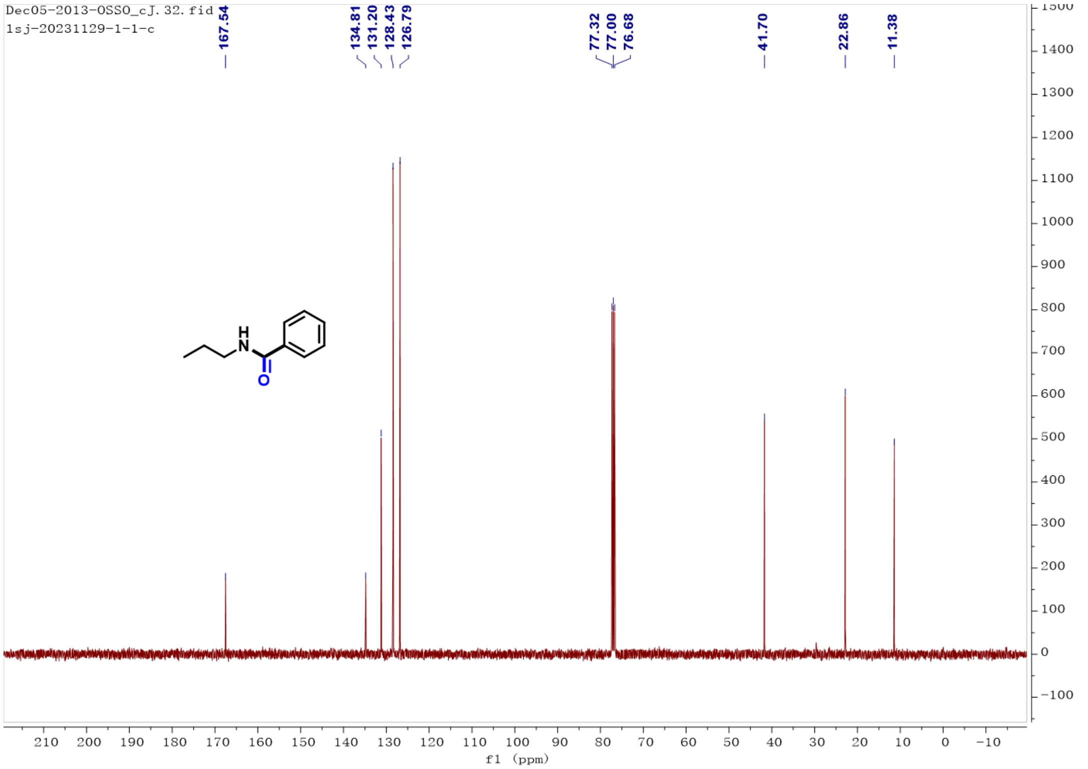

3u *N*-butylbenzamide

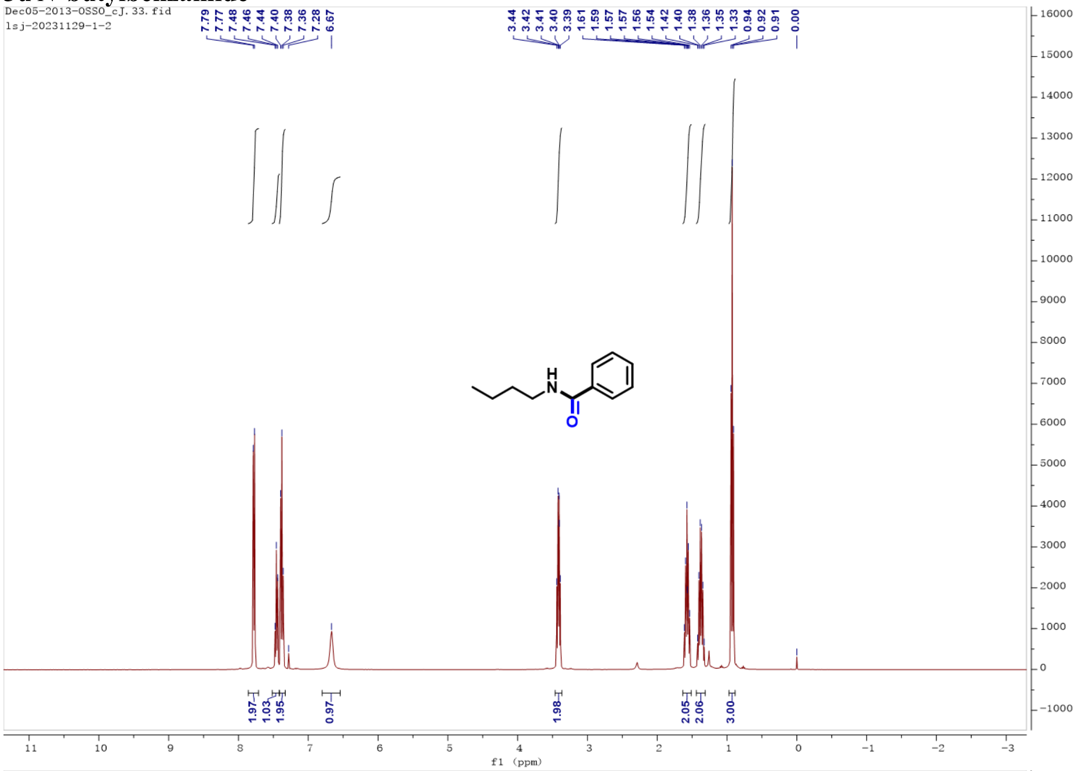

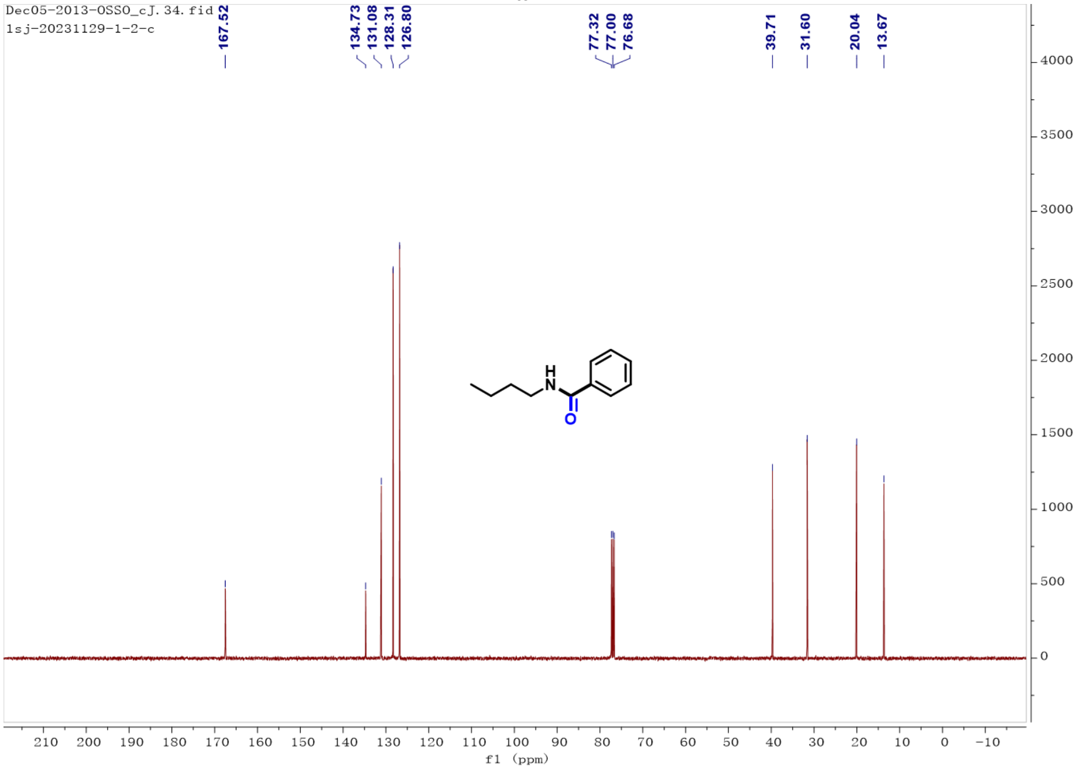

3v *N*-hexylbenzamide

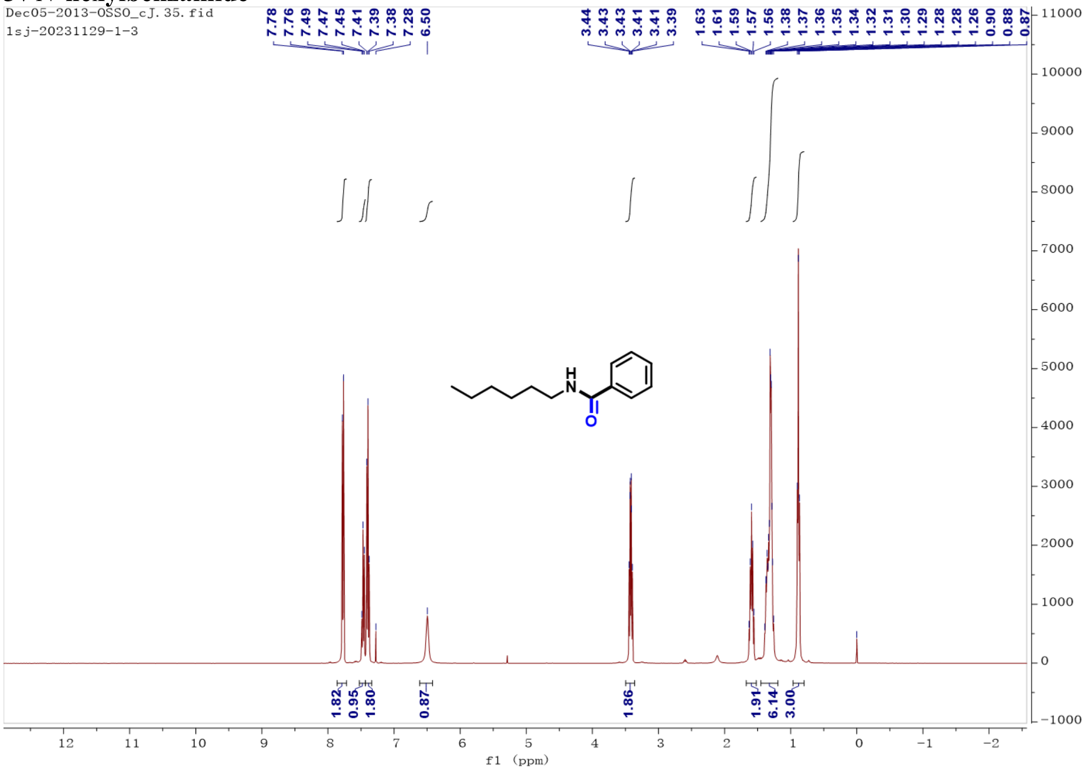

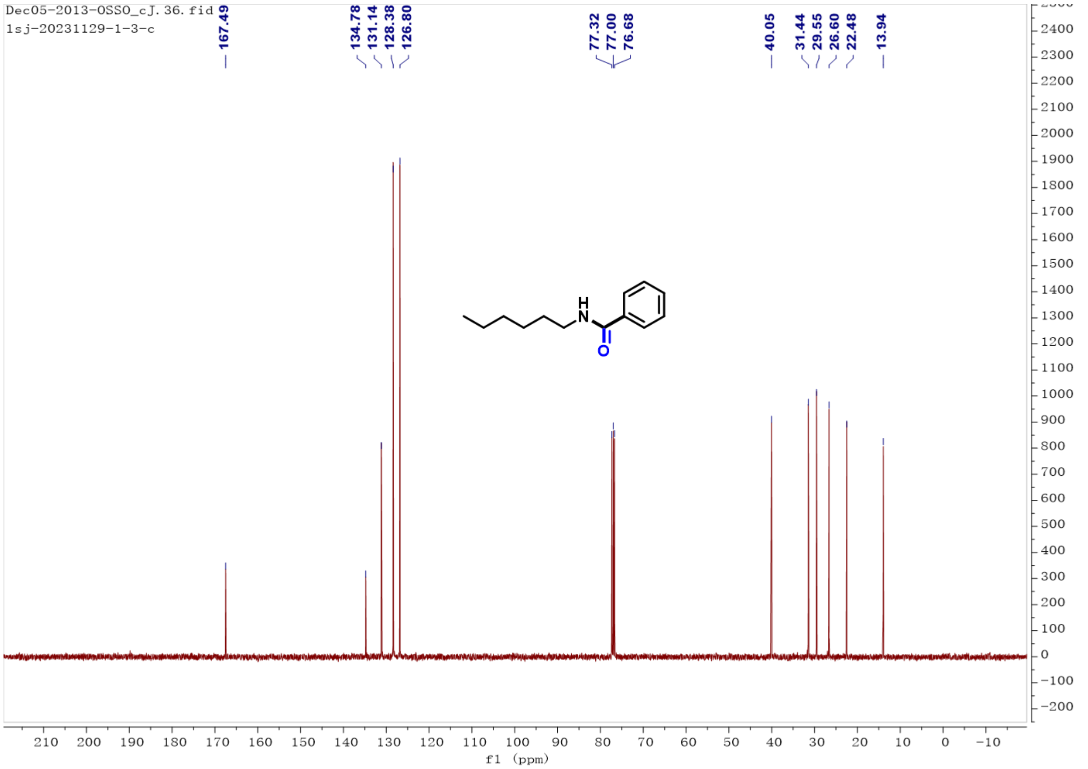

3w *N*-heptylbenzamide

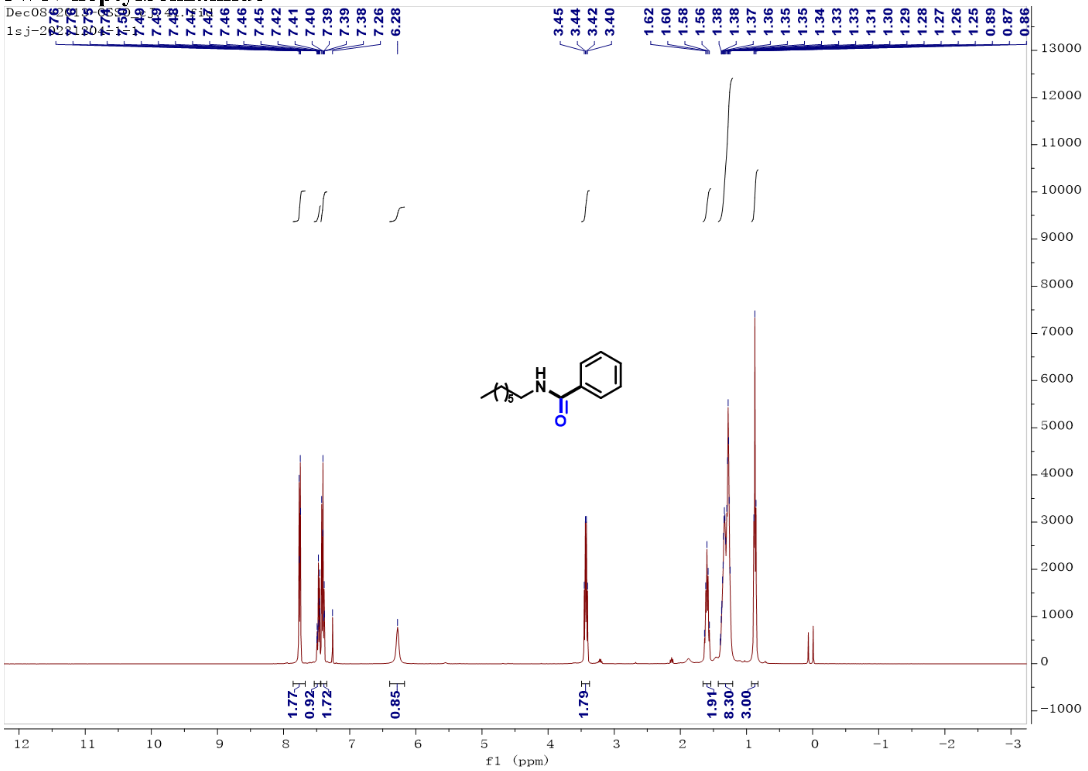

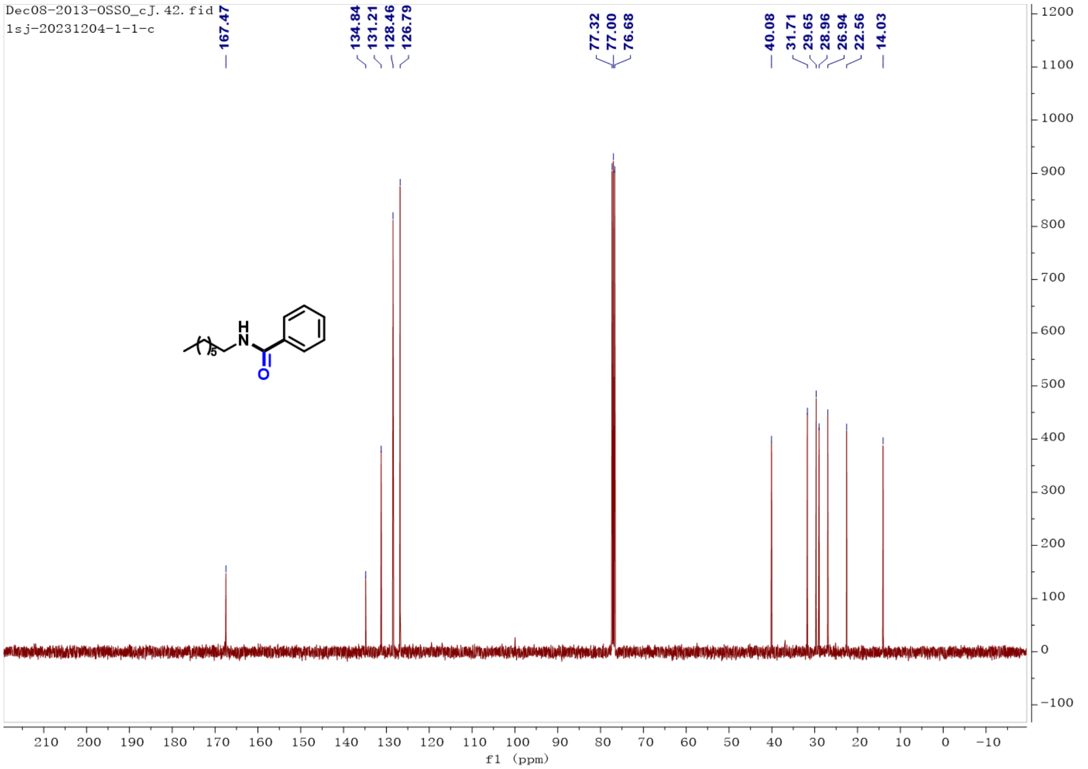

3x *N*-octylbenzamide

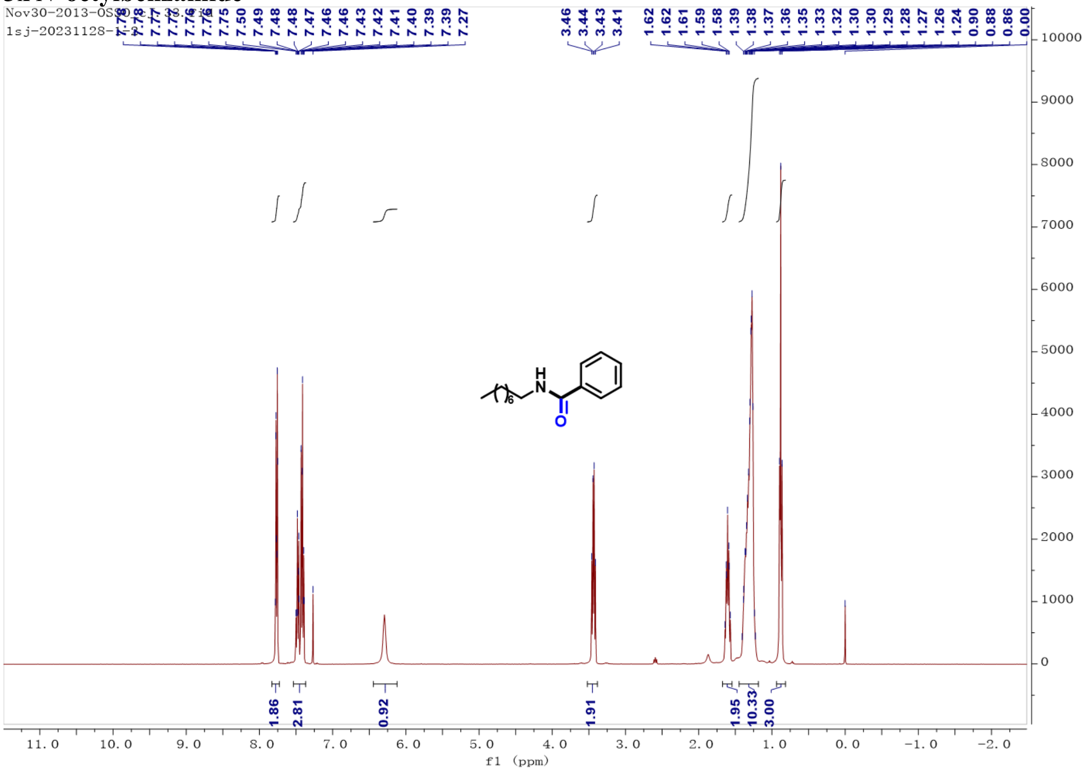

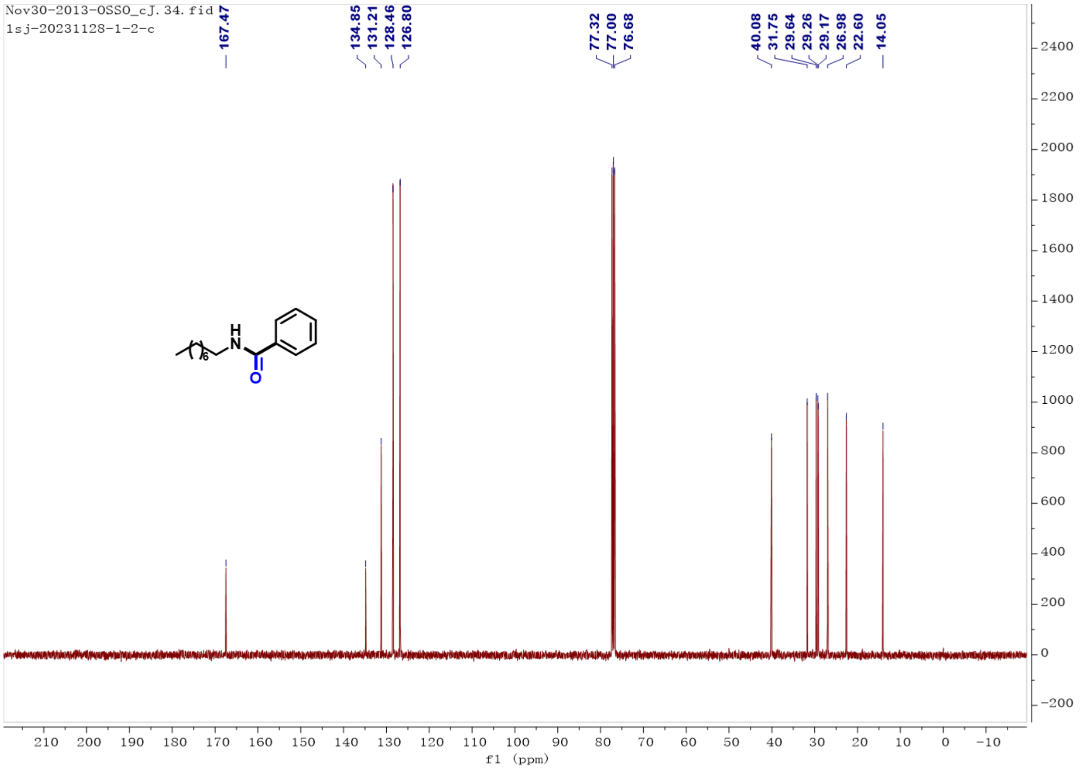

3y *N*-dodecylbenzamide

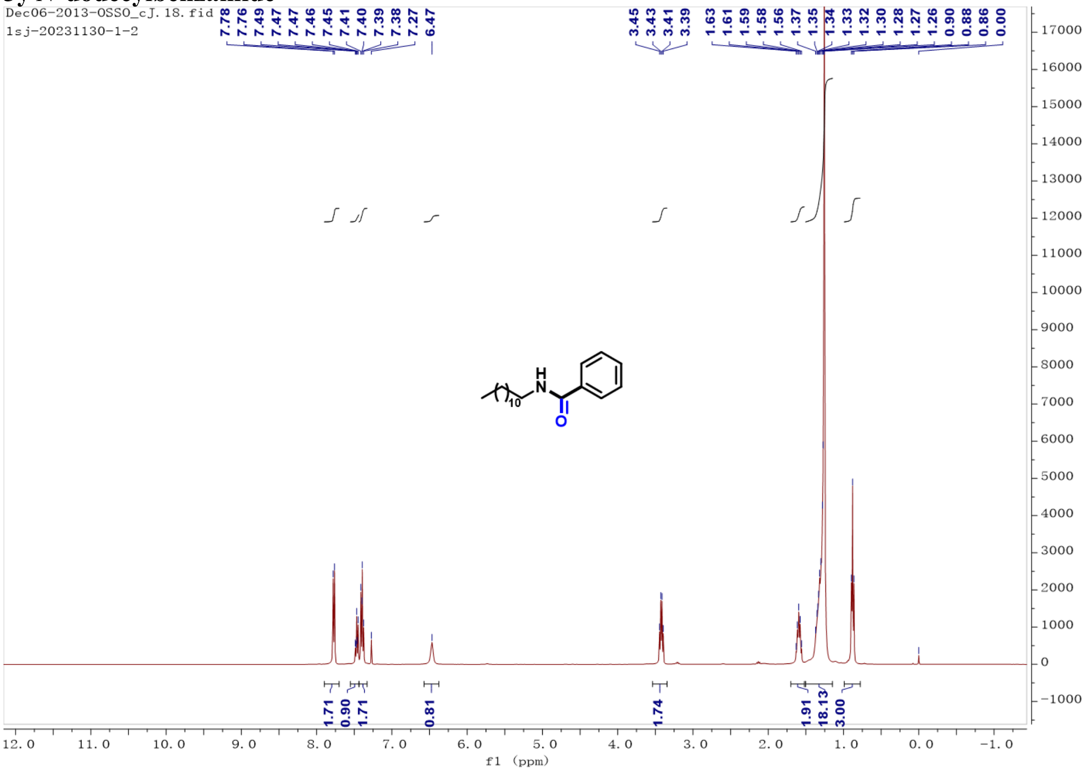

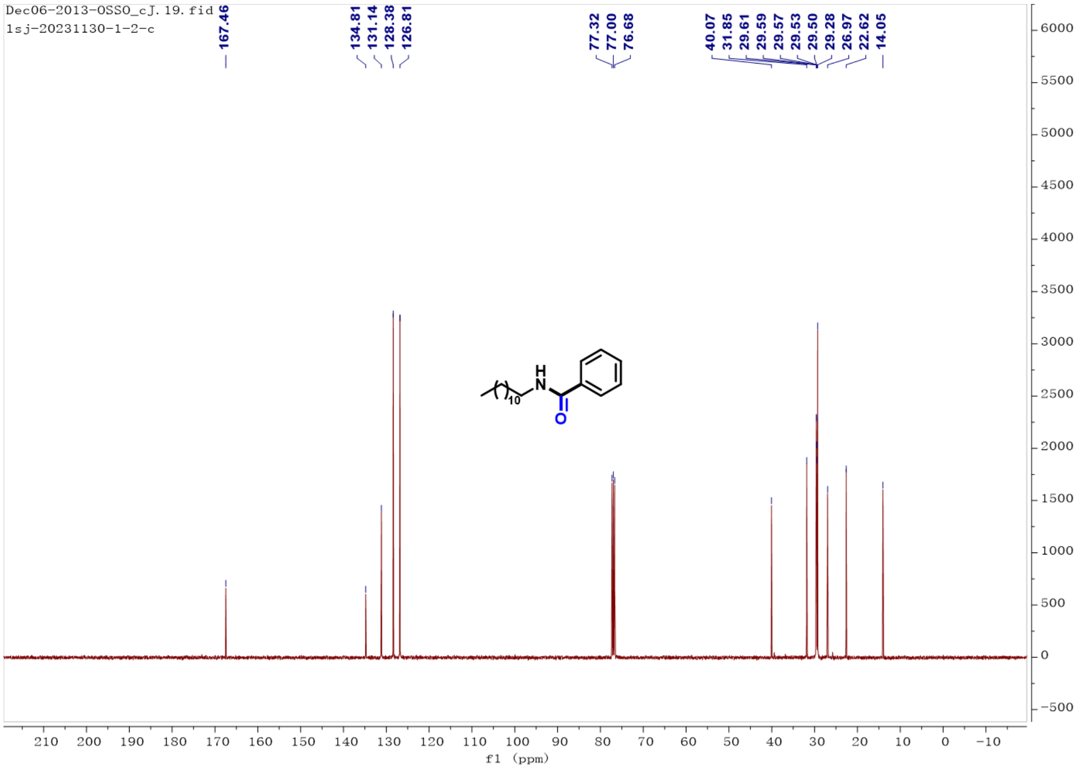

3z *N*-(sec-butyl)benzamide

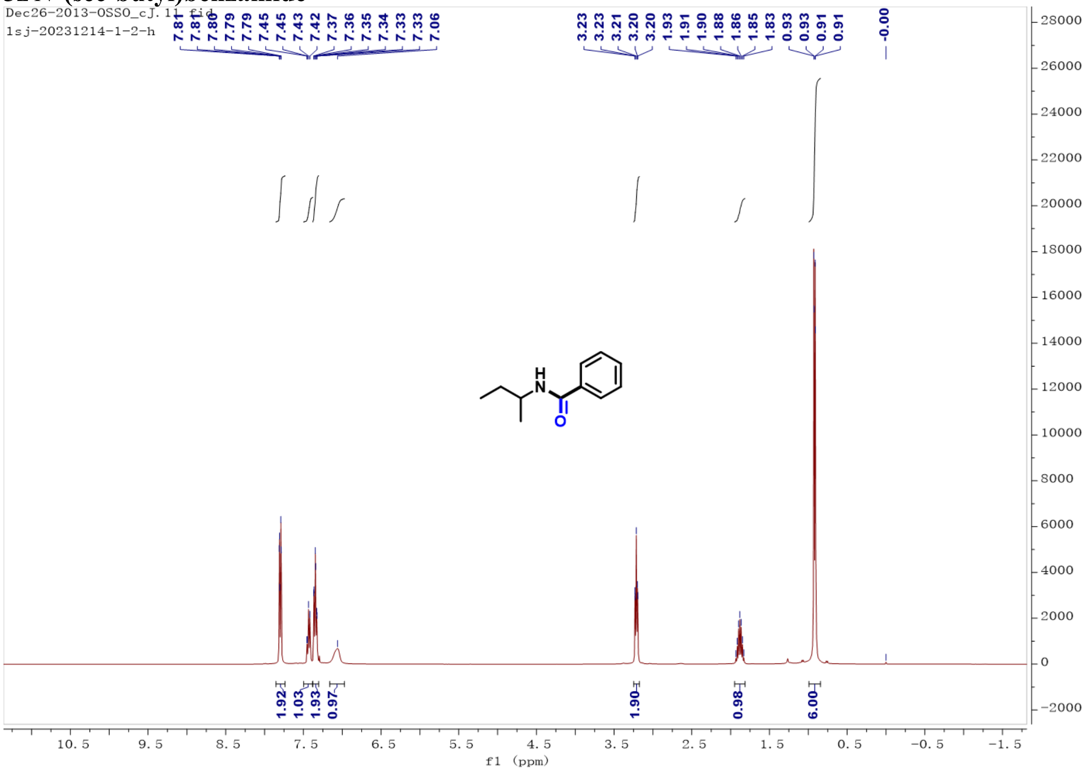

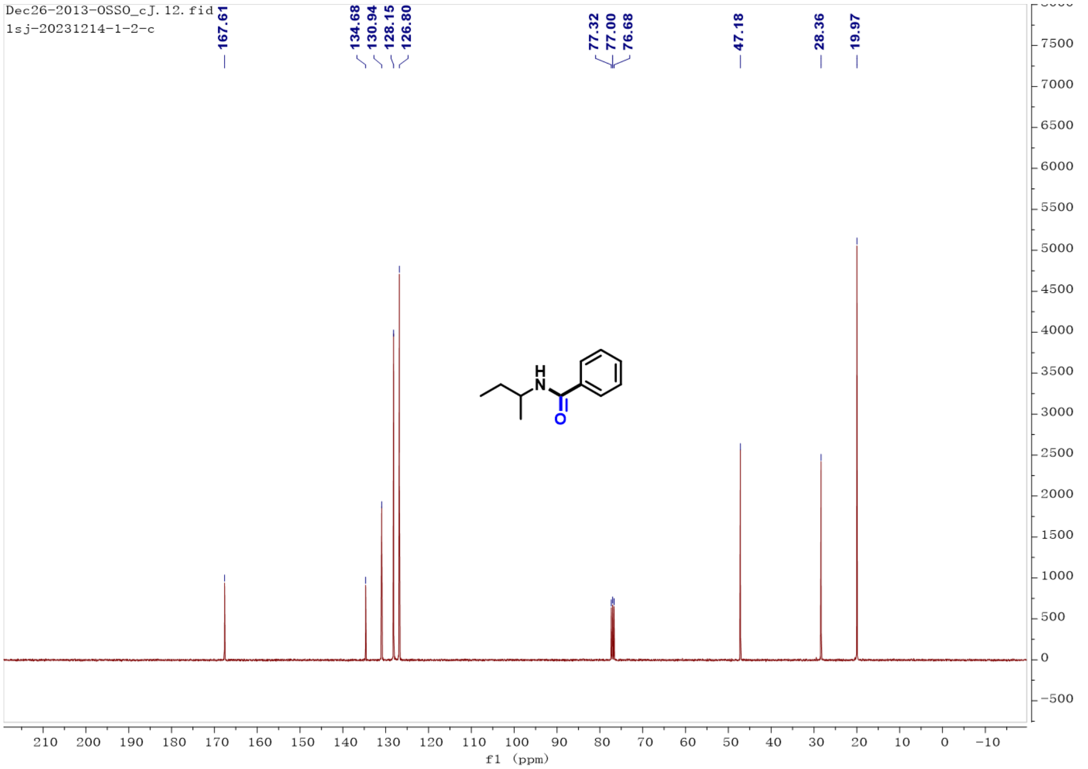

3aa *N*-cyclopentylbenzamide

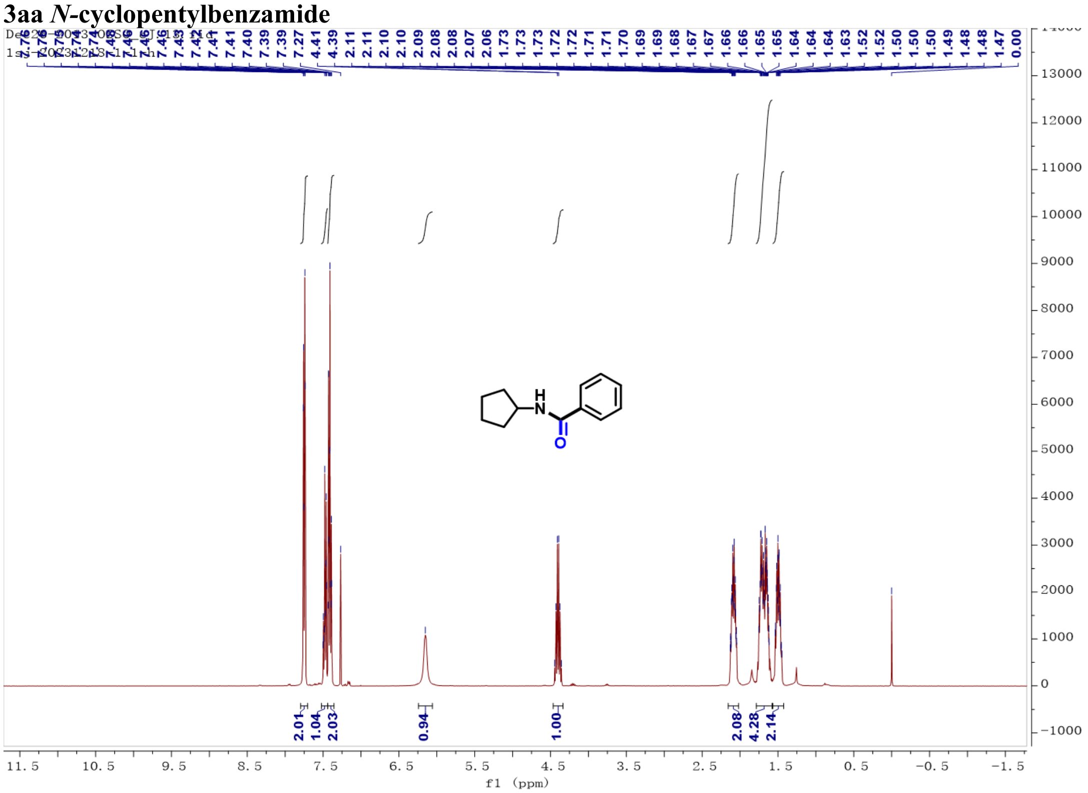

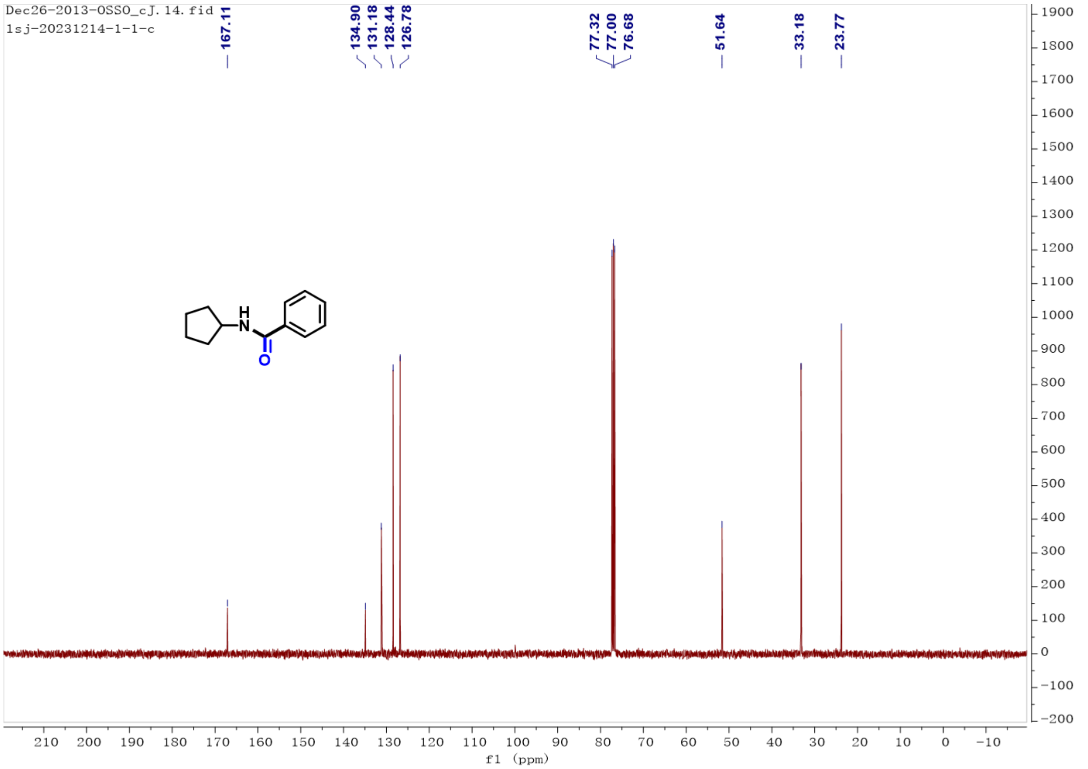

3ab *N*-cyclohexylbenzamide

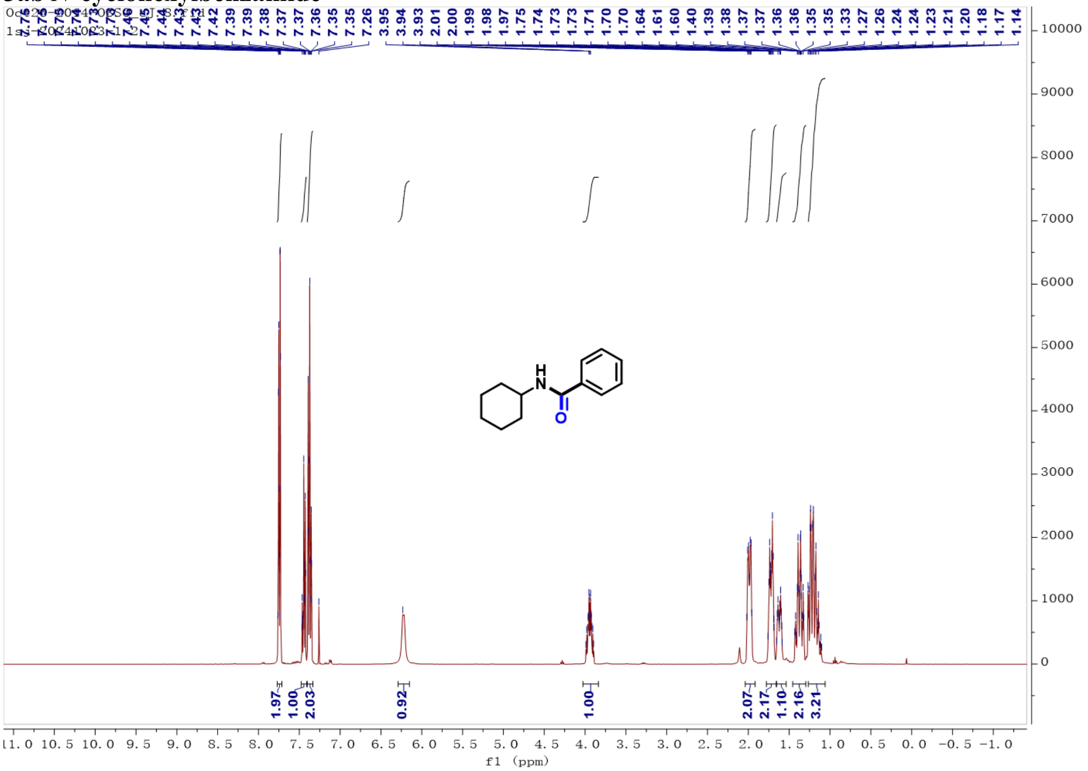

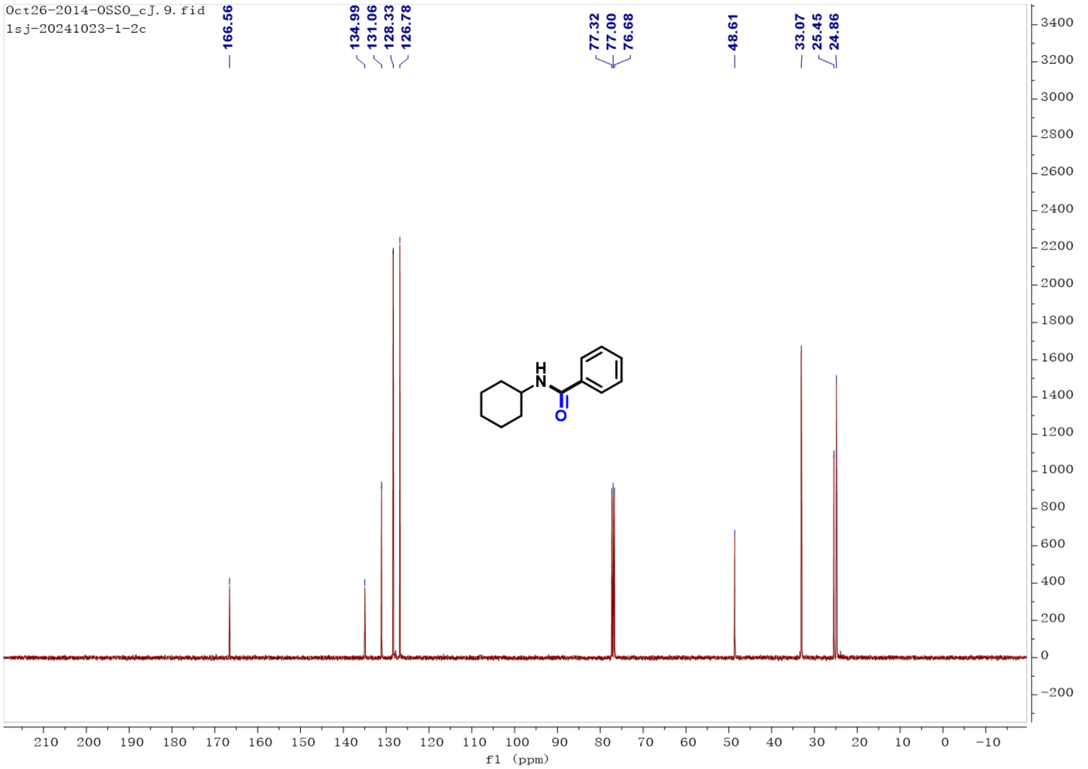

3ac *N*-(adamantan-1-yl)benzamide

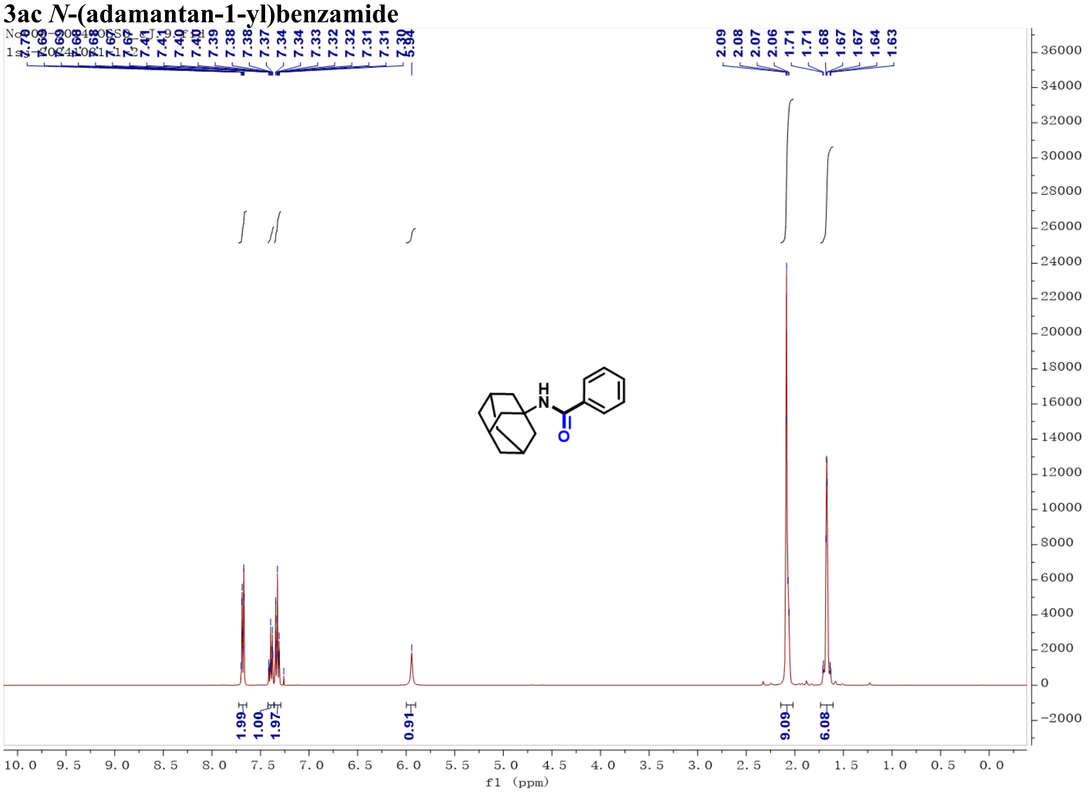

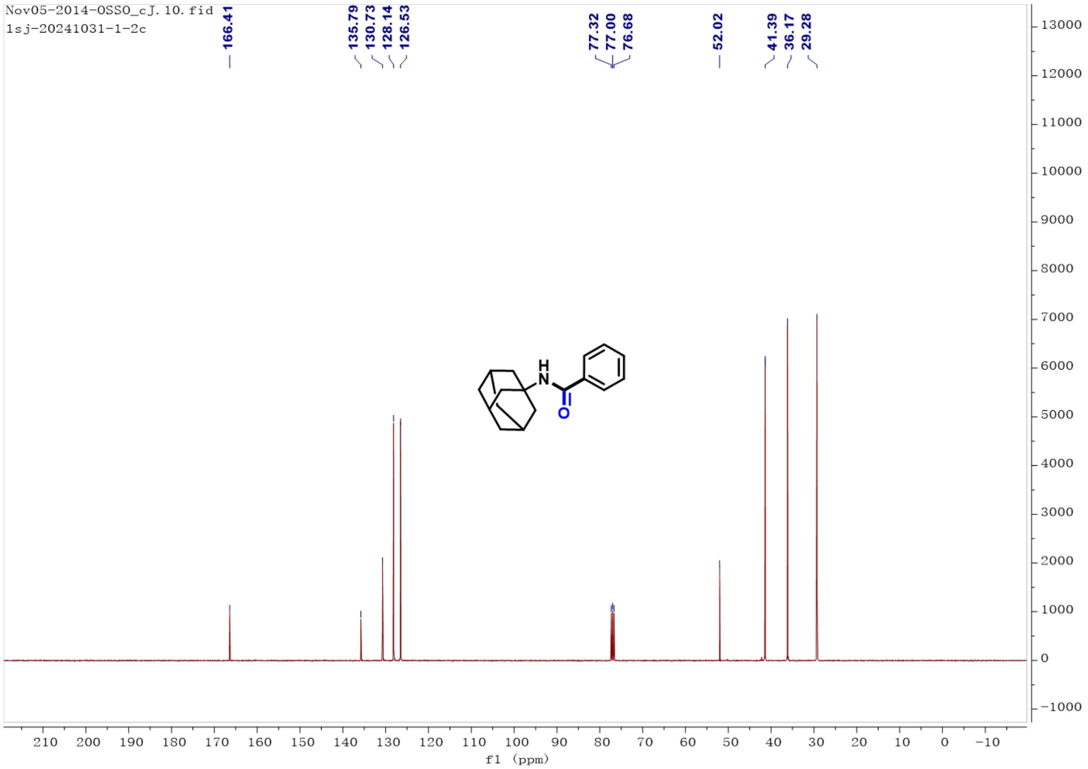

3ad *N*-(cyclopropylmethyl)benzamide

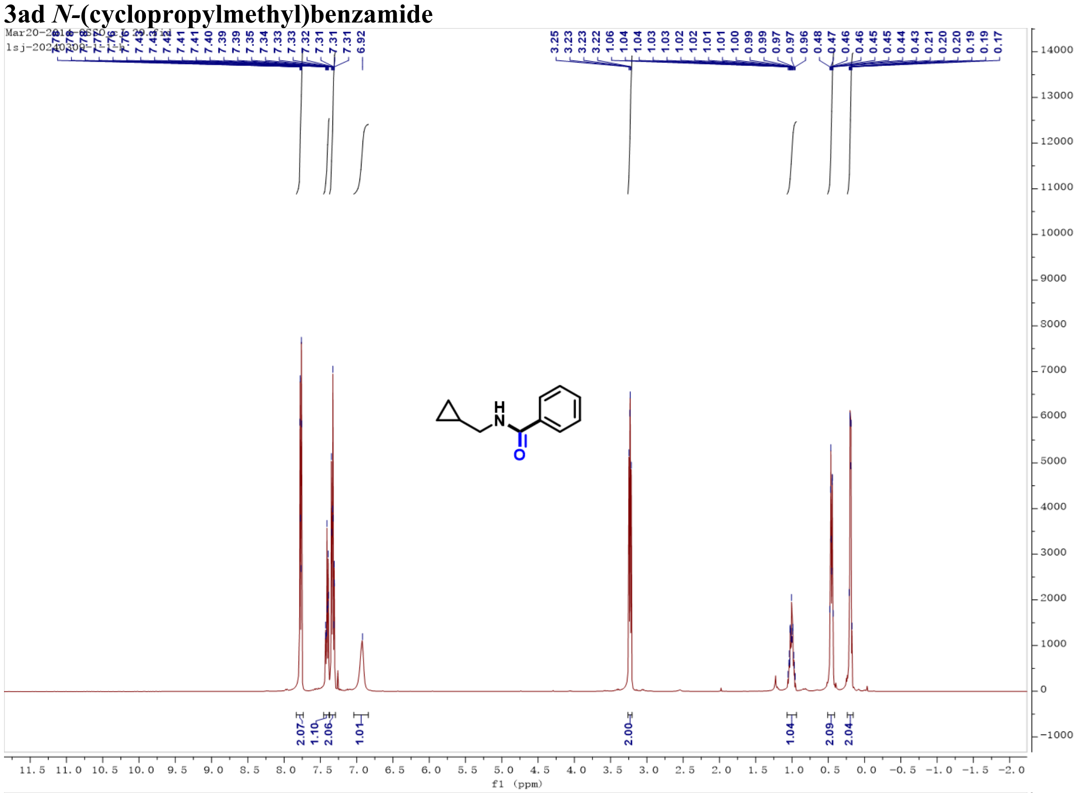

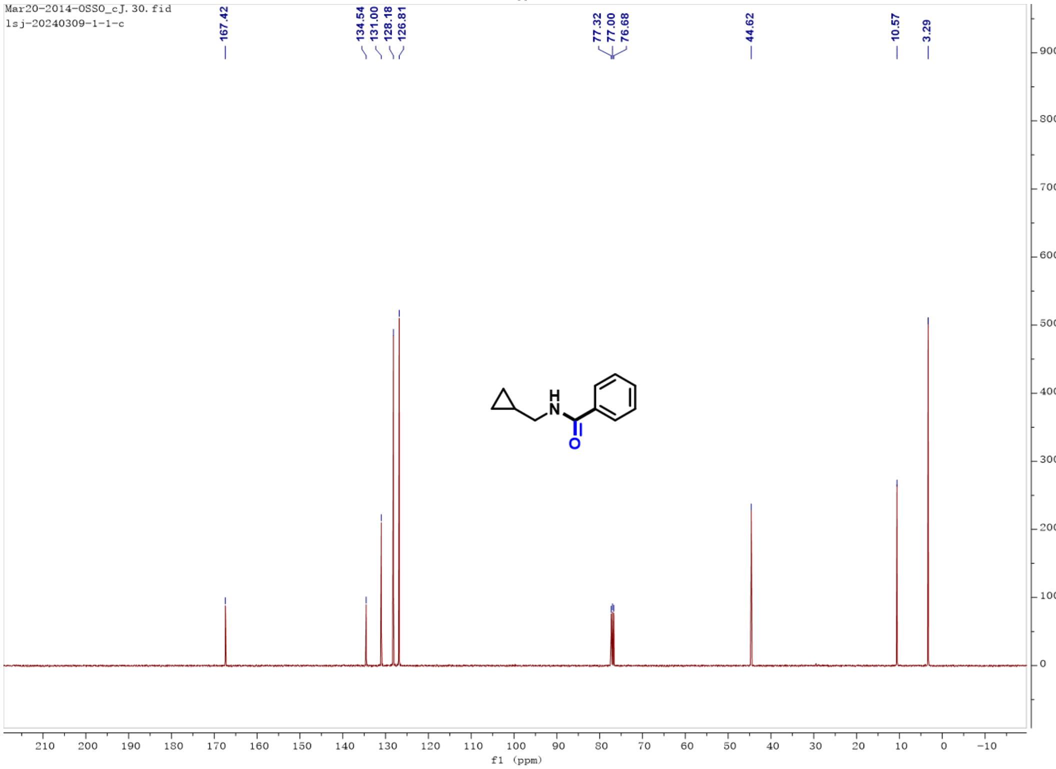

3ae ***N*-(cyclohexylmethyl)benzamide**

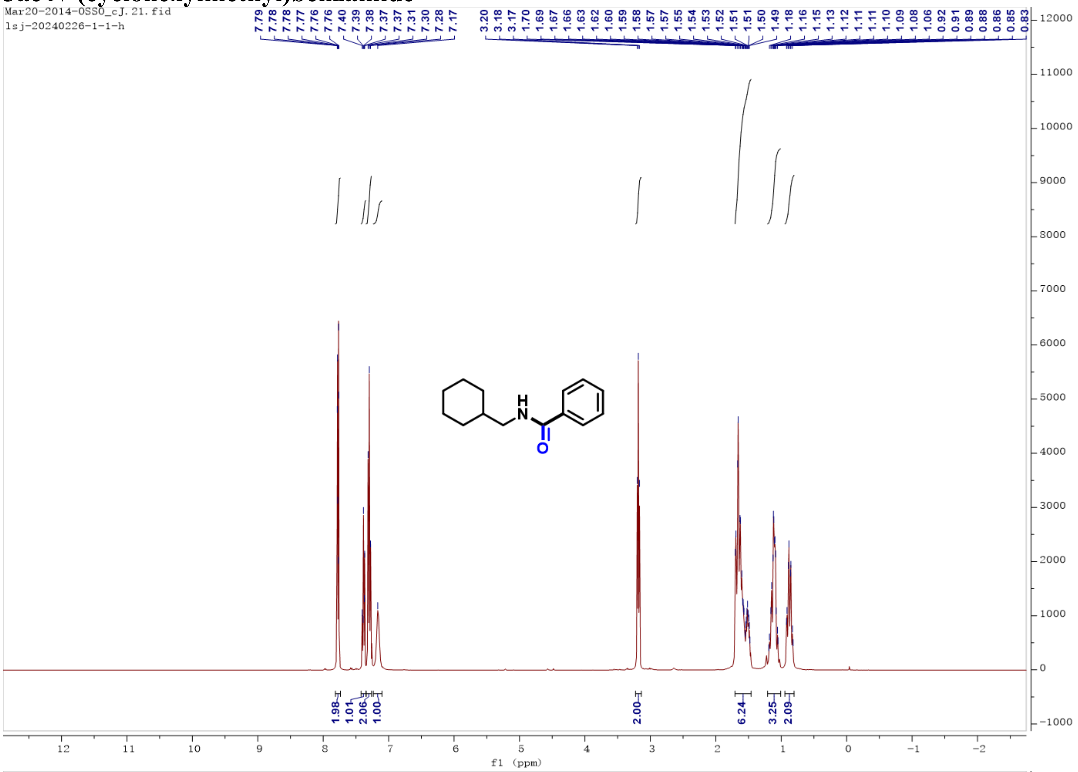

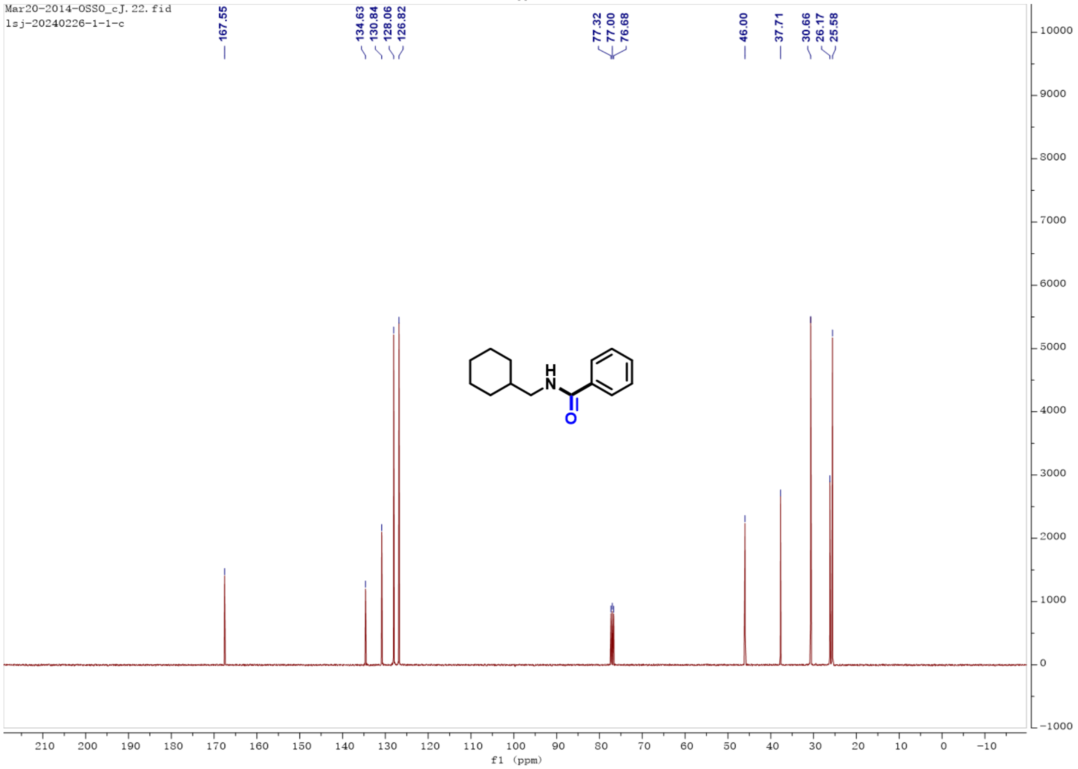

3af *N*-((tetrahydro-2*H*-pyran-4-yl)methyl)benzamide

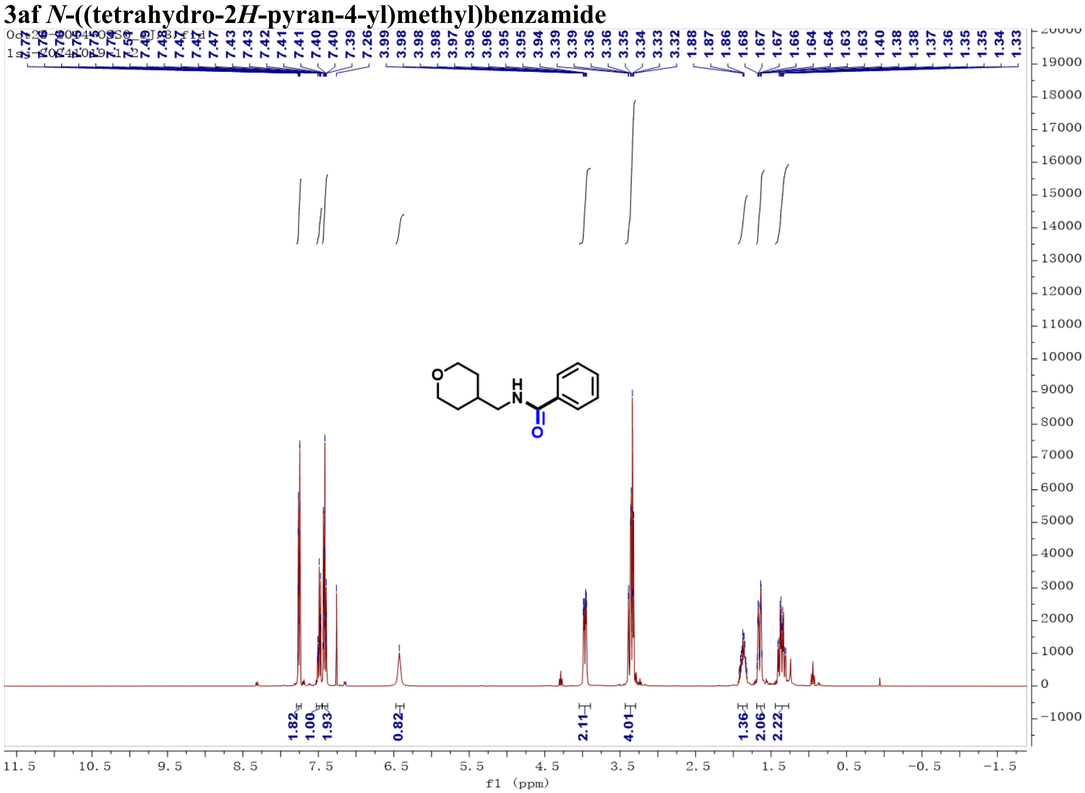

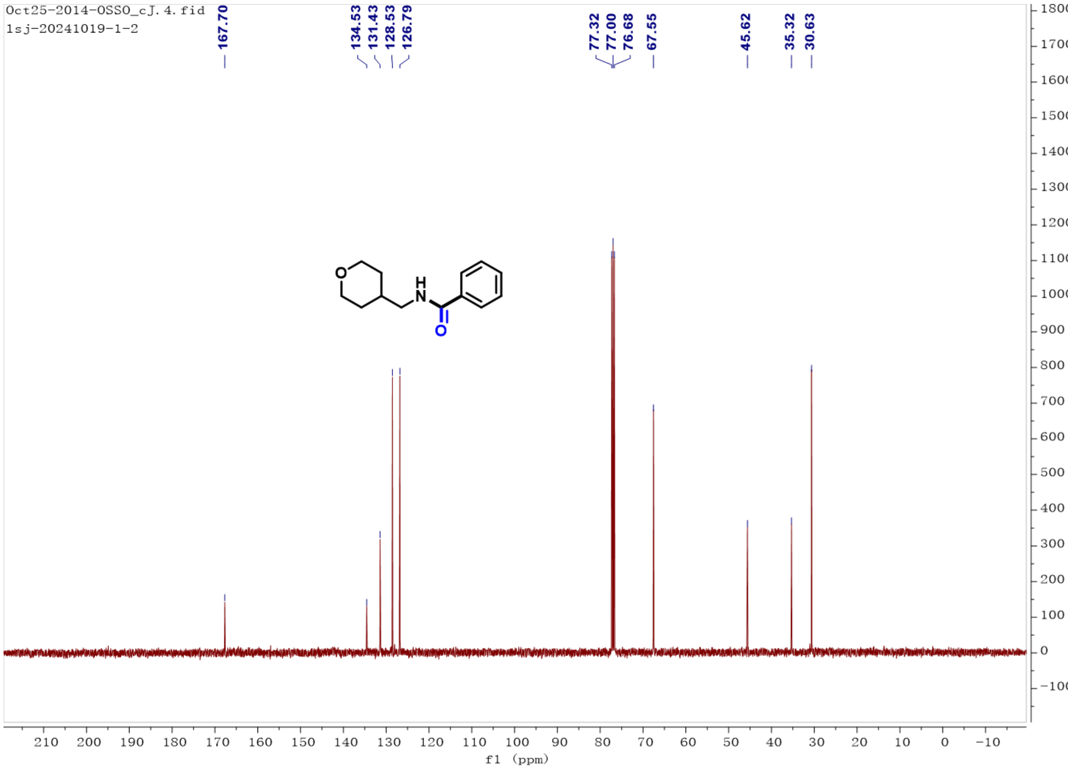

3ag *N*-(furan-2-ylmethyl)benzamide

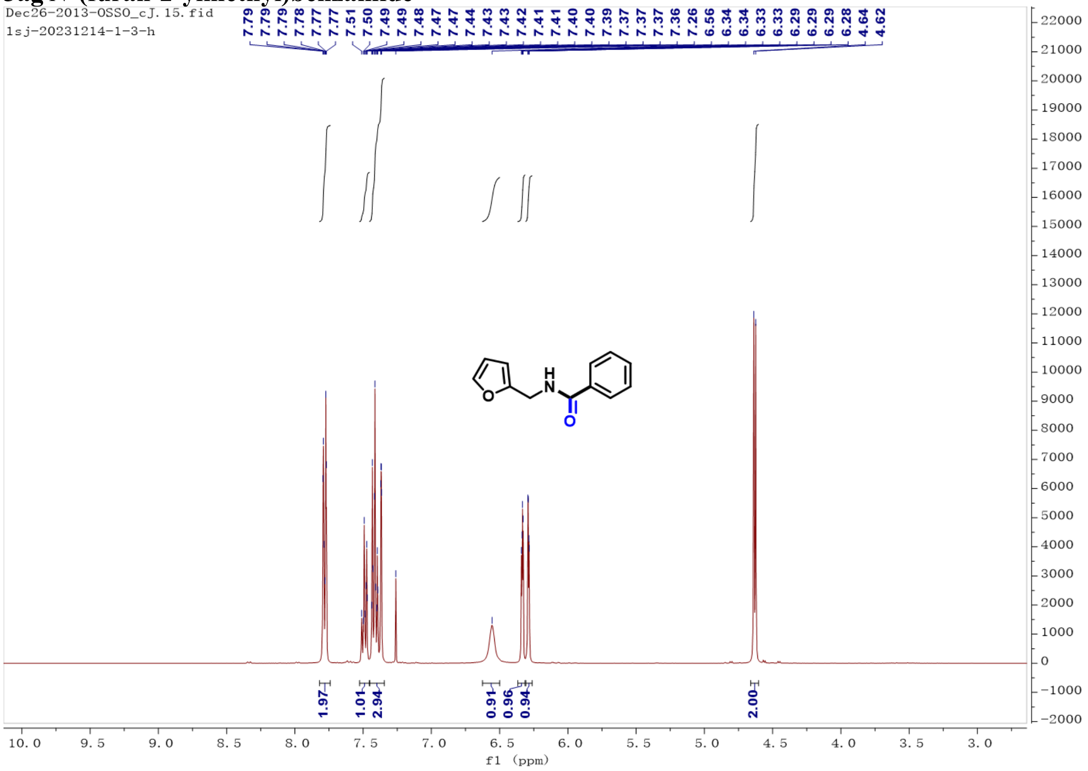

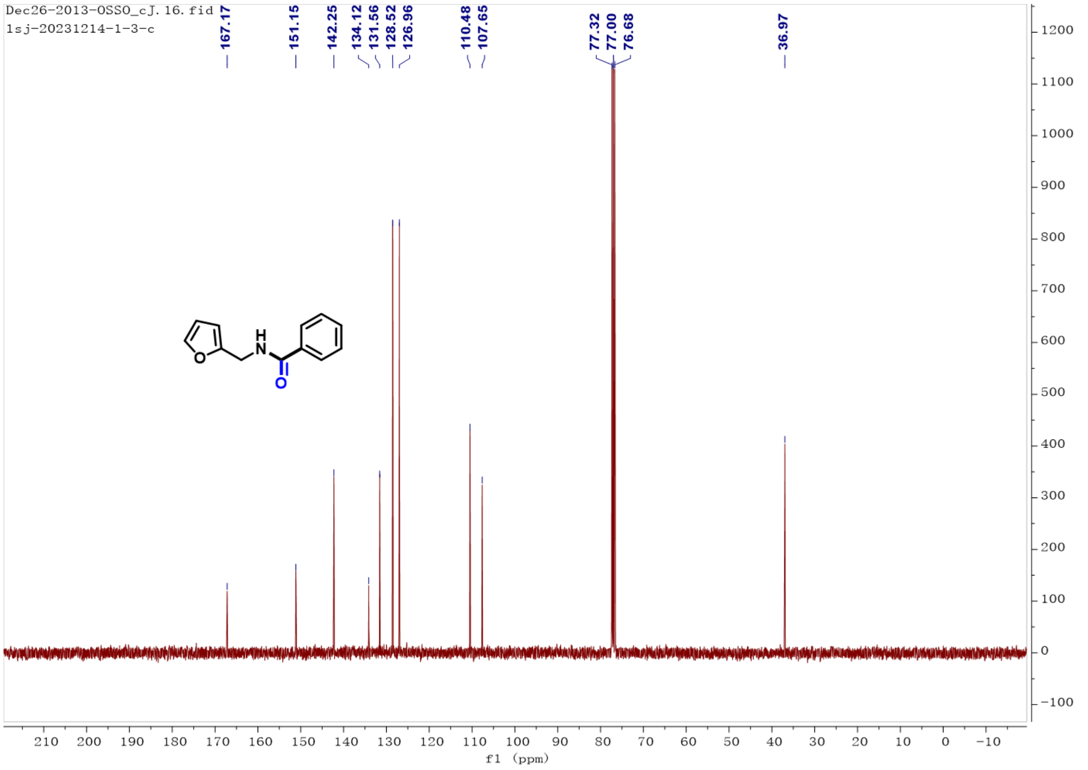

3ah *N*-(pyridin-3-ylmethyl)benzamide

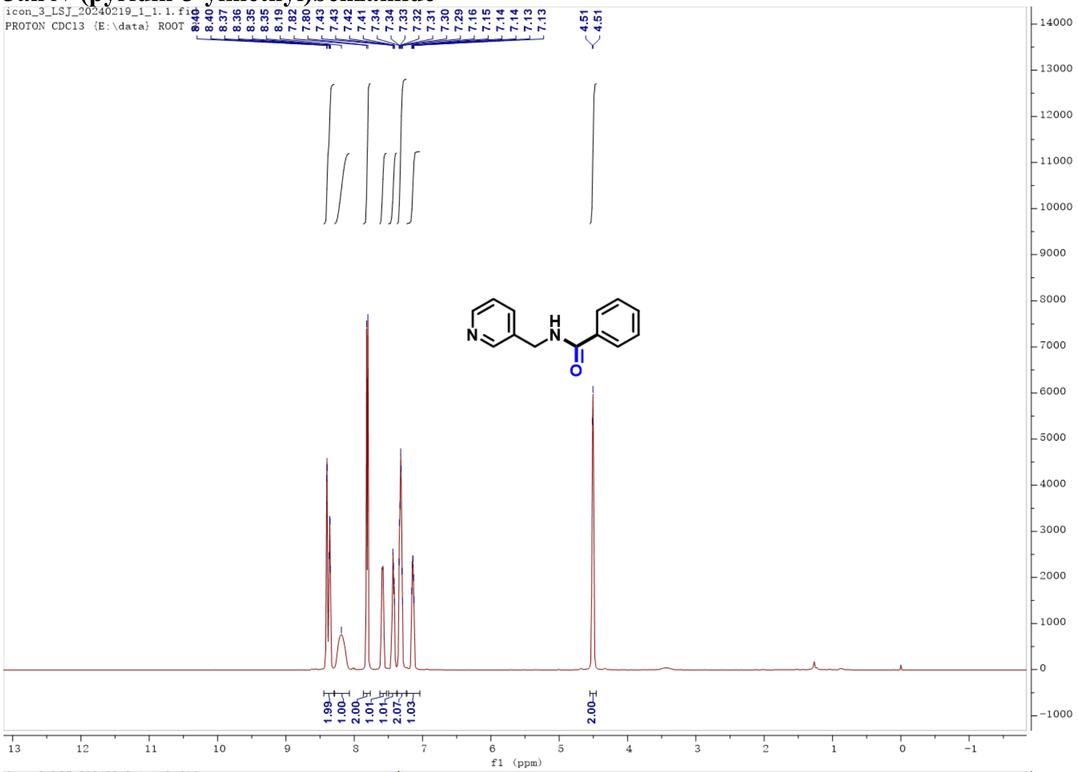

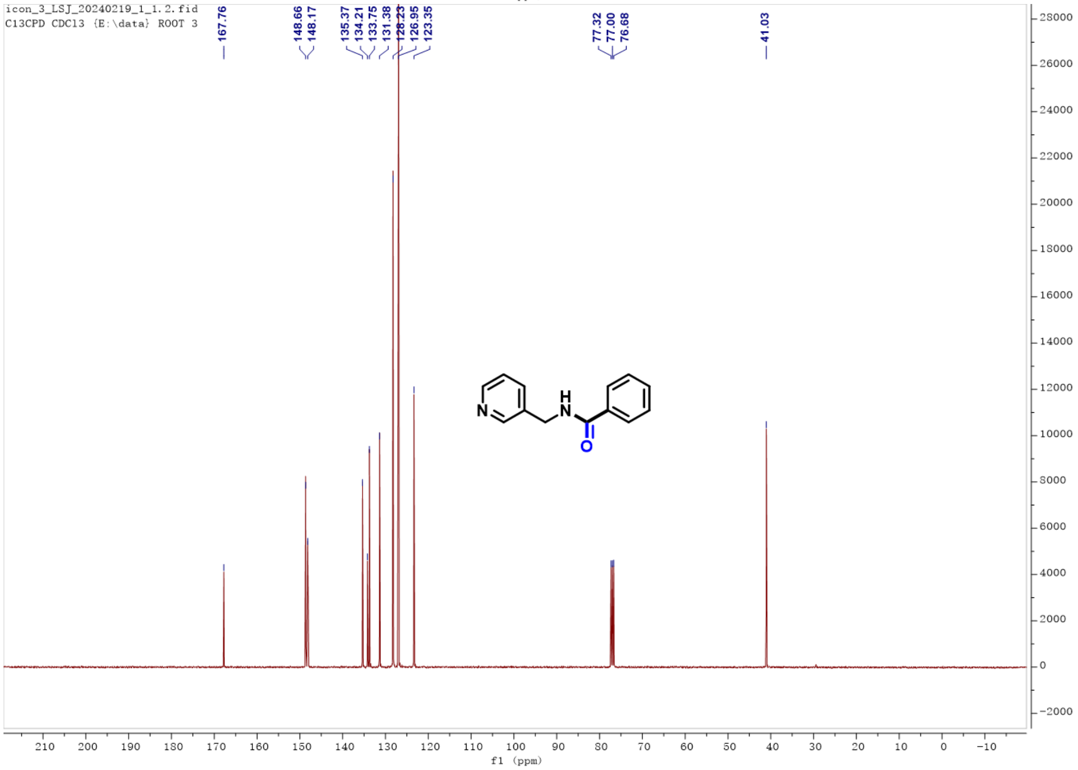

3ai *N*-(thiophen-2-ylmethyl)benzamide

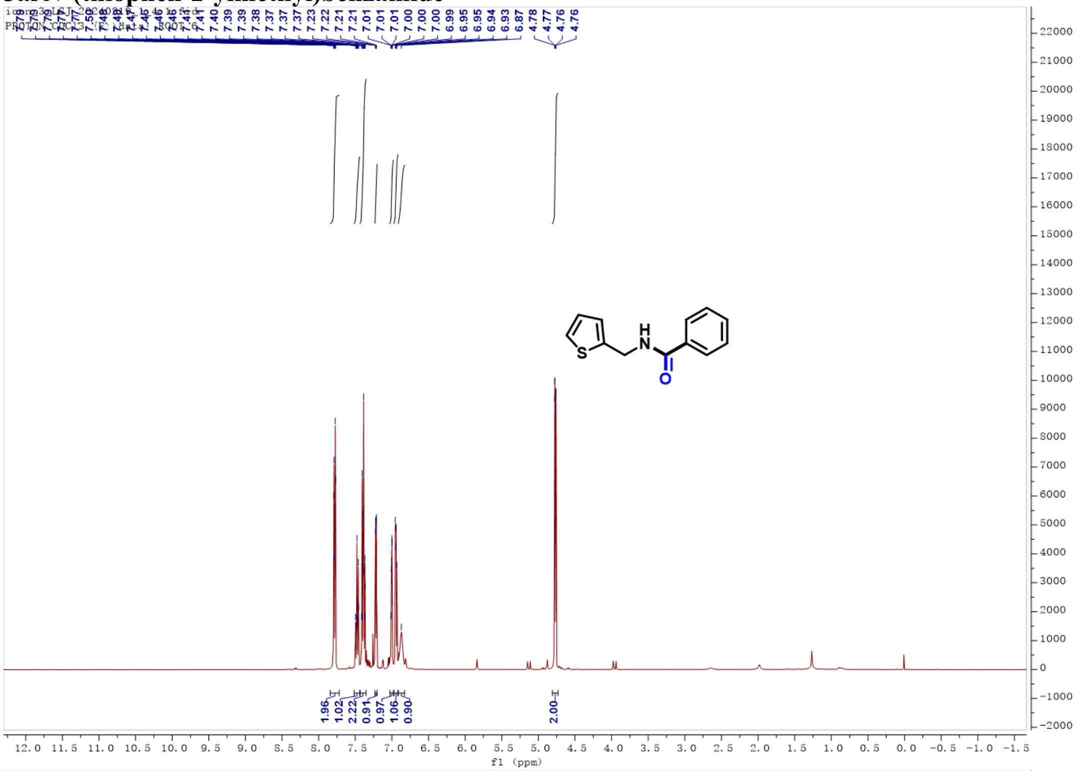

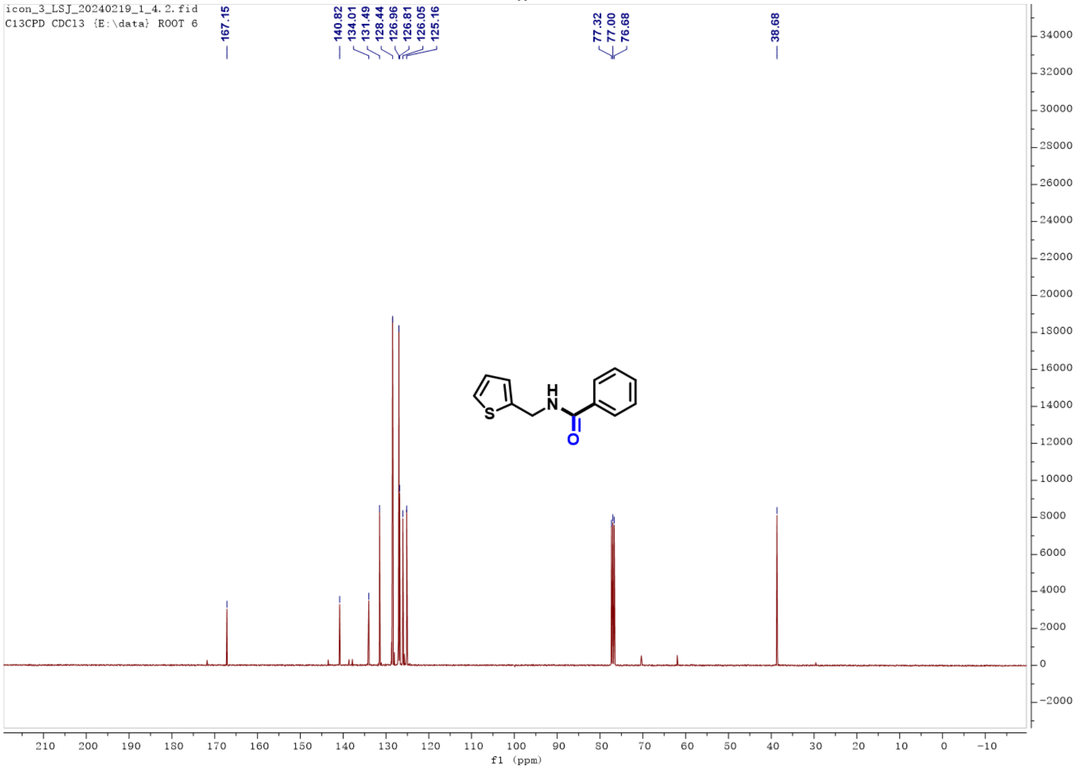

3aj *N*-benzylbenzamide

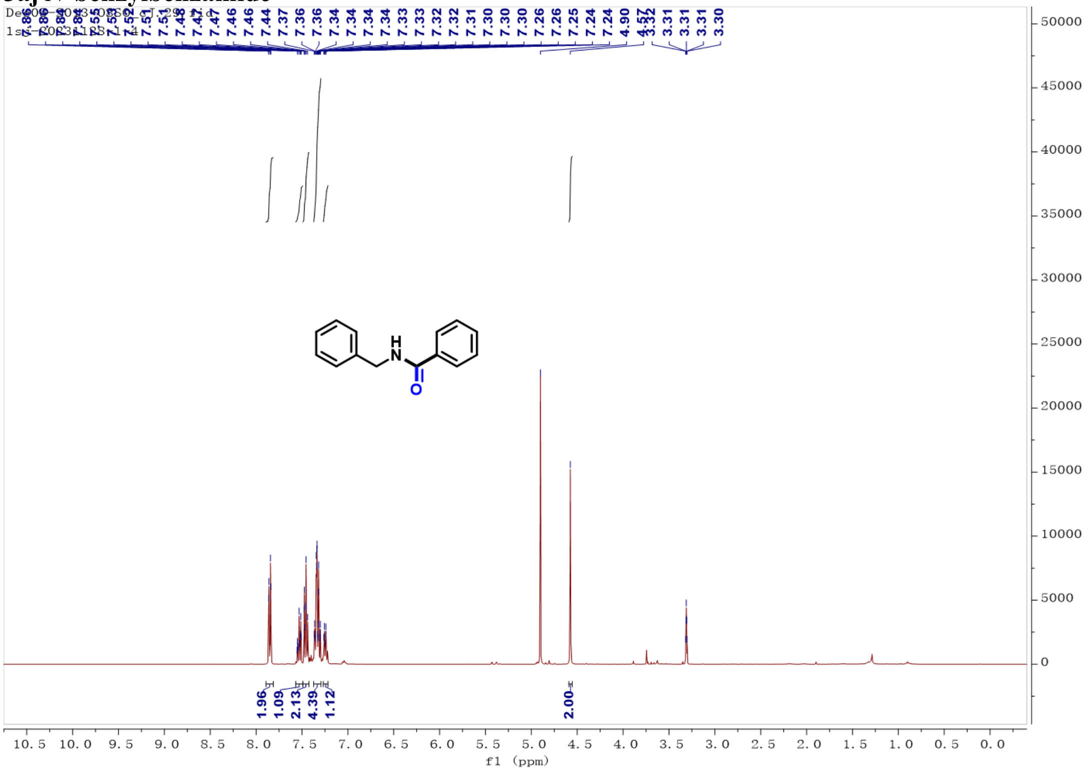

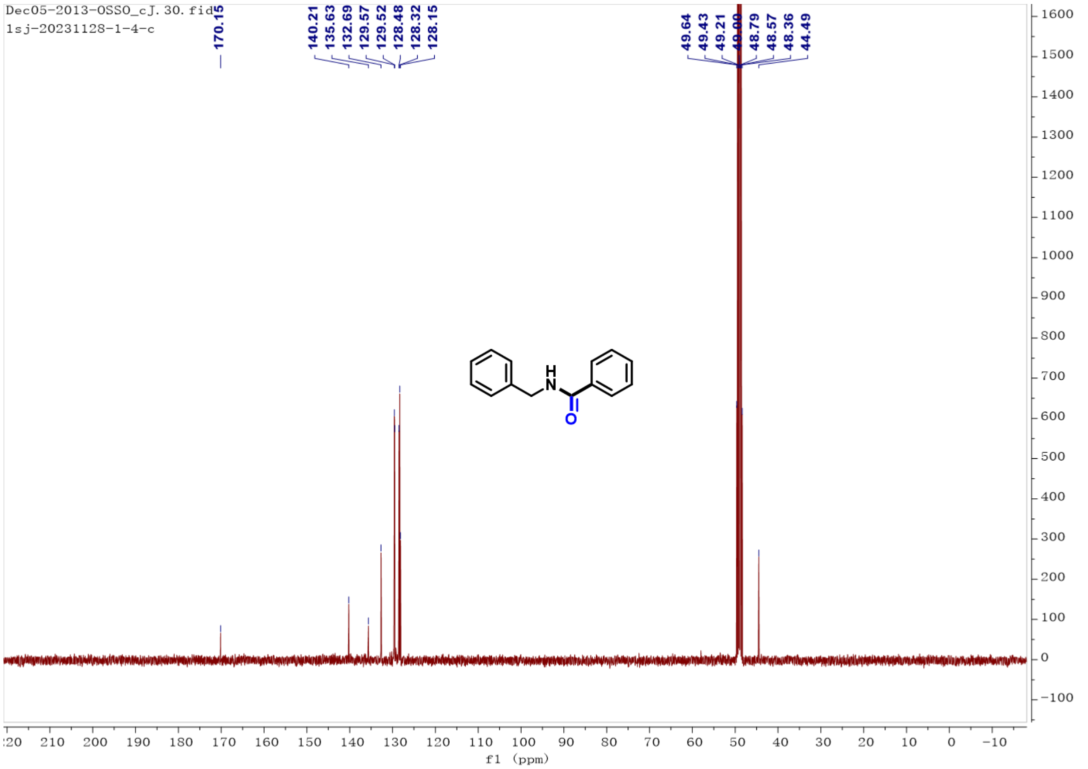

3ak *N*-(1-(p-tolyl)ethyl)benzamide

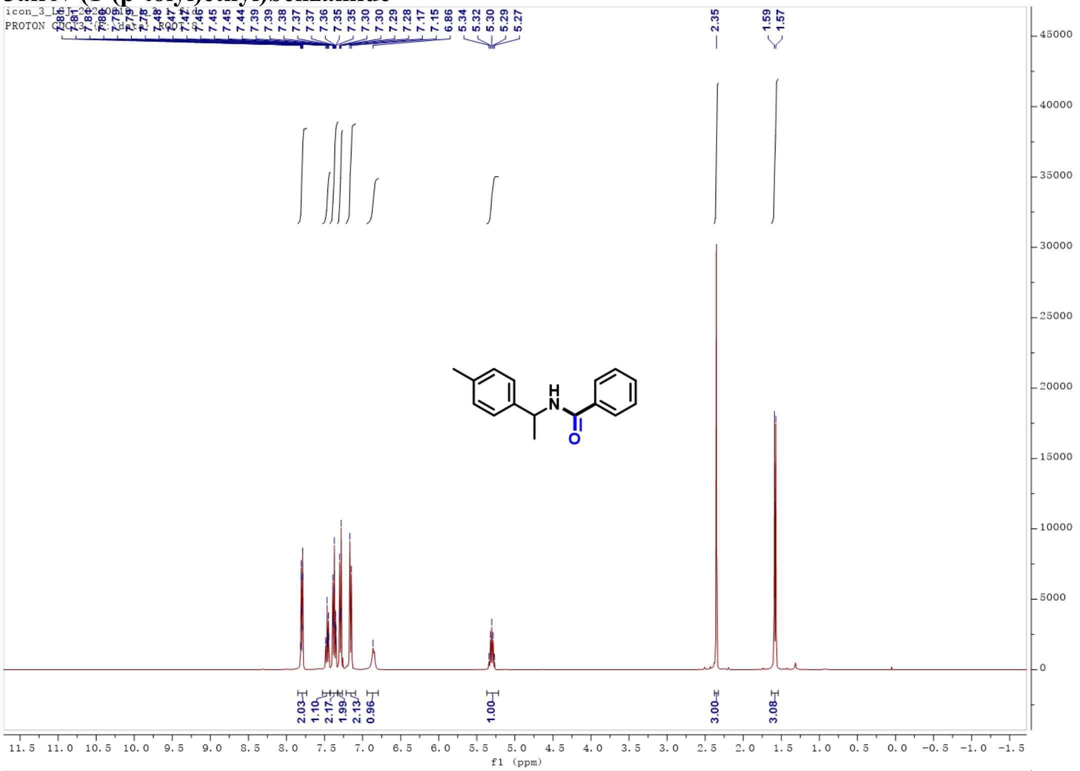

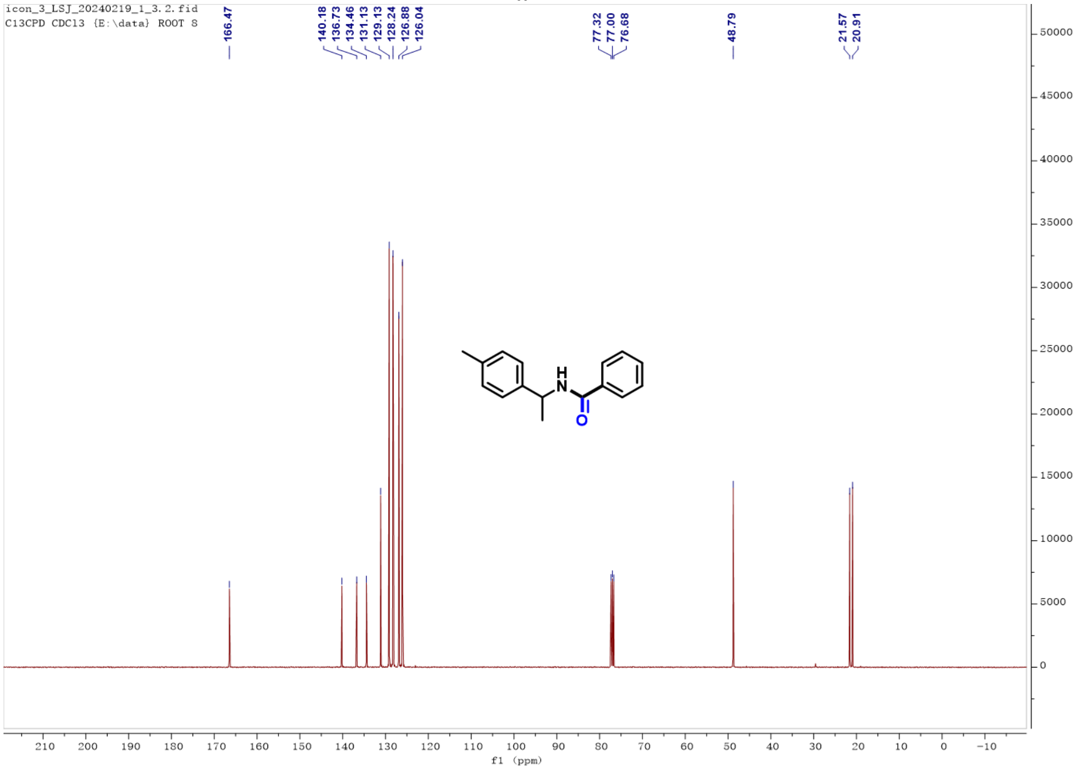

3al *N*-morpholinobenzamide

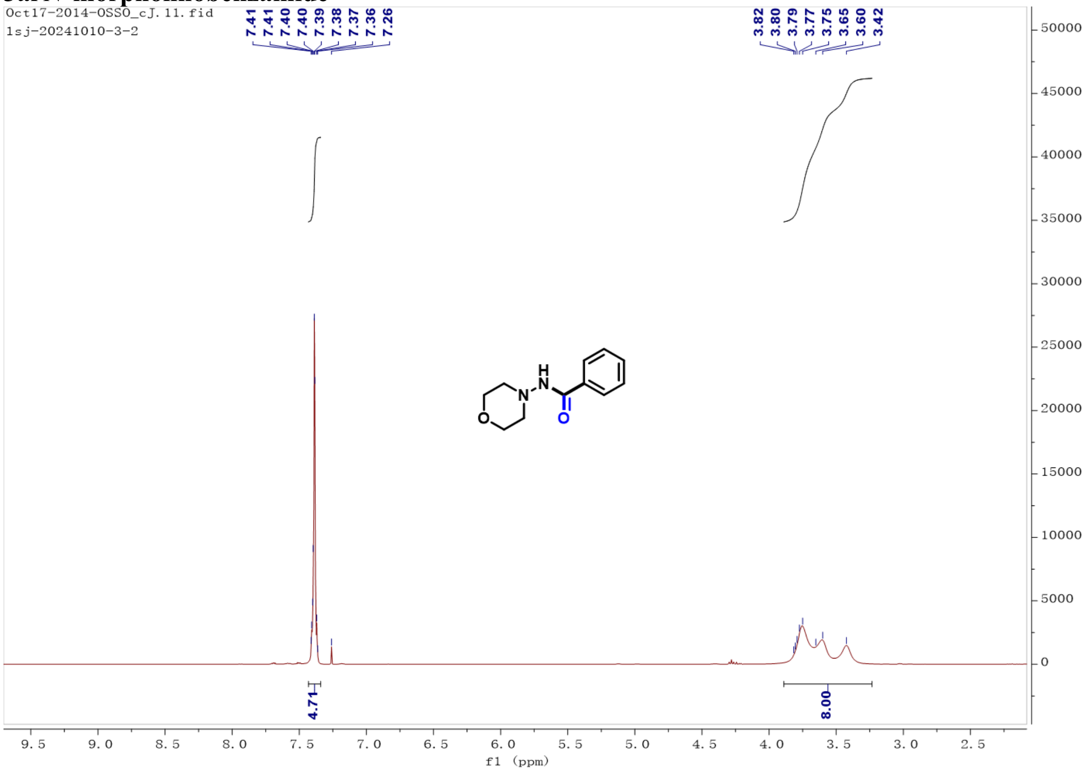

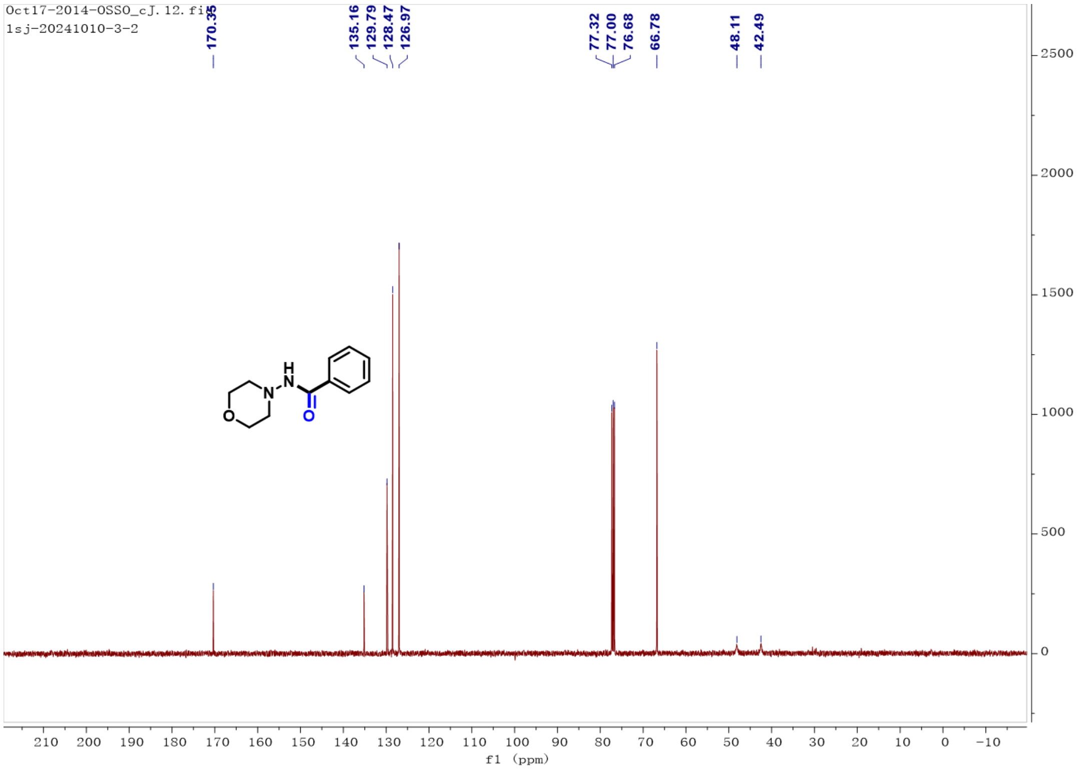

3am *N*-(2-(cyclohex-1-en-1-yl)ethyl)benzamide

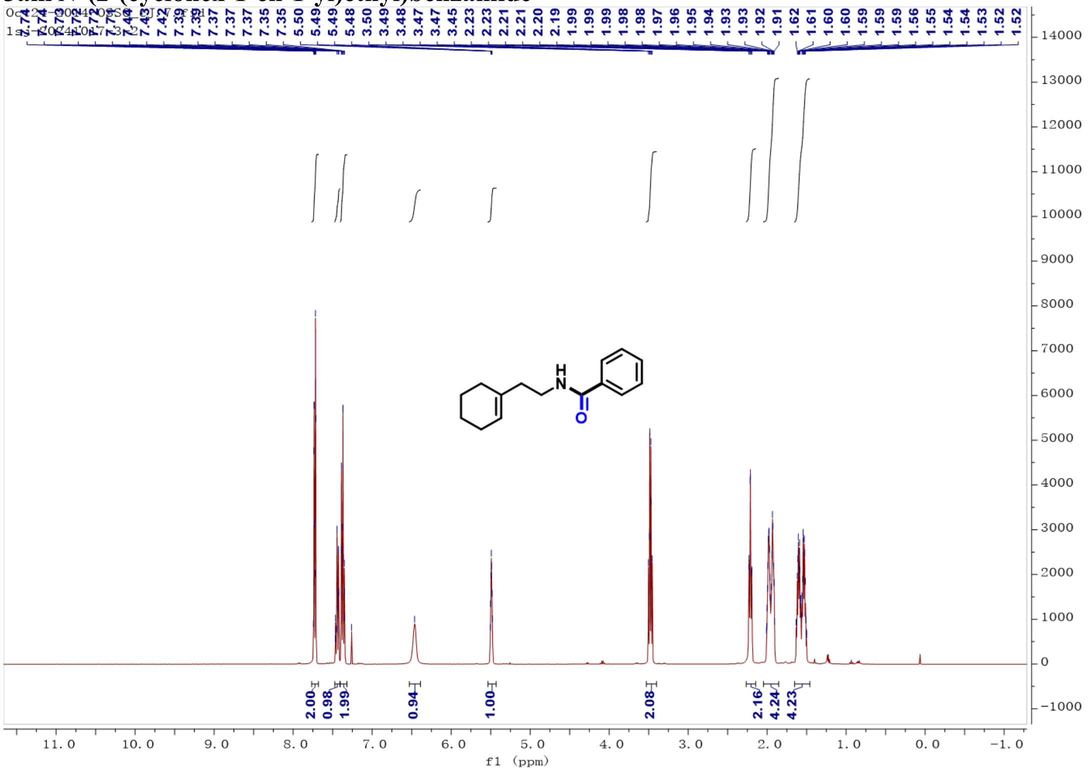

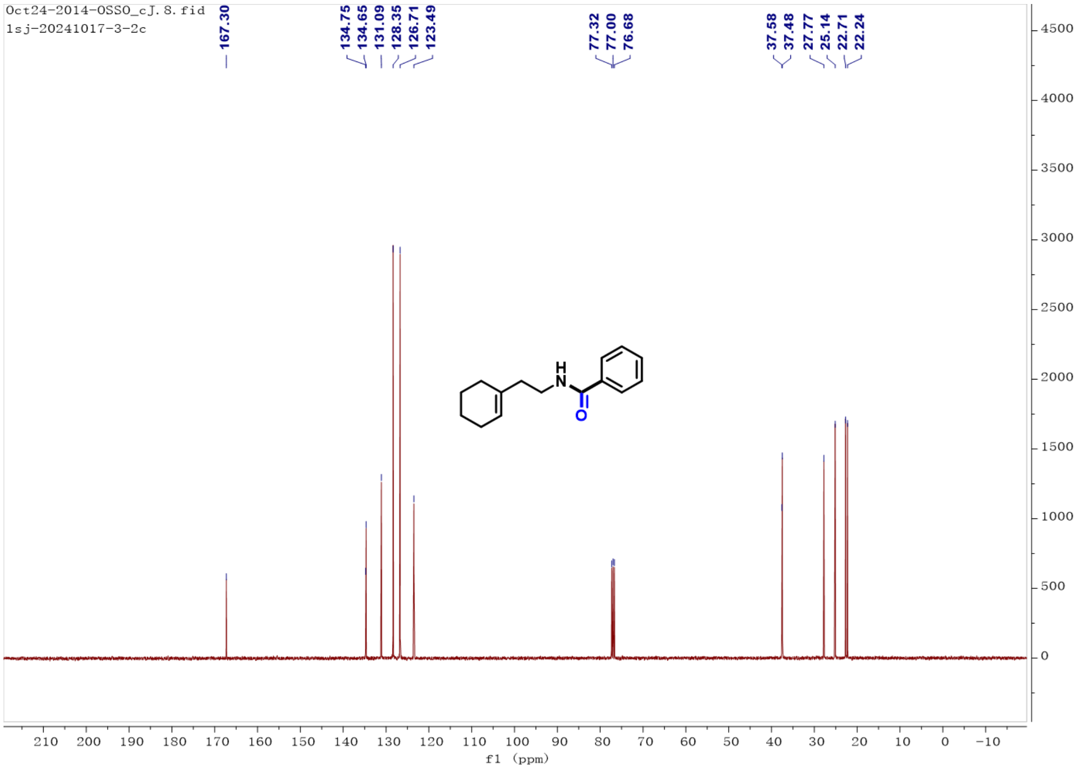

3an morpholino(*p*-tolyl)methanone

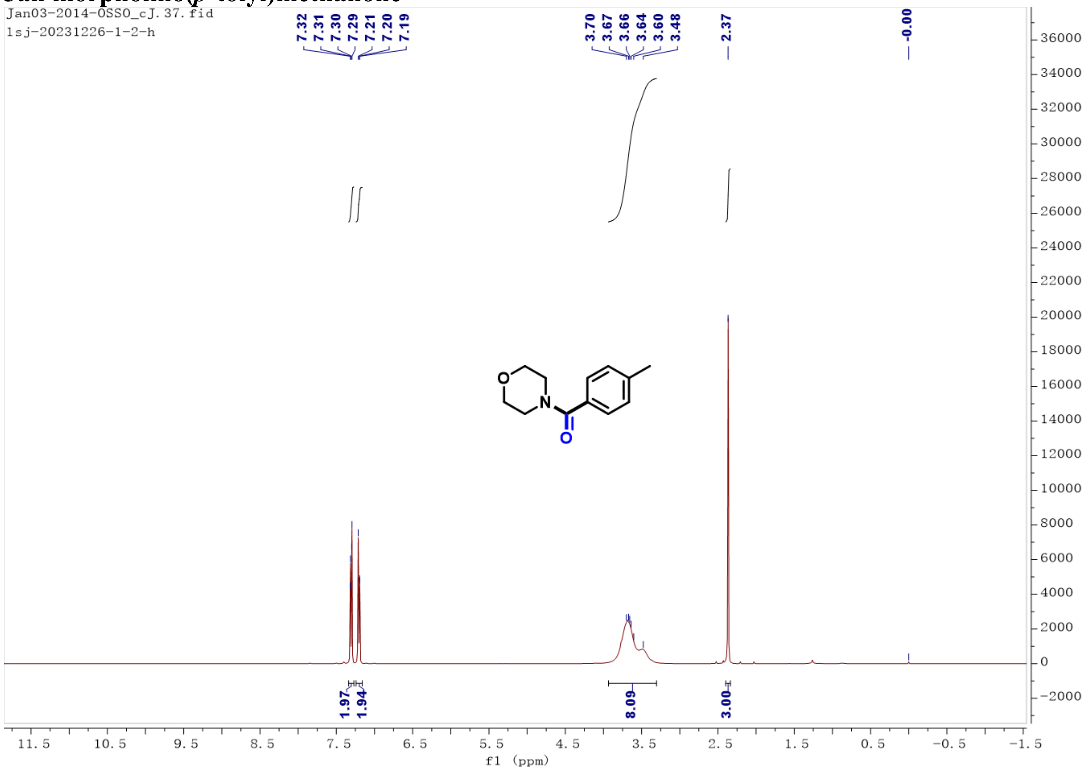

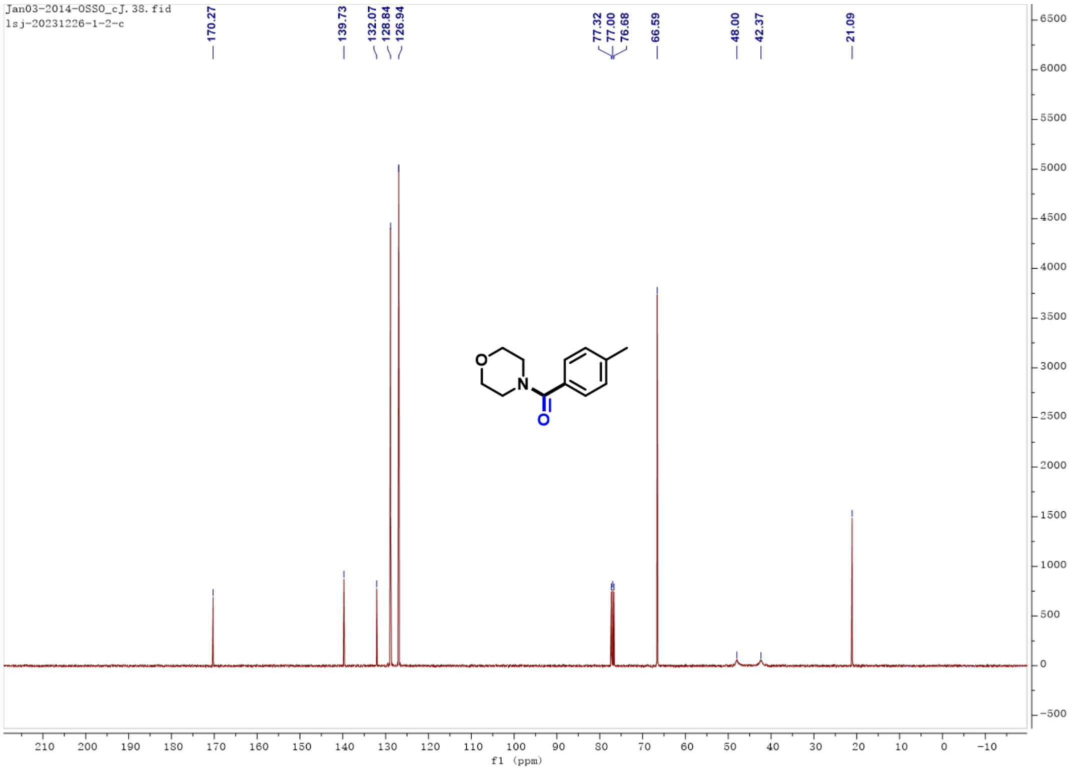

3ao (4-methoxyphenyl)(morpholino)methanone

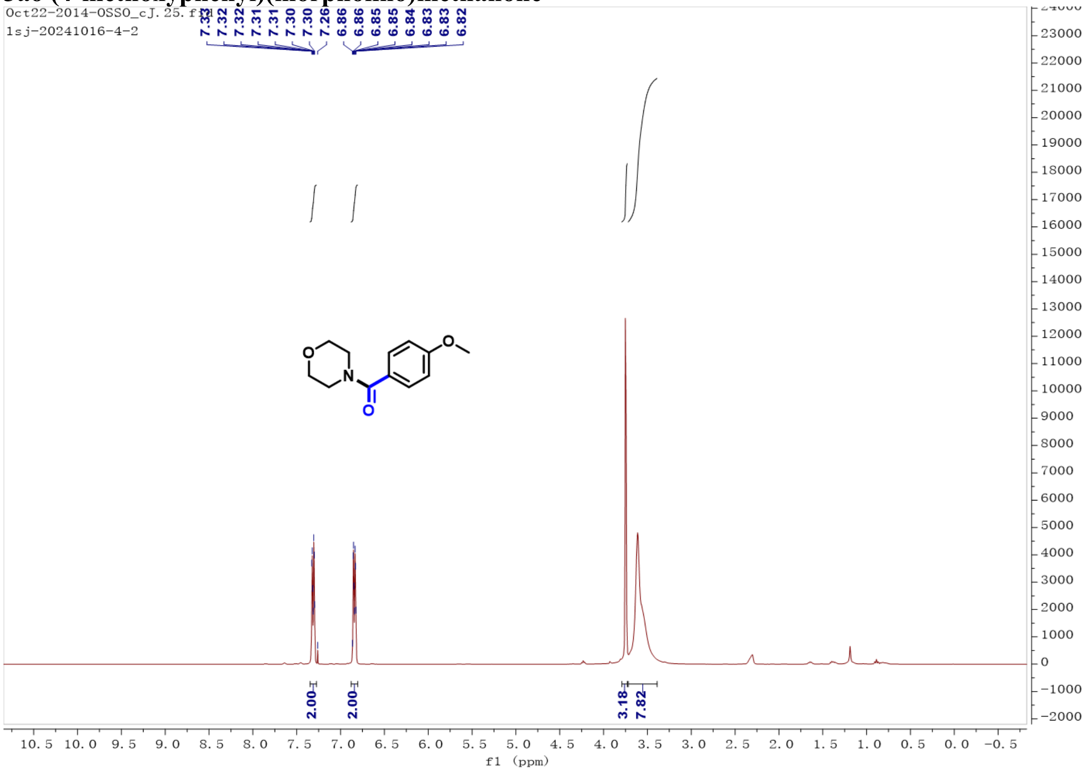

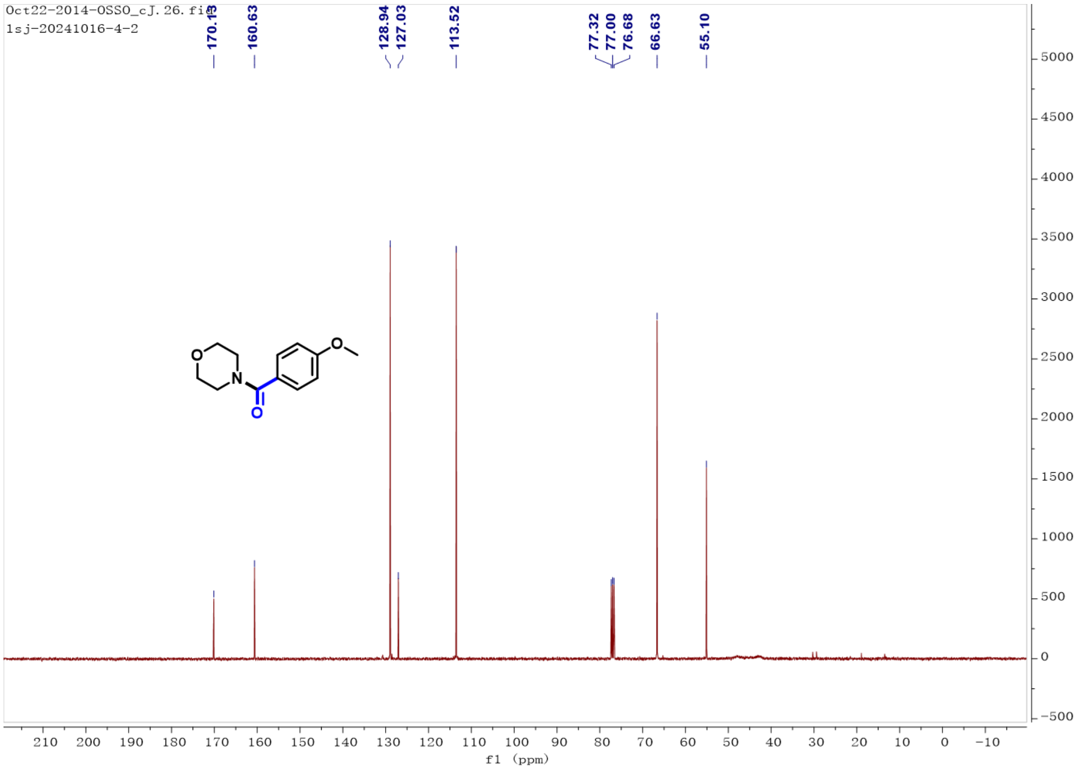

3ap [1,1'-biphenyl]-4-yl(morpholino)methanone

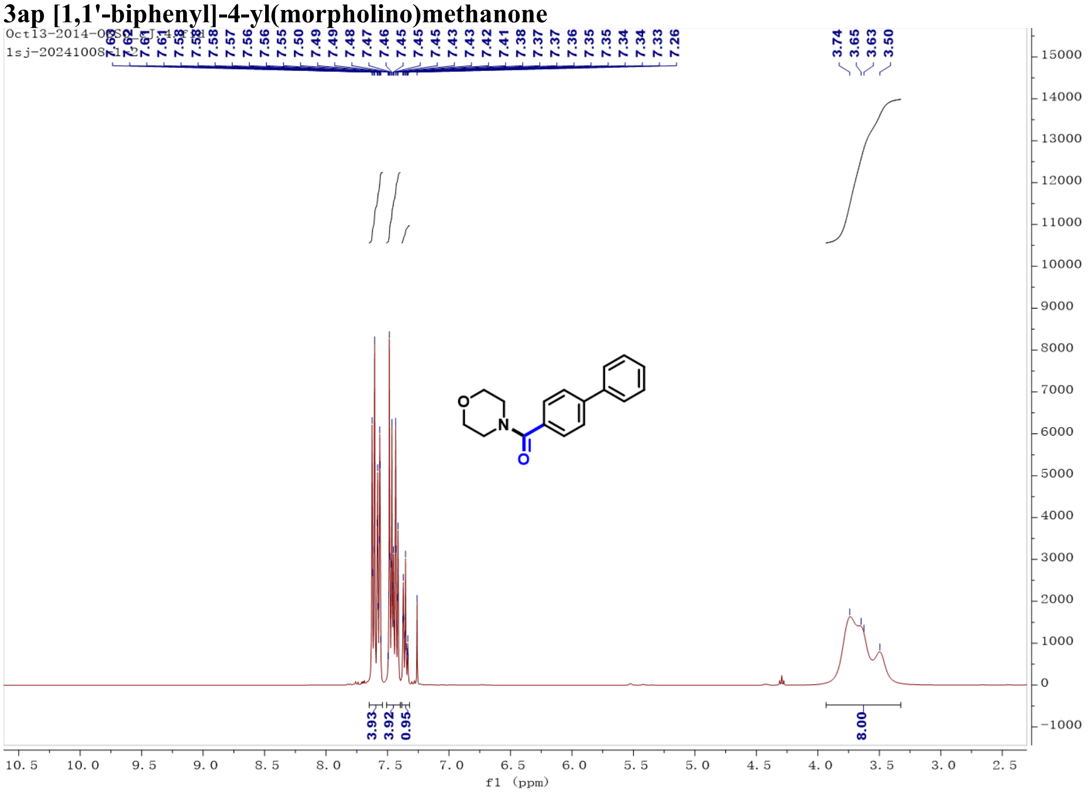

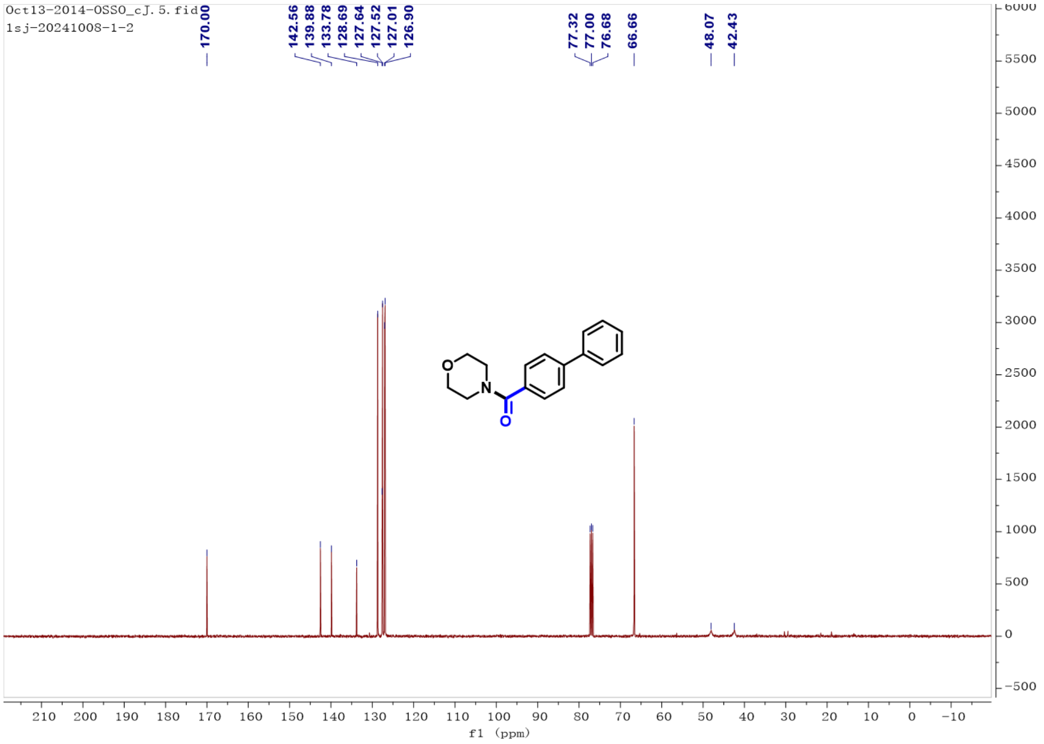

3aq (4-(hydroxymethyl)phenyl)(morpholino)methanone

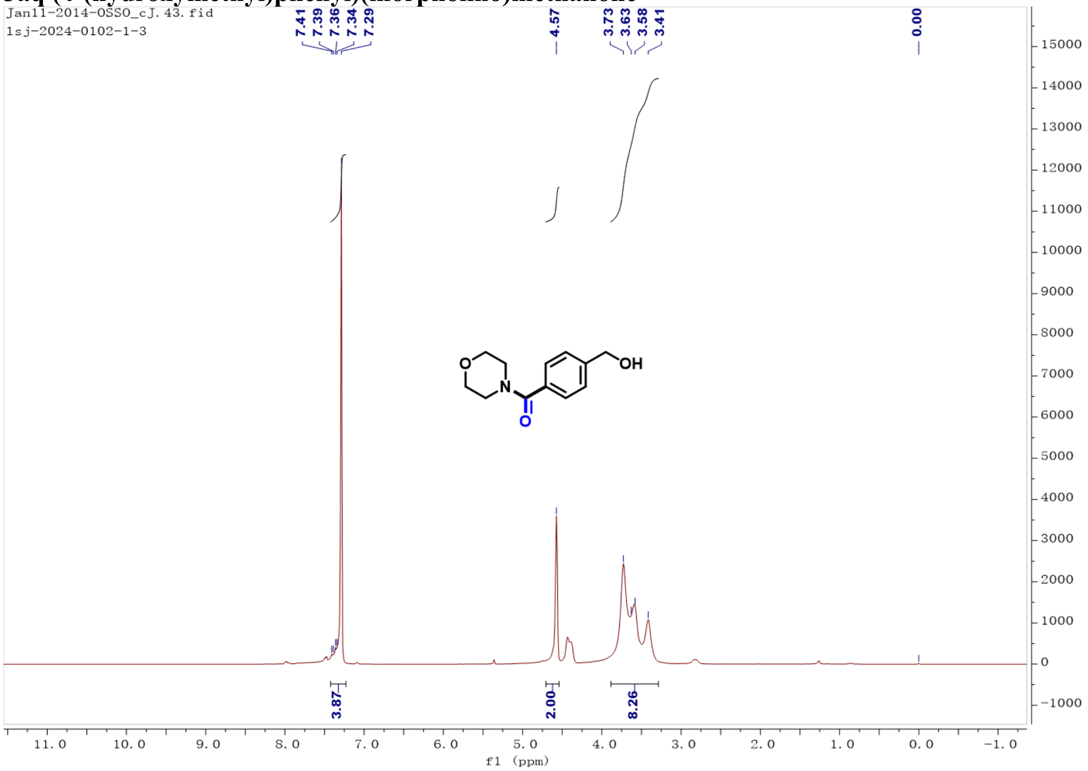

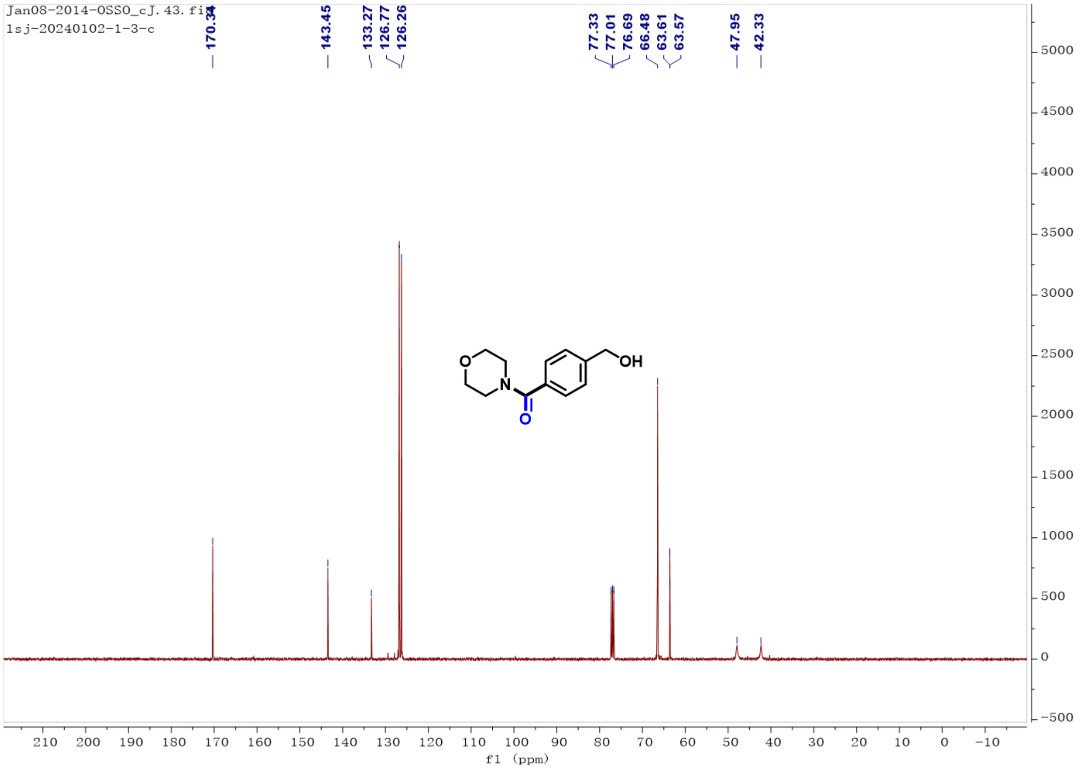

3ar (4-fluorophenyl)(morpholino)methanone

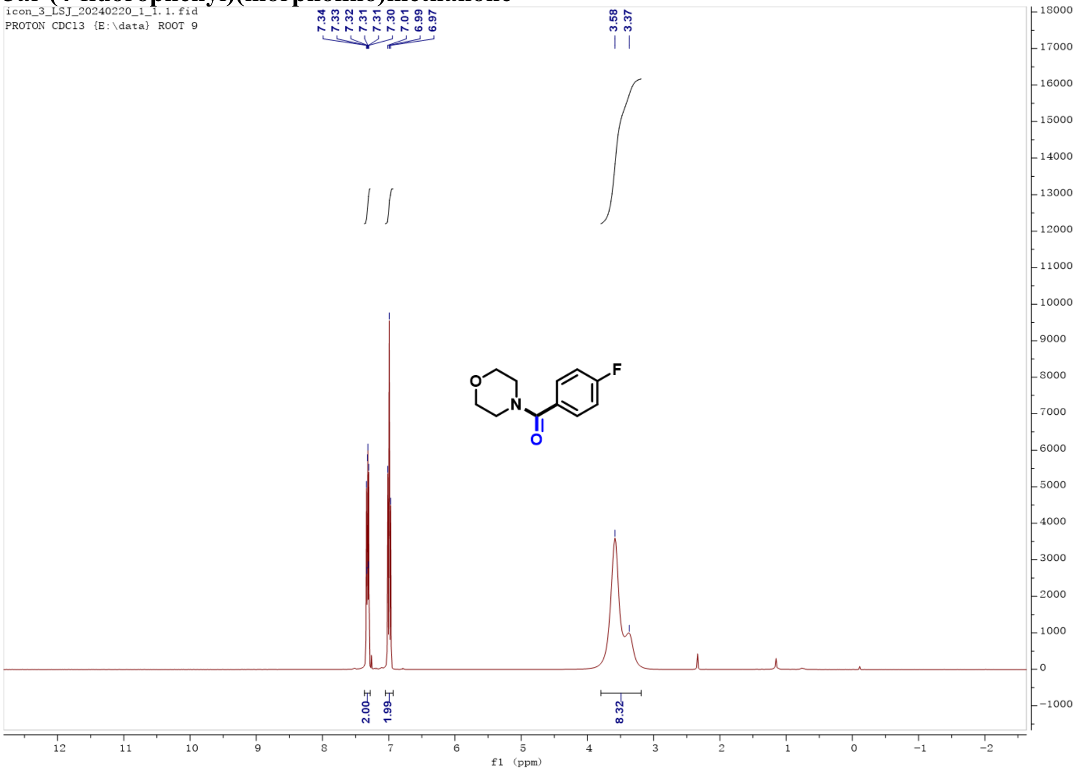

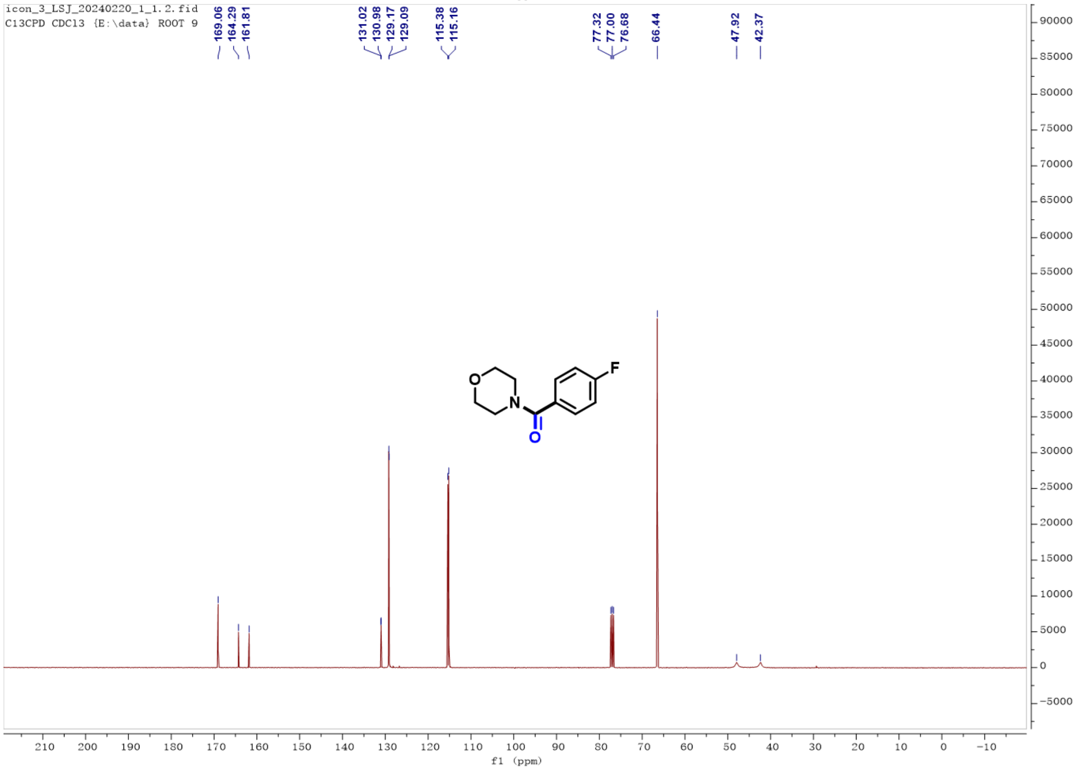

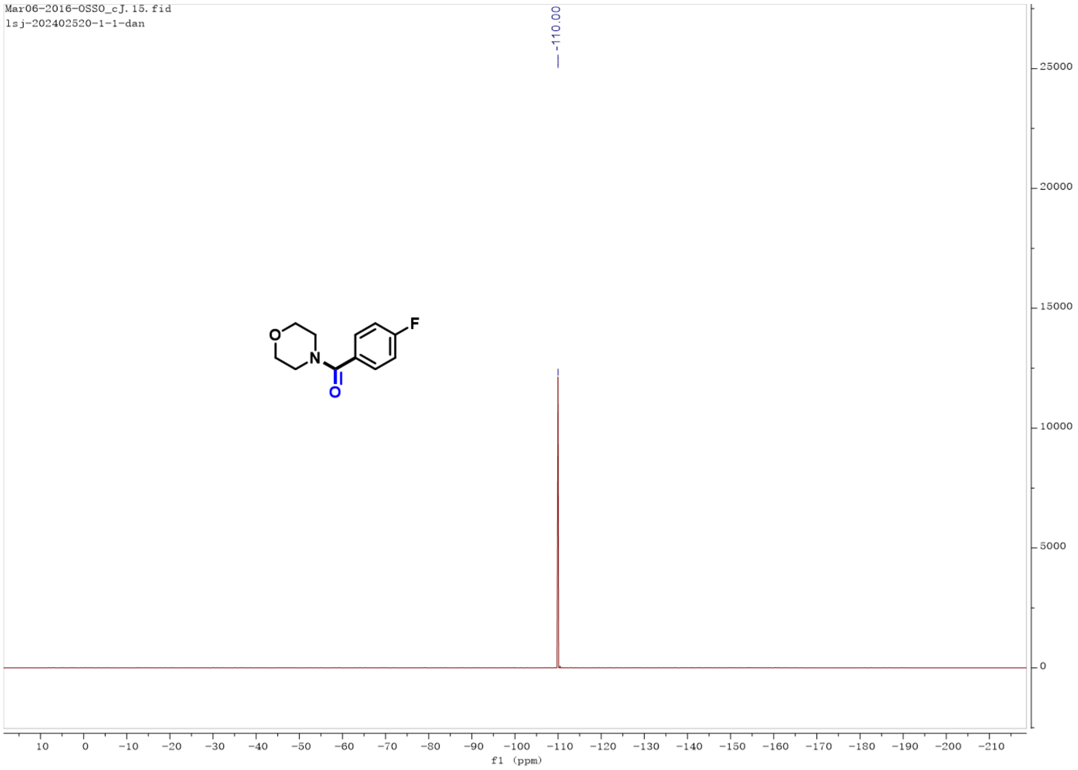

Mar06-2016-OSSO_cJ.15.fid
lsj-202402520-1-1-dan
-110.00
O
N
O
F
25000
20000
15000
10000
5000
0
10
0
-10
-20
-30
-40
-50
-60
-70
-80
-90
-100
-110
-120
-130
-140
-150
-160
-170
-180
-190
-200
-210
f1 (ppm)

3as (4-chlorophenyl)(morpholino)methanone

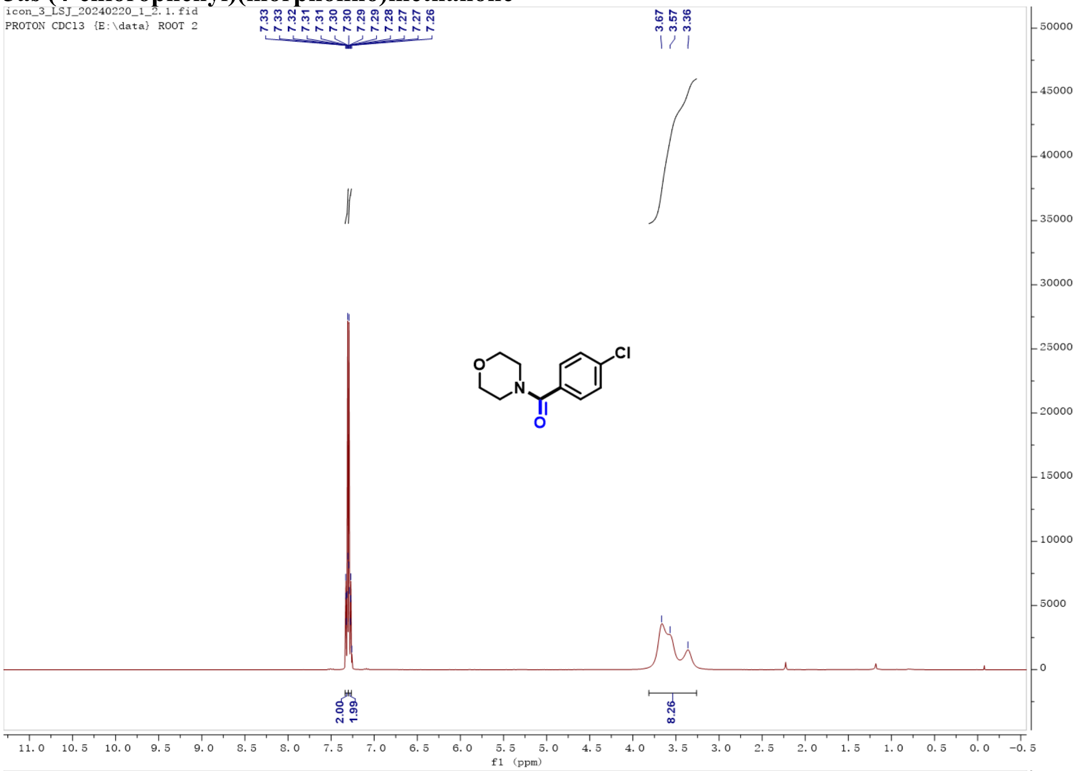

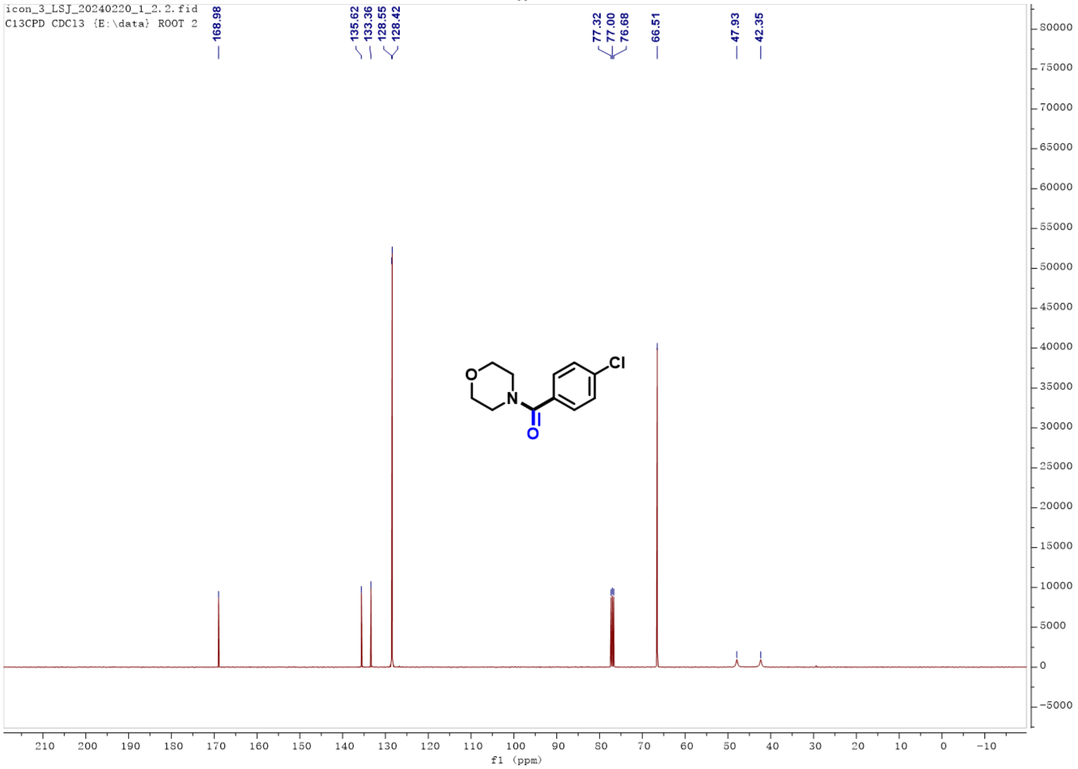

3at (4-bromophenyl)(morpholino)methanone

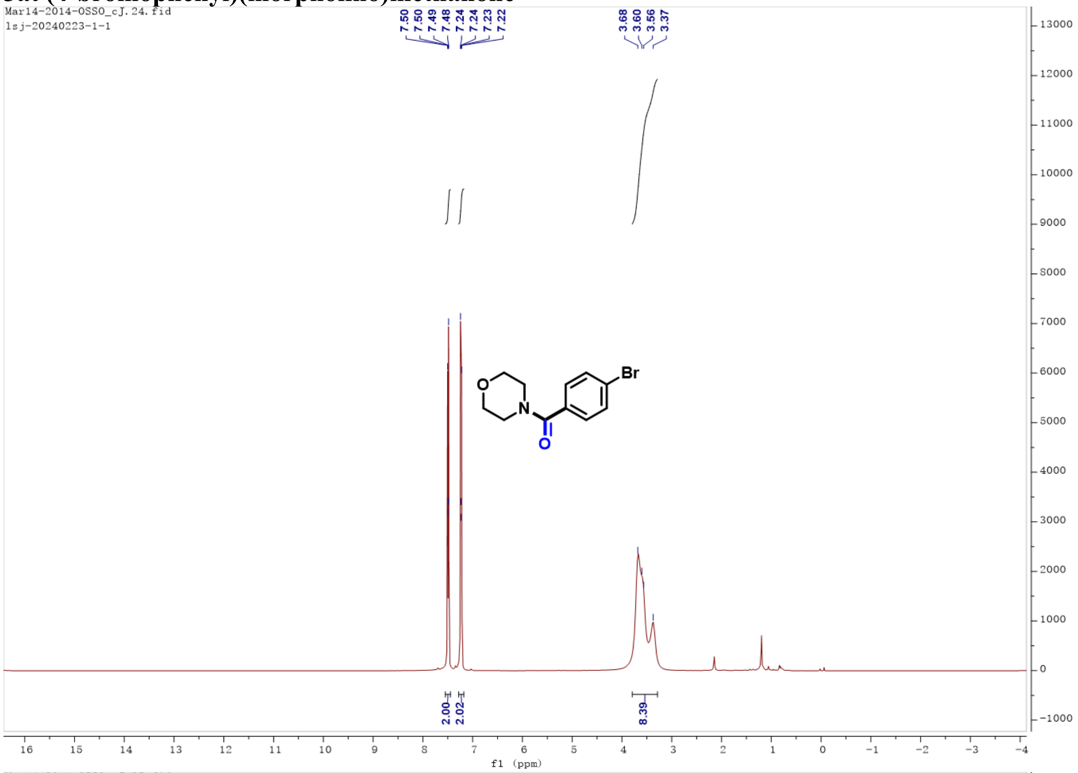

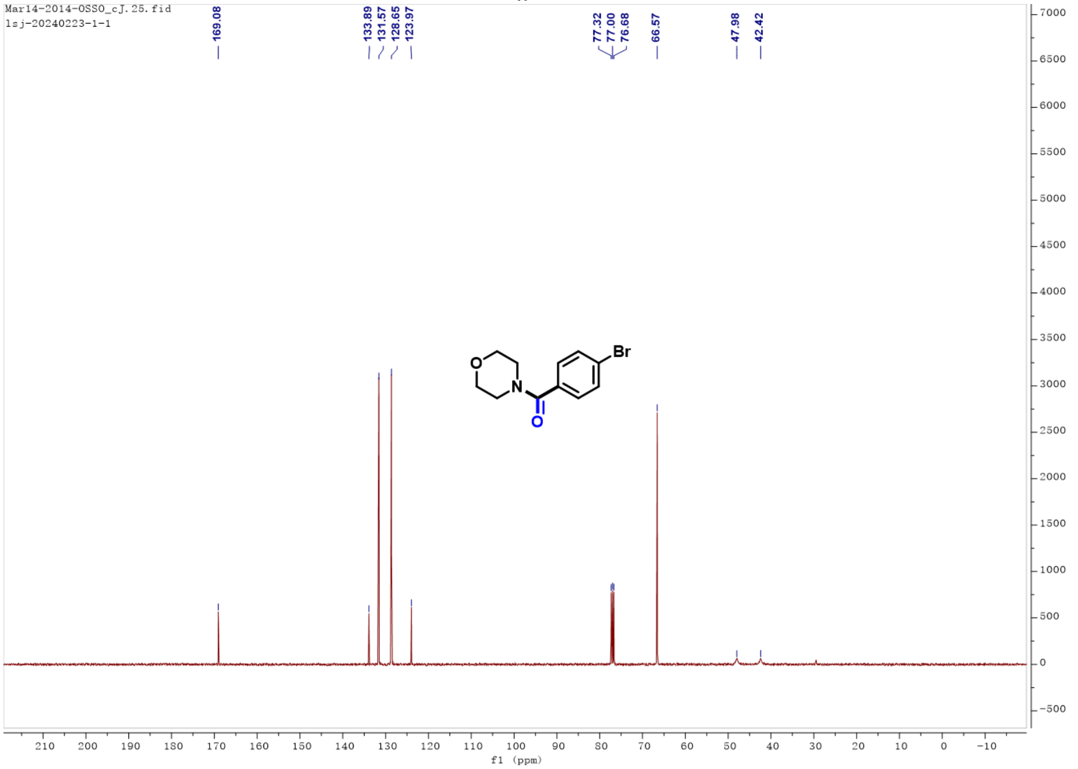

3au 4-(morpholine-4-carbonyl)benzonitrile

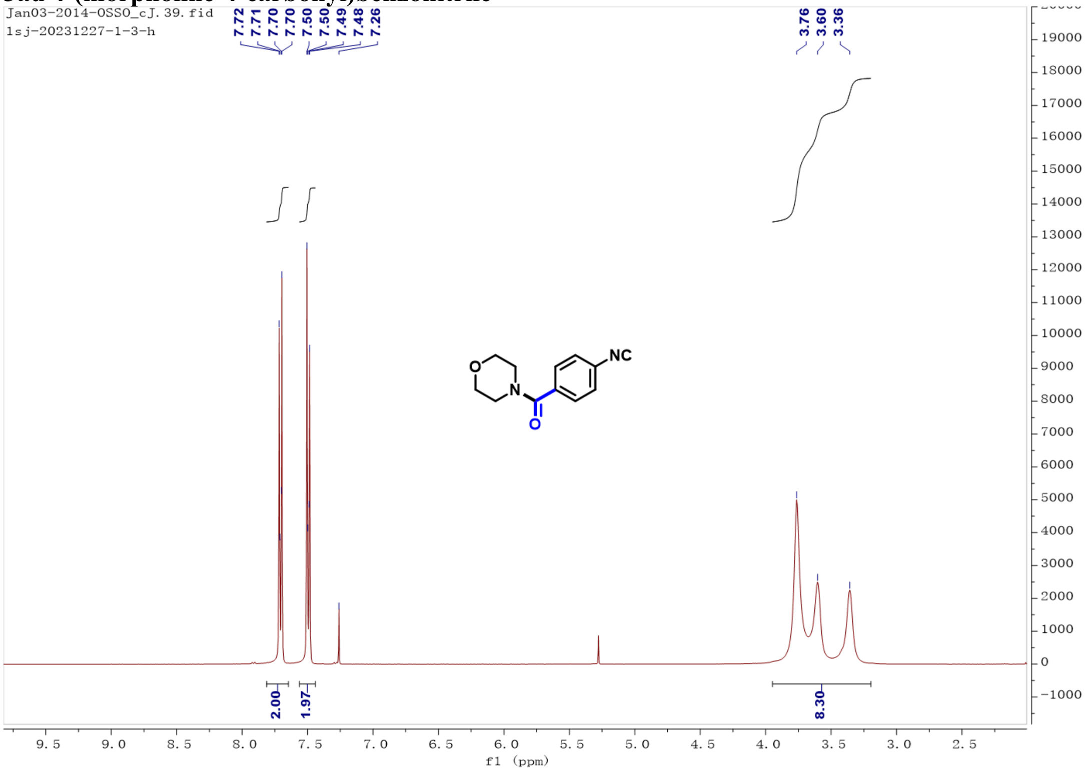

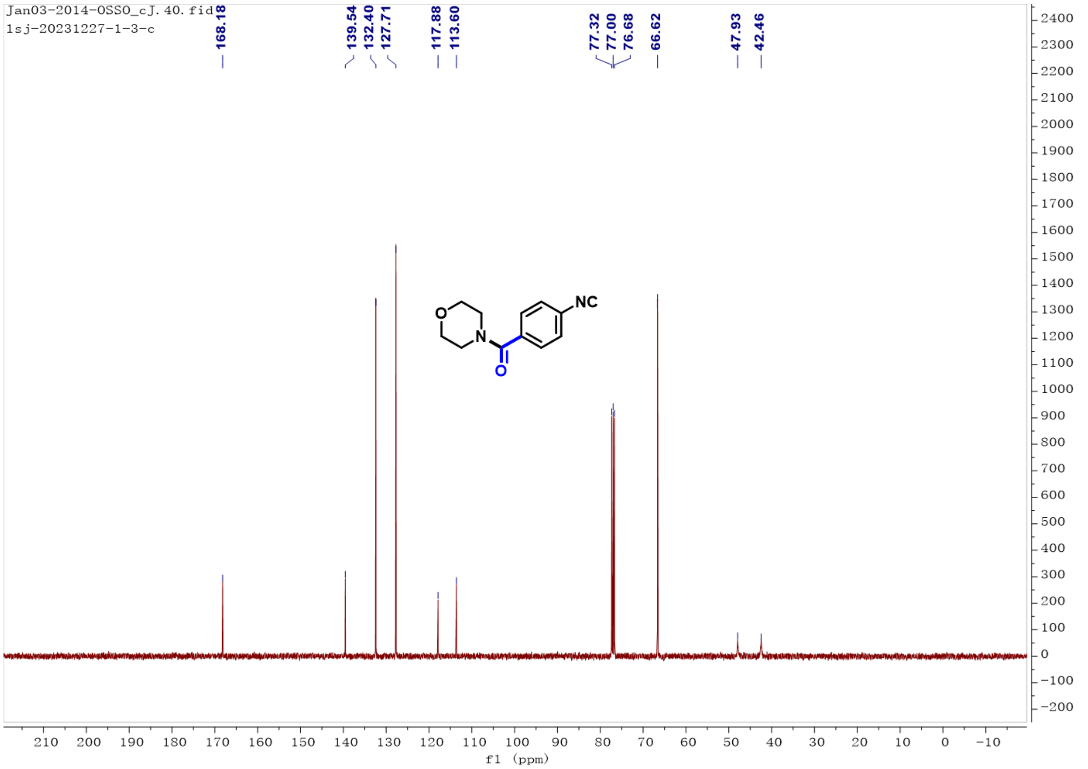

3av morpholino(4-nitrophenyl)methanone

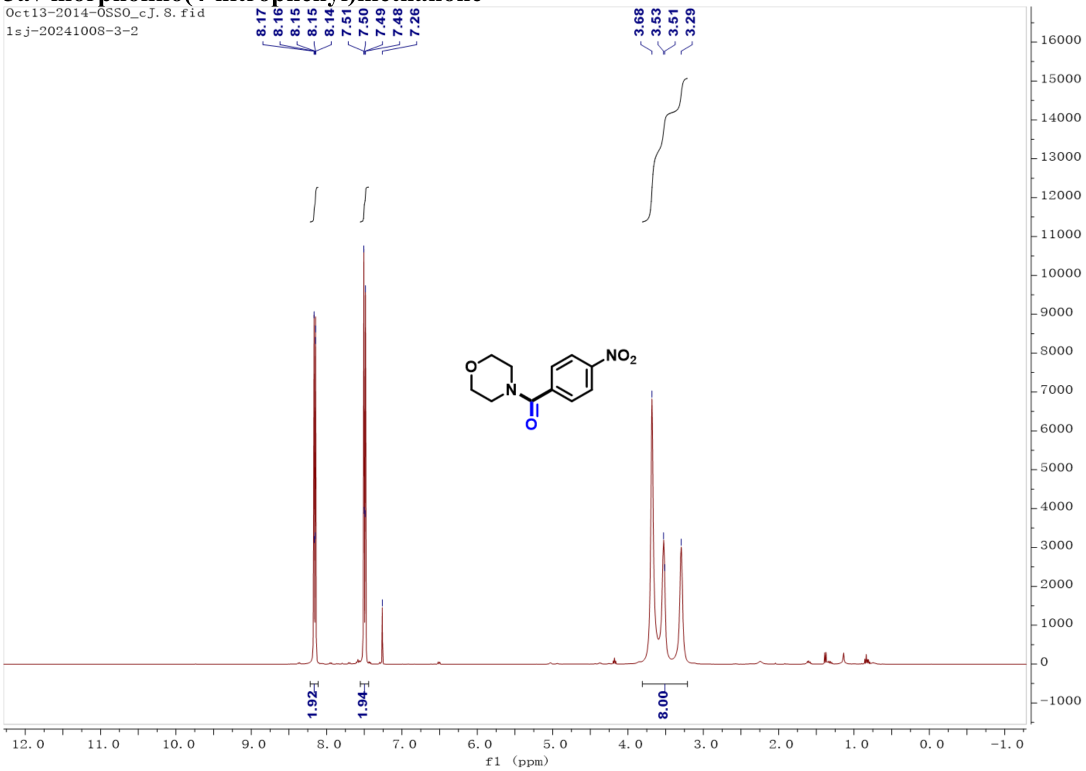

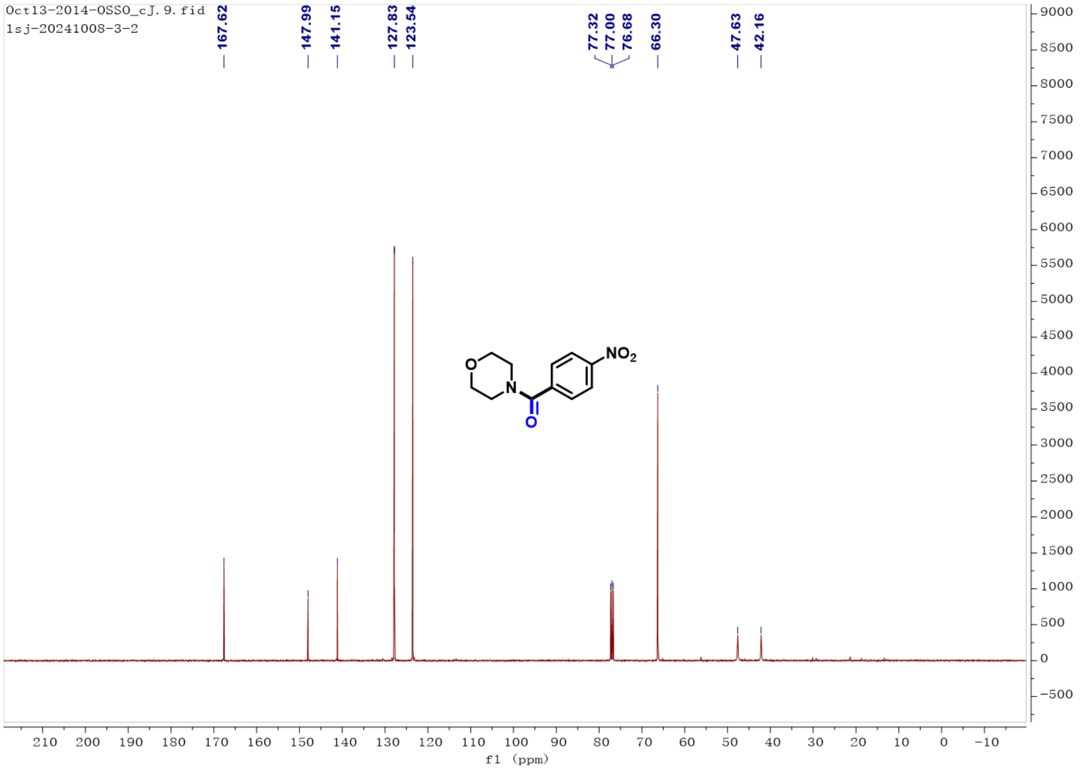

3aw morpholino(4-vinylphenyl)methanone

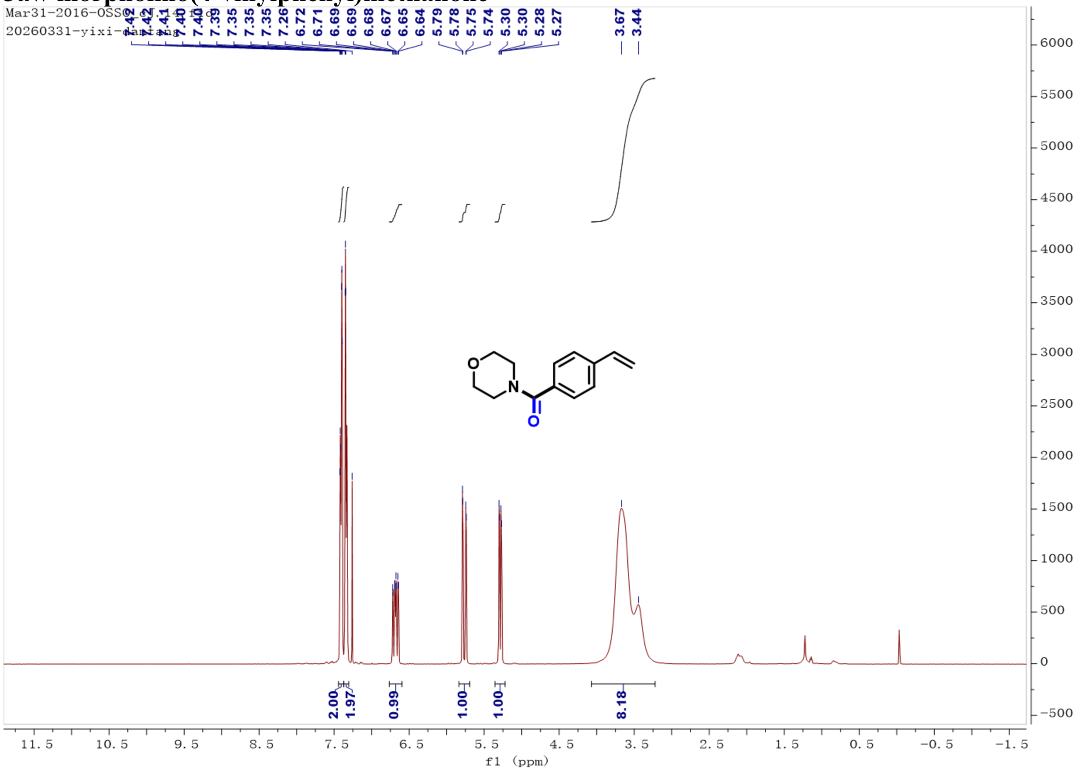

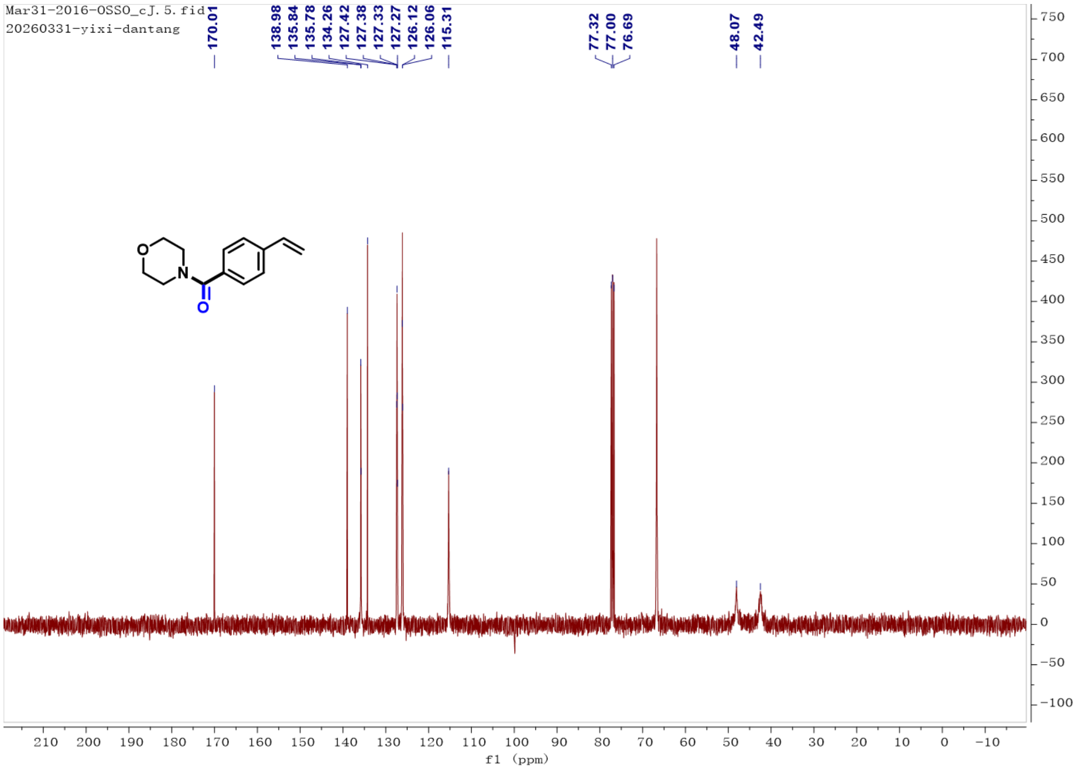

3ax morpholino(4-(prop-1-en-2-yl)phenyl)methanone

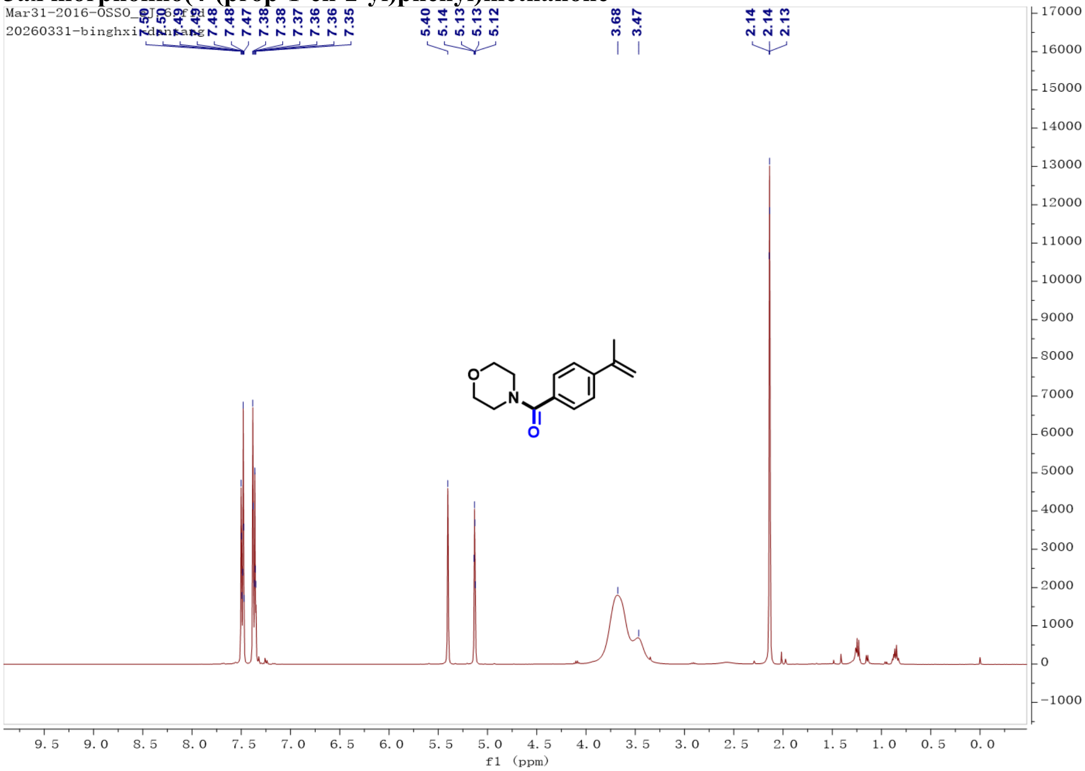

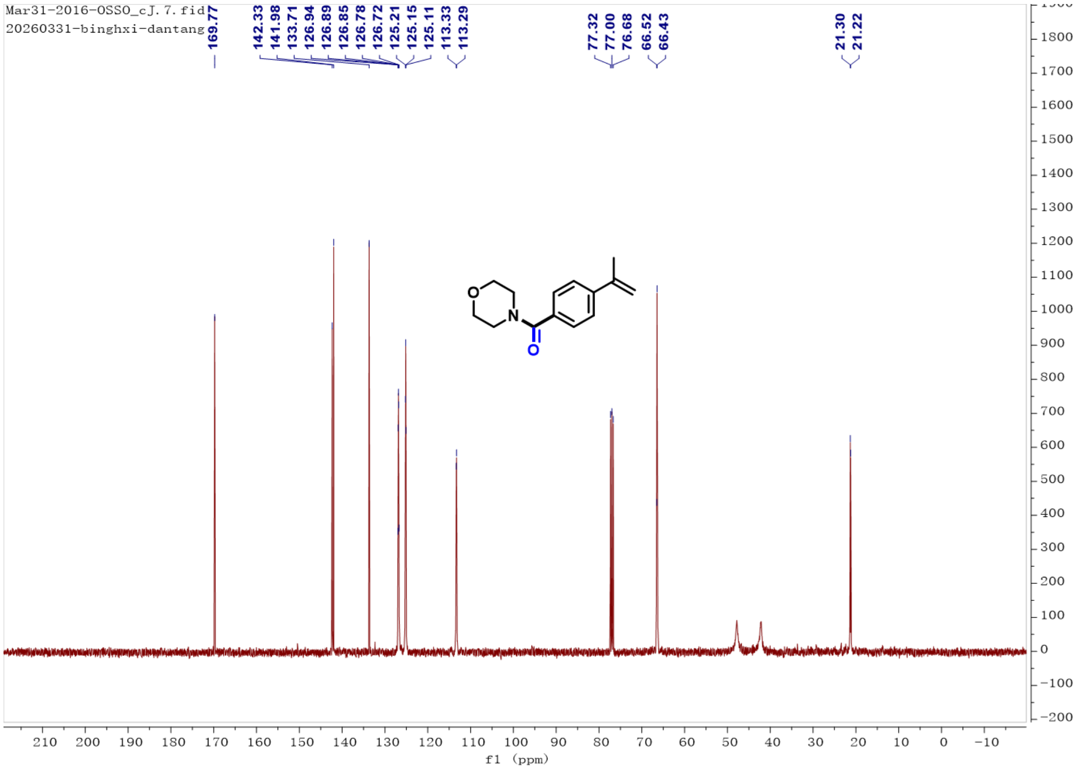

3aw morpholino(m-tolyl)methanone

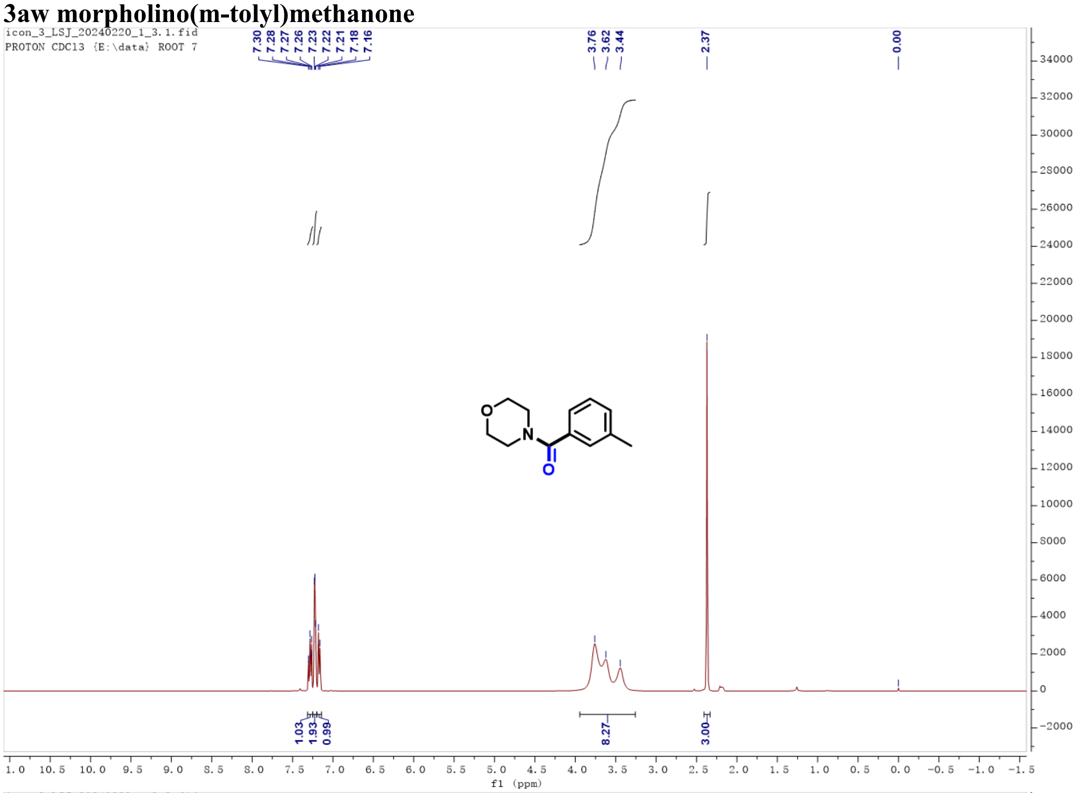

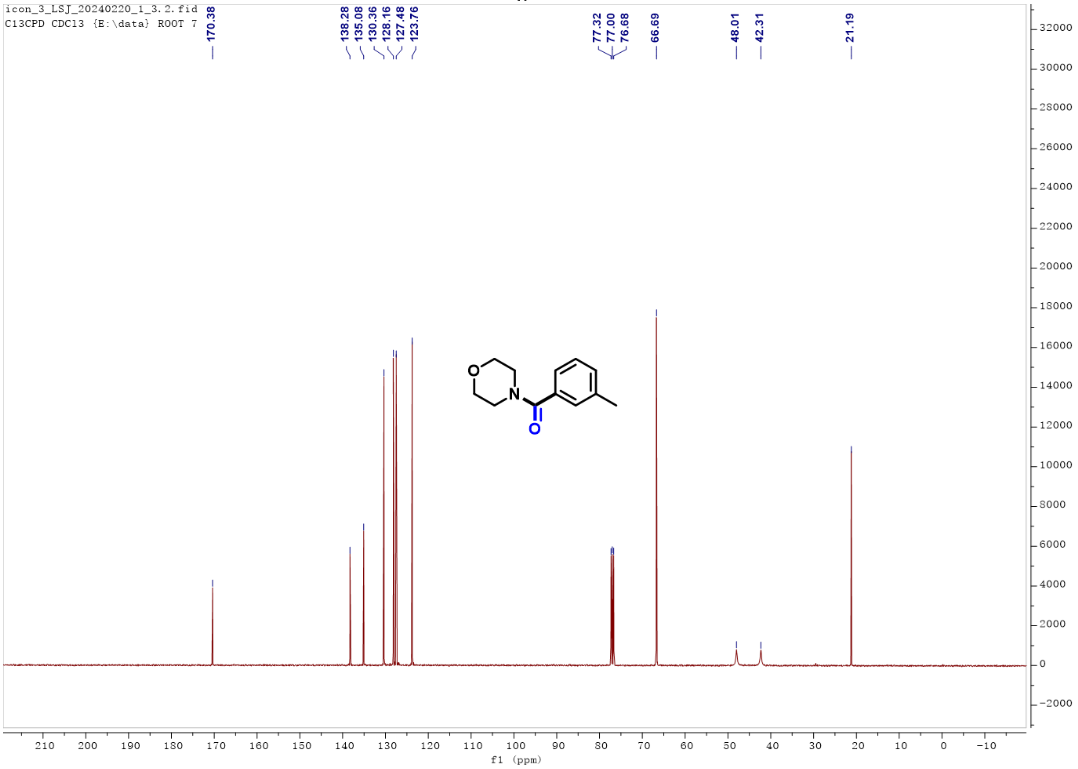

3ax (4-aminophenyl)(morpholino)methanone

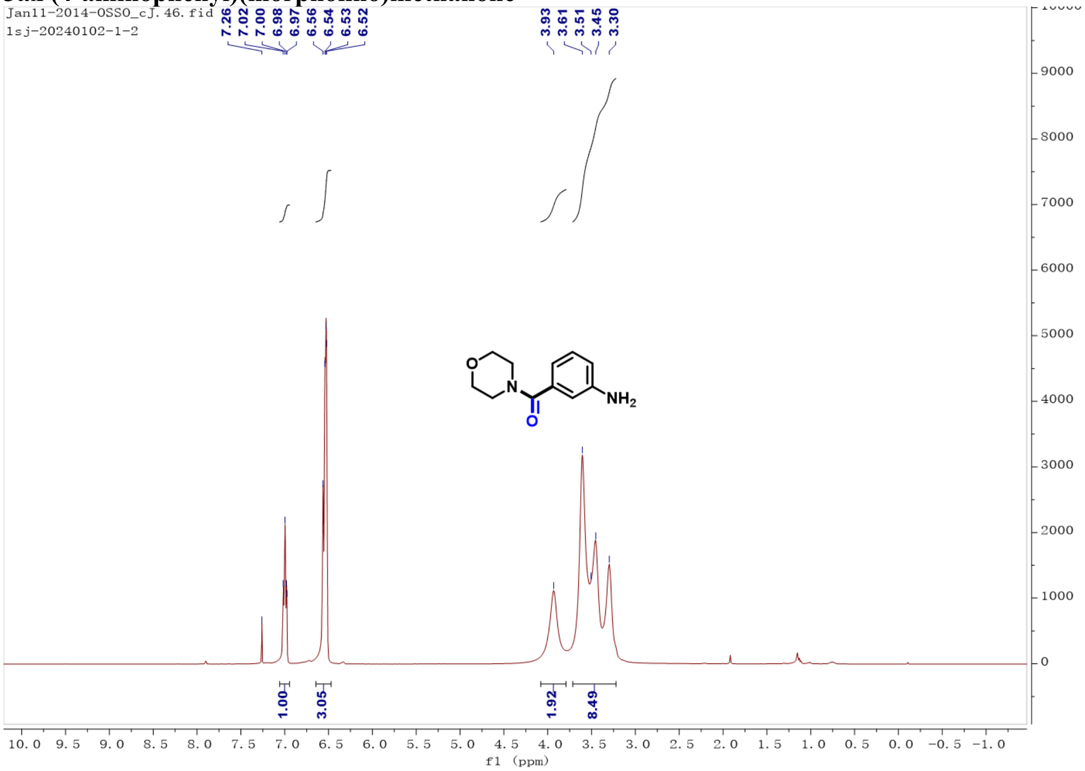

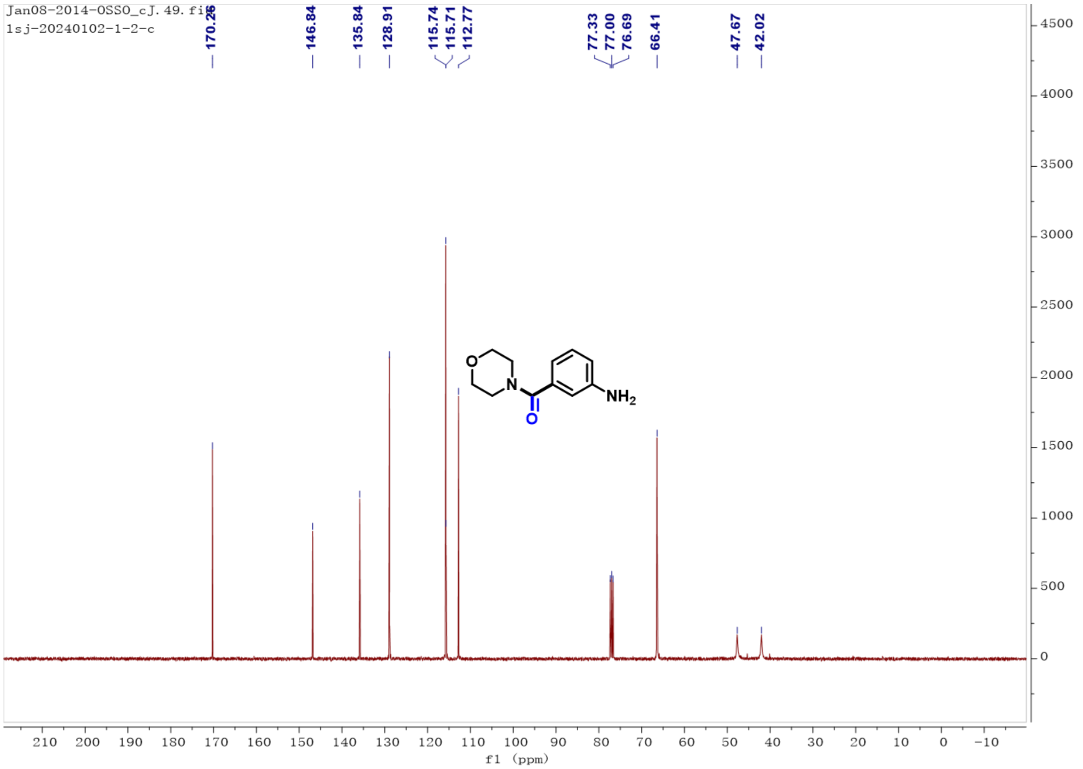

3ay (3-chlorophenyl)(morpholino)methanone

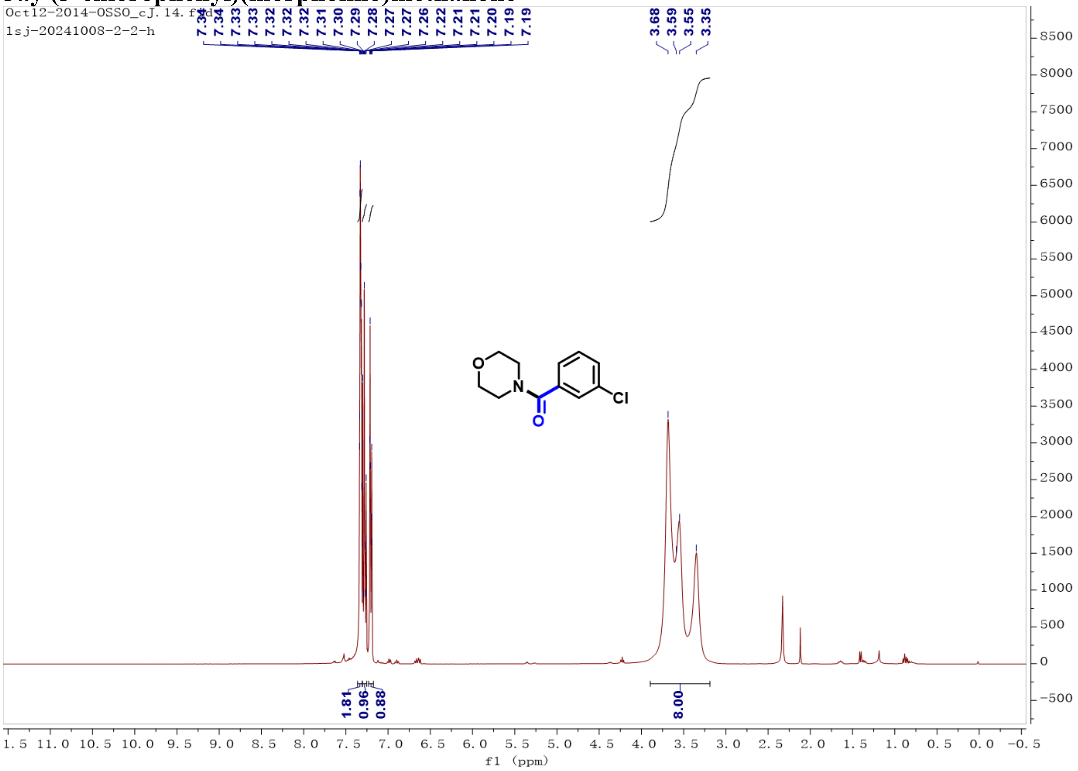

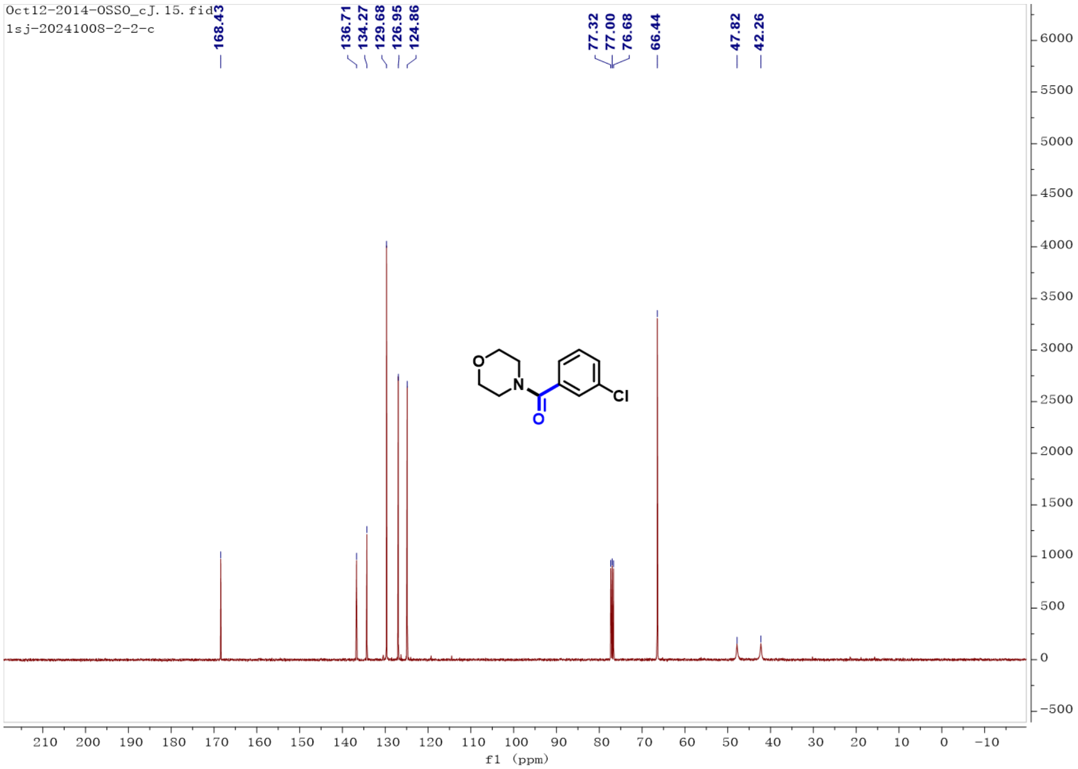

3az morpholino(pyridin-3-yl)methanone

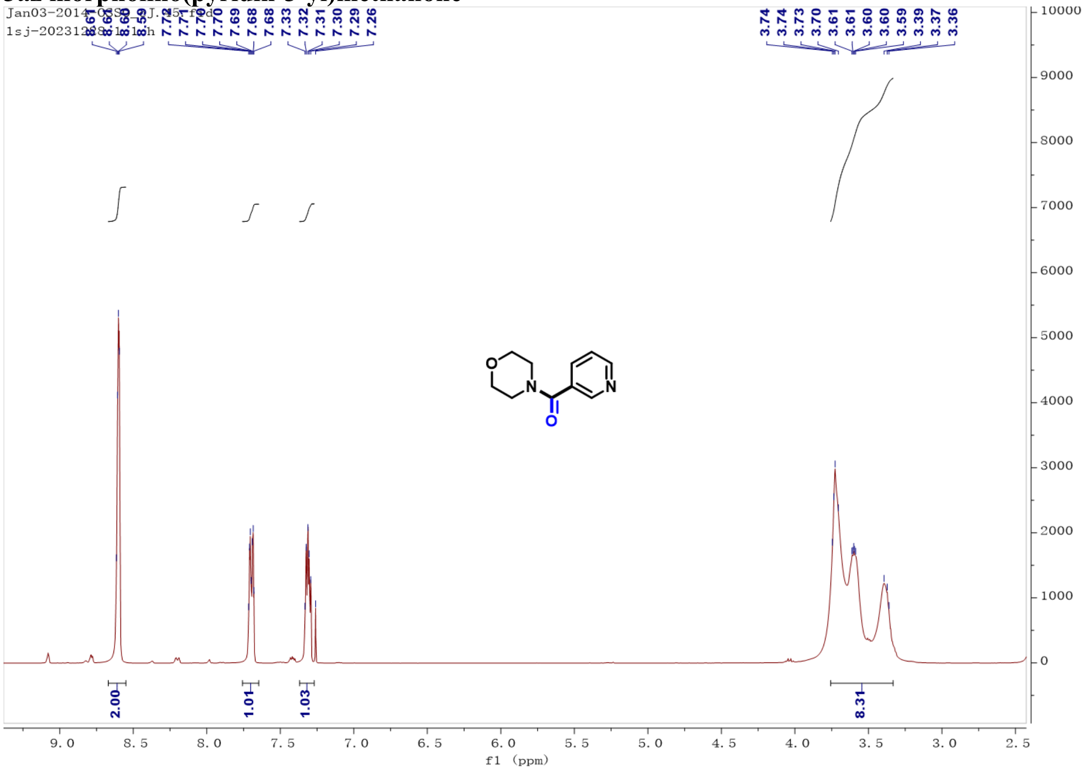

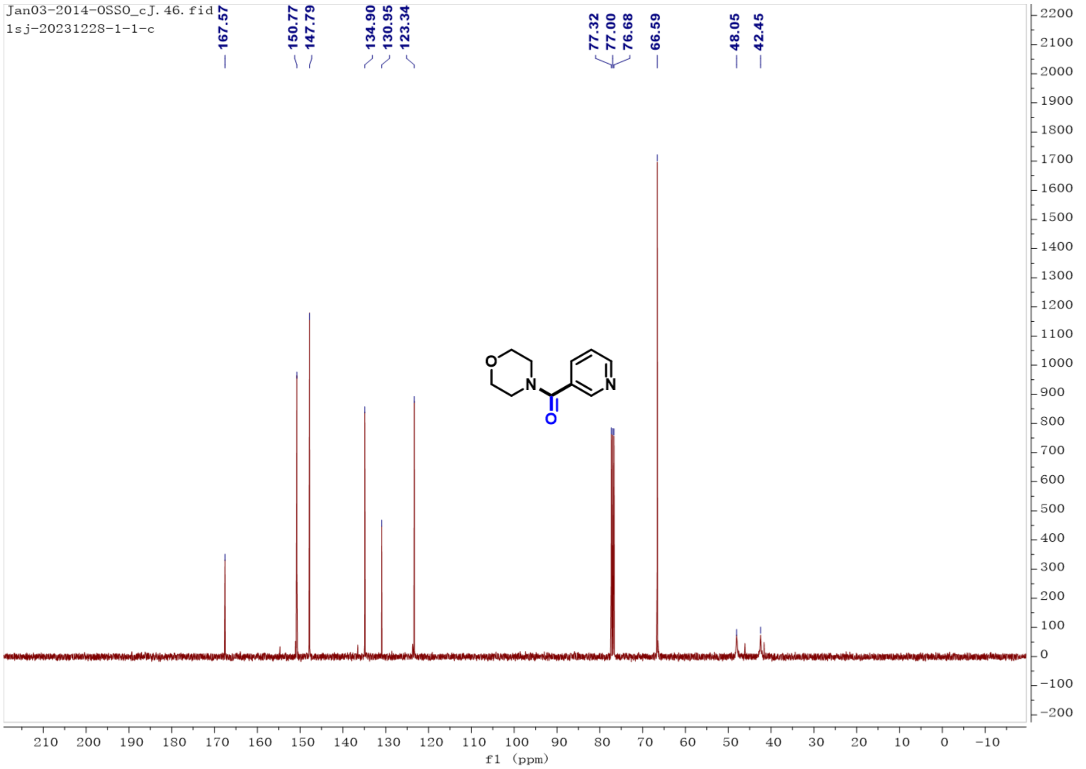

3ba (2,3-dihydrobenzo[*b*][1,4]dioxin-6-yl)(morpholino)methanone

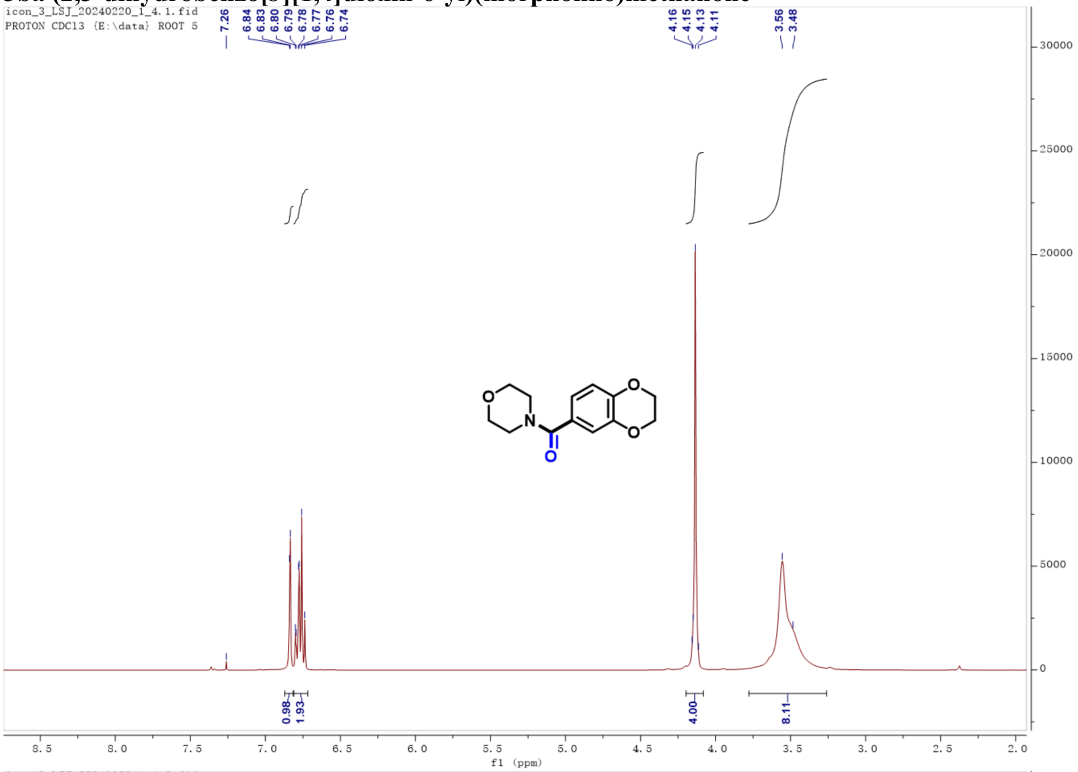

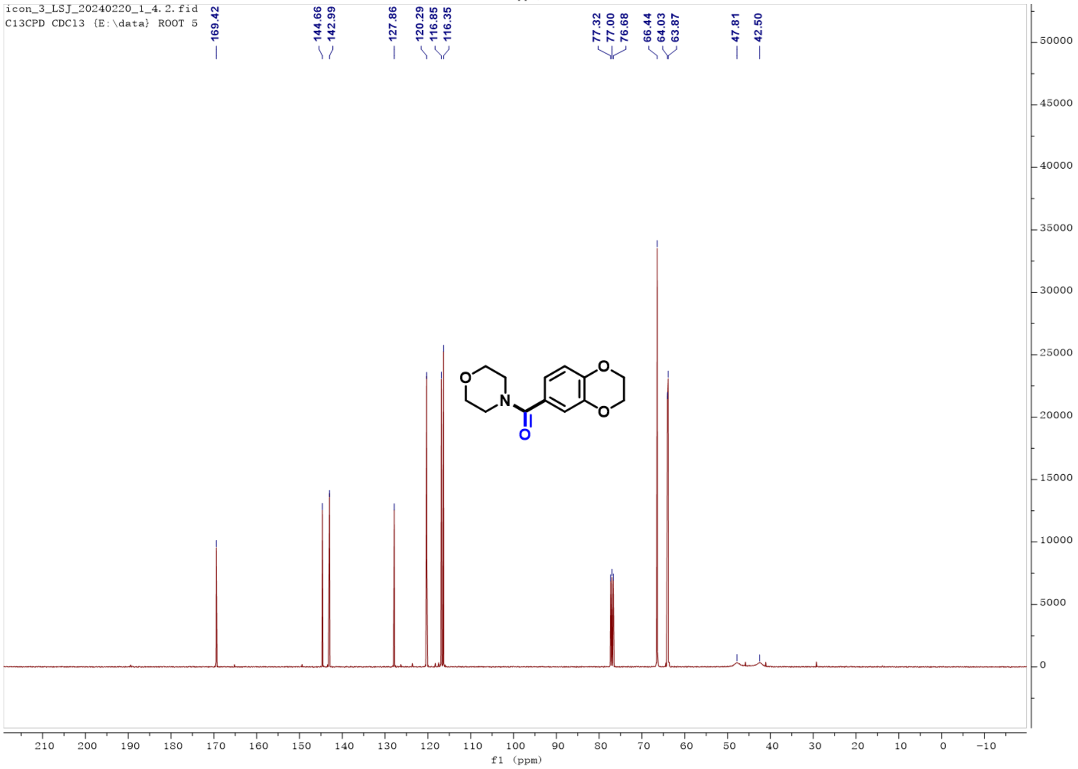

3bb morpholino(naphthalen-1-yl)methanone

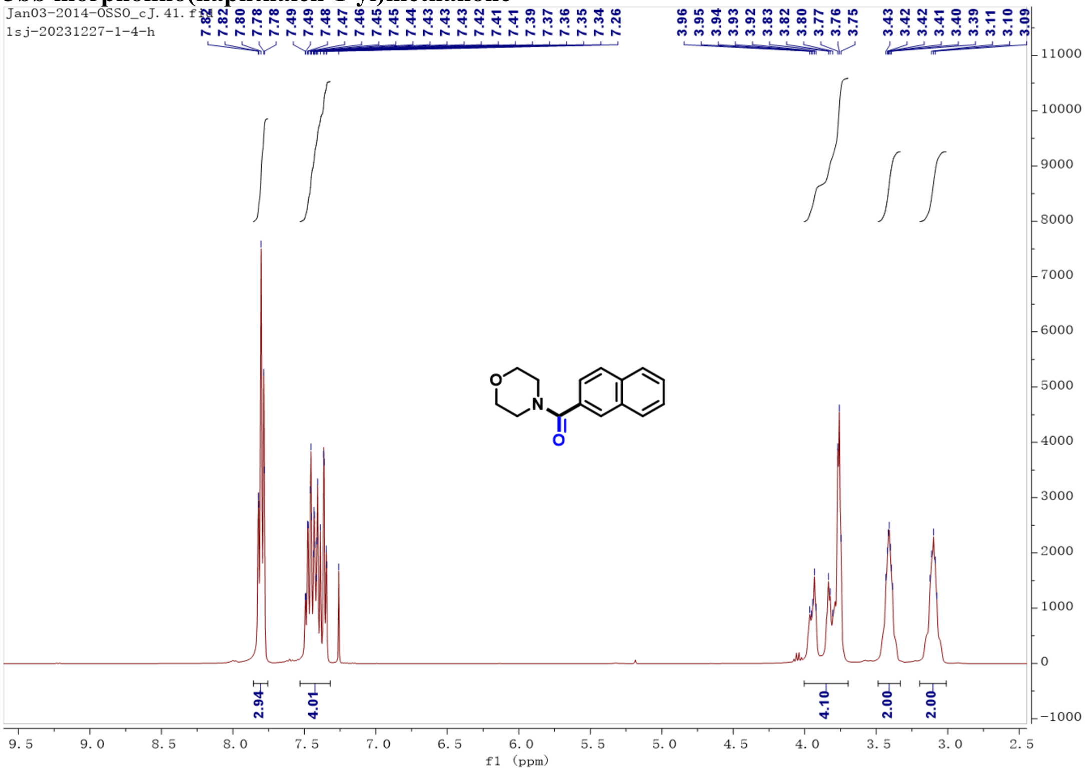

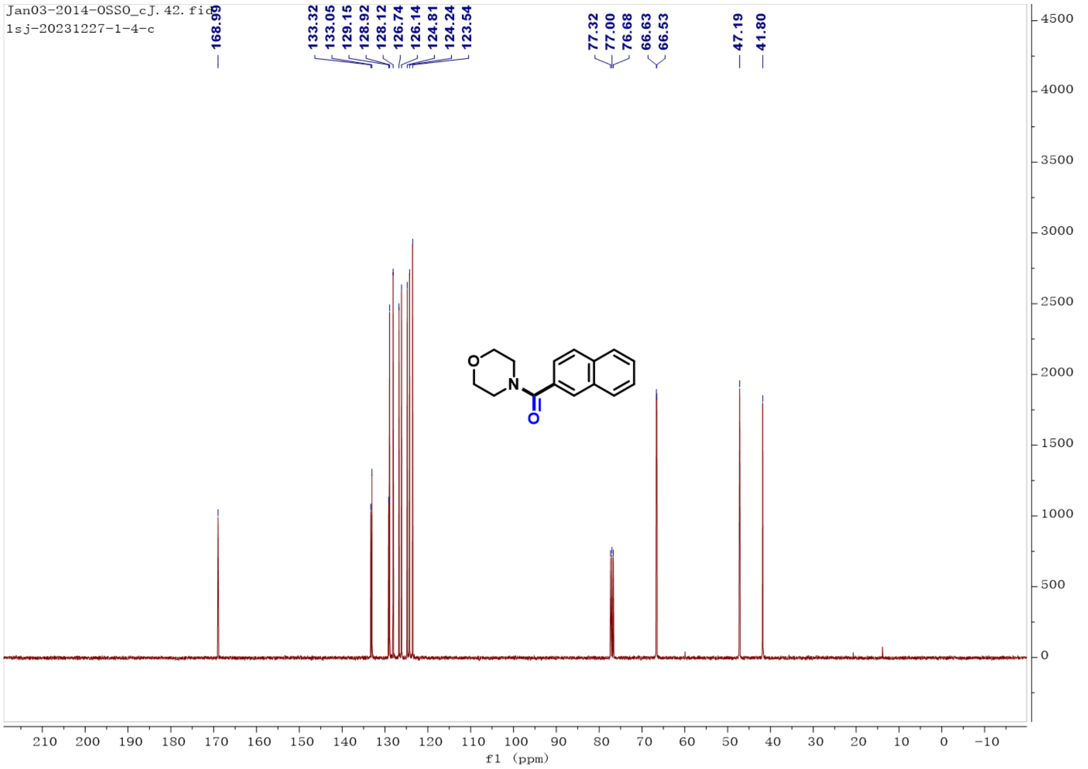

4a 1-morpholino-2-phenylethane-1,2-dione

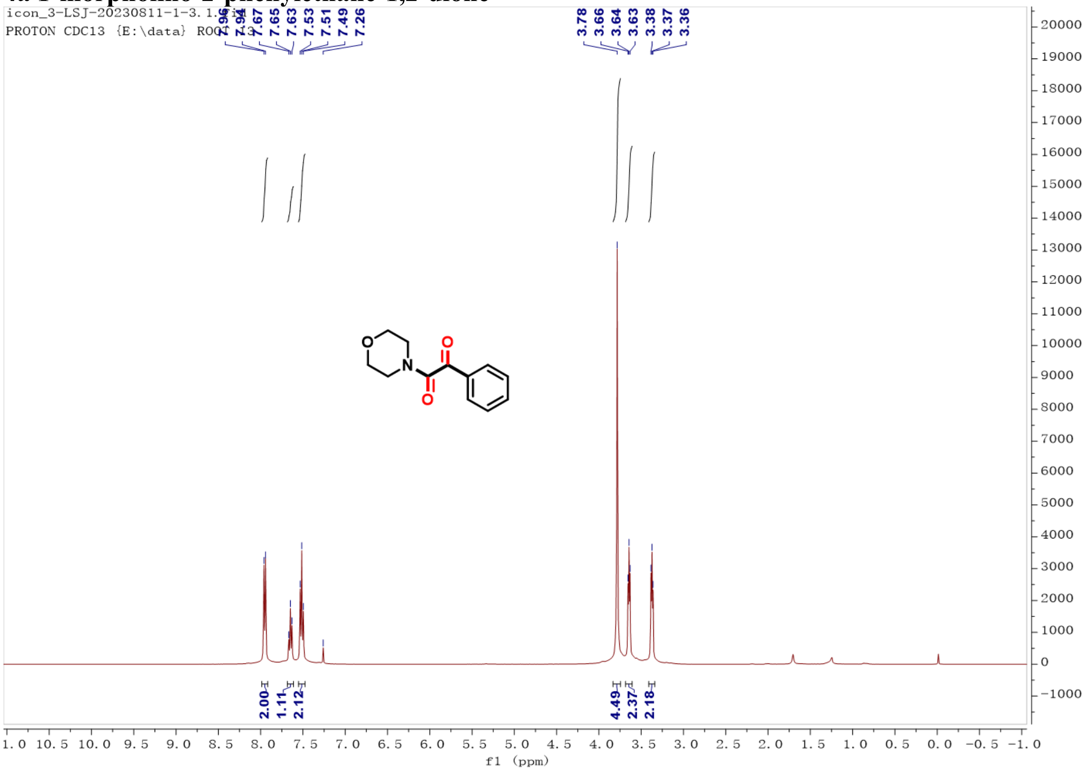

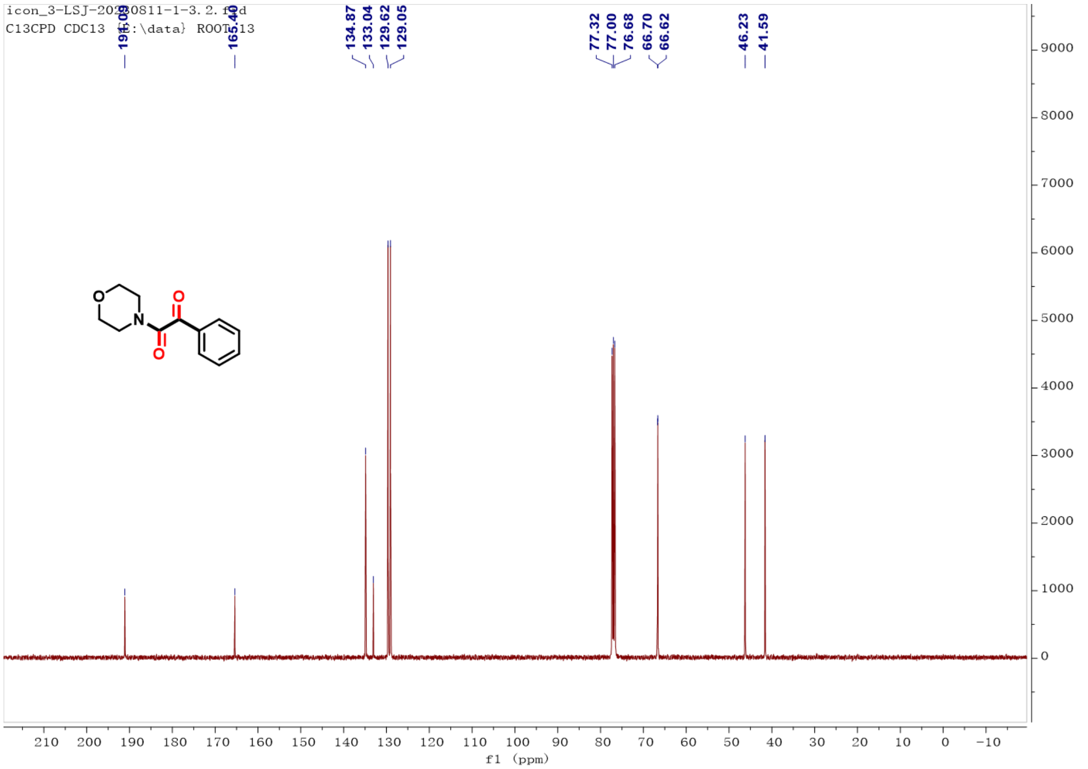

4b 1-(azepan-1-yl)-2-phenylethane-1,2-dione

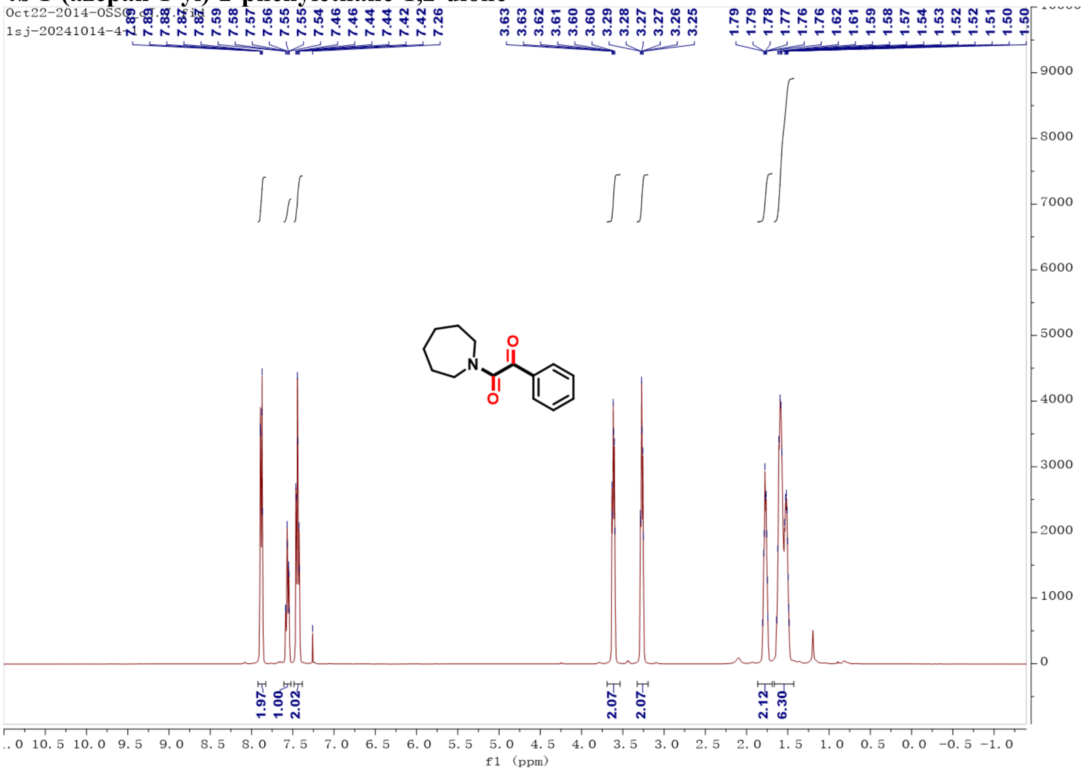

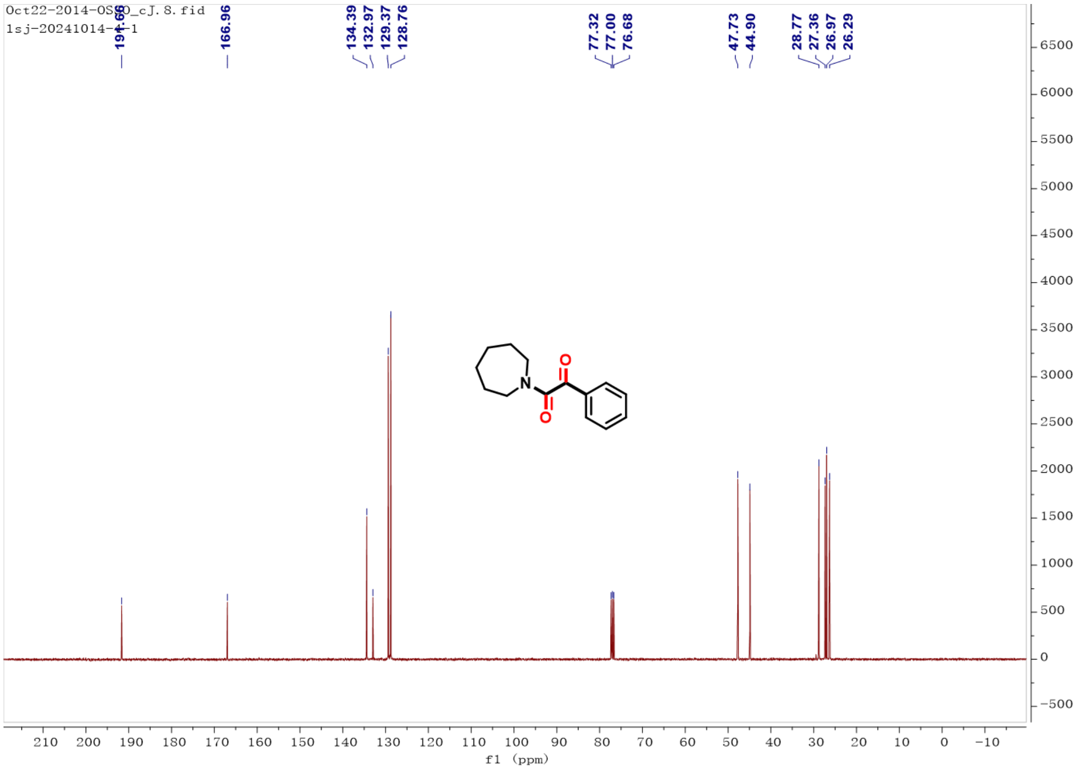

4c 1-phenyl-2-(pyrrolidin-1-yl)ethane-1,2-dione

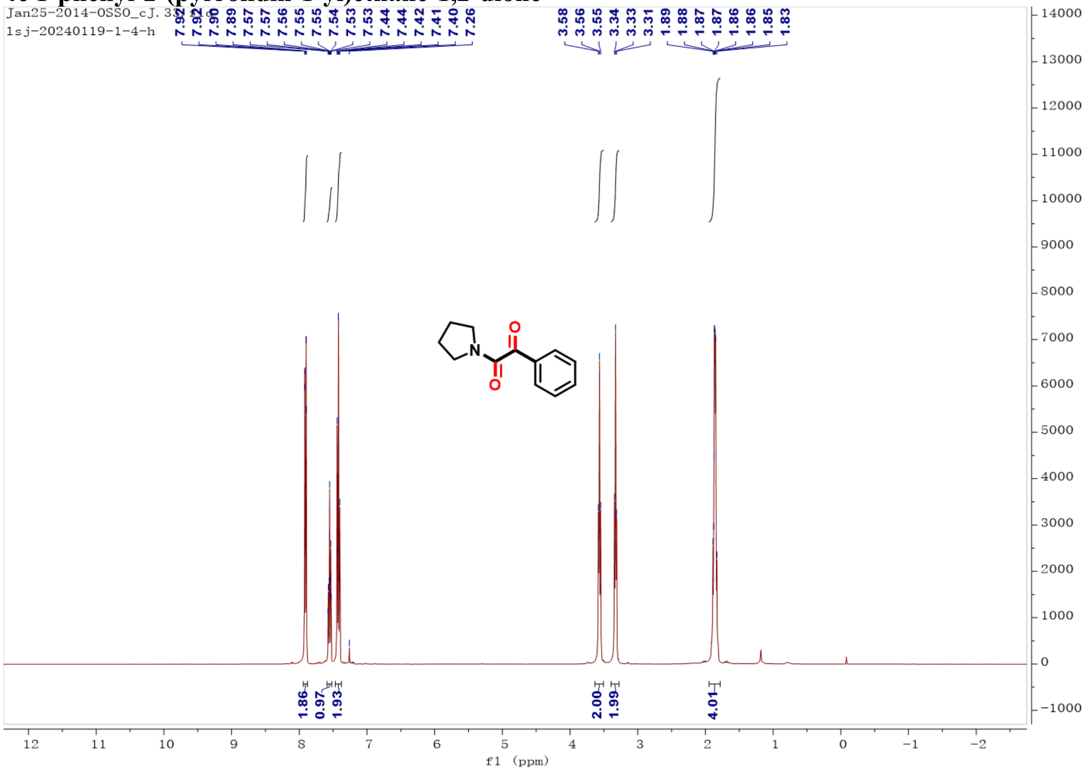

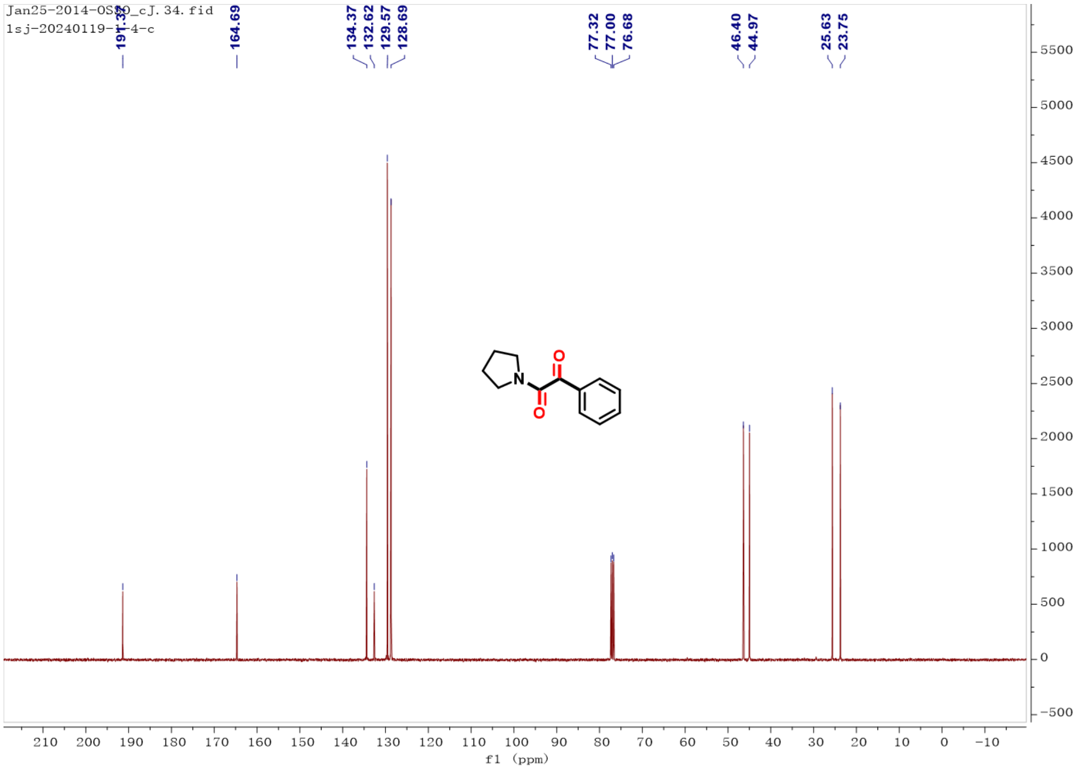

4d 1-phenyl-2-(piperidin-1-yl)ethane-1,2-dione

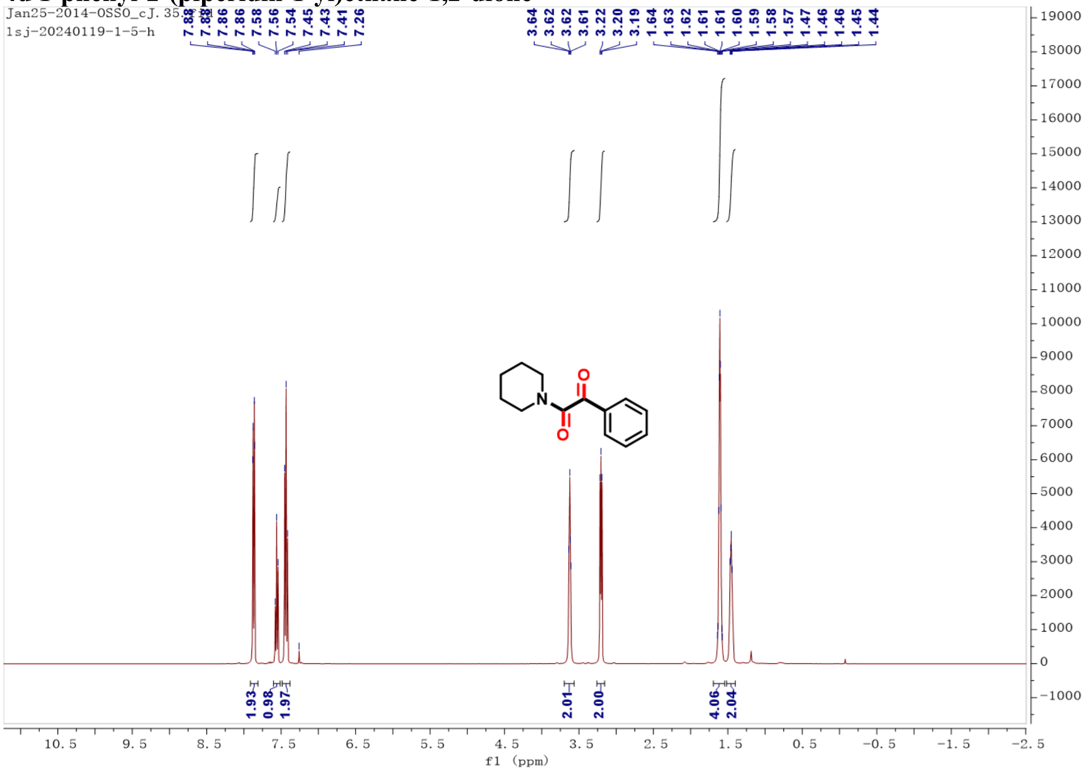

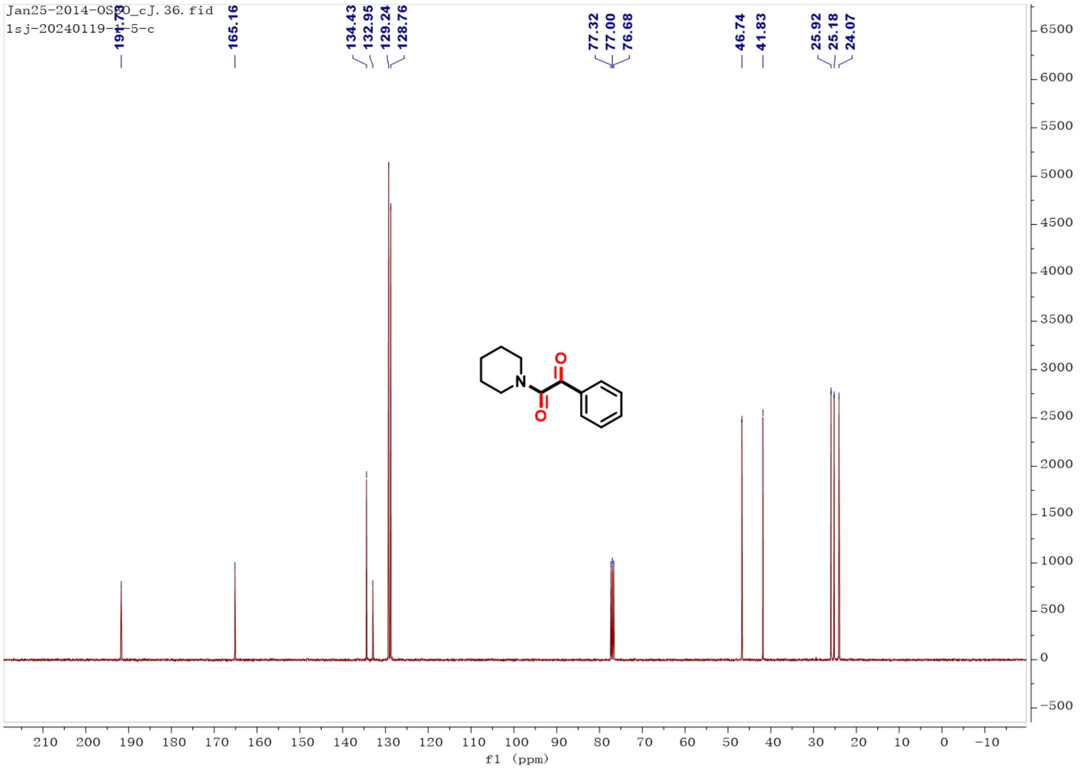

4e 1-(4-methylpiperidin-1-yl)-2-phenylethane-1,2-dione

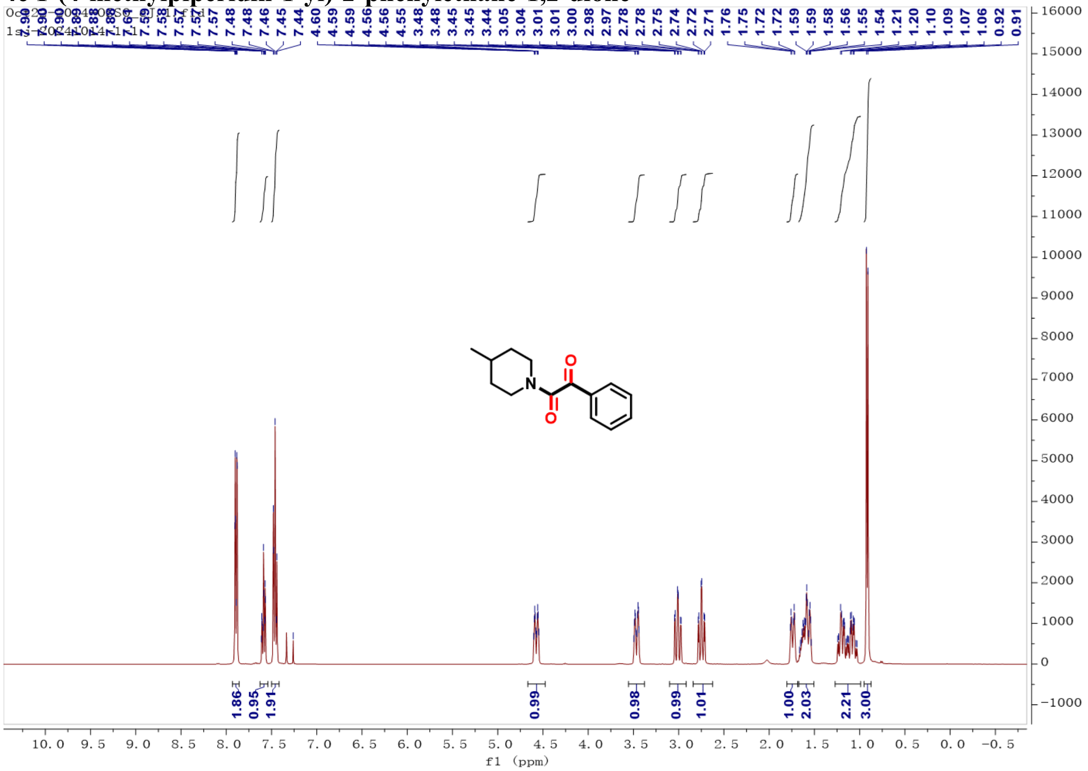

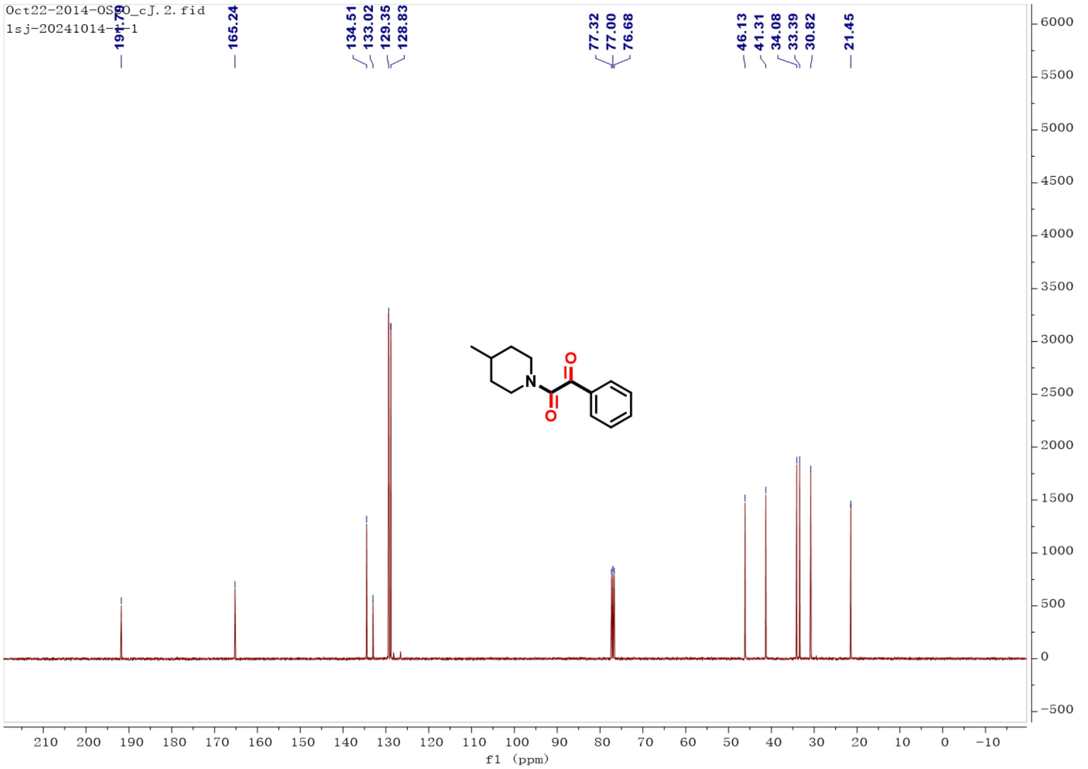

4f methyl 1-(2-oxo-2-phenylacetyl)piperidine-4-carboxylate

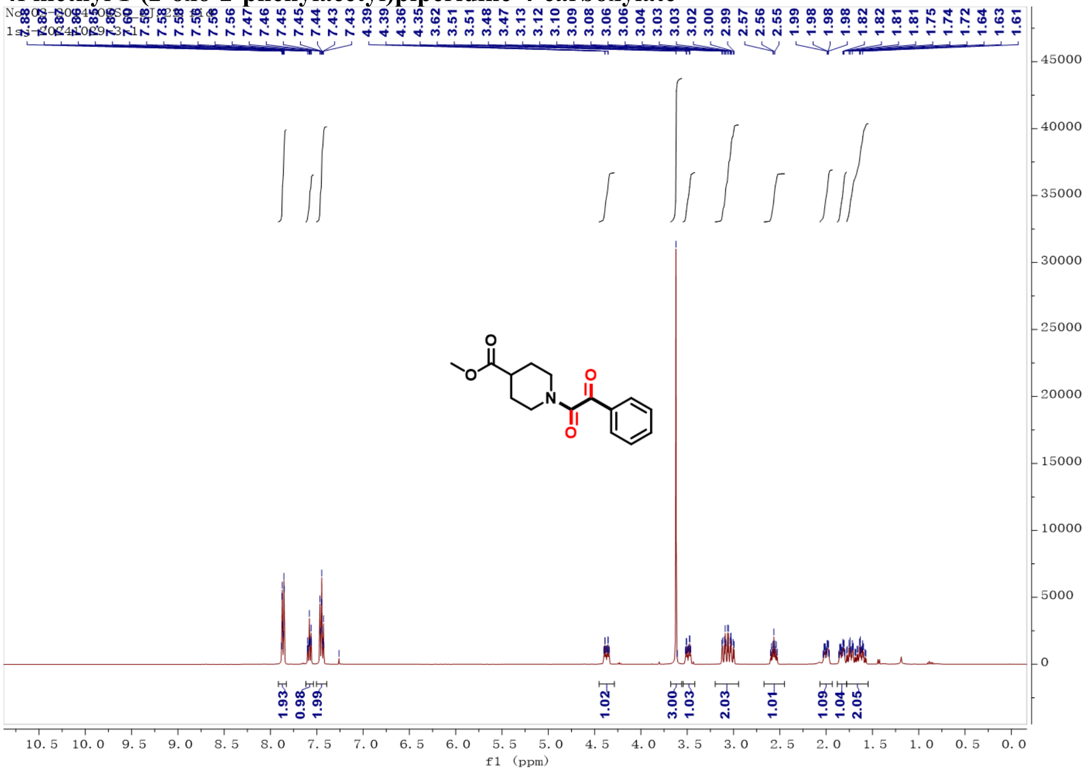

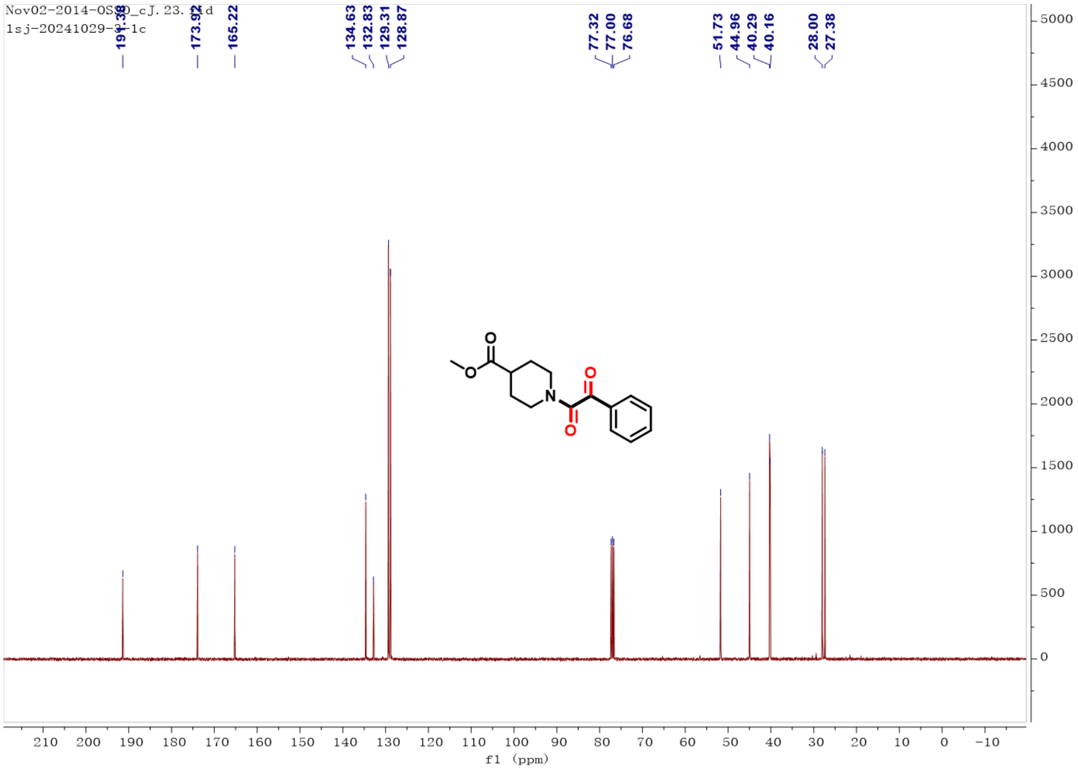

4g 1-(3,5-dimethylpiperidin-1-yl)-2-phenylethane-1,2-dione

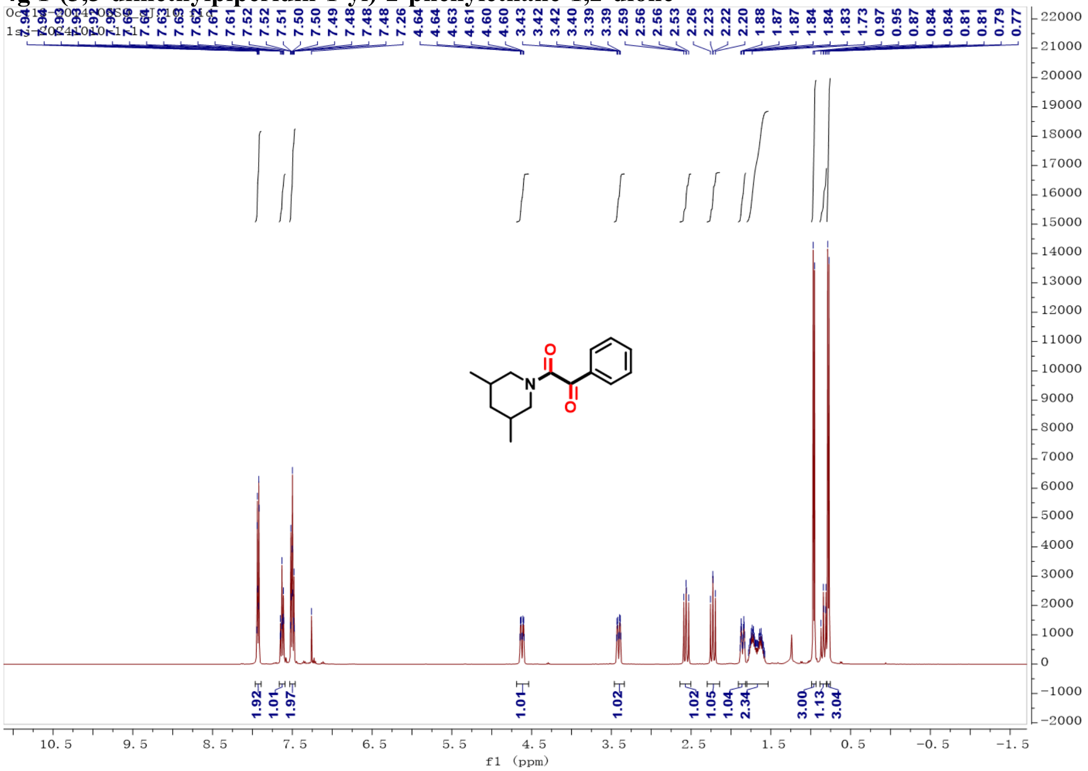

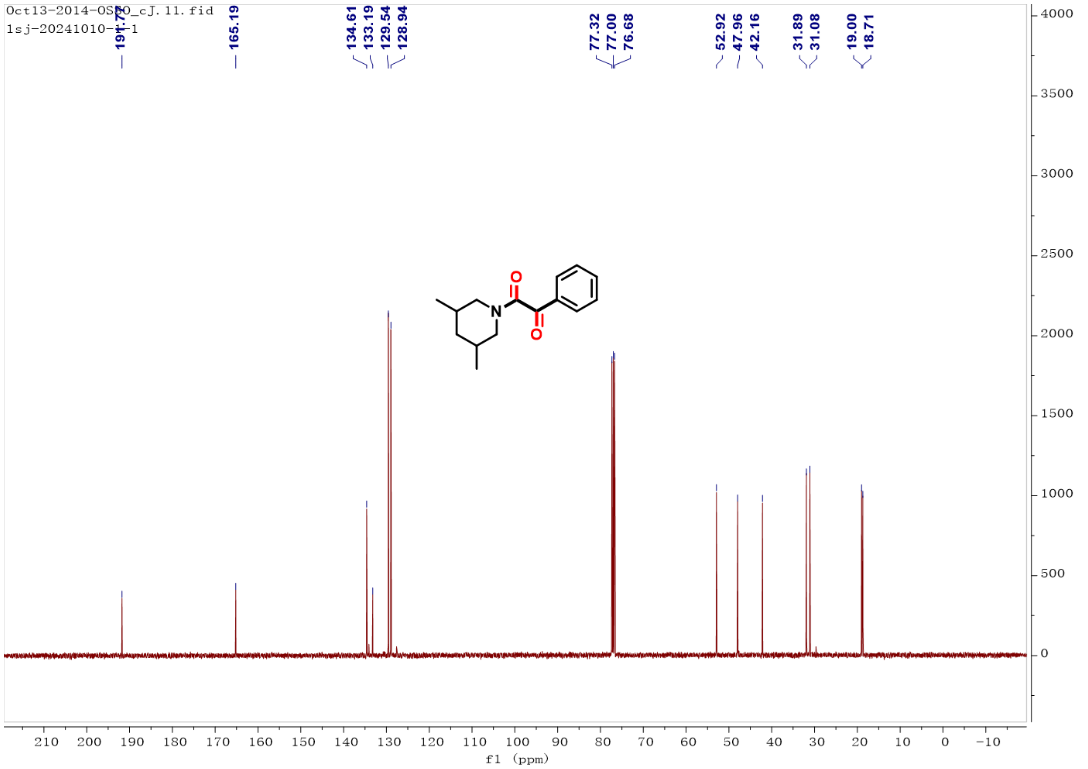

4h 1-(3,4-dihydroisoquinolin-2(1*H*)-yl)-2-phenylethane-1,2-dione

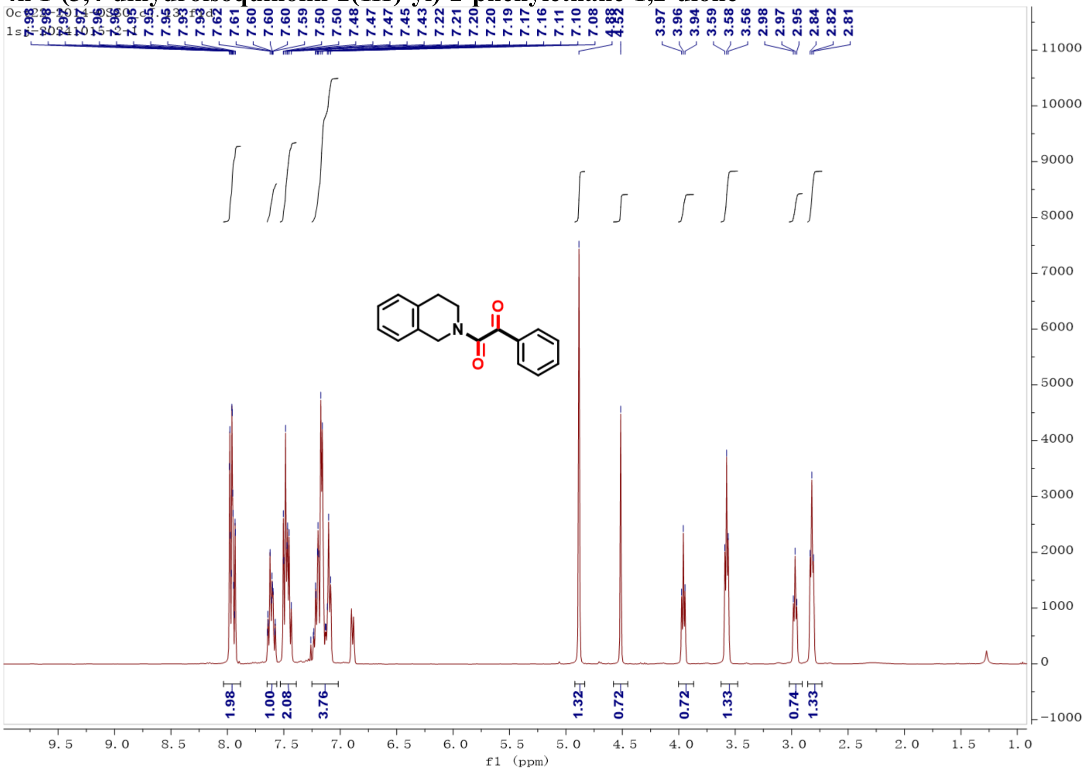

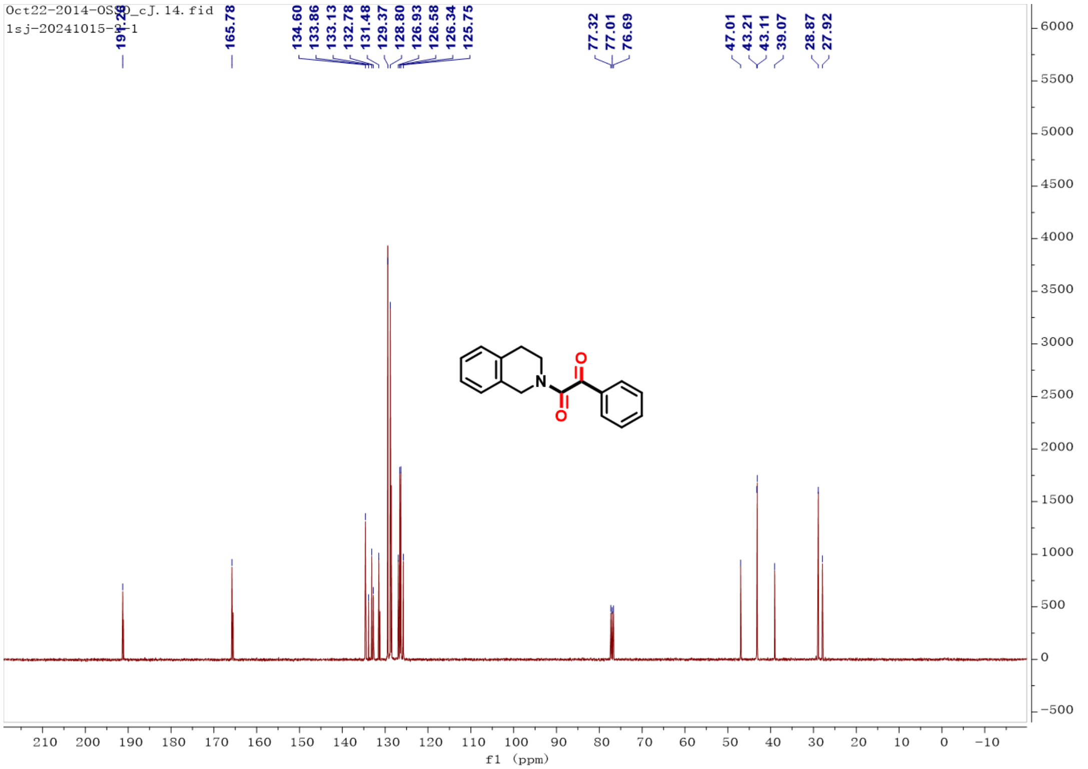

4i 1-(4-ethylpiperazin-1-yl)-2-phenylethane-1,2-dione

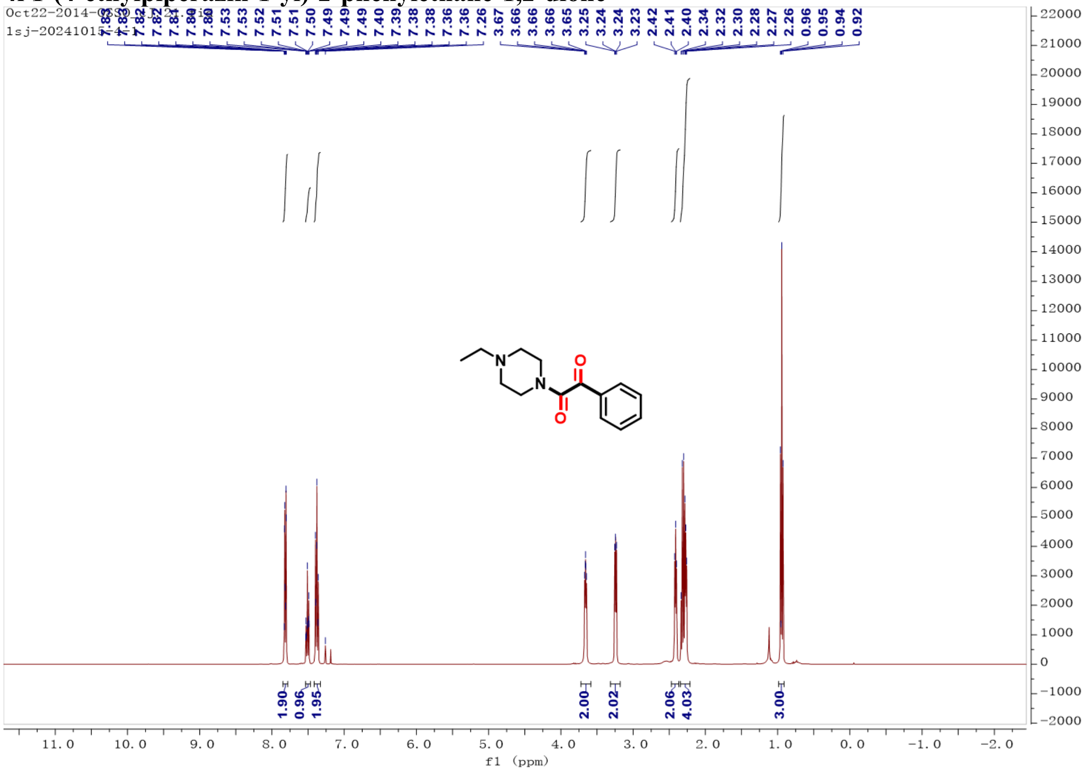

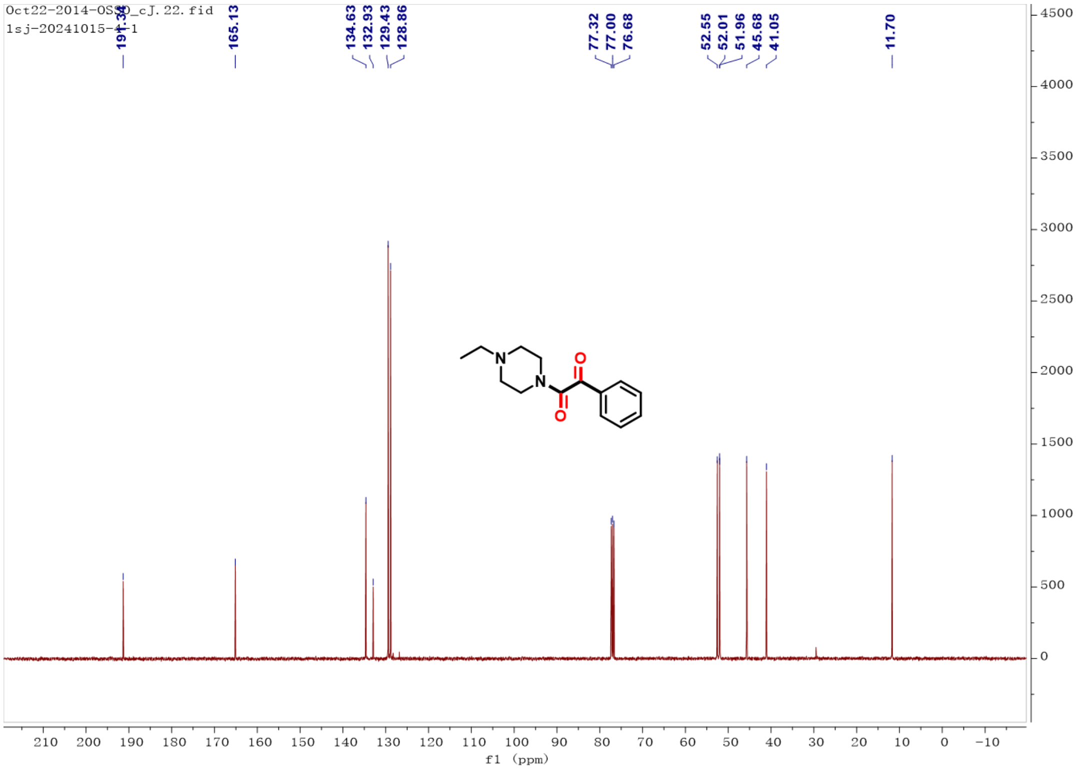

4j 1-phenyl-2-(4-phenylpiperazin-1-yl)ethane-1,2-dione

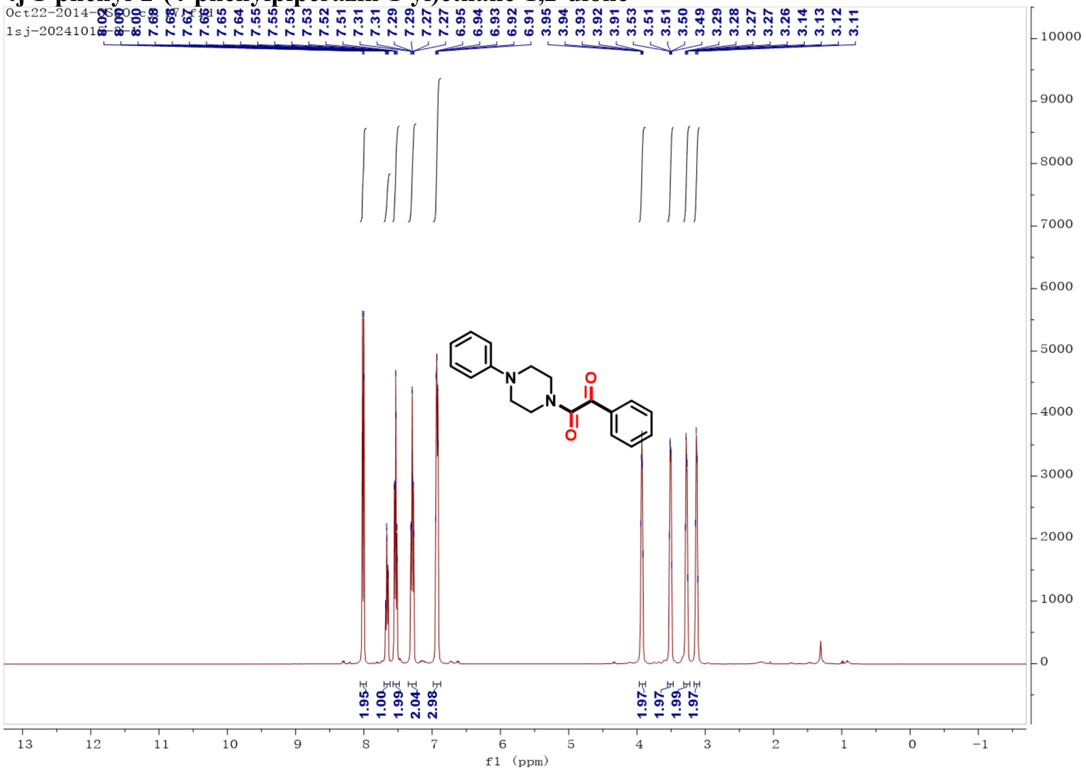

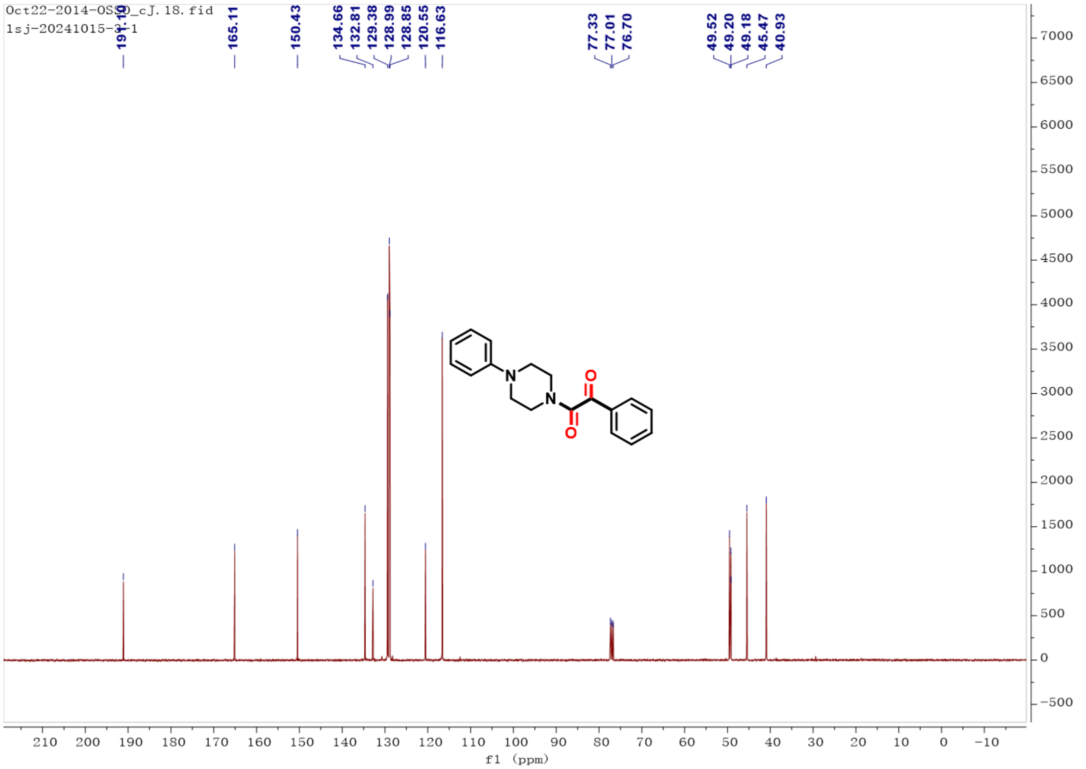

4k 1-(4-(2-hydroxyethyl)piperazin-1-yl)-2-phenylethane-1,2-dione

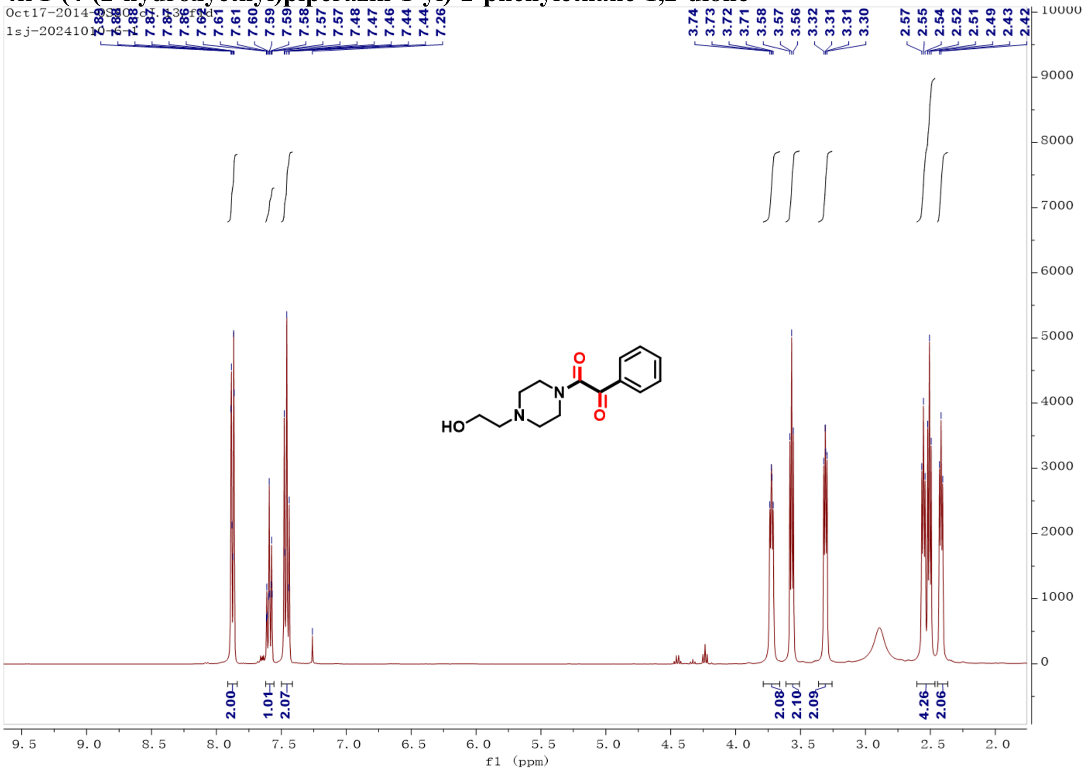

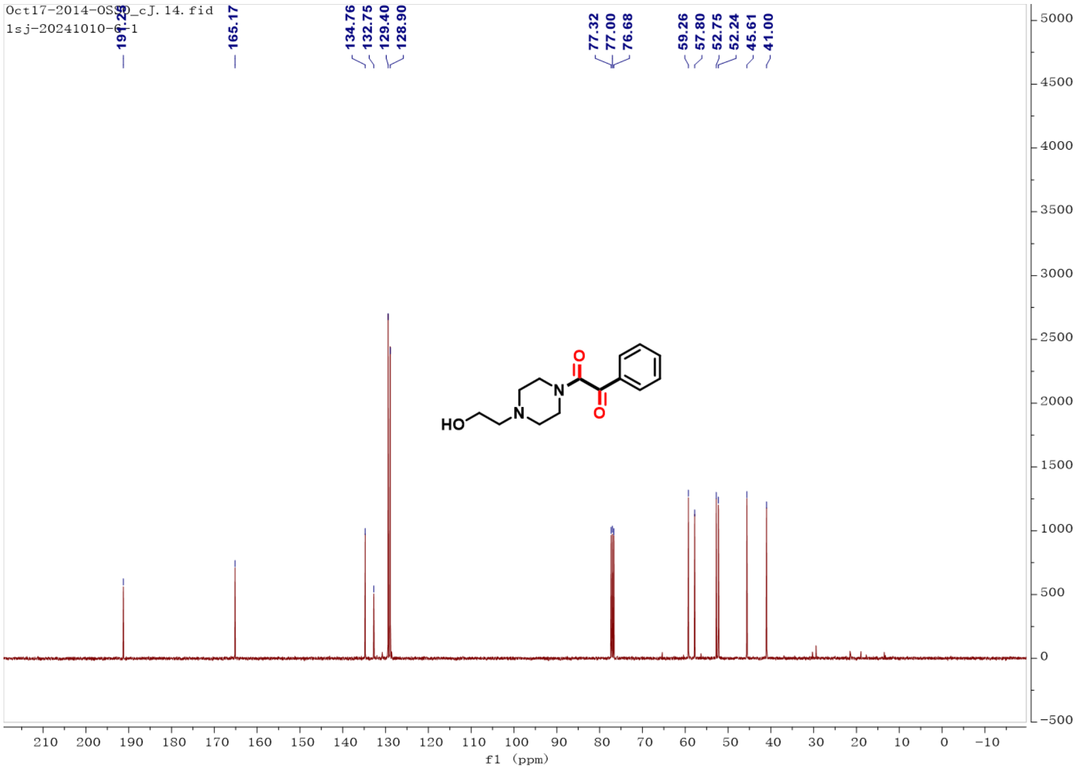

4l 1-phenyl-2-(4-(pyrimidin-2-yl)piperazin-1-yl)ethane-1,2-dione

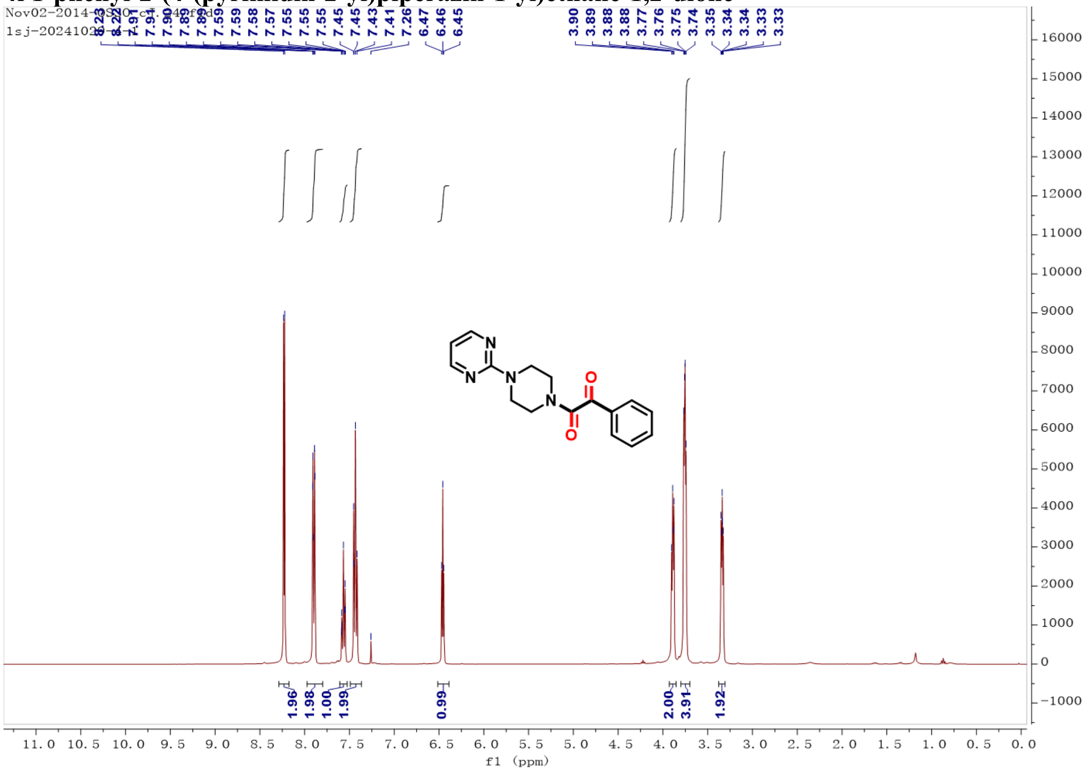

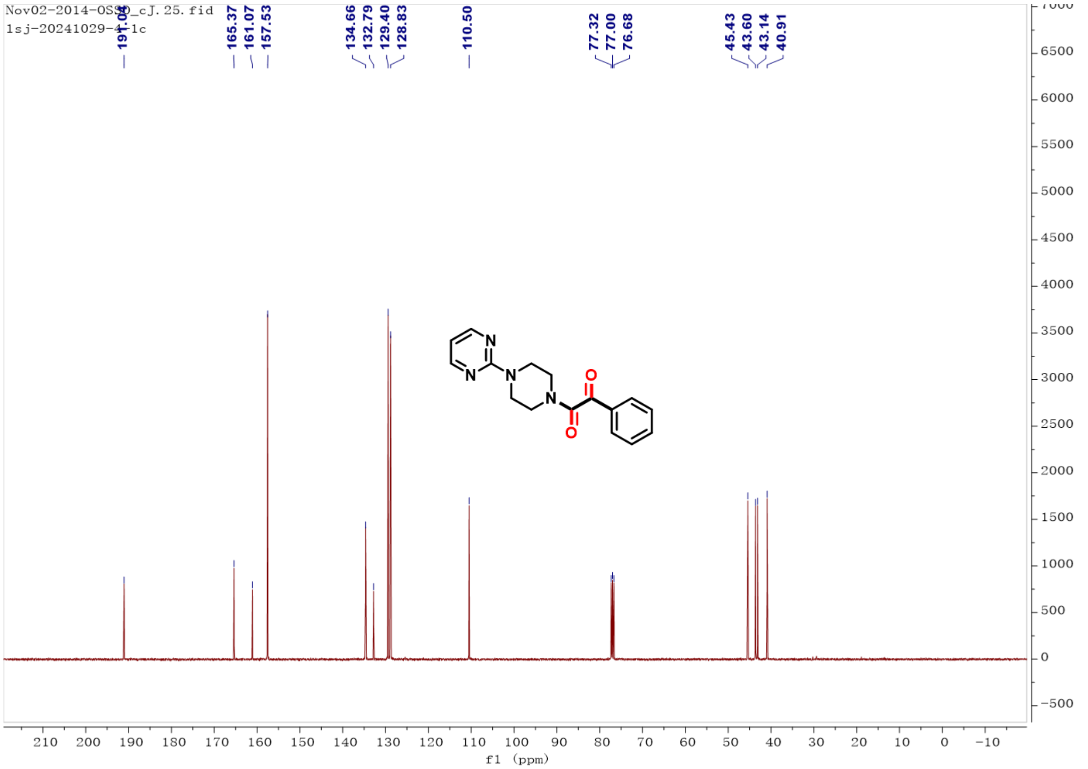

4m benzyl 4-(2-oxo-2-phenylacetyl)piperazine-1-carboxylate

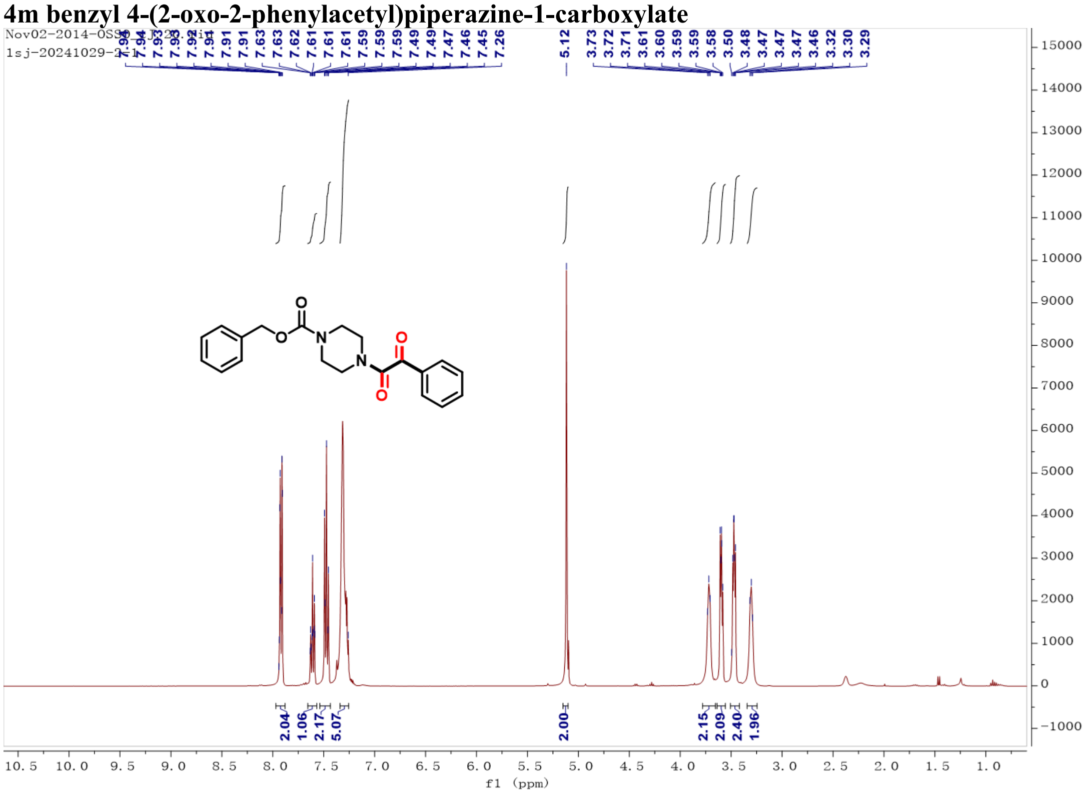

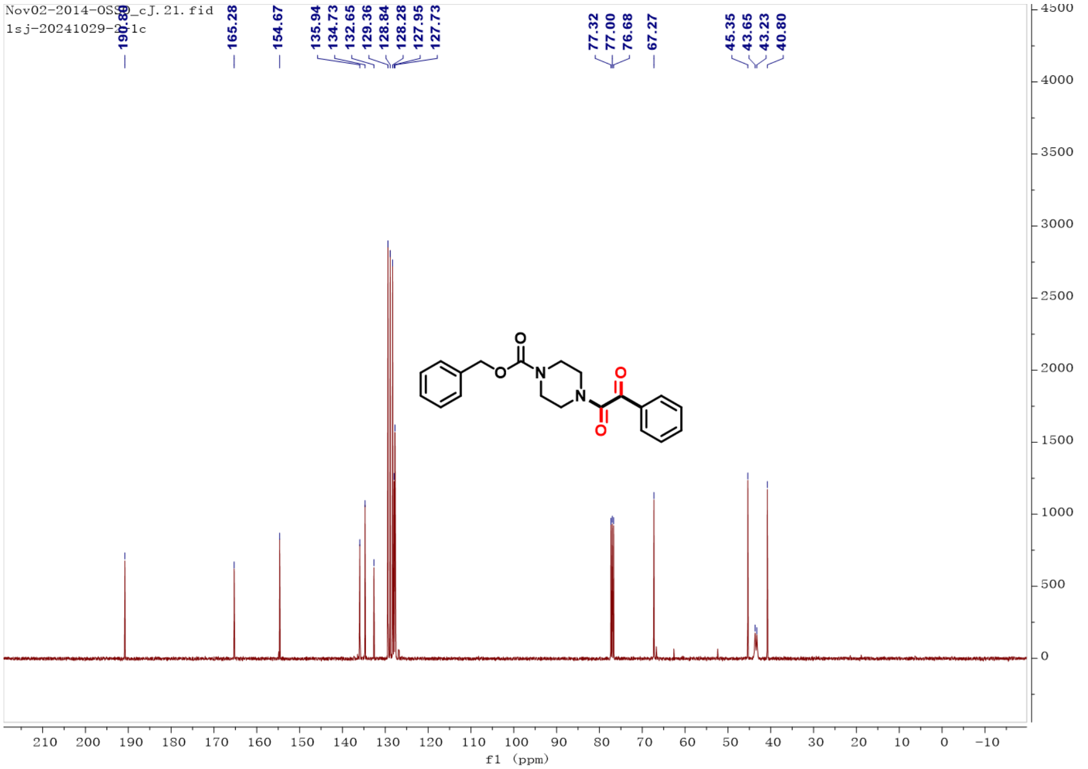

4n *N,N*-diethyl-2-oxo-2-phenylacetamide

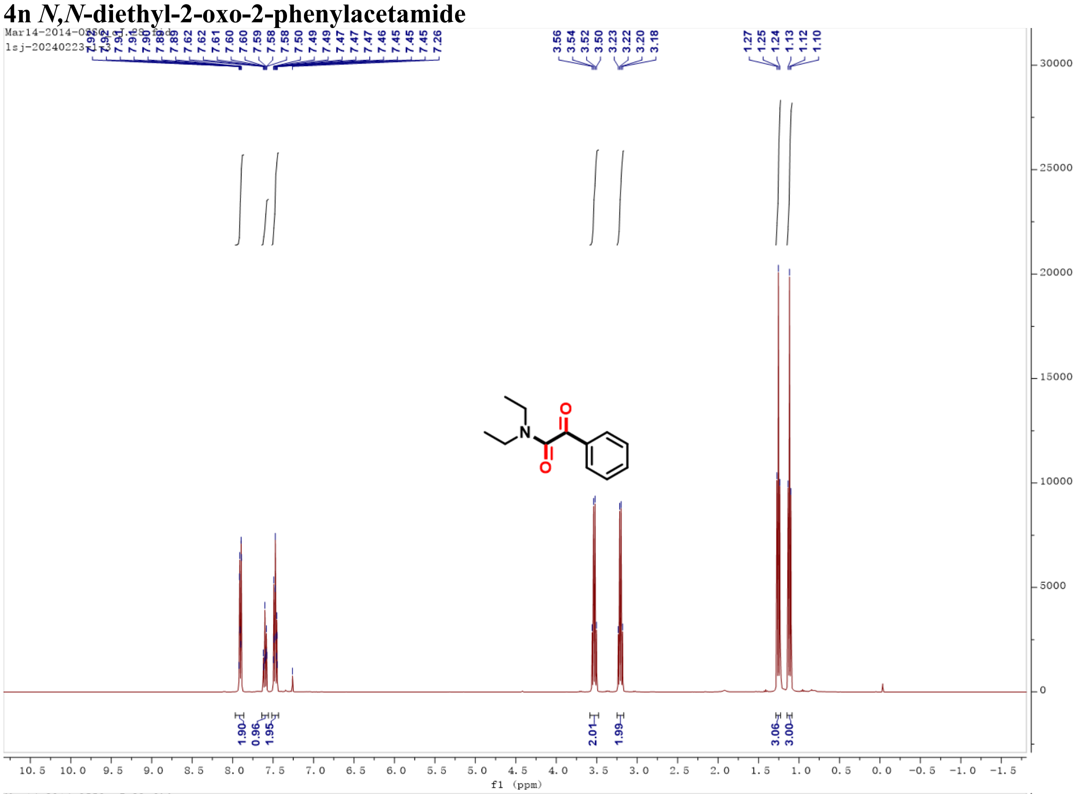

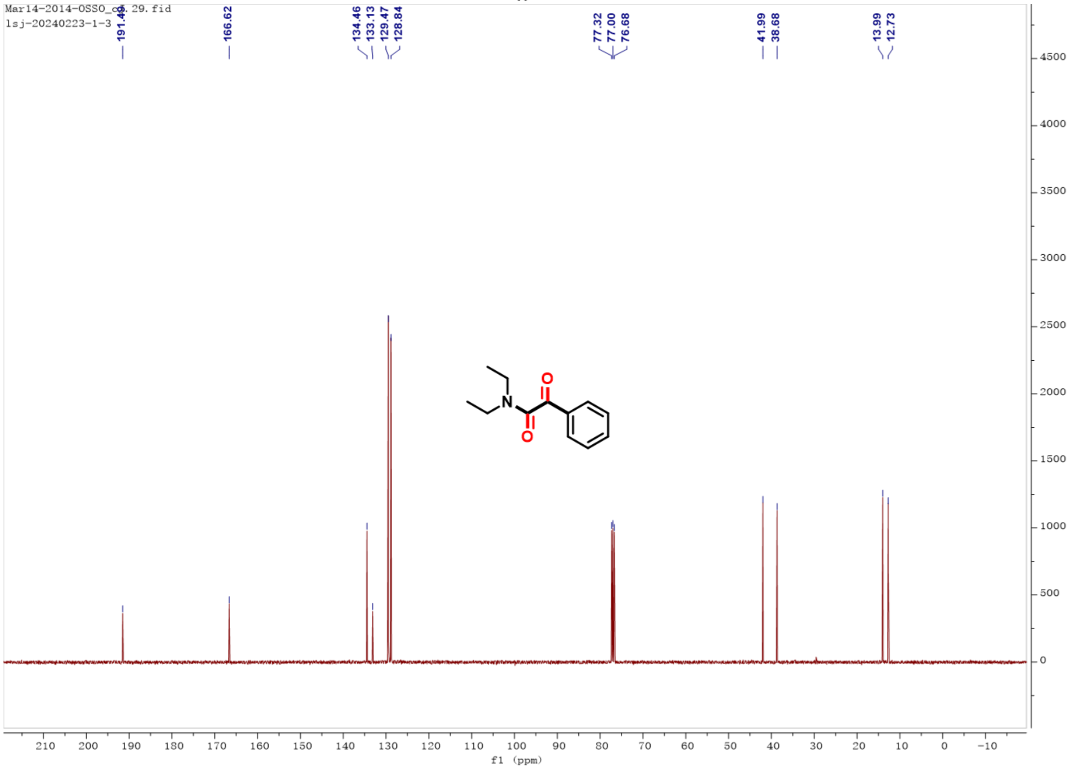

4o *N*,*N*-dibutyl-2-oxo-2-phenylacetamide

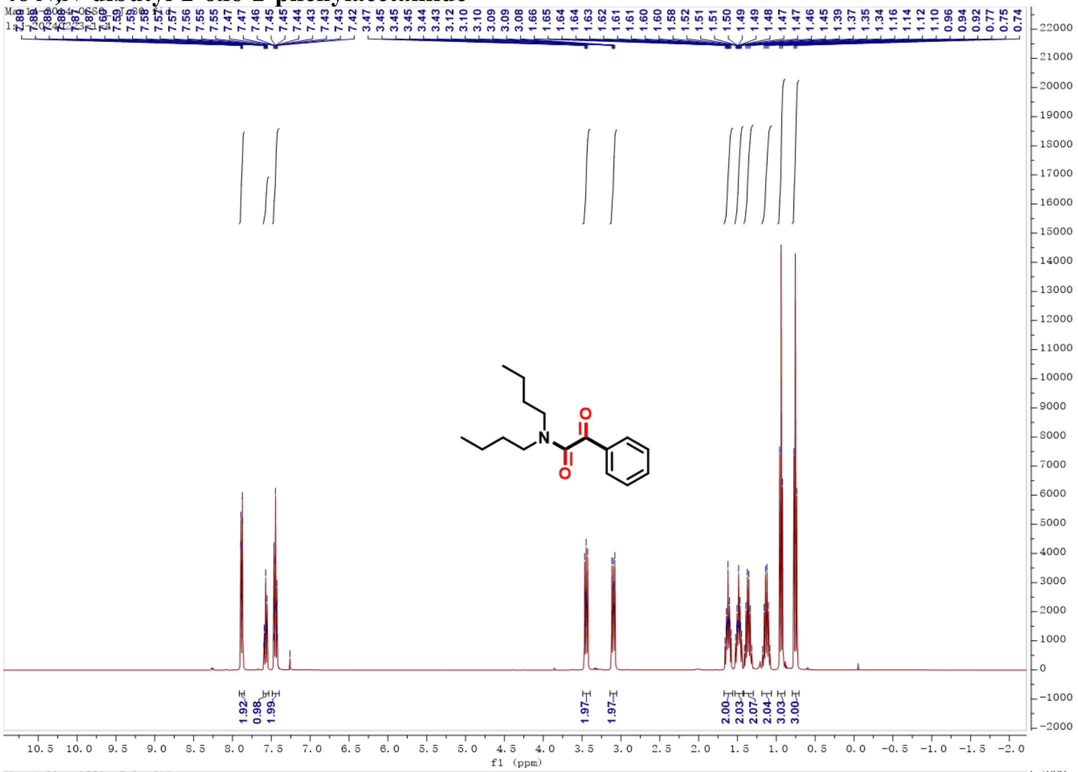

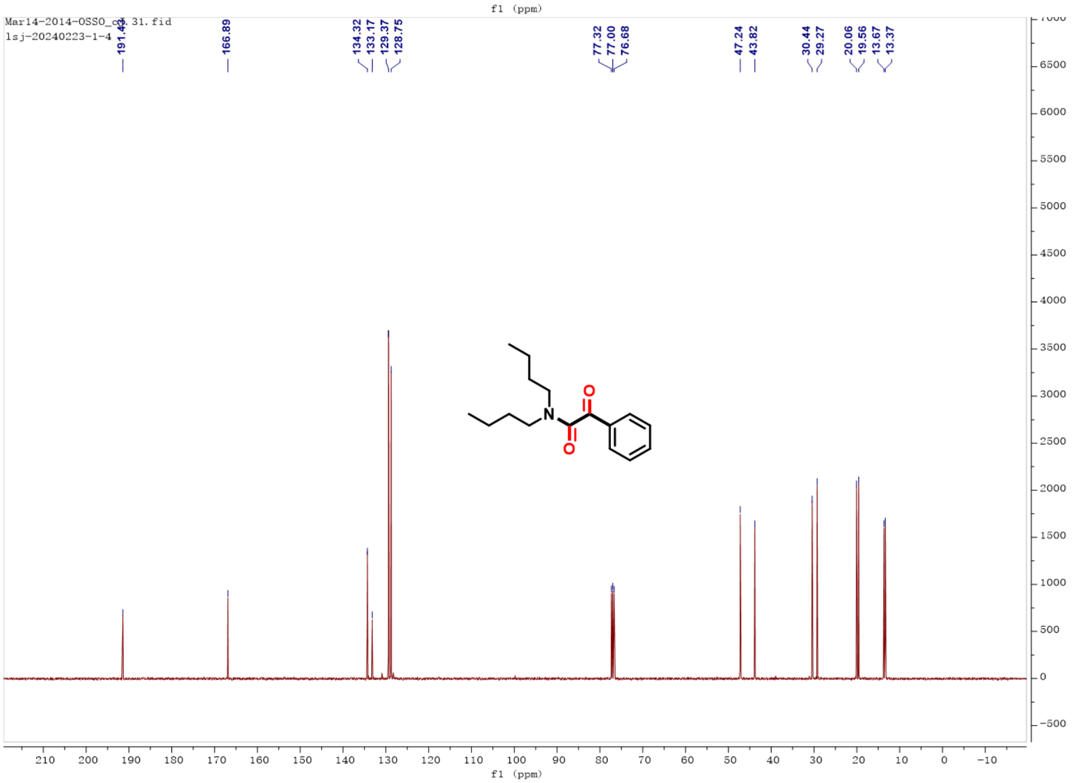

4p *N*-butyl-*N*-methyl-2-oxo-2-phenylacetamide

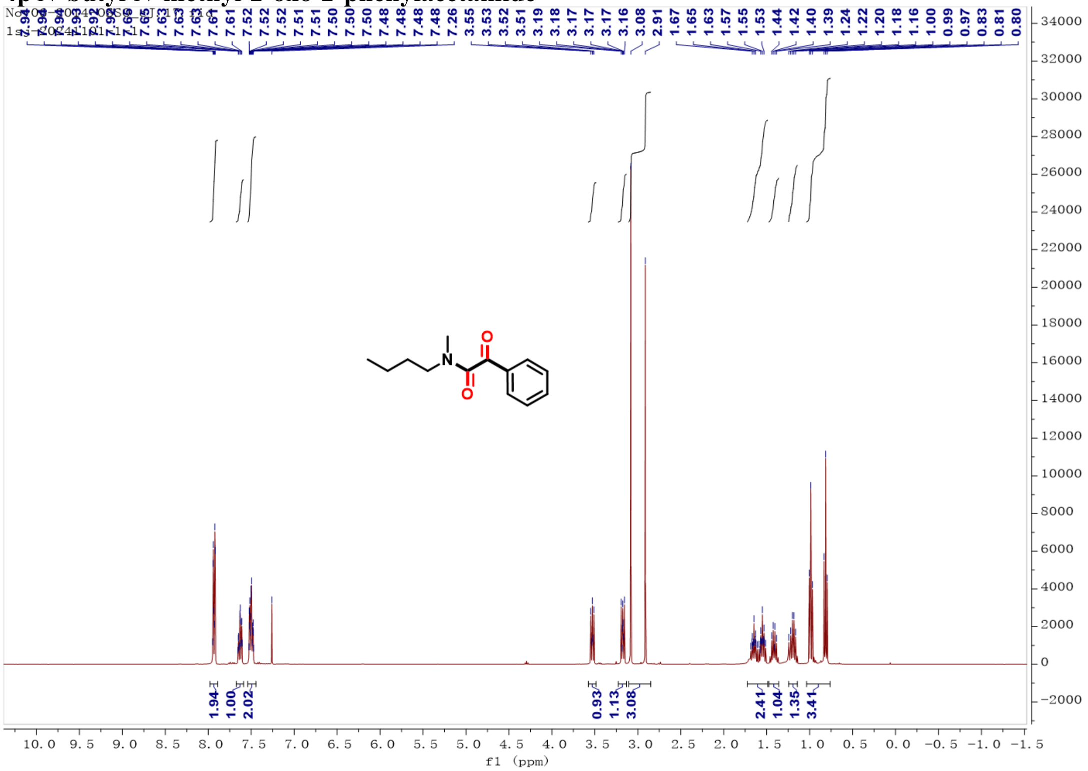

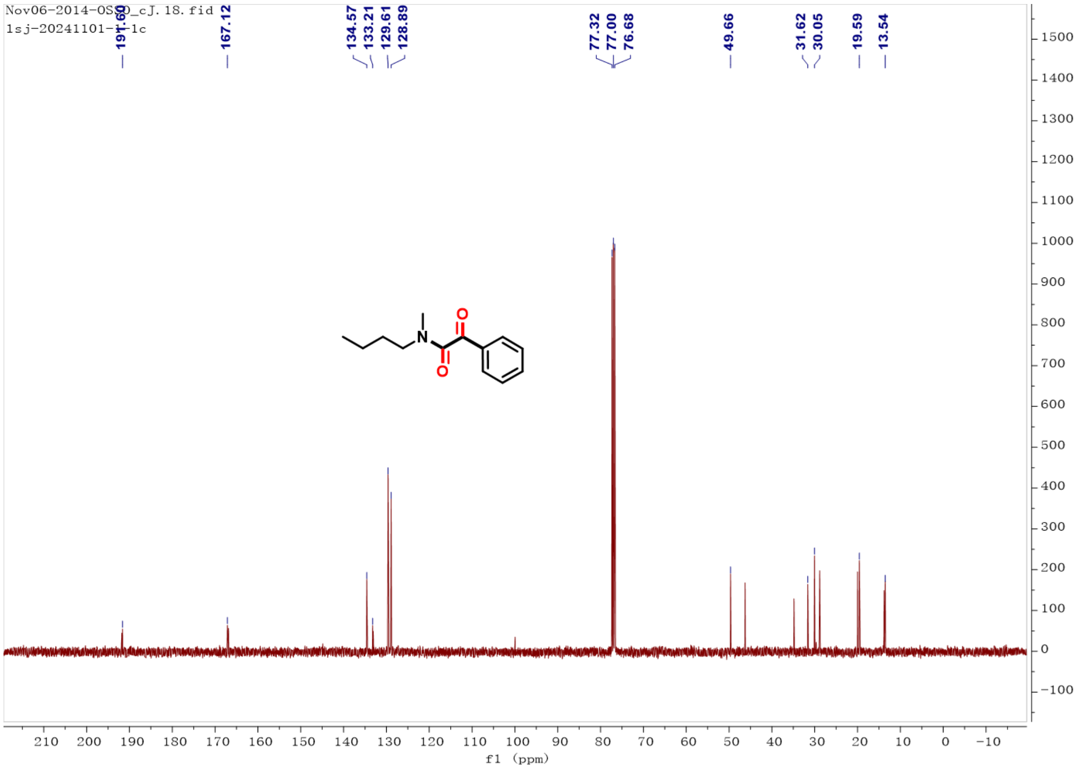

4q *N*-cyclohexyl-*N*-methyl-2-oxo-2-phenylacetamide

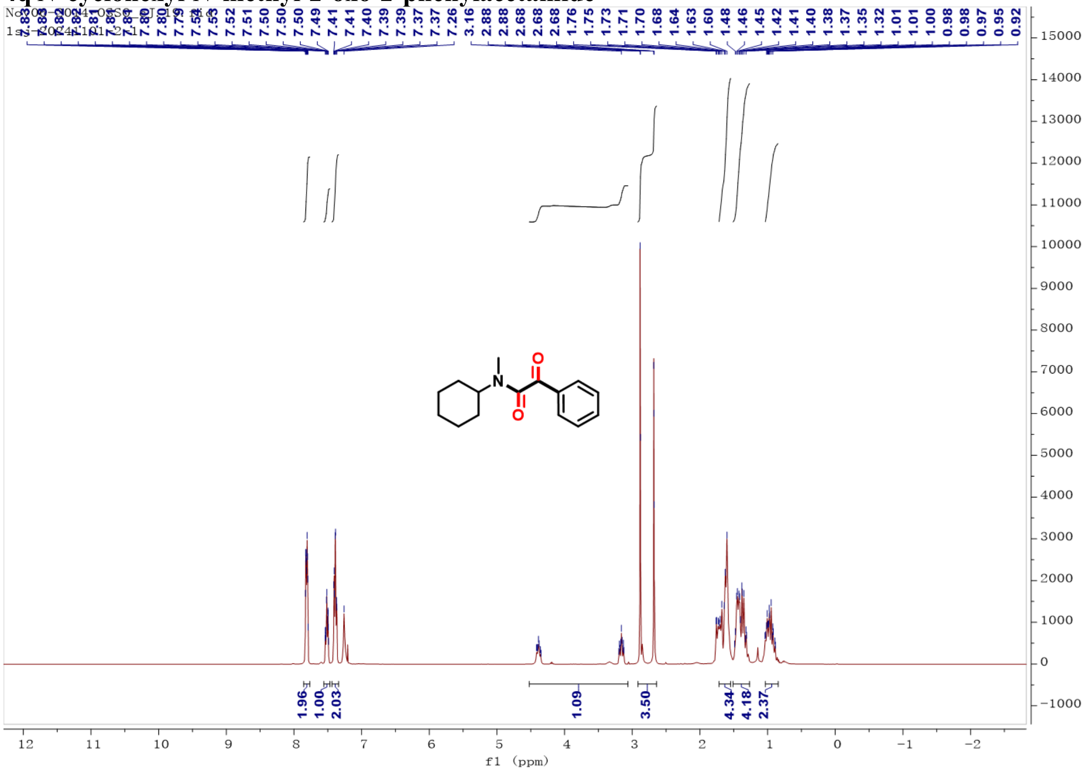

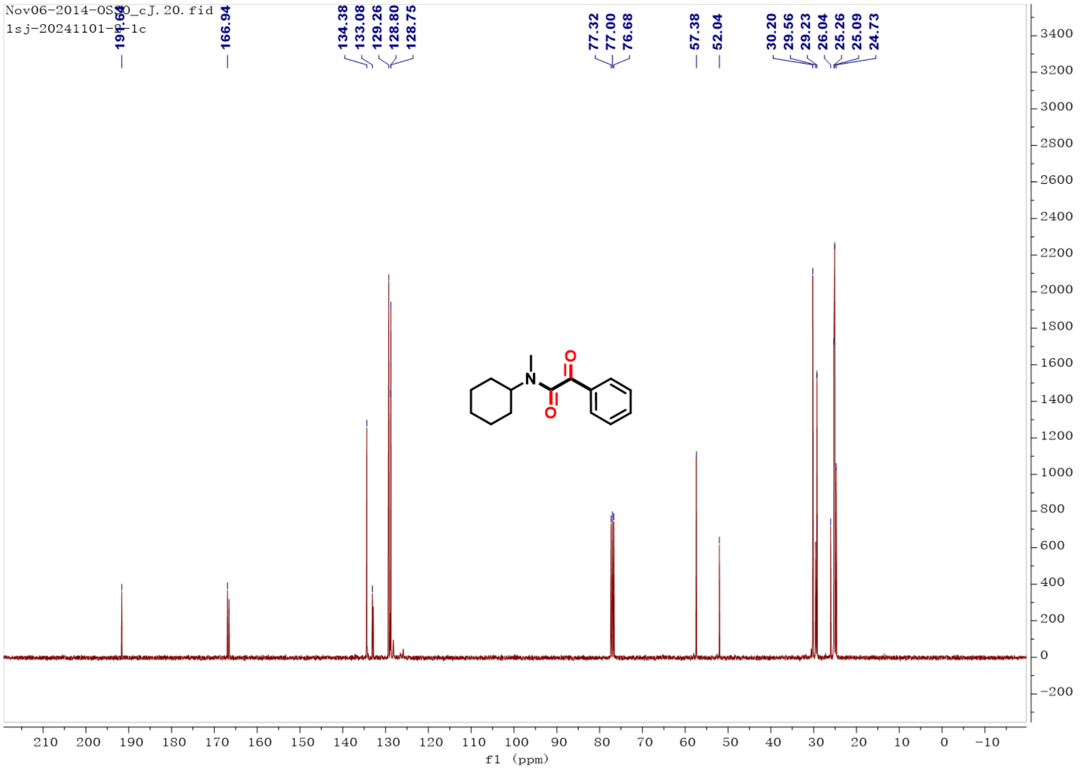

4r *N*-allyl-*N*-methyl-2-oxo-2-phenylacetamide

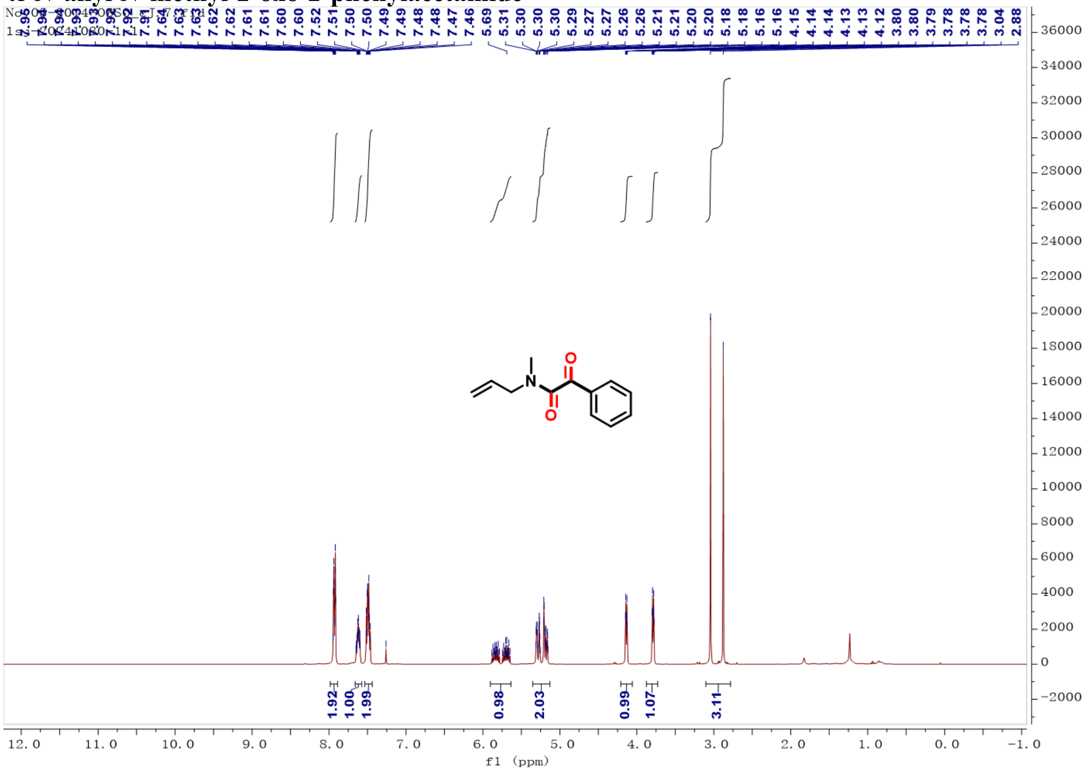

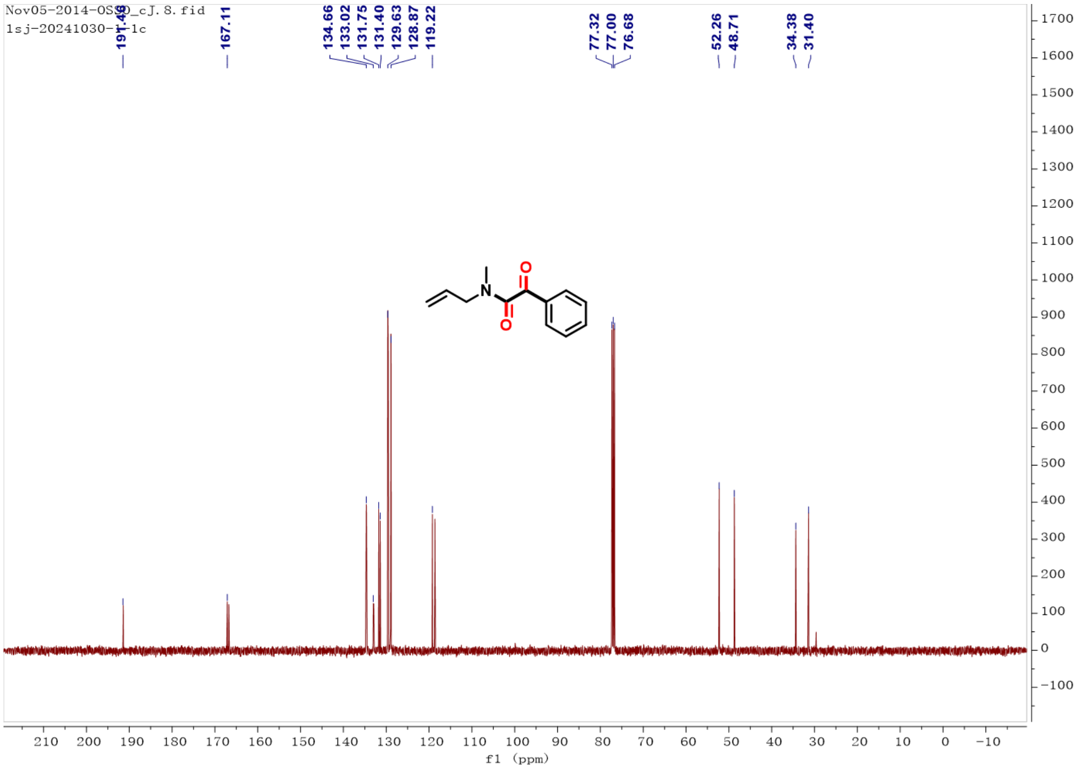

4s *N*-(2-methoxyethyl)-*N*-methyl-2-oxo-2-phenylacetamide

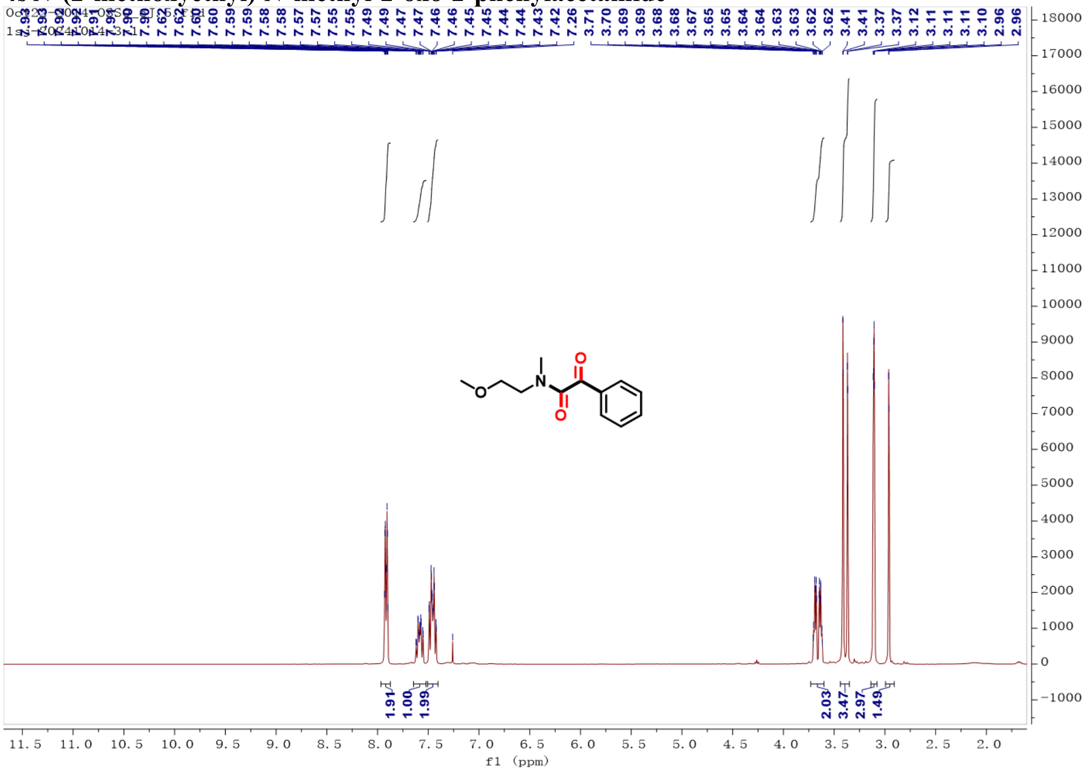

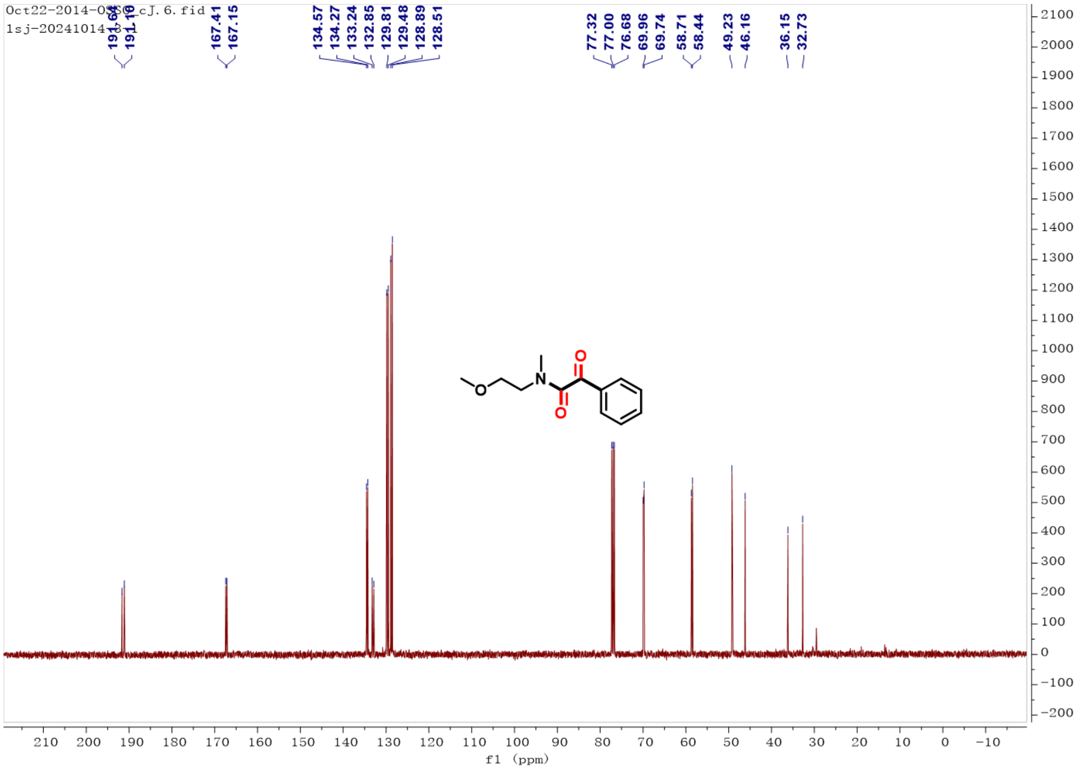

4t 2-oxo-2-phenyl-*N*-propylacetamide

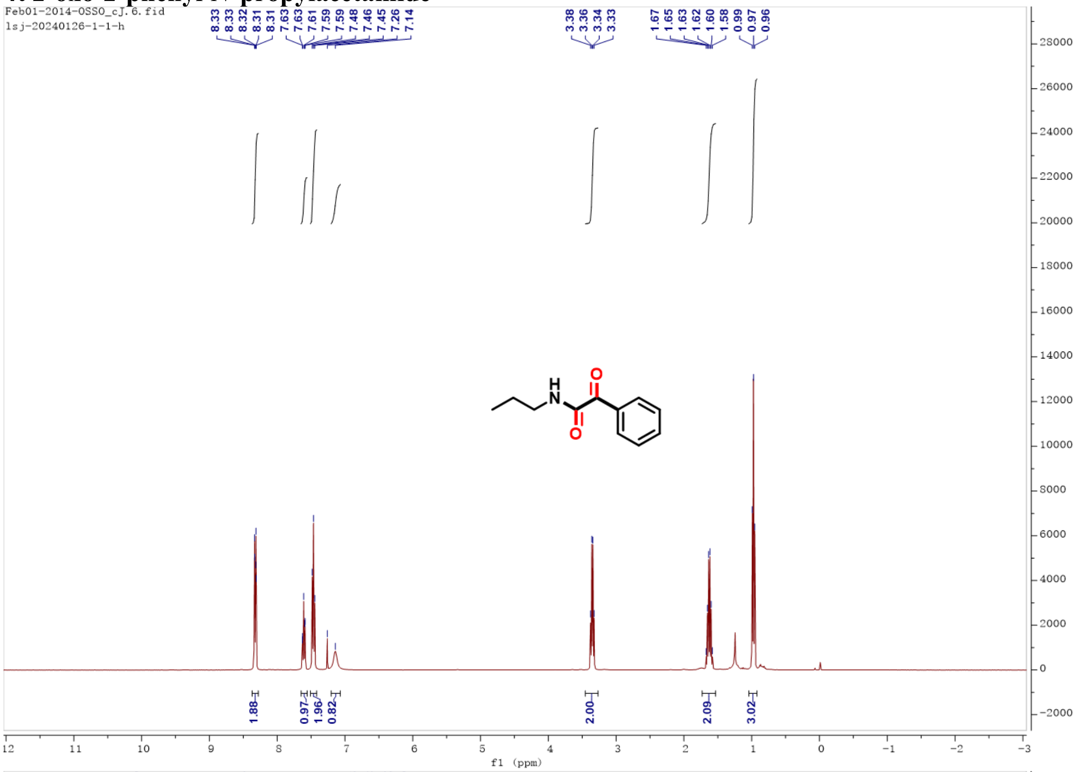

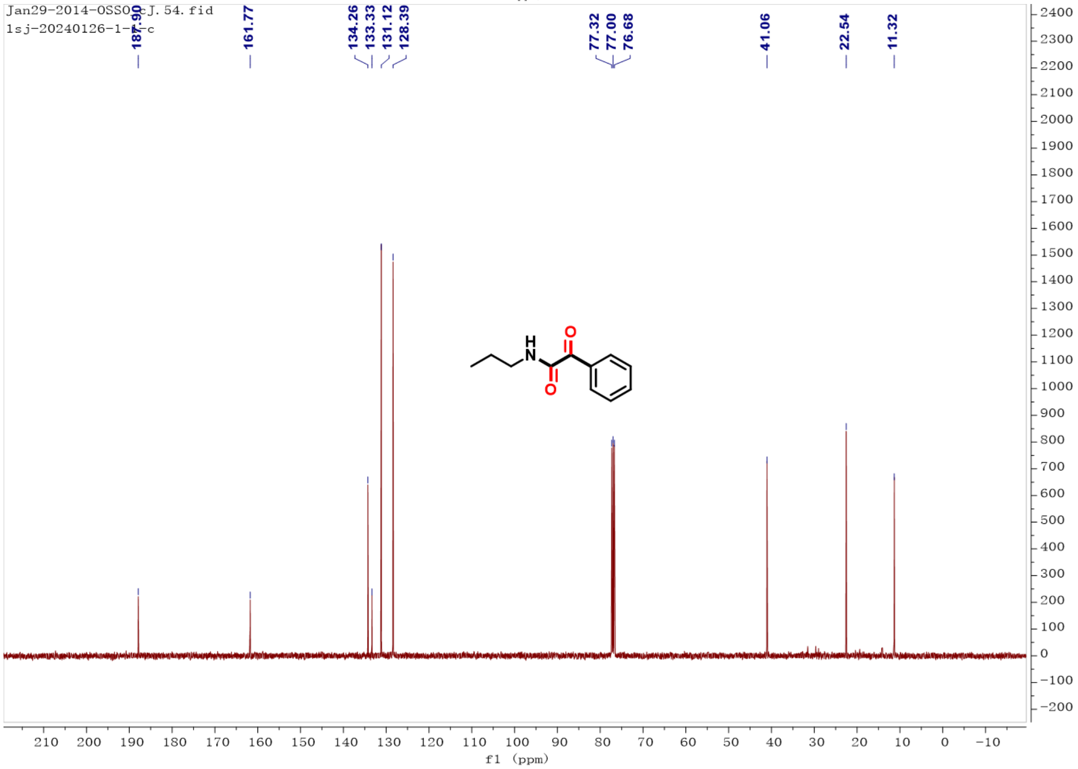

4u *N*-butyl-2-oxo-2-phenylacetamide

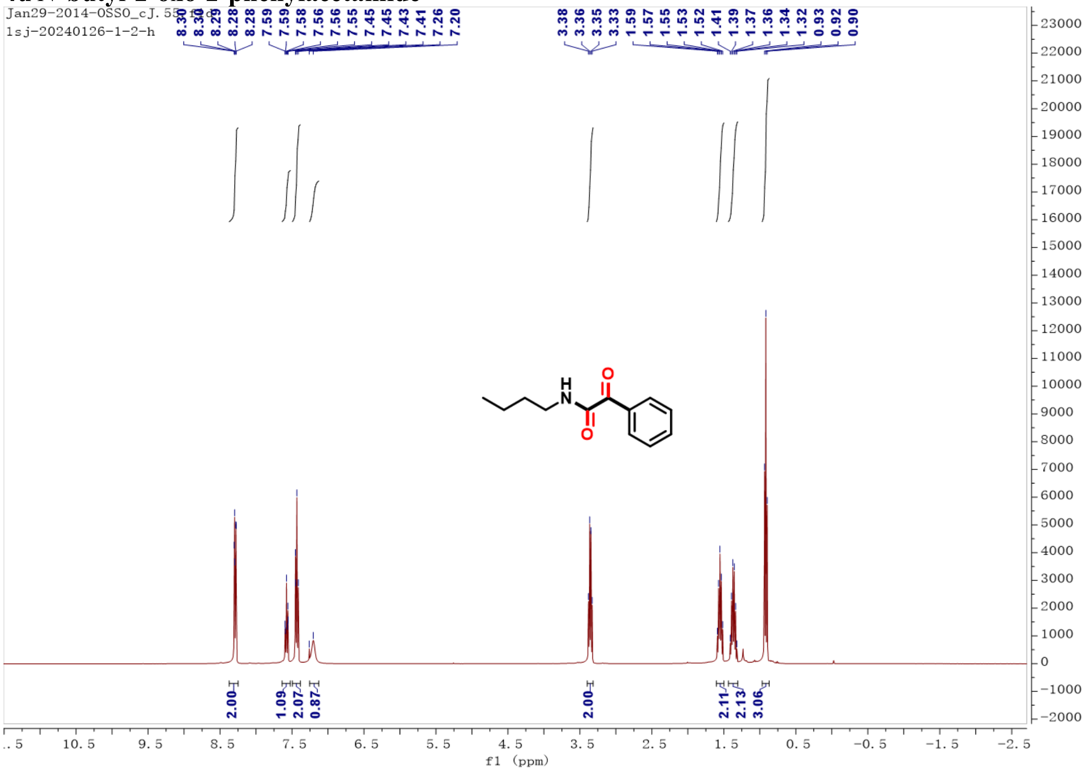

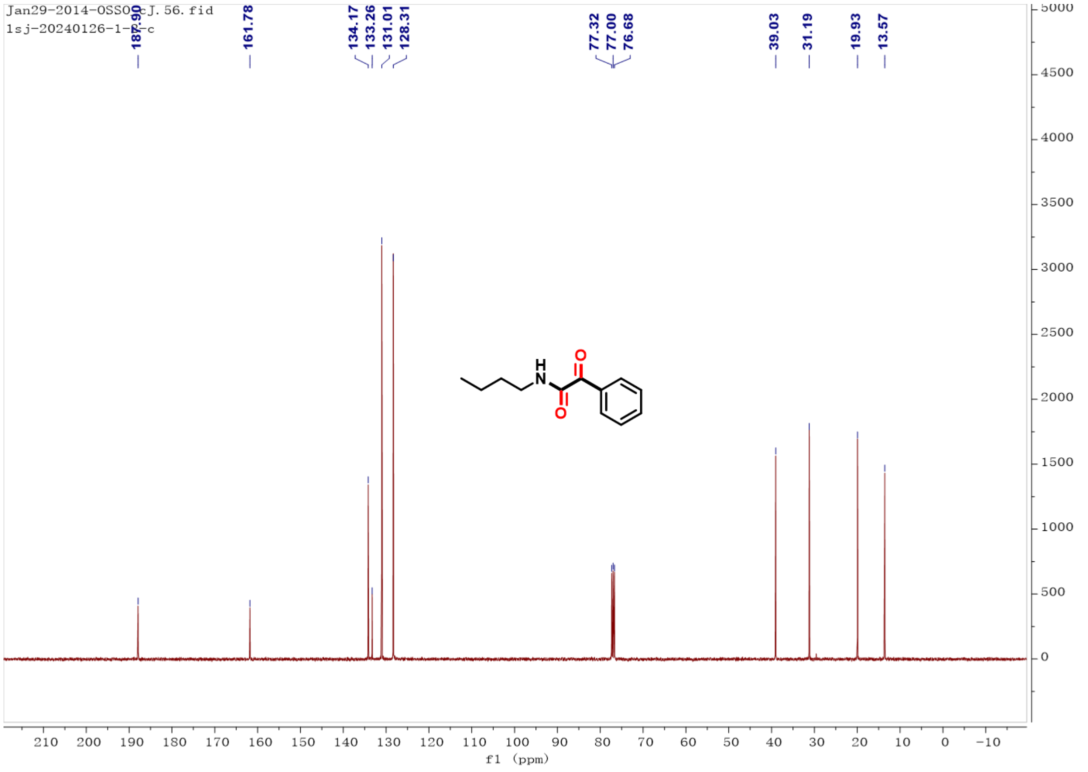

4v *N*-hexyl-2-oxo-2-phenylacetamide

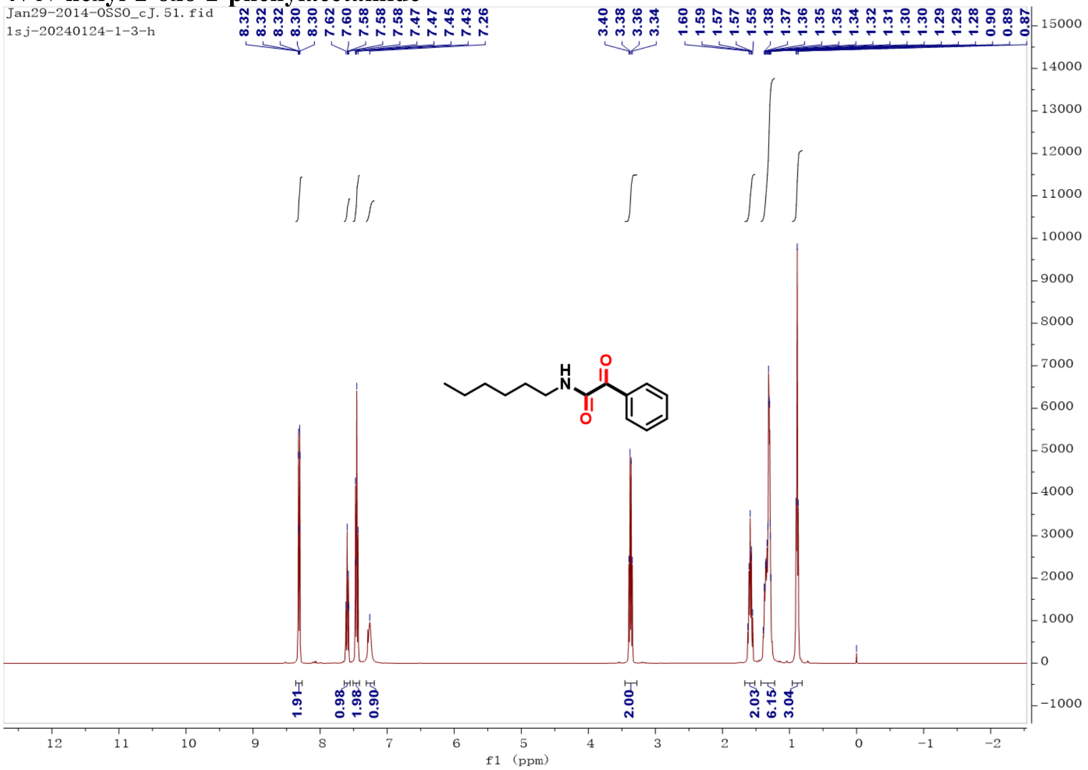

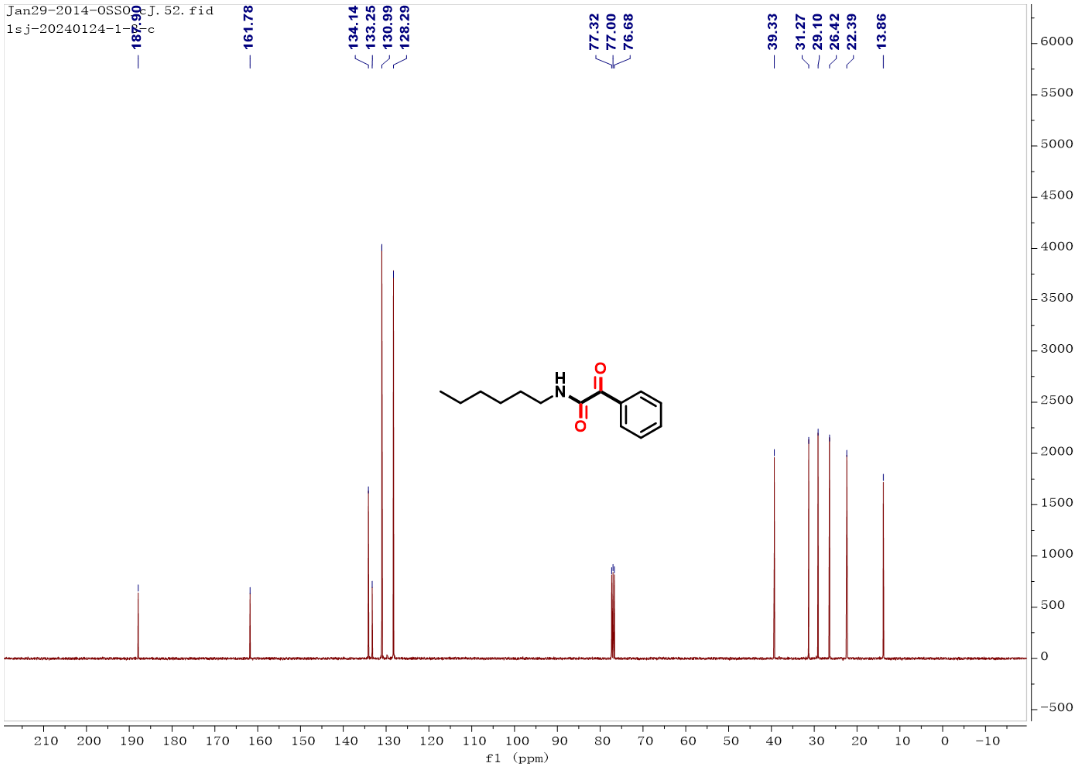

4w *N*-heptyl-2-oxo-2-phenylacetamide

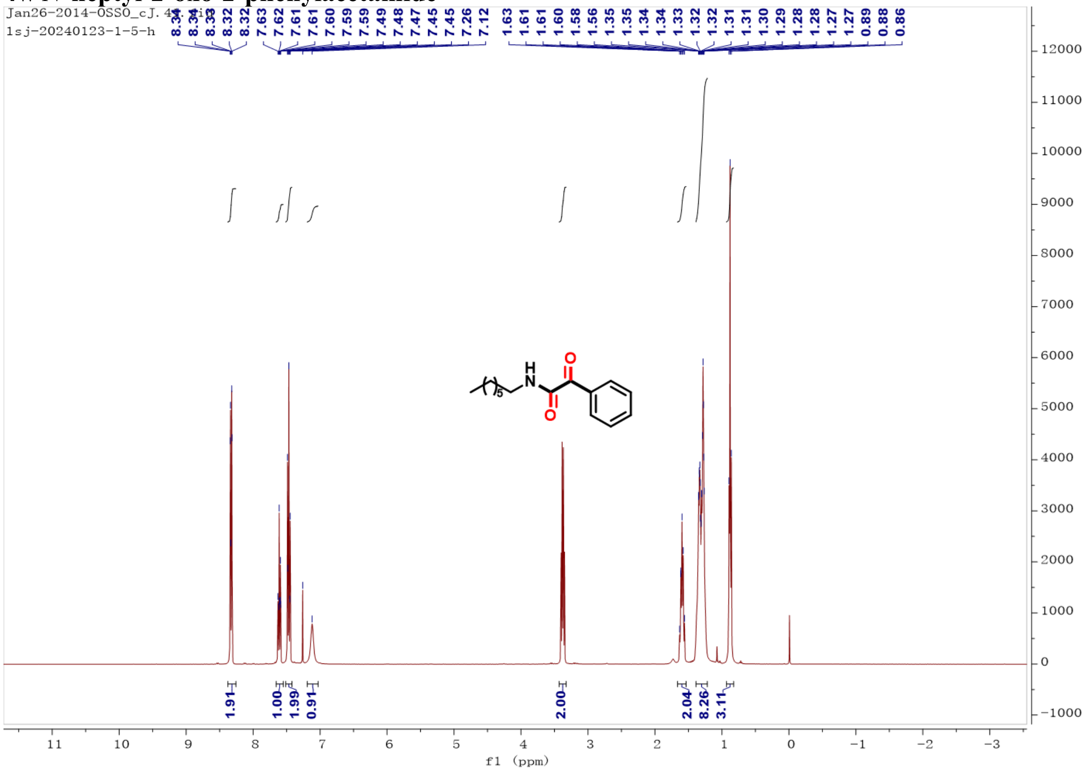

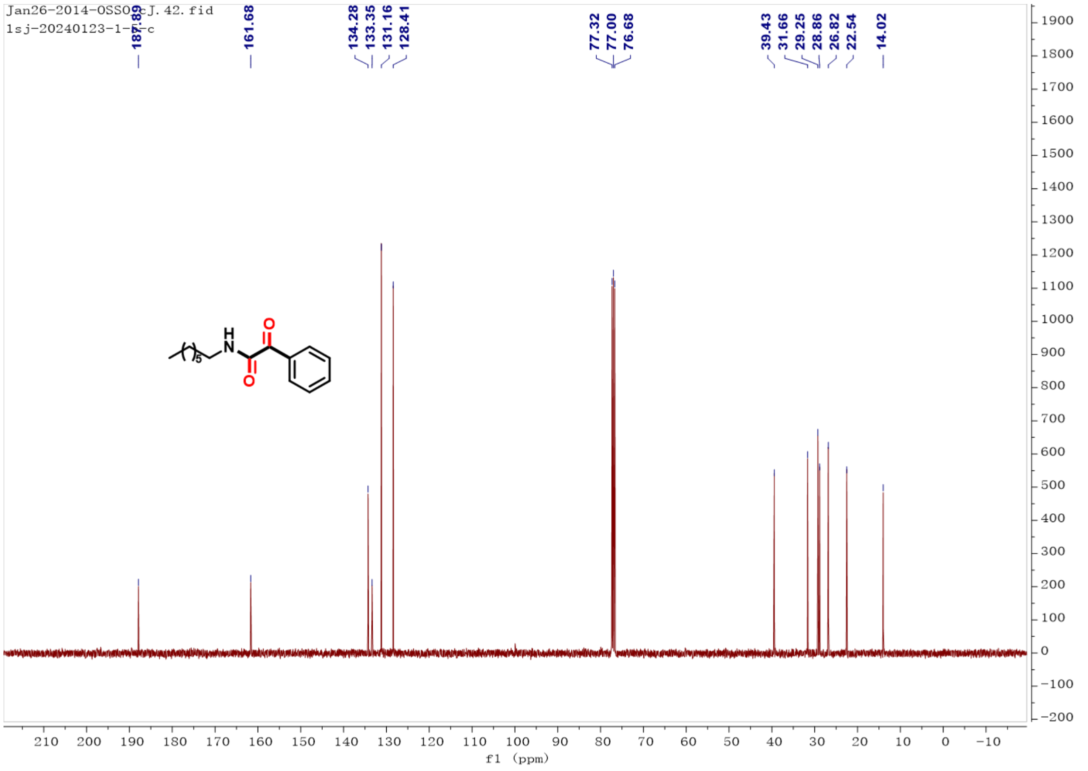

4x *N*-octyl-2-oxo-2-phenylacetamide

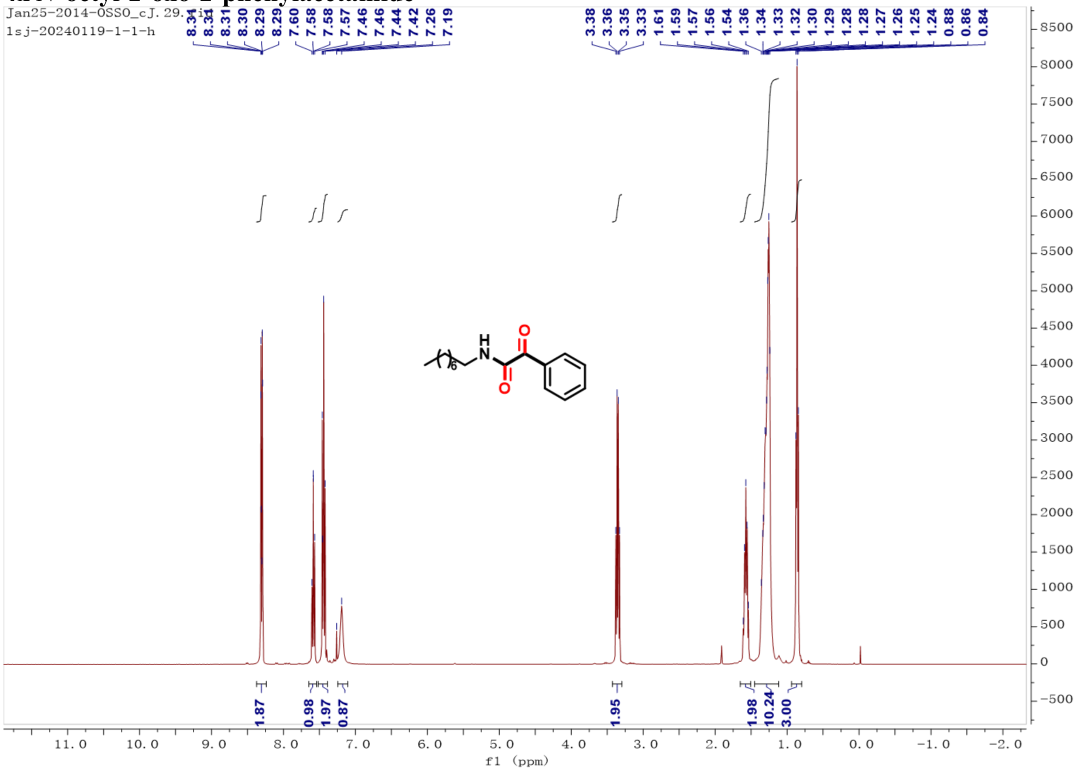

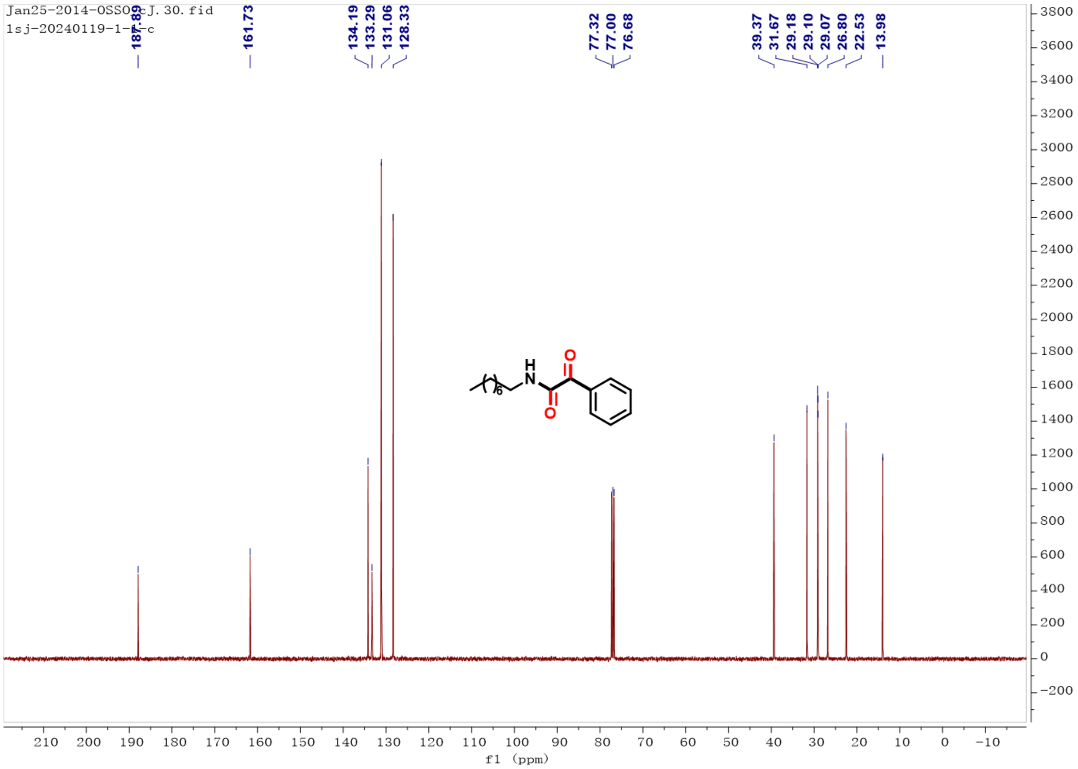

4y *N*-dodecyl-2-oxo-2-phenylacetamide

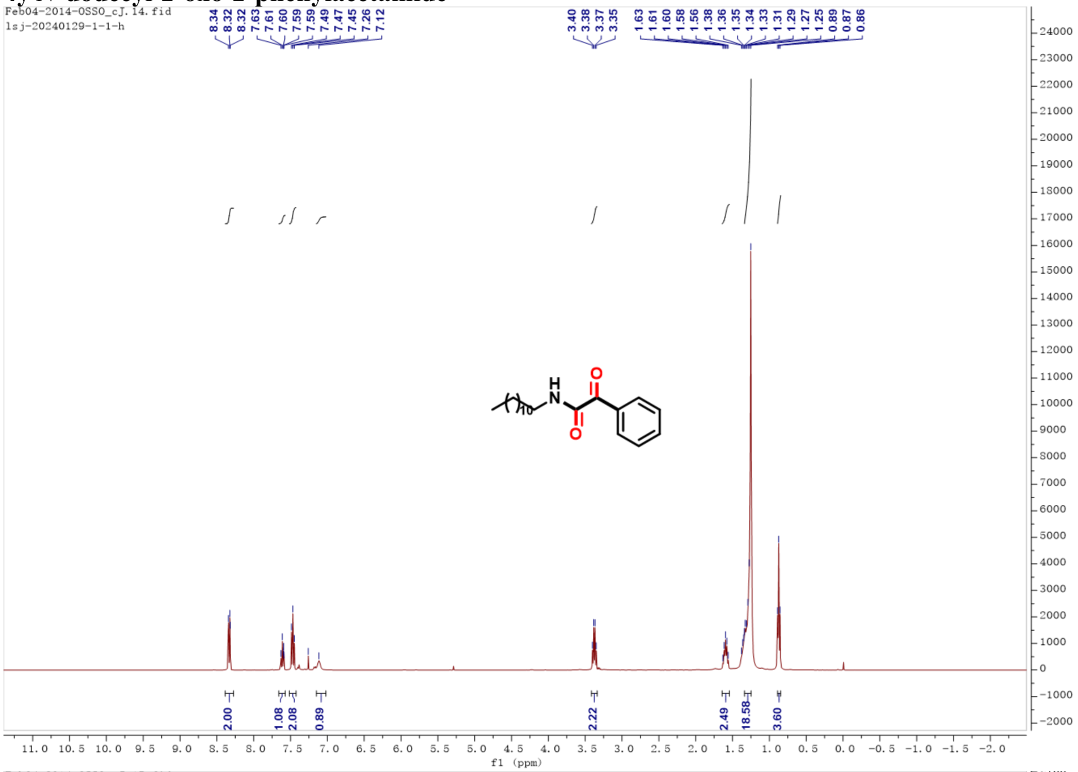

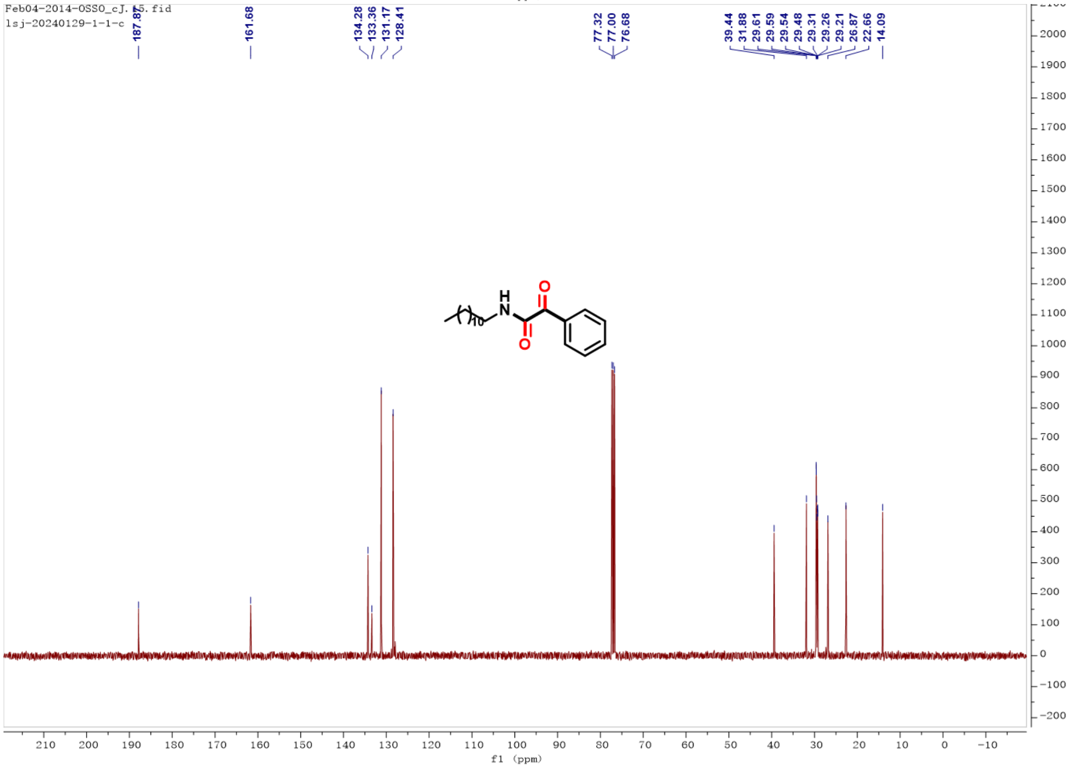

4z *N*-(sec-butyl)-2-oxo-2-phenylacetamide

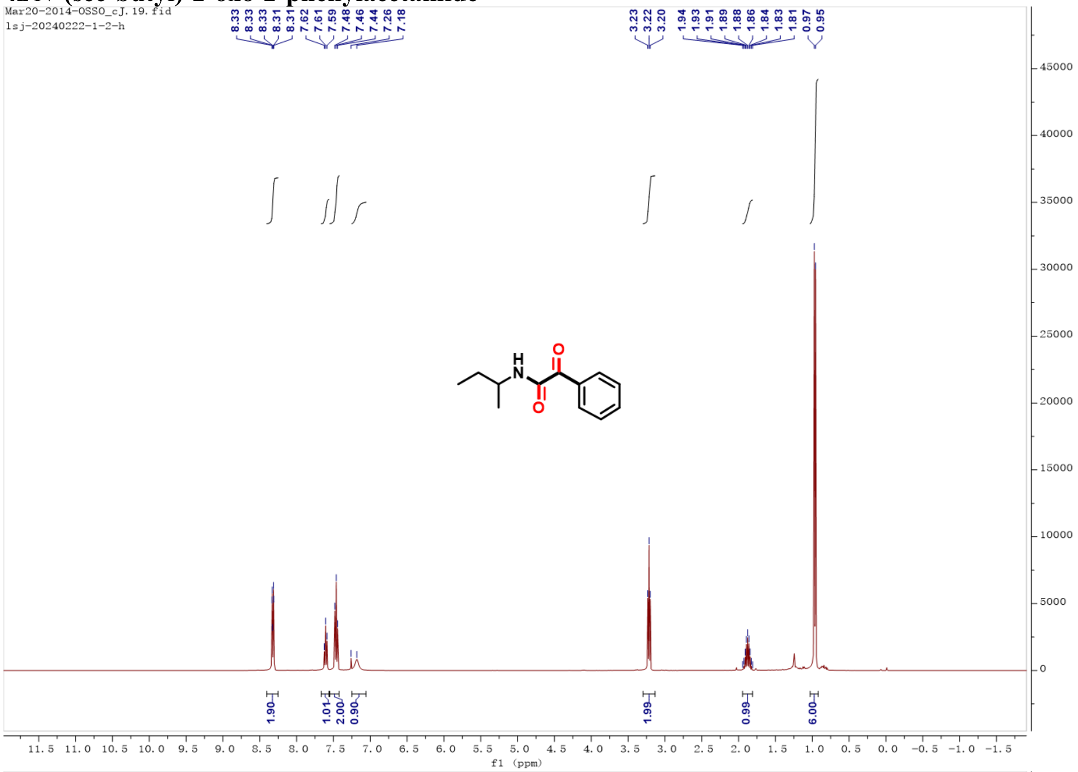

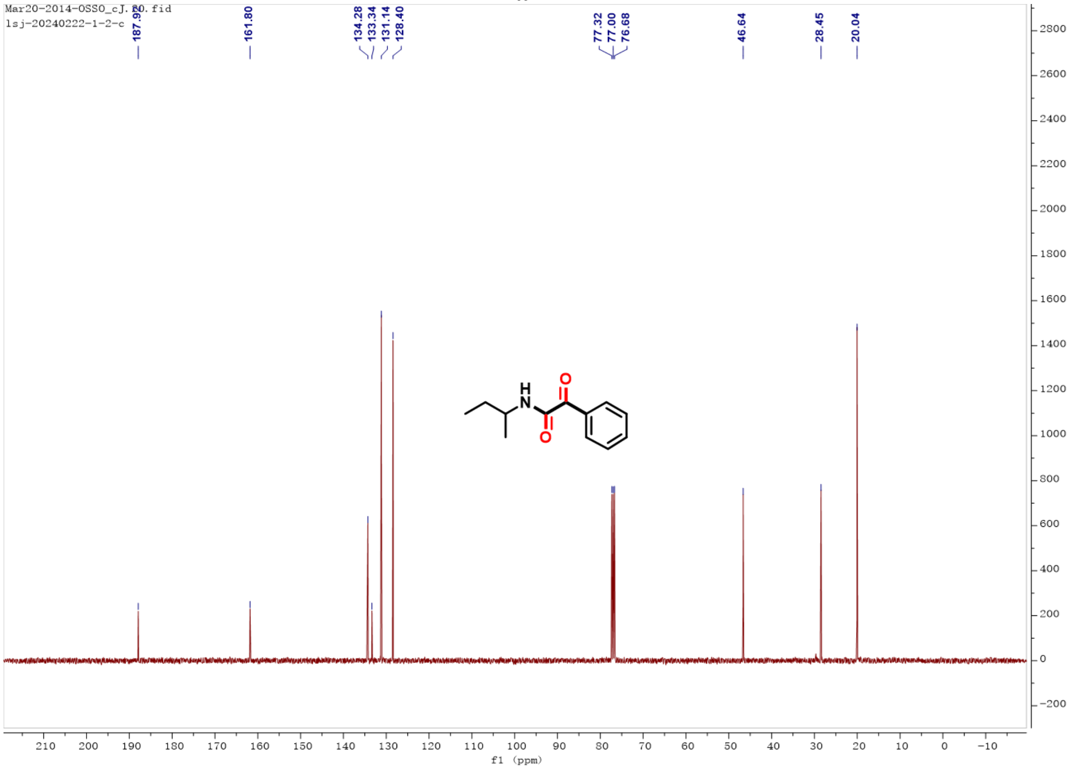

4aa *N*-cyclopentyl-2-oxo-2-phenylacetamide

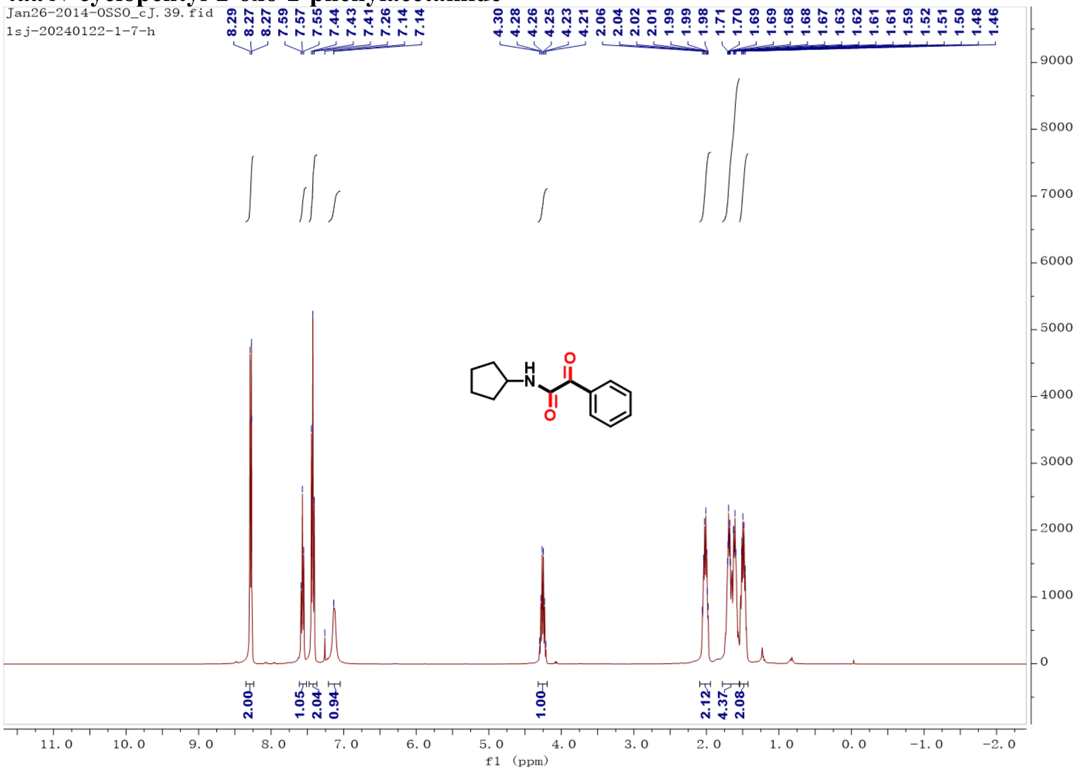

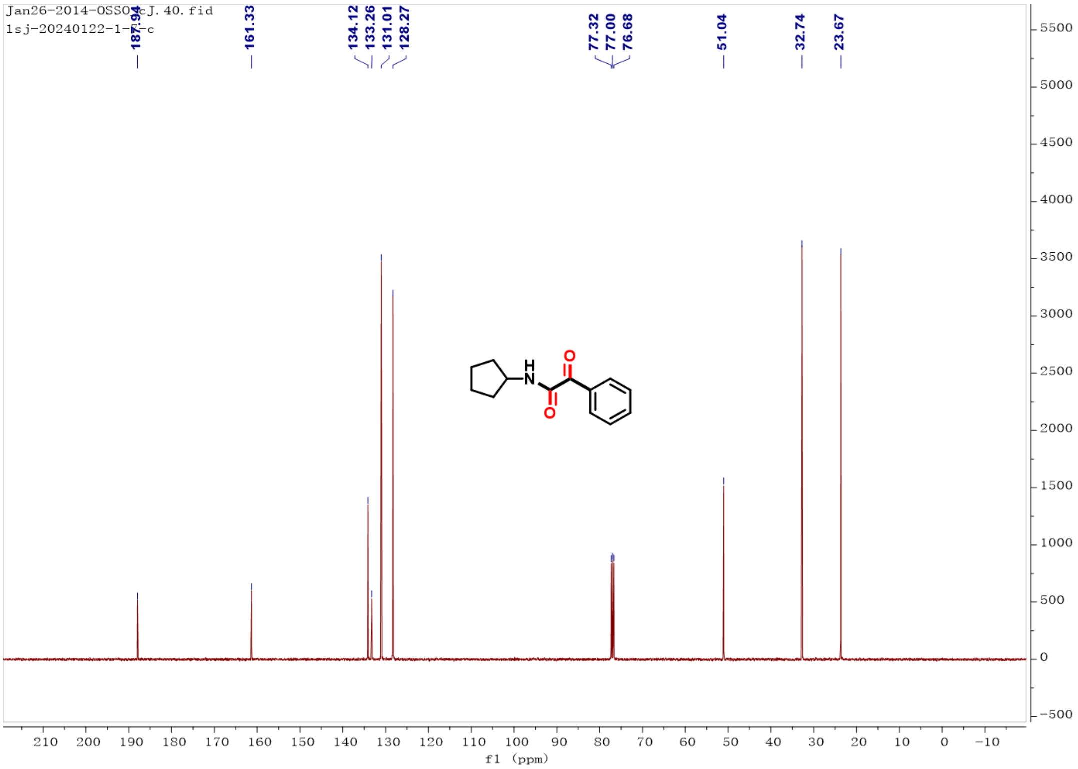

4ab *N*-cyclohexyl-2-oxo-2-phenylacetamide

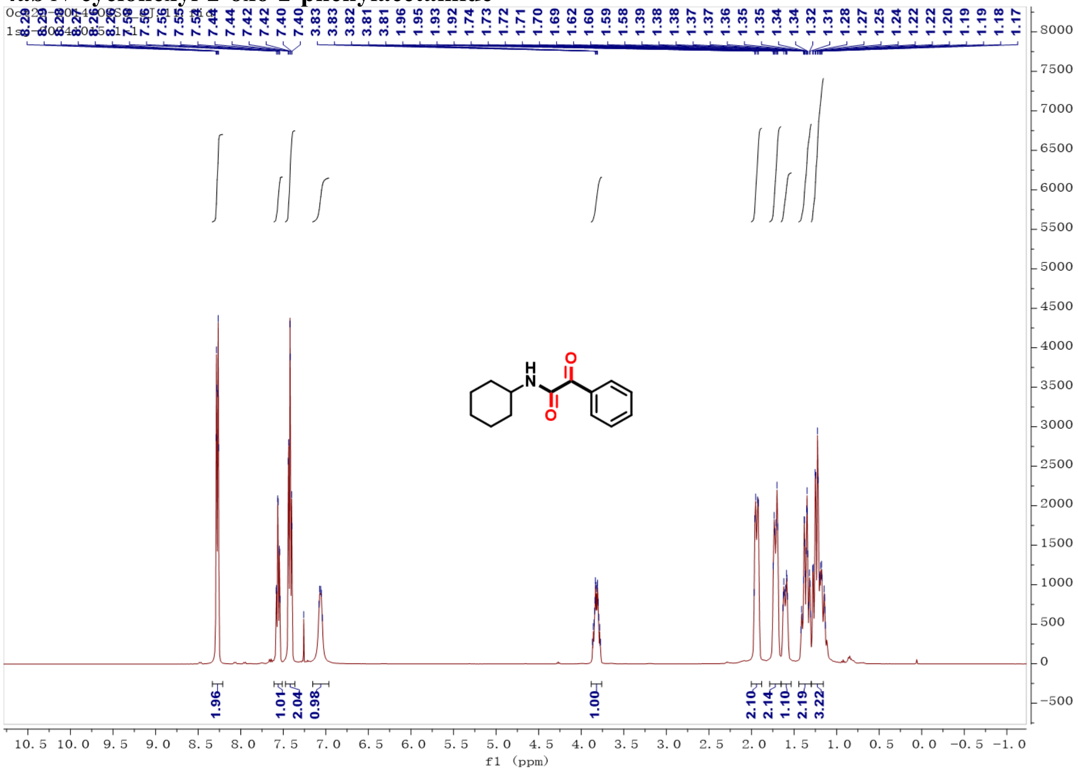

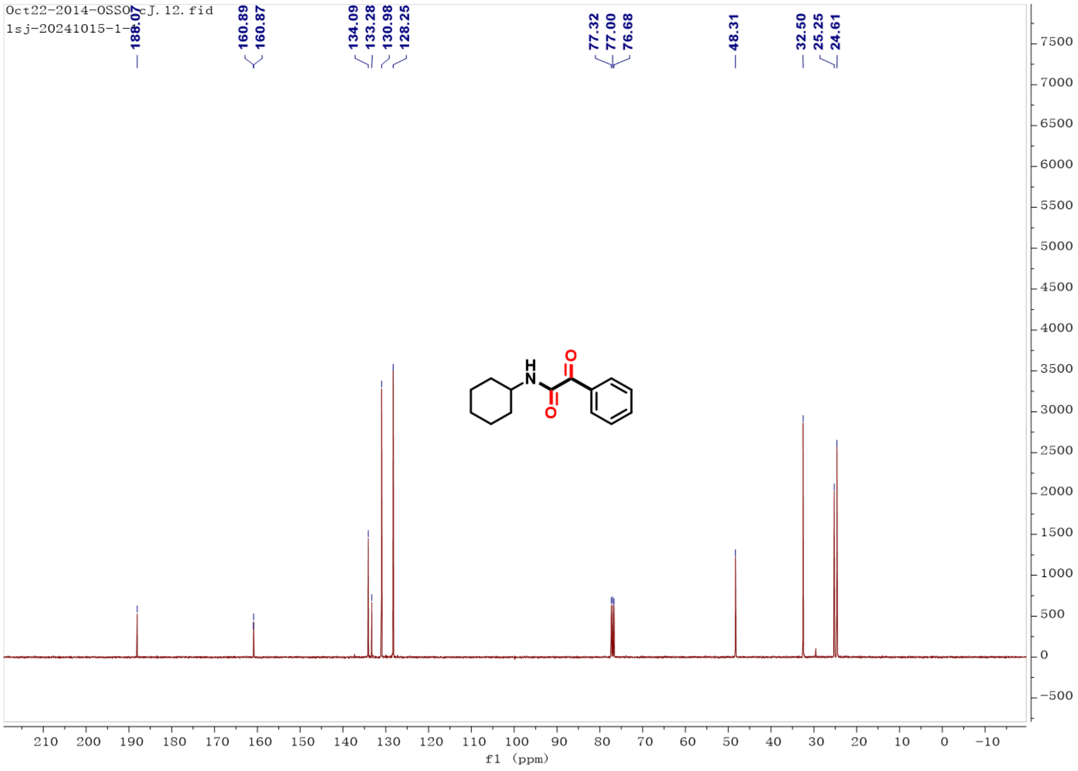

4ac N-(adamantan-1-yl)-2-oxo-2-phenylacetamide

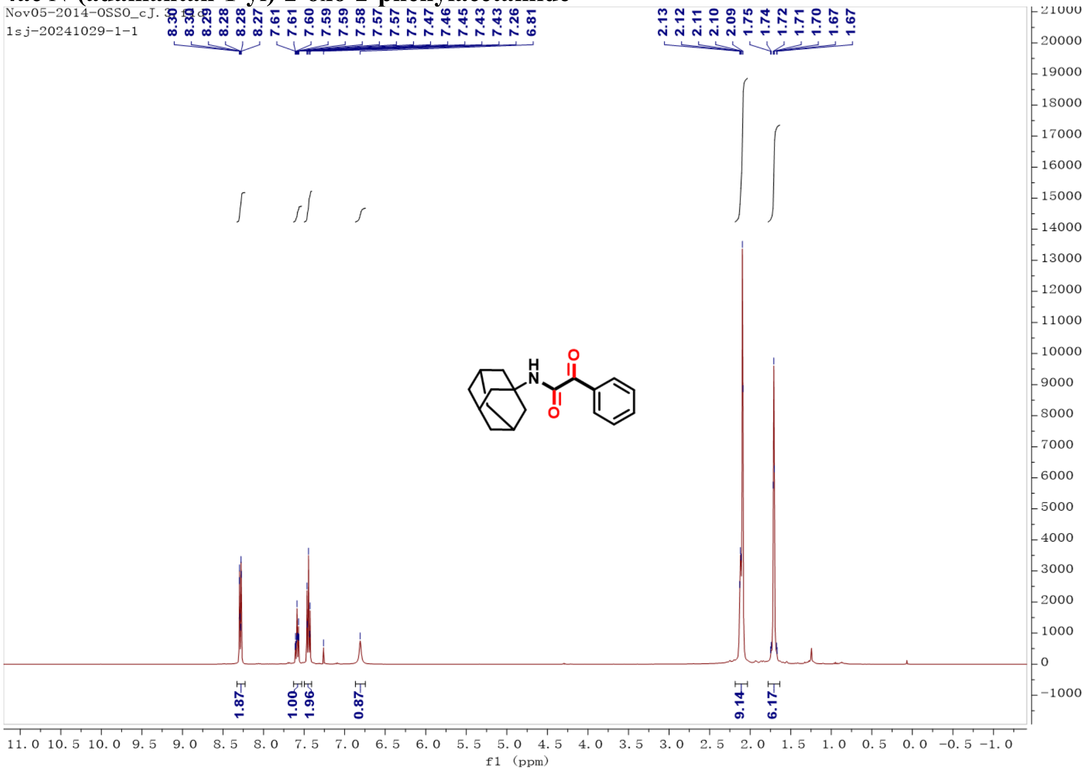

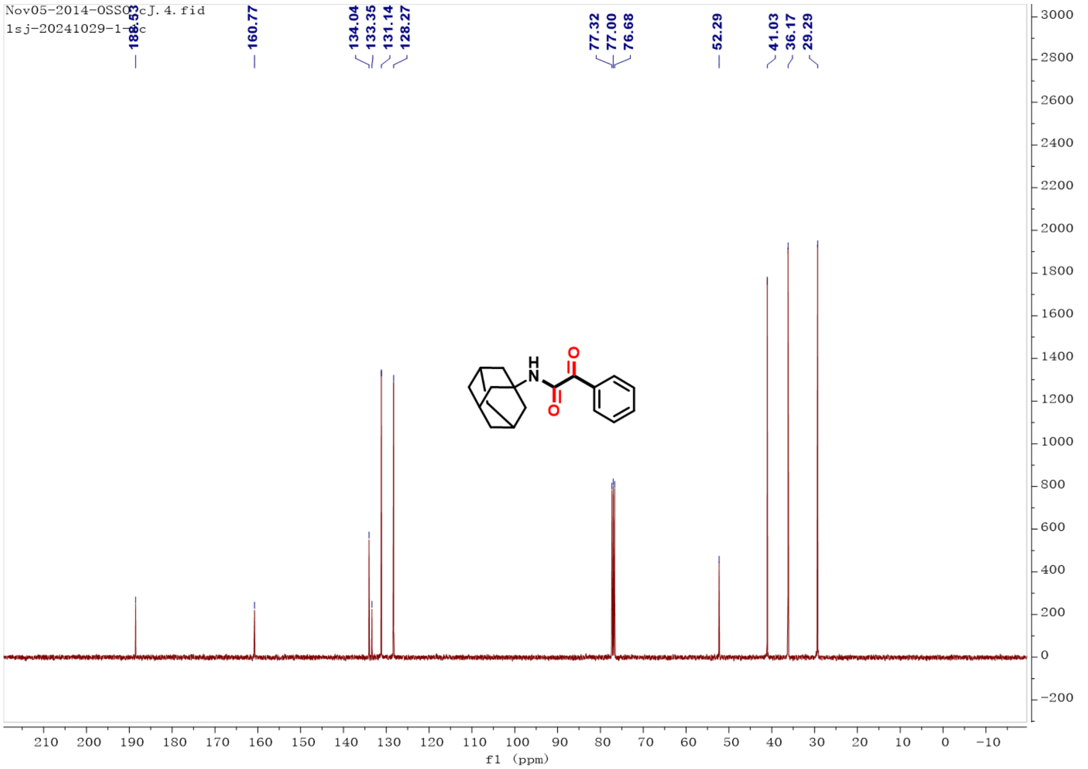

4ad *N*-(cyclopropylmethyl)-2-oxo-2-phenylacetamide

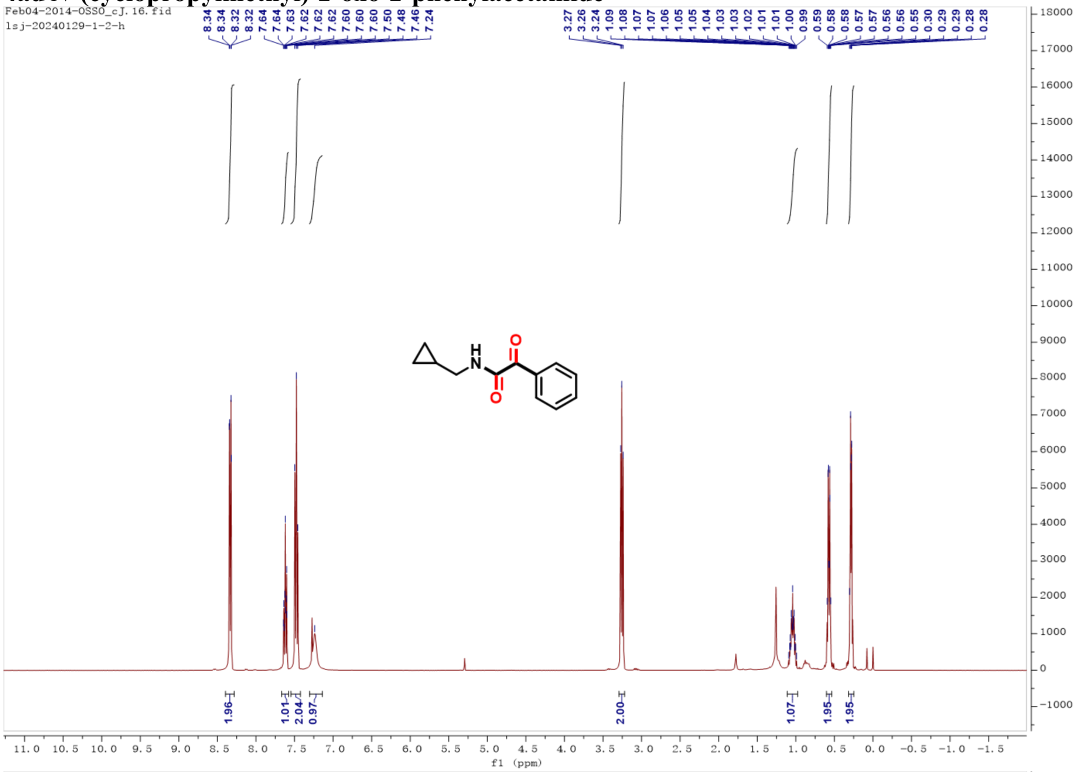

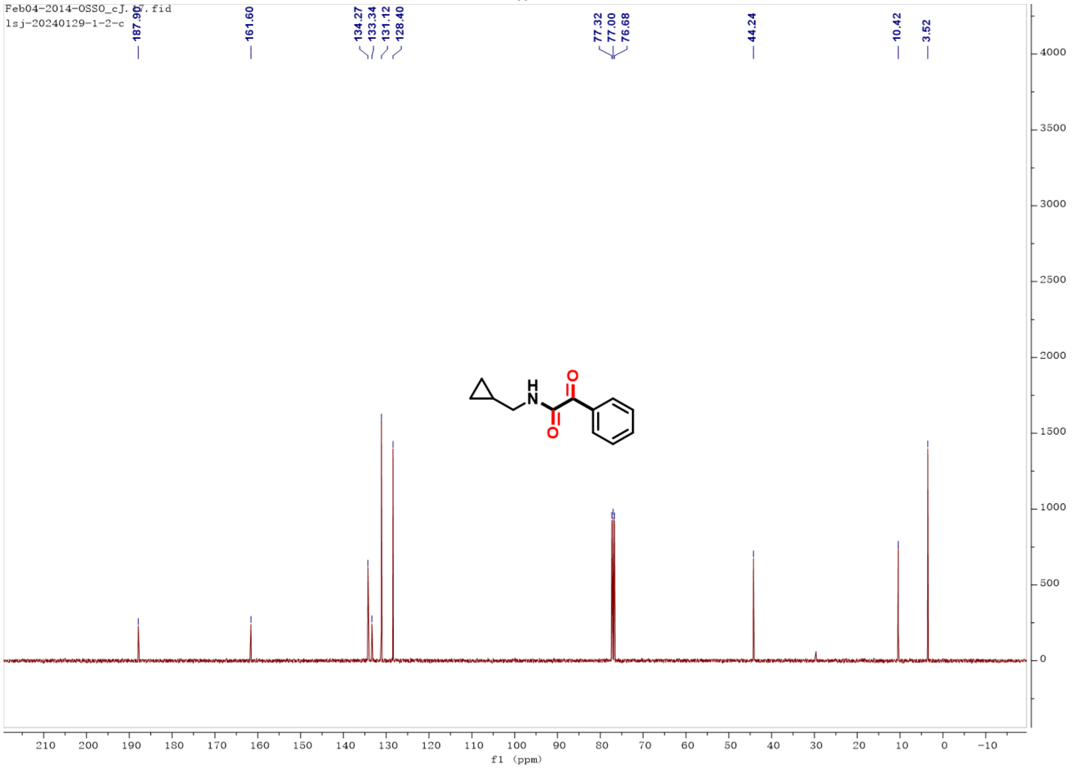

4ae *N*-(cyclohexylmethyl)-2-oxo-2-phenylacetamide

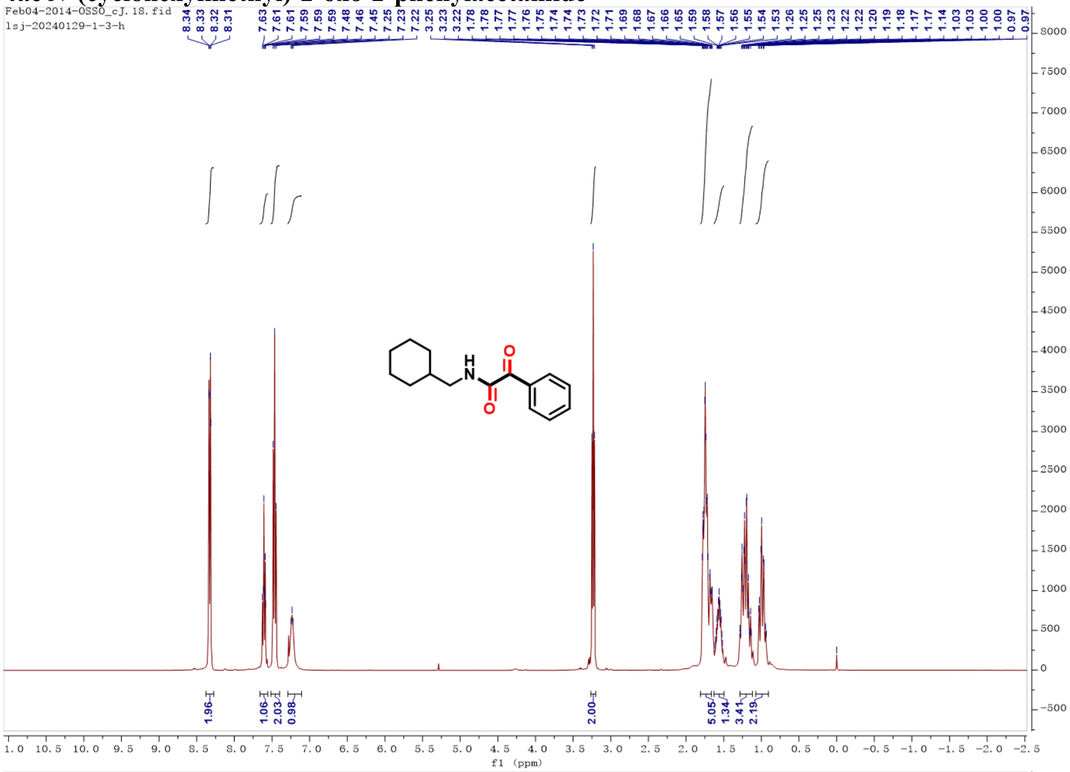

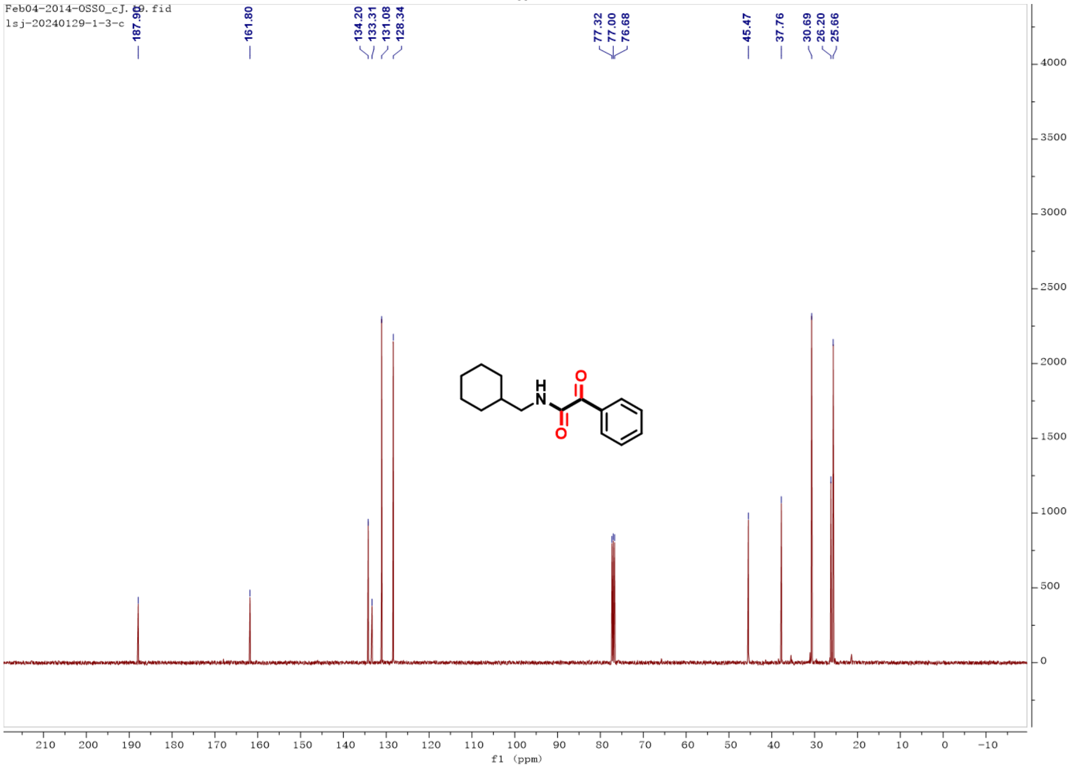

4af 2-oxo-2-phenyl-*N*-((tetrahydro-2*H*-pyran-4-yl)methyl)acetamide

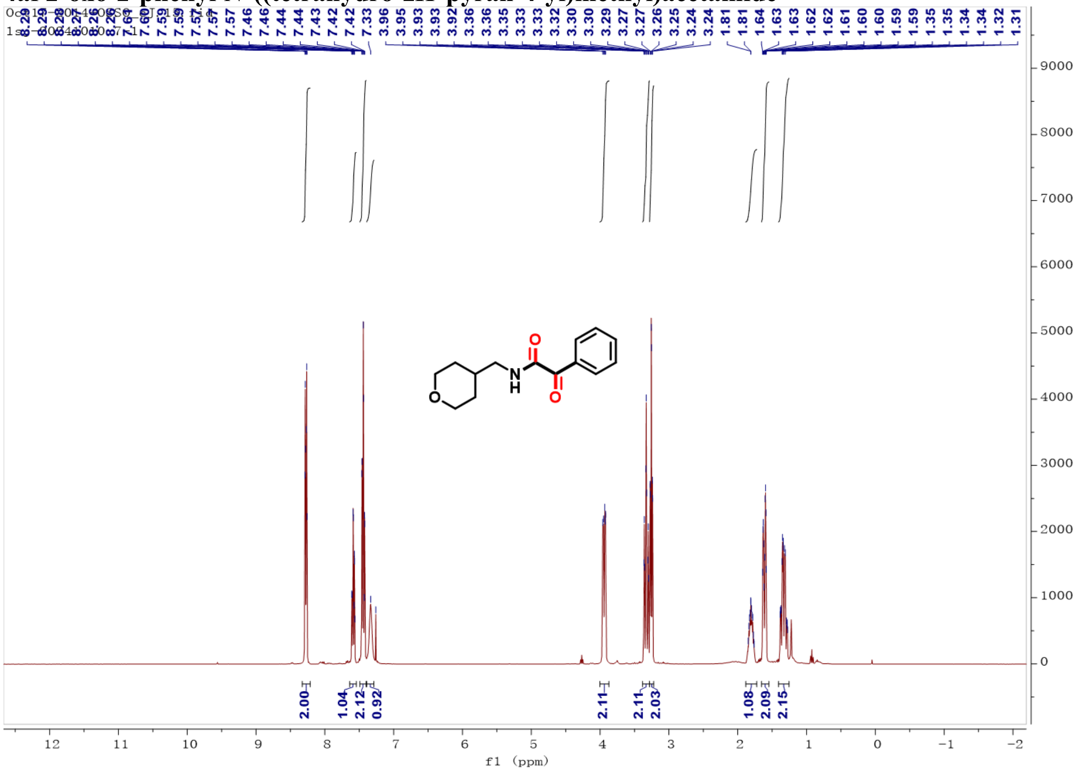

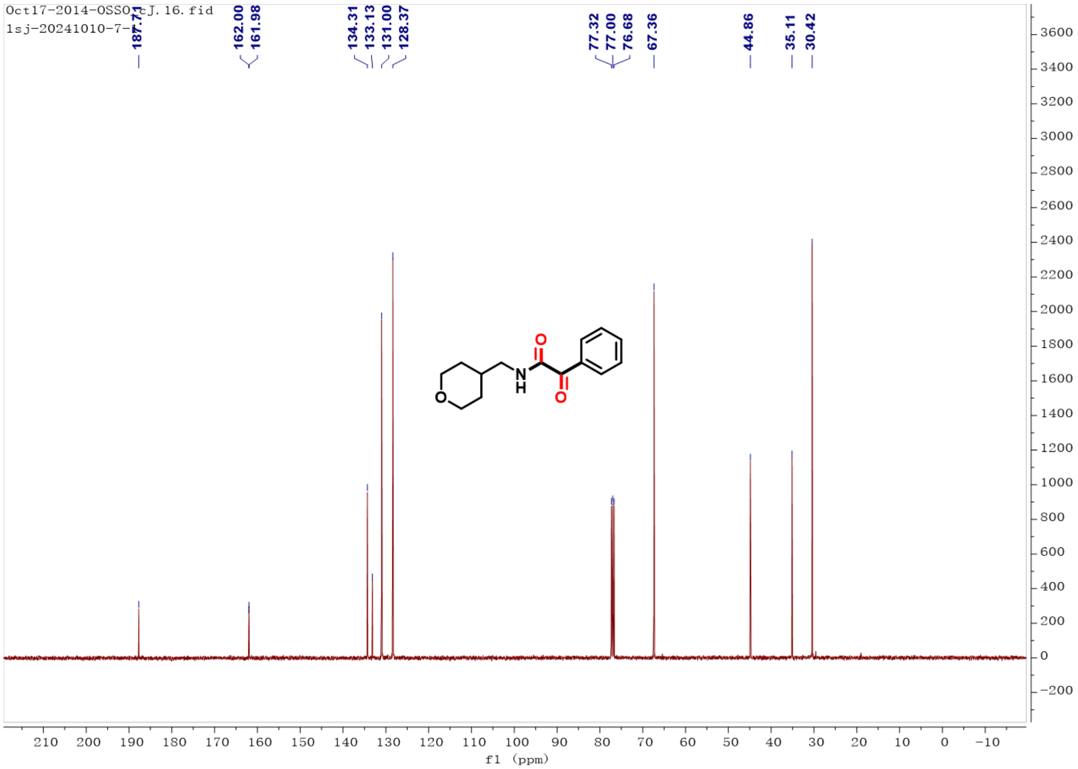

4ag *N*-(furan-2-ylmethyl)-2-oxo-2-phenylacetamide

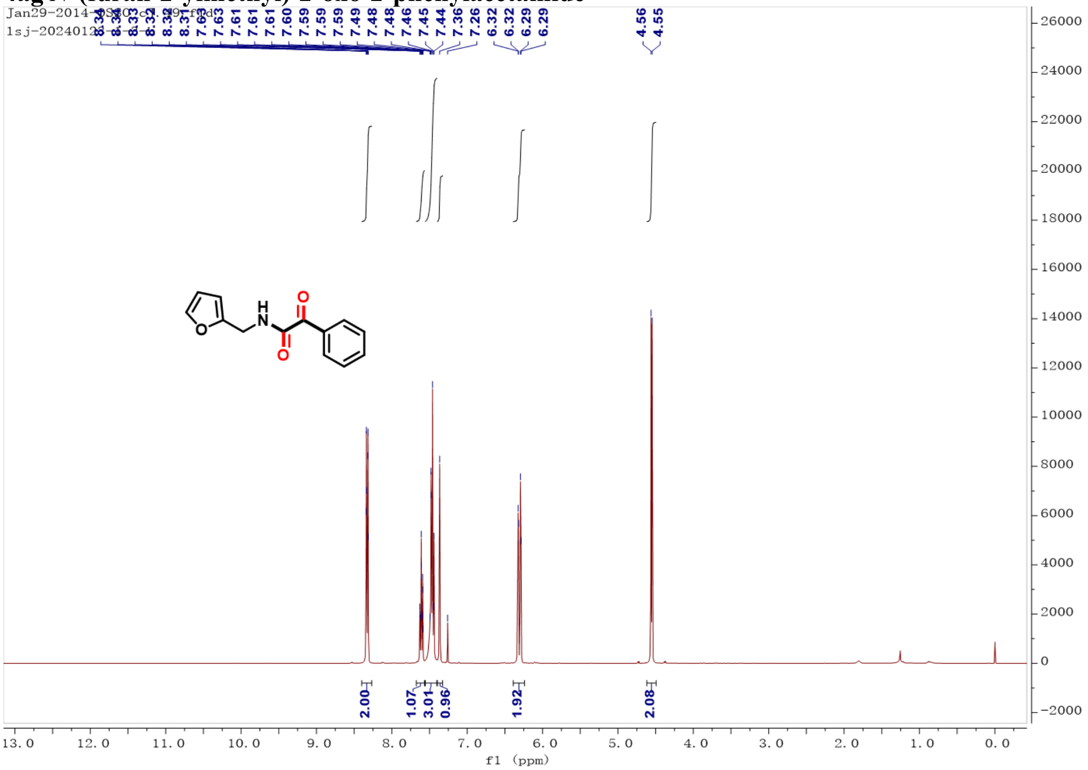

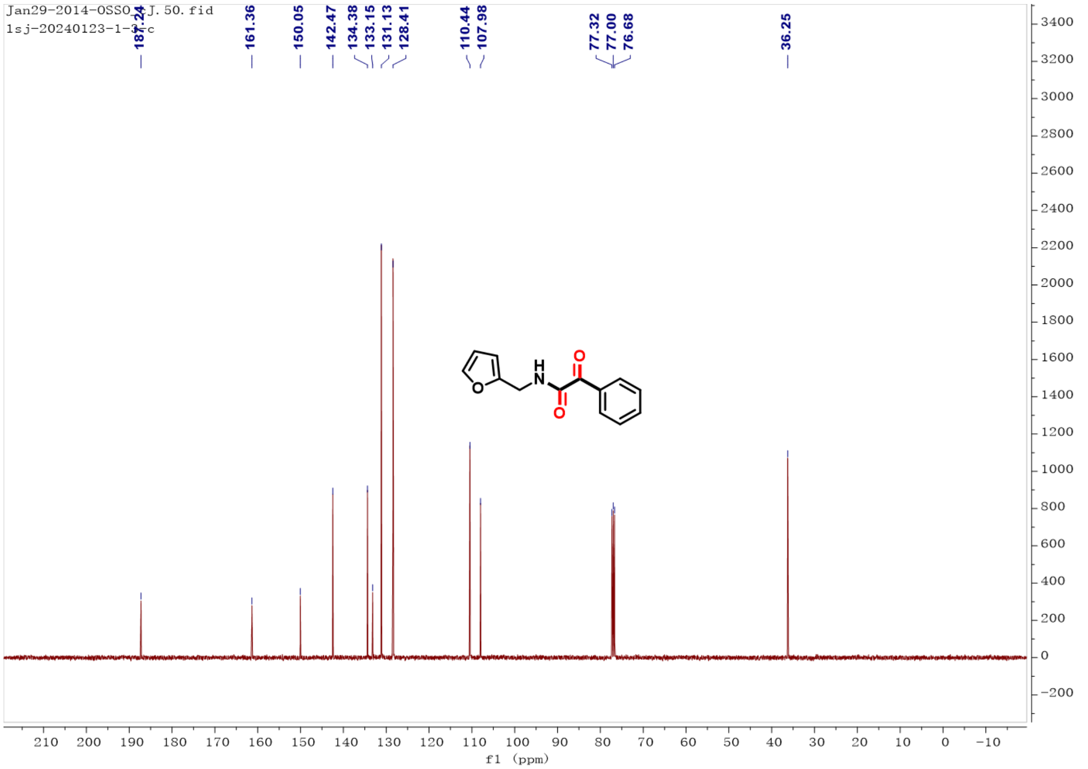

4ah 2-oxo-2-phenyl-*N*-(pyridin-3-ylmethyl)acetamide

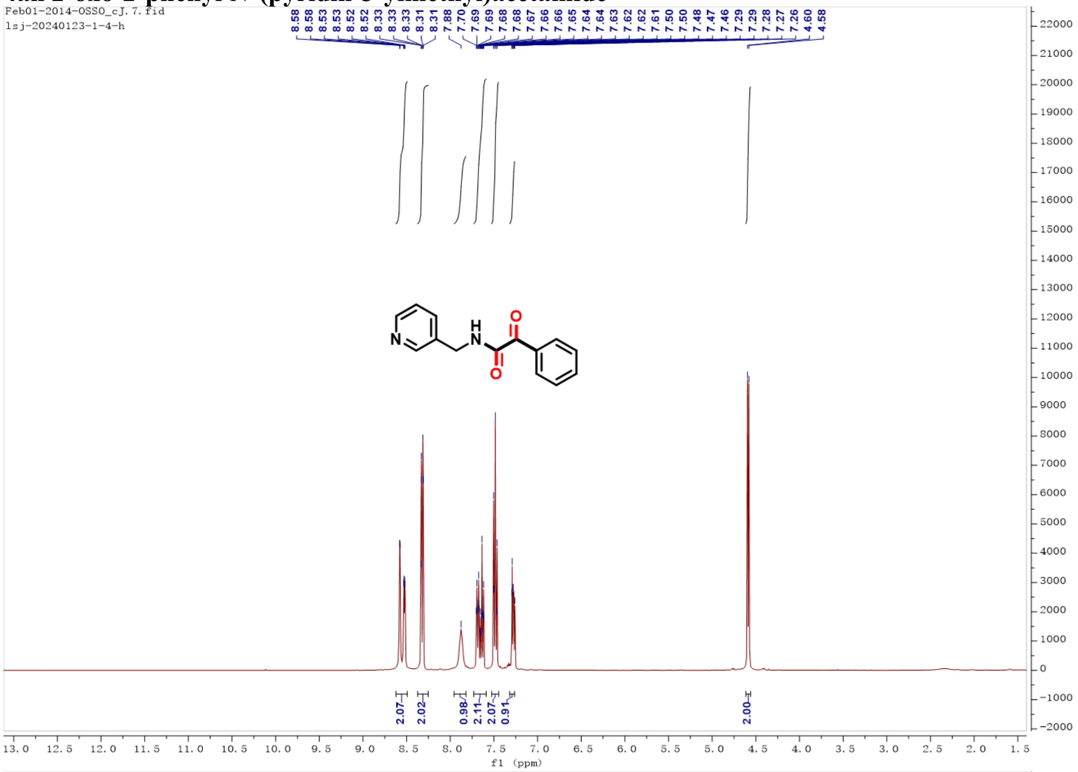

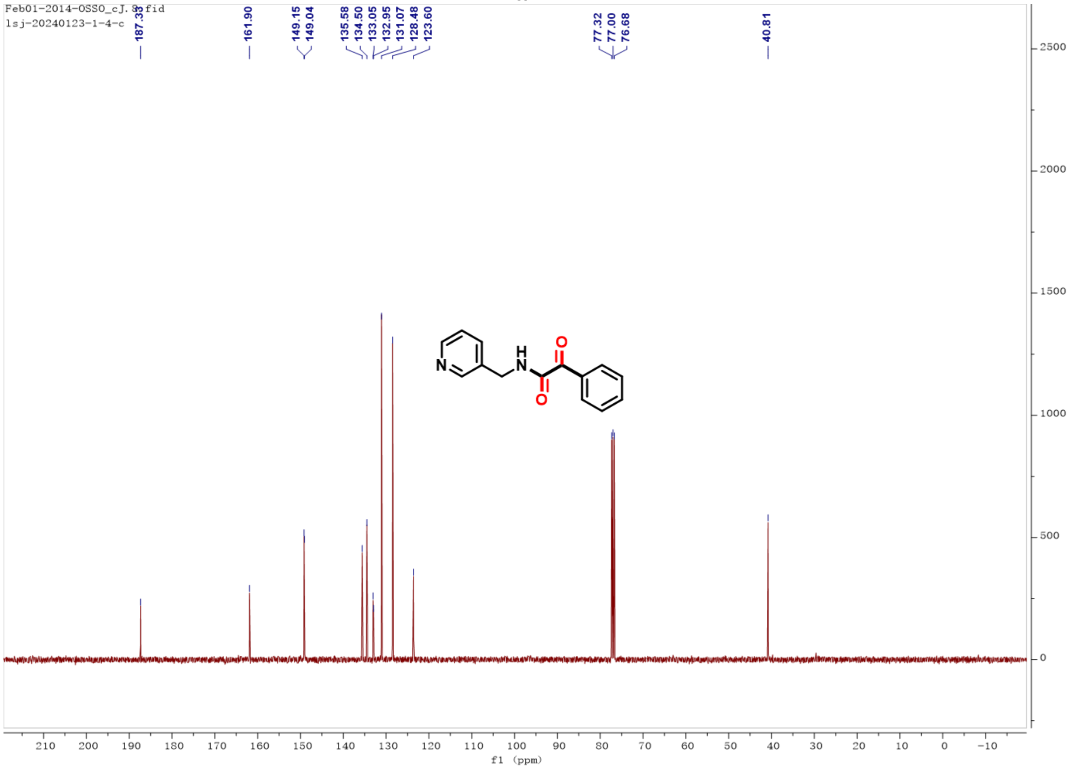

4ai 2-oxo-2-phenyl-*N*-(thiophen-2-ylmethyl)acetamide

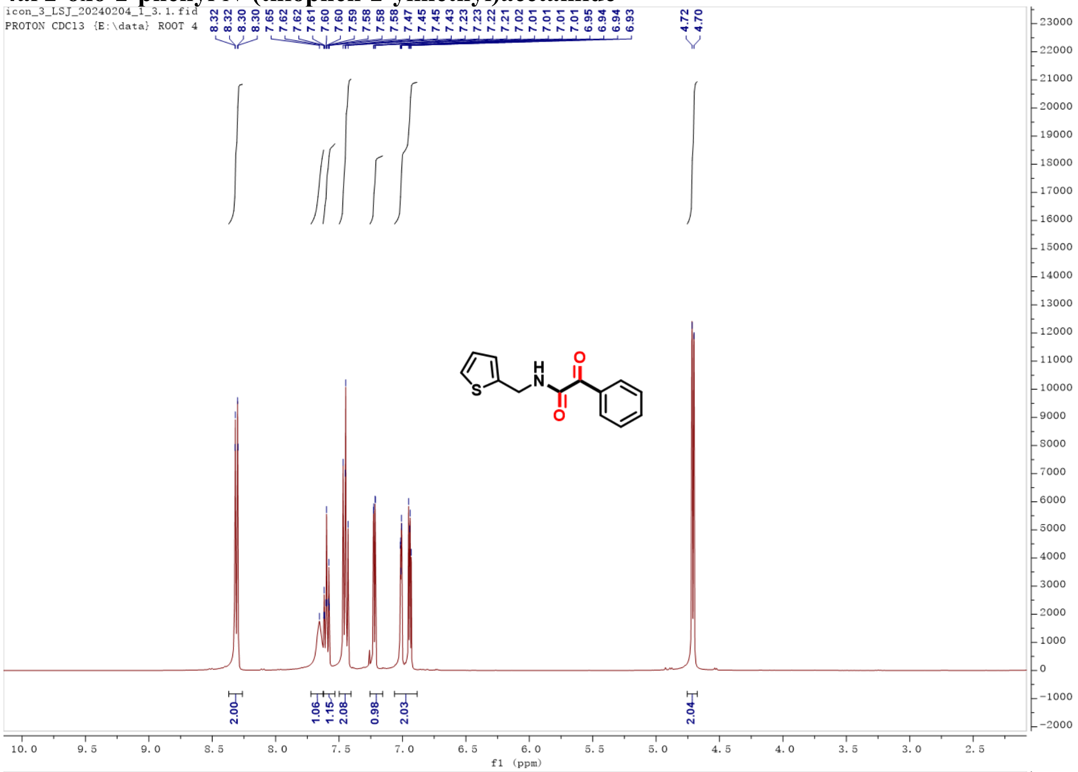

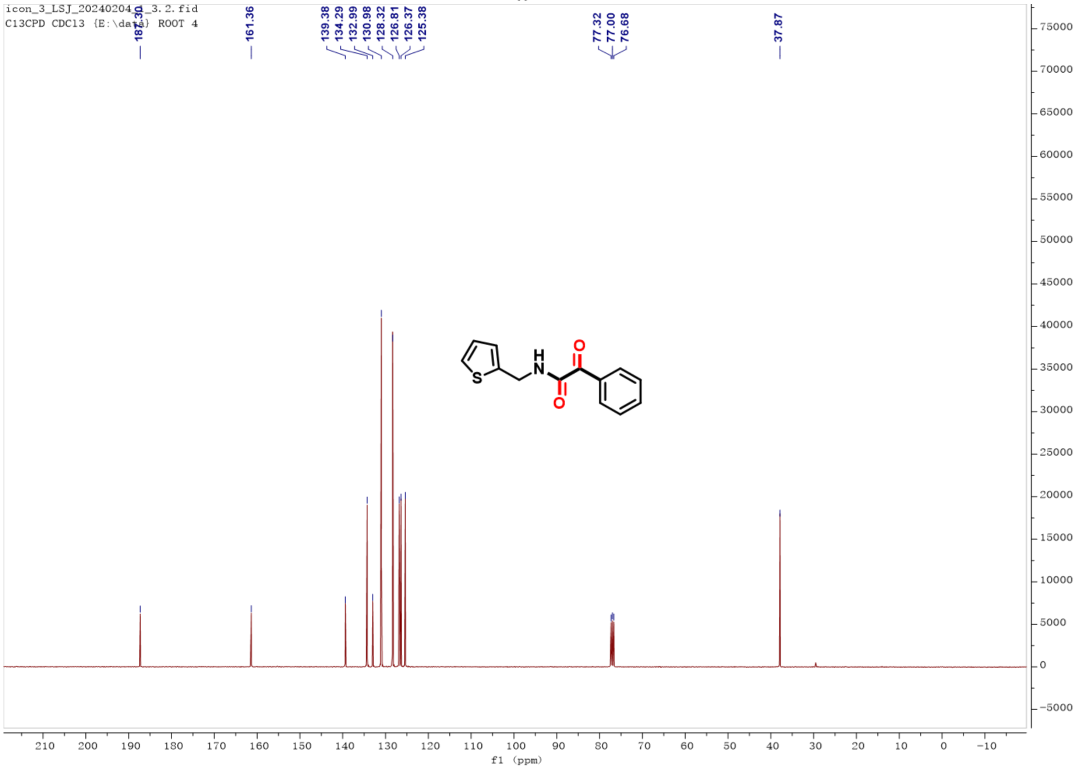

4aj *N*-benzyl-2-oxo-2-phenylacetamide

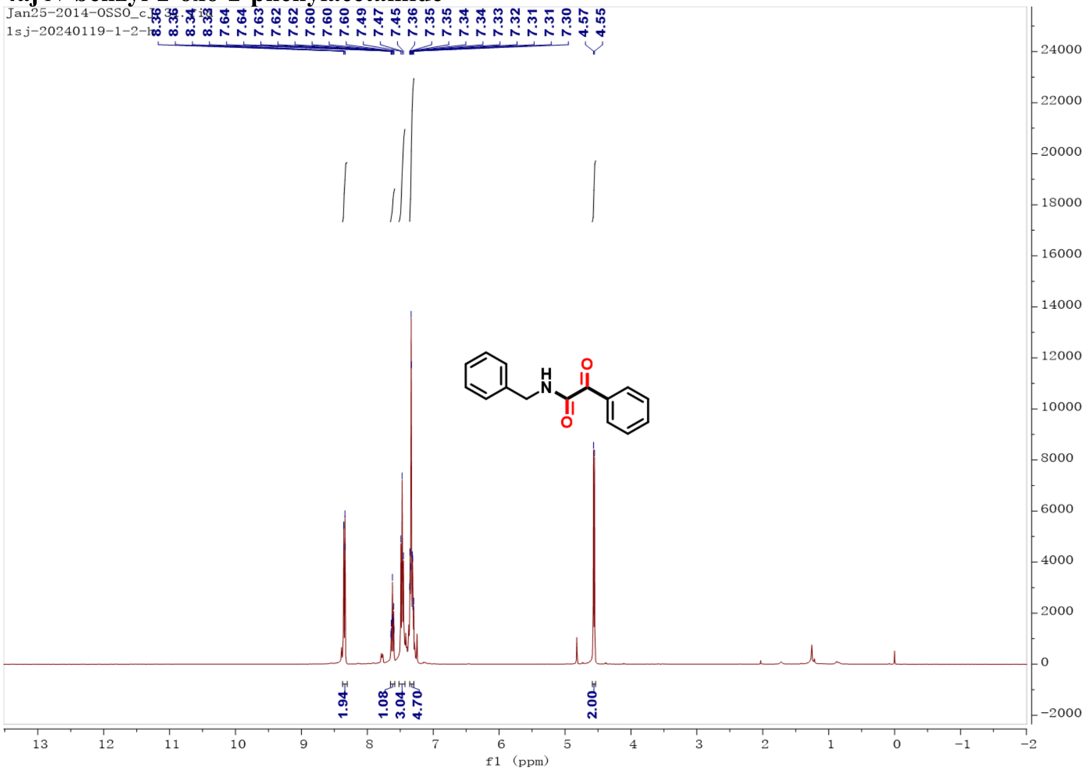

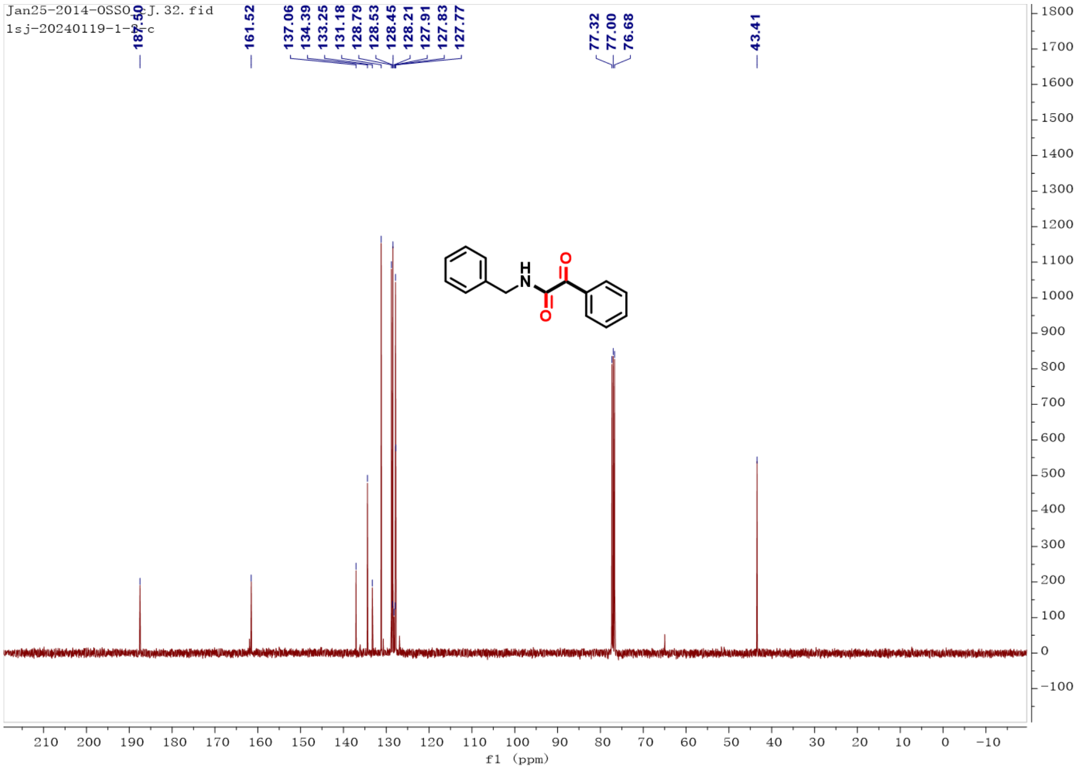

4ak 2-oxo-2-phenyl-*N*-(1-(p-tolyl)ethyl)acetamide

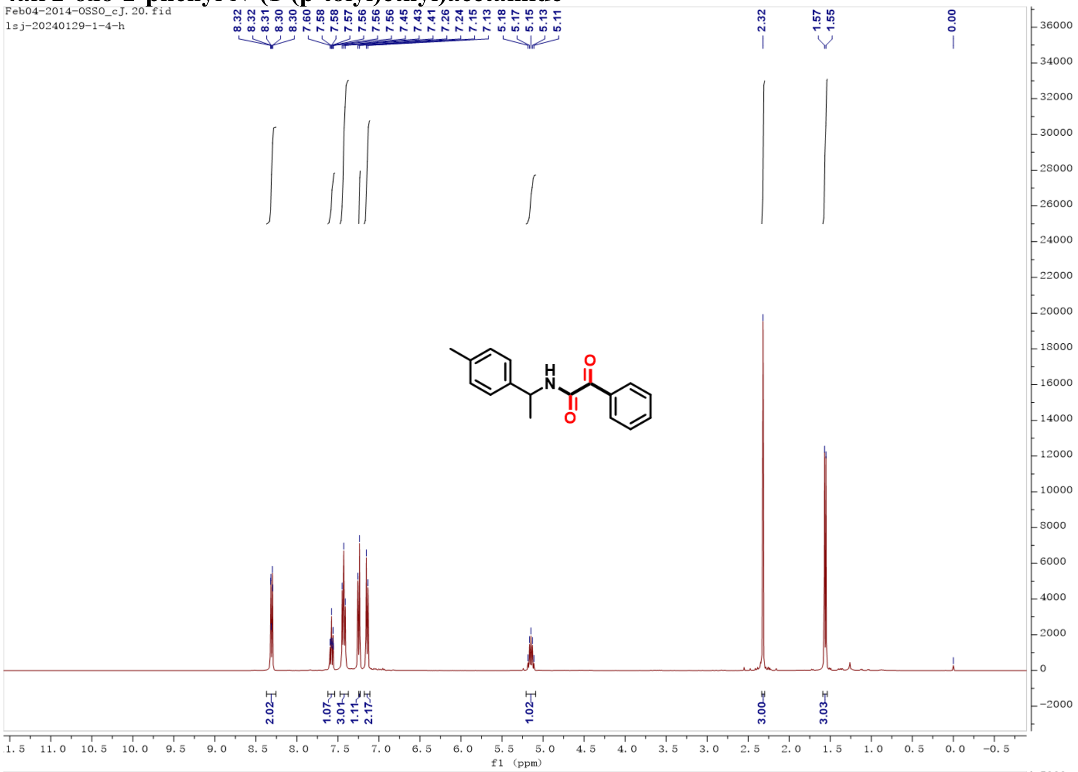

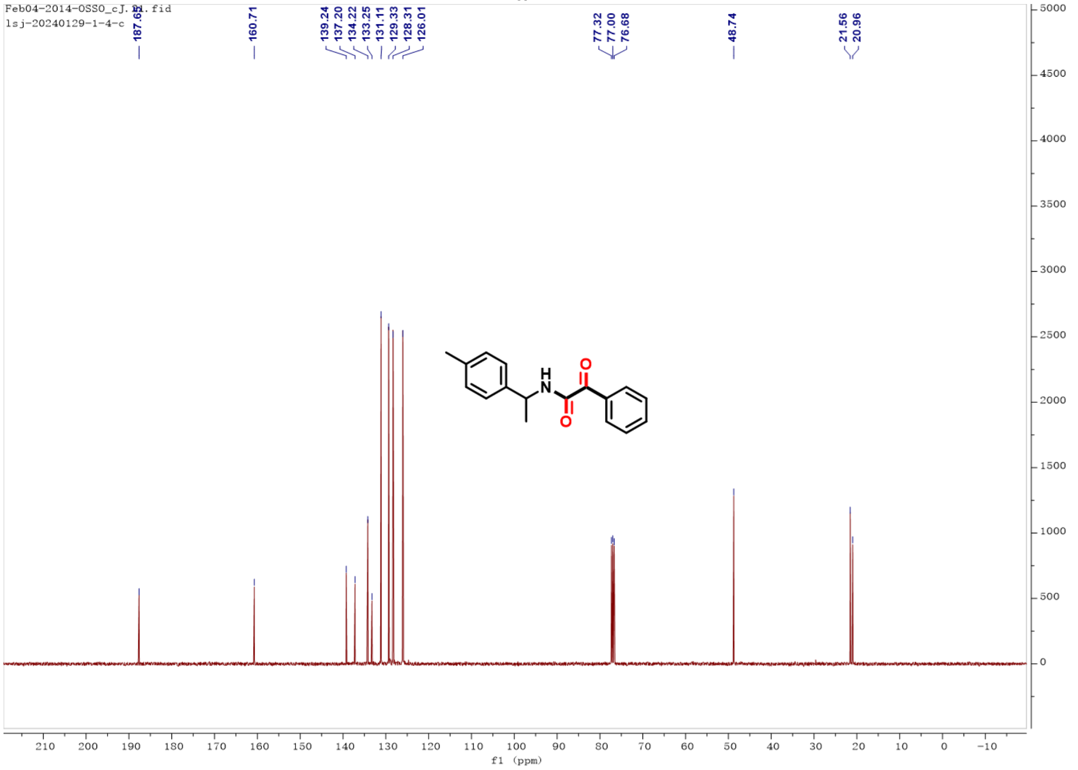

4al *N*-morpholino-2-oxo-2-phenylacetamide

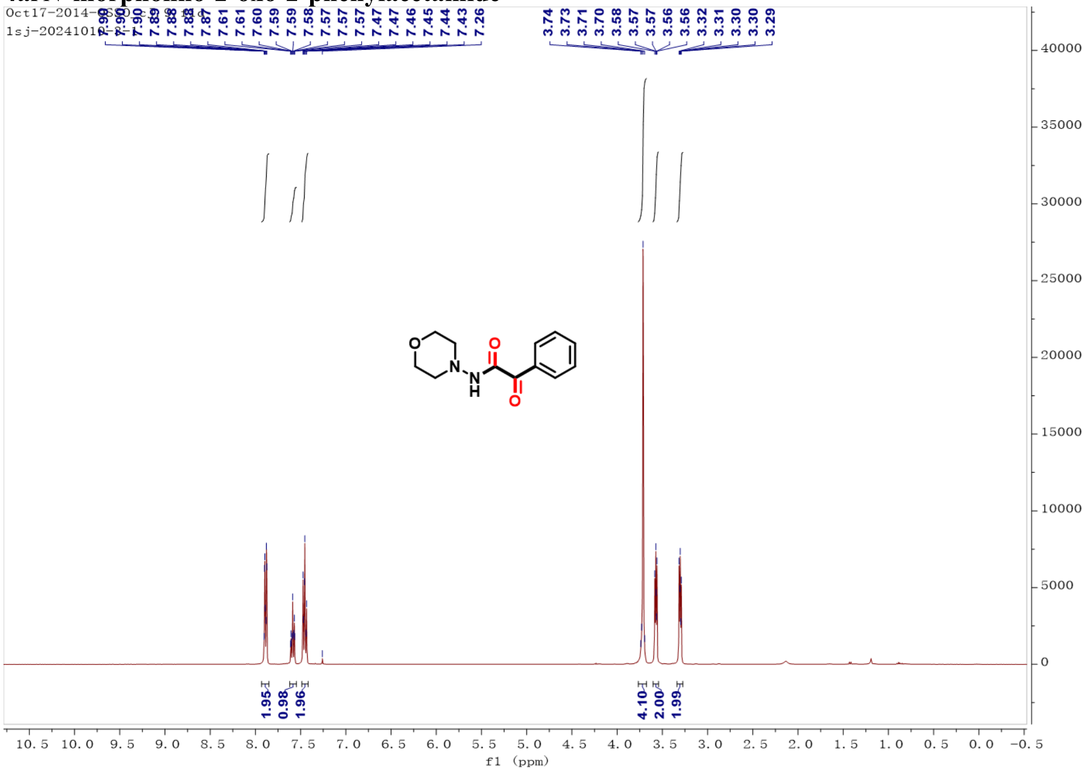

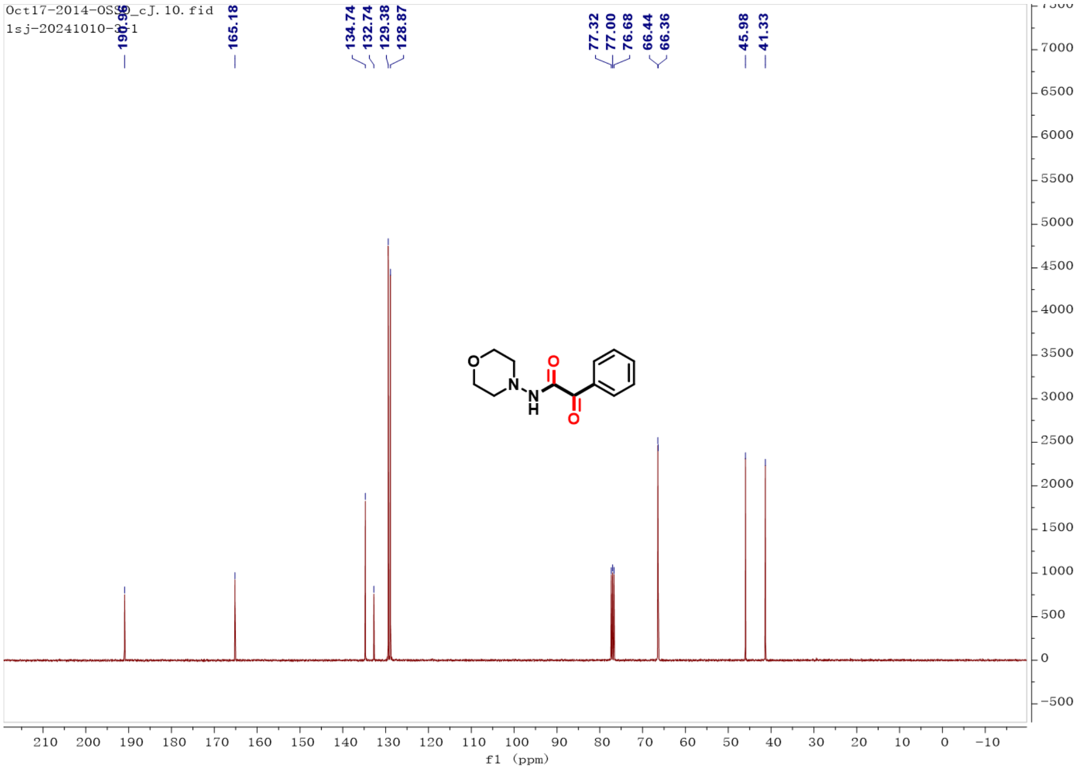

4am *N*-(2-(cyclohex-1-en-1-yl)ethyl)-2-oxo-2-phenylacetamide

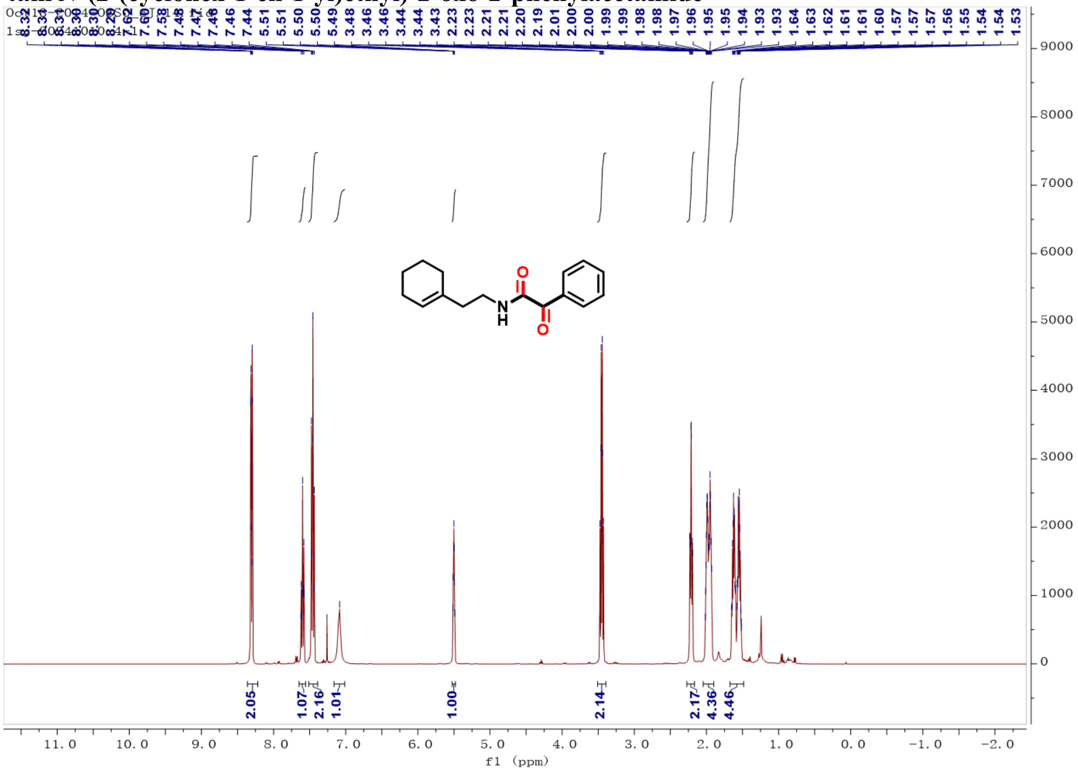

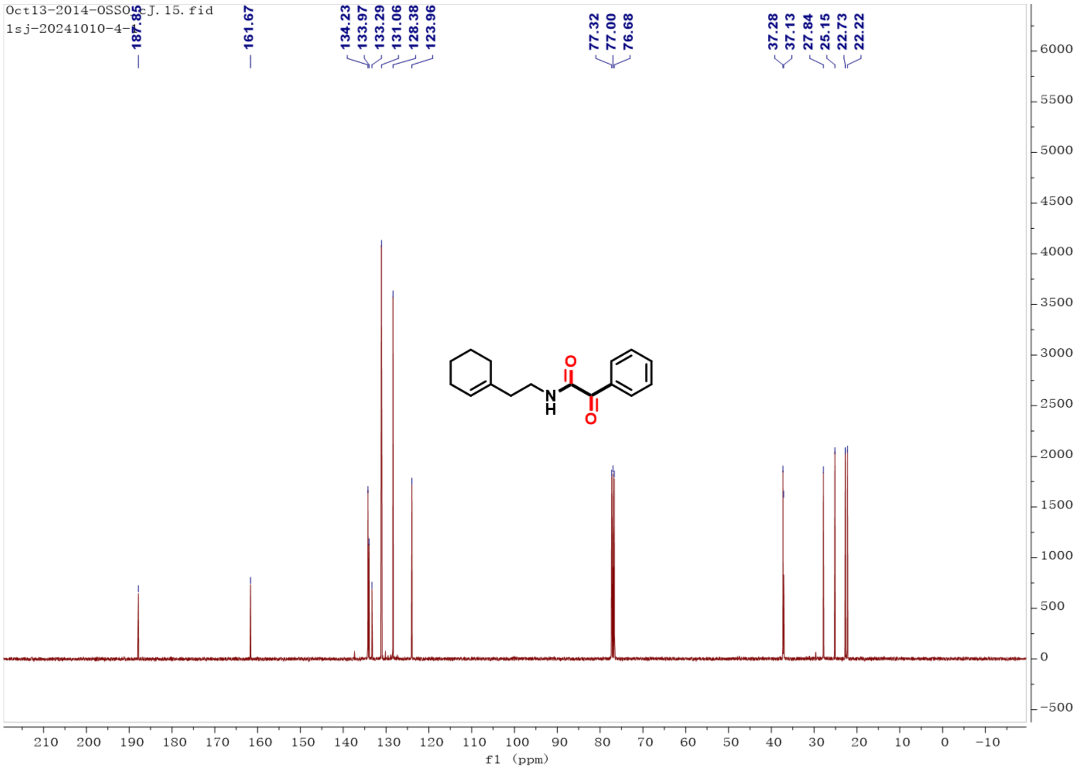

4an 1-morpholino-2-(p-tolyl)ethane-1,2-dione

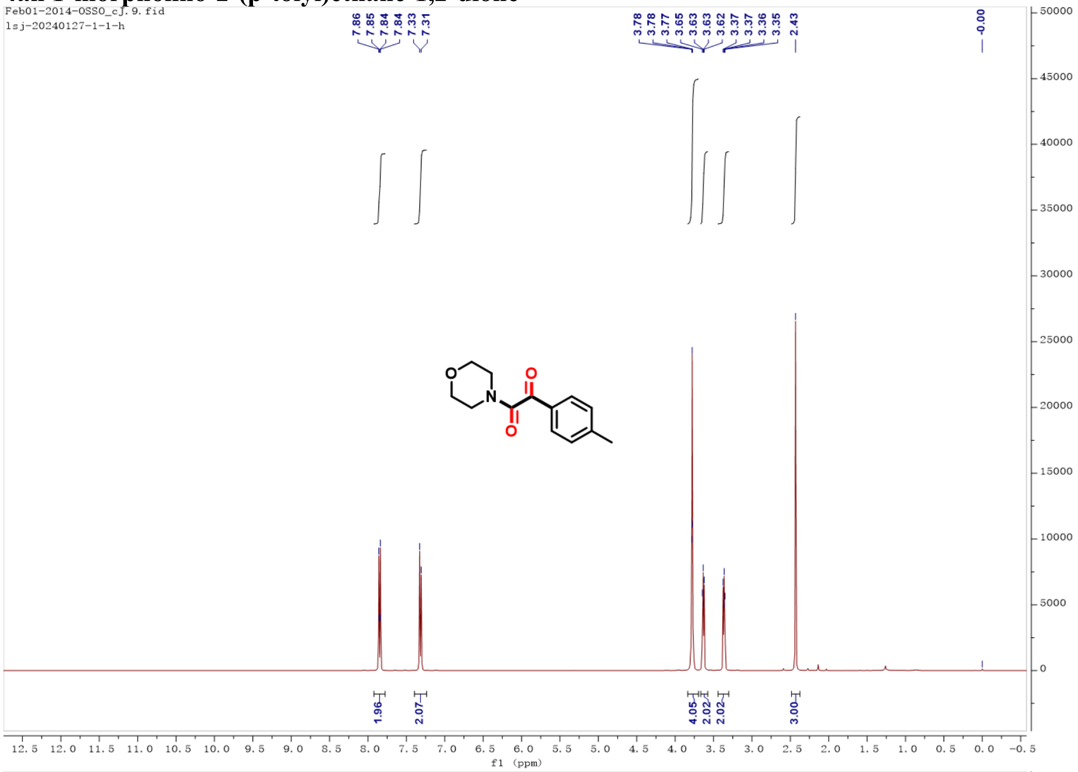

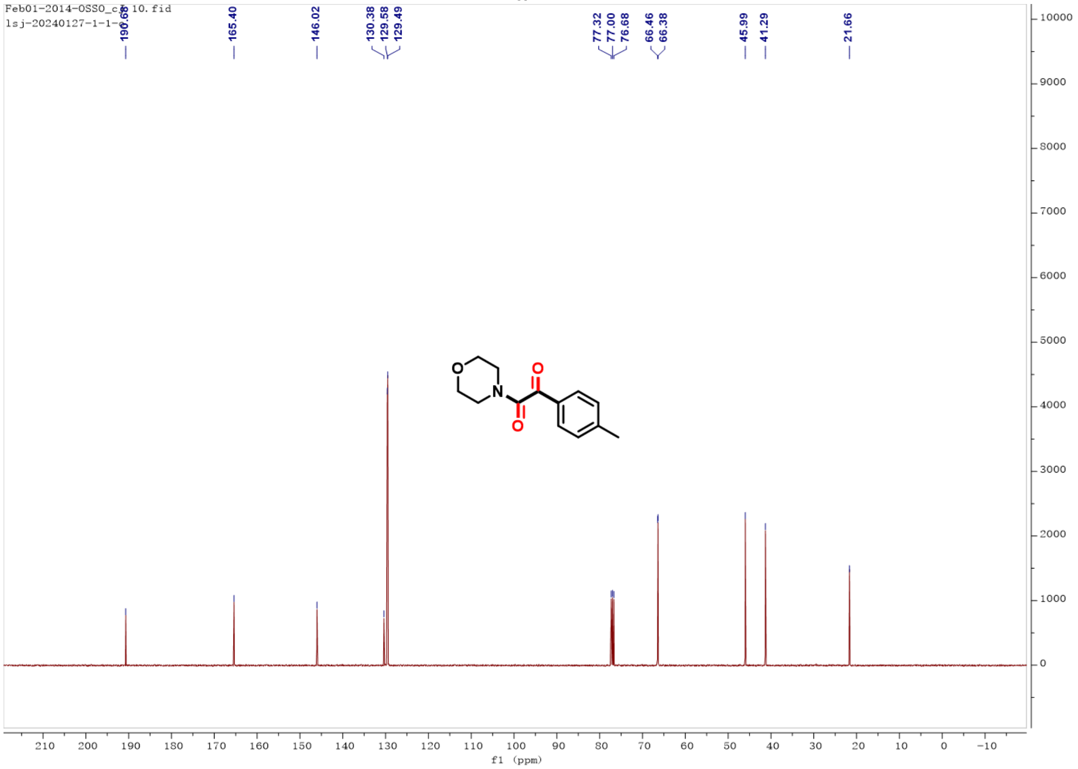

4ao 1-(4-methoxyphenyl)-2-morpholinoethane-1,2-dione

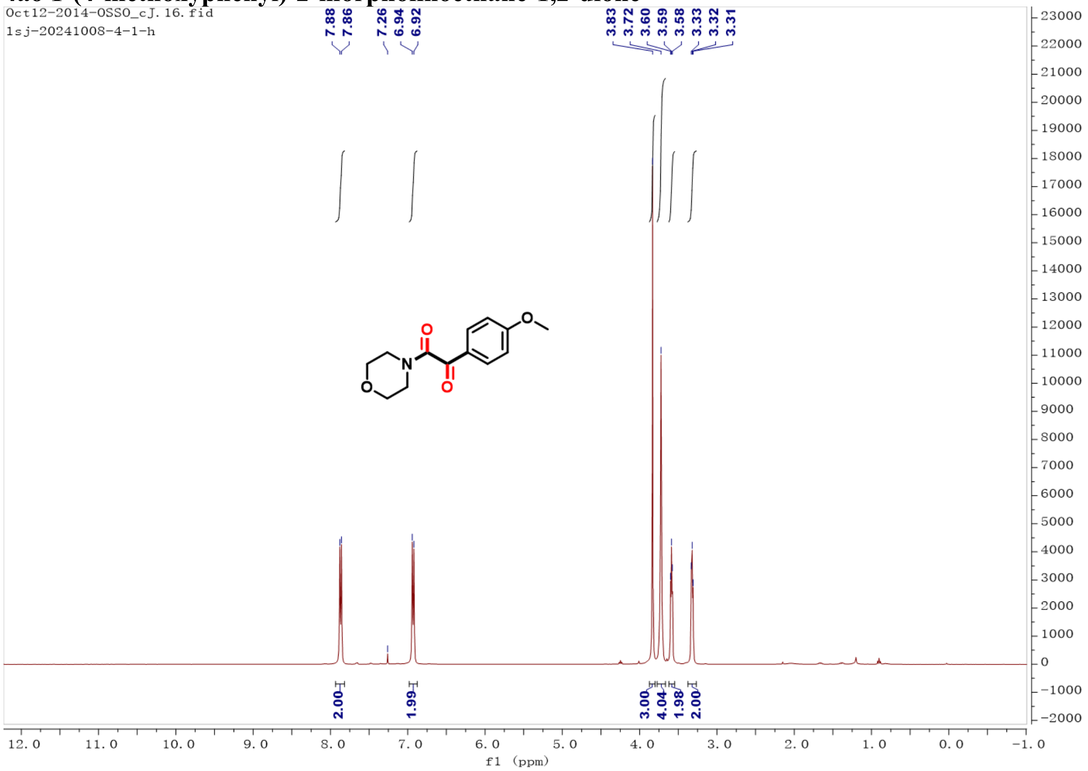

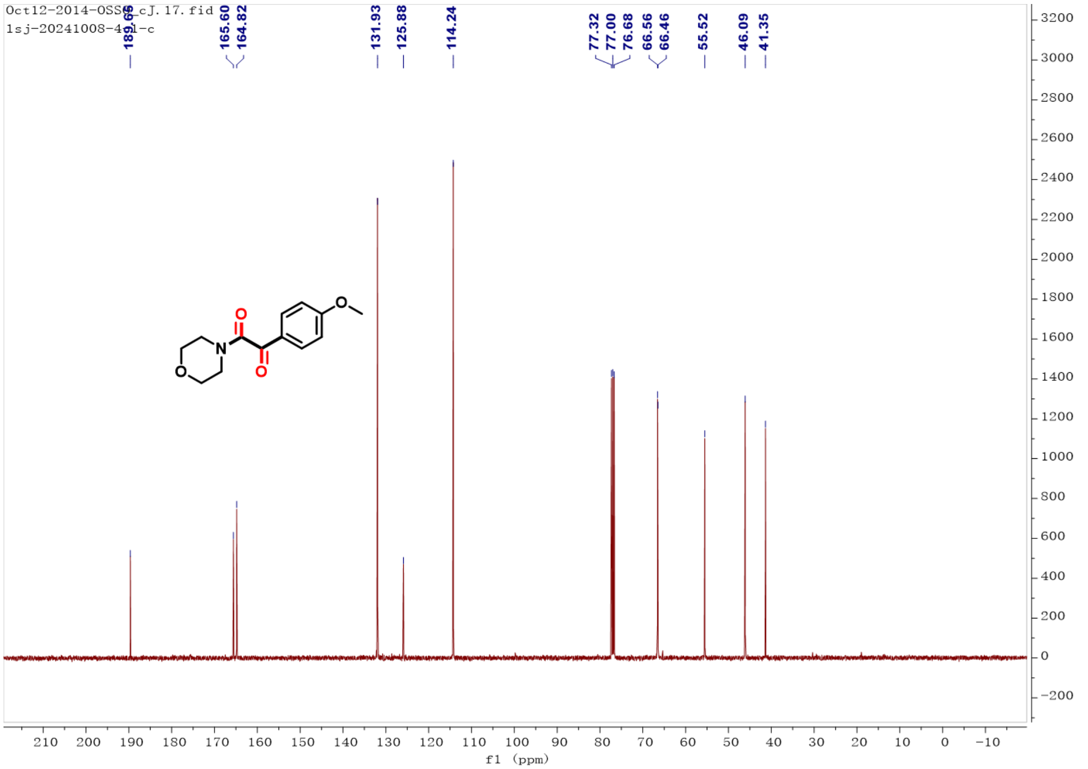

4ap 1-([1,1'-biphenyl]-4-yl)-2-morpholinoethane-1,2-dione

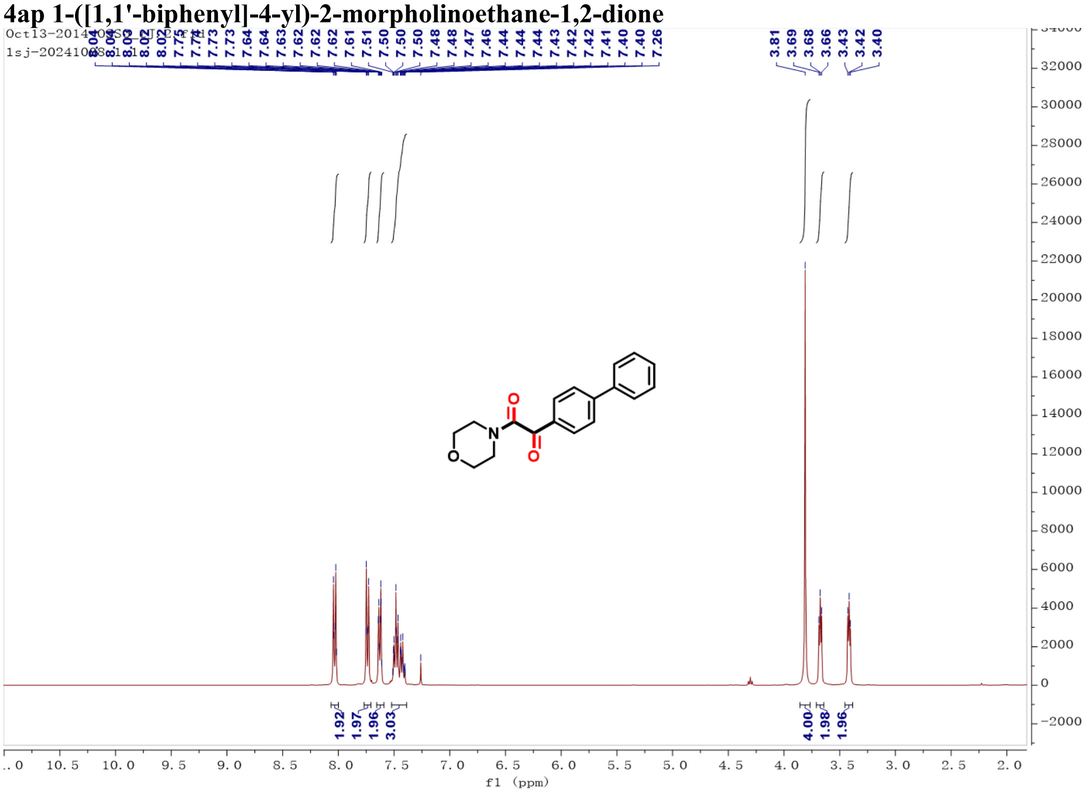

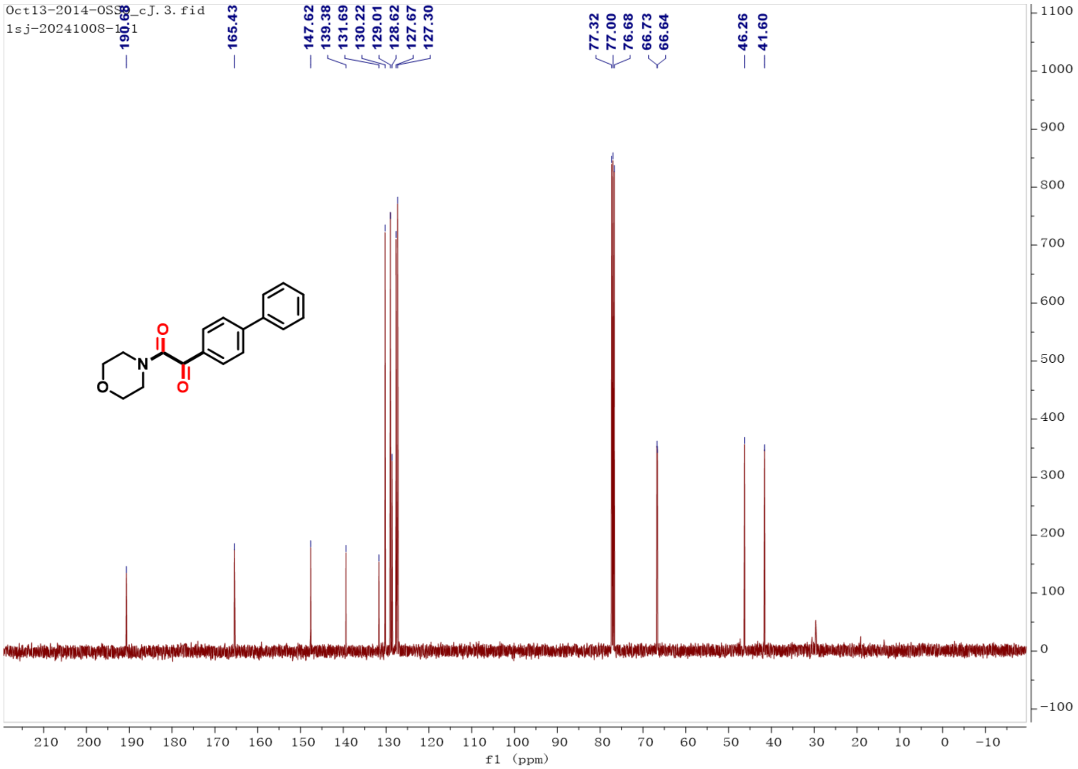

4aq 1-(4-(hydroxymethyl)phenyl)-2-morpholinoethane-1,2-dione

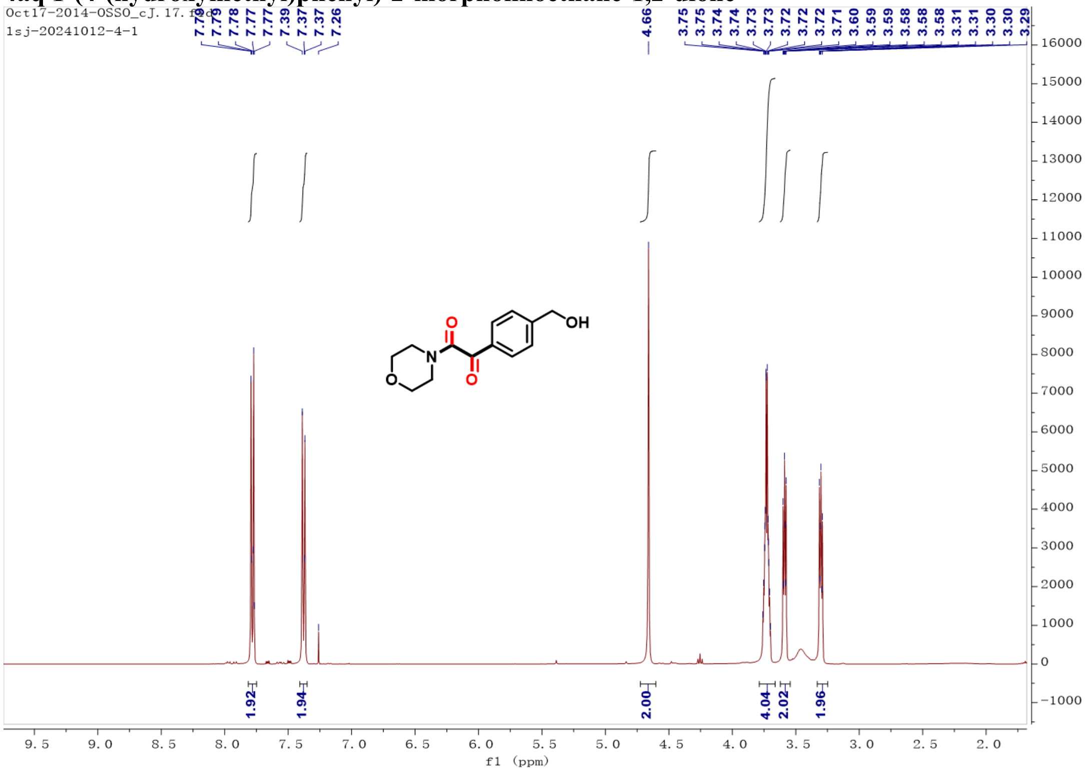

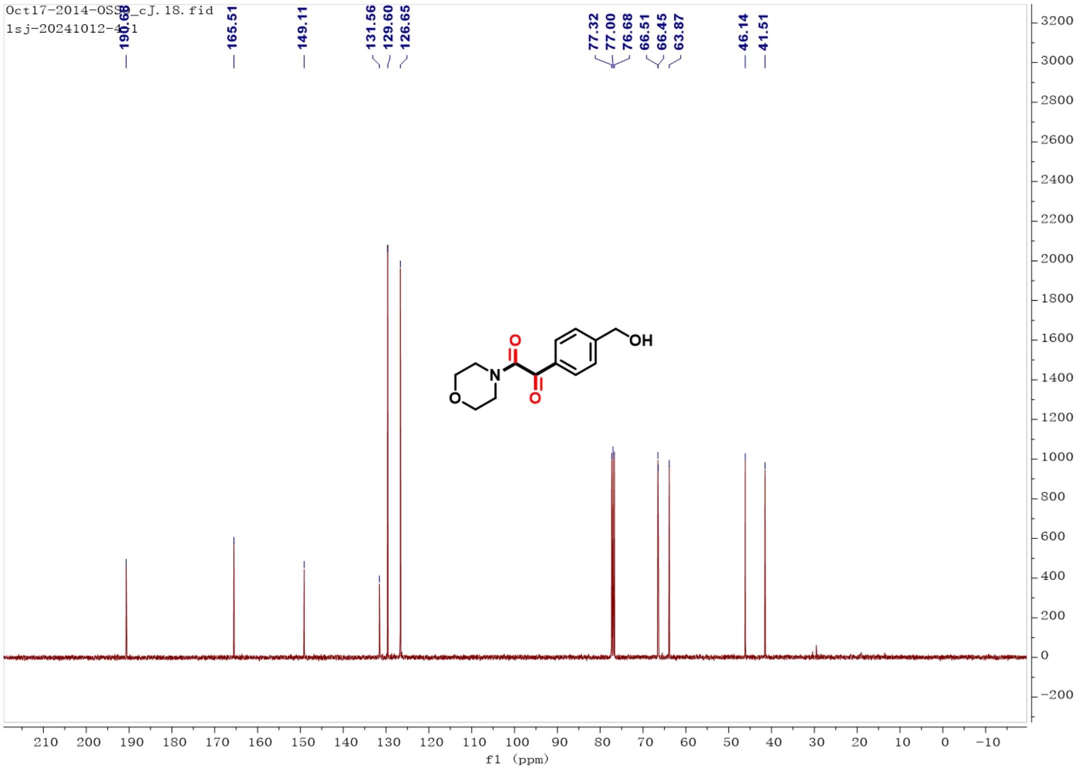

4ar 1-(4-fluorophenyl)-2-morpholinoethane-1,2-dione

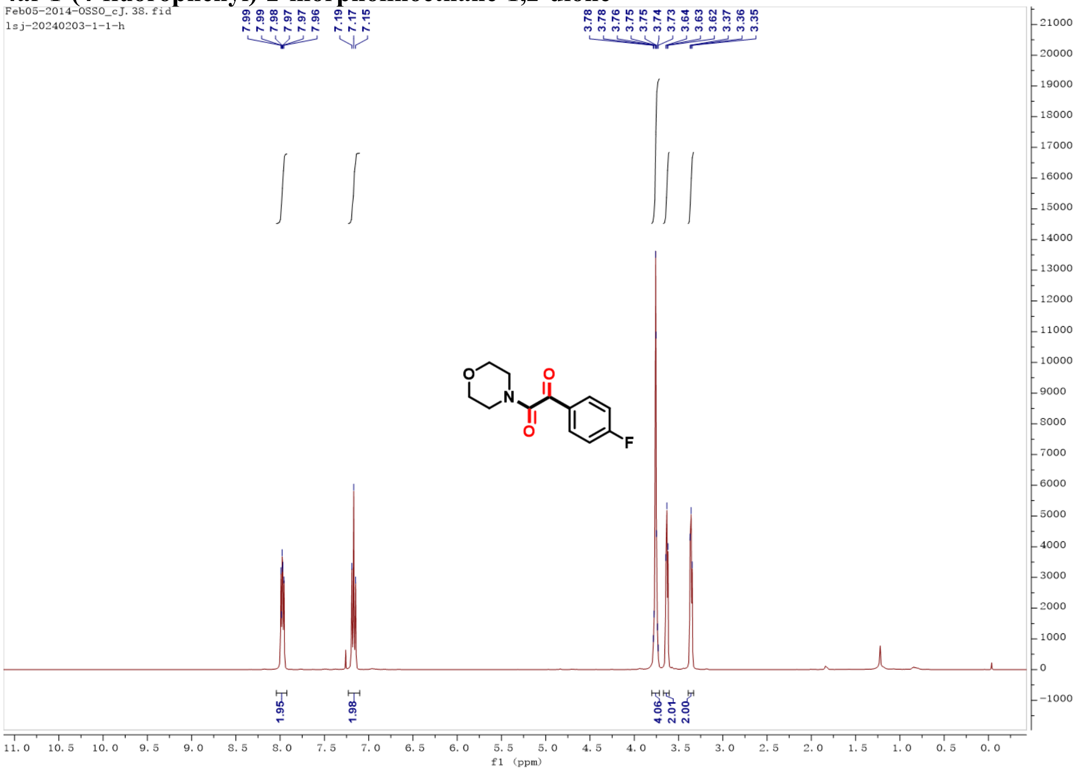

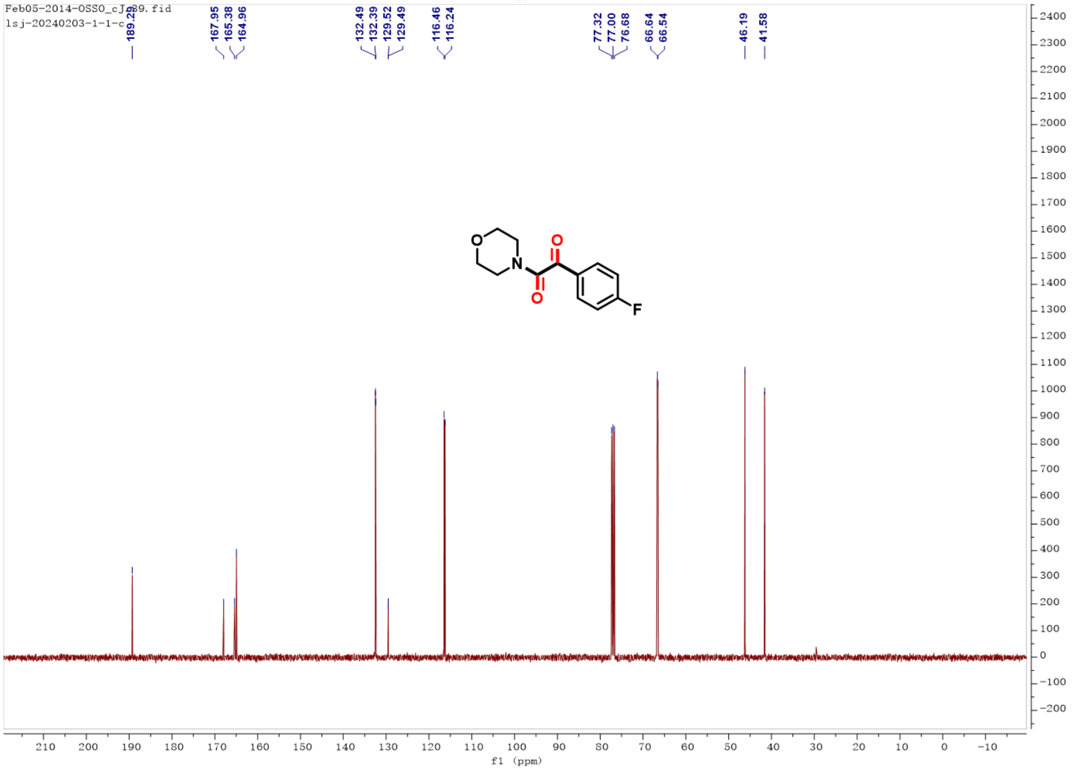

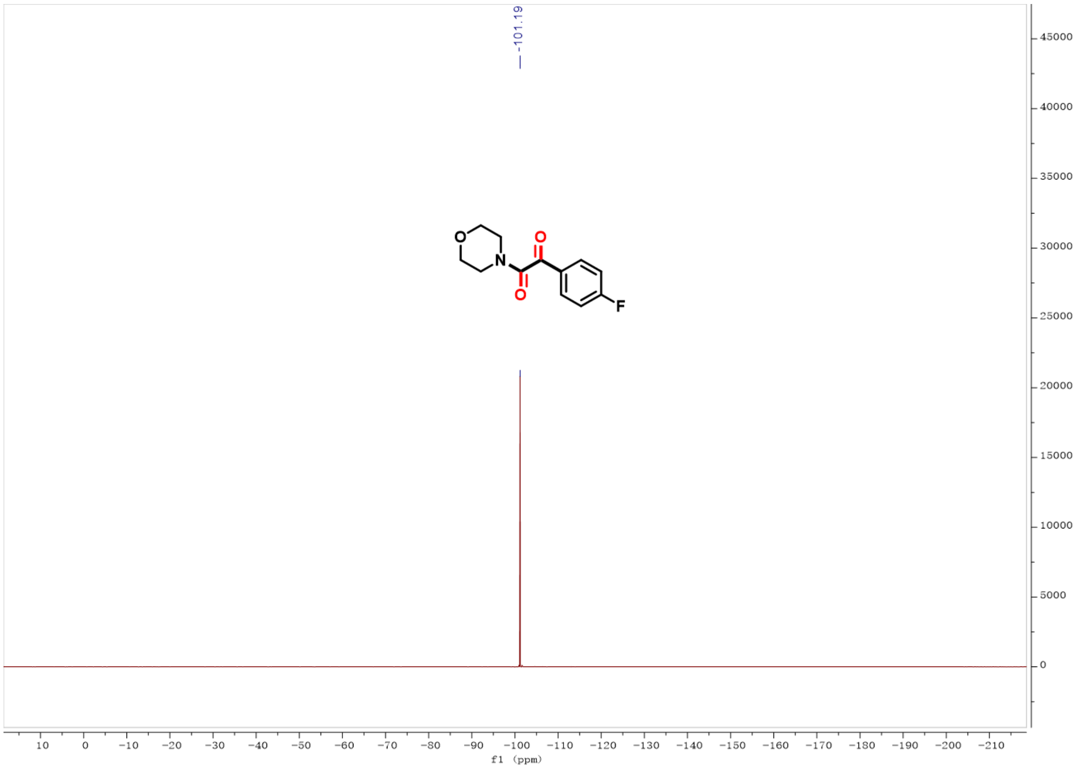

-101.19
O
N
O
O
F
45000
40000
35000
30000
25000
20000
15000
10000
5000
0
10 0 -10 -20 -30 -40 -50 -60 -70 -80 -90 -100 -110 -120 -130 -140 -150 -160 -170 -180 -190 -200 -210
f1 (ppm)

4as 1-(4-chlorophenyl)-2-morpholinoethane-1,2-dione

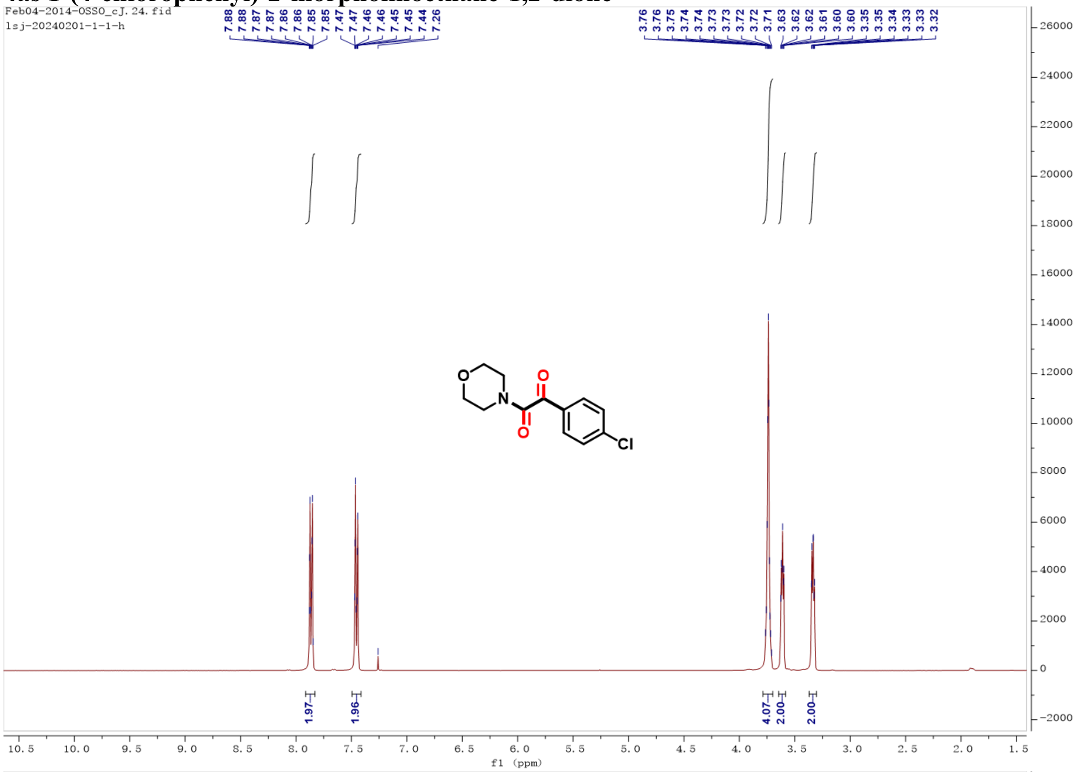

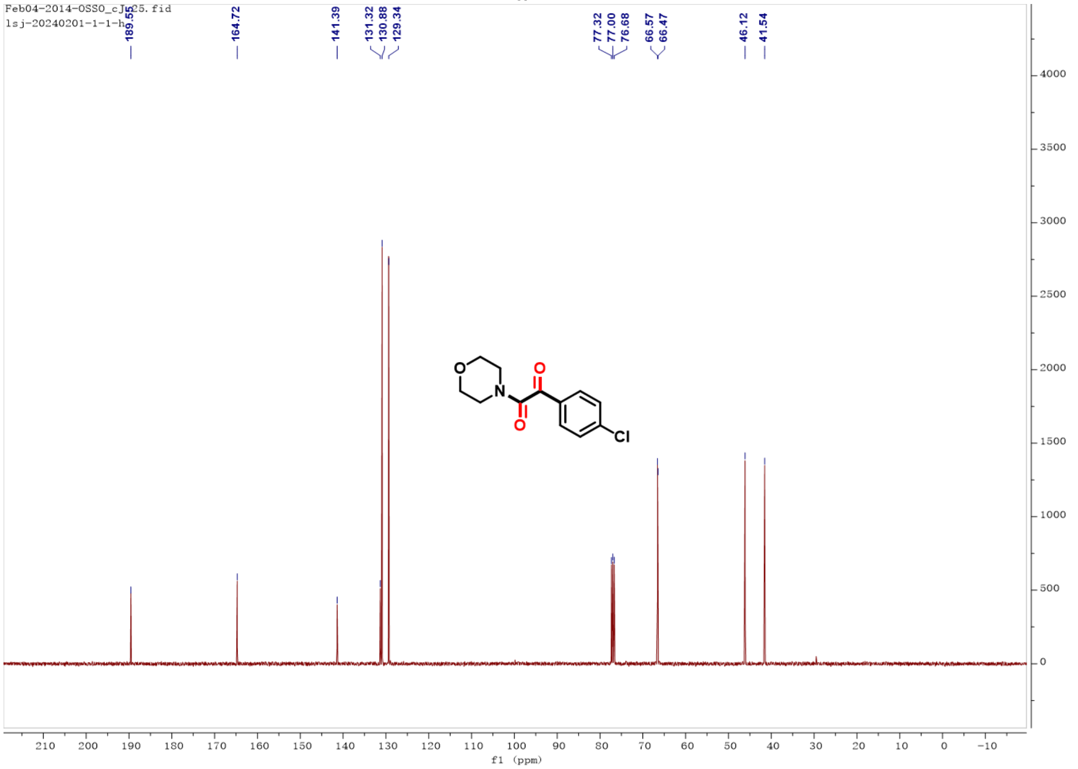

4at 1-(4-bromophenyl)-2-morpholinoethane-1,2-dione

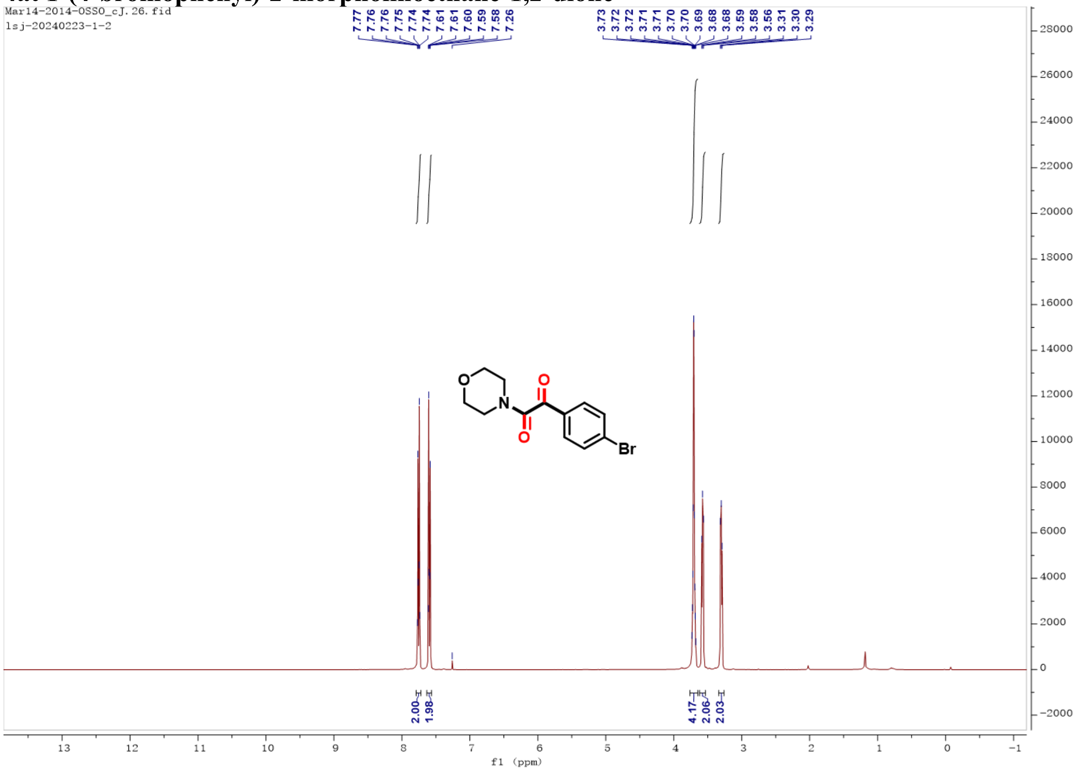

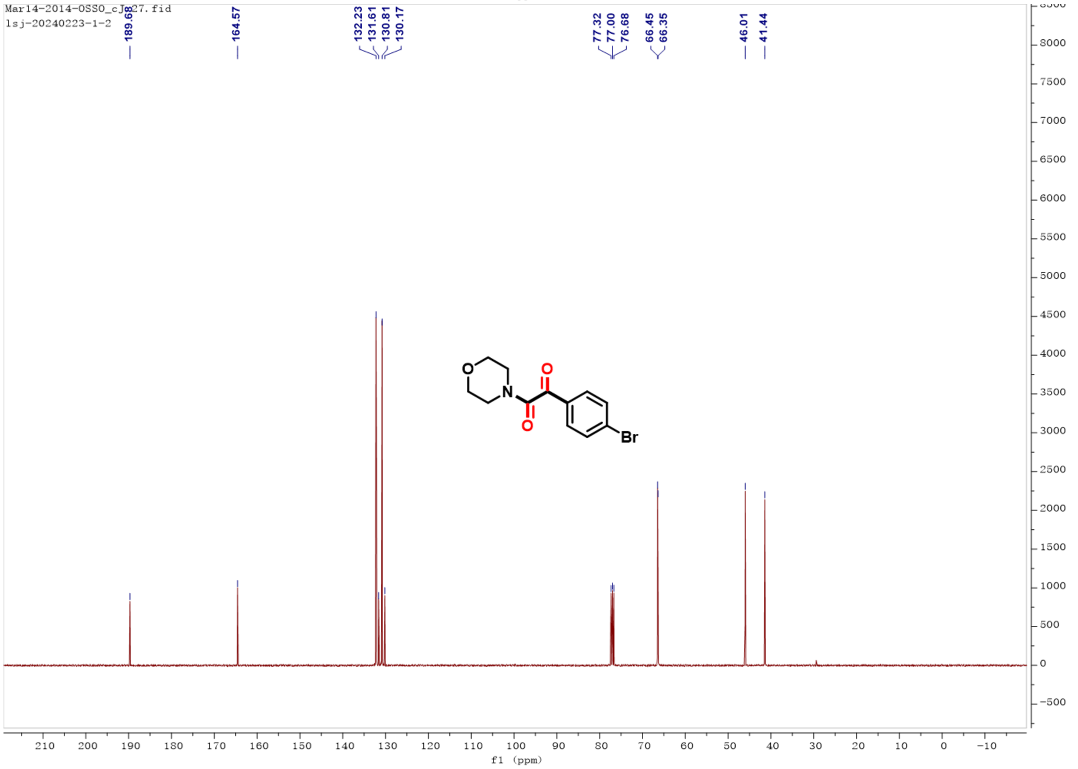

4au 1-(4-isocyanophenyl)-2-morpholinoethane-1,2-dione

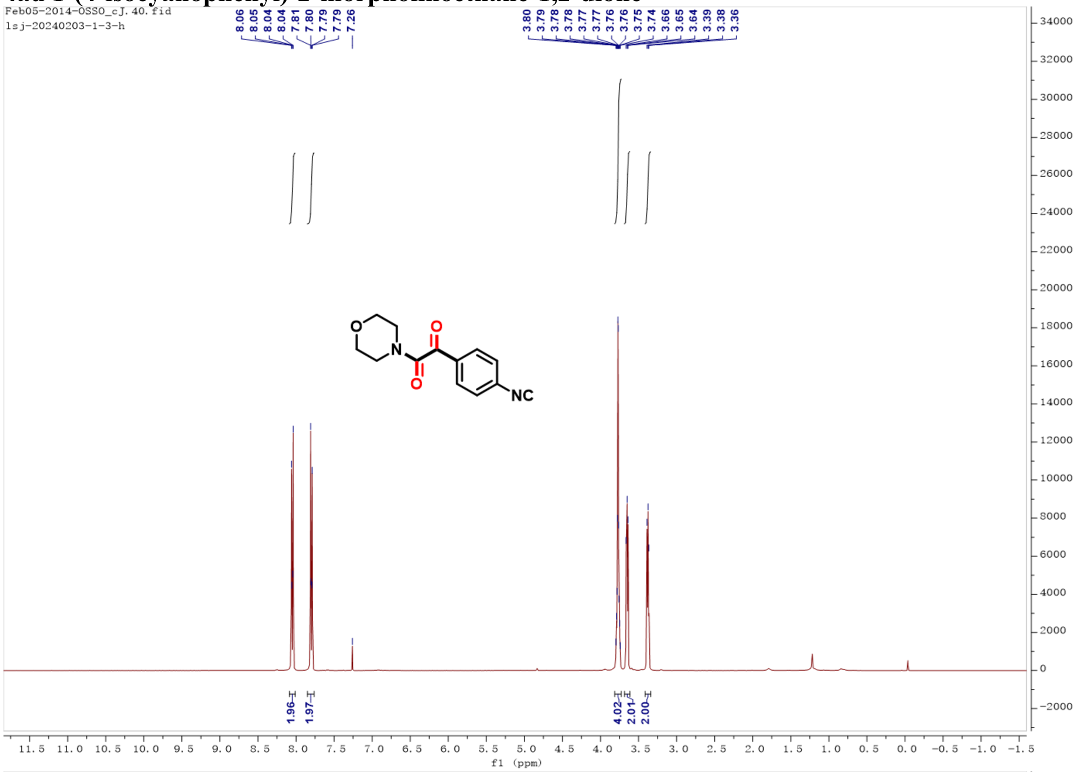

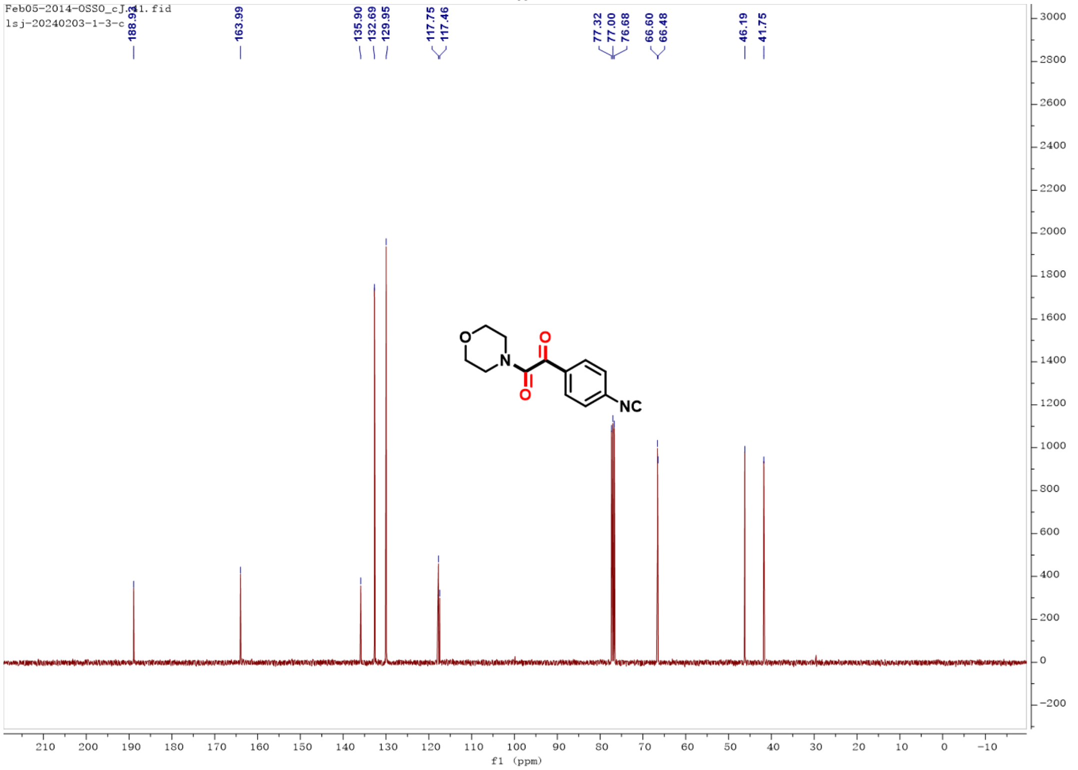

4av 1-morpholino-2-(4-nitrophenyl)ethane-1,2-dione

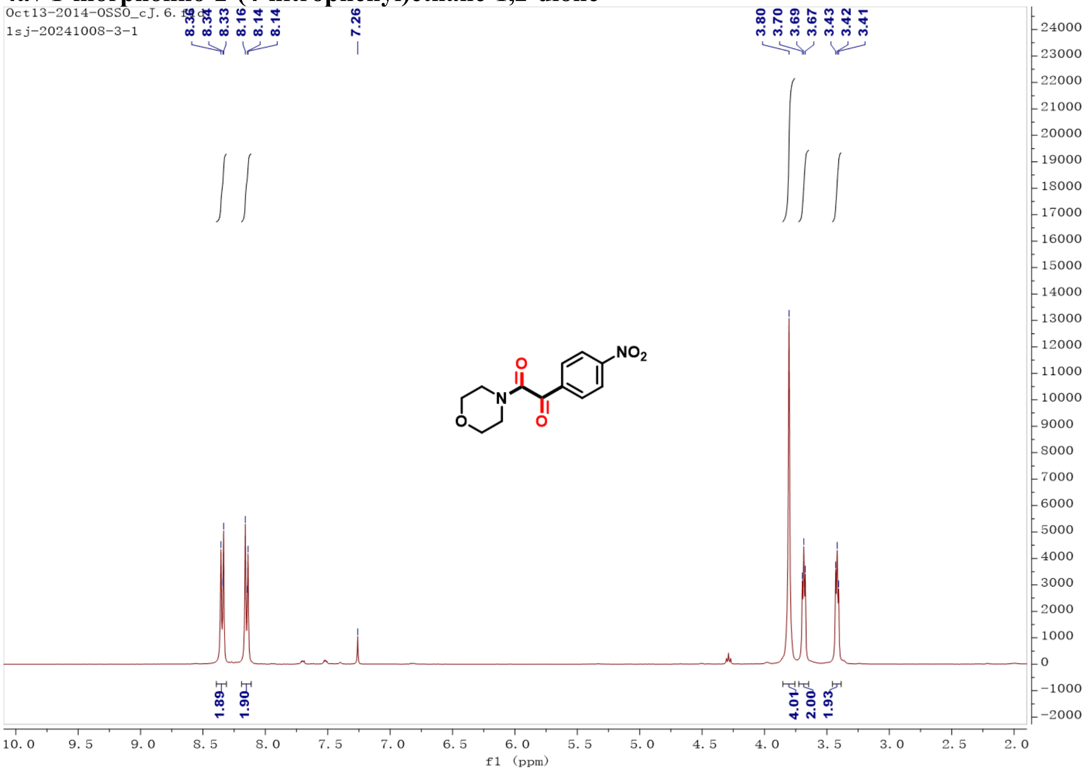

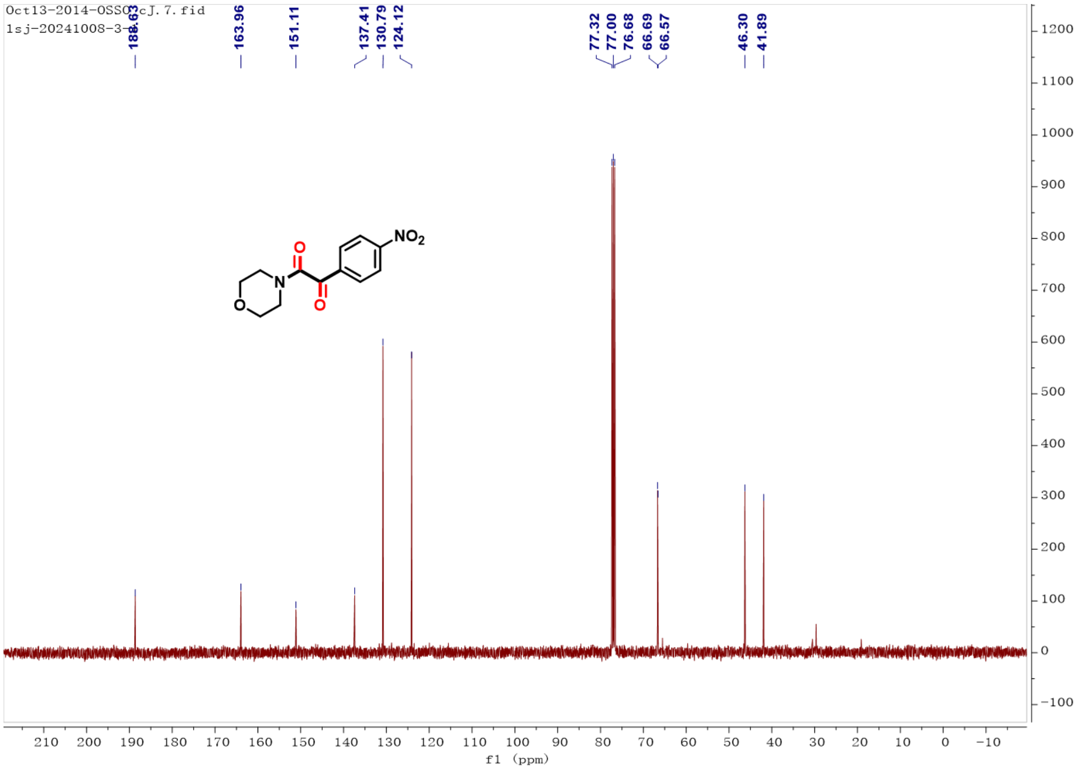

4ax 1-morpholino-2-(4-(prop-1-en-2-yl)phenyl)ethane-1,2-dione

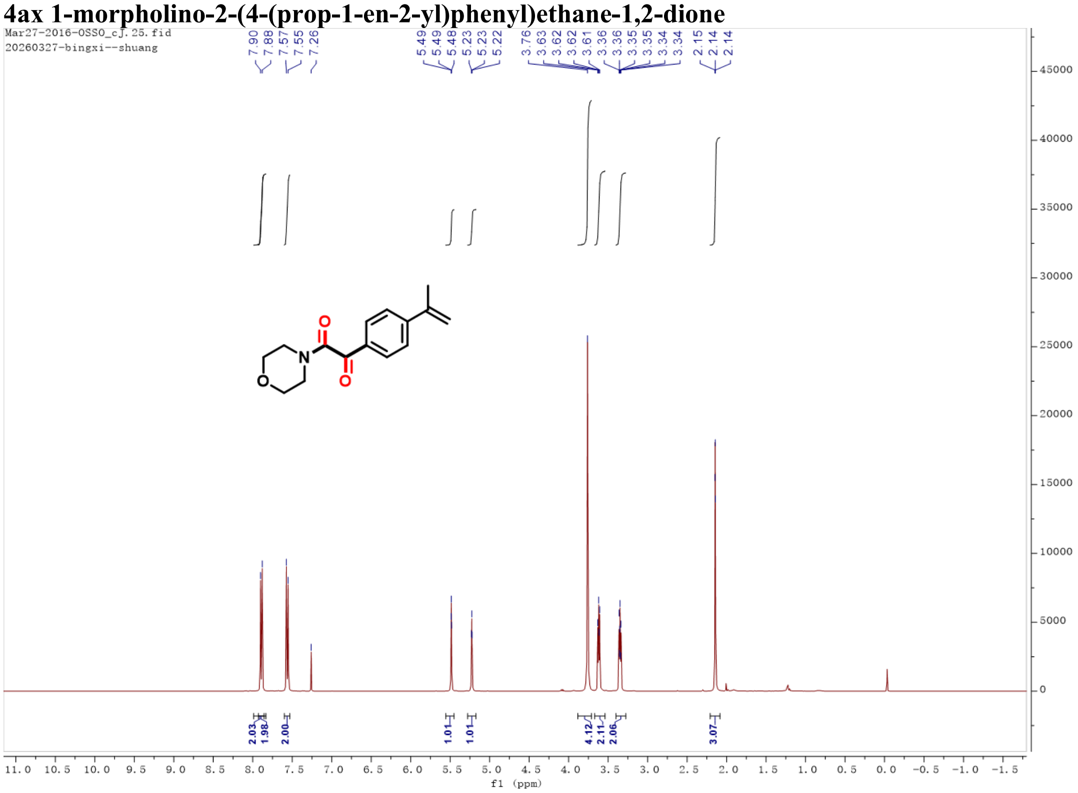

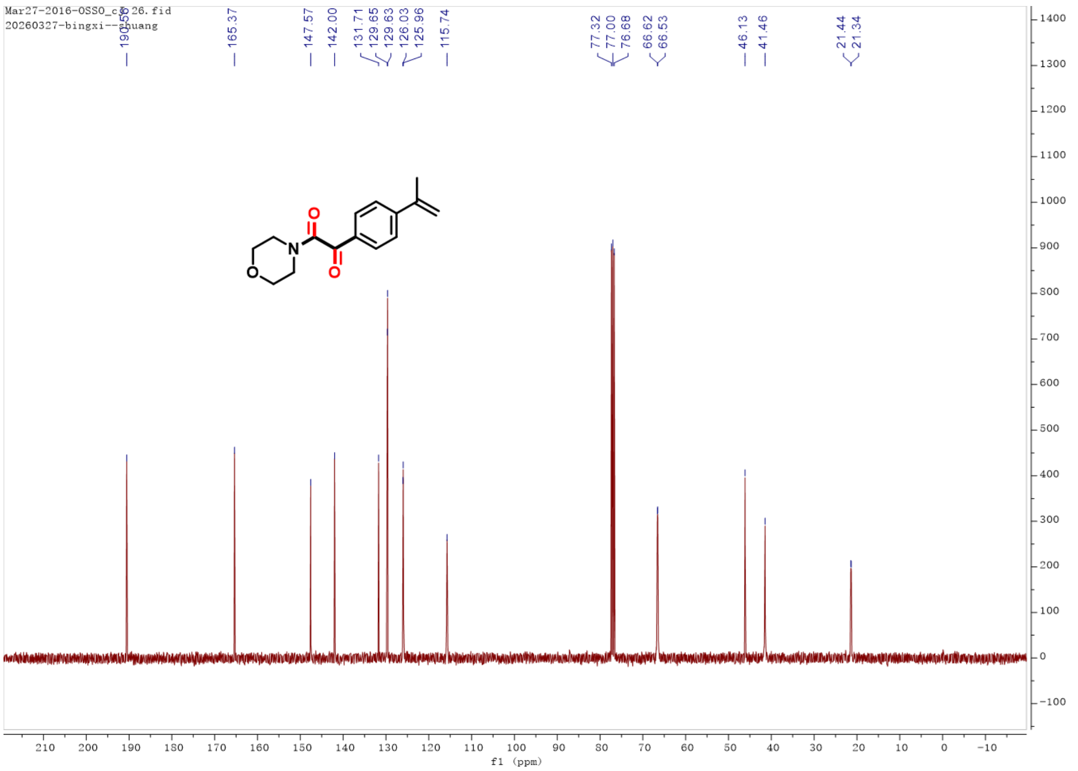

4ay 1-morpholino-2-(m-tolyl)ethane-1,2-dione

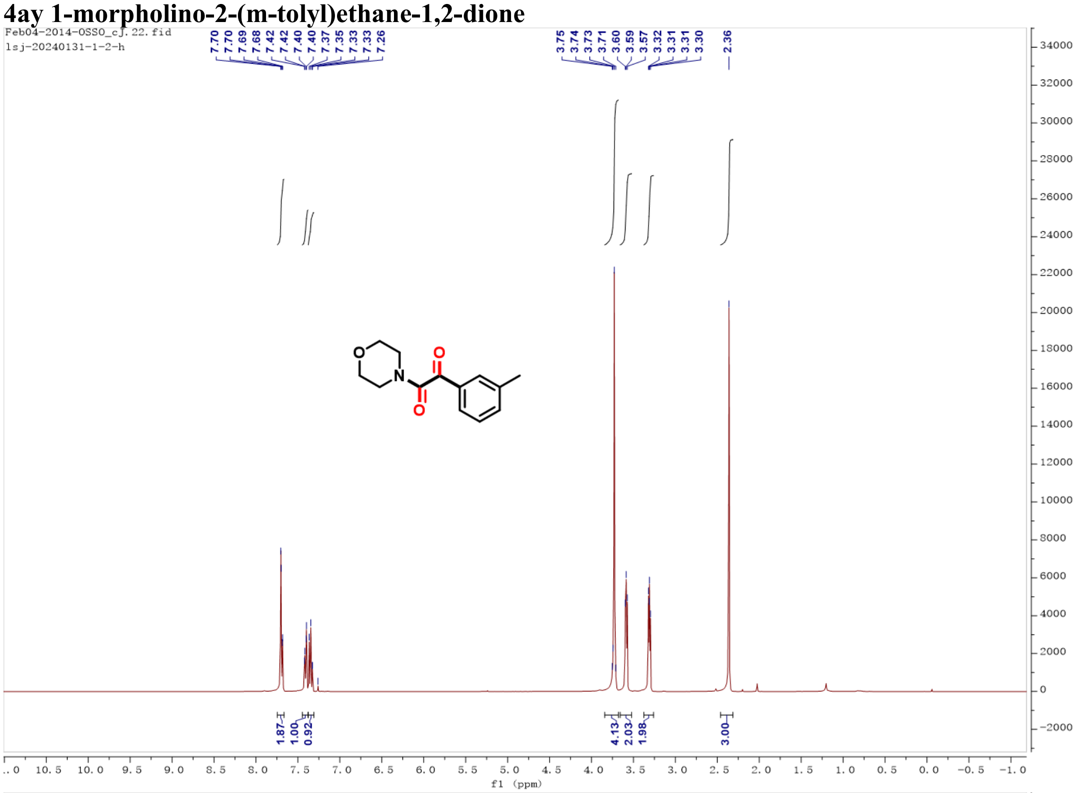

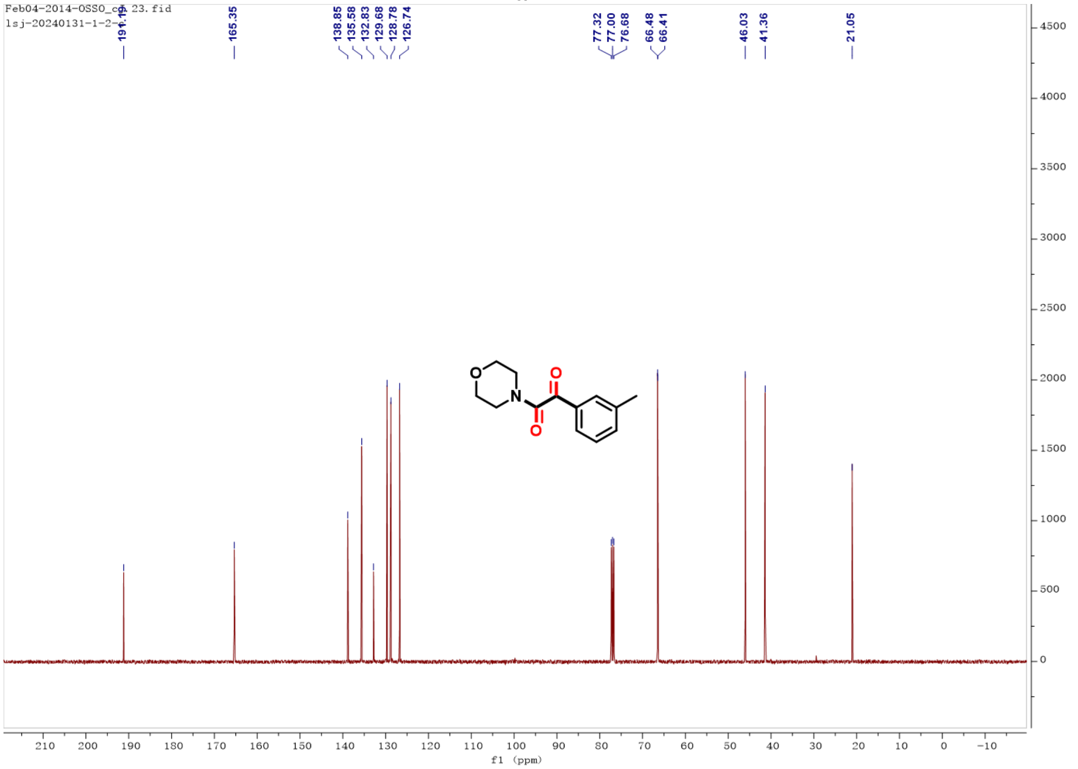

4az 1-(3-aminophenyl)-2-morpholinoethane-1,2-dione

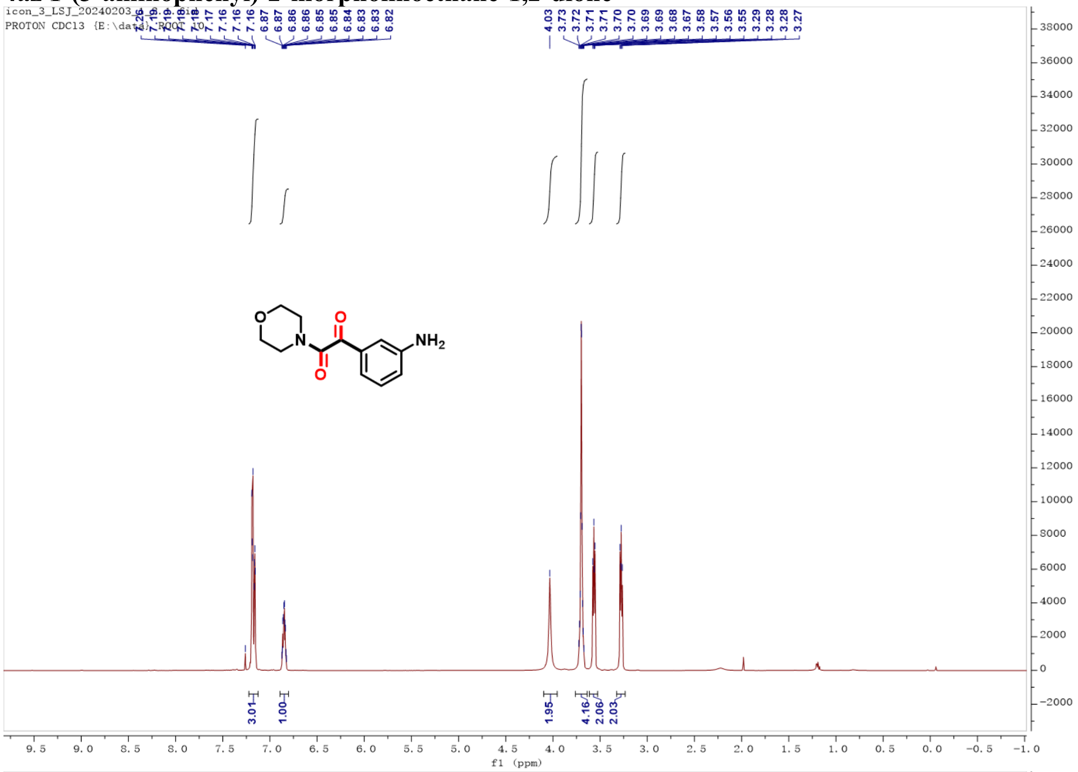

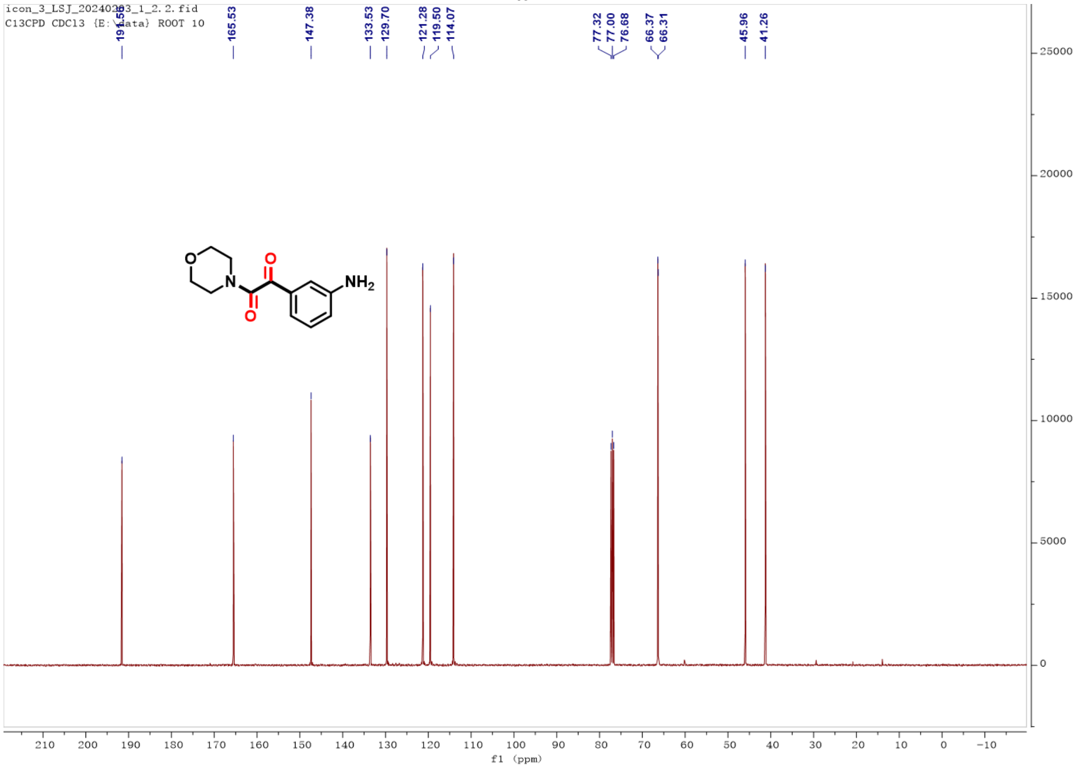

4ba 1-(3-chlorophenyl)-2-morpholinoethane-1,2-dione

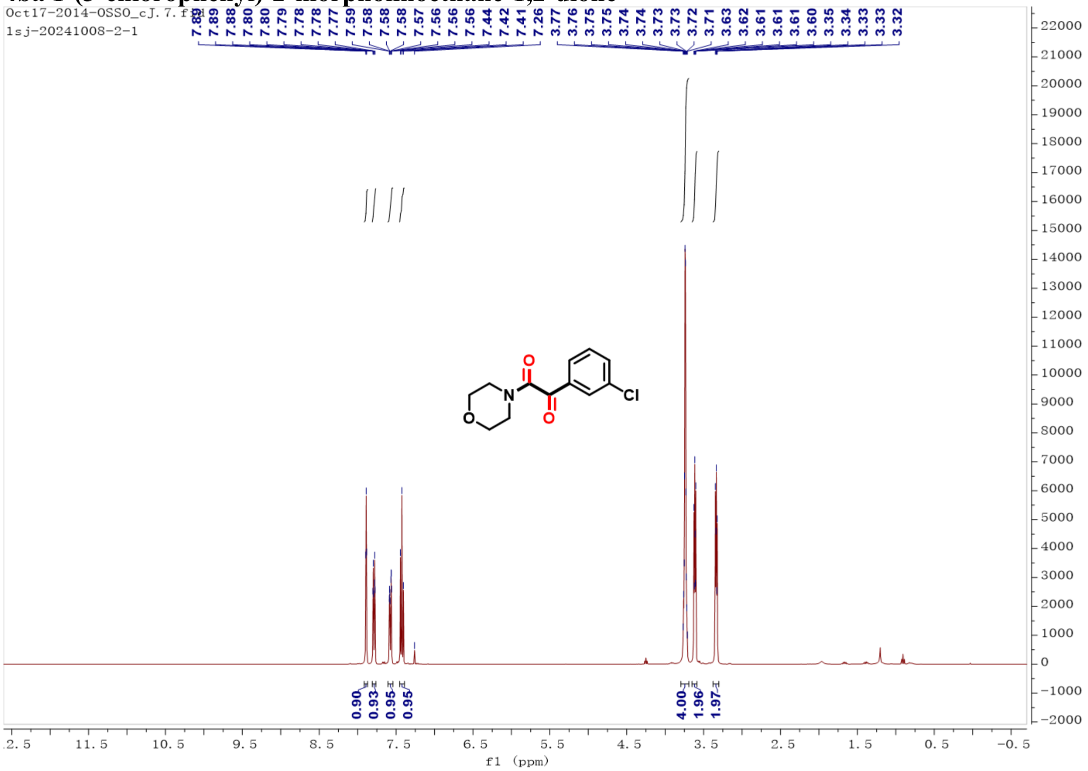

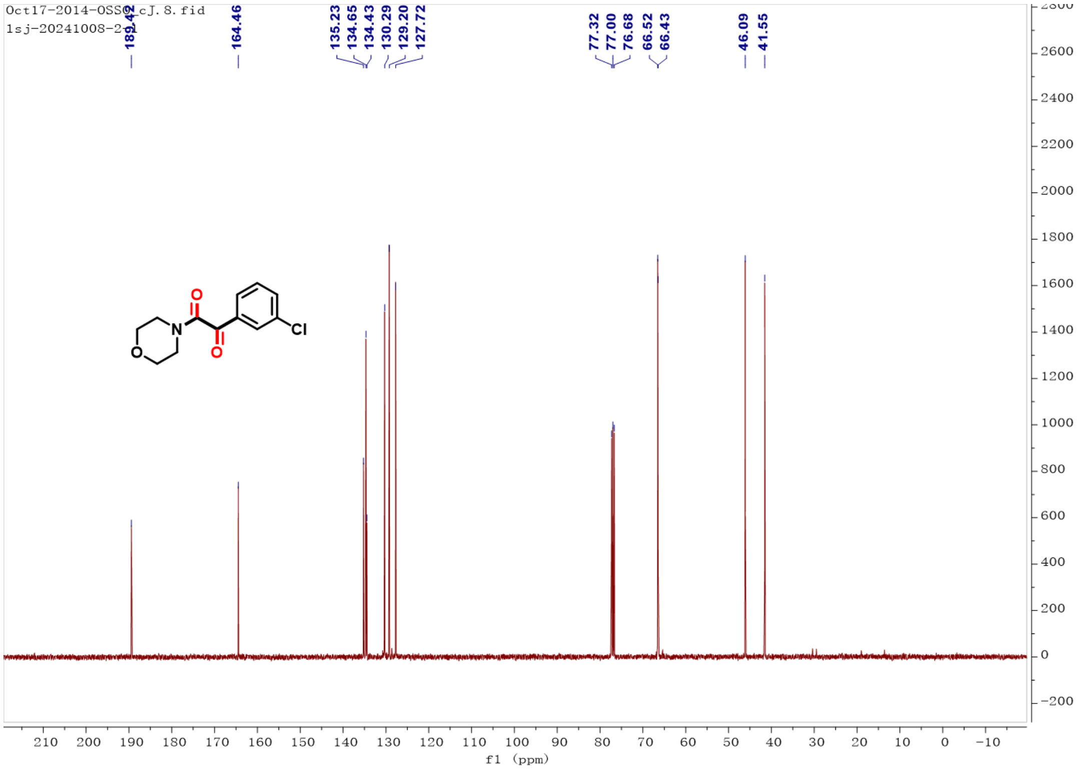

4bb 1-morpholino-2-(pyridin-3-yl)ethane-1,2-dione

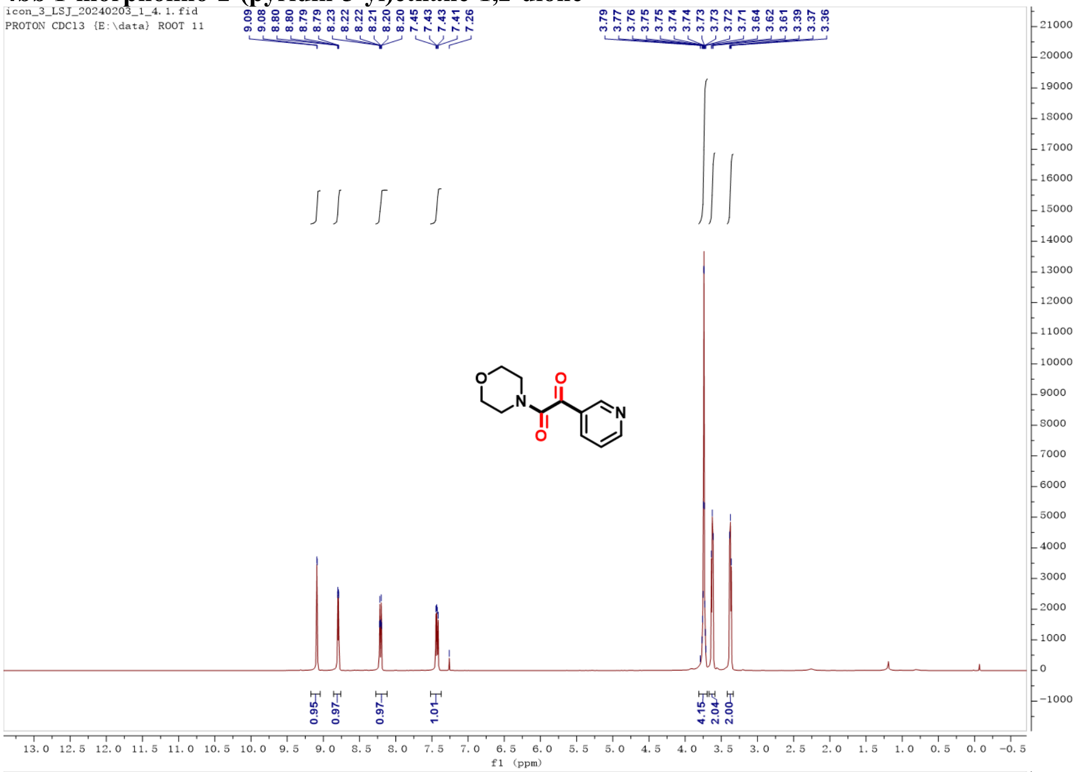

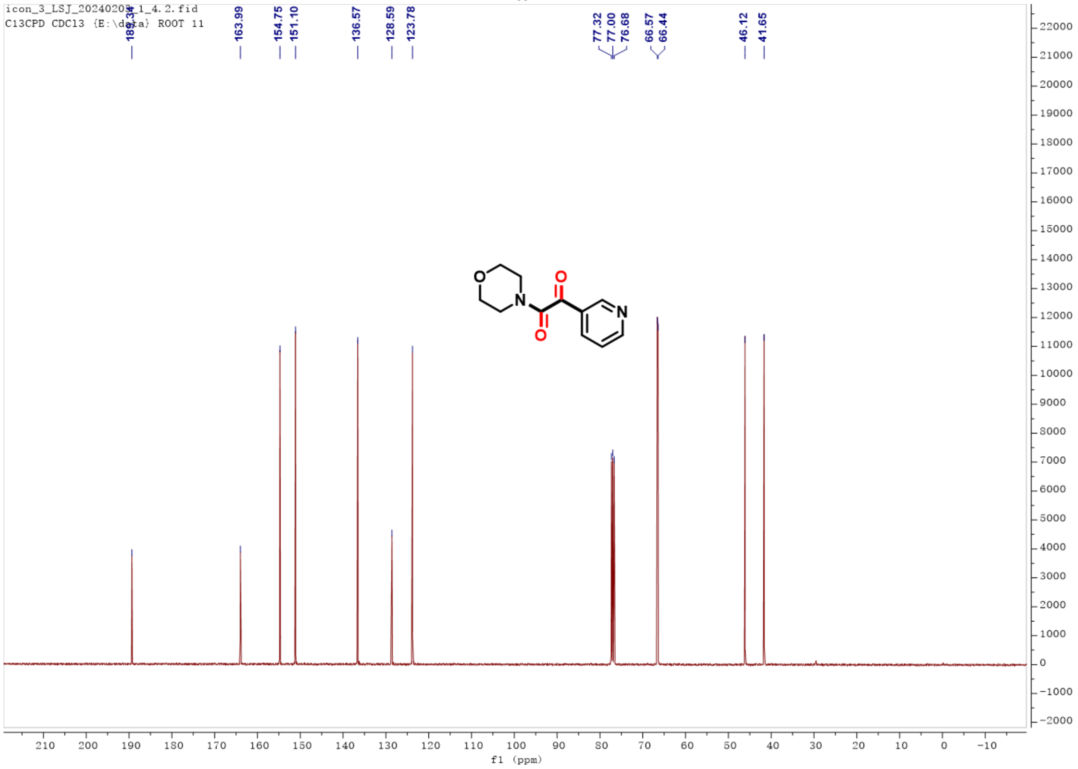

4bc 1-(2,3-dihydrobenzo[*b*][1,4]dioxin-6-yl)-2-morpholinoethane-1,2-dione

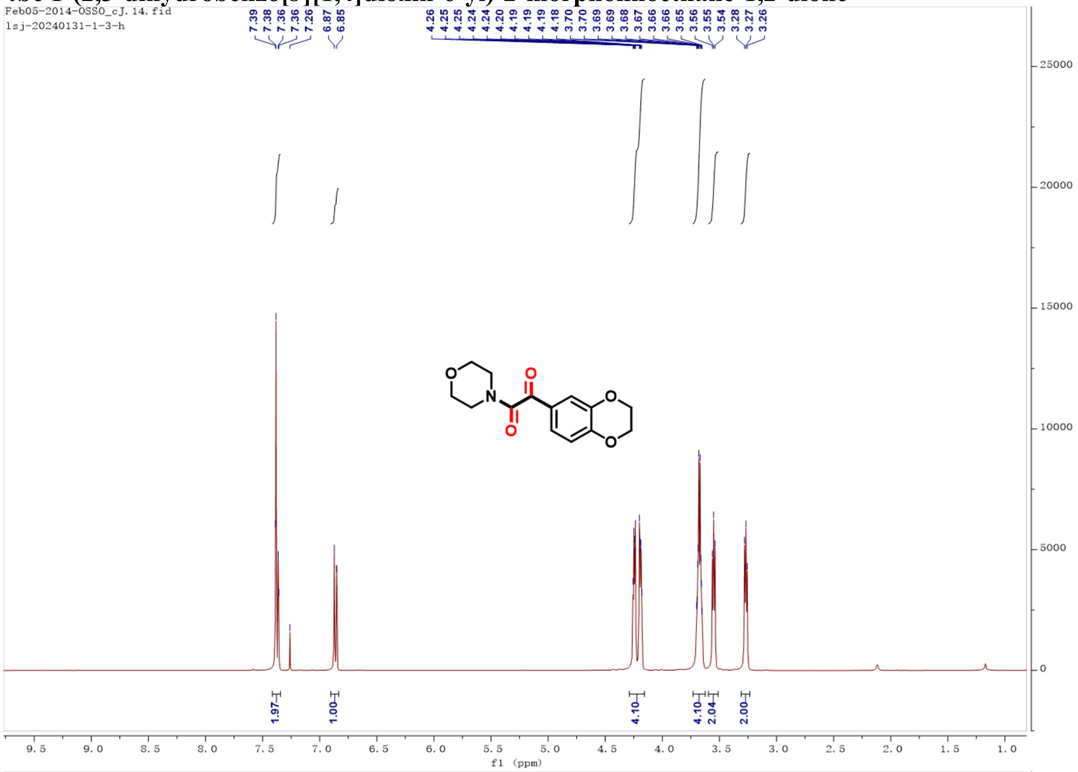

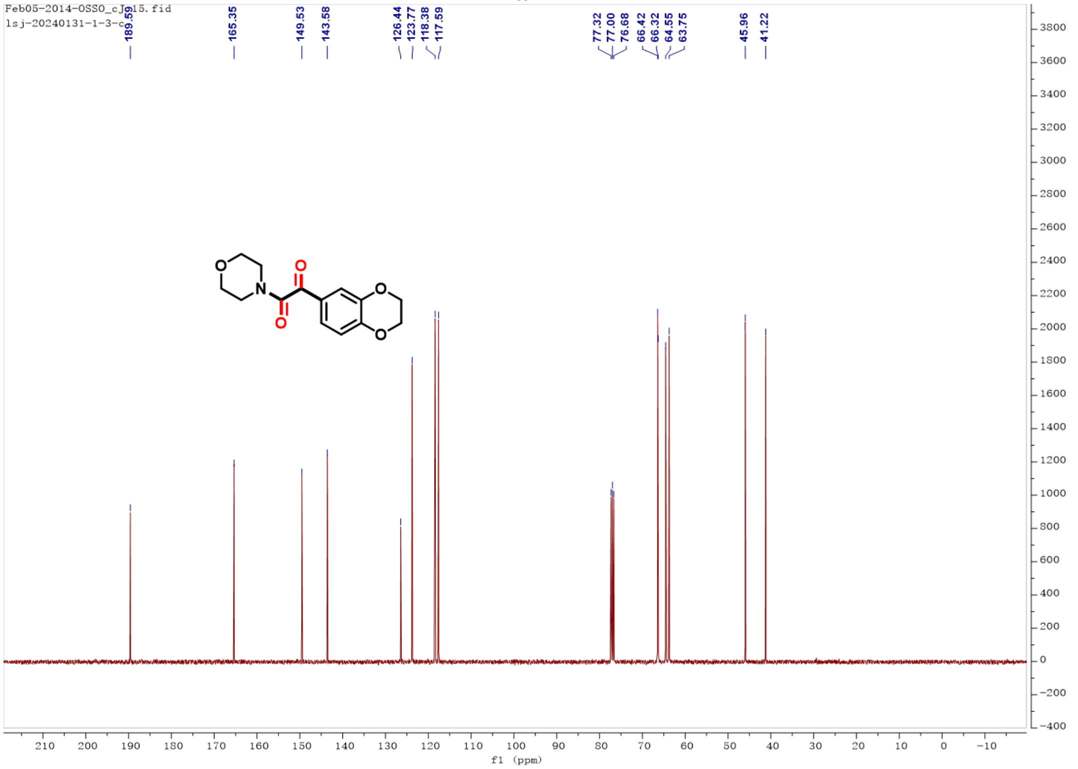

4bd 1-morpholino-2-(naphthalen-2-yl)ethane-1,2-dione

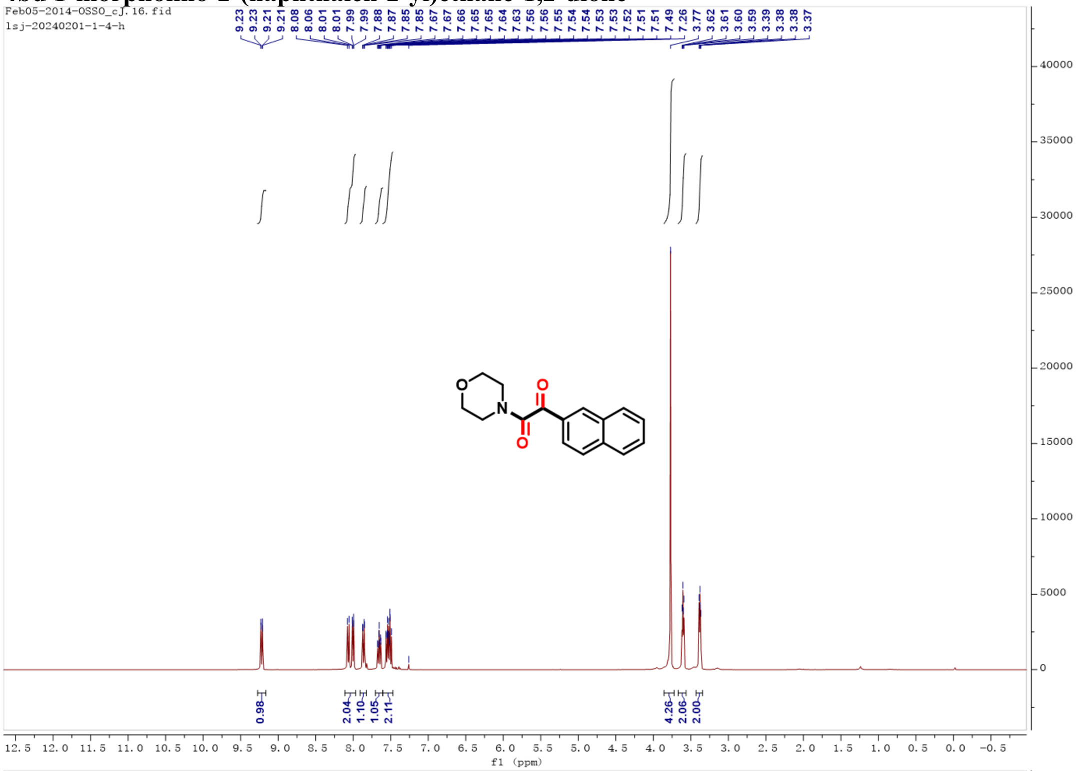

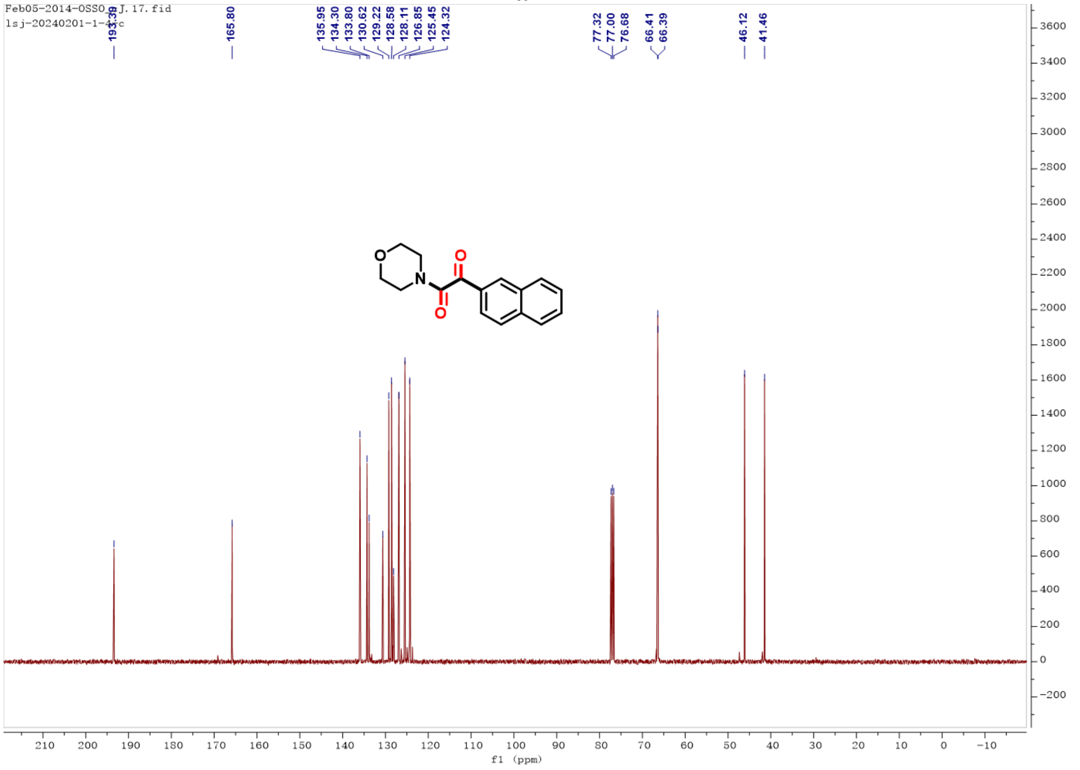

3be *(R)-N*-(1-cyclohexylethyl)benzamide

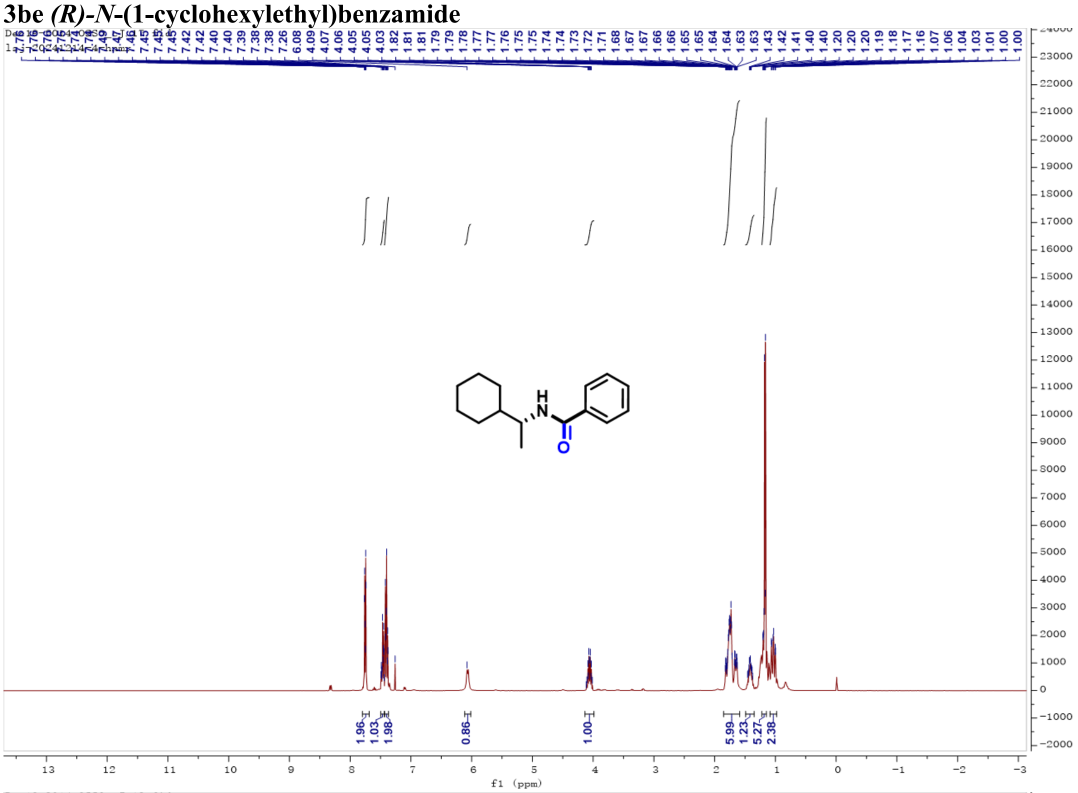

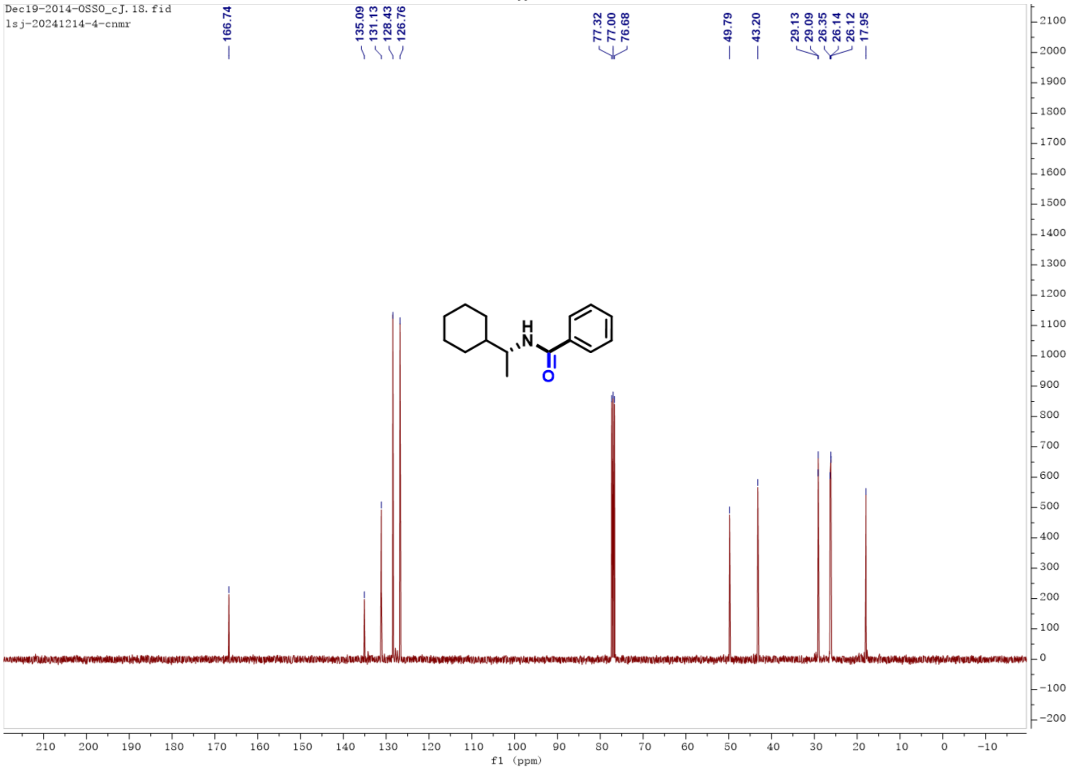

3bf *(R)*-N-methyl-*N*-(1-phenylethyl)benzamide

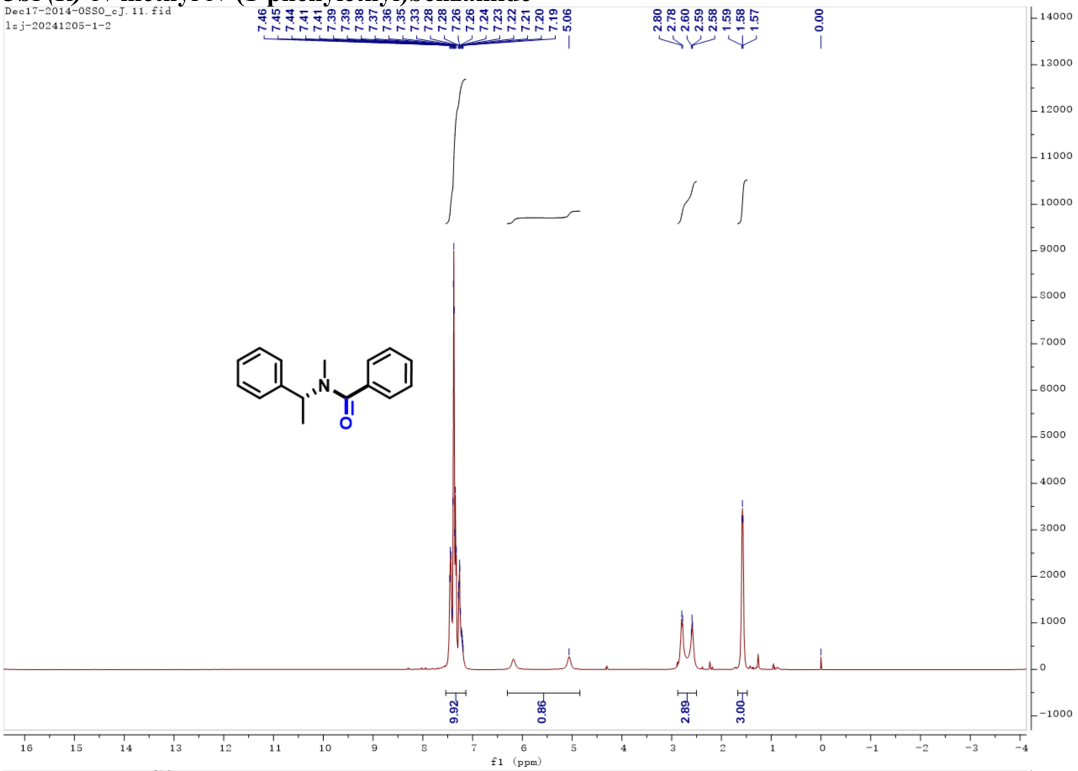

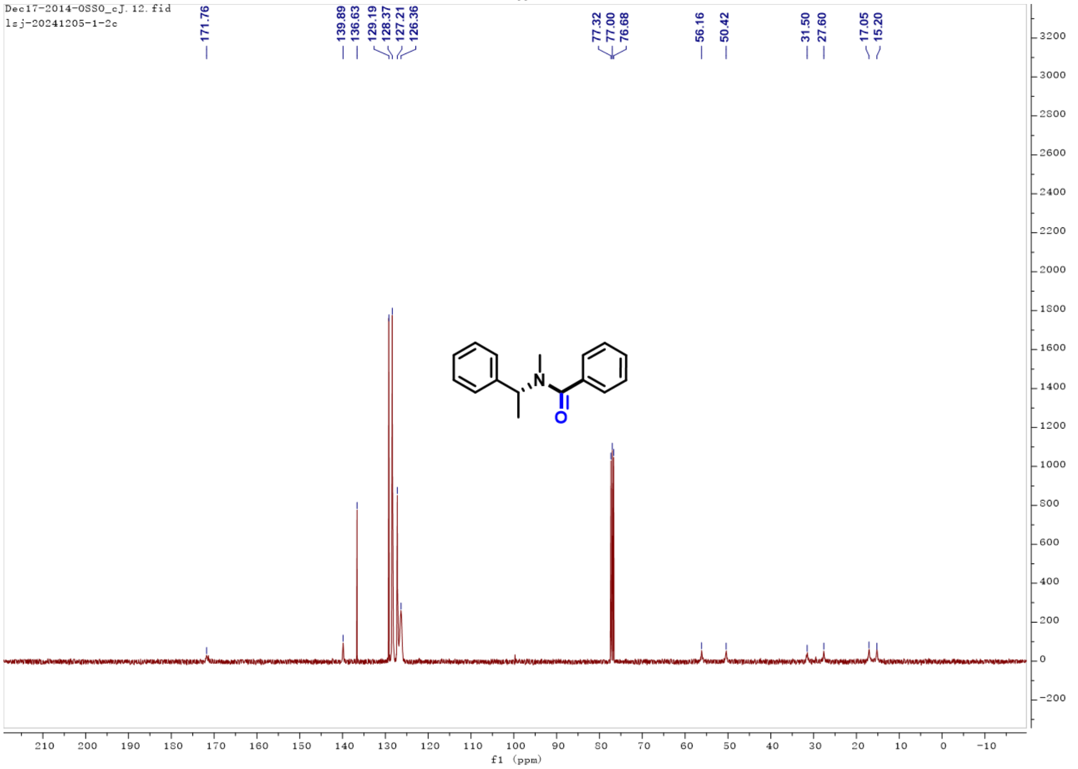

3bg ***(R)-N*-(hexan-2-yl)benzamide**

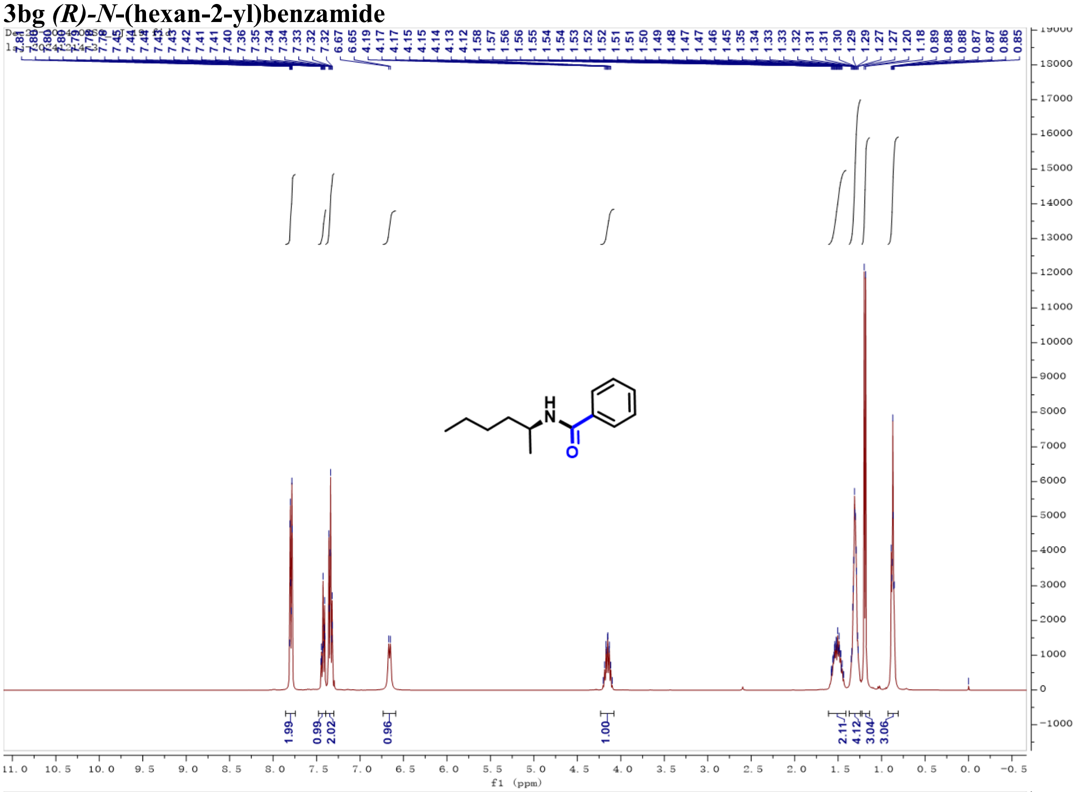

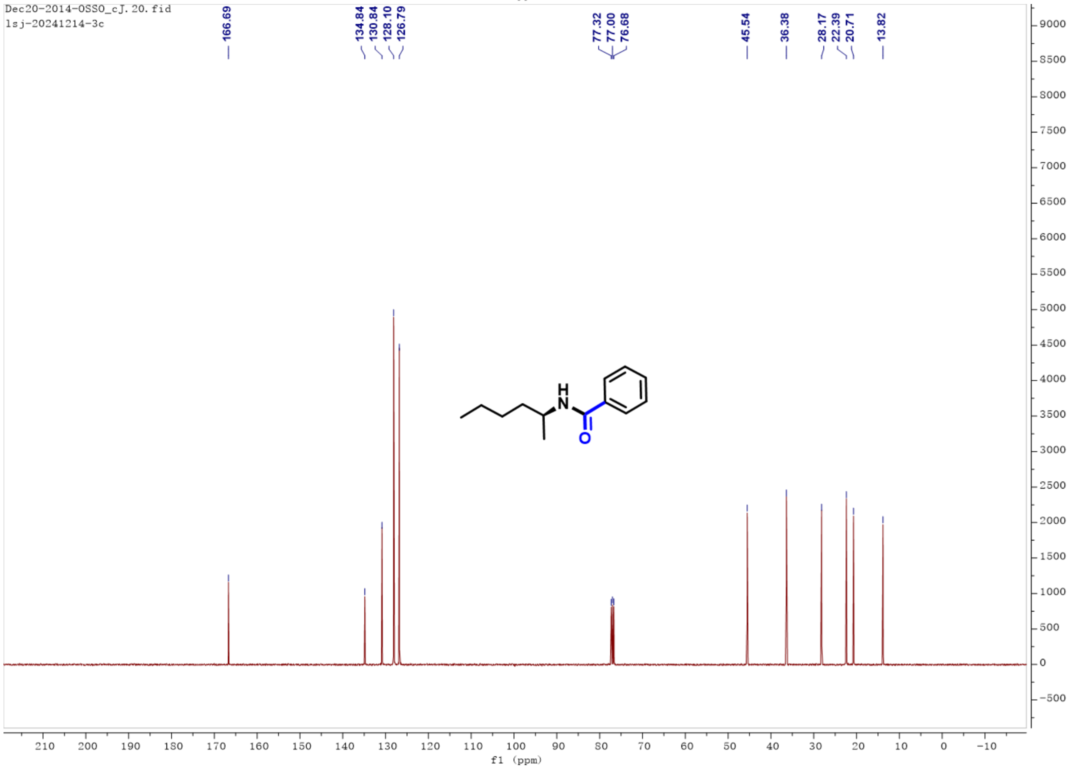

3bh ***((2S,6R)*-2,6-dimethylmorpholino)(phenyl)methanone**

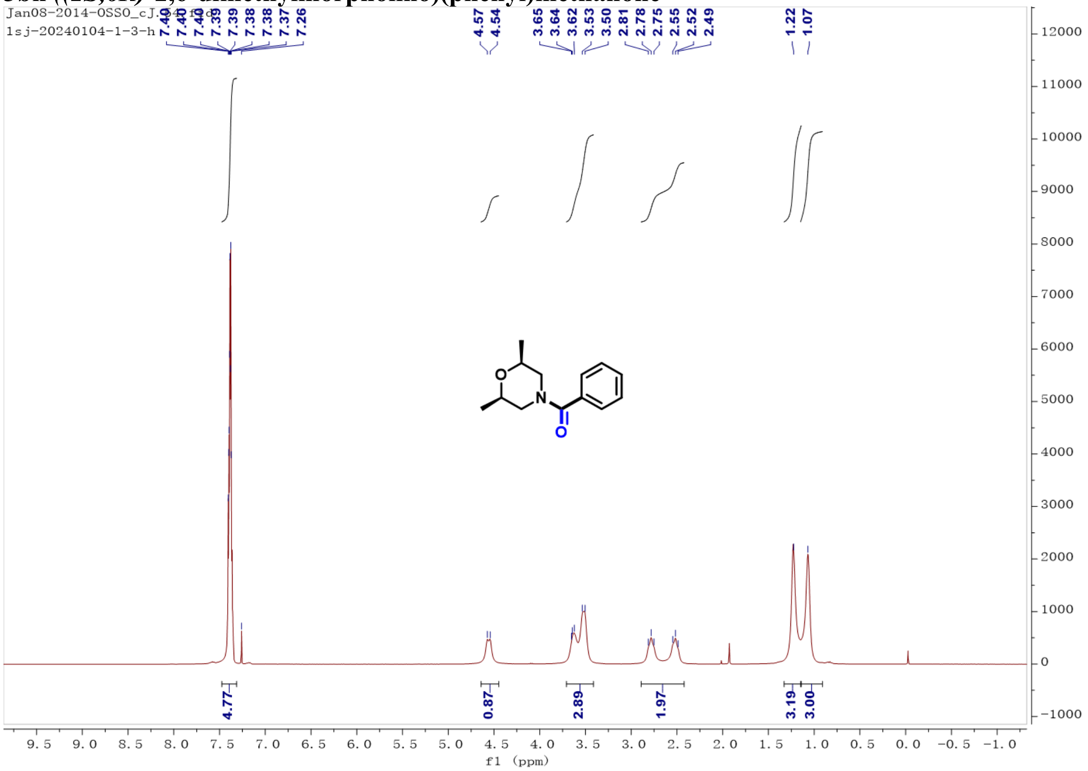

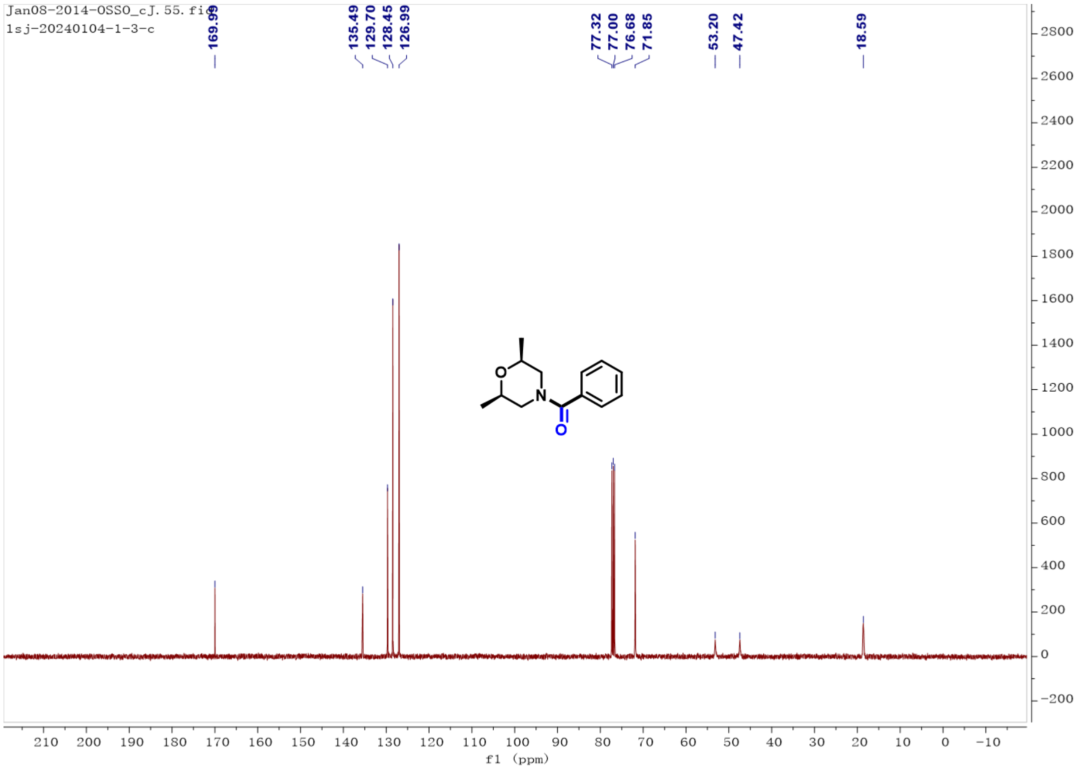

3bi (*R*)-*N*-(1-(naphthalen-1-yl)ethyl)benzamide

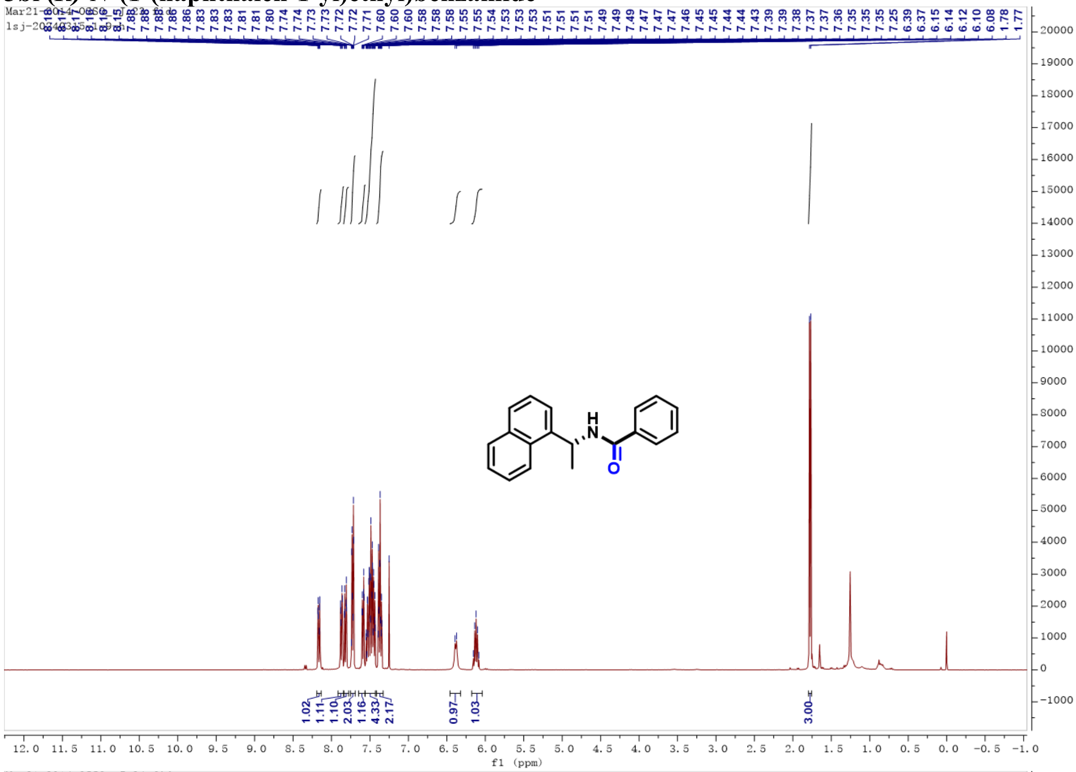

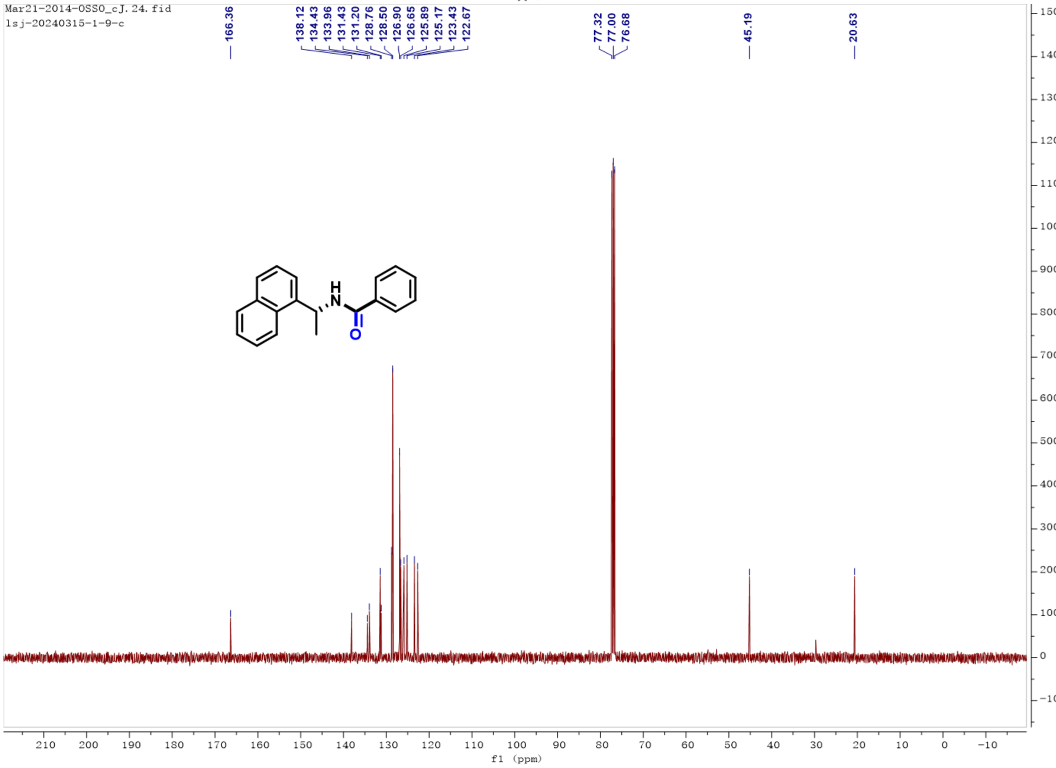

4be *N*-methyl-2-oxo-N-pentyl-2-phenylacetamide

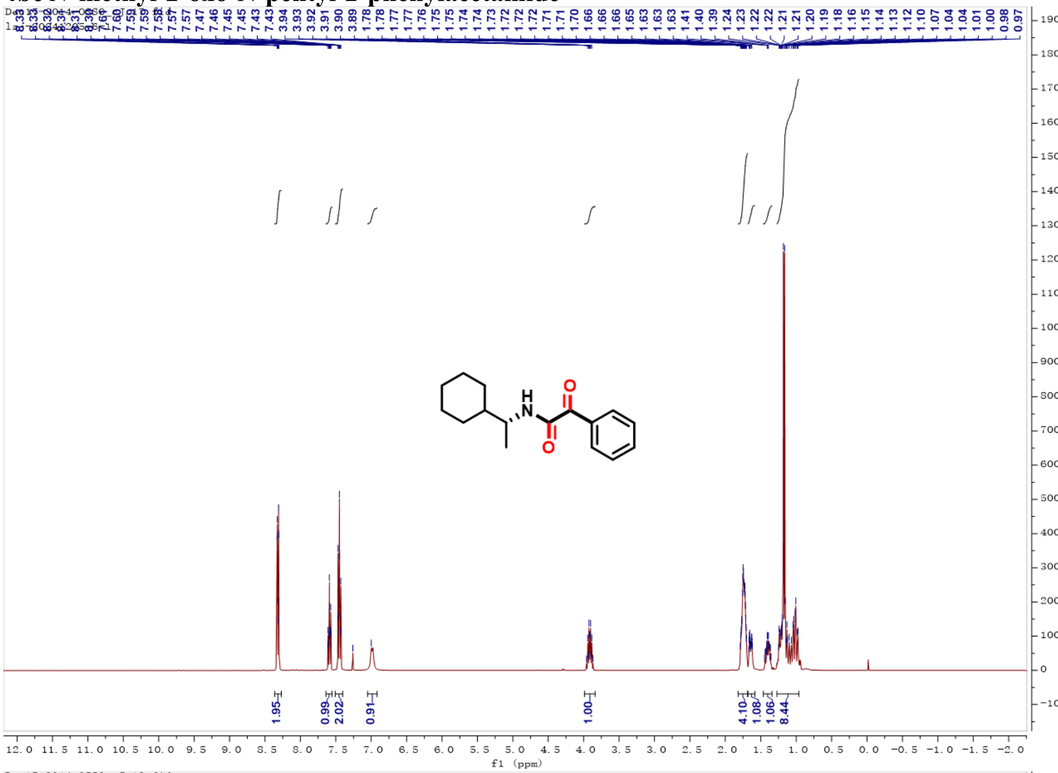

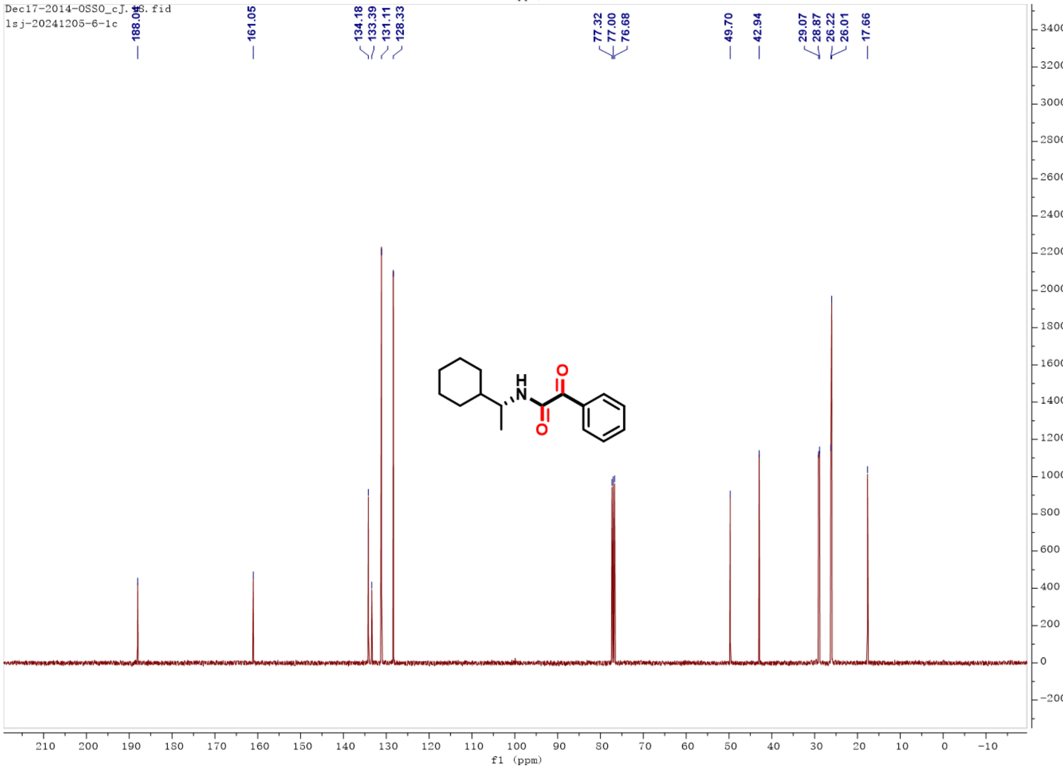

4bf *(R)*-*N*-methyl-2-oxo-2-phenyl-*N*-(1-phenylethyl)acetamide

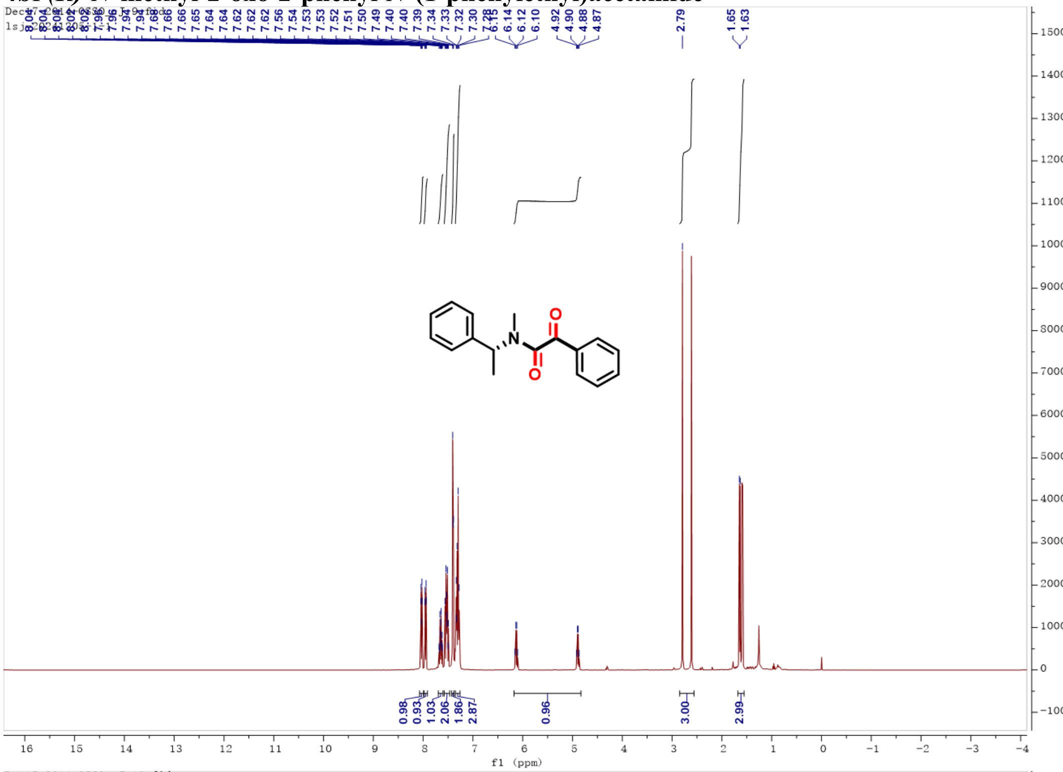

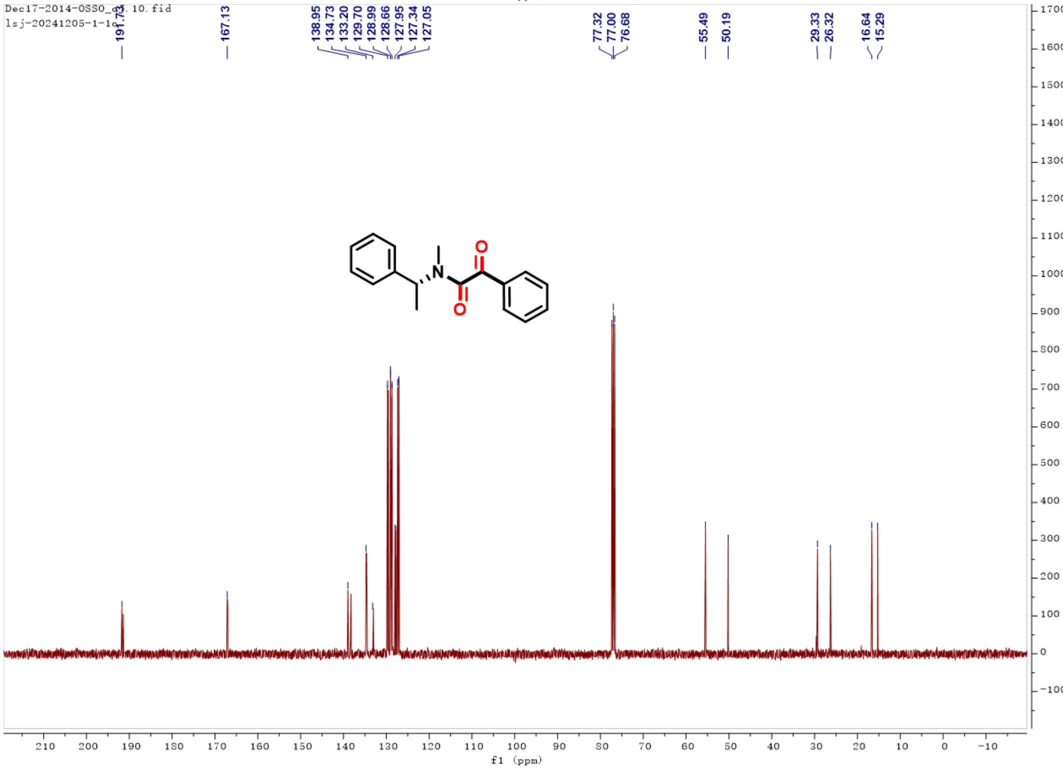

4bg *N*-methyl-2-oxo-N-pentyl-2-phenylacetamide

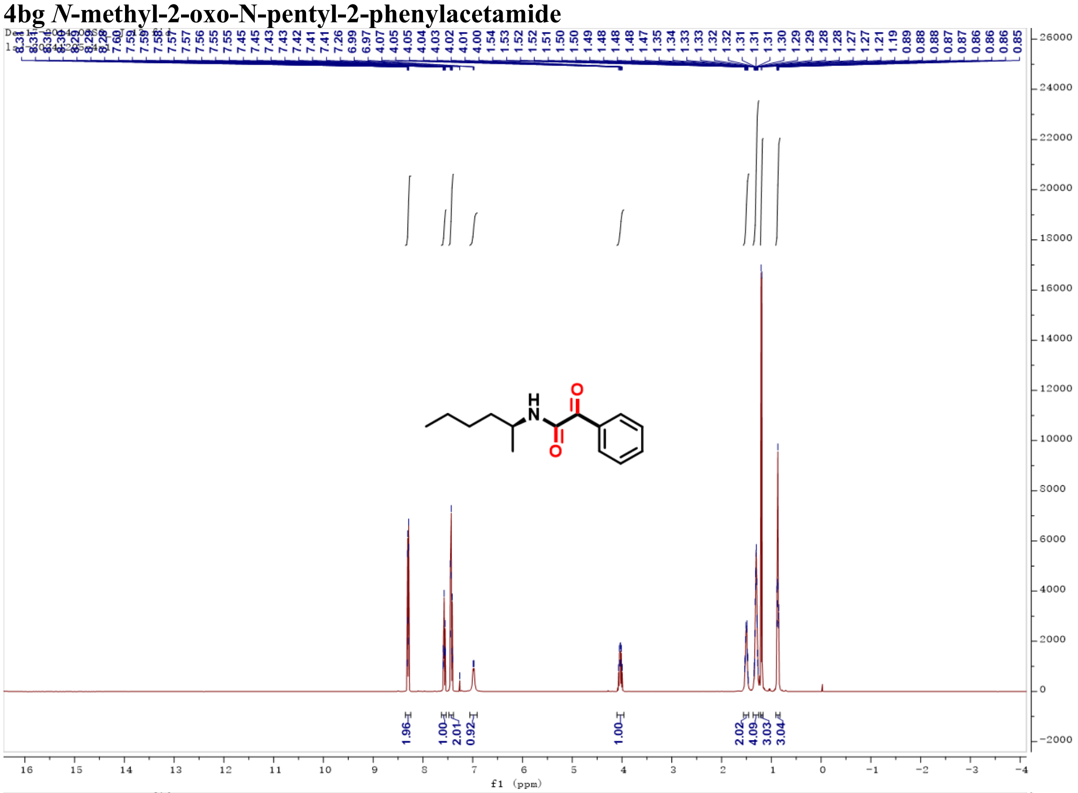

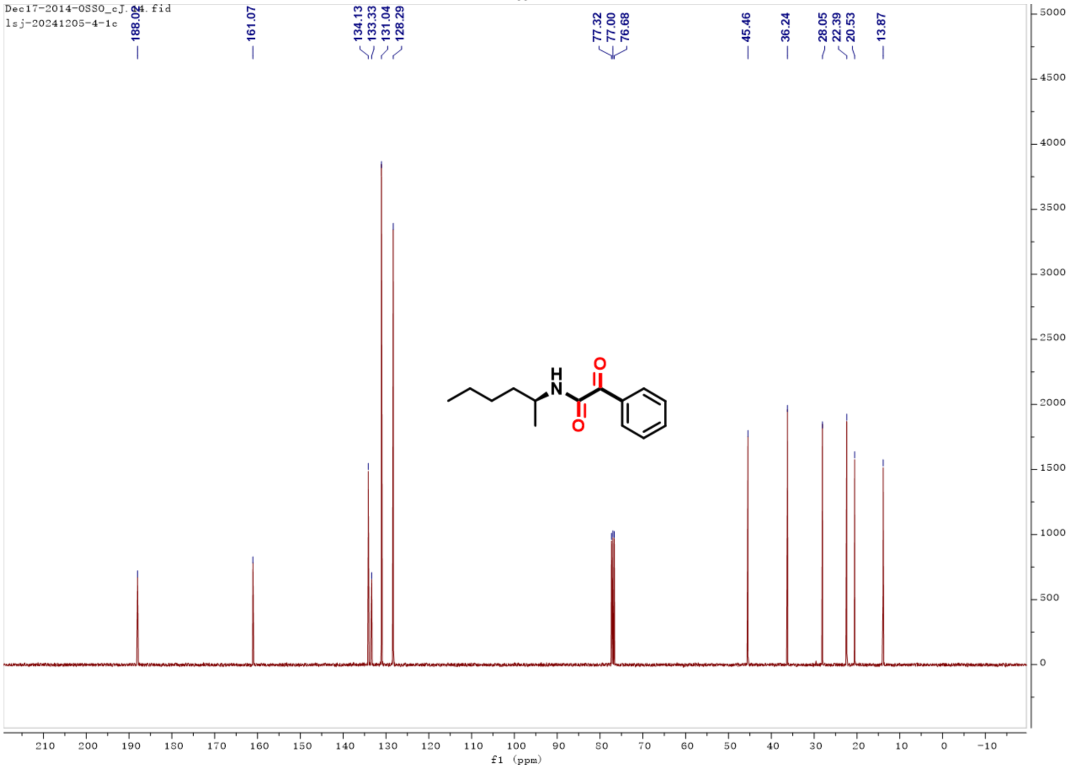

4bh 1-*((2R,6S)*-2,6-dimethylmorpholino)-2-phenylethane-1,2-dione

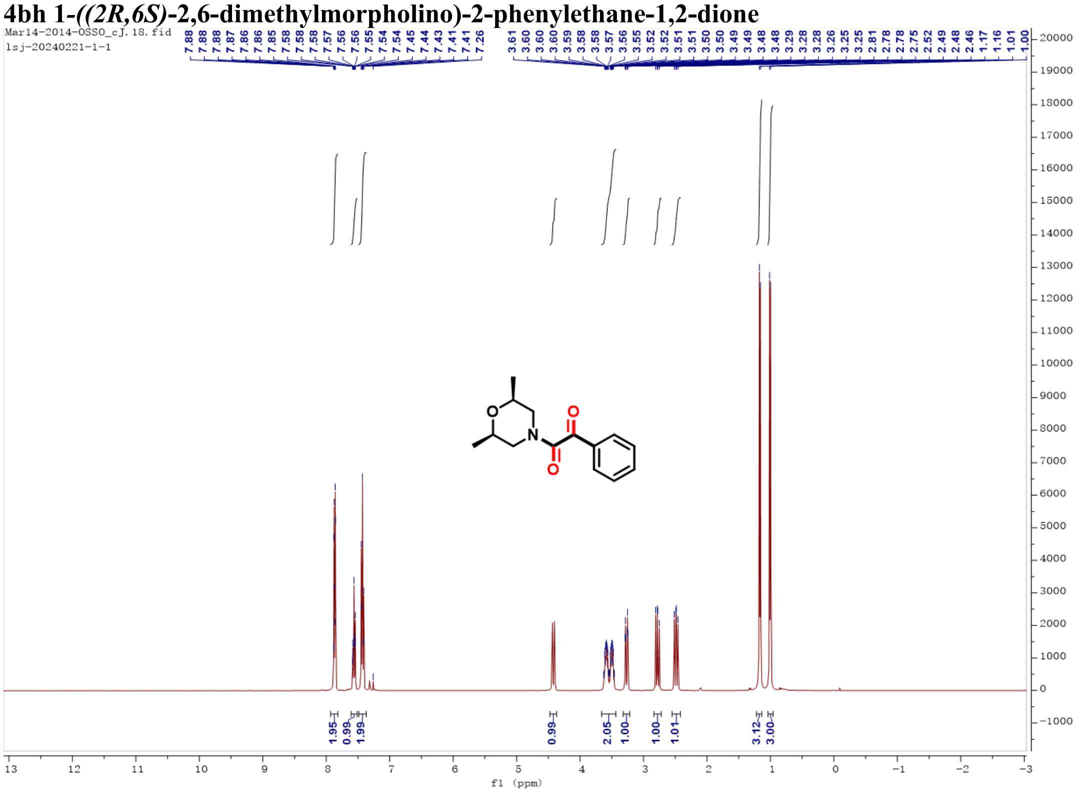

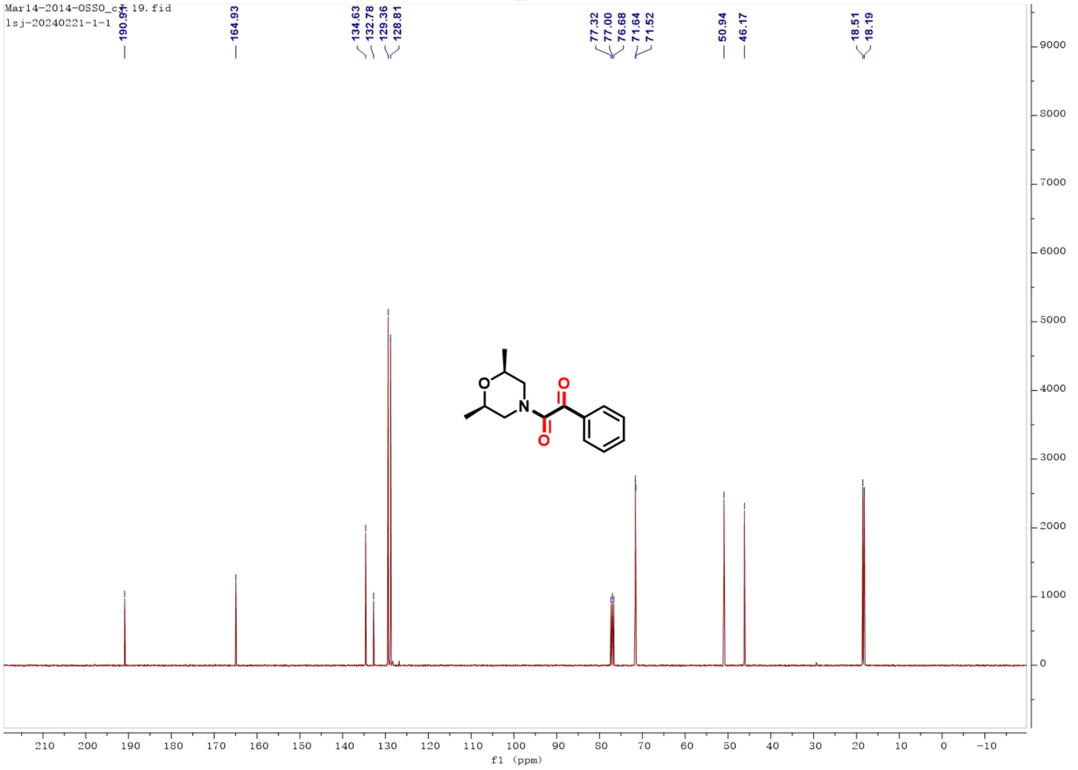

4bi *(R)*-*N*-(1-(naphthalen-1-yl)ethyl)-2-oxo-2-phenylacetamide

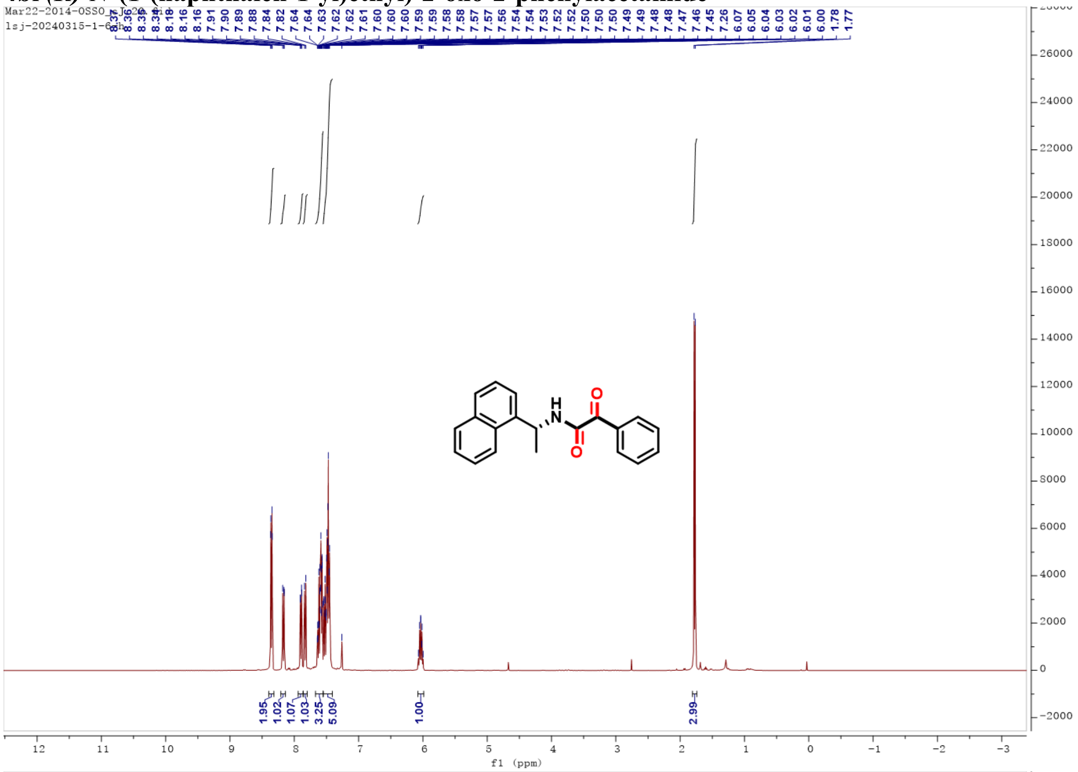

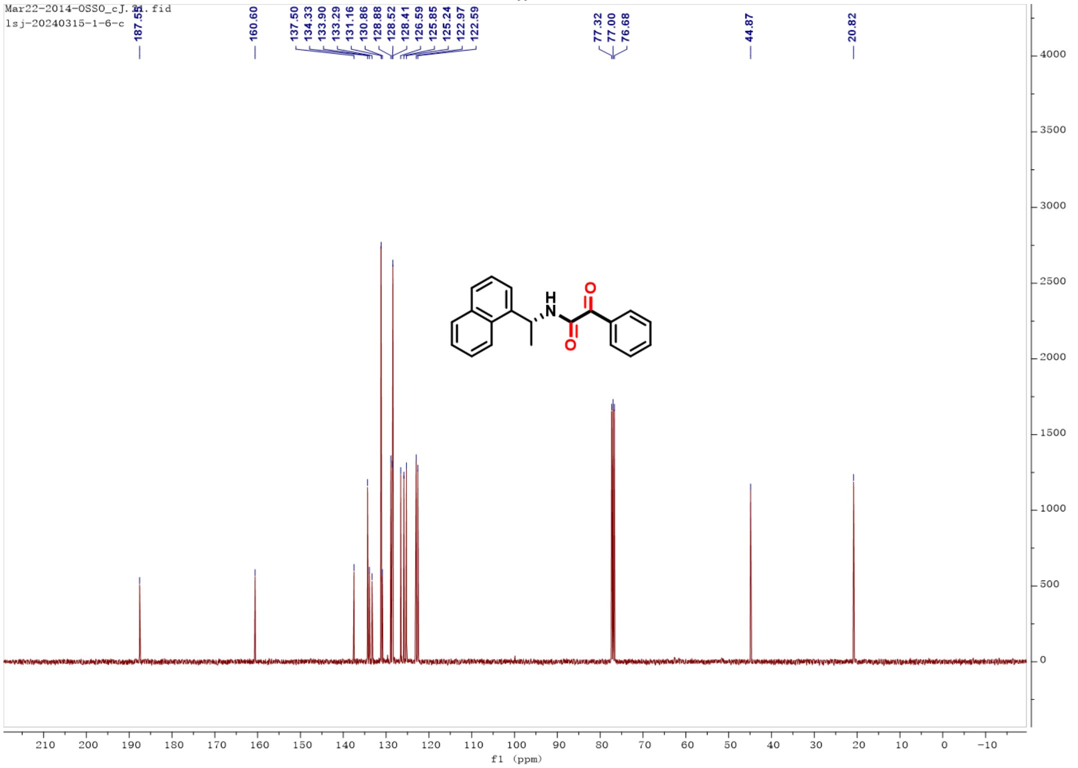

3bj CX-546

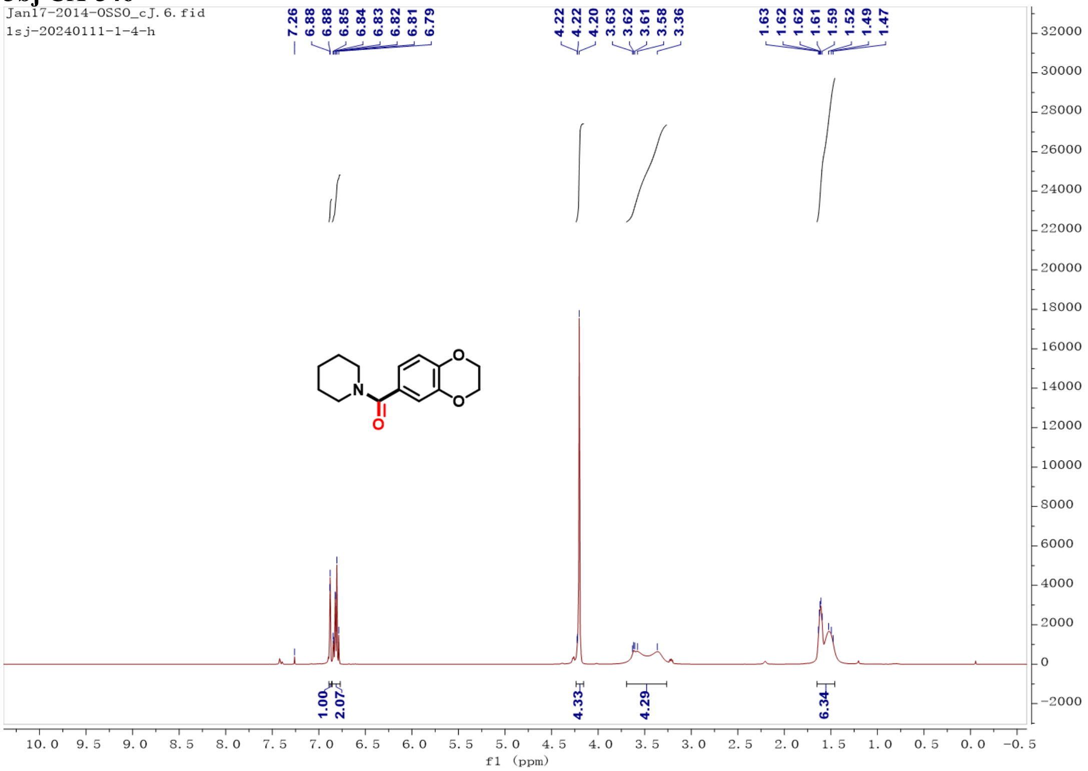
Jan17-2014-OSSO_cJ.6.fid
1sj-20240111-1-4-h
7.26
6.88
6.88
6.85
6.84
6.83
6.82
6.81
6.79
4.22
4.22
4.20
3.63
3.62
3.61
3.58
3.36
1.63
1.62
1.62
1.61
1.59
1.52
1.49
1.47
1.00
2.07
4.33
4.29
6.34
f1 (ppm)

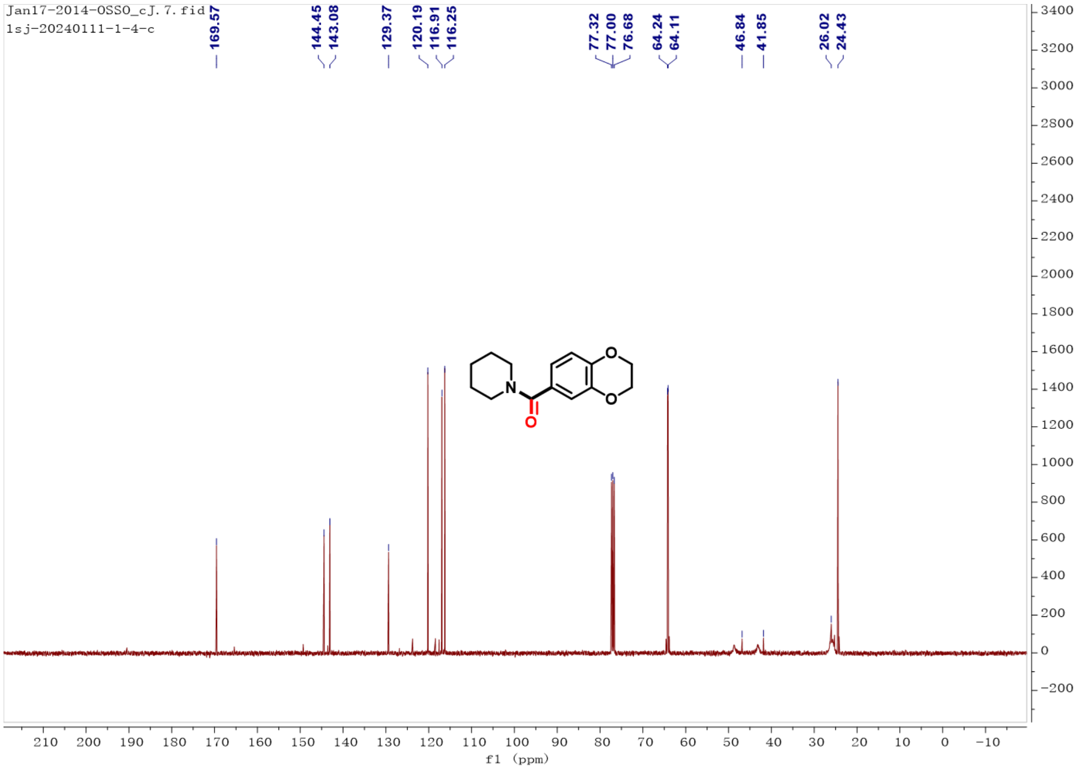
Jan17-2014-OSSO_cJ.7.fid
1sj-20240111-1-4-c
169.57
144.45
143.08
129.37
120.19
116.91
116.25
77.32
77.00
76.68
64.24
64.11
46.84
41.85
26.02
24.43
f1 (ppm)

3ak 4-chloro-*N*-(2-morpholinoethyl)benzamide (Moclobemide)

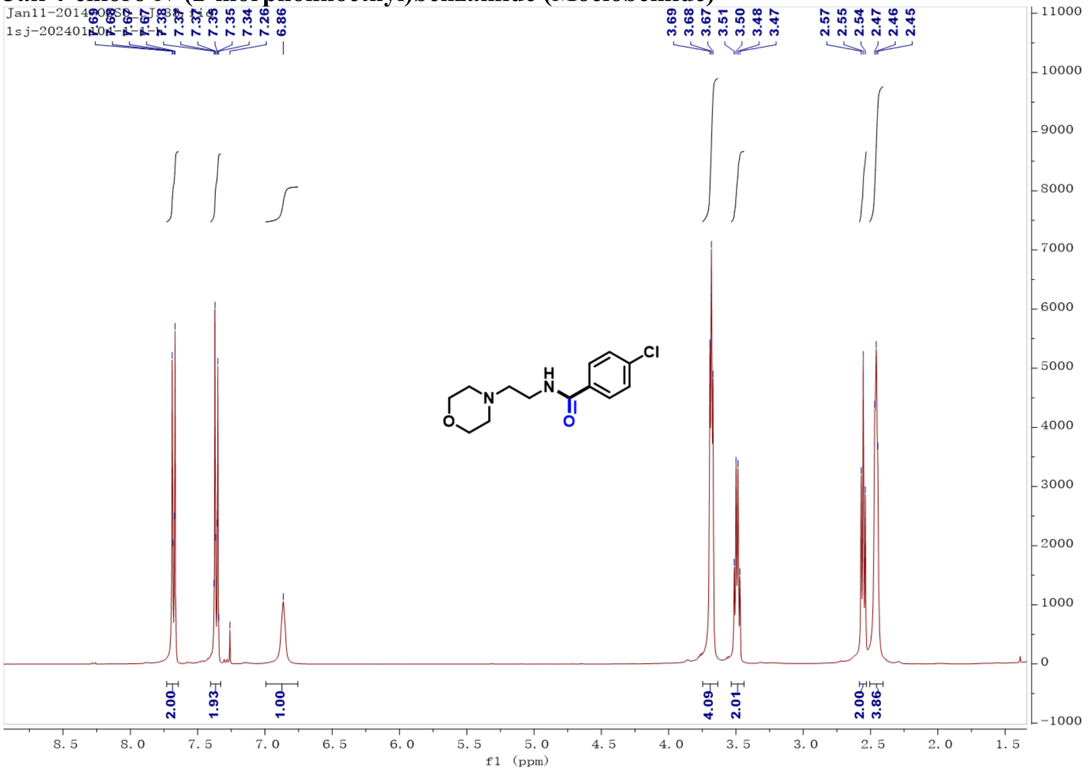

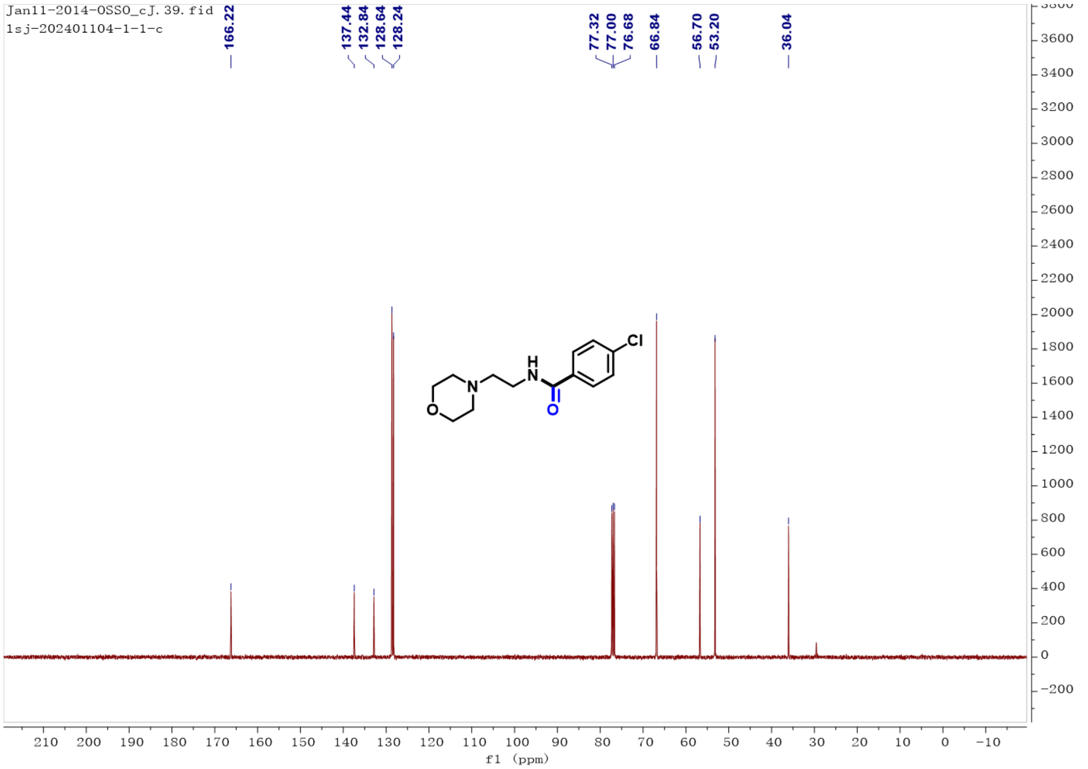

3bl *N,N*-diethylnicotinamide (Nikethamide)

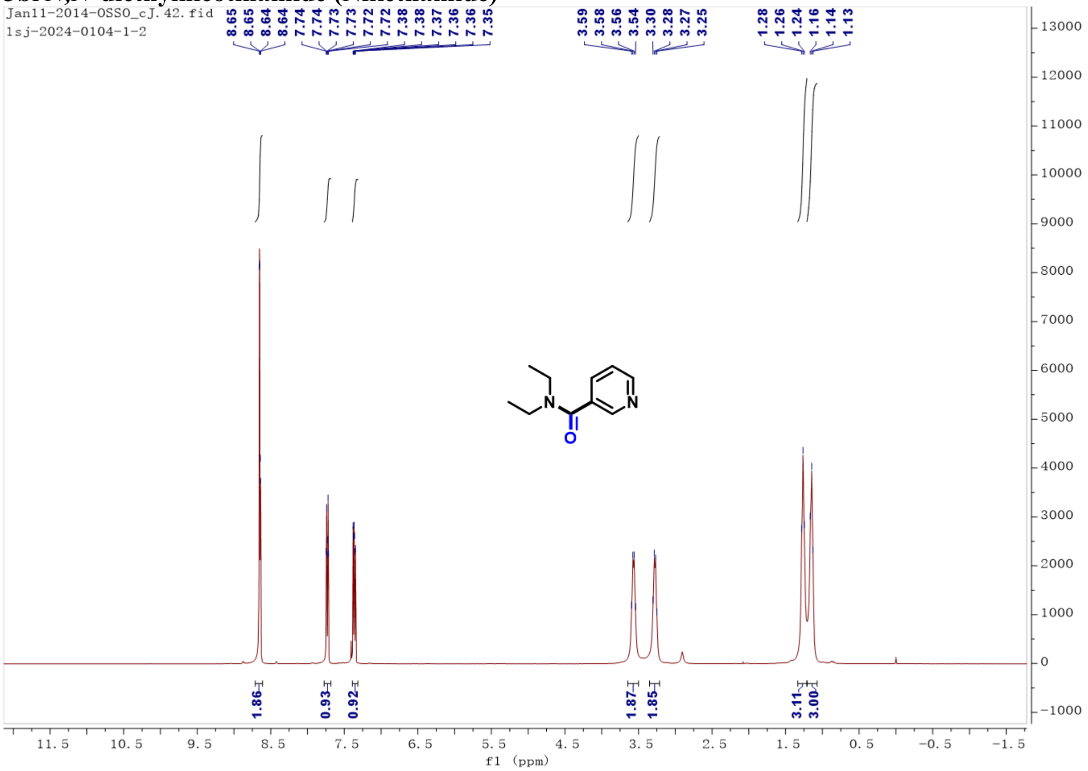

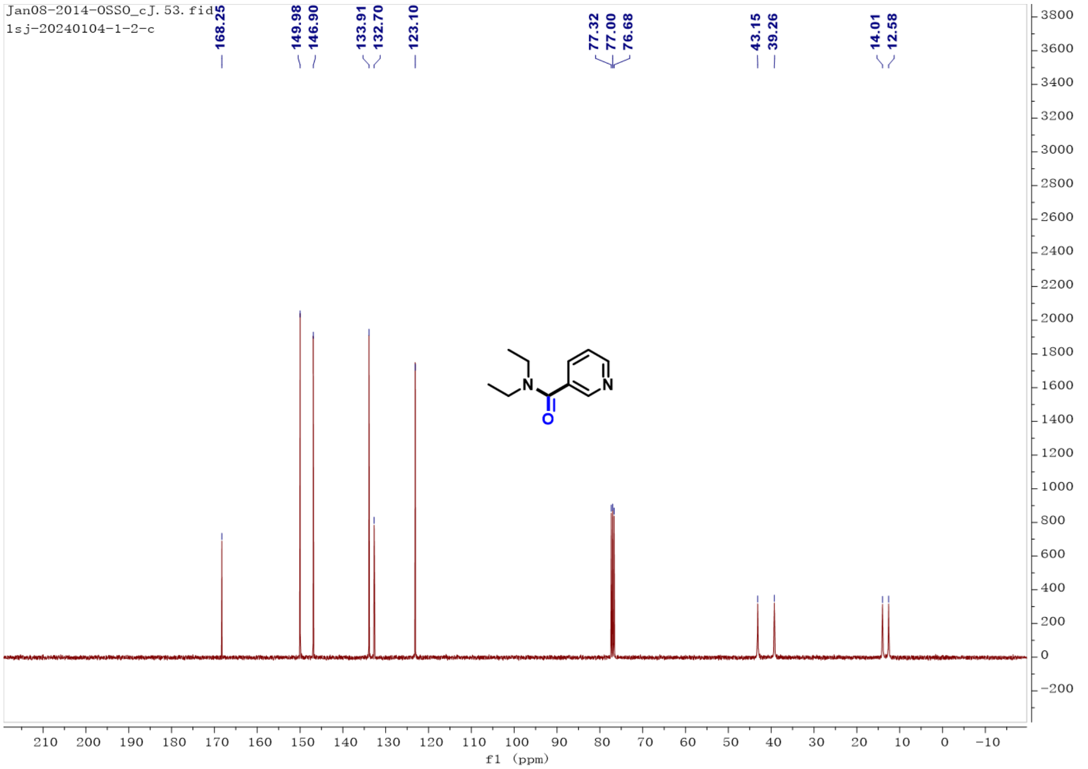

3bm *N*-(2-methoxyphenyl)-2-((4-nitrophenyl)thio)benzamide (RN-18)

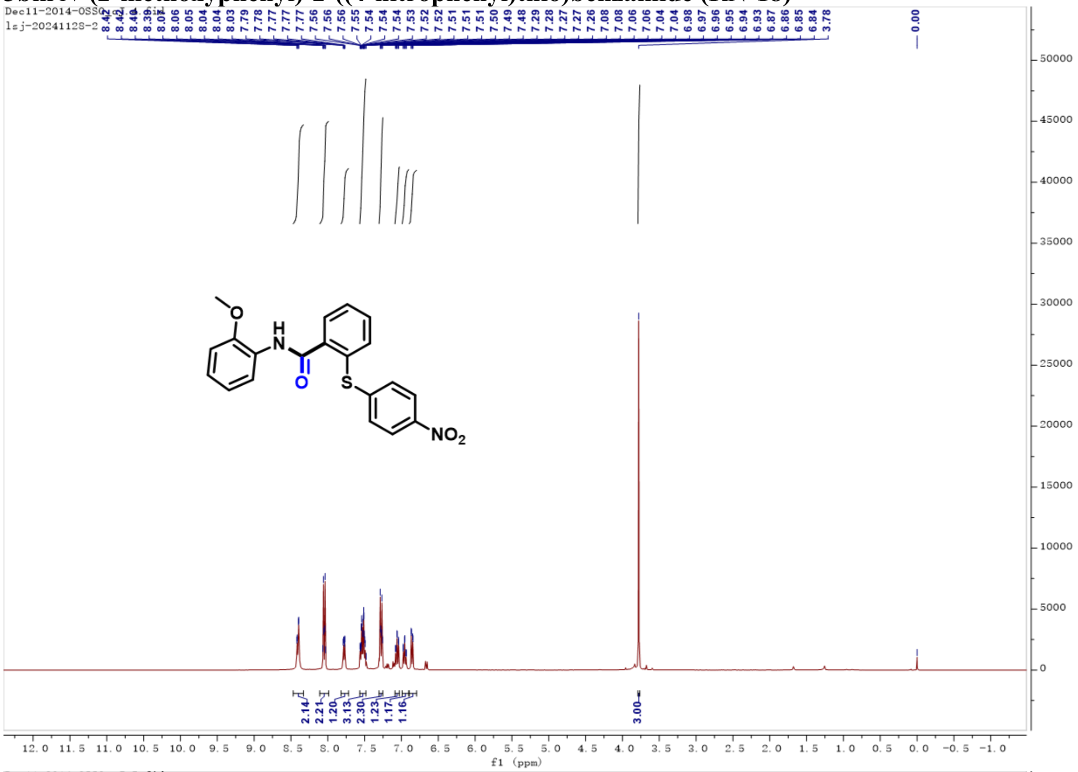

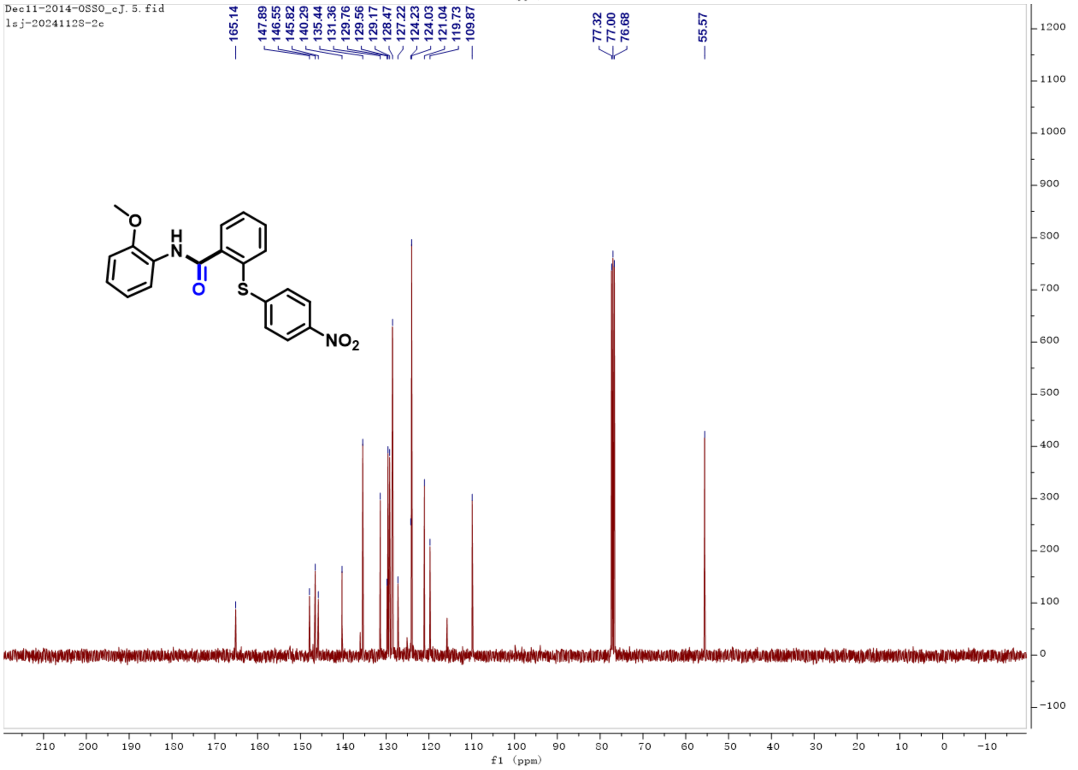

3bn *N,N*-diethyl-4-hydroxy-3-methoxybenzamide (Etamivan)

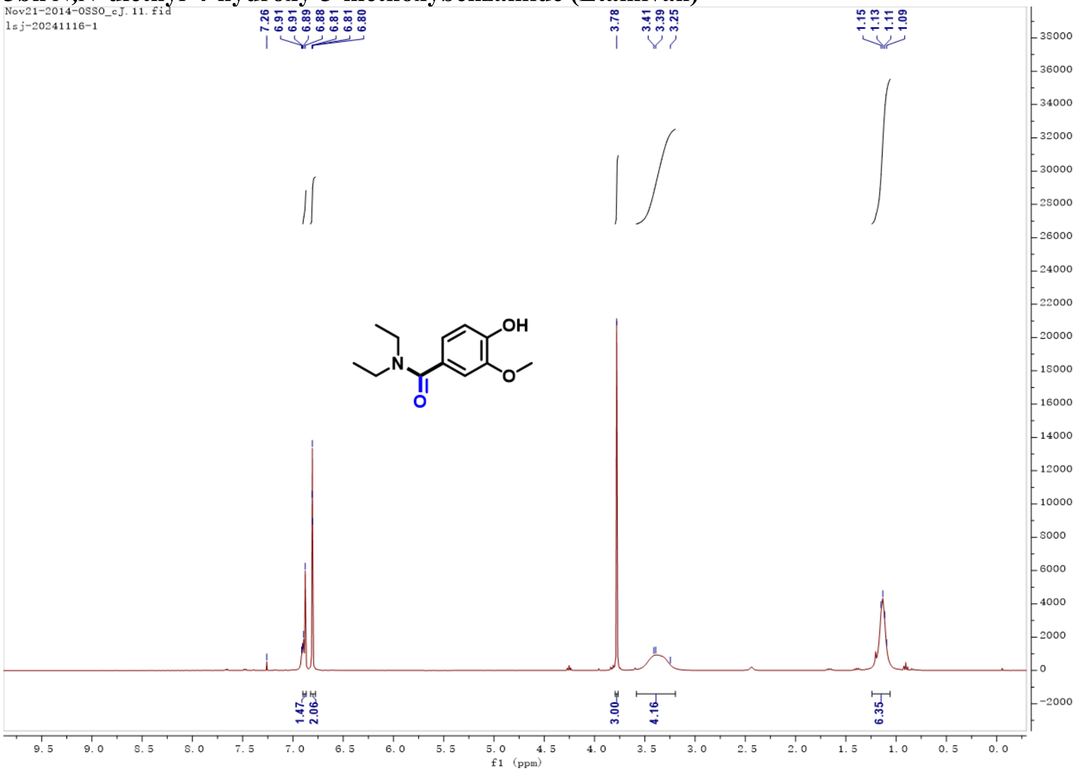

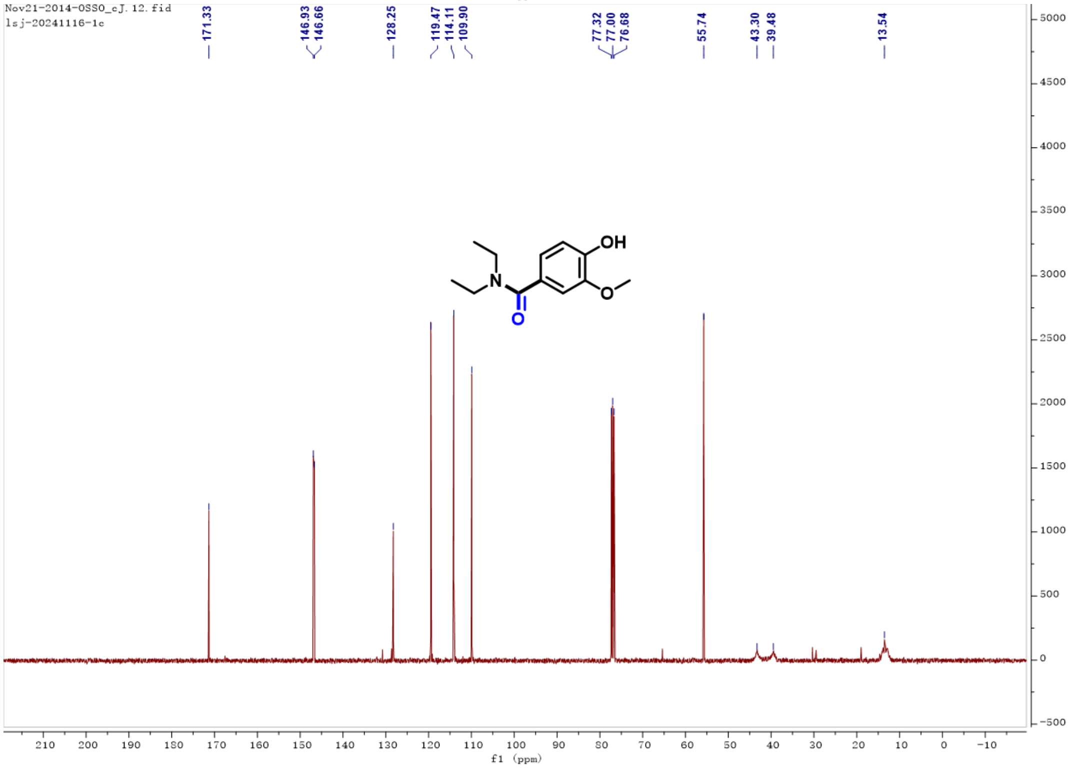

3bo (5-bromoindolin-1-yl)(3,4,5-trimethoxyphenyl)methanone (IV-23)

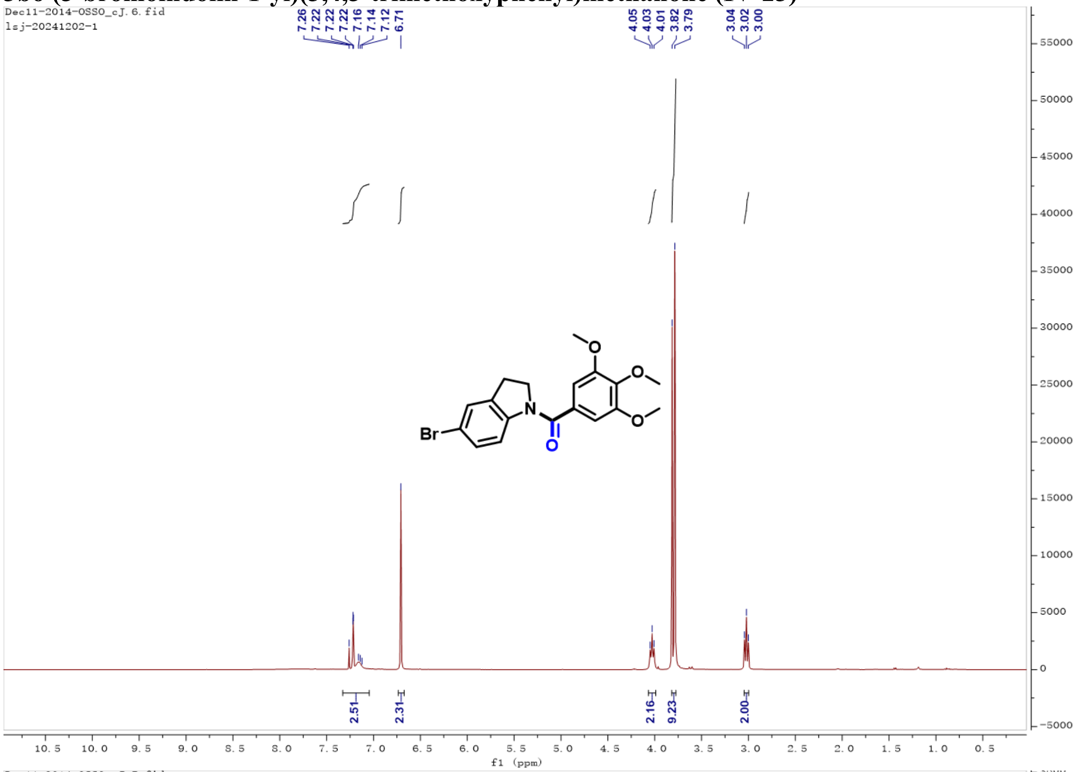

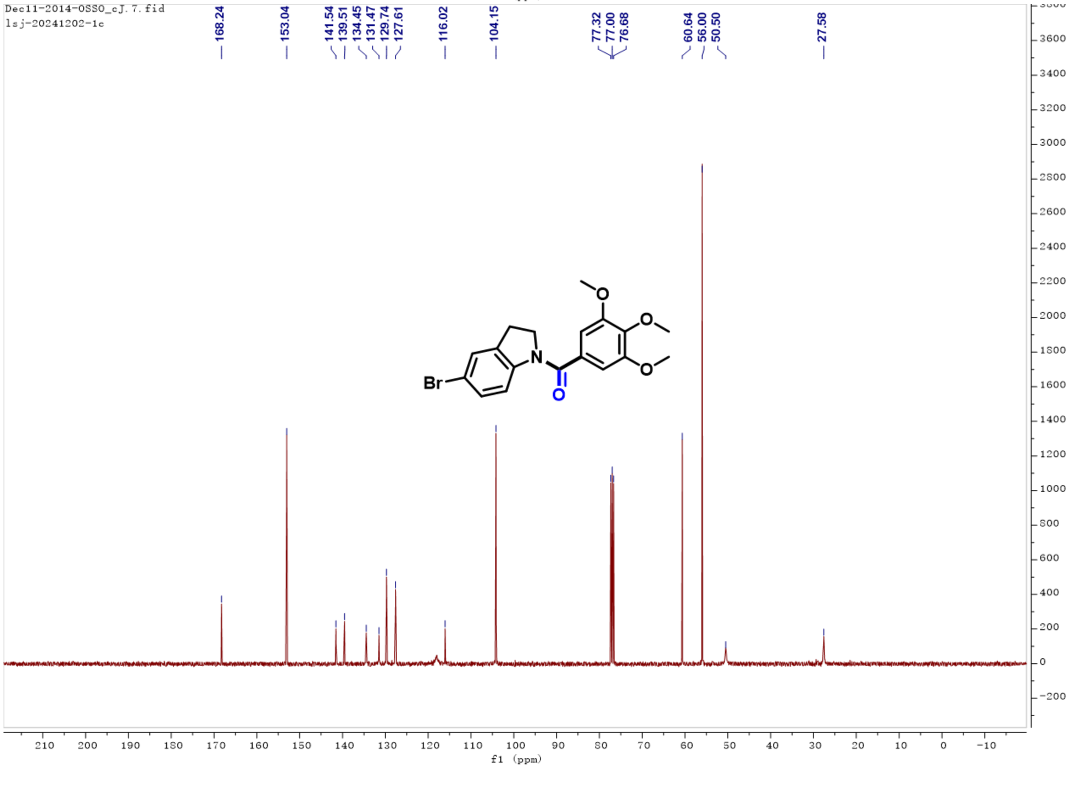

3bp ***N*****-(4-(2-(dimethylamino)ethoxy)benzyl)-3,4,5-trimethoxybenzamide**

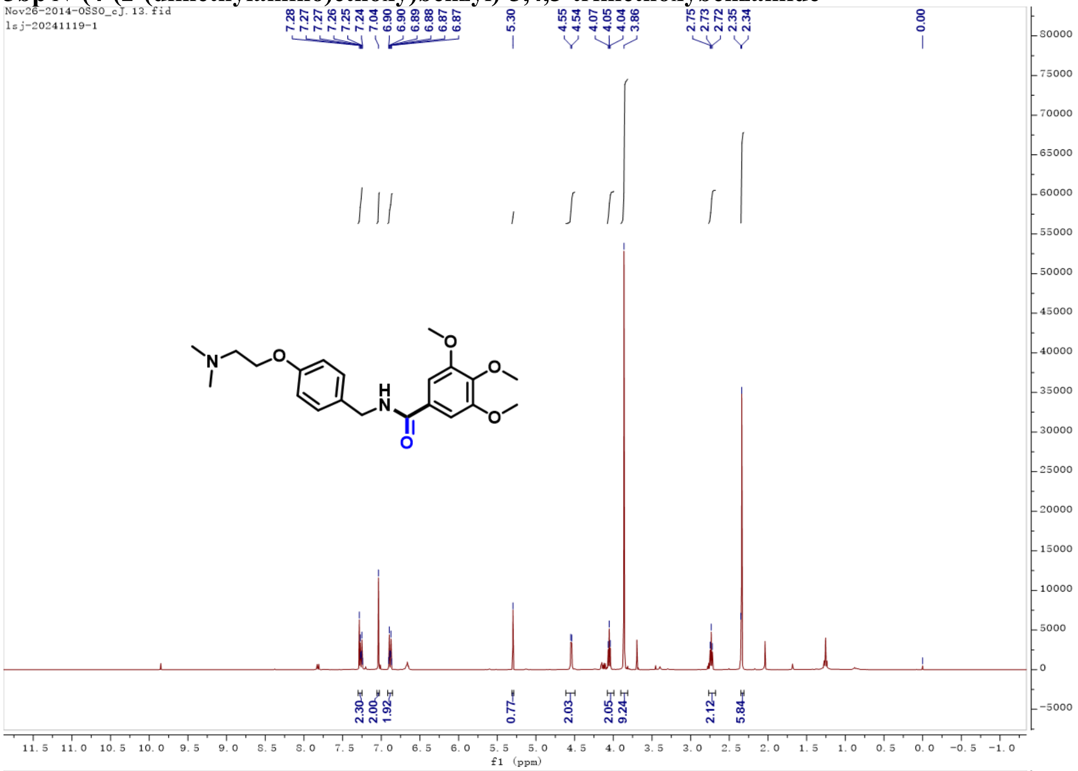

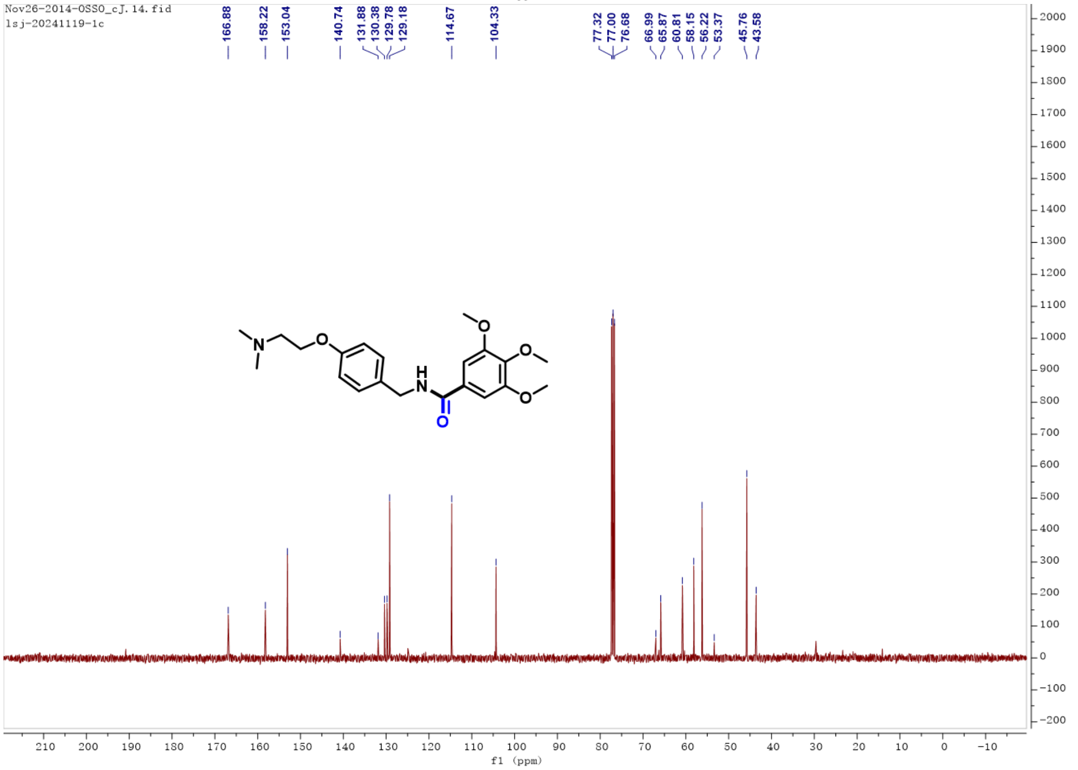

5a 2-((4-methoxyphenyl)amino)-1-morpholino-2-phenylethan-1-one

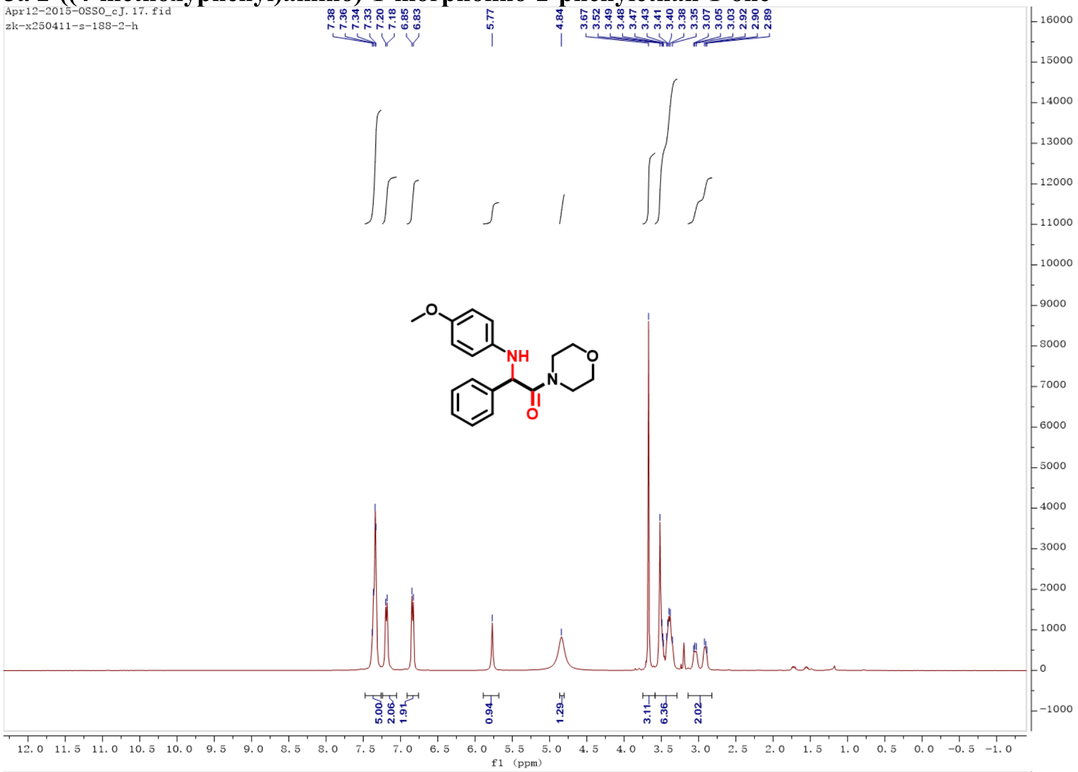

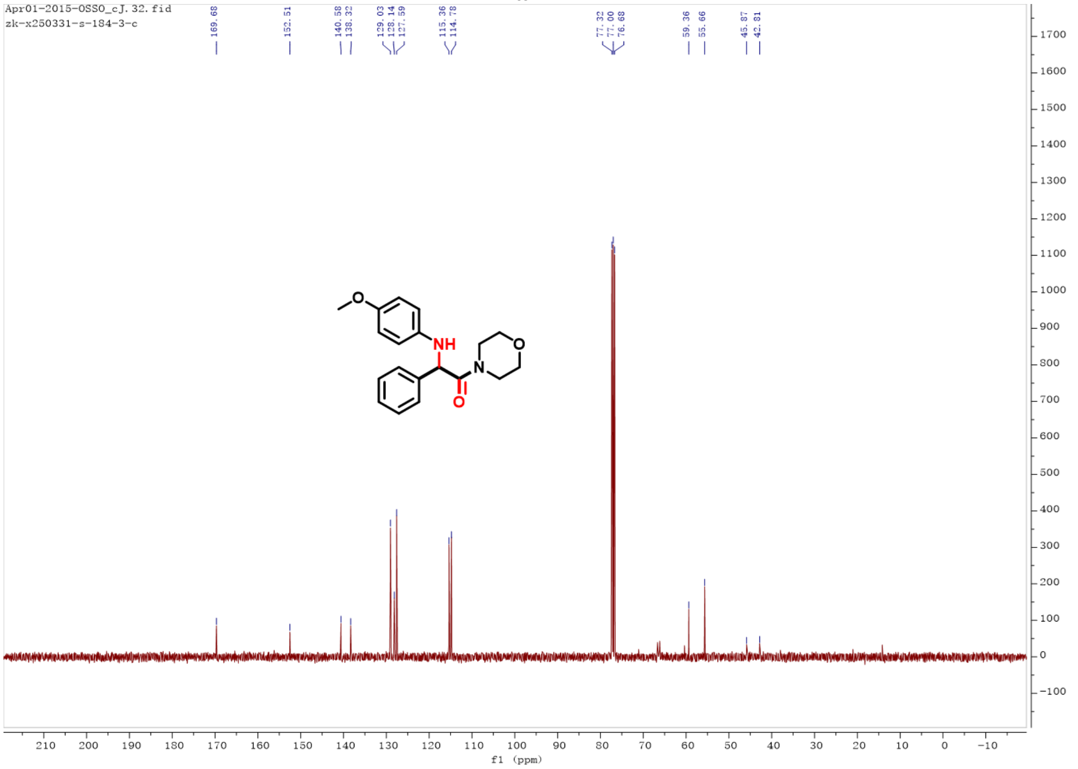

REFERENCES

1. S. Agudo-Álvarez, S. S. Díaz-Mínguez, R. Benito-Arenas, The amide group and its preparation methods by acid-amine coupling reactions: An overview. *Pure Appl. Chem.* **96**, 691–707 (2024).

2. V. R. Pattabiraman, J. W. Bode, Rethinking amide bond synthesis. *Nature* **480**, 471–479 (2011).

3. B. C. Haas, A. E. Goetz, A. Bahamonde, J. C. McWilliams, M. S. Sigman, Predicting relative efficiency of amide bond formation using multivariate linear regression. *Proc. Natl. Acad. Sci. U.S.A.* **119**, e2118451119 (2022).

4. H. Liu, G. Laurenczy, N. Yan, P. J. Dyson, Amide bond formation via C(sp^3)-H bond functionalization and CO insertion. *Chem. Commun.* **50**, 341–343 (2014).

5. N. E. Leadbeater, M. Marco, Preparation of polymer-supported ligands and metal complexes for use in catalysis. *Chem. Rev.* **102**, 3217–3274 (2002).

6. L. Zeng, H. Li, J. Hu, D. Zhang, J. Hu, P. Peng, S. Wang, R. Shi, J. Peng, C.-W. Pao, J.-L. Chen, J.-F. Lee, H. Zhang, Y.-H. Chen, A. Lei, Electrochemical oxidative aminocarbonylation of terminal alkynes. *Nat. Catal.* **3**, 438–445 (2020).

7. L. J. Lu, X. L. Pei, Y. Mei, Y. Deng, H. Zhang, L. N. Zhang, A. W. Lei, Carbon nanofibrous microspheres promote the oxidative double carbonylation of alkanes with CO. *Chem* **4**, 2861–2871 (2018).

8. Y. W. Cao, Y. Peng, D. Y. Cheng, L. Chen, M. L. Wang, C. Shang, L. R. Zheng, D. Ma, Z. P. Liu, L. He, Room-temperature co oxidative coupling for oxamide production over interfacial Au/ZnO catalysts. *ACS Catal.* **13**, 735–743 (2023).

9. Y. Zhang, H. Q. Geng, X. F. Wu, Palladium-catalyzed perfluoroalkylative carbonylation of unactivated alkenes: Access to beta-perfluoroalkyl esters. *Angew. Chem. Int. Ed. Engl.* **60**, 24292–24298 (2021).

10. C. De Risi, G. P. Pollini, V. Zanirato, Recent developments in general methodologies for the synthesis of α-ketoamides. *Chem. Rev.* **116**, 3241–3305 (2016).

11. F. Zhao, H.-J. Ai, X.-F. Wu, Copper-catalyzed substrate-controlled carbonylative synthesis of α-Keto amides and amides from alkyl halides. *Angew. Chem. Int. Ed. Engl.* **61**, e202200062 (2022).

12. S.-Q. Yang, Y.-Q. Yao, X.-C. Chen, Y. Lu, X.-L. Zhao, Y. Liu, Pd-catalyst containing a hemilabile P,C-hybrid ligand in amino dicarbonylation of aryl halides for synthesis of α-ketoamides. *Organometallics* **40**, 1032–1041 (2021).

13. N. Uzunlu, P. Pongracz, L. Kollar, A. Takacs, Alkyl levulinates and 2-methyltetrahydrofuran: Possible biomass-based solvents in palladium-catalyzed aminocarbonylation. *Molecules* **28**, 1–15 (2023).

14. B. Aranda, S. A. Moya, A. Vega, G. Valdebenito, S. Ramirez-Lopez, P. Aguirre, New palladium (II) complexes containing phosphine-nitrogen ligands and their use as catalysts in aminocarbonylation reaction. *Appl. Organomet. Chem.* **33**, e4709 (2019).

15. M. Genelot, N. Villandier, A. Bendjeriou, P. Jaithong, L. Djakovitch, V. Dufaud, Palladium complexes grafted onto mesoporous silica catalysed the double carbonylation of aryl iodides with amines to give α-ketoamides. *Cat. Sci. Technol.* **2**, 1886 (2012).

16. M. Papp, R. Skoda-Földes, Phosphine-free double carbonylation of iodobenzene in the presence of reusable supported palladium catalysts. *J. Mol. Catal. A Chem.* **378**, 193–199 (2013).

17. B. Urbán, E. Nagy, P. Nagy, M. Papp, R. Skoda-Földes, Double carbonylation of iodoarenes in the presence of a pyridinium SILP-Pd catalyst. *J. Organomet. Chem.* **918**, 121287 (2020).

18. M. Papp, P. Szabó, D. Srankó, G. Sáfrán, L. Kollár, R. Skoda-Földes, Mono- and double carbonylation of aryl iodides with amine nucleophiles in the presence of recyclable palladium catalysts immobilised on a supported dicationic ionic liquid phase. *RSC Adv.* **7**, 44587–44597 (2017).

19. A. W. Heard, J. M. Suárez, S. M. Goldup, Controlling catalyst activity, chemoselectivity and stereoselectivity with the mechanical bond. *Nat. Rev. Chem.* **6**, 182–196 (2022).

20. A. Milo, E. N. Bess, M. S. Sigman, Interrogating selectivity in catalysis using molecular vibrations. *Nature* **507**, 210–214 (2014).

21. B. Zhang, J. Wang, G. Liu, C. M. Weiss, D. Liu, Y. Chen, L. Xia, P. Zhou, M. Gao, Y. Liu, J. Chen, Y. Yan, M. Shao, H. Pan, W. Sun, A strongly coupled Ru-CrO_x

cluster-cluster heterostructure for efficient alkaline hydrogen electrocatalysis. *Nat. Catal.* **7**, 441–451 (2024).

22. A. Parastaev, V. Muravev, E. H. Osta, T. F. Kimpel, J. F. M. Simons, A. J. F. van Hoof, E. Uslamin, L. Zhang, J. J. C. Struijs, D. B. Burueva, E. V. Pokochueva, K. V. Kovtunov, I. V. Koptyug, I. J. Villar-Garcia, C. Escudero, T. Altantzis, P. Liu, A. Béché, S. Bals, N. Kosinov, E. J. M. Hensen, Breaking structure sensitivity in CO_2 hydrogenation by tuning metal-oxide interfaces in supported cobalt nanoparticles. *Nat. Catal.* **5**, 1051–1060 (2022).

23. S. Li, Y. Xu, Y. Chen, W. Li, L. Lin, M. Li, Y. Deng, X. Wang, B. Ge, C. Yang, S. Yao, J. Xie, Y. Li, X. Liu, D. Ma, Tuning the selectivity of catalytic carbon dioxide hydrogenation over iridium/cerium oxide catalysts with a strong metal-support interaction. *Angew. Chem. Int. Ed. Engl.* **56**, 10761–10765 (2017).

24. R. Gao, J. Xu, J. Wang, J. Lim, C. Peng, L. Pan, X. Zhang, H. Yang, J. J. Zou, Pd/Fe_2O_3 with electronic coupling single-site Pd-Fe pair sites for low-temperature semihydrogenation of alkynes. *J. Am. Chem. Soc.* **144**, 573–581 (2022).

25. Z. S. Zhu, S. Zhong, C. Cheng, H. Zhou, H. Sun, X. Duan, S. Wang, Microenvironment engineering of heterogeneous catalysts for liquid-phase environmental catalysis. *Chem. Rev.* **124**, 11348–11434 (2024).

26. X. Dai, S. Adomeit, J. Rabeah, C. Kreyenschulte, A. Bruckner, H. Wang, F. Shi, Sustainable co-synthesis of glycolic acid, formamides and formates from 1,3-dihydroxyacetone by a Cu/Al_2O_3 catalyst with a single active sites. *Angew. Chem. Int. Ed. Engl.* **58**, 5251–5255 (2019).

27. Z. Zhao, P. Wang, C. Song, T. Zhang, S. Zhan, Y. Li, Enhanced interfacial electron transfer by asymmetric Cu-O_v-In sites on In_2O_3 for efficient peroxymonosulfate activation. *Angew. Chem. Int. Ed. Engl.* **62**, e202216403 (2023).

28. X. Zhang, F. Kraushofer, Q. Yuan, Y.-X. Wang, M. Krinninger, Z. Su, H. Gai, K. Goodman, X. Zhang, Y. Wang, X. Tong, T. Cheng, J.-F. Wu, B. A. J. Lechner, M. Blum, Pd-promoted reduction and restructuring of an In2O3-based catalyst for CO2 hydrogenation at room temperature. *J. Catal.* **454**, 116618 (2026).

29. Q. Jiang, X. Li, Y. Hao, J. Zuo, R. Duan, J. Li, G. Cao, J. Wang, J. Wang, M. Li, X. Yang, M. Li, W. Li, Y. Xi, J. Zhang, W. Xiao, Oxygen-vacancy-assisted dual functional surface coatings suppressing irreversible phase transition of Li-rich layered oxide cathodes. *Adv. Funct. Mater.* **35**, 2400670 (2024).

30. C. Huang, Z. Jiang, F. Liu, W. Li, Q. Liang, Z. Zhao, X. Ge, K. Song, L. Zheng, X. Zhou, S. Qiao, W. Zhang, W. Zheng, Oxygen vacancies boosted hydronium intercalation: A paradigm shift in aluminum-based batteries. *Angew. Chem. Int. Ed. Engl.* **63**, e202405592 (2024).

31. L. Luo, L. Fu, H. Liu, Y. Xu, J. Xing, C.-R. Chang, D.-Y. Yang, J. Tang, Synergy of Pd atoms and oxygen vacancies on In_2O_3 for methane conversion under visible light. *Nat. Commun.* **13**, 2930 (2022).

32. S. Chen, H. Wang, Z. Kang, S. Jin, X. Zhang, X. Zheng, Z. Qi, J. Zhu, B. Pan, Y. Xie, Oxygen vacancy associated single-electron transfer for photofixation of CO_2 to long-chain chemicals. *Nat. Commun.* **10**, 788 (2019).

33. X. Zhu, Q. Guo, Y. Sun, S. Chen, J.-Q. Wang, M. Wu, W. Fu, Y. Tang, X. Duan, D. Chen, Y. Wan, Optimising surface d charge of AuPd nanoalloy catalysts for enhanced catalytic activity. *Nat. Commun.* **10**, 1428 (2019).

34. J. Chen, D. Zhang, B. Liu, K. Zheng, Y. Li, Y. Xu, Z. Li, X. Liu, Photoinduced precise synthesis of diatomic Ir_1Pd_1-In_2O_3 for CO_2 hydrogenation to methanol via angstrom-scale-distance dependent synergistic catalysis. *Angew. Chem. Int. Ed. Engl.* **63**, e202401168 (2024).

35. L. Chen, X. Guan, X. Wu, H. Asakura, D. G. Hopkinson, C. Allen, J. Callison, P. J. Dyson, F. R. Wang, Thermally stable high-loading single Cu sites on ZSM-5 for selective catalytic oxidation of NH_3. *Proc. Natl. Acad. Sci. U.S.A.* **121**, e2404830121 (2024).

36. A. P. Kourounakis, D. Xanthopoulos, A. Tzara, Morpholine as a privileged structure: A review on the medicinal chemistry and pharmacological activity of morpholine containing bioactive molecules. *Med. Res. Rev.* **40**, 709–752 (2020).

37. H. Iino, J.-i. Hanna, Liquid crystalline organic semiconductors for organic transistor applications. *Polym. J.* **49**, 23–30 (2017).

38. S. Liu, T. Li, F. Shi, H. Ma, B. Wang, X. Dai, X. Cui, Constructing multiple active sites in iron oxide catalysts for improving carbonylation reactions. *Nat. Commun.* **14**, 4973 (2023).

39. A. G. Sergeev, A. Spannenberg, M. Beller, Palladium-catalyzed formylation of aryl bromides: Elucidation of the catalytic cycle of an industrially applied coupling reaction. *J. Am. Chem. Soc.* **130**, 15549–15563 (2008).

40. J. Gu, F. Zhao, K. N. Houk, Q. Lu, F. Liu, Computational determination of the mechanism of the Pd-catalyzed formation of isatoic anhydrides from o-haloanilines, CO, and CO_2. *Dalton Trans.* **50**, 14453–14461 (2021).

41. A. Mukherjee, S. Mahato, D. S. Kopchuk, S. Santra, G. V. Zyryanov, A. Majee, O. N. Chupakhin, Synthesis of α-amino carbonyl compounds: A brief review. *Russ. Chem. Rev.* **92**, RCR5046 (2023).

42. Y. Zhang, J. Vanderghinste, J. Wang, S. Das, Challenges and recent advancements in the synthesis of α,α-disubstituted α-amino acids. *Nat. Commun.* **15**, 1474 (2024).

43. G. Kresse, J. Furthmuller, Efficiency of ab-initio total energy calculations for metals and semiconductors using a plane-wave basis set. *Comp. Mater. Sci.* **6**, 15–50 (1996).

44. P. E. Blöchl, Projector augmented-wave method. *Phys. Rev. B* **50**, 17953–17979 (1994).

45. J. P. Perdew, K. Burke, M. Ernzerhof, Generalized gradient approximation made simple. *Phys. Rev. Lett.* **77**, 3865–3868 (1996).

46. J. P. Perdew, K. Burke, M. Ernzerhof, Generalized gradient approximation made simple. *Phys. Rev. Lett.* **78**, 1396–1396 (1997).

47. T. Risthaus, S. Grimme, Benchmarking of london dispersion-accounting density functional theory methods on very large molecular complexes. *J. Chem. Theory. Comput.* **9**, 1580–1591 (2013).

48. H. Jónsson, G. Mills, K. W. Jacobsen, in *Classical and Quantum Dynamics in Condensed Phase Simulations*, B. J. Berne, G. Ciccotti, D. F. Coker, Eds. (World Scientific, 1998), pp. 385-404.

49. S. Zheng, Y. Wang, C. Zhang, J. Liu, C. Xia, NHC-Pd complex-catalyzed double carbonylation of aryl iodides with secondary amines to α-keto amides. *Appl. Organomet. Chem.* **28**, 48–53 (2013).

50. M. Vico Solano, G. González Miera, V. Pascanu, A. K. Inge, B. Martín-Matute, Versatile heterogeneous palladium catalysts for diverse carbonylation reactions under atmospheric carbon monoxide pressure. *ChemCatChem* **10**, 1089–1095 (2018).

51. H. Du, Q. Ruan, M. Qi, W. Han, Ligand-free Pd-catalyzed double carbonylation of aryl iodides with amines to α-Ketoamides under atmospheric pressure of carbon monoxide and at room temperature. *J. Org. Chem.* **80**, 7816–7823 (2015).

52. B. Chen, F. Li, Z. Huang, T. Lu, G. Yuan, Stability or flexibility: Metal nanoparticles supported over cross-linked functional polymers as catalytic active sites for hydrogenation and carbonylation. *Appl. Catal. A Gen.* **481**, 54–63 (2014).

53. S. Maji, M. Roy, K. Shaikh, D. Adhikari, Organophotocatalytic dehydrogenative preparation of amides directly from alcohols. *Green Chem.* **25**, 8019–8025 (2023).

54. Y. Zheng, Y. Zhao, S. Tao, X. Li, X. Cheng, G. Jiang, X. Wan, Green esterification of carboxylic acids promoted by tert-butyl nitrite. *Eur. J. Org. Chem.* **2021**, 2713–2718 (2021).

55. J. Su, J.-N. Mo, X. Chen, A. Umanzor, Z. Zhang, K. N. Houk, J. Zhao, Generation of oxyphosphonium ions by photoredox/cobaloxime catalysis for scalable amide and peptide synthesis in batch and continuous-flow. *Angew. Chem. Int. Ed. Engl.* **61**, e202112668 (2022).

56. D. I. Tzaras, M. Gorai, T. Jacquemin, T. Arndt, B. M. Zimmermann, M. Breugst, J. F. Teichert, Site-selective copper(I)-catalyzed hydrogenation of amides. *J. Am. Chem. Soc.* **147**, 1867–1874 (2025).

57. K. P. Patel, E. M. Gayakwad, G. S. Shankarling, Graphene oxide: A convenient metal-free carbocatalyst for facilitating amidation of esters with amines. *New J. Chem.* **44**, 2661–2668 (2020).

58. A. Sarswat, R. Kumar, L. Kumar, N. Lal, S. Sharma, Y. S. Prabhakar, S. K. Pandey, J. Lal, V. Verma, A. Jain, J. P. Maikhuri, D. Dalela, Kirti, G. Gupta, V. L. Sharma, Arylpiperazines for management of benign prostatic hyperplasia: Design, synthesis, quantitative structure-activity relationships, and pharmacokinetic studies. *J. Med. Chem.* **54**, 302–311 (2011).

59. V. Vinayagam, S. K. Sadhukhan, D. V. Botla, R. R. Chittem, S. R. Kasu, T. V. Hajay Kumar, Mild method for deprotection of the *N*-benzyloxycarbonyl (N-Cbz) group by the combination of $AlCl_3$ and HFIP. *J. Org. Chem.* **89**, 5665–5674 (2024).

60. X. Chen, T. Chen, Q. Li, Y. Zhou, L. B. Han, S. F. Yin, Copper-catalyzed aerobic oxidative inert C-C and C-N bond cleavage: A new strategy for the synthesis of tertiary amides. *Chemistry* **20**, 12234–12238 (2014).

61. S. Ghinato, D. Territo, A. Maranzana, V. Capriati, M. Blangetti, C. Prandi, A fast and general route to ketones from amides and organolithium compounds under aerobic conditions: Synthetic and mechanistic aspects. *Chemistry* **27**, 2868–2874 (2021).

62. J. Zhao, J. Shi, Y. Li, Benzyne-mediated esterification reaction. *Org. Lett.* **23**, 7274–7278 (2021).

63. D. Xu, L. Shi, D. Ge, X. Cao, H. Gu, Platinum nanowires catalyzed direct amidation with aldehydes and amines. *Sci. China Chem.* **59**, 478–481 (2016).

64. A. R. Bayguzina, A. R. Lutfullina, R. I. Khusnutdinov, Synthesis of N-(adamantan-1-yl)carbamides by ritter reaction from adamantan-1-ol and nitriles in the presence of Cu-catalysts. *Russ. J. Org. Chem.* **54**, 1127–1133 (2018).

65. G. W. Wang, N. G. McCreanor, M. H. Shaw, W. G. Whittingham, J. F. Bower, New initiation modes for directed carbonylative C-C bond activation: Rhodium-catalyzed (3 + 1 + 2) cycloadditions of aminomethylcyclopropanes. *J. Am. Chem. Soc.* **138**, 13501–13504 (2016).

66. N. Wang, X. Zou, J. Ma, F. Li, The direct synthesis of N-alkylated amides via a tandem hydration/N-alkylation reaction from nitriles, aldoximes and alcohols. *Chem. Commun.* **50**, 8303–8305 (2014).

67. K. S. Sharma, N. Thadem, G. Pandey, Visible-light-induced secondary benzylic C(sp^3)-H functionalization for nucleophilic substitution: An intermolecular C-X (C-N, C-C, and C-Br) bond forming reaction. *J. Org. Chem.* **90**, 3384–3390 (2025).

68. M. Ayub Ali, S. M. A. Hakim Siddiki, K. Kon, K.-i. Shimizu, Fe^{3+}-exchanged clay catalyzed transamidation of amides with amines under solvent-free condition. *Tetrahedron Lett.* **55**, 1316–1319 (2014).

69. Y. He, C. Du, J. Han, J. Han, C. Zhu, J. Xie, Manganese-catalyzed anti-markovnikov hydroarylation of enamides: Modular synthesis of arylethylamines. *Chin. J. Chem.* **40**, 1546–1552 (2022).

70. Q. Li, D. D. Ma, J. Zhao, W. Wei, S. G. Han, X. T. Wu, R. Zou, Q. Xu, Q. L. Zhu, Modular synchronous synthesis of amides and α-Ketoamides realized by matching electrolysis-paired tandems. *Angew. Chem. Int. Ed. Engl.*, **64**, e202503440 (2025).

71. Y. Chen, S. Gu, Y. Zhao, Y. You, F. Ma, F. Zhu, S. Zhu, L.-G. Xie, Degradation of sulfur hexafluoride and its application in the synthesis of esters and amides. *Green Chem.* **27**, 2921–2930 (2025).

72. C. Y. Huang, A. G. Doyle, Electron-deficient olefin ligands enable generation of quaternary carbons by Ni-catalyzed cross-coupling. *J. Am. Chem. Soc.* **137**, 5638–5641 (2015).

73. K. Zhu, M. P. Shaver, S. P. Thomas, Chemoselective nitro reduction and hydroamination using a single iron catalyst. *Chem. Sci.* **7**, 3031–3035 (2016).

74. T. Truong, G. H. Dang, N. V. Tran, N. T. Truong, D. T. Le, N. T. S. Phan, Oxidative cross-dehydrogenative coupling of amines and α-carbonyl aldehydes over heterogeneous Cu-MOF-74 catalyst: A ligand- and base-free approach. *J. Mol. Catal. A Chem.* **409**, 110–116 (2015).

75. W. Wei, Y. Shao, H. Hu, F. Zhang, C. Zhang, Y. Xu, X. Wan, Coupling of methyl ketones and primary or secondary amines leading to α-ketoamides. *J. Org. Chem.* **77**, 7157–7165 (2012).

76. J. Y. Chen, H. Y. Wu, Q. W. Gui, X. R. Han, Y. Wu, K. Du, Z. Cao, Y. W. Lin, W. M. He, Electrochemical synthesis of α-Ketoamides under catalyst-, oxidant-, and electrolyte-free conditions. *Org. Lett.* **22**, 2206–2209 (2020).

77. X. Wang, Y. Li, N. Zhao, H. Liu, Y. Zhou, Copper-catalyzed synthesis of α-keto amides from sulfoxonium ylides. *J. Org. Chem.* **88**, 8268–8278 (2023).

78. F. Heaney, J. Fenlon, P. McArdleb, D. Cunninghamb, α-Keto amides as precursors to heterocycles-generation and cycloaddition reactions of piperazin-5-one nitrones. *Org. Biomol. Chem.* **1**, 1122–1132 (2003).

79. S. Singh, S. Popuri, Q. M. Junaid, S. Sabiah, J. Kandasamy, Diversification of α-ketoamides via transamidation reactions with alkyl and benzyl amines at room temperature. *Org. Biomol. Chem.* **19**, 7134–7140 (2021).

80. S. Das, S. Mondal, S. P. Midya, S. Mondal, E. Ghosh, P. Ghosh, Base-promoted tandem pathway for keto-amides: Visible light-mediated room-temperature amidation using molecular oxygen as an oxidant. *J. Org. Chem.* **88**, 14847–14859 (2023).

81. Z. Li, Y. Zhang, K. Li, Z. Zhou, Z. Zha, Z. Wang, Selective electrochemical oxidation of aromatic hydrocarbons and preparation of mono/multi-carbonyl compounds. *Sci. China Chem.* **64**, 2134–2141 (2021).

82. Q. W. Tan, P. Chovatia, M. C. Willis, Copper-catalysed synthesis of alkylidene 2-pyrrolinone derivatives from the combination of α-keto amides and alkynes. *Org. Biomol. Chem.* **16**, 7797–7800 (2018).

83. J. Ma, X. Cui, J. Xu, Y. Tan, Y. Wang, X. Wang, Y. Li, One-pot synthesis of α-ketoamides from α-keto acids and amines using ynamides as coupling reagents. *J. Org. Chem.* **87**, 3661–3667 (2022).

84. D. Chen, C. Cheng, S. Zeng, Y. Luo, J. Zhang, W. Deng, Z. Zeng, R. Wang, J. Xiang, $Cu(OAc)_2$ and acids promoted the oxidative cleavage of α-aminocarbonyl compounds with amines: Efficient and selective synthesis of 2-t-amino-2-imino-carbonyl and 2-amino-2-oxocarbonyl. *Tetrahedron Lett.* **61**, 151913 (2020).

85. T. S. Brunner, P. W. Roesky, Enantiopure amidinate complexes of lutetium. *J. Organomet. Chem.* **849-850**, 150–156 (2017).

86. J. Iley, R. Tolando, L. Constantino, Chemical and microsomal oxidation of tertiary amides: Regio- and stereoselective aspects. *J. Chem. Soc. Perkin Trans. 2*, 1299–1305 (2001).

87. J. Li, B. Cheng, X. Shu, Z. Xu, C. Li, H. Huo, Enantioselective alkylation of α-amino $C(sp^3)$−H bonds via photoredox and nickel catalysis. *Nat. Catal.* **7**, 889–899 (2024).

88. A. Welker, C. Kersten, C. Muller, R. Madhugiri, C. Zimmer, P. Muller, R. Zimmermann, S. Hammerschmidt, H. Maus, J. Ziebuhr, C. Sotriffer, T. Schirmeister, Structure-activity relationships of benzamides and isoindolines designed as SARS-CoV protease inhibitors effective against SARS-CoV-2. *ChemMedChem* **16**, 340–354 (2021).

89. F. Jin, G. Xu, F. Li, W. Cao, B. Liu, Y. Xu, Y. Chu, W. Song, P. Peng, K. Feng, Copper-mediated oxidative coupling of difluoromethyl bromides/chlorides with primary amines: Direct synthesis of α-ketoamides. *J. Org. Chem.* **90**, 11811–11817 (2025).

90. A. Ali, J. Wang, R. S. Nathans, H. Cao, N. Sharova, M. Stevenson, T. M. Rana, Synthesis and structure-activity relationship studies of HIV-1 virion infectivity factor (Vif) inhibitors that block viral replication. *ChemMedChem* **7**, 1217–1229 (2012).

91. R. J. Huang, T. G. Ong, R. J. Chein, Total synthesis of cassane-type diterpenoid pikrosalvin. *J. Chin. Chem. Soc.* **70**, 2127–2135 (2023).

92. S. Y. Wang, X. Liu, L. W. Meng, M. M. Li, Y. R. Li, G. X. Yu, J. Song, H. Y. Zhang, P. Chen, S. Y. Zhang, T. Hu, Discovery of indoline derivatives as anticancer agents via inhibition of tubulin polymerization. *Bioorg. Med. Chem. Lett.* **43**, 128095 (2021).

93. S. Kar, Y. Xie, Q. Q. Zhou, Y. Diskin-Posner, Y. Ben-David, D. Milstein, Near-ambient-temperature dehydrogenative synthesis of the amide bond: Mechanistic insight and applications. *ACS Catal.* **11**, 7383–7393 (2021).
